# Microbiome and mitogenomics of the chigger mite *Pentidionis agamae*: potential role as an *Orientia* vector and associations with divergent clades of *Wolbachia* and *Borrelia*

**DOI:** 10.1186/s12864-024-10301-6

**Published:** 2024-04-17

**Authors:** Hadil A. Alkathiry, Samia Q. Alghamdi, Amit Sinha, Gabriele Margos, Alexandr A. Stekolnikov, Abdulaziz N. Alagaili, Alistair C. Darby, Benjamin L. Makepeace, Jing Jing Khoo

**Affiliations:** 1 Imam Mohammad Ibn Saud Islamic University, Riyadh, Saudi Arabia; 2 Institute of Infection, Veterinary & Ecological Sciences, University of Liverpool, 146 Brownlow Hill, Liverpool, L3 5RF UK; 3 Department of Biology, Faculty of Science, Al-Baha University, P.O.Box1988, Al-Baha, 65799 Saudi Arabia; 4 New England Biolabs, Ipswich, Massachusetts 01938 USA; 5 National Reference Centre for Borrelia, Bavarian Health and Food Safety Authority, Veterinärstr. 2, Oberschleissheim, 85764 Germany; 6 Laboratory of Parasitic Arthropods, Zoological Institute of the Russian Academy of Sciences, Universitetskaya embankment 1, St. Petersburg, 199034 Russia; 7 Department of Zoology, King Saud University, Riyadh, Kingdom of Saudi Arabia

**Keywords:** Scrub typhus, Chiggers, Metagenomics, *Orientia*, *Wolbachia*, *Borrelia*, Mitochondrial genome, *Acomys dimidiatus*

## Abstract

**Background:**

Trombiculid mites are globally distributed, highly diverse arachnids that largely lack molecular resources such as whole mitogenomes for the elucidation of taxonomic relationships. Trombiculid larvae (chiggers) parasitise vertebrates and can transmit bacteria (*Orientia* spp.) responsible for scrub typhus, a zoonotic febrile illness. *Orientia tsutsugamushi* causes most cases of scrub typhus and is endemic to the Asia-Pacific Region, where it is transmitted by *Leptotrombidium* spp. chiggers. However, in Dubai, *Candidatus* Orientia chuto was isolated from a case of scrub typhus and is also known to circulate among rodents in Saudi Arabia and Kenya, although its vectors remain poorly defined. In addition to *Orientia*, chiggers are often infected with other potential pathogens or arthropod-specific endosymbionts, but their significance for trombiculid biology and public health is unclear.

**Results:**

Ten chigger species were collected from rodents in southwestern Saudi Arabia. Chiggers were pooled according to species and screened for *Orientia* DNA by PCR. Two species (*Microtrombicula muhaylensis* and *Pentidionis agamae*) produced positive results for the *htrA* gene, although *Ca*. Orientia chuto DNA was confirmed by Sanger sequencing only in *P. agamae*. Metagenomic sequencing of three pools of *P. agamae* provided evidence for two other bacterial associates: a spirochaete and a *Wolbachia* symbiont. Phylogenetic analysis of 16S rRNA and multi-locus sequence typing genes placed the spirochaete in a clade of micromammal-associated *Borrelia* spp. that are widely-distributed globally with no known vector. For the *Wolbachia* symbiont, a genome assembly was obtained that allowed phylogenetic localisation in a novel, divergent clade. Cytochrome c oxidase I (*COI*) barcodes for Saudi Arabian chiggers enabled comparisons with global chigger diversity, revealing several cases of discordance with classical taxonomy. Complete mitogenome assemblies were obtained for the three *P. agamae* pools and almost 50 SNPs were identified, despite a common geographic origin.

**Conclusions:**

*P. agamae* was identified as a potential vector of *Ca.* Orientia chuto on the Arabian Peninsula. The detection of an unusual *Borrelia* sp. and a divergent *Wolbachia* symbiont in *P. agamae* indicated links with chigger microbiomes in other parts of the world, while *COI* barcoding and mitogenomic analyses greatly extended our understanding of inter- and intraspecific relationships in trombiculid mites.

**Supplementary Information:**

The online version contains supplementary material available at 10.1186/s12864-024-10301-6.

## Introduction

Chiggers, the larval stage of trombiculid mites, are miniscule ectoparasites that feed on a wide range of terrestrial vertebrates and humans are incidental hosts for some species. The two main potential clinical impacts of chigger infestations are trombiculiasis (or “scrub itch”), which is an allergic dermatitis caused by hypersensitivity reactions to mite saliva [1], and acquisition of scrub typhus, which is caused by *Orientia* spp. These are obligate intracellular bacteria in the family Rickettsiaceae (order Rickettsiales) maintained in chiggers as vertically transmitted symbionts [2]. Scrub typhus is the more serious chigger-related condition, since the median mortality of the untreated disease in the Asia-Pacific region (where is it is caused by *Orientia tsutsugamushi*) is 6% [3]. Two other *Orientia* spp. are recognised although not yet formally described: *Candidatus* Orientia chiloensis [4], which has only been reported from Chile (~100 cases to date, none fatal), and *Candidatus* Orientia chuto, which is known from a single, nonfatal human case contracted in Dubai [5], but has also been detected in chiggers in Kenya [6] and wild rodents in Saudi Arabia [7]. Only two genera of chiggers are known to transmit *Orientia* spp. to humans; these are *Leptotrombidium* spp. across the Asia-Pacific (vectors of *O. tsutsugamushi*) [2] and *Herpetacarus* spp. in Chile (vectors of *Ca*. O. chiloensis) [8]. However, *Orientia* spp. have been found in several other chigger genera that are thought to maintain infection in wild hosts, including *Microtrombicula* spp. from Kenya, in which *Ca*. O. chuto was detected [6]. The chigger vector(s) of *Ca*. O. chuto in the Arabian Peninsula remain unknown.

In addition to *Orientia* spp., a number of other potentially pathogenic bacteria and viruses have been reported from chiggers in targeted surveys or 16S rRNA amplicon sequencing, including *Bartonella* spp., *Rickettsia* spp., *Borrelia* spp., *Anaplasma* spp., hantaviruses, and Dabie bandavirus (reviewed in [9]). Among the bacteria, the genera *Rickettsia* (family Rickettsiaceae) and *Anaplasma* (family Anaplasmataceae) are obligate intracellular organisms related to *Orientia*. They utilise a range of arthropod hosts (primarily ticks, mites, fleas, or lice for *Rickettsia* spp.; or predominantly ticks for *Anaplasma* spp.), and many species can be transmitted to humans, causing potentially severe disease. Major pathogens of medical significance include *Rickettsia rickettsii* (aetiological agent of Rocky Mountain spotted fever) and *Anaplasma phagocytophilum* (agent of human granulocytic anaplasmosis), both of which can be fatal if not treated promptly [10]. The spotted fever group of rickettsiae is transmitted mainly by ticks, while one member of the more recently defined transitional group of rickettsiae, *Rickettsia akari* (agent of rickettsialpox), is vectored by gamasid mites [11]. Detection of *Rickettsia* spp. DNA in chiggers has been reported from geographically diverse locations [12–15], but a role in transmission of rickettsiae to vertebrates has not been established, and at least some of these rickettsiae may be arthropod-specific symbionts. Interestingly, DNA of both *Rickettsia* spp. [16] and *A. phagocytophilum* [17] has been amplified from unfed chiggers, which is strong evidence for vertical transmission and long-term symbiotic relationships.

The Gram-negative, facultatively intracellular genus *Bartonella* (order Hyphomicrobiales) comprises bacteria that infect vertebrate erythrocytes and are highly prevalent in micromammals, especially rodents. It is generally accepted that they are maintained in the mammalian populations by arthropod vectors (sandflies for *Bartonella bacilliformis*; and fleas, lice, and perhaps ticks for other species) [18]. Of the many species within the genus, *B. bacilliformis, Bartonella quintana,* and *Bartonella henselae* are the most important human pathogens and only the latter (agent of cat-scratch fever) is zoonotic [19]. Other zoonotic, rodent-associated *Bartonella* spp. have been reported from various chigger species in Southeast Asia, but data supporting a vector role for them in human disease remain circumstantial [20, 21].

The genus *Borrelia* includes the causative agents of Lyme borreliosis [also referred to as Lyme disease (LD) in the USA] and relapsing fever (RF) borreliosis [22]. These spirochaetal bacteria are commonly maintained in natural transmission cycles by tick vectors, and rodents are important reservoirs for many of the human-pathogenic species [23]. While evidence for *Borrelia* spp. in both trombiculid and gamasid mites has been reported, their vector status remains questionable [24–26]. One group of spirochaetal bacteria of uncertain taxonomic status has been found previously in the tissues of small mammals, but a vector for this micromammal-specific clade has yet to be identified [27, 28].

Lastly, vertically-transmitted endobacteria that do not infect vertebrates (predominantly *Wolbachia*, *Rickettsiella* and *Cardinium*) have been detected in several chigger microbiome studies (reviewed in [9]), all of which were performed on Asian species of trombiculids. However, any potential phenotypic effects of these symbionts (such as cytoplasmic incompatibility) [29], or inhibition or enhancement of pathogen transmission [30] by chiggers, remain unexplored.

Compounding these knowledge gaps regarding the vector biology and microbiome of chiggers, the population genetics and molecular systematics of trombiculid mites remain in their infancy. Only two nuclear genomes (both from *Leptotrombidium* spp. [31, 32]) and five mitogenomes (three from *Leptotrombidium* spp. [33]) for chiggers are publicly available. Most other genetic data for trombiculid mites consist of cytochrome *c* oxidase I (*COI*) DNA barcodes, but even these display poor geographic representation, with most being obtained from Southeast Asia [34], East Asia [35] and Europe [36], with none available for the Middle East.

Here, we present evidence that the chigger *Pentidionis agamae* may be a vector of *Ca*. O. chuto in Saudi Arabia. Moreover, applying a metagenomic approach, we obtained complete mitogenomes from this species and place it in the phylogenetic context of other Saudi Arabian chigger species, as well as trombiculid diversity worldwide, through analysis of *COI* barcodes. Finally, sequences from two additional, non-*Orientia* bacterial associates of *P. agamae* are shown to represent a poorly described, micromammal-associated *Borrelia* sp. and a member of a novel, deep-branching clade of *Wolbachia* symbionts.

## Results

### Chigger sampling

In total, 156 rodents were captured, belonging to six different species: *Acomys dimidiatus*, *Meriones rex*, *Mus musculus*, *Ochromyscus yemeni* and *Rattus rattus* (Additional file 1: Table S1). A total 7,329 chiggers were recovered from 27 and 55 rodents in ‘Asir and Al-Bahah provinces, respectively. Of these, 4,226 chiggers belonging to 20 trombiculid species were identified (Table 1). The remaining chiggers were excluded from the study as they were damaged, or the important identifying features were absent.

**Table 1:** Chigger species and numbers found at two sampling locations in Saudi Arabia

**Chigger species**	**Subfamily and tribe**	**Province**	
		**‘Asir**	**Al-Bahah**
*Schoengastiella hypoderma*	Gahrliepiinae	-	6
*Walchia parvula*	Gahrliepiinae	1	-
*Odontacarus thesigeri*	Leeuwenhoekiinae	-	3
*Ascoschoengastia browni*	Trombiculinae: Schoengastiini	32	112
*Helenicula lukshumiae*	Trombiculinae: Schoengastiini	171	29
*Schoutedenichia asirensis*	Trombiculinae: Schoengastiini	5	1
*Schoutedenichia originalis*	Trombiculinae: Schoengastiini	17	17
*Schoutedenichia saudi*	Trombiculinae: Schoengastiini	346	153
*Schoutedenichia zarudnyi*	Trombiculinae: Schoengastiini	227	878
*Ericotrombidium caucasicum*	Trombiculinae: Trombiculini	411	5
*Ericotrombidium kazeruni*	Trombiculinae: Trombiculini	735	-
*Microtrombicula abyssinica*	Trombiculinae: Trombiculini	2	-
*Microtrombicula felis*	Trombiculinae: Trombiculini	28	-
*Microtrombicula hoogstraali*	Trombiculinae: Trombiculini	-	1
*Microtrombicula hyracis*	Trombiculinae: Trombiculini	2	-
*Microtrombicula muhaylensis*	Trombiculinae: Trombiculini	305	157
*Microtrombicula peltifera*	Trombiculinae: Trombiculini	-	8
*Microtrombicula saperoi*	Trombiculinae: Trombiculini	1	-
*Microtrombicula traubi*	Trombiculinae: Trombiculini	-	15
*Pentidionis agamae*	Trombiculinae: Trombiculini	87	471

### *Ca*. O. chuto in *P. agamae*

**Fig. 1:**
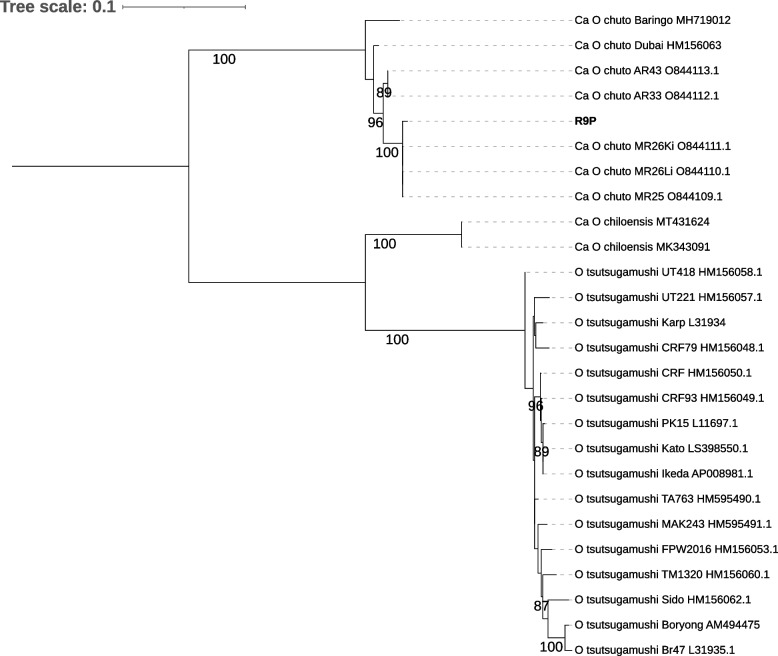
Maximum-likelihood tree of *Orientia htrA* sequence detected from *P. agamae* R9P pool (in bold) from Saudi Arabia. Tree was constructed based on 1,500 nucleotide positions and the best-fit model according to BIC: K3Pu+F+I+R2. The tree was rooted mid-point. Ultra-fast bootstrap values above 80 are displayed on branches

*Orientia* screening by qPCR (*traD*) and nested PCR (*htrA*) was performed on 165 pools of chiggers, consisting of 3,286 individuals (Additional file 1: Table S2). A single pool each of *P. agamae* (R9P) and *M. muhaylensis* (R19M) – both obtained from *A. dimidiatus* hosts in ‘Asir province - yielded positive amplification in the *traD* qPCR assay. However, Sanger sequencing of the *htrA* nested PCR product only produced a high-quality sequence from R9P for further analyses. The *htrA* sequence from R9P formed a single (100% bootstrap-supported) clade with the *htrA* sequences from *Ca* O. chuto reported from the tissues of *A. dimidiatus* captured from ‘Asir Province (MR25, MR26Ki, MR26Li) in our previous study [7], and the sequences from Saudi Arabia remained in a single (96% bootstrap supported) clade distinct from *Ca* O. chuto from Dubai, United Arab Emirates (UAE) (Fig. 1). Genetic pairwise distance calculated between the *Ca.* O. chuto *htrA* sequences (based on 659 bp) from Saudi Arabia also showed that R9P *htrA* is more closely related to the sequences from ‘Asir Province (MR25, MR26Ki, MR26Li: pairwise distance = 0.003) than sequences from Al-Bahah Province (AR33 and AR43: pairwise distance = 0.017).

Following this finding, the R9P pool of *P. agamae* was subjected to metagenomic sequencing using Illumina technology to obtain additional genes for comparison with the sequenced culture isolate of *Ca*. O. chuto str. Dubai [5]. Since *P. agamae* is a potential vector of *Ca* O. chuto based on the *traD* qPCR and *htrA* nested-PCR, an additional two pools of *P. agamae*, Pa1 and Pa2, obtained from the ‘Asir region in a previous sampling effort [37], were also subjected to metagenomic sequencing. An overview of the microbiome associated with each of the chigger pools are presented in Kronagrams generated from the Kraken2 output at a confidence threshold of 0.1 (Additional files 2, 3 and 4). In general, contigs assigned as Bacteria constituted a small proportion (0.2-5%) of all contigs classified, with members of *Pseudomonadota*, *Terrabacteria* and the FCB group bacteria dominating the microbiome. This dataset likely represents the microbiome of the chiggers and the associated animal host skin from which the mites were removed.

The Kraken2 assignment found contigs assigned to *Orientia* (Additional file 1: Table S3). A single contig from R9P overlapped 137 bp at the 3’ end of the *htrA* sequence from Sanger sequencing at 100% identity, indicating that the contig did not contain the full-length coding sequence of the gene. We did not find the *htrA* sequence from the contigs from Pa2; however, BLASTn analyses showed 99%-100% matches of the contigs to various *O. tsutsugamushi* strains (Additional file 1: Table S4). Five of these contigs only had matches to *Orientia* species. Phylogenetic analyses of the only contig with matches to other bacteria also placed the contig within a clade of other *Orientia* sequences (Additional file 1: Fig. S1). Diamond BLASTx revealed matches to a number of *O. tsutsugamushi* proteins, namely dihydrolipoyl dehydrogenase, toprim domain protein, conjugal transfer protein TraN, transposase and two different hypothetical proteins (Additional file 1: Table S4). None of the contigs from *P. agamae* pool Pa1 were verified as *Orientia* sequences from BLASTn analyses.

### *Wolbachia* and *Rickettsia*

**Fig. 2:**
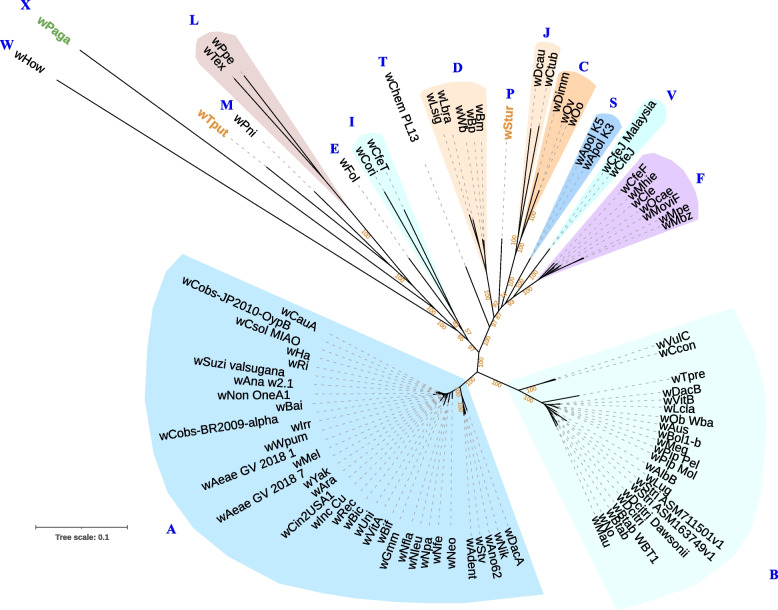
Maximum-likelihood phylogeny based on the concatenated alignments of 32 single copy orthologs (4,365 amino acid sites) from *Wolbachia* using a partitioned best-fit model for each ortholog. Letters represent the major clades or supergroups; *w*Paga (in green type) constitutes new supergroup X and is distinct from the symbionts of *Tyrophagus putrescentiae* (*w*Tput) and *Syringophilopsis turdi* (*w*Stur) (in orange type)

Contigs assigned to *Wolbachia* were also found in all three pools (Additional file 1: Table S3), with over 200 different contigs identified in Pa2. An improved *Wolbachia* metagenome-assembled genome (MAG) was obtained from this pool by mapping the short reads to metaSPAdes and Megahit-assembled contigs, and reassembling the mapped reads using metaSpades. This workflow resulted in a new draft assembly with BUSCO improvement from 60.4% to 78.8%. Maximum likelihood phylogeny placed this assembly, which we designate as *w*Paga, in its own clade (new supergroup X – bootstrap 100), which was close to the more divergent clades, including supergroups W, M, L, E, and I (Fig. 2). As genome assemblies for other *Wolbachia* symbionts from acariform mites (the mould mite *Tyrophagus putrescentiae* [38] and the quill mite *Syringophilopsis turdi* [39]) were made available recently on NCBI, we included these in the phylogenomic analysis. We determined that they were both very distinct from *w*Paga, with *w*Tput from *T*. *putrescentiae* displaying closer affinities with supergroup M (Fig. 2), whereas *w*Stur from *S. turdi* is a member of a distinct, more distant supergroup (P), as previously reported [39].

Multiple *Wolbachia* have been observed in some arthropod hosts; for instance *w*AlbA and *w*AlbB in the mosquito *Aedes albopictus*. In the blobplot analysis of *Wolbachia* MAG from *P. agamae*, all *Wolbachia* contigs were observed to cluster tightly around a median coverage (Additional file 1: Fig. S2), indicating the presence of a single *Wolbachia* genome. Further, the BUSCO analysis did not show a high rate of duplication of core *Wolbachia* genes, which can typically be observed in MAGs containing more than one *Wolbachia* (an example being the *Wolbachia* symbionts of the sheep ked, *Melophagus ovinus* [40]). As further quality check to ensure that the *w*Paga placement in the phylogenomic tree represents its true genetic divergence from other *Wolbachia* rather than being an artefact caused by combining MAGs originating from *Wolbachia* of different known supergroups, individual phylogenetic trees of each of the 32 single copy orthologs were inspected carefully. In these trees, the *w*Paga ortholog was always placed separate from all known supergroups (Additional file 5). A comprehensive sequence similarity comparison of *w*Paga sequences against genomes from all supergroups demonstrated their median percentage identity to be 78% over a median query coverage of only 26% (Additional file 1: Fig. S3a). This is in contrast to 97% median percent identity over 76% of query sequence from known supergroup A *Wolbachia w*DacA and supergroup B *Wolbachia w*DacB (Additional file 1: Fig. S3b and c). Interestingly, the sequence similarity patterns of *w*Paga were more similar to those of the highly diverged supergroup L *Wolbachia, w*Tex (Additional File 1: Fig. S3d), further supporting the hypotheses that the *w*Paga sequences represent another highly diverged, novel *Wolbachia*.

A number of contigs were also assigned as *Rickettsia* (Additional file 1: Table S3). However, further verification with BLASTn analyses revealed that most of these contigs either had no match to any existing sequences in GenBank, or matched with *Rickettsia* sequences with low percentage identity (<95%, data not shown), suggesting the presence of more genetically distant *Rickettsiales* bacteria.

### Detection of a micromammal-associated *Borrelia* sp. in *P. agamae*

**Fig. 3:**
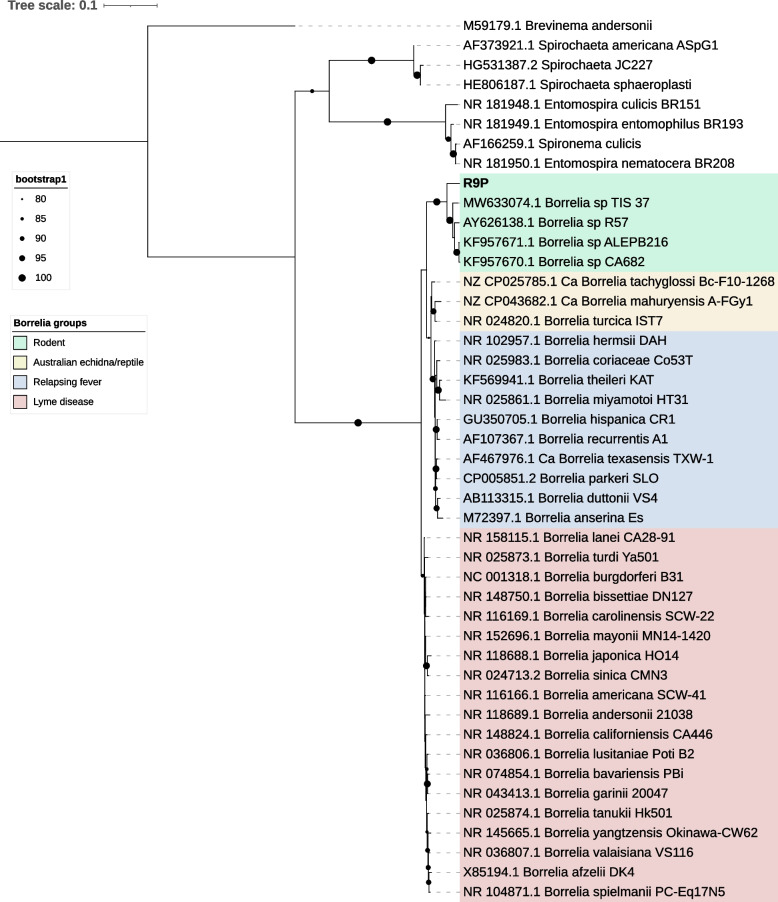
Maximum-likelihood tree of spirochaete *16S rRNA* sequence detected from *P. agamae* R9P pool (in bold) from Saudi Arabia. Tree was constructed with 1,866 nucleotide sites and the best-fit model according to BIC was TIM3+F+R3. Ultra-fast bootstrap values above 80 are indicated with black circles on the branches. The tree was rooted mid-point

**Fig. 4:**
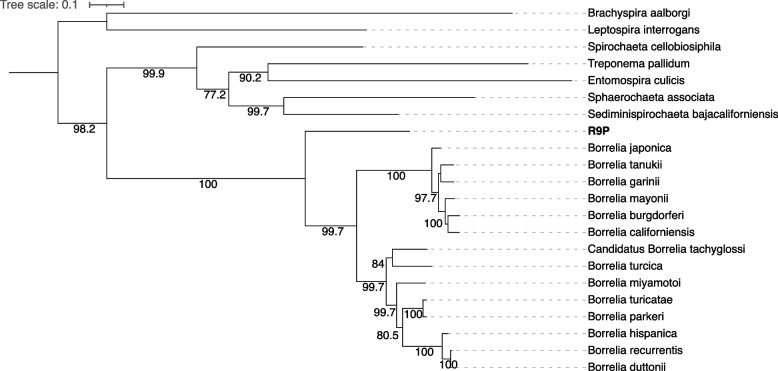
Maximum-likelihood tree of Spirochaetia including *P. agamae* (in bold) based on 3,669 bp of concatenated *clpX*, *recG* and *uvrA* sequences, with best-fit model determined for each gene separately. The tree was rooted mid-point. Accession numbers for MLST gene sequences used in analyses were given in Table S5 (Additional file 1)

All three *P. agamae* pools had contigs assigned as *Borrelia* (Additional file 1: Table S3). We recovered sequences for 16S rRNA and several genes from the *Borrelia* multi-locus sequence typing (MLST) scheme [41] - *clpX*, *recG* and *uvrA* - from R9P contigs, which were used to construct phylogenetic trees with other published spirochaete sequences from GenBank. We were unable to recover these genes from Pa1 and Pa2. In the 16S rRNA phylogenetic tree (Fig. 3), the *Borrelia* sp. from R9P clustered with *Borrelia* spp. previously reported from micromammals (mainly rodents), namely *Borrelia* sp. isolates R57 [42], BRAUS (TIS 37), CA682, and ALEPB216 [27, 28]. Since MLST gene sequences for the rodent group *Borrelia* spp. are not currently available, *Borrelia* sp. R9P forms a sister clade to other known *Borrelia* spp. from the LD and RF groups in the phylogenies based on the concatenated matrix of *clpX*, *recG* and *uvrA* (Fig. 4). Phylogenetic trees constructed from the individual MLST genes (Additional file 1: Fig. S4) also showed consistent topology with the phylogeny of the concatenated matrix, indicating that the presence of single strain of *Borrelia* sp. in this sample.

### Mitochondrial assembly

Circularised mitochondrial genomes were assembled separately from Pa1 and Pa2 (14,755 bp). The MitoZ pipeline produced a linear mitochondrial assembly for R9P, which was then circularised (14,753 bp) with an additional step based on overlapping sequences at the end of the linear assembly. Both assemblies from Pa2 and R9P appeared to be almost identical to Pa1 (Additional file 1: Fig. S5), with 99.92% and 99.76% identity, respectively. The base composition of the mitochondrial genomes was approximately 45% (A), 25% (T), 10% (C), and 20% (G).

**Fig. 5:**
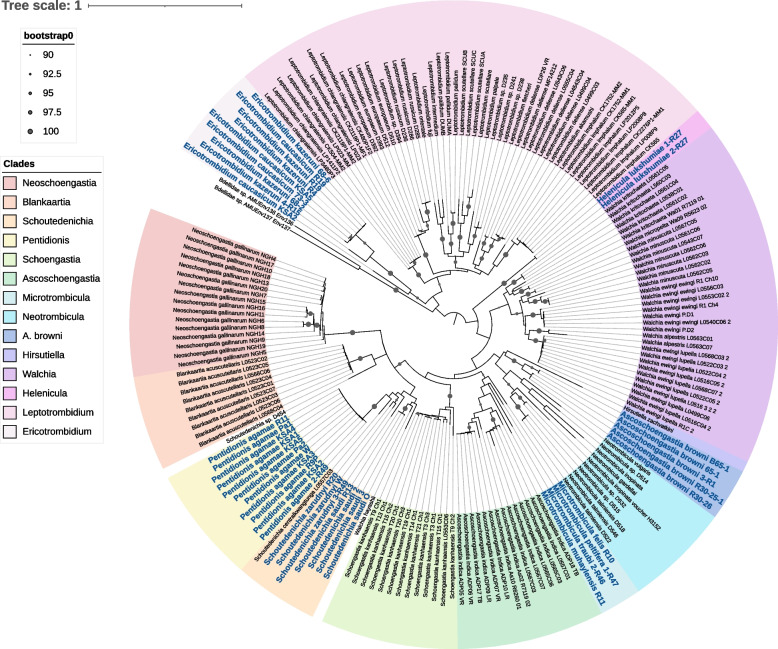
Maximum likelihood tree based on cytochrome oxidase I (*COI*) barcodes of Trombiculidae. Sequences generated in this study were highlighted in blue. Phylogeny was constructed based on 397 nucleotide positions and best-fit model according to BIC: TIM+F+I+G4. Ultrafast bootstrap values between 90 and 100 are indicated. *Bdellidae* sp. was used as an outgroup

Maximum likelihood phylogeny based on a partial *COI* gene fragment, combining data from the mitogenomic assemblies and additional *COI* PCR products from archived specimens, placed *P. agamae* in a single clade with *Schoutedenichia centralkwangtunga* (KY971498.1) from Laos and *Walchia hayashii* (NC010595.1) from Japan, with a bootstrap value of 94 (Fig. 5). This is surprising, as *Walchia* belongs to a different subfamily (*Gahrliepiinae*) than *Pentidionis* and *Schoutedenichia* (*Trombiculinae*), and the two latter genera belong to different tribes (Trombiculini and Schoengastiini, respectively) [43, 44].

Further inconsistencies of species placements were observed in this phylogeny. For instance, *W. hayashii* (represented by a complete mitogenomic assembly but lacking an accompanying publication) did not cluster within the clade containing the other *Walchia* spp. (all from Southeast Asia), and *Schoutedenichia* sp. D454 (OQ924405.1) from Albania seemed more closely related to *Blankaartia acuscutellaris* from Laos instead of *S. centralkwangtunga.* To provide the first molecular taxonomic data for chiggers from the Middle East, we generated *COI* barcodes for *Ericotrombidium caucasicum*, *E. kazeruni*, *Ascoschoengastia browni*, *Microtrombicula felis*, *M. peltifera*, *M. traubi*, *M. muhaylensis*, *Schoutedenichia zarudnyi*, *S. saudi*, and *Helenicula lukshumiae*, which were described from Saudi Arabia in our previous studies [7, 45]. For *Ericotrombidium* spp., *Microtrombicula* spp., and *Helenicula* spp., these were the first barcodes available for each genus and comparisons with congeneric species were thus not possible, although in each case, the genus formed a monophyletic group (Fig. 5). However, while the two *Schoutedenichia* spp. from Saudi Arabia clustered with *S. centralkwangtunga*, *A. browni* displayed closer affinities with *Hirsutiella zachvatkini* from Poland than to *Ascoschoengastia indica* from Thailand/Laos. Interestingly, the *H. lukshumiae* specimens were placed on a deep branch despite the classification of *Helenicula* in the tribe Schoengastiini with *Schoutedenichia* and *Ascoschoengastia* (Fig. 5. Additional file 1: Table S1). Nevertheless, the placements of *Ericotrombidium* and *Hirsutiella* appear to conform with the current classification system of chiggers based on larval morphology: (i) clustering of *Ericotrombidium* with *Leptotrombidium* (the former genus was described as a subgenus of the latter); (ii) clustering of *H. zachvatkini* with *Neotrombicula* (*Hirsutiella* is considered as a subgenus of *Neotrombicula* by some authors [46, 47]); and (iii) clustering of *A. indica* with *Microtrombicula* (*Ascoschoengastia* and *Microtrombicula*, although they belong to different tribes, in fact differ from each other by a single trait – trichobothria that are expanded in the former genus and flagelliform in the latter [48]). The affinity between two chigger species, namely *N. gallinarum* (tribe Schoengastiini) and *B. acuscutellaris* (tribe Trombiculini), which prefer avian hosts despite belonging to strikingly different genera and different tribes, was also noteworthy.

**Fig. 6:**
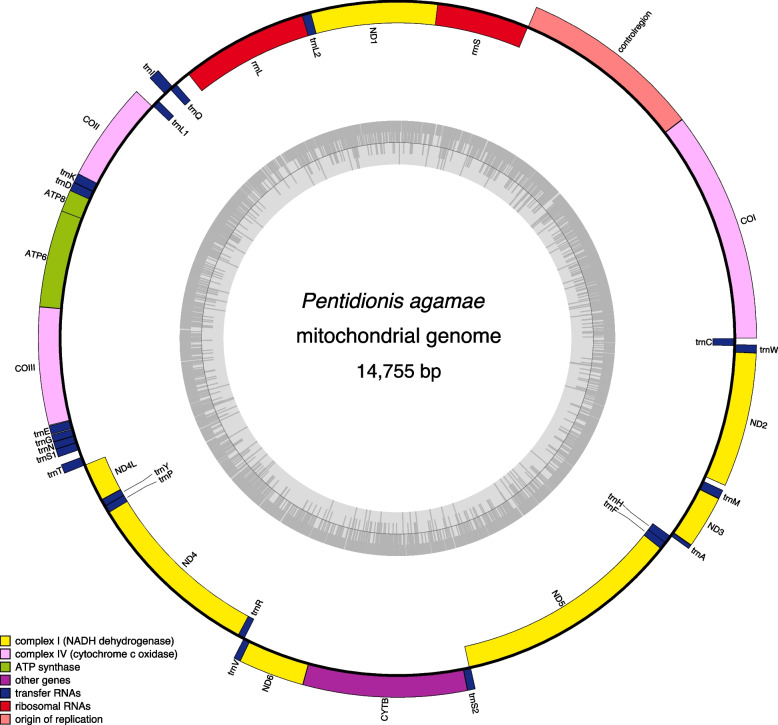
Mitochondrial genome from *P. agamae*. Outer ring shows CDS on the (+)-strand; inner ring shows CDS on the (-)-strand. The grey circle represents the GC content from low to high (outermost to innermost) and the circle inside the plot indicates the 50% threshold

Annotation of the assembly from Pa1 yielded thirteen protein CDS, two rRNAs and sixteen tRNAs (Fig. 6, Additional file 1: Fig. S6). We were able to identify the six missing tRNAs (*trnL1*, *tnrL2*, *trnA*, *tnrR*, *trnG* and *trnV*) by manually inspecting the conserved anti-codon regions in the alignments between the current assembly and the other five available mitochondrial assemblies from trombiculid mites (Additional file 1: Fig. S7). However, their predicted secondary structures appeared to have no T-arms and hence lack the typical clover leaf structure, or appeared to be extremely truncated (for *trnA*).

**Fig. 7:**
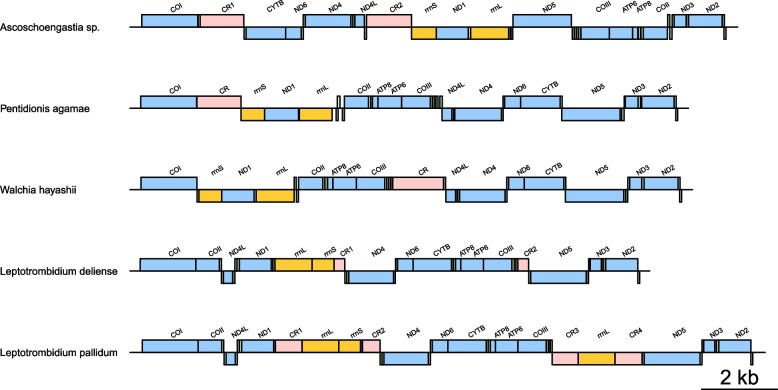
Mitochondrial genomes of trombiculid mites. The assembly for *P. agamae* was generated from the current study. Annotations for tRNAs are not shown in the figure. Blocks above the black line indicate genes on the (+)-strand, while blocks under the black line indicate genes on the (-)-strand. Blue blocks indicate the positions for protein CDS. Yellow blocks indicate the positions for rRNA. Pink blocks indicate the positions of control regions. Genomes were linearised at the position of the *COI* gene to facilitate comparisons

Relative to Pa1, 12 SNPs were observed in Pa2 and 36 SNPs (including two deletions) were detected in R9P. The SNPs in the Pa2 assembly were found in *trnT*, as well as in the* COI*, *CYTB* and *ND5* genes, causing non-synonymous substitutions in these protein CDS (Additional file 1: Table S6). A single non-synonymous substitution was observed in *COI*, while two non-synonymous substitutions were found each for *CYTB* and *ND5*. When gene arrangements were analysed, the mitochondrial genome from *P. agamae* displayed closest synteny to *W. hayashii*, with rearrangement of the positions of the control region and *trnQ* (Fig. 7). The control region for *P. agamae* lies upstream of *rrnS*, and *trnQ* lies downstream of *rrnL*. Unlike the mitochondrial genomes for *Ascoschoengastia* sp. TATW-1, *Leptotrombidium deliense* or *Leptotrombidium pallidum*, there was no duplication of any mitochondrial genes in *P. agamae*.

## Discussion

The ecology of *Ca* O. chuto has remained enigmatic since its discovery over a decade ago. The endemic region of this pathogen is potentially vast, with evidence of circulation across the Arabian Peninsula [5, 7], East Africa [6], and perhaps West Africa [49]. Despite this wide range, only one human case of scrub typhus caused by this species has been reported, which was contracted in Dubai [5]; however, no studies on *Ca*. O. chuto in chiggers or non-human vertebrate hosts in the UAE have been published to date. Following the publication of details of the clinical isolate of *Ca*. O. chuto from Dubai [5], pathogen DNA was detected in one pool of *Microtrombicula* spp. chiggers (of five pools from multiple host species screened), which was obtained from a Natal multimammate mouse (*Mastomys natalensis*) in Baringo county, Kenya [6]. More recently, *Ca*. O. chuto DNA was amplified from the tissues of 7.3% (*n* = 82) rodents (Eastern spiny mice - *Acomys dimidiatus*, or Wagner’s gerbil - *Dipodillus dasyurus*) trapped in ‘Asir and Al-Bahah provinces of Saudi Arabia [7]. Most of the positive rodents lacked chigger infestations, but chiggers of five species (*E. caucasicum, E. kazeruni, S. saudi, S. zarudnyi,* and *M. hoogstraali*) were obtained from two infected individuals and were shown to be negative for *Orientia* DNA.

The current study, in which a much more extensive collection of Saudi chiggers was screened, represents the first report of *Ca*. O. chuto DNA from potential vector species from the Arabian Peninsula (albeit >1,500 km distant from the clinical case reported from Dubai). Since the *htrA* PCR amplicon from the pool of *M. muhaylensis* failed to provide a high-quality sequence, the apparent positive result for this chigger species remains provisional. However, it is noteworthy that this species is in the same genus as the positive chigger pool reported from Kenya [6]. For *P. agamae*, molecular evidence for infection with *Ca*. O. chuto was obtained using Sanger sequencing of a *htrA* nested PCR product and metagenomic sequencing via Illumina short-read technology. Unfortunately, no additional *Orientia* genes could be assembled using the Illumina data from *P. agamae* pool R9P to obtain further phylogenetic information for comparison with the Dubai isolate. This means that current data for *Ca*. O. chuto in Saudi Arabia is comprised of only six sequences from a single gene [7], and the Kenyan data by two gene sequences (16S rRNA and *htrA*) from a single sample [6]. Nevertheless, the *htrA* sequences exhibit differences that correspond to geographic distance, with the R9P sequence from *P. agamae* collected in ‘Asir clustering with sequences from rodents obtained from the same province, and genetic distances increasing stepwise compared with sequences from Al-Bahah province, UAE, or Kenya, respectively. The lack of other *Orientia* sequence data in pool R9P suggests a very low level of *Ca*. O. chuto DNA in this sample, perhaps representing only a single positive chigger; neither can we rule out traces of host-derived pathogen DNA from mite mouthparts or gut contents in the absence of systemic chigger infection. Future studies could attempt to obtain additional *Orientia* genome data by sequence capture with DNA extracts from human samples and chigger specimens, which has been performed successfully using specific probes for *O. tsutsugamushi* [50].

Interestingly, while an *htrA* sequence could not be recovered from the metagenomic dataset for another *P. agamae* pool, Pa2, several other *Orientia* genes were identified in this sample. These had closest matches to *O. tsutsugamushi* sequences, especially the Karp-like strain UT176, which is a clinical isolate from Thailand [51]. However, genes from the multi-locus sequence typing scheme for *O. tsutsugamushi* were not assembled and caution is needed in interpreting these data as evidence of *O. tsutsugamushi* in Saudi Arabia, as the only *Ca*. O. chuto genome assembly available (str. Dubai) is incomplete [5]. Notwithstanding this limitation, the detection of *Orientia* sequences in this second pooled DNA sample from *P. agamae* adds to the evidence that this chigger species may act as a vector, at least between wild hosts. Unfortunately, in common with other chigger species in the Middle East, *P. agamae* is poorly studied with limited host records. Prior to our rodent studies in Saudi Arabia, *P. agamae* was only known from agamid lizard hosts in the Persis region of Iran [52] and around Lake Tiberias (Galilee) [53], although it is widespread on *A. dimidiatus* in both ‘Asir [37] and Al-Bahah [45] provinces. Whether *P. agamae* could bite humans and act as a clinically-relevant scrub typhus vector is an important open question, especially as the mountainous regions of southwest Saudi Arabia are popular destinations for tourists seeking cooler temperatures in the summer months. With respect to the origin of the only confirmed case of scrub typhus in the Middle East, limited data are available on the chigger fauna of UAE, with four chigger species reported recently from a very small sample of *A. dimidiatus* (*n* = 3) [54]. However, *P. agamae* was not present among these.

In the past five years, interest in the trombiculid mite microbiome has blossomed on the back of technological advances that have enabled 16S rRNA amplicon sequencing studies on low-input DNA samples. The current study constitutes the first genuine metagenomic analysis of a trombiculid mite since the publication of the *Leptotrombidium deliense* genome [31], thus providing the potential to obtain multiple gene sequences or even genome assemblies for members of the chigger microbiome. Here, we found a *Wolbachia* symbiont of *P. agamae* (*w*Paga) to be sufficiently represented to allow a genome assembly and phylogenomic analysis. It is important to note that single gene trees for *Wolbachia* are not always congruent, thereby leading to the move towards MLST trees built on supermatrices of multiple genes [55]. However, the widely used MLST scheme for *Wolbachia* has significant limitations [56]. Whole genome-based trees represent a superior extension of the same paradigm, utilizing the maximum sequence information available.

*Wolbachia* has been detected previously from trombiculid mites in Southeast Asia and East Asia using 16S rRNA amplicon sequencing [16, 57], but the use of a single conserved gene has precluded robust phylogenetic placement. Our data locate *w*Paga firmly among the early-branching clades of *Wolbachia* that have been poorly studied compared with the ubiquitous, so-called “pandemic” supergroups (A and B) [58], but it is sufficiently distinct to constitute the first member of a new supergroup. Unfortunately, we did not recover the 16S rRNA sequence from *w*Paga, but its position on a long branch is consistent with that of a previous reported symbiont from *Leptotrombidium scutellare* in Japan [16]. It has been hypothesized that *Wolbachia* evolved in the soil milieu [59] through associations with parasitic nematodes of plants (supergroup L [60]) or saprotrophic flies (W [61]), and may have been horizontally transmitted via plants, honeydew, and/or insect carcasses to other hosts of early-branching *Wolbachia* clades including the banana aphid (M), springtails (E), oribatid mites (E), and fleas (I) – the latter being detritivorous in the larval stage. In accordance with this model, the free-living lifecycle of trombiculid mites proceeds underground, where the nymphal and adult stages predate small edaphic arthropods or their eggs. We also assigned a phylogenetic placement to another *Wolbachia* symbiont of mite origin, *w*Tput from *T. putrescentiae*, which was close to supergroup M but may be a member of another novel clade. While renowned as a pest of stored foodstuffs, *T*. *putrescentiae* is also common in outdoor agricultural biomes [62] and is likely to share habitats with other hosts of the non-pandemic *Wolbachia* clades listed above. Even though hundreds of *Wolbachia* strains have been discovered, the true extent of *Wolbachia* diversity remains unexplored. Most of the commonly discovered *Wolbachia* belong to supergroups A and B, but as new orders of arthropods (such as mites and fleas), and nematodes (including non-filarial groups) are being analysed, distinct and more diverse *Wolbachia* are being discovered, which highlights the need for a wider surveys of *Wolbachia* from under-sampled host phyla.

In addition to *Wolbachia*, *Borrelia* spp. have been reported from trombiculid mites from several locations worldwide. Spirochaetes of the LD clade have been detected molecularly in harvest mites (*Neotrombicula autumnalis*) in Europe [24] and this chigger has been shown to acquire borreliae experimentally from infected rodents [26]. There is also some evidence for vertical transmission of LD borreliae in harvest mites [24, 26], while unassigned *Borrelia* spp. 16S rRNA sequences have been detected at high prevalence in chiggers collected from wild micromammals in Thailand [57, 63]. In the current study, we were able to acquire multiple gene sequences for a chigger-associated *Borrelia* spp. for the first time, allowing robust phylogenetic classification. Surprisingly, the sequences associated with *P. agamae* were not of LD or RF *Borrelia* spp. origin but belonged to a clade associated with rodents and shrews previously reported from Spain [42], California [28], and New South Wales [27]. On the basis of 16S rRNA and *groEL* gene sequences, this clade (originally described from Spain as isolate R57) has been known to be distinct from the LD and RF groups for nearly two decades, but its biology has remained enigmatic. Importantly, it has never been detected in arthropods or mammalian blood, but only ear punch biopsies. Our data suggest that chiggers (many species of which have a predilection for the pinna and ear canal as feeding sites) [64] may be the vector for this micromammal clade of borreliae. While we cannot rule out that the *Borrelia* spp. DNA is an incidental finding due to ingestion of host tissue fluid by chiggers, the fact we could assemble several genes from the organism coupled with the absence of prior PCR detection in hard ticks that are often contaminated with host skin, renders this possibility less likely.

In the past five years, molecular barcoding (primarily based on the mitochondrial *COI* gene) has been applied to chigger mites to determine whether low-throughput morphological identification can be supplanted, or at least complemented, by less laborious procedures. The first study to analyse *COI* barcodes from multiple chigger species, which was conducted in South-East Asia, demonstrated that the technique reliably binned individual specimens by morphotyped species and clustered subgenera in cognate groups [34]. However, certain species exhibited multiple haplotypes, sometimes even if recovered from the same individual host. Importantly, barcoding studies of European chiggers have revealed clear cases both of phenotypic plasticity within trombiculid species (which is linked to the host species used for larval development) [65] and cryptic diversity, where single chigger morphotypes show genetic distances similar to that between recognised species [66]. In the current study, while most species clustered by subgenus when compared with published barcodes, *Ascoschoengastia* spp. and *Schoutedenichia* spp. were striking exceptions. Moreover, the system of subfamilies and tribes within the family Trombiculidae that has existed for over half-a-century was not reflected in the *COI*-based phylogeny. A clear example of this was the apparent affinity of *Pentidionis* with *Schoutedenichia*, despite their classification in different tribes (Trombiculini and Schoengastiini, respectively). While these findings suggest that the classification of trombiculid mites based on larval morphology has significant limitations, phylogenetic relationships cannot be resolved using a single mitochondrial gene, and there is an urgent need to develop multi-locus-based approaches to trombiculid taxonomy.

Here, we were able to successfully generate complete mitogenomic assemblies from three pools of *P. agamae*, which is the first time multiple mitogenomes from a single chigger species have been obtained for intraspecific comparisons. Notably, we found tRNA gene annotation to be dependent on manual comparisons with available trombiculid mitogenomes due to previously recognised non-canonical features of these genes in multiple acariform taxa [67–69]. Several SNPs were identified between pools of *P. agamae*, including non-synonymous substitutions, despite the mites being collected from the same province. Unfortunately, the paucity of whole mitogenome data from other trombiculid species severely limited interspecific comparisons. This is particularly problematic, as of the five other complete mitogenomes available from trombiculid mites, three are from a single genus (*Leptotrombidium* spp.) [33], and one of the non-*Leptotrombidium* assemblies is from a mite identified to genus level only (*Ascoschoengastia* sp. TATW-1). While mitochondrial gene order in *P. agamae* was most closely related that of *W. hayashii*, the phylogenetic position of the latter in the COI tree was unexpected, as it did not cluster with published sequences available for five *Walchia* spp. from South-East Asia. No information on how *W. hayashii* specimens were identified prior to sequencing is available, as the mitogenome record on NCBI is not linked to a publication and the depositors are no longer active in research. Thus, it is unclear if this is a case of misidentification or if the subgenus *Walchia* is paraphyletic.

Since we have demonstrated that assembly of chigger mitogenomes is feasible using ethanol-preserved pools and Illumina technology, which is declining rapidly in cost per sample, we hope these results with spur routine sequencing of trombiculid mitogenomes. Indeed, this has happened already for ticks, revolutionising phylogenetics for the Ixodidae and Argasidae [70]. However, it is important that phylogenetically-informative nuclear markers such as ITS2 are also utilised due to differing evolutionary rates between nuclear and mitochondrial genomes [71], the potential for vertically-transmitted symbionts such as *Wolbachia* to cause cytonuclear discordance [72], and the possibility that trombiculid species may hybridise [73].

## Conclusions

Our PCR-based screening and sequencing of chigger mites from Saudi Arabia has revealed *P. agamae* as a potential vector of *Ca*. O. chuto, but further research is required to determine if this species may be anthropophilic and thus important in scrub typhus epidemiology in the Middle East. Moreover, this first metagenomic analysis of a trombiculid mite outside the genus *Leptotrombidium* has enabled deeper insights into chigger-associated *Wolbachia* and *Borrelia* bacteria that were only known previously from 16S rRNA gene data, as well as providing a reference mitogenome for the genus *Pentidionis* and initial evidence for intraspecific variation. Overall, the metagenomic approach we applied here has demonstrated its potential to generate complete mitogenomes for phylogenetic and population genetic studies of trombiculids with relative ease; furthermore, it can greatly improve our understanding of chigger microbiomes that so far have been studied predominantly by 16S rRNA amplicon-based methods.

## Methods

### Chigger collection and identification

**Fig. 8:**
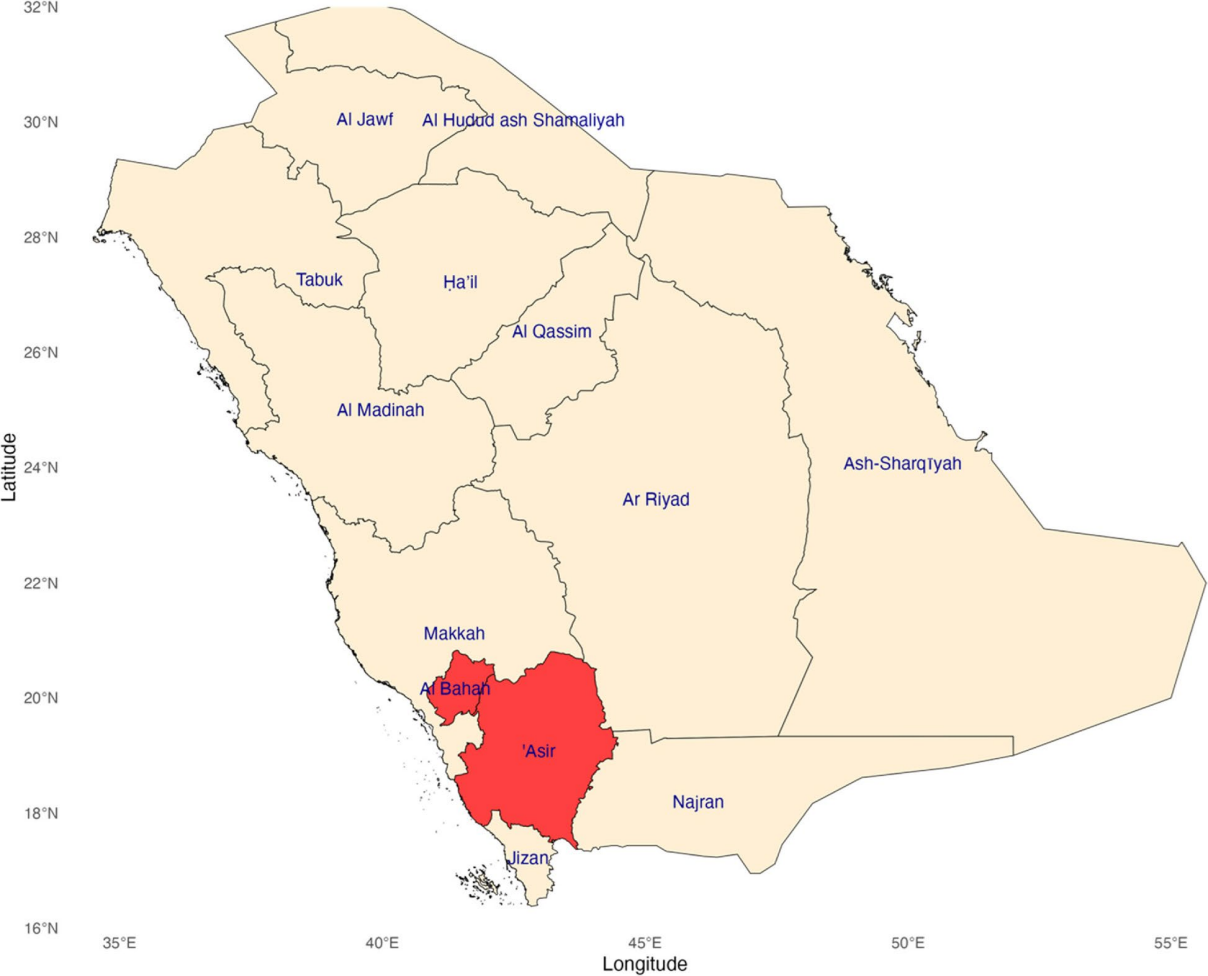
A map of Saudi Arabia highlighting ‘Asir and Al-Bahah provinces (outlined in red) where rodent sampling was conducted

Wild rodents were trapped overnight in southwestern Saudi Arabia on mountainsides and scrublands in ‘Asir (October 2020) and Al-Bahah (August 2021) provinces (Fig. 8) as described previously [7]. Rodents were euthanized by inhalational anaesthetic isoflurane overdose or dislocated in the cervical region. The identification of rodents was based on morphological features and confirmed molecularly through the amplification of *cytB* gene fragment [7]. Each rodent was carefully inspected for chiggers including inside ears and removed chiggers were preserved in 70% ethanol. The fieldwork was approved by the Saudi Wildlife Authority (approval no. 288/33/A) and Animal Welfare and Ethics Review Board of the University of Liverpool. As representative specimens, 10% of chiggers were selected by purposive sampling and fixed permanently using Berlese fluid (TCS Bioscience Ltd, Buckingham, UK). The measurements and identification of chiggers were performed on a fluorescence microscope (ZEISS Axio Imager M2 microscope through GT Vision GXCapture-T software). The remaining chiggers were identified without the usage of mountant and pooled on the basis of species from each rodent (Additional file 1: Table S2). For each chigger species, 8-31 individuals were pooled from each rodent. Chigger species with less than eight individuals were excluded from the study.

### Molecular detection of *Orientia* sp.

Genomic DNA from chigger pools were extracted using the Qiagen DNeasy Blood & Tissue Kit (Qiagen) according to the manufacturer’s protocol. DNA concentration and quality were assessed by a Qubit High Sensitivity dsDNA Quantification Assay kit (Invitrogen) and NanoDrop One/One^C^ Microvolume UV-Vis Spectrophotometer (Thermo Scientific). A quantitative PCR assay (qPCR) targeting the multicopy *traD* gene was used in the initial screening of chigger pools for detection of *Orientia* sp. [74]. Positive samples were subjected to a nested PCR assay for amplification of the *htrA* gene [6]. The PCR amplicons were purified and submitted to Eurofins Genomics (https://www.eurofins.com) for Sanger sequencing in both directions. Paired sequences were aligned to generate a corrected consensus and manually quality-trimmed using Bioedit 7.2.5 [75] to produce the final sequence for phylogenetic analyses.

### Illumina sequencing

Illumina library preparation and sequencing from chigger pools were performed at the Centre for Genomic Research (CGR) at the University of Liverpool. NEBNext Ultra II FS kit paired- end libraries (2×150 bp) with a 350 bp insert were generated and sequenced on an Illumina NovaSeq 6000 using SP or S4 chemistry. The CGR performed the following read curation: the raw fastq files were trimmed for the presence of Illumina adapter sequences using Cutadapt v1.2.1 [76] with option -O 3; the reads were further trimmed using Sickle v1.200 with a minimum window quality score of 20 (https://github.com/najoshi/sickle); and reads shorter than 15 bp after trimming were removed.

### Metagenomic assembly and taxonomic classification

Trimmed paired-end Illumina reads were assembled using metaSPAdes v3.15.5 [77] or Megahit v1.2.9 [78] genome assemblers. When the memory requirements for metaSPAdes exceeded the memory available on our servers, we removed the reads mapped to *A. dimidiatus* (GCA_907164435.1) to reduce the proportion of animal host sequences and used the unmapped reads for assembly with metaSPAdes. Short-read mapping was performed using bowtie2 v2.5.1 [79]. We applied Kraken2 (v2.1.3) classification using the NCBI non-redundant nucleotide database (02/05/2023) for taxonomic classification. Contigs classified to taxon of interest were extracted from the Kraken2 output using the “extract_kraken_reads.py” script from KrakenTools [80]. Kronagrams were generated from the Kraken2 reports using KronaTools v2.8.1 [81]. Sequence annotation for further verification of the assigned contigs was performed by a DIAMOND BLASTX v2.0.14.152 [82] search against the NCBI non-redundant protein database and BLASTn search against the NCBI non-redundant nucleotide database.

For *Wolbachia* assembly, paired-end read sequences were separately mapped to the contigs produced from metaSPAdes and Megahit using bowtie2 and re-paired and merged using FLASH v1.2.11 [83]. Mapped reads were reassembled with metaSPAdes, and Blobtoolkit v4.2.1 was used to remove eukaryotic sequences in the assembly and to generate the blobplot [84]. Prokka v1.14.6 was used for gene prediction and annotation [85]. Genome completeness was assessed by the Benchmarking Universal Single-Copy Orthologs (BUSCO) pipeline 5.0 and the rickettsiales_odb10 database [86].

### Mitochondrial genome assembly

The *P. agamae* mitochondrial genome was assembled using the MitoZ toolkit v3.3 [87] with additional annotations of protein coding sequences (CDS), ribosomal RNA (rRNA) and transfer RNA (tRNA) sequences using the MITOS2 web service [88], which produces circular and non-circular assemblies. The non-circular assembly was artificially circularised using the Simple-Circularise python script (https://github.com/Kzra/Simple-Circularise). Missing tRNAs were identified by aligning the assembly with existing mitochondrial genomes from trombiculid mites available in NCBI GenBank (*Leptotrombidium pallidum*, AB180098.1; *Leptotrombidium deliense*, AB194044.1; *Leptotrombidium akamushi*, NC_007601.1; *Walchia hayashii*, NC_010595.1; and *Ascoschoengastia* sp. TATW-1, AB300501.1) and manually inspecting the presence of conserved regions for tRNAs. Alignments of putative tRNA sequences were performed with MAFFT v6.864b [89] as described above and visualised using Jalview v2 [90]. RNA secondary structures were predicted using mfold [91] in the UNAFold web service (http://www.unafold.org) and the predicted structures for the tRNAs from *L. pallidum* [92] as a reference. The mitochondrial assembly was visualised using the web version of OGDRAW [93] to produce the circular genome plot in Fig. 6. Alignments of the mitochondrial assembly and the detection of single nucleotide polymorphisms (SNPs) were performed using nucmer and dnadiff from the MUMmer4 package [94]. Genome synteny was visualised using genoPlotR [95]. Existing chigger mitogenomes analysed for gene arrangements comprised *Ascoschoengastia* sp. TATW-1, *W. hayashii*, *L. deliense* and *L. pallidum* (accession nos. above). Sequences for the *COI* gene were generated from pooled archived chigger specimens described in our earlier studies [37, 45] by PCR amplification using the HCO2198 and LCO1490 primers [96] followed by Sanger sequencing.

### Phylogenetic tree construction and genetic pairwise distance calculation

Sequences for genes of interest were aligned using MAFFT v6.864b [89] along with existing sequences from NCBI GenBank. Concatenated alignments and partition files were generated using FASconCAT-G (https://github.com/PatrickKueck/FASconCAT-G). Maximum-likelihood phylogenies were produced from the single or concatenated nucleotide alignments using IQTREE v2.2.2.9[97] with 1,000 ultra-fast bootstraps [98] and best model selection from ModelFinder [99]. The Interactive Tree of Life online tool (https://itol.embl.de) was used to visualize the consensus trees produced and to generate the tree figures. Numbers at nodes represent ultra-fast bootstrap values and tree scales represent number of nucleotide substitutions. Genetic pairwise distances were calculated from alignments using the DistanceCalculator class from the Bio.Phylo.TreeConstruction module in Biopython v1.79 [100].

For the *Wolbachia* phylogenomic tree, Orthofinder v2.5.4 was used to produce a set of orthologous sequences [101]. Protein sequences for each single-copy orthogroup (OG) were aligned using MAFFT v7.149b [89]. Gblocks v0.91b was used to trim noisy or poorly aligned protein positions [102]. The trimmed alignments were concatenated into a supermatrix used to construct maximum likelihood trees in IQTREE v2.1.2. We used ModelFinder within IQTREE to determine the appropriate model for each protein. Branch support was calculated using the following options in IQTREE: (i) ultra- fast bootstrap, (ii) SH- aLRT support, (iii) local bootstrap support and (iv) aBayes Bayesian support, with all options set to 1,000, and all options produced highly similar values. Values from the ultra-fast bootstrap option [103] were displayed along with the consensus trees in the final figures. Sequence comparisons of *w*Paga, *w*DacA, *w*DacB and *w*Tex genomes to all the genomes represented in the phylogenomic tree was performed using megablast. The output results were filtered to remove self-hits and the best hit for each contig was retained. The percentage identity and query coverage in three Blast hits were analysed and plotted, with each contig hit coloured by supergroup affiliation of the best matching subject sequence. In the analysis of *w*Tex, hits to the only other *Wolbachia* (*w*Ppe) from the same supergroup L were also excluded to simulate a novel, highly divergent *Wolbachia* supergroup.

### Supplementary Information

**Additional file 1:** Supplementary tables and figures

**Additional file 2:** Kronagram for Kraken2 output at confidence threshold 0.1 for *Pentidionis agamae *pool R9P

**Additional file 3:** Kronagram for Kraken2 output at confidence threshold 0.1 for *Pentidionis agamae *pool Pa1

**Additional file 4:** Kronagram for Kraken2 output at confidence threshold 0.1 for *Pentidionis agamae *pool Pa2

**Additional file 5:** Individual gene trees for single copy orthologs from *Wolbachia*

## Data Availability

Sequencing reads and assembled sequences produced in this study have been deposited in NCBI GenBank with the BioProject accession number PRJNA1031942. The following sequences were also deposited in NCBI Genbank: The *Orientia* sp. *htrA* sequence was deposited with the accession number OR966881. *Borrelia* sp. sequences were deposited with accession numbers OR817655 and OR817732-OR817734. The *Wolbachia* *w*Paga assembly was deposited with accession number JAZHPY000000000. Chigger mitochondrial *COI* sequences were deposited with accession numbers OR820617-OR820651. The mitochondrial genome assembly for *P. agamae* Pa1 was deposited with the accession number OR817658.

## References

[1] Womersley H (1952). The scrub-typhus and scrub-itch mites (Trombiculidae, Acarina) of the Asiatic-Pacific region. Rec S Aust Mus..

[2] Elliott I, Pearson I, Dahal P, Thomas NV, Roberts T, Newton PN (2019). Scrub typhus ecology: a systematic review of *Orientia* in vectors and hosts. Parasit Vectors..

[3] Bonell A, Lubell Y, Newton PN, Crump JA, Paris DH (2017). Estimating the burden of scrub typhus: a systematic review. PLOS Negl Trop Dis..

[4] Abarca K, Martínez-Valdebenito C, Angulo J, Jiang J, Farris C, Richards A (2020). Molecular description of a novel *Orientia* species causing scrub typhus in Chile. Emerging Infect Dis..

[5] Izzard L, Fuller A, Blacksell Stuart D, Paris Daniel H, Richards Allen L, Aukkanit N (2010). Isolation of a novel *Orientia* species (*O. chuto* sp. nov.) from a patient Infected in Dubai. J Clin Microbiol..

[6] Masakhwe C, Linsuwanon P, Kimita G, Mutai B, Leepitakrat S, Yalwala S (2018). Identification and characterization of *Orientia chuto* in trombiculid chigger mites collected from wild rodents in Kenya. J Clin Microbiol..

[7] Alkathiry HA, Alghamdi SQ, Morgan HEJ, Noll ME, Khoo JJ, Alagaili AN (2023). Molecular detection of *Candidatus* Orientia chuto in wildlife. Saudi Arabia. Emerging Infect Dis..

[8] Weitzel T, Silva-de la Fuente MC, Martínez-Valdebenito C, Stekolnikov AA, Pérez C, Pérez R (2022). Novel vector of scrub typhus in sub-antarctic Chile: evidence from human exposure. Clin Infect Dis..

[9] Chaisiri K, Linsuwanon P, Makepeace BL (2023). The chigger microbiome: big questions in a tiny world. Trends Parasitol..

[10] Lessner K, Krawiec C (2020). Tick-borne-associated illnesses in the pediatric intensive care unit. J Pediatr Infect Dis..

[11] Eremeeva ME, Muniz-Rodriguez K (2020). Rickettsialpox — a rare but not extinct disease: review of the literature and new directions. Russ J Infect Immun..

[12] Ponnusamy L, Garshong R, McLean BS, Wasserberg G, Durden LA, Crossley D (2022). *Rickettsia felis* and other *Rickettsia* species in chigger mites collected from wild rodents in North Carolina. USA. Microorganisms..

[13] Kuo C-C, Lee P-L, Wang H-C (2022). Molecular identification of *Rickettsia* spp. in chigger mites in Taiwan. Med Vet Entomol..

[14] Bassini-Silva R, Jacinavicius FdC, Maturano R, Muñoz-Leal S, Ochoa R, Bauchan G (2018). *Blankaartia sinnamaryi* (Trombidiformes: Trombiculidae) parasitizing birds in southeastern Brazil, with notes on *Rickettsia* detection. Revista Brasileira de Parasitologia Veterinária..

[15] Huang Y, Zhao L, Zhang Z, Liu M, Xue Z, Ma D (2017). Detection of a novel *Rickettsia* from *Leptotrombidium scutellare* mites (Acari: Trombiculidae) from Shandong of China. J Med Entomol..

[16] Ogawa M, Takahashi M, Matsutani M, Takada N, Noda S, Saijo M (2020). Obligate intracellular bacteria diversity in unfed *Leptotrombidium scutellare* larvae highlights novel bacterial endosymbionts of mites. Microbiol Immunol..

[17] Fernández-Soto P, Pérez-Sánchez R, Encinas-Grandes A (2001). Molecular detection of *Ehrlichia phagocytophila* genogroup organisms in larvae of *Neotrombicula autumnalis* (Acari: Trombiculidae) captured in Spain. J Parasitol..

[18] Chomel BB, Kasten RW (2010). Bartonellosis, an increasingly recognized zoonosis. J Appl Microbiol..

[19] Centers for Disease Control and Prevention. *Bartonella* Infection. https://www.cdc.gov/bartonella/index.html Accessed 28 Dec 2023.

[20] Loan HK, Cuong NV, Takhampunya R, Klangthong K, Osikowicz L, Kiet BT (2015). *Bartonella* species and trombiculid mites of rats from the Mekong Delta of Vietnam. Vector-Borne and Zoonotic Diseases..

[21] Kabeya H, Colborn JM, Bai Y, Lerdthusnee K, Richardson JH, Maruyama S (2009). Detection of *Bartonella tamiae* DNA in ectoparasites from rodents in Thailand and their sequence similarity with bacterial cultures from Thai patients. Vector Borne Zoonotic Dis..

[22] Margos G, Gofton A, Wibberg D, Dangel A, Marosevic D, Loh S-M (2018). The genus *Borrelia* reloaded. PLoS One.

[23] Wolcott KA, Margos G, Fingerle V, Becker NS (2021). Host association of *Borrelia burgdorferi* sensu lato: A review. Ticks Tick Borne Dis..

[24] Literak I, Stekolnikov AA, Sychra O, Dubska L, Taragelova V (2008). Larvae of chigger mites *Neotrombicula* spp. (Acari: Trombiculidae) exhibited *Borrelia* but no *Anaplasma* infections: a field study including birds from the Czech Carpathians as hosts of chiggers. Exp Appl Acarol.

[25] Netušil J, Zákovská A, Horváth R, Dendis M, Janouškovcová E (2005). Presence of *Borrelia burgdorferi* Sensu Lato in mites parasitizing small rodents. Vector-Borne and Zoonotic Diseases..

[26] Kampen H, Schöler A, Metzen M, Oehme R, Hartelt K, Kimmig P (2004). *Neotrombicula autumnalis* (Acari, Trombiculidae) as a vector for *Borrelia burgdorferi* sensu lato?. Exp Appl Acarol..

[27] Egan SL, Taylor CL, Banks PB, Northover AS, Ahlstrom LA, Ryan UM (2021). The bacterial biome of ticks and their wildlife hosts at the urban–wildland interface. Microb Genom..

[28] Fedorova N, Kleinjan JE, James D, Hui LT, Peeters H, Lane RS (2014). Remarkable diversity of tick or mammalian-associated Borreliae in the metropolitan San Francisco Bay Area. California. Ticks Tick Borne Dis..

[29] Shropshire JD, Leigh B, Bordenstein SR (2020). Symbiont-mediated cytoplasmic incompatibility: What have we learned in 50 years?. eLife..

[30] Wang G-H, Gamez S, Raban RR, Marshall JM, Alphey L, Li M (2021). Combating mosquito-borne diseases using genetic control technologies. Nat Commun..

[31] Dong X, Chaisiri K, Xia D, Armstrong SD, Fang Y, Donnelly MJ (2018). Genomes of trombidid mites reveal novel predicted allergens and laterally transferred genes associated with secondary metabolism. GigaScience..

[32] Kim JH, Roh J, Yoon KA, Kim K, Shin Eh, Park M-Y (2020). Genome/transcriptome analysis of the chigger mite *Leptotrombidium pallidum*, a major vector for scrub typhus, with a special focus on genes more abundantly expressed in larval stage. J Asia-Pacif Entomol..

[33] Shao R, Barker SC, Mitani H, Takahashi M, Fukunaga M (2006). Molecular mechanisms for the variation of mitochondrial gene content and gene arrangement among chigger mites of the genus *Leptotrombidium* (Acari: Acariformes). J Mol Evol..

[34] Kumlert R, Chaisiri K, Anantatat T, Stekolnikov AA, Morand S, Prasartvit A (2018). Autofluorescence microscopy for paired-matched morphological and molecular identification of individual chigger mites (Acari: Trombiculidae), the vectors of scrub typhus. PLOS ONE..

[35] Motohiko O, Nobuhiro T, Shinichi N, Mamoru T, Minenosuke M, Daisuke K (2023). Genetic variation of *Leptotrombidium* (Acari: Trombiculidae) mites carrying *Orientia tsutsugamushi*, the bacterial pathogen causing scrub typhus. J Parasitol..

[36] Zajkowska P, Postawa T, Mąkol J (2023). Let me know your name: a study of chigger mites (Acariformes: Trombiculidae) associated with the edible dormouse (*Glis glis*) in the Carpathian-Balkan distribution gradient. Exp Appl Acarol..

[37] Stekolnikov AA, Al-Ghamdi SQ, Alagaili AN, Makepeace BL (2019). First data on chigger mites (Acariformes: Trombiculidae) of Saudi Arabia, with a description of four new species. Syst Appl Acarol..

[38] Erban T, Klimov PB, Harant K, Talacko P, Nesvorna M, Hubert J (2021). Label-free proteomic analysis reveals differentially expressed *Wolbachia* proteins in *Tyrophagus putrescentiae*: Mite allergens and markers reflecting population-related proteome differences. J Proteomics..

[39] Głowska E, Gerth M (2023). Draft genome sequence of a *Wolbachia* endosymbiont from *Syringophilopsis turdi* (Fritsch, 1958) (Acari, Syringophilidae). Microbiol Resour Announc..

[40] Sinha A, Li Z, Poole CB, Ettwiller L, Lima NF, Ferreira MU (2023). Multiple lineages of nematode-*Wolbachia* symbiosis in Supergroup F and convergent loss of bacterioferritin in filarial *Wolbachia*. Genome Biol Evol..

[41] Margos G, Binder K, Dzaferovic E, Hizo-Teufel C, Sing A, Wildner M (2015). PubMLST.org – The new home for the *Borrelia* MLSA database. Ticks Tick Borne Dis..

[42] Gil H, Barral M, Escudero R, García-Pérez Ana L, Anda P (2005). Identification of a new *Borrelia* species among small mammals in areas of northern Spain where Lyme disease is endemic. Appl Environ Microbiol..

[43] Kudryashova NI. Chigger mites (Acariformes, Trombiculidae) of East Palaearctics. Moscow: KMK Scientific Press; 1998. p. 342. (In Russian).

[44] Stekolnikov AA (2018). Taxonomy and distribution of African chiggers (Acariformes, Trombiculidae). Eur J Taxon..

[45] Alghamdi SQ, Alkathiry HA, Stekolnikov AA, Alagaili AN, Makepeace BL (2023). Additions to the chigger mite fauna (Acariformes: Trombiculidae) of Saudi Arabia, with the description of a new species. Acarologia..

[46] Vercammen-Grandjean PH, Kolebinova MG (1985). Revision of *Neotrombicula* complex (Acarina, Trombiculidae). Acta Zoologica Bulgarica..

[47] Kolebinova MG. Fauna Bulgarica, 21. Acariformes, Trombidioidea, Trombiculidae, and Leeuwenhoekiidae. Sofia: Academie Scientiarium Bulgaricae; 1992. p. 172 (in Bulgarian).

[48] Nadchatram M, Dohany AL (1974). A pictorial key to the subfamilies, genera, and subgenera of Southeast Asian chiggers (Acari; Prostigmata, Trombiculidae. Bulletin of the Institute for Medical Research, Kuala Lumpur, Malaysia..

[49] Cosson JF, Galan M, Bard E, Razzauti M, Bernard M, Morand S (2015). Detection of *Orientia* sp. DNA in rodents from Asia, West Africa and Europe. Parasit Vectors.

[50] Elliott I, Thangnimitchok N, de Cesare M, Linsuwanon P, Paris DH, Day NPJ (2021). Targeted capture and sequencing of *Orientia tsutsugamushi* genomes from chiggers and humans. Infect, Genet Evol..

[51] Batty EM, Chaemchuen S, Blacksell S, Richards AL, Paris D, Bowden R (2018). Long-read whole genome sequencing and comparative analysis of six strains of the human pathogen *Orientia tsutsugamushi*. PLOS Negl Trop Dis..

[52] Vercammen-Grandjean PH, Rohde CJ, Mesghali H (1970). Twenty larval Trombiculidae (Acarina) from Iran. J Parasitol..

[53] André M. Nouvelle forme larvaire de *Thrombicula* parasite sur un Saurien de Palestine. Bulletin du Muséum national d’Histoire naturelle, 2ème série. 1929;1:401–405 (in French).

[54] Stekolnikov AA (2023). New records of chigger mites (Acariformes, Trombiculidae) from the Arabian Peninsula. Acarina..

[55] Baldo L, Dunning Hotopp JC, Jolley Keith A, Bordenstein SR, Biber SA, Choudhury RR (2006). Multilocus sequence typing system for the endosymbiont *Wolbachia pipientis*. Appl Environ Microbiol..

[56] Bleidorn C, Gerth M (2018). A critical re-evaluation of multilocus sequence typing (MLST) efforts in *Wolbachia*. FEMS Microbiol Ecol..

[57] Chaisiri K, Gill AC, Stekolnikov AA, Hinjoy S, McGarry JW, Darby AC (2019). Ecological and microbiological diversity of chigger mites, including vectors of scrub typhus, on small mammals across stratified habitats in Thailand. Animal Microbiome..

[58] Gerth M, Gansauge M-T, Weigert A, Bleidorn C (2014). Phylogenomic analyses uncover origin and spread of the *Wolbachia* pandemic. Nat Commun..

[59] Rodrigues J, Lefoulon E, Gavotte L, Perillat-Sanguinet M, Makepeace B, Martin C (2023). *Wolbachia* springs eternal: symbiosis in Collembola is associated with host ecology. R Soc Open Sci..

[60] Weyandt N, Aghdam SA, Brown AMV (2022). Discovery of early-branching *Wolbachia* reveals functional enrichment on horizontally transferred genes. Front Microbiol..

[61] Dudzic JP, Curtis CI, Gowen BE, Perlman SJ (2022). A highly divergent *Wolbachia* with a tiny genome in an insect-parasitic tylenchid nematode. Proc Biol Sci..

[62] Oliveira CMd, Návia D, Frizzas MR (2007). First record of *Tyrophagus putrescentiae* (Schrank)(Acari: Acaridae) in soybean plants under no tillage in Minas Gerais, Brazil. Ciência Rural.

[63] Takhampunya R, Korkusol A, Pongpichit C, Yodin K, Rungrojn A, Chanarat N, et al. Metagenomic approach to characterizing disease epidemiology in a disease-endemic environment in northern Thailand. Front Microbiol. 2019;10:319. 10.3389/fmicb.2019.00319 PMC6399164 30863381

[64] Stekolnikov AA, Shamsi M, Saboori A, Zahedi Golpayegani A, Hakimitabar M (2022). Distribution of chigger mites (Acari: Trombiculidae) over hosts, parasitopes, collection localities, and seasons in northern Iran. Exp Appl Acarol..

[65] Moniuszko H, Zaleśny G, Mąkol J (2015). Host-associated differences in morphometric traits of parasitic larvae *Hirsutiella zachvatkini* (Actinotrichida: Trombiculidae). Exp Appl Acarol..

[66] Zajkowska P, Mąkol J (2022). Parasitism, seasonality, and diversity of trombiculid mites (Trombidiformes: Parasitengona, Trombiculidae) infesting bats (Chiroptera) in Poland. Exp Appl Acarol..

[67] Xue X-F, Deng W, Qu S-X, Hong X-Y, Shao R (2018). The mitochondrial genomes of sarcoptiform mites: are any transfer RNA genes really lost?. BMC Genomics..

[68] Xue X-F, Guo J-F, Dong Y, Hong X-Y, Shao R (2016). Mitochondrial genome evolution and tRNA truncation in Acariformes mites: new evidence from eriophyoid mites. Sci Rep..

[69] Yuan M-L, Wei D-D, Wang B-J, Dou W, Wang J-J (2010). The complete mitochondrial genome of the citrus red mite *Panonychus citri* (Acari: Tetranychidae): high genome rearrangement and extremely truncated tRNAs. BMC Genomics..

[70] Kelava S, Mans BJ, Shao R, Moustafa MAM, Matsuno K, Takano A (2021). Phylogenies from mitochondrial genomes of 120 species of ticks: Insights into the evolution of the families of ticks and of the genus *Amblyomma*. Ticks Tick Borne Dis..

[71] Allio R, Donega S, Galtier N, Nabholz B (2017). Large variation in the ratio of mitochondrial to nuclear mutation rate across animals: implications for genetic diversity and the use of mitochondrial DNA as a molecular marker. Mol Biol Evol..

[72] Cariou M, Duret L, Charlat S (2017). The global impact of *Wolbachia* on mitochondrial diversity and evolution. J Evol Biol..

[73] Kadosaka T, Fujiwara M, Kimura E, Kaneko K (1994). Hybridization experiments using 3 species of the scrub typhus vectors, *Leptotrombidium akamushi*, *L. deliense* and *L. fletcheri*. Med Entomol Zool..

[74] Chao C-C, Belinskaya T, Zhang Z, Jiang L, Ching W-M (2019). Assessment of a sensitive qPCR assay targeting a multiple-copy gene to detect *Orientia tsutsugamushi* DNA. Trop Med Infect Dis..

[75] Hall T, Biosciences I, Carlsbad C (2011). BioEdit: an important software for molecular biology. GERF Bull Biosci..

[76] Martin M (2011). Cutadapt removes adapter sequences from high-throughput sequencing reads. EMBnetjournal..

[77] Nurk S, Meleshko D, Korobeynikov A, Pevzner PA (2017). metaSPAdes: a new versatile metagenomic assembler. Genome Res..

[78] Li D, Liu C-M, Luo R, Sadakane K, Lam T-W (2015). MEGAHIT: an ultra-fast single-node solution for large and complex metagenomics assembly via succinct de Bruijn graph. Bioinformatics..

[79] Langmead B, Salzberg SL (2012). Fast gapped-read alignment with Bowtie 2. Nat Methods..

[80] Lu J, Rincon N, Wood DE, Breitwieser FP, Pockrandt C, Langmead B (2022). Metagenome analysis using the Kraken software suite. Nat Protoc..

[81] Ondov BD, Bergman NH, Phillippy AM (2011). Interactive metagenomic visualization in a Web browser. BMC Bioinformatics..

[82] Buchfink B, Xie C, Huson DH (2015). Fast and sensitive protein alignment using DIAMOND. Nat Methods..

[83] Magoč T, Salzberg SL (2011). FLASH: fast length adjustment of short reads to improve genome assemblies. Bioinformatics..

[84] Challis R, Richards E, Rajan J, Cochrane G, Blaxter M (2020). BlobToolKit – Interactive quality assessment of genome assemblies. G3.

[85] Seemann T (2014). Prokka: rapid prokaryotic genome annotation. Bioinformatics..

[86] Manni M, Berkeley MR, Seppey M, Simão FA, Zdobnov EM (2021). BUSCO Update: Novel and streamlined workflows along with broader and deeper phylogenetic coverage for scoring of eukaryotic, prokaryotic, and viral genomes. Mol Biol Evol..

[87] Meng G, Li Y, Yang C, Liu S (2019). MitoZ: a toolkit for animal mitochondrial genome assembly, annotation and visualization. Nucleic Acids Res..

[88] Donath A, Jühling F, Al-Arab M, Bernhart SH, Reinhardt F, Stadler PF (2019). Improved annotation of protein-coding genes boundaries in metazoan mitochondrial genomes. Nucleic Acids Res..

[89] Katoh K, Misawa K, Kuma Ki, Miyata T (2002). MAFFT: a novel method for rapid multiple sequence alignment based on fast Fourier transform. Nucleic Acids Res..

[90] Waterhouse AM, Procter JB, Martin DMA, Clamp M, Barton GJ (2009). Jalview Version 2—a multiple sequence alignment editor and analysis workbench. Bioinformatics..

[91] Zuker M (2003). Mfold web server for nucleic acid folding and hybridization prediction. Nucleic Acids Res..

[92] Shao R, Mitani H, Barker SC, Takahashi M, Fukunaga M (2005). Novel mitochondrial gene content and gene arrangement indicate illegitimate Inter-mtDNA recombination in the chigger mite. Leptotrombidium pallidum. J Mol Evol..

[93] Greiner S, Lehwark P, Bock R (2019). OrganellarGenomeDRAW (OGDRAW) version 1.3.1: expanded toolkit for the graphical visualization of organellar genomes. Nucleic Acids Res..

[94] Marçais G, Delcher AL, Phillippy AM, Coston R, Salzberg SL, Zimin A (2018). MUMmer4: A fast and versatile genome alignment system. PLoS Comp Biol..

[95] Guy L, Roat Kultima J, Andersson SGE (2010). genoPlotR: comparative gene and genome visualization in R. Bioinformatics..

[96] Folmer O, Black M, Hoeh W, Lutz R, Vrijenhoek (1994). DNA primers for amplification of mitochondrial cytochrome c oxidase subunit I from diverse metazoan invertebrates. Mol Mar Biol Biotechnol..

[97] Nguyen L-T, Schmidt HA, von Haeseler A, Minh BQ (2015). IQ-TREE: A fast and effective stochastic algorithm for estimating maximum-likelihood phylogenies. Mol Biol Evol..

[98] Hoang DT, Chernomor O, von Haeseler A, Minh BQ, Vinh LS (2018). UFBoot2: Improving the Ultrafast Bootstrap Approximation. Mol Biol Evol..

[99] Kalyaanamoorthy S, Minh BQ, Wong TKF, von Haeseler A, Jermiin LS (2017). ModelFinder: fast model selection for accurate phylogenetic estimates. Nat Methods..

[100] Cock PJA, Antao T, Chang JT, Chapman BA, Cox CJ, Dalke A (2009). Biopython: freely available Python tools for computational molecular biology and bioinformatics. Bioinformatics..

[101] Emms DM, Kelly S (2019). OrthoFinder: phylogenetic orthology inference for comparative genomics. Genome Biol..

[102] Talavera G, Castresana J (2007). Improvement of phylogenies after removing divergent and ambiguously aligned blocks from protein sequence alignments. Syst Biol..

[103] Stamatakis A, Hoover P, Rougemont J (2008). A rapid bootstrap algorithm for the RAxML web servers. Syst Biol..

