# Supplementary material for: Microbiome and mitogenomics of the chigger mite Pentidionis agamae: potential role as an Orientia vector and associations with divergent clades of Wolbachia and Borrelia
Source: BMC Genomics. 2024 Apr 17;25:380. doi: 10.1186/s12864-024-10301-6 (PMC11025265; doi:10.1186/s12864-024-10301-6)
Supplement: Supplementary file 1 — Additional file 1: Supplementary tables and figures [file 12864_2024_10301_MOESM1_ESM.pdf]

Table S1 Rodent species captured in Saudi Arabia.

Rodent species	Total No.
Acomys dimidiatus	120
Dipodillus dasyurus	4
Meriones rex	17
Mus musculus	6
Ochromyscus yemeni	8
Rattus rattus	1

Table S2 Total number of chigger pools screened for *Orientia*.

Chigger species	No. of tested pools	Range of chiggers in pools*	Total no. of chiggers
Ascoschoengastia browni	4	14 - 30	104
Ericotrombidium caucasicum	17	8 - 30	310
Ericotrombidium kazeruni	26	15 - 30	519
Helenicula lukshumiae	9	8 - 26	163
Microtrombicula felis	1	21	21
Microtrombicula muhaylensis	17	11 - 31	346
Microtrombicula traubi	1	11	11
Pentidionis agamae	24	11 - 31	484
Schoutedenichia saudi	22	8 - 28	365
Schoutedenichia zarudnyi	44	9 - 30	963
Total	165		3286

Table S3 Kraken2 assignments at different confidence thresholds

Taxon	Pool	Confidence threshold		
		0.0	0.1	0.3
Orientia	R9P	4	1	0
	Pa1	3	0	0
	Pa2	16	7	6
Wolbachia	R9P	13	0	0
	Pa1	27	4	2
	Pa2	201	8	1
Rickettsia	R9P	32	4	0
	Pa1	62	12	3
	Pa2	110	16	0
Borrelia	R9P	131	0	0
	Pa1	36	1	0
	Pa2	38	4	0

Table S4 BLAST matches for metaSPAdes contigs from Pa2 *P. agamae* pool assigned as *Orientia* by Kraken2

BLASTn

Contig ID	Length (bp)	Description	Bit (score)	E value	Percentage identity	Coverage
NODE_758076_length_270_cov_1.748837	270	LS398547.1 Orientia tsutsugamushi isolate UT176 genome assembly	496	1.00E-135	100	99
NODE_799222_length_264_cov_1.837321	264	LS398552.1 Orientia tsutsugamushi isolate UT76 genome assembly	488	2.00E-133	100	100
NODE_831272_length_260_cov_0.936585	260	LS398547.1 Orientia tsutsugamushi isolate UT176 genome assembly	481	4.00E-131	100	100
NODE_858984_length_256_cov_2.865672	256	LS398547.1 Orientia tsutsugamushi isolate UT176 genome assembly	468	3.00E-127	99	100
NODE_905908_length_251_cov_0.928571	251	LS398547.1 Orientia tsutsugamushi isolate UT176 genome assembly	464	3.00E-126	100	100
NODE_1139936_length_228_cov_1.109827	228	LS398547.1 Orientia tsutsugamushi isolate UT176 genome assembly	422	2.00E-113	100	100

Diamond BLASTx

Contig ID	Length (bp)	Description	Max score	E value	Percentage identity	Coverage
NODE_758076_length_270_cov_1.748837	270	WP_109489843.1 dihydrolipoyl dehydrogenase [Orientia tsutsugamushi]	254	4.27E-48	100	98.9
NODE_799222_length_264_cov_1.837321	264	KJW01686.1 toprim domain protein [Orientia tsutsugamushi str. Sido]	122	1.19E-48	96.6	98.9
NODE_831272_length_260_cov_0.936585	260	SPP26532.1 conjugal transfer protein TraN [Orientia tsutsugamushi]	78	2.17E-40	97.1	78.5
NODE_858984_length_256_cov_2.865672	256	WP_109490068.1 hypothetical protein [Orientia tsutsugamushi]	237	1.30E-36	100	75
NODE_905908_length_251_cov_0.928571	251	KJV51302.1 hypothetical protein OTSGILL_2366 [Orientia tsutsugamushi str. Gilliam]	141	7.57E-44	94	99.2
NODE_1139936_length_228_cov_1.109827	228	SPR03127.1 transposase [Orientia tsutsugamushi]	119	1.42E-43	100	93.4

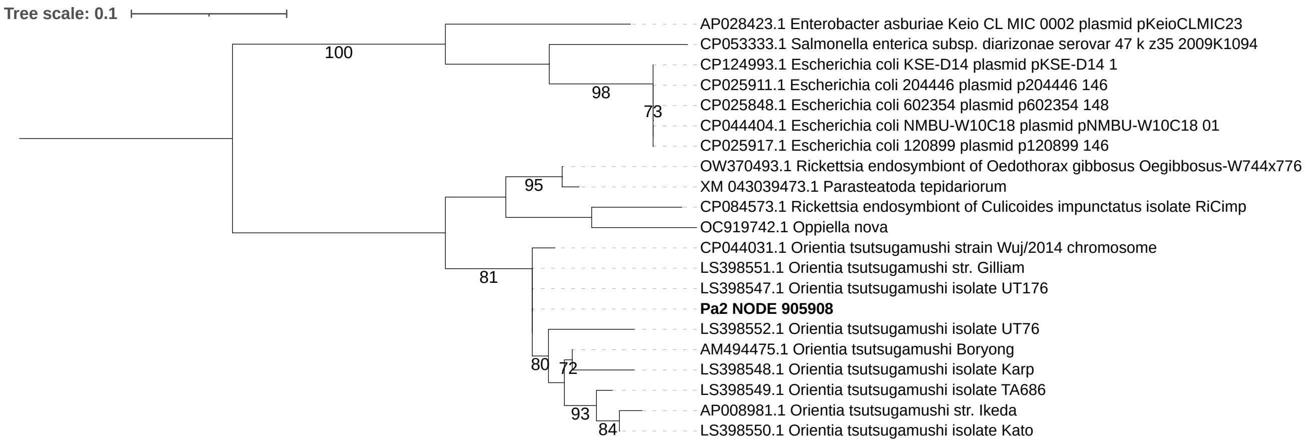

Fig. S1 Maximum-likelihood tree based on sequences related to *P. agamae* Pa2 contig 905908. Best fit model was determined as HKY+F. Number of nucleotide sites used were 253. The tree was rooted mid-point.

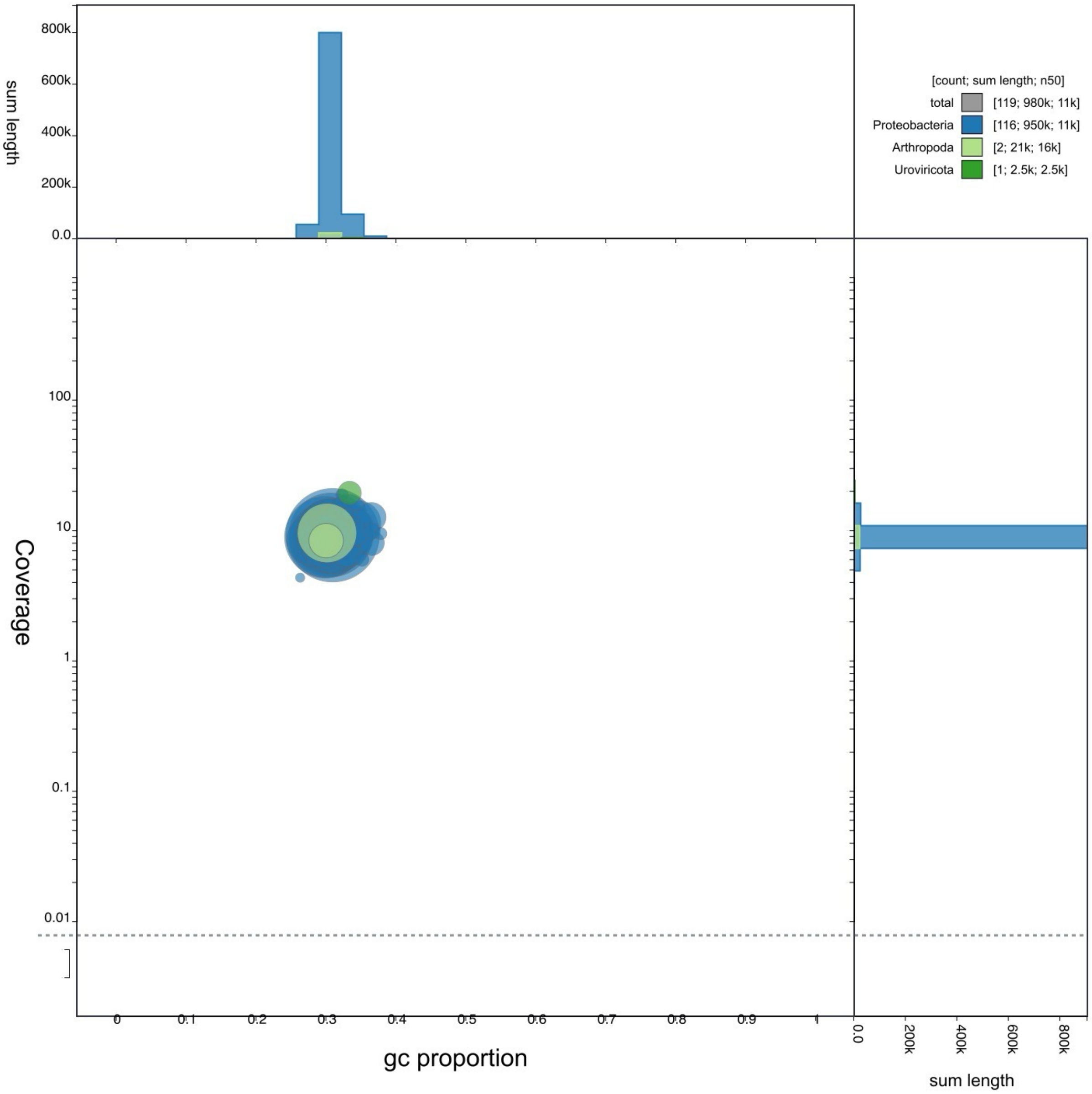

Fig. S2 Blobplot for *w*Paga MAG from *P. agamae* Pa2 pool.

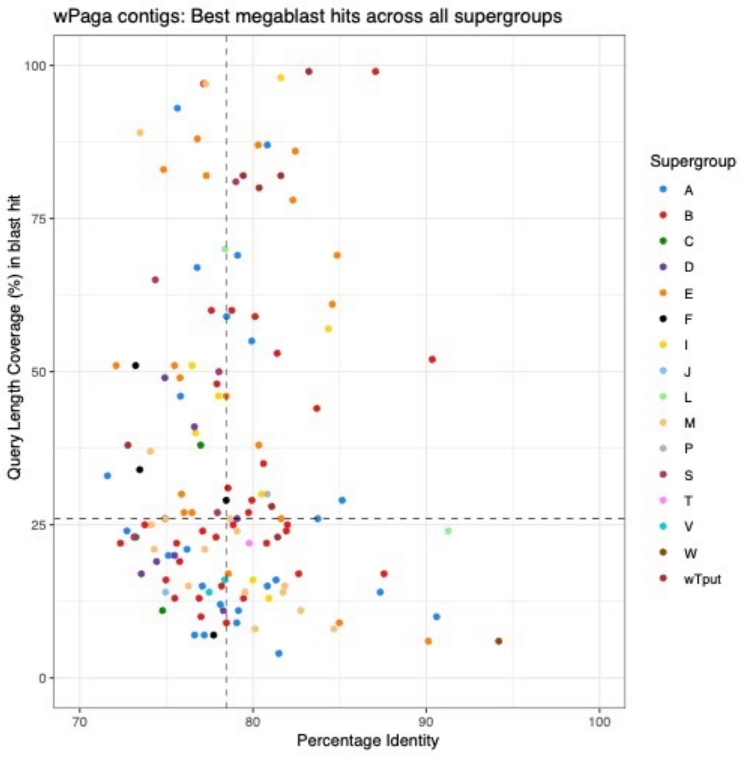

Fig. S3a Megablast hits of *w*Paga contigs across all *Wolbachia* supergroups

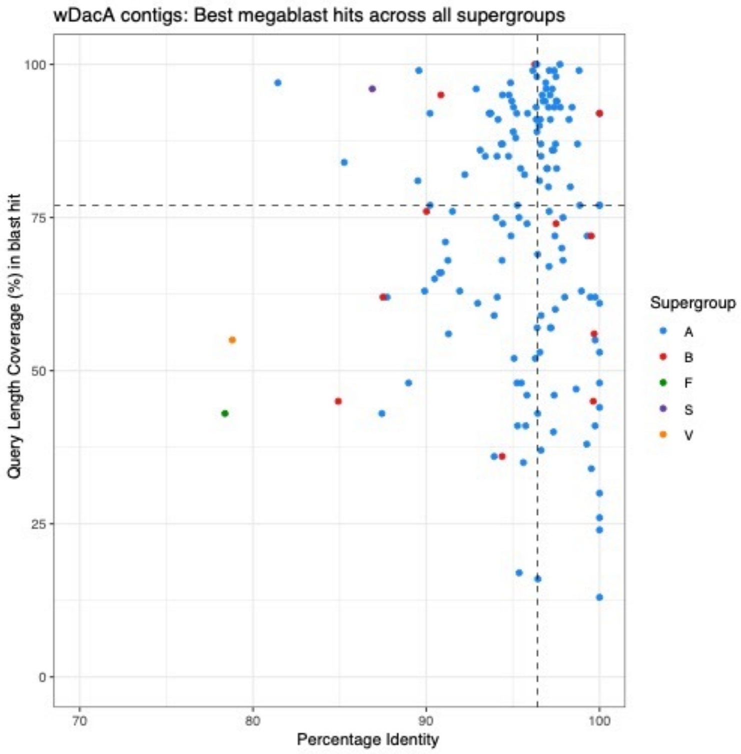

Fig. S3b Megablast hits of *w*DacA contigs across all *Wolbachia* supergroups

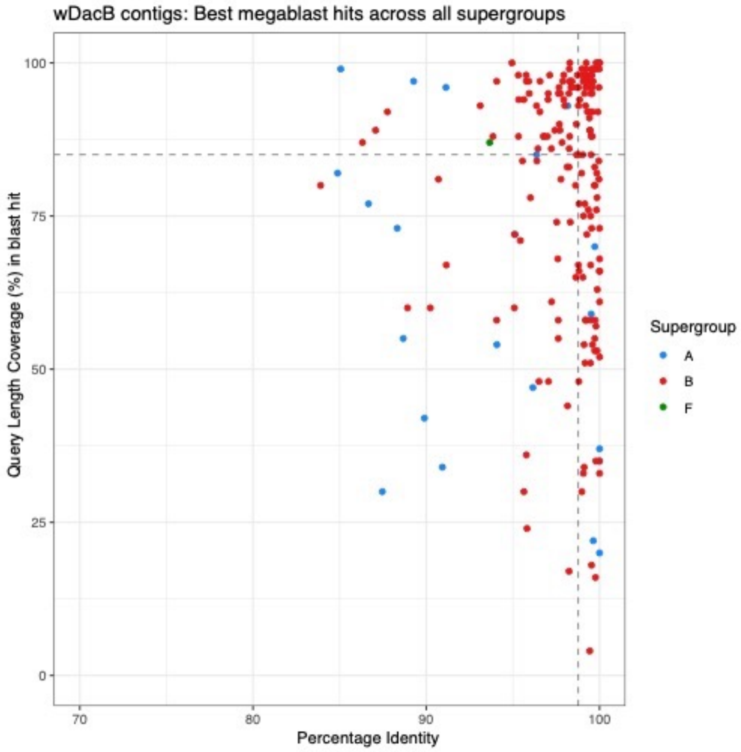

Fig. S3c Megablast hits of *w*DacB contigs across all *Wolbachia* supergroups

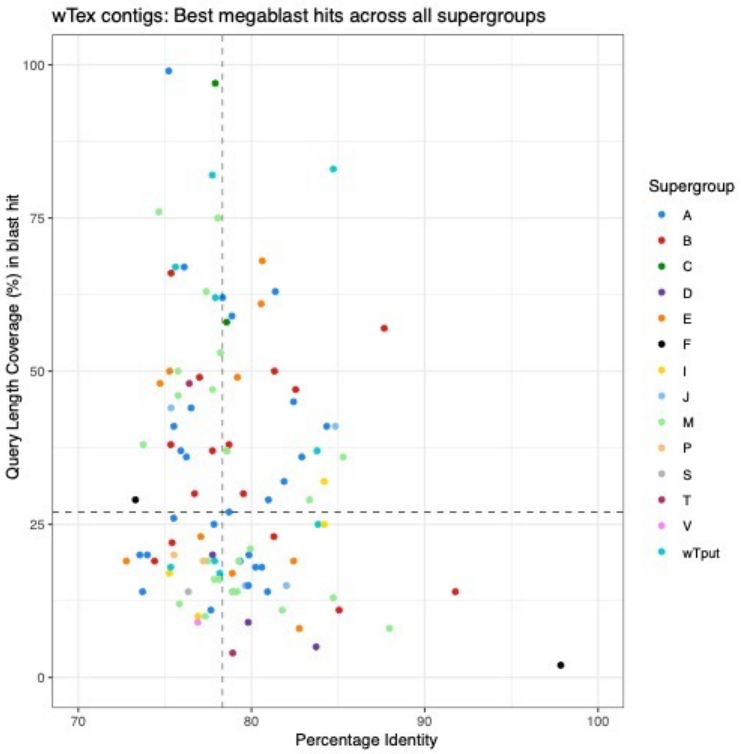

Fig. S3d Megablast hits of *w*Tex contigs across all *Wolbachia* supergroups

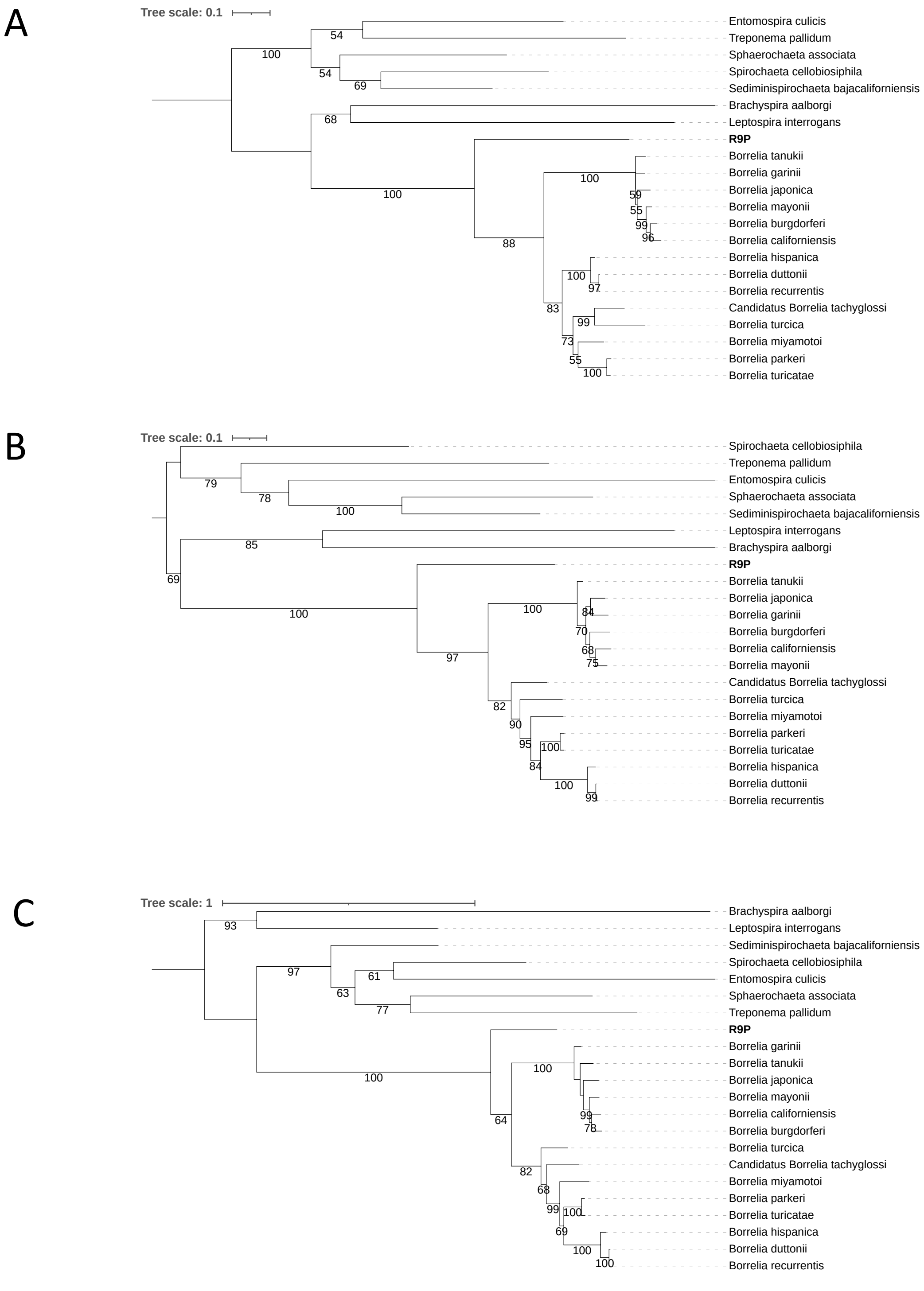

Fig. S4 Maximum-likelihood tree based on (A) *clpX*, (B) *recG* and (C) *uvrA* sequences of *Spirochaetia* including *P. agamae* (in bold) from Saudi Arabia. Best fit model were determined as (A) TPM3u+F+I+G4, (B) TPM2u+F+I+R3 and (C) TPM3u+F+I+G4. Number of nucleotide sites used were (A) 1,056, (B) 1,006 and (C) 1,607. The trees were rooted mid-point.

Table S5 NCBI Genbank accession numbers for *Borrelia* MLST gene sequences used in phylogenetic analyses

Species	MLST genes		
	**clpX**	**recG**	**uvrA**
Borreliella japonica	NZ_CP124066.1	NZ_CP124066.1	NZ_CP124066.1
Borreliella californiensis	NZ_CP124076.1	NZ_CP124076.1	NZ_CP124076.1
Borreliella mayonii	CP015780.1	CP015780.1	CP015780.1
Borreliella burgdorferi	CP124108.1	CP124108.1	CP124108.1
Borreliella tanukii	NZ_CP124038.1	NZ_CP124038.1	NZ_CP124038.1
Borrelia garinii	CP003151.1	CP003151.1	CP003151.1
Borrelia turcica	NZ_CP028884.1	NZ_CP028884.1	NZ_CP028884.1
Candidatus Borreliella tachyglossi	NZ_CP025785.1	NZ_CP025785.1	NZ_CP025785.1
Borrelia hispanica	NZ_AYOU01000121.1	NZ_AYOU01000121.1	NZ_AYOU01000121.1
Borrelia recurrentis	NC_011244.1	NC_011244.1	NC_011244.1
Borrelia duttonii	CP000976.1	CP000976.1	CP000976.1
Borrelia miyamotoi	NZ_CP021872.1	NZ_CP021872.1	NZ_CP021872.1
Borrelia turicatae	CP073192.1	CP073192.1	CP073192.1
Borrelia parkeri	CP073159.1	CP073159.1	CP073159.1
Spirochaeta lutea	NZ_JNUP01000052.1	NZ_JNUP01000066.1	NZ_JNUP01000003.1
Sediminispirochaeta bajacaliforniensis	NZ_KB899417.1	NZ_KB899409.1	NZ_KB899419.1
Sphaerochaeta associata	CP094929.1	CP094929.1	CP094929.1
Treponema pallidum	CP003115.1	CP003115.1	CP003115.1
Brachyspira pilosicoli	CP098754.1	CP098754.1	CP098754.1
Leptospira interrogans	CP096129.2	CP096129.2	CP096129.2

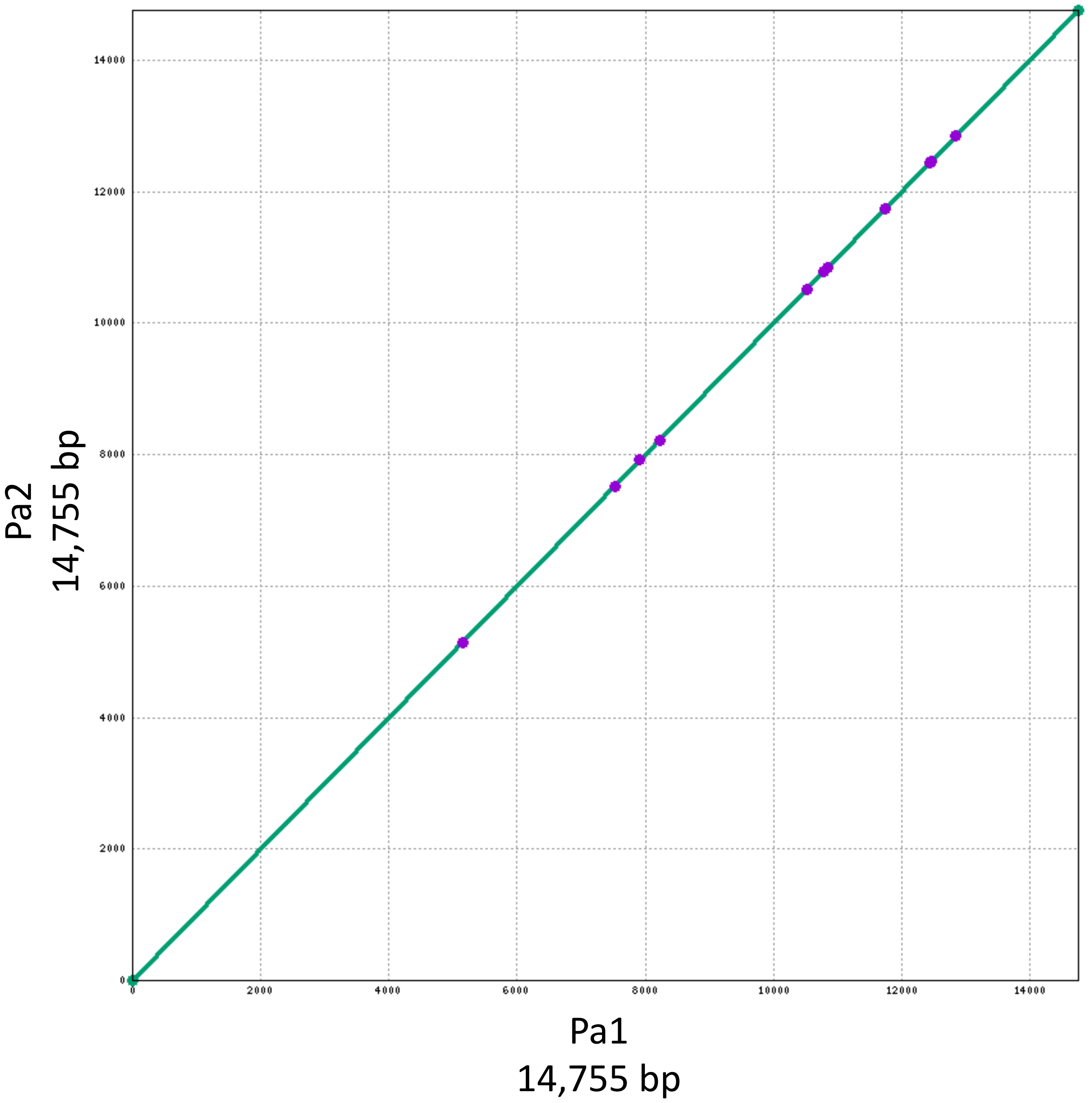

Fig. S5a Alignment of mitochondrial assembly (14,755 bp) from *Pentidionis agamae* pools Pa1 and Pa2. Green region represents 100% identity. Purple region indicates nucleotide differences between both assemblies.

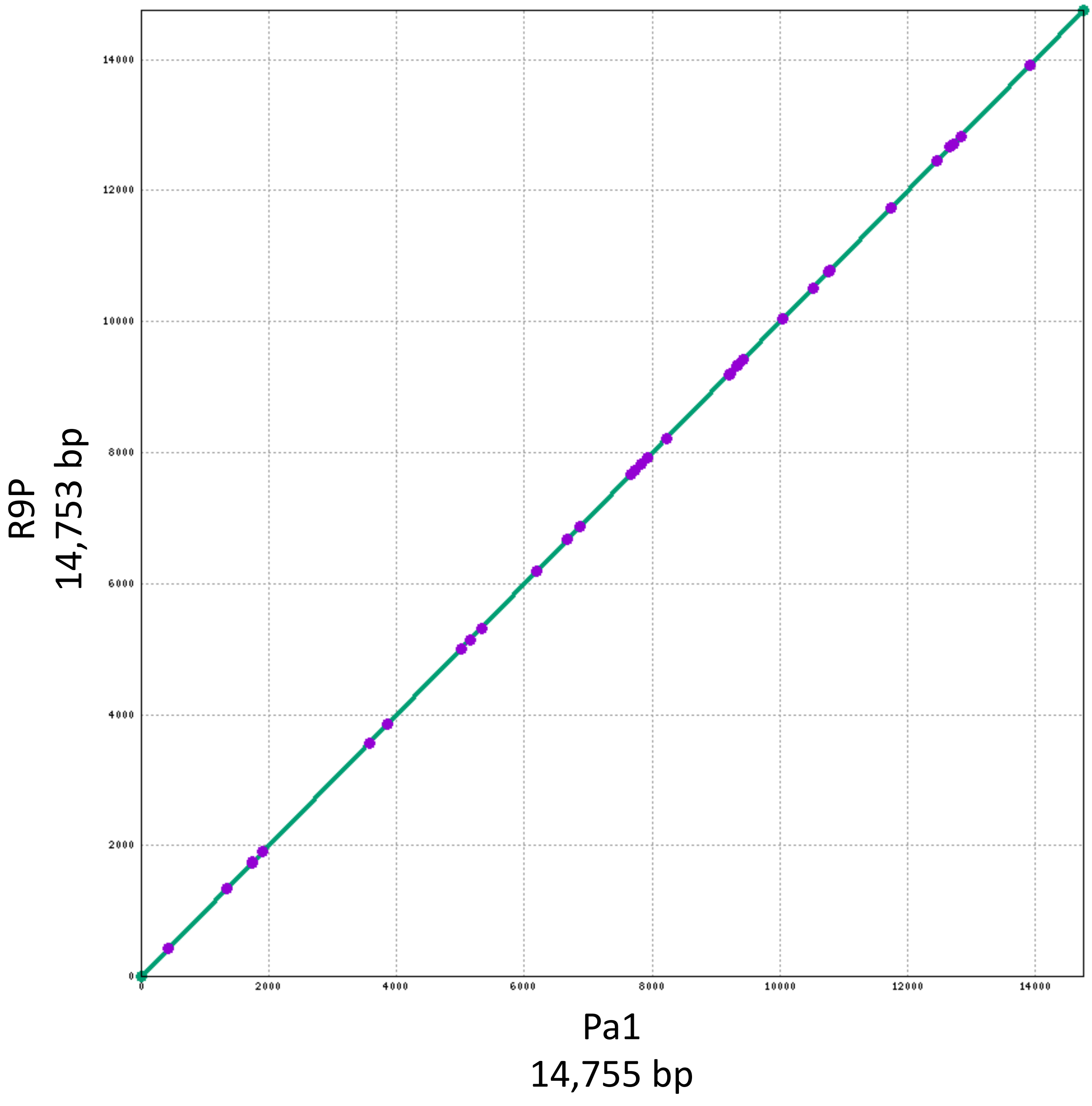

Fig. S5b Alignment of mitochondrial assembly from *Pentidionis agamae* pools Pa1 and R9P. Green region represents 100% identity. Purple region indicates nucleotide differences between both assemblies.

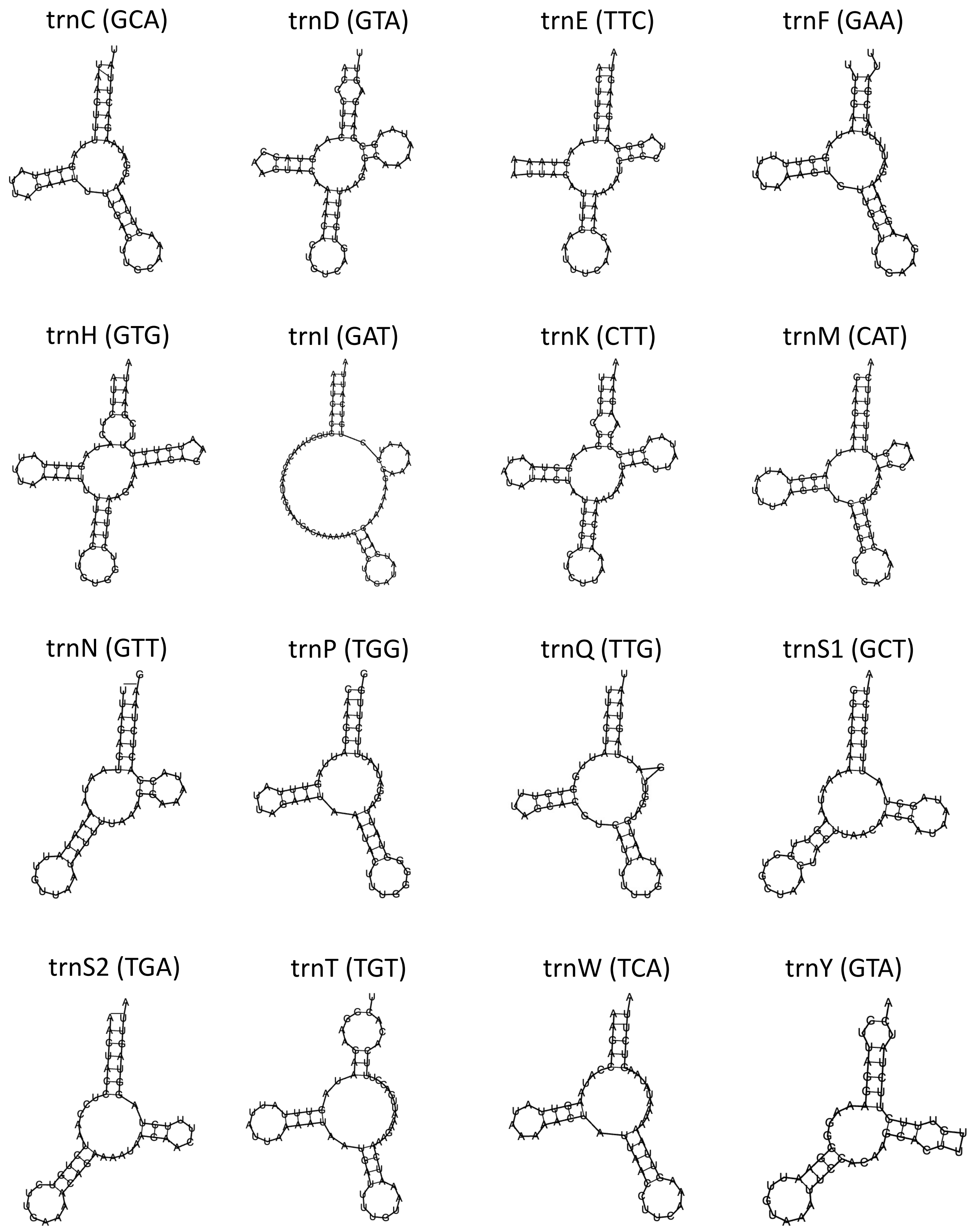

Fig. S6 Predicted mitochondrial tRNAs from *P. agamae* Pa1. MIFTI-predicted secondary structures from the Mitos2 annotation pipeline. Anti-codon sequences are given in the brackets next to the tRNAs.

trnL1 (TAG)

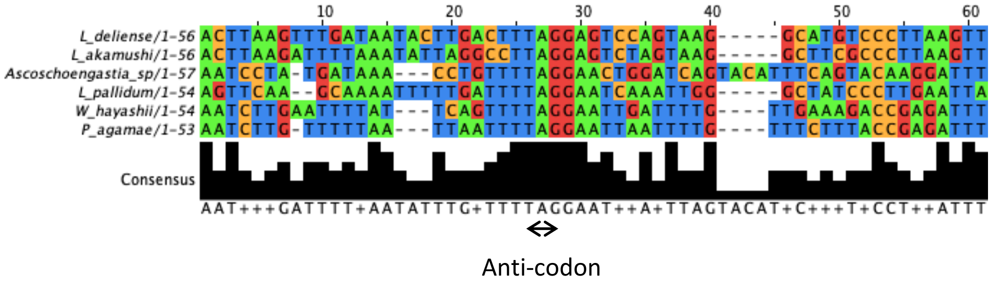

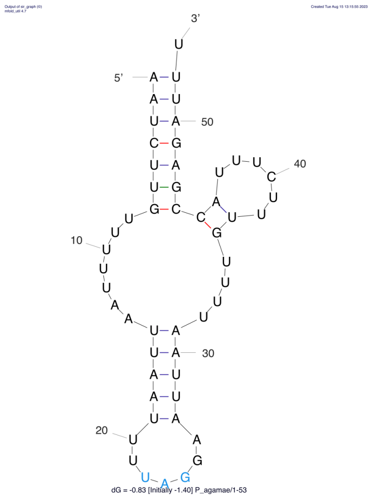

trnL2 (TAA)

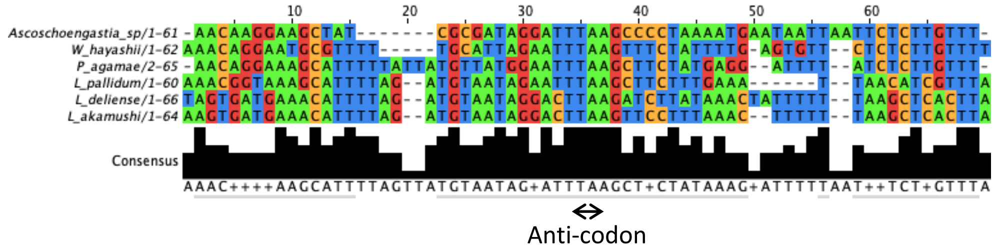

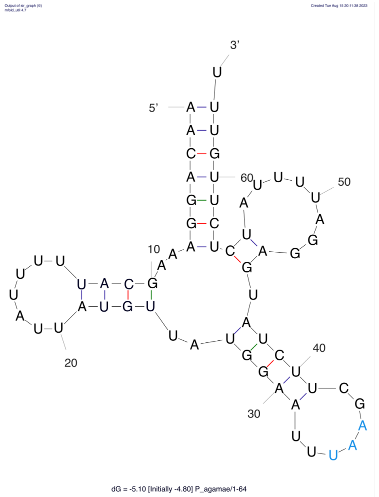

trnG (TCC)

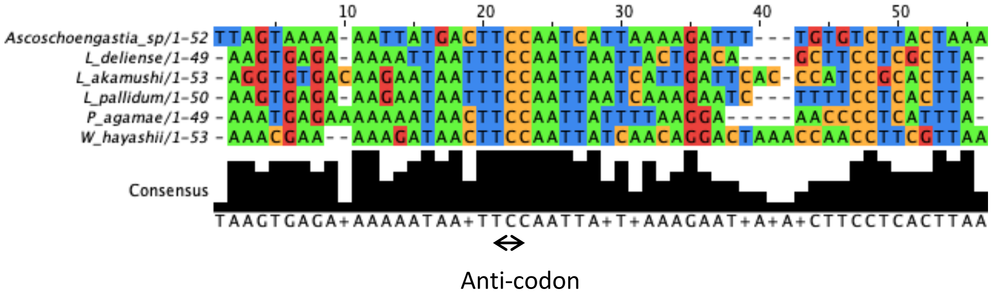

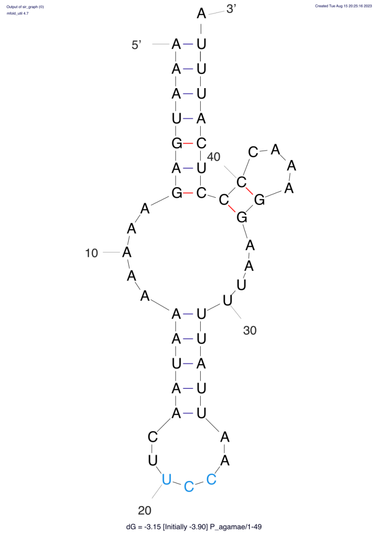

Fig. S7 Alignment of putative mitochondrial tRNA sequences from *P. agamae* Pa1 and other trombiculid mites and the predicted RNA secondary structures. Anti-codon sequences are given in the brackets next to the tRNA and highlighted in blue in the RNA secondary structures.

trnR (TCG)

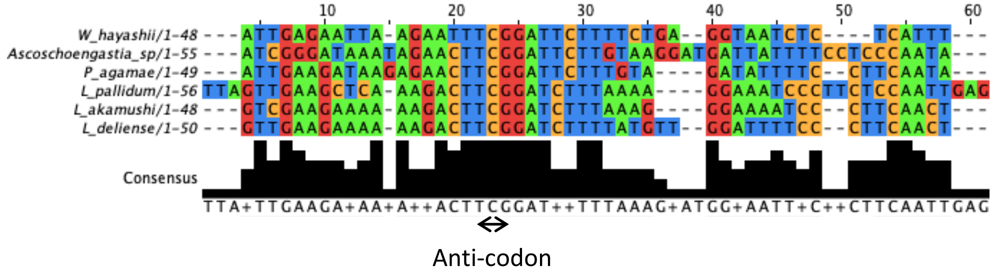

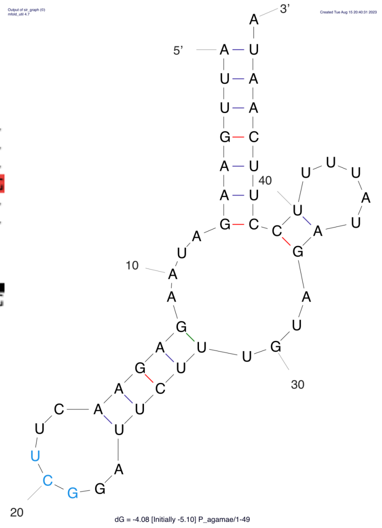

trnV (TAC)

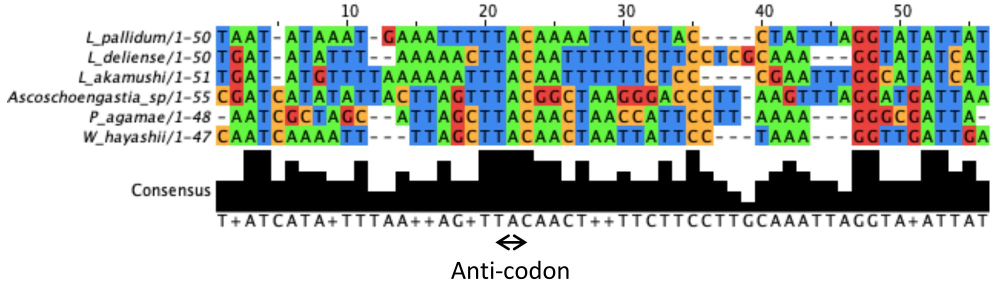

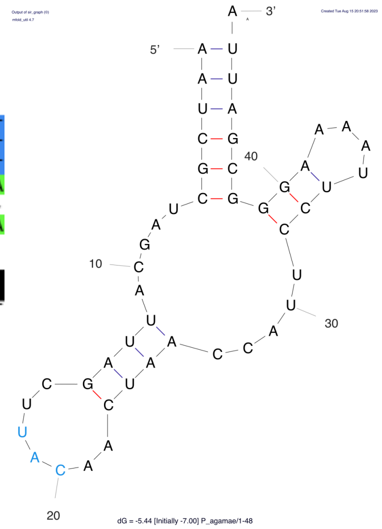

trnA (TGC)

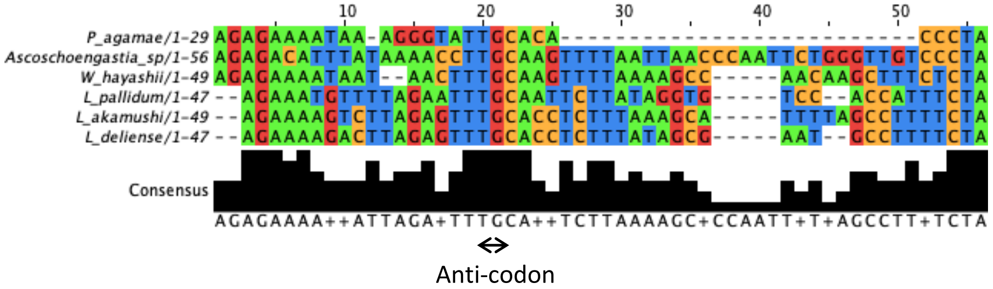

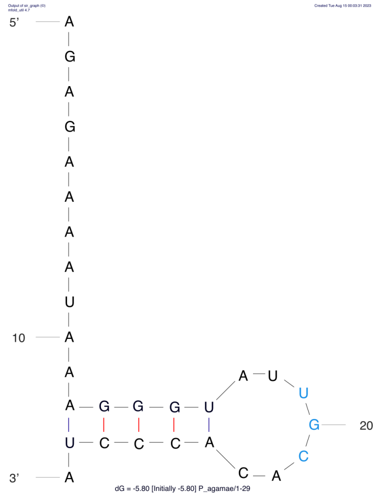

Fig. S7 (continued) Alignment of putative mitochondrial tRNA sequences from *P. agamae* and other trombiculid mites and the predicted RNA secondary structures. Anti-codon sequences are given in the brackets next to the tRNA and highlighted in blue in the RNA secondary structures.

Table. S6 SNPs in *P. agamae* mitochondrial assemblies.

Positions		Nucleotide		Gene/region
Pa1	Pa2	Pa1	Pa2	
5148	5148	C	T	*rrnL*
7518	7518	T	A	*cox3*
7915	7915	C	A	*cox3*
8223	8223	G	A	*trnT*
10518	10518	C	T	*cob*
10788	10788	A	G	*cob*
10844	10844	G	A	*cob*
11745	11745	T	C	*nad5*
12443	12443	C	A	*nad5*
12451	12451	T	A	*nad5*
12458	12458	C	T	*nad5*
12838	12838	A	G	*nad5*

Positions		Nucleotide		Gene/region
Pa1	R9P	Pa1	R9P	
429	429	T	C	*cox1*
1338	1338	T	C	*cox1*
1741	1741	A	C	control region
1747	1746	A	.	control region
1904	1903	T	C	control region
3571	3570	G	A	*nad1*
3857	3856	T	C	*nad1*
5006	5005	T	C	*rrnL*
5009	5007	A	.	*rrnL*
5148	5146	C	T	*rrnL*
5322	5320	T	C	-
6196	6194	T	C	*cox2*
6673	6671	G	A	*atp6*
6864	6862	C	A	*atp6*
7677	7675	A	G	*cox3*
7731	7729	T	C	*cox3*
7819	7817	G	A	*cox3*
7920	7918	C	T	*cox3*
8223	8221	G	A	*trnT*
9196	9194	C	T	*nad4*
9217	9215	T	C	*nad4*
9331	9329	A	C	*nad4*
9337	9335	C	A	*nad4*
9421	9419	C	G	*nad4*
9424	9422	C	T	*nad4*
10039	10037	A	T	*nad6*
10518	10516	C	T	*cob*
10756	10754	A	G	*cob*
10788	10786	A	G	*cob*
11745	11743	T	C	*nad5*
12451	12449	T	A	*nad5*
12458	12456	C	T	*nad5*
12668	12666	G	T	*nad5*
12713	12711	C	A	*nad5*
12838	12836	A	G	*nad5*
13909	13907	G	A	*nad3*
