# Supplementary material for: Microbiome and mitogenomics of the chigger mite Pentidionis agamae: potential role as an Orientia vector and associations with divergent clades of Wolbachia and Borrelia
Source: BMC Genomics. 2024 Apr 17;25:380. doi: 10.1186/s12864-024-10301-6 (PMC11025265; doi:10.1186/s12864-024-10301-6)
Supplement: Supplementary file 2 — Additional file 2: Kronagram for Kraken2 output at confidence threshold 0.1 for Pentidionis agamae pool R9P [file 12864_2024_10301_MOESM2_ESM.html]

Javascript must be enabled to view this page.

members
magnitude
magnitudeUnassigned
count
unassigned
taxon
rank

R9P\_spades\_01\_report


 19.54 17.94 0.00 0.02 0.00 0.01 0.01 0.01 0.01 0.00 0.00 0.00 0.00
718049
5544
13
14192

1
117
333
10
superkingdom

 0.06
10239

0
1
4
14
12333
no rank

 0.00

1
2
1
0
no rank

 0.00
156614

1
1
species

 0.00
278008

species

 0.00
38018
13
1

0
173
39
1
2731341

 0.04
clade


 0.04
kingdom
2731360
173
38
1
0

173
1
37
0

 0.04
phylum
2731618


 0.04
class
2731619
36
1
173
10

0
1
1
5
2842327
family

 0.00

subfamily

 0.00
2732588
4
1
1
0

2732589
genus

 0.00
0
1
3
1

1
2
1
0
species

 0.00
2732591

1958907
no rank

 0.00
1
1

2560065

 0.00
family
0
1
3
2

no rank

 0.00
2572872
2
1
2
0

2831613
species

 0.00
1
2

93
no rank
9
2788787

1
1
species

 0.00
2911157

species

 0.00
2827830
3
1

2828150

 0.00
species
1
1


 0.01
species
2170413
66
1

13
1

 0.00
species
2202564

species

 0.00
2825082
1
1

1
6

 0.00
species
2202567

1
1

 0.00
species
1636270

2827828
species

 0.00
1
1

2
1
2843418
genus

 0.00

1
1
family

 0.00
2946160

2560132
genus

 0.00
1
1
4
5

0
1
1
2
2562338
no rank

 0.00

1
1

 0.00
species
2575350

species

 0.00
2560476
1
3

1
1

 0.00
subfamily
1982875

1
1
1982251

 0.00
genus

1978007
order

 0.00
0
2
1
1

1
1
species

 0.00
2781373

family

 0.00
2731643
6
1
6
0

no rank

 0.00
2731990
1
2
4
0

2723762
species

 0.00
4
1

0
2
1
3
2731949

 0.00
genus

0
1
2
2
2733926
species

 0.00

no rank

 0.00
1983784
2
1

2100420

 0.01
no rank
0
50
1
2


 0.01
species
2100421
1
50

clade

 0.00
2559587
1
10
2
0

0
2
1
9
2732397
kingdom

 0.00

8
1
2
0
phylum

 0.00
2732409

2
7
1
0
class

 0.00
2732514

2169561

 0.00
order
0
2
6
1

0
2
5
1
11632

 0.00
family

1
1
no rank

 0.00
35276

3
1
1
0
subfamily

 0.00
327045

1
2
1
0

 0.00
genus
11646

1
1
species

 0.00
11676

no rank

 0.00
186616
1
2
4
0

species

 0.00
340016
4
1

no rank

 0.01
12429
12
1
56
0

1
26

 0.00
species
2963861

1608440

 0.00
species
1
9

21
1
9
0
no rank

 0.00
2204151

13
1
2749196

 0.00
species

0
1
6
7
35342
no rank

 0.00

family

 0.00
2058757
1
4
6
0


 0.00
species
2761503
1
4

2502018

 0.00
clade
0
1
2
2


 0.00
no rank
2569970
1
2

1
1

 0.00
species
2502796

1
1
2839052
species

 0.00

0
11
1
3
12877

 0.00
clade

11
1
2
0
clade

 0.00
2609695

1
11

 0.00
species
2963853

1
46
63
0
clade

 0.01
2731342

2732092

 0.01
kingdom
0
37
1
53

2732416

 0.00
phylum
2
1
16
33

2748377
no rank

 0.00
2
5
1
25

2875809

 0.00
species
13
1

clade

 0.00
2202562
9
2
1
0

1
9
2202563

 0.00
species

1
1
2859451

 0.00
species

0
1
5
1
2732424
class

 0.00

0
1
4
1
2732539
order

 0.00

0
1
1
3
1910928
family

 0.00

no rank

 0.00
1941235
1
2
1
0


 0.00
species
2814944
1
1

class

 0.00
2732423
5
1
5
0

order

 0.00
2732536
1
4
5
0

39724
family

 0.00
0
1
3
5

0
5
1
2
642248
no rank

 0.00

5
1

 0.00
species
1788444

20
1
20
0

 0.00
phylum
2732415

6
1
2
0

 0.00
class
2732421

0
2
5
1
2732533
order

 0.00

151340

 0.00
family
1
1
4
2

333774

 0.00
no rank
0
1
3
1


 0.00
clade
173087
1
2
1
0

1
1
species

 0.00
10566

18
1
13
0
class

 0.00
2732422

1
12
18
0

 0.00
order
2732534

18
1
11
0

 0.00
family
10780

no rank

 0.00
535600
13
1
3
1

2794528
species

 0.00
1
11

2794463

 0.00
species
1
1

5
7
1
0
subfamily

 0.00
40119

0
2
1
3
10803

 0.00
genus

1511895
species

 0.00
0
2
2
1

1
2
no rank

 0.00
341673

3
3
1
0
no rank

 0.00
207678

1341019
species

 0.00
1
3
1
2

1
2
1395615
no rank

 0.00


 0.00
kingdom
2732091
10
1
8
0

0
10
1
7
2732412

 0.00
phylum

class

 0.00
2732413
1
6
10
0

10
1
5
0

 0.00
order
2732414

family

 0.00
10841
10
4
1
1

3
3
1
9
117574
no rank

 0.00

2202644

 0.00
species
1
5

1
1

 0.00
species
2202559

2
1
5
0

 0.00
no rank
2787854

no rank

 0.00
28384
2
1
4
0

no rank

 0.00
81077
1
3
2
1

111786

 0.00
no rank
0
2
1
1

species

 0.00
111789
1
1

2759

 12.70
superkingdom
5404
3323
1
91200

85012
2753
1
954

 11.84
clade
33154

4751
kingdom

 0.16
2
304
1
1130

4
1
293
1123
451864

 0.16
subkingdom

223
1
347
0

 0.05
phylum
4890

344
215
1
5
clade

 0.05
716545

1
35
80
0

 0.01
subphylum
147537

0
80
34
1
4891

 0.01
class

4892

 0.01
order
0
1
33
80

family

 0.00
115784
3
1
3
0

genus

 0.00
460517
1
2
3
0

1
3
species

 0.00
4922

1
3
1
0

 0.00
family
34353

0
2
1
1
4951
genus

 0.00

4952
species

 0.00
1
1

57
6
1
0

 0.01
family
4893

32
55
1
2
4930

 0.01
genus

23
1
4932

 0.00
species

33170

 0.00
genus
0
3
1
2

45285

 0.00
species
0
2
2
1

1
2

 0.00
strain
931890

1
15
18
1
clade

 0.00
2916678

family

 0.00
27319
3
1
3
0

0
3
2
1
27320

 0.00
genus

1
3
species

 0.00
2163413

0
1
11
14
766764

 0.00
family


 0.00
genus
412764
1
2
2
0

561895

 0.00
species
2
1


 0.00
clade
1535325
9
3
1
0

9
1
2
0
genus

 0.00
5475

1
9

 0.00
species
5480

2
1
2
0

 0.00
genus
507510

2
1
species

 0.00
717740

1
3
1
0
genus

 0.00
766728

2
1
1
0
species

 0.00
4929

294746
strain

 0.00
1
1

0
1
5
1
2926619
clade

 0.00


 0.00
family
34366
4
1
1
0

0
1
1
3
4943
genus

 0.00

1
2
1
0
no rank

 0.00
2636529

1
1

 0.00
species
2826930

147538

 0.04
subphylum
2
259
179
1

18
257
1
178
716546
clade

 0.04

84
1
105
4

 0.01
clade
715989

0
8
1
7
147548

 0.00
class

0
7
1
7
5178

 0.00
order

0
7
6
1
28983
family

 0.00

5179

 0.00
genus
1
1

0
1
4
6
33196

 0.00
genus

40559
species

 0.00
0
2
2
1

332648
strain

 0.00
1
2

species

 0.00
1964551
4
1

class

 0.01
147550
75
1
94
18

43
1
63
9

 0.01
subclass
222543

1028384
order

 0.00
0
7
1
2

0
1
6
2
681950
family

 0.00

genus

 0.00
5455
1
5
2
0


 0.00
no rank
2872341
1
2
1
0

species

 0.00
1543685
1
1

2707335

 0.00
no rank
0
2
1
1

species

 0.00
145971
1
1


 0.01
order
5125
35
1
52
18


 0.00
family
110618
1
11
9
0

5506
genus

 0.00
1
10
1
9

species group

 0.00
171631
3
1
2
0

0
2
1
2
5507
species

 0.00

660027
strain

 0.00
2
1


 0.00
species group
450425
1
2
1
0

231269

 0.00
species
1
1

171627
species group

 0.00
0
4
2
1

species

 0.00
5127
4
1

0
1
2
1
569360

 0.00
species group

1
1
101028

 0.00
species

4
1
1
0
no rank

 0.00
162454

0
1
3
1
159075
genus

 0.00

1
1
2
0
no rank

 0.00
2689091

1
1

 0.00
species
305424

5
1
6
2

 0.00
family
34397

124426
genus

 0.00
0
1
2
1

1
1
species

 0.00
1159556

5112
genus

 0.00
0
2
1
3

5113

 0.00
species
1
1

species

 0.00
42805
1
1

14
7
1
8

 0.00
family
474943

2
4
1
4
45234

 0.00
genus

1
1
73501
species

 0.00

0
1
2
1
114497
species

 0.00

1
1
1081104

 0.00
strain


 0.00
genus
5581
2
1
2
0


 0.00
species
176275
2
1

5
6
1
0
family

 0.00
5129

genus

 0.00
5543
5
1
5
0

species

 0.00
5544
2
1
1
0

strain

 0.00
983964
1
1

2
1
51453
species

 0.00

1
2
species

 0.00
63577

subclass

 0.00
222544
1
26
12
1

0
1
4
1
639021

 0.00
order


 0.00
family
2528436
1
1
3
0

1
1
2
0

 0.00
genus
48558

1
1
318829

 0.00
species


 0.00
order
5114
2
1
9
0

1
1
5
0
family

 0.00
399129

no rank

 0.00
218105
1
4
1
0

305399
genus

 0.00
0
1
3
1

2029752
species

 0.00
1
1

2625752

 0.00
no rank
1
0

1
3
1
0
family

 0.00
767018

genus

 0.00
36922
1
2
1
0


 0.00
species
748121
1
1

8
1
12
0

 0.00
order
5139

1
4
1
0
family

 0.00
5148

genus

 0.00
5146
1
3
1
0

2
1
1
0

 0.00
species
5147

1
1
strain

 0.00
771870


 0.00
family
35718
1
7
7
4

2609811
genus

 0.00
0
2
1
3

2587410
species

 0.00
0
2
1
2

2
1
578455
strain

 0.00

1
3
1
0
genus

 0.00
5149

1
2
1
0

 0.00
species
38033

1
1

 0.00
strain
306901


 0.00
subclass
222545
5
1
1
0

1
1
4
0

 0.00
order
37989


 0.00
family
2033035
1
1
3
0


 0.00
genus
42360
1
1
2
0

1
1
326647
species

 0.00

1
1
8
0

 0.00
class
147547


 0.00
clade
1520881
7
1
1
0

388435

 0.00
subclass
0
1
6
1

0
1
5
1
5197

 0.00
order

157822
suborder

 0.00
0
1
4
1

family

 0.00
56478
1
1
3
0

93111
genus

 0.00
0
1
1
2

1
1
2732470
species

 0.00

0
92
1
46
147545

 0.01
class

1
8
5
0
subclass

 0.00
451870

0
5
7
1
34395

 0.00
order

43219

 0.00
family
0
5
1
6

genus

 0.00
5583
4
1
3
0

91925
species

 0.00
1
2

2
1
215243
species

 0.00

1
2
1
0

 0.00
genus
82105

569365
species

 0.00
1
1

1
37
87
0

 0.01
subclass
451871

86
1
31
4
order

 0.01
5042

1131492

 0.01
family
1
82
1
30

13
75
1
22
5052
genus

 0.01

138277

 0.00
species
1
14

2720874
subgenus

 0.00
0
3
1
3

3
2
1
0

 0.00
species
41413

3
1

 0.00
strain
1160497


 0.00
species
1220207
1
1

33
5
1
0

 0.00
subgenus
2720870


 0.00
species
75750
1
2
22
0

1036612
strain

 0.00
22
1

46472

 0.00
species
0
11
1
2

1
11
strain

 0.00
1036611

2
1
4
0
subgenus

 0.00
2720872

746128

 0.00
species
1
4


 0.00
subgenus
2720871
2
1
3
0


 0.00
species
306088
2
1
2
0


 0.00
strain
1392250
1
2

1
1
species

 0.00
319627

182096
species

 0.00
1
1

1
2
1
0
species

 0.00
51019

1
1
strain

 0.00
1448321

1
1
species

 0.00
1220188

species

 0.00
41058
1
1

5073

 0.00
genus
0
4
1
1

1
3
1
0
no rank

 0.00
254878

1108849

 0.00
species
0
1
2
1

strain

 0.00
500485
1
1

0
1
3
5
70110
genus

 0.00

species

 0.00
41063
5
2
1
0


 0.00
strain
1073090
5
1

33183
order

 0.00
0
5
1
1

0
1
4
1
33184
family

 0.00

0
1
1
3
5500

 0.00
genus


 0.00
species
199306
1
2
1
0

1
1
strain

 0.00
443226

41
1
39
0

 0.01
clade
715962

147541
class

 0.01
2
1
38
41

subclass

 0.00
451867
1
1
4
0

2726946

 0.00
order
0
1
3
1

1
2
1
0
family

 0.00
452563

5498
genus

 0.00
1
1

0
1
4
1
159987

 0.00
no rank

2810619
genus

 0.00
0
1
1
3

1
2
1
0
species

 0.00
61459

1
1
1168221
strain

 0.00

0
1
29
37
451868
subclass

 0.01

order

 0.01
92860
28
1
37
0

suborder

 0.01
715340
37
1
27
1

35
1
23
0

 0.00
family
28556

genus

 0.00
5502
1
1
2
0

95742

 0.00
species
1
1

0
1
3
1
33194

 0.00
genus

species

 0.00
101162
2
1
1
0

strain

 0.00
930090
1
1

91493

 0.00
genus
0
3
1
1


 0.00
species
93612
1
1
2
0

1
1
671987

 0.00
strain

genus

 0.00
5598
14
1
32
2

2
1
1
0

 0.00
section
2499262

1
1
48100

 0.00
species

2499258
section

 0.00
17
8
1
27

2
1

 0.00
species
566460

1
1
1187951
species

 0.00

2
1
430562

 0.00
species

2
1
297637
species

 0.00

1
1
181014

 0.00
species

species

 0.00
1187925
1
1

1
1

 0.00
species
283354


 0.00
section
2499270
1
1
2
0

1
1
1187941
species

 0.00

1
1
2499237
section

 0.00

0
1
3
1
683158
family

 0.00

749461

 0.00
genus
0
1
1
2

1
1
species

 0.00
749465

451866

 0.00
subphylum
0
3
1
7

0
3
1
6
147554

 0.00
class

34346

 0.00
order
0
3
5
1

4894

 0.00
family
0
1
4
3

4895

 0.00
genus
0
1
3
3

3
1
2
0
species

 0.00
866546

3
1

 0.00
strain
653667

5204
phylum

 0.11
0
1
69
772

452284

 0.10
subphylum
1
751
16
1

0
1
6
2
5257

 0.00
class

5267

 0.00
order
0
2
5
1

0
2
4
1
5268

 0.00
family

0
3
1
2
5269

 0.00
genus


 0.00
species
5270
1
2
2
0

237631
strain

 0.00
2
1

1538075
class

 0.10
0
9
1
748

748
1
8
0

 0.10
order
162474

family

 0.10
742845
748
1
7
0

2
748
1
6
55193
genus

 0.10

76773

 0.01
species
1
76

2
1

 0.00
species
55194

76775
species

 0.09
533
1
2
666

425264

 0.02
strain
133
1


 0.00
species
76777
2
1

5302
subphylum

 0.00
0
19
45
1

155619
class

 0.00
0
34
1
12

355688
no rank

 0.00
0
1
15
8

452342

 0.00
order
0
7
10
1

1
4
1
0

 0.00
family
103376

5644
genus

 0.00
0
1
3
1

1
2
1
0
species

 0.00
40492

1
1

 0.00
strain
721885

6
5
1
0
family

 0.00
40420

6
1
4
0

 0.00
genus
13562

256003

 0.00
no rank
0
6
1
3

3
6
2
1
984962
species

 0.00

3
1

 0.00
strain
747525

5303

 0.00
order
0
1
4
1


 0.00
family
5317
1
3
1
0

5324

 0.00
genus
0
1
2
1

1
1
5327
species

 0.00

452333

 0.00
subclass
0
18
1
4

1
5
1
0
order

 0.00
68889

227332
suborder

 0.00
0
1
4
1

3
1
1
0

 0.00
family
227336

5379

 0.00
genus
0
1
1
2

1
1
48587

 0.00
species

0
1
12
3
5338

 0.00
order

2982305

 0.00
suborder
0
1
1
4

5339
family

 0.00
0
1
3
1

0
2
1
1
5340
genus

 0.00

species

 0.00
5341
1
1

7
1
2
0
suborder

 0.00
2982303

5351
family

 0.00
0
1
1
3

1
2
1
0

 0.00
genus
40144

species

 0.00
40145
1
1

930979
family

 0.00
0
3
1
1

47720

 0.00
genus
0
1
1
2

1
1
species

 0.00
64660

0
7
1
10
155616

 0.00
class

7
1
9
0
order

 0.00
5234

1884633

 0.00
family
0
7
1
8

3
3
1
0
genus

 0.00
5206

species group

 0.00
1897064
3
1
2
0

3
1

 0.00
species
5207

4
4
1
0

 0.00
genus
490731

1734106
species

 0.00
3
1

1
1
2
0
species

 0.00
453459


 0.00
strain
1296096
1
1

29000

 0.00
subphylum
0
2
1
7

0
1
6
2
162484

 0.00
class

2
1
5
0
order

 0.00
5258


 0.00
family
5262
1
4
2
0


 0.00
genus
5296
2
3
1
0

27350

 0.00
species
0
2
1
2

168172

 0.00
forma specialis
2
1

no rank

 0.00
57731
2
1
1
0

1
1

 0.00
species
175245

no rank

 0.00
112252
8
1
4
0

7
1
4
0

 0.00
phylum
1913637

214504
subphylum

 0.00
0
4
6
1

214506
class

 0.00
0
5
1
4

36750
order

 0.00
0
4
4
1

36751

 0.00
family
0
1
3
4

0
4
2
1
1129544

 0.00
genus

species

 0.00
588596
4
1

82928
2448
1
89

 11.55
kingdom
33208

0
1
7
3
6040

 0.00
phylum

class

 0.00
6042
6
1
3
0

5
1
3
0
subclass

 0.00
1779146

4
1
3
0
order

 0.00
6049

3
3
1
0

 0.00
family
68562

genus

 0.00
68563
3
1
2
0

species

 0.00
68564
3
1

166
1
2440
82836
6072

 11.54
clade

6073

 0.00
phylum
0
11
1
29

1927913

 0.00
class
0
3
1
6


 0.00
order
37528
5
1
3
0

0
4
1
3
1927915

 0.00
suborder

0
3
3
1
1927917

 0.00
family

2
1
3
0

 0.00
genus
37533


 0.00
species
313498
3
1

1
7
2
0

 0.00
class
6074

37516

 0.00
subclass
0
2
6
1

order

 0.00
406427
2
5
1
0

0
1
4
2
406428

 0.00
suborder

6094
family

 0.00
0
1
3
2

6095

 0.00
genus
0
1
2
2

species

 0.00
6096
1
2

6101

 0.00
class
0
15
1
6

6102
subclass

 0.00
0
1
14
6

0
1
8
5
6103

 0.00
order


 0.00
family
45349
1
1
3
0

45350
genus

 0.00
0
2
1
1

species

 0.00
45351
1
1

0
4
1
4
86626

 0.00
suborder

0
1
3
4
478428
family

 0.00

0
4
1
2
478394
genus

 0.00

1789172
species

 0.00
4
1

6125
order

 0.00
0
1
5
1

123760

 0.00
suborder
0
1
4
1

1
3
1
0

 0.00
family
46736


 0.00
genus
1920453
1
1
2
0

1
1
species

 0.00
48498

8666
82659
2410
1
33213

 11.51
clade

1
858
64363
13

 8.96
clade
33511

0
1
5
1
10219

 0.00
phylum

0
1
4
1
10220
class

 0.00

0
1
3
1
10221

 0.00
family

1
2
1
0

 0.00
genus
10222

1
1
10224

 0.00
species

7586

 0.00
phylum
0
19
1
5

clade

 0.00
133551
5
1
18
0

1
9
4
0
superclass

 0.00
7587

7588

 0.00
class
0
4
1
8

0
4
1
7
41242

 0.00
superorder

0
4
1
6
7599

 0.00
order

0
4
1
5
7600
family

 0.00

3
2
1
0
genus

 0.00
7608

species

 0.00
7609
1
3

0
1
2
1
7601
genus

 0.00

1
1
species

 0.00
7604

0
1
8
1
7624
superclass

 0.00


 0.00
class
7625
7
1
1
0

7638
subclass

 0.00
0
1
1
6

1
5
1
0
superorder

 0.00
7674

31184

 0.00
order
0
1
4
1

1
3
1
0

 0.00
family
31185

7652

 0.00
genus
0
2
1
1

1
1
7654
species

 0.00

64344
833
1
41

 8.96
phylum
7711

0
1
6
2
7735
subphylum

 0.00

5
1
2
0

 0.00
class
2682552

0
4
1
2
2682553

 0.00
order

family

 0.00
7736
2
1
3
0


 0.00
genus
7737
2
1
2
0

7740
species

 0.00
2
1

4
1
10
0
subphylum

 0.00
7712

7713
class

 0.00
0
4
9
1


 0.00
order
7716
1
4
1
0

0
1
3
1
7717

 0.00
family

7718

 0.00
genus
0
2
1
1

1
1
7719

 0.00
species

32436

 0.00
order
0
1
4
3

family

 0.00
201955
3
1
3
0

1
2
3
0

 0.00
genus
201956

1
3
species

 0.00
2771288

64297
1
816
0
subphylum

 8.95
89593

4
64297
815
1
7742

 8.95
clade

1
808
64292
26
clade

 8.95
7776

0
1
18
37
7777
class

 0.01

0
37
17
1
7778

 0.01
subclass

119203

 0.00
infraclass
0
9
11
1

9
1
10
0

 0.00
clade
119197

119195

 0.00
superorder
0
9
1
9

0
7
4
1
30503
order

 0.00

3
1
7
0

 0.00
family
40580

34767

 0.00
genus
0
1
2
7

1
7
species

 0.00
36176

order

 0.00
30483
4
1
2
0

family

 0.00
7826
3
1
2
0


 0.00
genus
7829
1
2
2
0


 0.00
species
7830
1
2

1
5
28
0

 0.00
superorder
117893

0
28
4
1
7858

 0.00
order

28
1
3
0
family

 0.00
30475


 0.00
genus
117853
28
1
2
0

1
28
386614

 0.00
species


 8.94
clade
117570
64229
789
1
0

64229
1
788
1355
clade

 8.94
117571

3
2093
1
343
7898
superclass

 0.29

337
1
2084
29
class

 0.29
186623

17
1
8
0

 0.00
subclass
32440

7899

 0.00
order
0
7
1
17

suborder

 0.00
186622
6
1
17
0

1
5
17
0
family

 0.00
7900

0
4
1
17
124129
subfamily

 0.00

124130

 0.00
tribe
0
1
3
17

0
2
1
17
7901
genus

 0.00

7906
species

 0.00
1
17

1
328
2038
1
subclass

 0.28
41665


 0.28
infraclass
32443
2037
1
327
1

10
1
326
2036
1489341

 0.28
clade

0
1
9
13
1489343
clade

 0.00

clade

 0.00
31089
13
8
1
0

0
13
7
1
41712
order

 0.00

12
3
1
0
family

 0.00
27723

27726
genus

 0.00
0
12
2
1

12
1
113540
species

 0.00

family

 0.00
31092
1
1
3
0

1
1
2
0
genus

 0.00
91732

1
1
species

 0.00
1676925

1
316
2013
94

 0.28
no rank
186625

4
171
75
1
186634

 0.02
cohort

0
11
1
18
282425

 0.00
subcohort

32446

 0.00
order
0
1
10
18


 0.00
suborder
1489460
1
5
1
0

family

 0.00
55118
1
4
1
0

7948
subfamily

 0.00
0
1
3
1

genus

 0.00
7949
2
1
1
0


 0.00
species
7950
1
1

1489459

 0.00
suborder
0
4
1
17

17
3
1
0
family

 0.00
299319

0
2
1
17
299320

 0.00
genus

1
17

 0.00
species
299321

subcohort

 0.02
32519
149
1
63
3


 0.02
clade
186626
125
1
56
5

105
26
1
0
superorder

 0.01
186627

7952
order

 0.01
0
105
1
25

1
4
8
0

 0.00
suborder
30725

278171
family

 0.00
0
1
3
8

0
2
1
8
160394

 0.00
genus

8
1
135647
species

 0.00

5
97
20
1
30727
suborder

 0.01

0
1
4
1
2743726
family

 0.00

2743731

 0.00
subfamily
0
1
3
1

1
1
2
0

 0.00
genus
51137

species

 0.00
90988
1
1

7953
family

 0.01
0
1
9
45

38
1
5
0
subfamily

 0.01
2743694

75365
genus

 0.00
0
1
1
2

1
1

 0.00
species
75366


 0.01
genus
7961
37
1
2
0


 0.01
species
7962
37
1

2743693

 0.00
subfamily
0
3
1
7

40829

 0.00
genus
0
7
1
2


 0.00
species
40830
1
7

2743709

 0.01
family
0
46
6
1

subfamily

 0.01
2743711
46
1
5
0

4
1
46
1

 0.01
genus
7954

1142201
species

 0.00
1
15

19
1
7955
species

 0.00

242068

 0.00
species
11
1


 0.00
superorder
186628
15
29
1
0

1
14
12
0
order

 0.00
7995

5
1
1
0
suborder

 0.00
1489791

31002
family

 0.00
0
1
4
1

1
3
1
0
subfamily

 0.00
503143

2
1
1
0

 0.00
genus
52091

2019912
species

 0.00
1
1

0
11
1
8
1489793
suborder

 0.00

0
3
3
1
7999

 0.00
family

3
2
1
0

 0.00
genus
30992


 0.00
species
310915
3
1

30989

 0.00
family
0
1
4
8

0
1
3
8
94992

 0.00
genus

1
1

 0.00
species
175797

7
1
933932

 0.00
species

0
2
9
1
7991

 0.00
order

suborder

 0.00
1489739
8
1
2
0

7992

 0.00
family
0
4
1
1

0
1
1
3
42595
subfamily

 0.00

7993
genus

 0.00
0
2
1
1

species

 0.00
7994
1
1


 0.00
family
42495
3
1
1
0

genus

 0.00
42525
1
2
1
0

1
1
42526

 0.00
species

1
5
1
0
order

 0.00
8002

4
1
1
0
suborder

 0.00
1489620

30771
family

 0.00
0
3
1
1

8004

 0.00
genus
0
1
1
2

8005

 0.00
species
1
1

186633
clade

 0.00
0
6
1
21

21
1
5
0

 0.00
order
29140

186632

 0.00
suborder
0
4
1
21

21
3
1
0

 0.00
family
29142

29143

 0.00
genus
0
21
1
2

species

 0.00
29144
21
1


 0.24
cohort
1489388
1748
240
1
388

0
19
1
824
41705
clade

 0.11

order

 0.11
8006
18
1
824
0

family

 0.11
8015
824
1
17
99

504567
subfamily

 0.06
0
421
4
1

27772
genus

 0.06
1
1
3
421

2
1
420
0

 0.06
no rank
2649731

420
1
861768
species

 0.06

504568
subfamily

 0.04
30
1
12
304

6
216
1
2
8028
genus

 0.03


 0.03
species
8032
210
1

8016
genus

 0.01
23
57
7
1

8022

 0.00
species
1
4


 0.00
species
8017
1
5

3
1
8019
species

 0.00

74940
species

 0.00
1
3

8018

 0.00
species
1
18

8023

 0.00
species
1
1

0
1
2
1
8033
genus

 0.00

8040
species

 0.00
1
1

0
536
220
1
123365
clade

 0.07

0
536
1
219
123366
clade

 0.07

536
1
218
0

 0.07
clade
123367

clade

 0.07
123368
536
1
217
6

0
12
1
8
1489838
clade

 0.00

0
12
1
7
1489841
clade

 0.00


 0.00
clade
1489843
6
1
12
0

12
5
1
0

 0.00
order
8043


 0.00
suborder
1489845
12
4
1
0

0
12
1
3
8045
family

 0.00


 0.00
genus
8048
12
1
2
0

8049
species

 0.00
1
12

1
208
518
9

 0.07
clade
123369

11
6
1
0

 0.00
clade
181483

0
1
5
11
1490028
order

 0.00

47697
family

 0.00
0
1
4
11

47698

 0.00
subfamily
0
11
1
3

0
11
2
1
47699
genus

 0.00

1
11
586833
species

 0.00

141
498
1
201
1489872
clade

 0.07


 0.03
clade
1489922
1
59
197
11

1489923
no rank

 0.00
0
11
6
1

5
1
11
0

 0.00
family
30870

3
2
1
0

 0.00
genus
215359

1
3

 0.00
species
240163

genus

 0.00
215357
2
1
8
0


 0.00
species
215358
1
8

1
1
6
0

 0.00
order
31022


 0.00
suborder
31028
1
5
1
0

1
4
1
0

 0.00
superfamily
32517


 0.00
family
31031
1
3
1
0

2
1
1
0

 0.00
genus
31032


 0.00
species
31033
1
1

1489928

 0.00
order
0
1
4
1

0
3
1
1
8247

 0.00
family

98381
genus

 0.00
0
1
2
1

1
1

 0.00
species
1203425

0
73
4
1
1489939

 0.01
order

1204718

 0.01
family
0
73
3
1

8163
genus

 0.01
0
1
2
73

73
1
species

 0.01
315492

1
6
6
0
order

 0.00
1489931

6
5
1
0

 0.00
family
8169

8176
genus

 0.00
0
1
2
1

1
1
8177

 0.00
species


 0.00
genus
8174
1
2
5
0

1
5
8175
species

 0.00


 0.01
order
8111
94
32
1
1


 0.00
suborder
8100
10
1
25
0

0
11
5
1
8192
infraorder

 0.00

56724

 0.00
family
0
11
1
4

181468

 0.00
subfamily
0
1
3
11

1
2
11
0

 0.00
genus
56725

11
1
56726
species

 0.00

1490021
infraorder

 0.00
0
1
4
14

8092
family

 0.00
0
14
1
3

0
1
2
14
61642
genus

 0.00

14
1
61643

 0.00
species

1
6
1
0

 0.00
suborder
8112

1
1
5
0
family

 0.00
8165

0
1
4
1
698016
subfamily

 0.00

54318
genus

 0.00
0
1
1
3

0
1
2
1
909700
subgenus

 0.00

species

 0.00
417921
1
1

1489943
suborder

 0.01
0
8
1
52

30871
family

 0.01
0
52
1
7

274794

 0.01
subfamily
0
52
1
6

1505891
tribe

 0.01
0
52
5
1

1
11
2
1
94231
genus

 0.00


 0.00
species
293821
1
10

2
1
41
0
genus

 0.01
134629

160734
species

 0.01
1
41

0
15
7
1
8205

 0.00
suborder

3
3
1
0

 0.00
family
36203

56715

 0.00
genus
0
1
2
3

1
3
56716
species

 0.00

30806

 0.00
family
0
1
3
12

52238

 0.00
genus
0
2
1
12

species

 0.00
52239
1
12

clade

 0.00
1489885
1
8
34
0

0
34
7
1
1489894

 0.00
order

0
1
6
34
8224

 0.00
family

subfamily

 0.00
186745
34
1
5
0


 0.00
tribe
186749
1
4
34
0

34
3
1
27
genus

 0.00
8234

4
1

 0.00
species
8236

1
3
8240
species

 0.00

1489875

 0.00
clade
0
7
7
1

1489876
order

 0.00
0
6
1
7

1489877

 0.00
suborder
0
1
5
7

0
4
1
7
83881
family

 0.00

475176
subfamily

 0.00
0
7
1
3

2
1
7
0

 0.00
genus
375763

7
1
375764

 0.00
species

33
1
39
0

 0.01
clade
1489904

21
1
15
0
order

 0.00
8252

21
14
1
0
suborder

 0.00
30942


 0.00
family
8256
5
3
1
0

5
1
2
0

 0.00
genus
8266

5
1

 0.00
species
195615

3
1
8
0

 0.00
family
30948

genus

 0.00
28828
8
2
1
0


 0.00
species
28829
1
8

52902

 0.00
family
0
5
1
3

5
1
2
0
genus

 0.00
52903

52904
species

 0.00
5
1

0
3
4
1
30947
family

 0.00

603456

 0.00
subfamily
0
3
1
3

genus

 0.00
106173
2
1
3
0


 0.00
species
244447
1
3


 0.00
order
1489907
1
10
15
0

family

 0.00
173245
1
3
3
0

0
1
2
3
173246
genus

 0.00

173247

 0.00
species
3
1

12
1
6
0

 0.00
family
8157

8160

 0.00
genus
0
1
3
1

302047

 0.00
species
0
1
2
1


 0.00
subspecies
1841481
1
1


 0.00
genus
36211
1
2
11
0

species

 0.00
36212
1
11

0
3
7
1
1489905

 0.00
no rank

8184

 0.00
family
0
1
3
1

1
2
1
0
genus

 0.00
8186

8187

 0.00
species
1
1

0
3
1
2
30876
family

 0.00

270536

 0.00
genus
0
2
1
2

2
1
941984
species

 0.00

0
6
1
7
1489874

 0.00
clade

8064

 0.00
order
0
7
5
1


 0.00
family
8065
7
4
1
0

subfamily

 0.00
390319
7
3
1
0


 0.00
genus
289381
7
2
1
0

1
7

 0.00
species
390379

0
1
7
1
1489883

 0.00
clade

1
6
1
0

 0.00
order
129912


 0.00
suborder
1489884
1
5
1
0

family

 0.00
72045
1
1
4
0

1
3
1
0
subfamily

 0.00
129914

103719
genus

 0.00
0
2
1
1

1
1

 0.00
species
161584

11
1
15
0
clade

 0.00
1489892

3
5
1
0
order

 0.00
43697


 0.00
suborder
129920
3
1
4
0

94233

 0.00
family
0
3
3
1

0
3
2
1
94234
genus

 0.00

3
1
205130
species

 0.00

1489900

 0.00
order
0
9
1
8

50370
suborder

 0.00
0
8
1
8


 0.00
family
64142
6
3
1
0

64143
genus

 0.00
0
2
1
6

1
6

 0.00
species
64144

0
2
4
1
270602
family

 0.00

158449

 0.00
subfamily
0
3
1
2

158455

 0.00
genus
0
2
1
2

1
2

 0.00
species
158456

4
1
65
61
1489908
clade

 0.01


 0.00
superorder
1489910
1
13
3
0

0
12
1
3
1489911

 0.00
order

1
11
3
0

 0.00
family
8113


 0.00
clade
319095
1
1
5
0

0
1
1
4
318546

 0.00
subfamily


 0.00
tribe
319069
1
1
3
0

272715
genus

 0.00
0
1
1
2


 0.00
species
34807
1
1

319056
clade

 0.00
0
2
5
1

0
2
1
4
318559

 0.00
subfamily

0
2
3
1
318529

 0.00
tribe

2
2
1
0

 0.00
genus
61816


 0.00
species
63155
1
2

1489920

 0.00
clade
0
15
12
1

order

 0.00
1489921
15
1
11
0

suborder

 0.00
123349
10
5
1
0

63826

 0.00
family
0
10
4
1

557415
subfamily

 0.00
0
10
1
3

0
10
1
2
210581

 0.00
genus

10
1

 0.00
species
441366

1
5
5
0
suborder

 0.00
56717

0
4
1
5
56718

 0.00
family

0
3
1
5
703913

 0.00
subfamily

94311
genus

 0.00
0
5
1
2

1
5
181472
species

 0.00

1489913

 0.00
superorder
0
1
35
32

6
1
11
0

 0.00
order
76071

0
11
5
1
28781

 0.00
suborder

0
11
1
4
47757
family

 0.00

0
11
3
1
8088
subfamily

 0.00

0
11
1
2
8089
genus

 0.00

8090
species

 0.00
11
1

0
7
1
9
8075

 0.00
order

270656
family

 0.00
0
5
1
3

0
5
1
2
300305
genus

 0.00


 0.00
species
300306
5
1

238703
family

 0.00
0
4
3
1

1
2
4
0

 0.00
genus
270533

446457

 0.00
species
4
1

order

 0.00
28738
12
21
1
0

11
1
16
0
suborder

 0.00
8087

family

 0.00
8079
1
7
9
0

0
9
6
1
586240
subfamily

 0.00

1
1

 0.00
genus
8082

8080
genus

 0.00
0
2
1
7

1
7
species

 0.00
8081

0
1
2
1
33527

 0.00
genus

species

 0.00
33528
1
1

28758
family

 0.00
0
1
3
1

208332
genus

 0.00
0
2
1
1

1
1
208333
species

 0.00

5
1
1
0

 0.00
family
8076


 0.00
subfamily
136836
4
1
1
0

3
1
1
0

 0.00
tribe
136838

28741
genus

 0.00
0
1
2
1

1
1
28743
species

 0.00

1
4
1
0

 0.00
suborder
45443

0
1
3
1
28771
family

 0.00

52669
genus

 0.00
0
1
1
2

52670

 0.00
species
1
1

0
7
4
1
1489909

 0.00
no rank

0
7
3
1
205120
family

 0.00

0
7
2
1
210631

 0.00
genus


 0.00
species
210632
7
1

1338366
class

 0.00
0
6
5
1

1
4
6
0

 0.00
order
8288

8289
family

 0.00
0
1
3
6

27686

 0.00
genus
0
6
2
1

6
1
27687

 0.00
species

8287

 8.46
superclass
0
60781
444
1


 8.46
clade
1338369
60781
1
443
1

32523
clade

 8.46
17
442
1
60780

413
1
60755
256

 8.46
clade
32524

11
262
1
59498
40674
class

 8.29

0
5
1
1
9254
clade

 0.00


 0.00
order
9255
1
1
4
0

family

 0.00
9256
1
1
3
0

9257
genus

 0.00
0
2
1
1

9258

 0.00
species
1
1

clade

 8.28
32525
1
256
59486
5


 8.28
clade
9347
1
250
59478
47

9348

 0.00
superorder
0
6
1
1

order

 0.00
948950
1
5
1
0

0
1
4
1
948953
suborder

 0.00

1
1
3
0

 0.00
family
227508

1
2
1
0
genus

 0.00
9357

1
1
27675
species

 0.00

448
59429
237
1
1437010
clade

 8.28

314145

 0.32
superorder
107
1
103
2281

order

 0.00
9397
15
10
1
0

30560
suborder

 0.00
1
9
1
15

1
4
1
0
family

 0.00
58055


 0.00
subfamily
186995
1
1
3
0

genus

 0.00
49442
2
1
1
0

89399
species

 0.00
1
1

family

 0.00
9431
12
1
3
0

genus

 0.00
27671
1
2
12
0

1
12
59474

 0.00
species

family

 0.00
9415
1
1

91561
order

 0.02
5
38
1
130

suborder

 0.00
9834
4
1
3
0

family

 0.00
9835
3
3
1
1

1
2
2
0
genus

 0.00
9836

2
1
419612
species

 0.00

0
13
1
10
2653789
suborder

 0.00

9721

 0.00
infraorder
0
13
9
1

1
4
6
0
parvorder

 0.00
9722

family

 0.00
9726
3
1
6
0

9732

 0.00
genus
0
6
2
1

9733

 0.00
species
6
1

0
7
1
4
9761

 0.00
parvorder

0
7
1
3
9765

 0.00
family

genus

 0.00
9766
2
1
7
0

9771

 0.00
species
7
1

0
1
18
95
9845
suborder

 0.01

95
1
17
17
infraorder

 0.01
35500

family

 0.01
9895
59
1
12
5

0
9
7
1
9963

 0.00
subfamily

9935

 0.00
genus
1
8
4
1

9940

 0.00
species
1
2

5
2
1
0

 0.00
species
37174

112262
subspecies

 0.00
1
5

0
1
1
2
9922

 0.00
genus

9925

 0.00
species
1
1

subfamily

 0.01
27592
4
1
45
2

9903
genus

 0.01
35
3
1
43

1
5

 0.00
species
72004

3
1
species

 0.00
9913

family

 0.00
9850
19
1
4
0

3
1
19
0

 0.00
subfamily
34878

9859

 0.00
genus
0
1
2
19

species

 0.00
9860
1
19

suborder

 0.00
35497
14
5
1
0

9821

 0.00
family
1
4
1
14

0
13
3
1
9822

 0.00
genus

9823
species

 0.00
4
1
2
13

1
9
subspecies

 0.00
415978

172
1
31
3

 0.02
order
33554

suborder

 0.02
379584
19
1
112
42

family

 0.00
9655
13
1
12
2


 0.00
subfamily
2892069
4
1
1
0

48419

 0.00
genus
0
1
3
1

0
1
2
1
48420

 0.00
species

subspecies

 0.00
2888765
1
1

169417

 0.00
subfamily
0
2
5
1

1
2
1
0
genus

 0.00
9656

9657
species

 0.00
1
1

2
1
1
0
genus

 0.00
71112

1
1
76717

 0.00
species

0
1
3
7
1008252
subfamily

 0.00

0
7
2
1
9661

 0.00
genus

1
7

 0.00
species
9662

9709
family

 0.00
1
1

0
57
1
4
9608
family

 0.01

genus

 0.01
9611
1
3
57
0

53
1
2
57
9612

 0.01
species

subspecies

 0.00
9615
1
4

57
11
1
1

 0.01
suborder
379583

1
54
1
4
9681

 0.01
family

subfamily

 0.01
338152
3
1
53
0

1
2
53
0
genus

 0.01
9682

53
1
species

 0.01
9685

family

 0.00
9676
1
3
1
0

0
2
1
1
95911

 0.00
genus

species

 0.00
95912
1
1

9697

 0.00
family
0
1
3
1

0
1
2
1
37031
genus

 0.00

37032

 0.00
species
1
1

3
5
1
0
order

 0.00
9787

0
3
1
4
9788

 0.00
family

3
3
1
0
genus

 0.00
9789

1
1

 0.00
species
9796

89248

 0.00
species
1
2

9362
order

 0.26
0
18
1
1854

9376

 0.00
family
0
1
6
2

1
2
1
0

 0.00
subfamily
183662

36801
genus

 0.00
1
1

0
1
1
3
183663

 0.00
subfamily

0
1
1
2
9379

 0.00
genus

1
1
42254

 0.00
species

family

 0.26
9363
1
11
1852
0

1852
10
1
379

 0.26
subfamily
30577

9367

 0.16
genus
0
1
2
1125

9368

 0.16
species
1125
1

343
1
2
0

 0.05
genus
9364

1
343
9365

 0.05
species

1
3
1
0

 0.00
genus
111451

977876

 0.00
species
0
1
1
2

1
1
1756611
subspecies

 0.00

30579

 0.00
genus
0
4
2
1

1
4
species

 0.00
262775

1
133
56700
152
superorder

 7.90
314146

order

 4.54
9443
47
1
32628
5


 0.00
suborder
376911
4
1
8
0

376915

 0.00
infraorder
0
4
1
7

1
3
1
0

 0.00
family
30615

1
2
1
0
genus

 0.00
13149

1
1
species

 0.00
30608

3
1
3
0

 0.00
family
9445

9446
genus

 0.00
0
2
1
3

9447
species

 0.00
3
1

376913
suborder

 4.54
1
32619
38
1

314293
infraorder

 4.54
34
32618
37
1

129
32582
30
1
9526

 4.54
parvorder

51
32439
1
18
314295
superfamily

 4.52

family

 4.51
9604
1
14
32387
135

32240
9
1
1957
subfamily

 4.49
207598

0
1
2
30211
9605
genus

 4.21

9606

 4.21
species
1
30211


 0.00
genus
9592
5
1
3
0

1
5
2
1
9593

 0.00
species

4
1
subspecies

 0.00
9595

9596

 0.01
genus
2
67
1
3


 0.00
species
9597
1
2

1
63

 0.01
species
9598


 0.00
subfamily
607660
4
1
12
0

0
12
3
1
9599
genus

 0.00

species

 0.00
9600
1
1


 0.00
species
9601
1
11

0
1
1
3
9577

 0.00
family

1
2
1
0

 0.00
genus
325165

1
1
61853
species

 0.00

314294

 0.00
superfamily
0
11
1
14

1
10
14
0

 0.00
family
9527

12
1
8
0

 0.00
subfamily
9528

0
2
1
3
9554

 0.00
genus

3
1
species

 0.00
9555

genus

 0.00
9564
1
2
1
0

9565
species

 0.00
1
1

0
8
3
1
9539
genus

 0.00

9544

 0.00
species
1
6

1
2
9541
species

 0.00


 0.00
subfamily
9569
2
1

9479
parvorder

 0.00
0
2
6
1

0
1
3
1
376918

 0.00
family

1
2
1
0

 0.00
genus
9504

37293

 0.00
species
1
1

9498

 0.00
family
0
1
2
1

1
1
38070
subfamily

 0.00

314147
clade

 3.33
3
23919
81
1

order

 3.33
9989
23916
1
80
124

6
52
1
23754
1963758
suborder

 3.31

51
1
23748
906

 3.31
clade
337687

722
22698
1
34
10066

 3.16
family


 0.02
subfamily
39107
1
22
147
21

8
1
4
1

 0.00
genus
10114

species

 0.00
10117
1
1


 0.00
species
10116
5
1

1
1

 0.00
species
35732

0
1
2
1
61153
genus

 0.00

1
1

 0.00
species
61156

1
2
1
0

 0.00
genus
121588

491861
species

 0.00
1
1

0
1
2
1
73108
genus

 0.00

1
1
species

 0.00
742503


 0.00
genus
10128
3
1
8
0

2
1
8
0
species group

 0.00
400053

8
1

 0.00
species
10129


 0.01
genus
10088
107
8
1
1

16
5
1
105
862507
subgenus

 0.01


 0.01
species
10090
2
1
84
83

subspecies

 0.00
10091
1
1

4
1
10096

 0.00
species

1
1
species

 0.00
10089

862508

 0.00
subgenus
0
2
1
1

10093
species

 0.00
1
1

326408

 3.04
subfamily
0
21826
8
1

3220
7
1
21826
10067

 3.04
genus

83762

 0.19
species
1381
1

8
1
10068

 0.00
species

species

 1.89
60746
1
13606

1
3489
60744

 0.49
species

1
6

 0.00
species
92878

83527
species

 0.02
1
116

10045
subfamily

 0.00
0
3
3
1


 0.00
genus
10046
3
1
2
0


 0.00
species
10047
1
3

family

 0.00
337664
4
1
1
0

10061
subfamily

 0.00
0
1
3
1

2
1
1
0
genus

 0.00
30636


 0.00
species
1026970
1
1

family

 0.02
337677
12
1
143
8

5
1
5
1

 0.00
subfamily
39087

1
1
2
0
genus

 0.00
10053

1
1

 0.00
species
100897

10049
genus

 0.00
0
2
1
3

1047088

 0.00
species
3
1

337963
subfamily

 0.02
0
120
3
1

2
1
120
0
genus

 0.02
38667

1
120
38674

 0.02
species

1
3
10
1

 0.00
subfamily
10026

9
2
1
0

 0.00
genus
10028

1
9
10029

 0.00
species

0
20
1
4
33550
suborder

 0.00

10167
family

 0.00
0
20
1
3


 0.00
genus
10180
20
1
2
0

species

 0.00
10181
1
20

10
1
11
0
suborder

 0.00
1963757

family

 0.00
29132
3
1
1
0


 0.00
genus
10184
1
2
1
0


 0.00
species
51338
1
1

7
1
9
0
family

 0.00
10015

38663
subfamily

 0.00
0
4
1
1

0
1
3
1
37442
genus

 0.00

1
2
1
0
species

 0.00
38669

214514
subspecies

 0.00
1
1

8
2
1
0

 0.00
subfamily
38662


 0.00
genus
10016
1
8

8
12
1
0

 0.00
suborder
33553

8
11
1
0

 0.00
family
55153

0
4
1
5
337726
subfamily

 0.00

4
1
4
1

 0.00
tribe
337730

9992
genus

 0.00
0
3
1
3

species

 0.00
9993
3
2
1
0

3
1

 0.00
subspecies
9994

subfamily

 0.00
9991
4
5
1
0


 0.00
tribe
337752
1
4
4
0

1
4
1
3
10001
genus

 0.00

1
1
species

 0.00
55149

1
2
species

 0.00
30640

order

 0.00
9392
1
4
1
0

family

 0.00
9393
3
1
1
0

1
1
2
0
genus

 0.00
9394

1
1
37347
species

 0.00

0
1
6
1
311790

 0.00
superorder


 0.00
order
9774
1
5
1
0

family

 0.00
9775
4
1
1
0

genus

 0.00
9776
1
1
3
0

species

 0.00
9778
1
1
2
0

127582
subspecies

 0.00
1
1

0
5
1
3
9263

 0.00
clade

38609
order

 0.00
0
3
1
4

0
1
3
3
38624

 0.00
family

38625
genus

 0.00
0
3
1
2

38626
species

 0.00
3
1

1001
1
150
0

 0.14
clade
8457

clade

 0.14
32561
1001
149
1
1

0
113
1
989
1329799

 0.14
clade

2841271

 0.00
subclass
0
1
9
2


 0.00
order
8459
2
1
8
0

0
2
7
1
8464
suborder

 0.00

1579337
clade

 0.00
1
2
1
6

5
1
1
0

 0.00
clade
1579336

27791
superfamily

 0.00
0
1
1
4

0
1
3
1
27792
family

 0.00

27793
genus

 0.00
0
1
1
2

1
1
27794

 0.00
species

987
103
1
0
clade

 0.14
8492

0
1
102
987
436486

 0.14
clade

clade

 0.14
436489
101
1
987
0


 0.14
clade
436491
100
1
987
0

1
99
987
0
clade

 0.14
436492

5
987
98
1
8782

 0.14
class

8825

 0.13
infraclass
17
966
1
83

order

 0.00
9219
4
1
1
0

1
3
1
0

 0.00
family
9220

0
1
2
1
56078
genus

 0.00

1
1
56079
species

 0.00

0
1
4
1
56308
order

 0.00

0
1
1
3
56309
family

 0.00

1
2
1
0

 0.00
genus
57396

species

 0.00
57397
1
1

0
3
6
1
2607030
clade

 0.00

1
5
3
0
order

 0.00
8902

48283
family

 0.00
0
3
4
1

48286

 0.00
subfamily
0
3
1
3

0
3
2
1
48284

 0.00
genus

3
1
111811

 0.00
species

0
4
1
381
8929

 0.05
order

8930
family

 0.05
0
3
1
381

36242
genus

 0.05
0
381
2
1

1
381
177155
species

 0.05

5
20
1
23
9126
order

 0.00

175121
superfamily

 0.00
0
1
1
5


 0.00
family
37611
4
1
1
0

3
1
1
0

 0.00
subfamily
40155

1
2
1
0
genus

 0.00
59728

1
1
species

 0.00
59729

0
7
1
3
9183
family

 0.00

0
7
2
1
36283

 0.00
genus

species

 0.00
37610
7
1

9153
family

 0.00
0
1
3
1

genus

 0.00
156562
1
2
1
0

1
1
156563

 0.00
species

114313
family

 0.00
1
3
5
1

196026
genus

 0.00
0
1
1
2


 0.00
species
296741
1
1

114328
genus

 0.00
0
1
1
2

1
1
species

 0.00
114329

6
1
3
0
superfamily

 0.00
2116661

0
5
1
3
36270

 0.00
family

3
1
4
0
subfamily

 0.00
330750

0
3
1
3
39620
genus

 0.00

species

 0.00
48156
1
2
3
0

3
1
subspecies

 0.00
126889

0
8
1
8
2558200

 0.00
order

7
1
8
0

 0.00
family
56259

8
1
6
5
subfamily

 0.00
8955

8960

 0.00
genus
0
1
3
1

species

 0.00
8962
1
1
2
0

1
1
223781
subspecies

 0.00


 0.00
genus
8956
2
1
2
0

species

 0.00
8957
1
2

superorder

 0.07
1549675
26
1
533
0

8826

 0.00
order
0
8
1
4

8830
family

 0.00
0
7
1
4

0
2
1
3
2068716

 0.00
subfamily

2
2
1
0
genus

 0.00
8835


 0.00
species
8839
1
2

2068722

 0.00
subfamily
0
2
1
3

8842
genus

 0.00
0
2
1
2

8845

 0.00
species
1
2

order

 0.07
8976
529
17
1
2

1
3
1
0

 0.00
family
8990

0
1
2
1
8995
genus

 0.00

1
1
8996

 0.00
species

11
1
13
526
9005

 0.07
family

471
3
1
0
subfamily

 0.07
9072

1
2
471
1

 0.07
genus
9030

9031

 0.07
species
1
470

466585
subfamily

 0.00
1
3
3
1

1
2
2
0
genus

 0.00
30409


 0.00
species
64668
1
2

34
3
1
0
subfamily

 0.00
466552

1
2
34
0
genus

 0.00
9102


 0.00
species
9103
34
1

0
7
1
3
466544
subfamily

 0.00

7
2
1
0

 0.00
genus
9090


 0.00
species
93934
1
7

0
1
3
1
8948
order

 0.00

0
1
2
1
8949
family

 0.00

8952
genus

 0.00
1
1

0
1
1
4
9230
order

 0.00

3
1
1
0

 0.00
family
9231

genus

 0.00
9232
1
2
1
0

1
1
9233
species

 0.00

superorder

 0.00
8783
16
1
14
0

8802
order

 0.00
0
1
4
1


 0.00
family
8803
1
1
3
0

0
1
2
1
8806
genus

 0.00

species

 0.00
30464
1
1

0
14
1
5
8819
order

 0.00

family

 0.00
8820
4
1
14
0

14
3
1
1
genus

 0.00
8821

0
13
1
2
2696672

 0.00
species

1
13
202946
subspecies

 0.00

0
1
4
1
8784
order

 0.00

3
1
1
0

 0.00
family
8788

0
1
1
2
8789
genus

 0.00

species

 0.00
8790
1
1

0
11
1
35
8504
class

 0.00

0
1
34
11
8509

 0.00
order


 0.00
clade
1329961
11
33
1
0


 0.00
clade
1329950
32
1
11
0

0
11
1
31
1329912

 0.00
clade

clade

 0.00
1329976
5
6
1
0

5
5
1
0

 0.00
clade
1329975

5
1
4
0
family

 0.00
8522

subfamily

 0.00
162266
5
3
1
0


 0.00
genus
42163
1
2
5
0

64176

 0.00
species
5
1

0
6
24
1
1329911
clade

 0.00

infraorder

 0.00
8570
5
1
17
0

superfamily

 0.00
34989
4
1
12
0

8578

 0.00
family
0
1
4
1

1
3
1
0

 0.00
subfamily
169862

1
1
2
0
genus

 0.00
34999

1
1
35005

 0.00
species


 0.00
family
8689
7
1
3
0

8690

 0.00
subfamily
0
1
3
2

2
2
1
0
genus

 0.00
8703

103942

 0.00
species
1
2

8710
subfamily

 0.00
0
1
1
3

0
1
2
1
8728
genus

 0.00

1
1
88082
species

 0.00

superfamily

 0.00
34979
1
4
1
0

0
3
1
1
34984

 0.00
family

37579

 0.00
genus
0
1
2
1


 0.00
species
176946
1
1

0
1
6
1
8548

 0.00
infraorder

5
1
1
0

 0.00
clade
1330544

superfamily

 0.00
1329920
1
4
1
0

8555
family

 0.00
0
1
3
1

genus

 0.00
8556
2
1
1
0

61221
species

 0.00
1
1

0
1
28
8
8292
class

 0.00


 0.00
order
8445
1
10
5
0

264006

 0.00
family
0
1
3
2

0
1
2
2
260994
genus

 0.00

260995
species

 0.00
1
2


 0.00
family
1277737
3
1
1
0

2
1
1
0
genus

 0.00
264009

1
1
1415580

 0.00
species

family

 0.00
30380
1
3
2
0

2
1
2
0

 0.00
genus
194407

species

 0.00
194408
2
1

41666

 0.00
superorder
0
3
17
1

1
16
3
0
order

 0.00
8342

0
1
5
1
8416
suborder

 0.00

4
1
1
0

 0.00
superfamily
8417

1
3
1
0
family

 0.00
192735

0
1
2
1
8419

 0.00
genus

248795
species

 0.00
1
1

4
1
1
0

 0.00
superfamily
8431

0
1
1
3
8432

 0.00
family

0
1
2
1
61615

 0.00
genus

1
1

 0.00
species
61616

0
6
1
1
30319

 0.00
superfamily

1
5
1
0

 0.00
family
8352

subfamily

 0.00
8360
1
4
1
0

0
1
3
1
8353
genus

 0.00


 0.00
subgenus
8363
1
2
1
0

species

 0.00
8364
1
1

1476529

 0.00
clade
0
1
1
6

1
5
1
0

 0.00
class
117569

4
1
1
0
order

 0.00
7745

1
3
1
0
family

 0.00
7746

7756

 0.00
genus
0
1
1
2

1
1
7757
species

 0.00

1
1551
9630
1627
clade

 1.34
33317

0
211
1
480
2697495

 0.07
clade


 0.07
clade
1206795
1
202
479
28

6340
phylum

 0.00
0
19
1
28

class

 0.00
42113
1
15
5
0

0
1
1
6
55824
subclass

 0.00

2218736
order

 0.00
0
1
5
1

0
4
1
1
2218739

 0.00
suborder

60930

 0.00
family
0
3
1
1

60957
genus

 0.00
0
2
1
1

60958

 0.00
species
1
1

6381
subclass

 0.00
0
4
1
8

2803884

 0.00
order
0
4
1
7

suborder

 0.00
6391
4
6
1
0


 0.00
family
6392
1
5
4
0

subfamily

 0.00
1046325
4
1
4
0

genus

 0.00
6397
4
3
1
0

4
2
1
0
no rank

 0.00
1050932

4
1
35632

 0.00
species

6341
class

 0.00
0
12
1
14

0
14
1
11
105390
subclass

 0.00

6348
order

 0.00
0
14
1
10

46593
family

 0.00
0
9
1
3

9
2
1
0

 0.00
genus
222002

species

 0.00
1210411
1
9

1
3
2
0
family

 0.00
104728

55701
genus

 0.00
0
1
2
2

species

 0.00
1210413
1
2

0
1
3
3
39820

 0.00
family

0
1
2
3
868094

 0.00
genus

3
1
880429

 0.00
species

0
1
6
2
6217

 0.00
phylum

6218

 0.00
class
0
2
1
5


 0.00
order
6219
2
4
1
0

0
2
3
1
6222

 0.00
family

0
2
2
1
6223
genus

 0.00

1
2
88925

 0.00
species

37
1
57
1

 0.01
phylum
6157

0
26
1
48
6178

 0.01
class

6179
subclass

 0.01
0
48
25
1

27871

 0.00
order
0
3
6
1

27872

 0.00
suborder
0
5
1
3

0
3
4
1
1776223

 0.00
superfamily

73421
family

 0.00
0
3
3
1

genus

 0.00
57077
2
1
3
0

57078

 0.00
species
1
3

0
45
1
18
6180
order

 0.01

31244

 0.01
superfamily
0
45
17
1

family

 0.01
31245
16
1
45
0

genus

 0.00
39198
1
2
5
0

39320

 0.00
species
1
5

100601

 0.00
genus
0
2
1
2

1
2
species

 0.00
157069

6
38
11
1
6181

 0.01
genus

2
1
1163369
species

 0.00

1
5
6189
species

 0.00

2
1
6184

 0.00
species

2
1
6182
species

 0.00


 0.00
species
6187
1
1

31246

 0.00
species
7
1


 0.00
species
6185
1
4

48269

 0.00
species
2
1

6188

 0.00
species
1
6

species

 0.00
6183
1
1


 0.00
class
6199
10
1
8
0


 0.00
subclass
6200
9
1
8
0

order

 0.00
6201
1
4
1
0

0
1
1
3
6214
family

 0.00

6215
genus

 0.00
0
1
1
2

1
1
85433

 0.00
species


 0.00
order
1224679
7
1
4
0

7
1
3
0
family

 0.00
28843

7
1
2
0
genus

 0.00
46580

species

 0.00
99802
1
7

10205
phylum

 0.00
0
5
1
17

class

 0.00
10206
16
1
5
0

0
1
15
5
10207
order

 0.00

0
1
5
1
193205

 0.00
suborder

4
1
1
0
superfamily

 0.00
193206

0
3
1
1
192924
family

 0.00

1
1
2
0
genus

 0.00
95169

1
1

 0.00
species
95170

558764

 0.00
suborder
0
9
1
4

193246

 0.00
superfamily
0
1
4
3

558762

 0.00
family
0
3
1
3

0
3
1
2
558754
genus

 0.00

1
3
558755
species

 0.00

193209

 0.00
superfamily
0
1
4
1

3
1
1
0

 0.00
family
10210

1970207
genus

 0.00
0
1
2
1

1
1
192920

 0.00
species

0
8
1
1
7568

 0.00
phylum

subphylum

 0.00
115360
7
1
1
0

1
1
6
0

 0.00
class
115361

1
5
1
0

 0.00
order
7570

115362

 0.00
superfamily
0
4
1
1

33491

 0.00
family
0
1
1
3

0
1
2
1
7571
genus

 0.00

species

 0.00
7574
1
1


 0.05
phylum
6447
1
105
367
7

1
9
4
0

 0.00
class
6605

6606

 0.00
subclass
0
4
8
1

0
4
1
7
215451
superorder

 0.00


 0.00
order
6638
4
6
1
0

suborder

 0.00
6646
5
1
4
0

family

 0.00
6647
1
4
4
0

6643
genus

 0.00
0
4
3
1

2607531
species

 0.00
1
3

37653
species

 0.00
1
1

6448
class

 0.01
1
63
1
46

subclass

 0.00
216305
14
1
9
0

clade

 0.00
216307
9
13
1
0

clade

 0.00
977775
1
6
7
0

977779

 0.00
clade
0
5
1
7

216441
superfamily

 0.00
0
7
1
4

0
1
3
7
6524

 0.00
family


 0.00
genus
6525
7
1
2
0

6526

 0.00
species
1
7


 0.00
clade
2836391
2
1
6
0

6497
order

 0.00
0
5
1
2

superfamily

 0.00
216318
1
4
2
0

0
2
3
1
6498

 0.00
family

6499

 0.00
genus
0
2
2
1

species

 0.00
6500
2
1

0
3
1
5
2219556

 0.00
subclass

4
1
3
0
superfamily

 0.00
216274

3
3
1
0

 0.00
family
55007

3
2
1
0

 0.00
genus
1735270

species

 0.00
1735272
3
1


 0.00
subclass
69675
1
10
10
0

0
5
1
7
146277

 0.00
superfamily


 0.00
family
6462
1
4
7
0

3
1
7
1
genus

 0.00
6463

species

 0.00
6465
1
3

1
3
88005

 0.00
species

1
4
3
0
superfamily

 0.00
216260

family

 0.00
69676
3
1
3
0


 0.00
genus
72691
2
1
3
0


 0.00
species
225164
3
1

216275

 0.01
subclass
0
40
16
1

10
1
39
0

 0.01
order
2315720

0
1
9
39
216285

 0.01
superfamily

family

 0.01
6466
1
8
39
0

1955429
subfamily

 0.01
1
1
7
39

2072689
genus

 0.00
0
2
1
15

15
1
216125

 0.00
species

2
1
11
0
genus

 0.00
1093071

1620919
species

 0.00
11
1

0
12
1
2
148341

 0.00
genus

1
12
703304

 0.00
species


 0.00
order
2315723
5
1
1
0

216276
superfamily

 0.00
0
1
4
1

1
3
1
0

 0.00
family
6451

0
1
1
2
6452
genus

 0.00

1
1

 0.00
species
6454

293
1
49
0

 0.04
class
6544

subclass

 0.04
2785011
293
1
48
8

32
19
1
0

 0.00
clade
6545

order

 0.00
6546
1
6
6
0

6
5
1
0
superfamily

 0.00
106220


 0.00
family
6547
6
1
4
0

6
1
3
0

 0.00
subfamily
2899742

356392
genus

 0.00
0
6
1
2

6
1
356393
species

 0.00

10
5
1
0

 0.00
order
106218


 0.00
superfamily
106219
4
1
10
0

0
10
3
1
6566

 0.00
family

6578

 0.00
genus
0
10
2
1

1
10

 0.00
species
6579

1
7
16
0
order

 0.00
6562


 0.00
superfamily
98302
16
6
1
0

family

 0.00
6563
16
1
5
0

genus

 0.00
37858
1
1
2
0

1
1

 0.00
species
37623

genus

 0.00
6564
15
2
1
0

species

 0.00
29159
1
15

0
253
28
1
6599

 0.04
clade

infraclass

 0.04
735337
27
1
253
0


 0.04
superorder
2785015
253
26
1
0

1
25
253
3

 0.04
clade
2908833


 0.00
order
6580
8
5
1
0

8
4
1
0
superfamily

 0.00
106231

family

 0.00
6581
1
3
8
0

0
8
1
2
6582

 0.00
genus

31201
species

 0.00
8
1

0
1
19
242
2783445
order

 0.03

0
220
1
11
105710

 0.03
superfamily


 0.03
family
55708
1
10
220
0

1
6
211
1
subfamily

 0.03
2787994

0
6
1
2
80817
genus

 0.00

6
1

 0.00
species
80818

204
3
1
0
genus

 0.03
52939

1
6
80829

 0.00
species

198
1
species

 0.03
80833

3
1
9
0

 0.00
subfamily
2787989

1
2
9
0

 0.00
genus
80820

80821
species

 0.00
1
9

superfamily

 0.00
98297
22
7
1
0

0
3
1
20
61354
family

 0.00

0
2
1
20
457750

 0.00
genus

20
1
2589376

 0.00
species

2
3
1
0

 0.00
family
121179


 0.00
no rank
1969323
2
2
1
0

2
1
species

 0.00
1969324

2697496
clade

 0.00
0
1
8
1

1
1
7
0

 0.00
phylum
10190

0
1
1
6
2816136

 0.00
class

0
1
5
1
44578

 0.00
subclass

104779

 0.00
order
0
1
4
1

104780

 0.00
family
0
1
3
1


 0.00
genus
104781
1
1
2
0

1
1
species

 0.00
104782

1206794

 1.05
clade
168
1339
1
7523

58
40
1
0

 0.01
phylum
6231

class

 0.01
119089
58
39
1
0

6308
order

 0.00
0
4
1
10

superfamily

 0.00
2572558
5
1
1
0

33278

 0.00
family
0
4
1
1


 0.00
subfamily
53477
1
3
1
0

51030
genus

 0.00
0
2
1
1

1
1
species

 0.00
51031

0
1
4
3
6314

 0.00
superfamily

0
1
3
3
126387

 0.00
family


 0.00
genus
6288
3
1
2
0

1
3
species

 0.00
6289

0
28
1
54
6236

 0.01
order

0
1
12
10
6300

 0.00
suborder


 0.00
infraorder
2082223
1
11
10
0

superfamily

 0.00
2082224
10
1
10
0

1
4
1
0

 0.00
family
114888

0
1
3
1
114889
genus

 0.00

2629767

 0.00
no rank
0
1
2
1

1
1
species

 0.00
114890

9
5
1
0
family

 0.00
6246

6247

 0.00
genus
0
2
1
7

34506

 0.00
species
1
7

131309

 0.00
genus
0
2
1
2

131310

 0.00
species
1
2

suborder

 0.01
2301116
44
1
15
0

2301119

 0.01
infraorder
0
1
14
44

55879
superfamily

 0.01
0
1
13
44

0
1
12
44
6243

 0.01
family

0
4
1
9
55885
subfamily

 0.00

6237
genus

 0.00
0
3
1
9

1
8
species

 0.00
6239

1
1

 0.00
species
6238

0
7
1
35
55887

 0.00
subfamily

35
1
6
1

 0.00
genus
42476

no rank

 0.00
2613844
2
3
1
0

species

 0.00
2879420
1
1

1
1
2879419
species

 0.00


 0.00
species
473156
1
1

31
1
141969
species

 0.00

88770
clade

 1.02
0
7297
1
1298

7297
1
1297
248

 1.02
phylum
6656

clade

 0.97
197563
6990
1234
1
5

clade

 0.97
197562
1233
1
6985
371

1
1183
6560
29

 0.91
subphylum
6960


 0.00
class
30001
1
5
4
0

79705

 0.00
order
0
1
4
4

4
3
1
0
family

 0.00
39131


 0.00
genus
39132
1
2
4
0

species

 0.00
39272
1
4

0
6527
1
1177
50557

 0.91
class

85512

 0.91
clade
0
6527
1176
1

1
1175
6527
17

 0.91
subclass
7496

infraclass

 0.00
33339
2
7
1
0


 0.00
order
6961
2
1
6
0

0
2
5
1
50488

 0.00
suborder

0
4
1
2
70894
superfamily

 0.00

0
2
1
3
70895

 0.00
family

0
2
2
1
79456
genus

 0.00

197161

 0.00
species
1
2

1
1167
6508
840

 0.91
infraclass
33340

1
55
75
5
cohort

 0.01
33341

0
8
1
19
50622

 0.00
order

5
13
1
0

 0.00
superfamily
70405


 0.00
family
143733
1
4
1
0

0
1
1
3
143769
subfamily

 0.00

0
1
1
2
143734

 0.00
genus

143735
species

 0.00
1
1

0
3
4
1
143727

 0.00
family

0
1
3
3
143771

 0.00
subfamily

143731
genus

 0.00
0
3
1
2

1
3

 0.00
species
2065413

family

 0.00
62802
1
4
1
0

3
1
1
0

 0.00
subfamily
143773

143721
genus

 0.00
0
1
2
1

1
1
species

 0.00
143722


 0.00
superfamily
70404
1
5
3
0

0
4
1
3
50623

 0.00
family

1
3
3
0

 0.00
subfamily
466866

0
3
2
1
143767
genus

 0.00

1
3

 0.00
species
552050

7020

 0.01
order
0
59
14
1

13
1
59
0
suborder

 0.01
523712

0
59
1
12
213545

 0.01
superfamily

213546
family

 0.01
0
11
1
59

61471
genus

 0.01
36
59
10
1

629360

 0.00
species
1
1

3
1
species

 0.00
629358

1
4
species

 0.00
61476

species

 0.00
61478
1
1

1
1
61484
species

 0.00

170557
species

 0.00
1
3

170555
species

 0.00
5
1

61472
species

 0.00
1
3

1
2
61474

 0.00
species

0
1
13
2
6970

 0.00
superorder

85823

 0.00
order
0
12
1
2

0
2
11
1
1049657
superfamily

 0.00

0
2
1
10
1912919
no rank

 0.00

5
1
1
0

 0.00
family
7501

0
1
4
1
127820

 0.00
subfamily

127821

 0.00
tribe
0
1
3
1

0
2
1
1
7502
genus

 0.00

1
1
species

 0.00
136037


 0.00
family
46562
1
4
1
0

105801
subfamily

 0.00
0
3
1
1


 0.00
genus
60568
1
2
1
0

105785

 0.00
species
1
1

0
8
1
1
6993
order

 0.00

0
1
1
7
7001
suborder

 0.00

0
1
6
1
1955150
infraorder

 0.00

70910
no rank

 0.00
0
1
5
1

92621
superfamily

 0.00
0
1
4
1

0
1
1
3
7002

 0.00
family

subfamily

 0.00
37267
1
2
1
0

7008
genus

 0.00
1
1

5579
1
1080
1869
cohort

 0.78
33392


 0.00
superorder
85817
7
1
3
0

0
3
1
6
7516
order

 0.00

suborder

 0.00
2029065
3
5
1
0

family

 0.00
7520
3
1
4
0

subfamily

 0.00
2029106
1
3
3
0

7521

 0.00
genus
0
3
1
2

1
3
189513

 0.00
species

210
1
562
3112
85604
superorder

 0.43

7088

 0.40
order
22
2866
1
548

0
2836
543
1
41191

 0.39
suborder

1
542
2836
0
infraorder

 0.39
41196

40
2836
541
1
41197
parvorder

 0.39

37567
clade

 0.39
692
1
533
2791

37582
superfamily

 0.00
1
22
12
1

family

 0.00
51653
3
1
3
0

3
2
1
0

 0.00
genus
51654

3
1
51655
species

 0.00

14
1
4
0

 0.00
family
687156

0
14
1
3
687147
genus

 0.00

species

 0.00
1870436
1
10

species

 0.00
1870435
4
1

0
1
4
4
33464

 0.00
family

3
1
4
0

 0.00
subfamily
397427

33465
genus

 0.00
0
4
2
1

4
1
263933
species

 0.00

15
119
68
1
104430

 0.02
clade

superfamily

 0.00
104434
31
15
1
0

family

 0.00
106496
31
14
1
0


 0.00
subfamily
106499
13
1
31
0

20
8
1
0

 0.00
tribe
301641

0
1
2
6
300855

 0.00
genus

1
6

 0.00
species
301037

106500
genus

 0.00
0
5
1
14

2
1
species

 0.00
1108569

1
6
species

 0.00
301036

1
4

 0.00
species
1660703

1108570

 0.00
species
2
1

0
4
1
11
301638

 0.00
tribe

3
1
11
1
genus

 0.00
287191

287197
species

 0.00
1
8

1
2
748215

 0.00
species

104437

 0.00
superfamily
0
4
1
2

252293

 0.00
family
0
1
3
2

genus

 0.00
252294
2
2
1
0

1
2
252295

 0.00
species

37568
superfamily

 0.01
0
49
1
35

family

 0.01
7139
34
1
49
2

1
1
24
40
81687
subfamily

 0.01

0
9
8
1
581387
tribe

 0.00

genus

 0.00
581588
1
3
3
0

1101027
species

 0.00
1
1

1
2
species

 0.00
1870148


 0.00
genus
293340
4
1
2
0

192188

 0.00
species
1
4

2
2
1
0
genus

 0.00
82599

1100963
species

 0.00
1
2

581385

 0.00
tribe
0
17
1
7

0
5
2
1
581513
genus

 0.00

1594315
species

 0.00
5
1


 0.00
genus
29054
12
4
1
2

989769
species

 0.00
1
1

1594293

 0.00
species
1
4

1594294
species

 0.00
5
1

581389
tribe

 0.00
0
13
1
8

0
3
1
4
572704
genus

 0.00


 0.00
species
1100916
2
1

1100915
species

 0.00
1
2


 0.00
genus
581658
3
2
1
0


 0.00
species
1100989
3
1

2
1
6
0

 0.00
genus
572852

1
6
species

 0.00
1869985

65022
subfamily

 0.00
0
7
9
1

81689
tribe

 0.00
0
3
1
1


 0.00
genus
192190
1
2
1
0

753214
species

 0.00
1
1

tribe

 0.00
173709
5
1
6
0

572799
genus

 0.00
0
6
4
1

758706
species

 0.00
1
1

2
1
1100899

 0.00
species

758717

 0.00
species
3
1

1
5
6
0

 0.00
superfamily
104432

0
4
1
6
186108

 0.00
family

6
1
3
0
subfamily

 0.00
1556158

6
2
1
0
genus

 0.00
655084

1
6
1101072

 0.00
species

16
1
8
0
superfamily

 0.00
104435

115353

 0.00
family
0
3
1
11


 0.00
genus
287107
11
2
1
0

11
1
species

 0.00
287200

115354
family

 0.00
0
1
4
5

3
1
5
0

 0.00
subfamily
287187

0
5
1
2
287110
genus

 0.00

5
1
species

 0.00
287375


 0.00
superfamily
41011
6
1
10
0

0
5
1
10
41012
family

 0.00

0
10
4
1
236781
subfamily

 0.00

genus

 0.00
41013
10
1
3
0


 0.00
species
1594354
6
1

1101063
species

 0.00
1
4

104431
clade

 0.27
921
429
1
1919

superfamily

 0.00
104423
10
1
11
0

0
1
9
11
104425

 0.00
family


 0.00
subfamily
104465
5
1
8
0

7
2
1
0
genus

 0.00
104426

1
7
104428
species

 0.00

0
1
1
2
721164
genus

 0.00


 0.00
species
721165
1
1

219490
subfamily

 0.00
0
3
1
3

219491
genus

 0.00
0
3
1
2

species

 0.00
721137
3
1


 0.06
superfamily
37570
166
1
417
79


 0.03
family
7100
1
102
223
47

0
9
1
6
95175

 0.00
subfamily

genus

 0.00
938225
1
2
4
0

1
4
species

 0.00
938226


 0.00
genus
56362
5
1
3
0


 0.00
species
987859
1
2


 0.00
species
987865
1
3

1
16
16
0

 0.00
subfamily
572922

0
1
2
3
988063
genus

 0.00

1
3

 0.00
species
1337163

6
1
3
0
genus

 0.00
689302

species

 0.00
987866
3
1

3
1
987872
species

 0.00

1
2
1
0
genus

 0.00
988070

1
1

 0.00
species
988071

2492374
genus

 0.00
0
2
1
1

1
1
species

 0.00
2492375

1
2
1
0

 0.00
genus
988080

species

 0.00
988081
1
1

2
2
1
0

 0.00
genus
997550

1
2
997551
species

 0.00

genus

 0.00
1101105
2
2
1
0

2
1
1101106
species

 0.00

subfamily

 0.00
95214
3
1
3
0

0
1
2
3
753440

 0.00
genus


 0.00
species
753441
1
3

0
20
1
12
95182
subfamily

 0.00

4
1
7
3

 0.00
genus
7106

1
2

 0.00
species
7109


 0.00
species
7108
1
1

1
1

 0.00
species
7107

1
2
2
0

 0.00
genus
1430964

1870430

 0.00
species
1
2

4
2
1
0
genus

 0.00
988055

988056
species

 0.00
1
4

0
1
3
7
56365
genus

 0.00

species

 0.00
689058
4
1


 0.00
species
987877
3
1

116124
subfamily

 0.00
0
8
1
4

3
1
8
0
genus

 0.00
116125

species

 0.00
116126
4
1

4
1

 0.00
species
987909

95178

 0.00
subfamily
0
3
1
1

0
1
2
1
7112

 0.00
genus

1
1
7113

 0.00
species

1
28
1
11
95186

 0.00
subfamily


 0.00
genus
254717
5
2
1
0

species

 0.00
689277
1
5

2
1
5
0
genus

 0.00
179673

1
5

 0.00
species
179674

254364

 0.00
genus
0
1
3
12

254365
species

 0.00
1
3

938171
species

 0.00
9
1


 0.00
genus
254361
5
3
1
0

254363
species

 0.00
2
1

3
1

 0.00
species
987893


 0.01
subfamily
95179
50
26
1
2


 0.00
genus
320076
1
2
2
0

species

 0.00
987895
1
2

tribe

 0.00
2555566
33
15
1
4

11
1
4
0
genus

 0.00
214276

753202
species

 0.00
1
2

1
4

 0.00
species
214277

987995

 0.00
species
5
1

10
4
1
0
genus

 0.00
320016

988049

 0.00
species
1
3

997545
species

 0.00
6
1

987431

 0.00
species
1
1

1
2
1
0
genus

 0.00
214282

1
1

 0.00
species
320037

320033

 0.00
genus
0
3
1
2

987925
species

 0.00
1
3

1
2
4
0

 0.00
genus
47766

4
1
species

 0.00
1857961

2555556

 0.00
tribe
1
8
1
13

688395
genus

 0.00
0
1
1
2

1
1
species

 0.00
987876

genus

 0.00
95189
8
1
3
1

2
1
875885
species

 0.00

1
5
689061

 0.00
species

2
1
3
0

 0.00
genus
882791

988174
species

 0.00
3
1

subfamily

 0.00
1430885
3
1
1
0

genus

 0.00
708062
1
2
1
0

1
1
708063

 0.00
species


 0.01
subfamily
95244
40
17
1
2

946290

 0.00
genus
0
1
2
7

988041

 0.00
species
1
7

0
1
2
1
320089

 0.00
genus

875884
species

 0.00
1
1

988123

 0.00
genus
0
2
1
1

1
1

 0.00
species
988125

55056
genus

 0.00
0
3
2
1

1
3
species

 0.00
55057


 0.00
genus
988059
2
1
8
0

1
8
988060

 0.00
species

10
4
1
1

 0.00
genus
103830

species

 0.00
987983
2
1


 0.00
species
987985
1
5

997540

 0.00
species
2
1

0
2
1
8
320087

 0.00
genus


 0.00
species
987933
1
8

695564

 0.01
family
5
84
37
1

1
16
30
1

 0.00
subfamily
30225

tribe

 0.00
1945703
1
7
15
2

214365
genus

 0.00
0
2
1
3

1
3
875880

 0.00
species

2
1
2
0

 0.00
genus
695183

1
2
species

 0.00
875881

0
2
1
8
214310
genus

 0.00

species

 0.00
214311
1
8

0
1
8
14
132199

 0.00
tribe

694848

 0.00
genus
0
1
2
4

987918
species

 0.00
4
1

genus

 0.00
464722
8
3
1
1

1
3
species

 0.00
987419

987424

 0.00
species
4
1

0
2
1
2
694657

 0.00
genus

1
2
species

 0.00
987980

subfamily

 0.00
95217
5
1
8
0

0
1
2
4
411962

 0.00
genus

1
4
411963
species

 0.00

95218

 0.00
genus
0
2
1
4

4
1
753189

 0.00
species

0
5
1
13
27548

 0.00
subfamily


 0.00
genus
13122
9
2
1
0

1
9
species

 0.00
78897

1
2
4
0
genus

 0.00
319798

species

 0.00
987935
4
1

95222
subfamily

 0.00
0
3
1
7

705937

 0.00
genus
0
7
2
1


 0.00
species
987440
7
1


 0.00
subfamily
1583079
21
1
7
1

938237

 0.00
genus
0
6
2
1

6
1
938238
species

 0.00


 0.00
genus
56375
8
2
1
0


 0.00
species
423510
8
1

0
6
1
2
292570
genus

 0.00

species

 0.00
938167
1
6

319783
family

 0.00
0
8
1
7

753688

 0.00
subfamily
0
3
1
4

2
1
4
0

 0.00
genus
572722

1
4
988004

 0.00
species


 0.00
subfamily
95245
3
1
4
0

1
2
4
0
genus

 0.00
56393

987977
species

 0.00
4
1

family

 0.00
37571
23
19
1
2

subfamily

 0.00
319766
1
3
3
0

2
1
3
0

 0.00
genus
56587

3
1
species

 0.00
987943

6
1
3
0

 0.00
subfamily
319773

genus

 0.00
13633
6
2
1
0

6
1
753216
species

 0.00

1
1
6
6
319765
subfamily

 0.00

0
3
3
1
214308
genus

 0.00

988018

 0.00
species
1
1

2
1
species

 0.00
988019


 0.00
genus
214280
2
1
2
0

753204
species

 0.00
2
1

0
4
3
1
319762
subfamily

 0.00

genus

 0.00
214089
4
2
1
0

4
1
species

 0.00
987902

3
1
2
0

 0.00
subfamily
319770

1
2
2
0
genus

 0.00
987448


 0.00
species
987449
2
1

0
1
19
20
37569
superfamily

 0.00

7089
family

 0.00
0
4
1
4

475327
subfamily

 0.00
0
3
1
4

4
2
1
0
genus

 0.00
7090

4
1
species

 0.00
7091

7128

 0.00
family
1
16
14
1

1
6
7
0

 0.00
subfamily
469321

523180

 0.00
tribe
0
1
5
7

522835
genus

 0.00
0
1
2
3

species

 0.00
522836
1
3

2
1
4
0
genus

 0.00
522847

4
1
522848

 0.00
species

1
7
1
8
82617
subfamily

 0.00

tribe

 0.00
523176
1
3
6
0

0
2
1
6
283833
genus

 0.00

6
1

 0.00
species
644661

0
1
3
1
523174

 0.00
tribe

genus

 0.00
82622
1
2
1
0


 0.00
species
987953
1
1

0
15
1
9
104493

 0.00
superfamily

8
1
15
0

 0.00
family
186111

0
15
7
1
186112
subfamily

 0.00

2
1
7
0

 0.00
genus
1594452

7
1
1594453
species

 0.00

0
6
2
1
467773

 0.00
genus

467774
species

 0.00
6
1


 0.00
genus
753424
1
2
2
0

1
2
1660692
species

 0.00

40092
superfamily

 0.00
0
16
1
21


 0.00
family
40093
21
15
1
0

4
3
1
0

 0.00
subfamily
40100

genus

 0.00
40102
1
2
4
0

1
4
species

 0.00
218760


 0.00
subfamily
218718
7
1
3
0

0
1
2
7
218719

 0.00
genus

species

 0.00
218720
7
1

0
10
8
1
40096
subfamily

 0.00

2839394

 0.00
tribe
0
1
7
10


 0.00
genus
218743
2
1
4
0

4
1
876063

 0.00
species

1
2
4
0
genus

 0.00
218734

1
4
291688

 0.00
species

218770

 0.00
genus
0
2
2
1

1
2
species

 0.00
272628

82592
superfamily

 0.02
0
68
1
138

13
67
1
138
82593
family

 0.02

104442
subfamily

 0.00
0
1
3
4

2
1
4
0

 0.00
genus
104446

104447
species

 0.00
4
1

27
1
66
3
subfamily

 0.01
82596

genus

 0.00
214128
11
1
3
1

species

 0.00
722662
1
7

1
3
875883
species

 0.00

934914
genus

 0.00
0
2
1
10

934915

 0.00
species
10
1

704816
genus

 0.00
0
9
1
2

9
1
934882
species

 0.00

2
1
1
0

 0.00
genus
393392

1
1
688445

 0.00
species


 0.00
genus
572919
1
2
3
0

species

 0.00
934894
3
1


 0.00
genus
692034
3
1
2
0

species

 0.00
934875
3
1

0
2
1
2
104473

 0.00
genus

2
1
104474

 0.00
species


 0.00
genus
190355
2
1
2
0

1
2

 0.00
species
190356

0
6
2
1
82594
genus

 0.00

1
6
82595

 0.00
species


 0.00
genus
104475
8
3
1
0

1
2
190331
species

 0.00

6
1
104476
species

 0.00

82597
genus

 0.00
0
6
1
2

6
1

 0.00
species
934813

0
1
2
2
704699

 0.00
genus

1
2

 0.00
species
934829

0
1
3
3
393382
subfamily

 0.00


 0.00
genus
104485
1
2
3
0

1
3
104486

 0.00
species

subfamily

 0.00
104461
2
3
1
0

genus

 0.00
934941
2
1
2
0


 0.00
species
934942
2
1

3
50
30
1
104450

 0.01
subfamily

104458
genus

 0.00
0
6
2
1

species

 0.00
104460
6
1

genus

 0.00
934939
2
1
4
0


 0.00
species
934940
4
1


 0.00
genus
214132
1
2
3
0

1
3
934839

 0.00
species

934916
genus

 0.00
0
2
1
2

species

 0.00
934917
2
1

1
2
1
0
genus

 0.00
326956

987013

 0.00
species
1
1

214392

 0.00
genus
0
1
2
4

934828
species

 0.00
1
4

0
1
2
2
873511
genus

 0.00

934888

 0.00
species
2
1

2
1
2
0

 0.00
genus
104451

1
2
104452
species

 0.00


 0.00
genus
214189
1
2
1
0

934876
species

 0.00
1
1

0
2
1
2
190368
genus

 0.00

934904
species

 0.00
1
2

1
2
1
0
genus

 0.00
934935

1
1
species

 0.00
934936

genus

 0.00
104456
1
2
1
0

1
1
104457
species

 0.00


 0.00
genus
214137
18
1
5
0


 0.00
species
934840
2
1

4
1
934844

 0.00
species

7
1
species

 0.00
934866

934847

 0.00
species
5
1

37573

 0.01
superfamily
4
70
30
1

0
20
11
1
7135
family

 0.00

299347

 0.00
subfamily
0
5
1
10

5
1
2
0
genus

 0.00
1101094

1
5
1101095

 0.00
species

5
1
2
0

 0.00
genus
1101109

5
1
1101110

 0.00
species

0
10
1
5
40083

 0.00
subfamily

2
1
6
0

 0.00
genus
989881

1
6
species

 0.00
1666458

0
1
2
4
687068

 0.00
genus

species

 0.00
1857951
4
1


 0.01
family
268499
18
1
46
0

30
1
12
4
subfamily

 0.00
40081

genus

 0.00
40084
1
2
5
0

5
1

 0.00
species
40085

1
2
4
0
genus

 0.00
1368975

1
4
1371681
species

 0.00

0
1
3
5
572808
genus

 0.00


 0.00
species
1660579
1
2

1
3
1594226

 0.00
species

0
4
1
2
572825
genus

 0.00

1594250

 0.00
species
4
1

8
2
1
0

 0.00
genus
168630

1
8
168631

 0.00
species

5
1
16
0
subfamily

 0.00
299362

2
1
10
0
genus

 0.00
687116

1594321
species

 0.00
1
10

2
1
6
0
genus

 0.00
1666817

1
6
species

 0.00
1666818


 0.04
superfamily
37572
306
110
1
30


 0.00
family
27544
32
20
1
0

42297
subfamily

 0.00
3
29
1
16

1
2
4
2
genus

 0.00
91737

species

 0.00
91739
1
2

0
2
1
2
203780
genus

 0.00

203781
species

 0.00
2
1


 0.00
genus
265382
2
2
1
0

1
2
species

 0.00
265386


 0.00
genus
138069
3
1
3
0

1
1
268709

 0.00
species

1
2
138070

 0.00
species


 0.00
genus
1821620
1
2
2
0

988025
species

 0.00
2
1


 0.00
genus
242266
2
1
4
0

242267

 0.00
species
4
1

genus

 0.00
42298
9
2
1
0

species

 0.00
203782
9
1

1
3
3
0

 0.00
subfamily
124406

3
2
1
0

 0.00
genus
265359

3
1
282391

 0.00
species

7114

 0.01
family
2
36
1
14

0
1
3
3
151208

 0.00
subfamily

3
1
2
0
genus

 0.00
189907

3
1

 0.00
species
189913

42449
subfamily

 0.00
0
31
10
1

tribe

 0.00
152601
1
9
31
0

0
1
2
8
129396
genus

 0.00

129397

 0.00
species
1
8


 0.00
genus
7115
1
4
19
2

2
1
64459

 0.00
species

1
2
7116
species

 0.00

13
1

 0.00
species
78633

0
1
2
4
72244

 0.00
genus

1
4
227532

 0.00
species

1
67
201
10
family

 0.03
33415

40037
subfamily

 0.00
0
18
1
11

16
1
7
1

 0.00
tribe
127312

0
4
2
1
405031

 0.00
genus

1
4
species

 0.00
405034


 0.00
genus
525812
8
1
2
0

405009

 0.00
species
1
8

127313

 0.00
genus
0
3
2
1

species

 0.00
191398
1
3


 0.00
tribe
127322
1
3
2
0

33416

 0.00
genus
0
1
2
2


 0.00
species
33443
2
1

subfamily

 0.00
100750
4
1
21
0

21
1
3
0
tribe

 0.00
215788


 0.00
genus
124410
1
2
21
0


 0.00
species
270466
21
1

1
17
1
61
40040

 0.01
subfamily

tribe

 0.01
171576
1
10
36
0

2
12
6
1
76218

 0.00
genus

442324

 0.00
subgenus
0
2
1
2

species

 0.00
171594
1
2

0
3
1
8
111880

 0.00
subgenus

1
5
111881
species

 0.00

3
1
171585

 0.00
species

42274

 0.00
genus
0
24
3
1

1
8
42275
species

 0.00

16
1
171605
species

 0.00

6
1
24
0

 0.00
tribe
171578

171580
subtribe

 0.00
0
5
1
24

104514

 0.00
genus
0
2
1
15

species

 0.00
113334
1
15

596672
genus

 0.00
0
9
1
2

113330
species

 0.00
1
9

42282

 0.01
subfamily
0
79
26
1

127320

 0.01
tribe
3
77
1
21

150886
subtribe

 0.00
0
3
1
2

1
2
2
0
genus

 0.00
111908

2
1
111912
species

 0.00

subtribe

 0.00
150884
1
3
8
0

111922

 0.00
genus
0
8
1
2

111923
species

 0.00
1
8

150883
subtribe

 0.00
0
28
1
6

0
28
5
1
111919

 0.00
genus

subgenus

 0.00
111885
2
1
5
0

species

 0.00
2795564
5
1

111950

 0.00
no rank
0
2
1
23

191418

 0.00
species
1
23

13
5
1
0
subtribe

 0.00
366209

genus

 0.00
111932
11
1
2
0

1
11

 0.00
species
116150

111915

 0.00
genus
0
2
2
1

1
2
species

 0.00
111917

0
1
3
23
167180
subtribe

 0.00

0
23
2
1
110367
genus

 0.00


 0.00
species
110368
23
1


 0.00
tribe
1664845
1
4
2
0

0
2
3
1
111897
genus

 0.00

1
1
111903
species

 0.00

447833

 0.00
species
1
1

0
12
1
8
127218
subfamily

 0.00

tribe

 0.00
42315
1
7
12
0

0
12
1
6
344711

 0.00
subtribe

64444
genus

 0.00
1
1
5
12

species

 0.00
331299
10
1
2
0

10
1
subspecies

 0.00
331333

0
1
2
1
304554
species

 0.00

1
1

 0.00
subspecies
331302


 0.00
family
7143
7
1
8
0


 0.00
subfamily
42289
7
1
7
0

189314

 0.00
tribe
0
1
3
6

157396
genus

 0.00
0
6
1
2

110791

 0.00
species
6
1

tribe

 0.00
189315
1
3
1
0

0
1
2
1
7145
genus

 0.00

66420
species

 0.00
1
1

superfamily

 0.00
37581
17
1
29
0


 0.00
family
2681869
9
8
1
0

0
4
1
3
2925401
no rank

 0.00

0
2
1
4
116120
genus

 0.00

1
4

 0.00
species
116121

0
5
1
4
116119
subfamily

 0.00

262437
genus

 0.00
0
5
1
3

1
3
1857958

 0.00
species


 0.00
species
1594222
1
2

0
10
1
4
57992

 0.00
family

10
3
1
0

 0.00
subfamily
116123

688988

 0.00
genus
0
1
2
10

1
10
species

 0.00
2870497

family

 0.00
173649
10
4
1
0

655692

 0.00
genus
6
10
3
1


 0.00
species
1869501
1
2


 0.00
species
2561016
1
2

37584

 0.00
superfamily
0
5
7
1

family

 0.00
98958
1
3
2
0

2
2
1
0

 0.00
genus
101736

1
2
101737

 0.00
species

0
3
1
3
30222

 0.00
family

2
1
3
0
genus

 0.00
753374

3
1

 0.00
species
753375

41024

 0.00
suborder
0
1
4
8

41025

 0.00
family
0
1
3
8

2
1
8
0

 0.00
genus
41026

1042620

 0.00
species
8
1

0
36
1
13
30263
order

 0.01

12
1
36
0

 0.01
suborder
93875

36
1
11
0
infraorder

 0.01
1683728

0
36
10
1
41033
superfamily

 0.01


 0.01
family
50645
9
1
36
0

177669

 0.01
subfamily
0
36
8
1


 0.01
tribe
177673
7
1
36
0

2
1
12
0

 0.00
genus
1271741

1271742

 0.00
species
12
1

0
24
1
4
177674
genus

 0.00


 0.00
species
692089
9
1

11
1
1271730
species

 0.00


 0.00
species
1218281
1
4

1
213
1
203
7399
order

 0.03

2
198
187
1
7400
suborder

 0.03

infraorder

 0.00
1955251
1
40
18
0

7401

 0.00
superfamily
0
33
1
17

12
1
23
0

 0.00
family
7408


 0.00
subfamily
65140
4
1
2
0

288186
no rank

 0.00
0
2
1
3

2
1
2
0

 0.00
genus
29048


 0.00
species
1539398
1
2

subfamily

 0.00
65170
1
4
3
0

0
1
3
3
172511

 0.00
tribe


 0.00
genus
27520
3
2
1
0

1
3

 0.00
species
2795680

4
1
2
0

 0.00
subfamily
65163

2
1
3
0
tribe

 0.00
176302

2
1
2
0
genus

 0.00
493657

2870495
species

 0.00
2
1

65167
subfamily

 0.00
0
5
1
1

231867
tribe

 0.00
0
1
1
4

3
1
1
0

 0.00
genus
65288


 0.00
subgenus
494744
1
2
1
0

1
1
2866289
species

 0.00

subfamily

 0.00
65171
4
1
5
0

172377

 0.00
tribe
0
4
1
4

231877
subtribe

 0.00
0
4
3
1


 0.00
genus
231878
2
1
4
0

1
4
1419289

 0.00
species

7402
family

 0.00
0
9
1
5


 0.00
subfamily
68882
3
1
2
0

2
1
2
0

 0.00
genus
37852


 0.00
species
684658
1
2

3
1
5
0
subfamily

 0.00
65207

genus

 0.00
51538
1
2
1
0

1
1
69319

 0.00
species

32390

 0.00
genus
0
1
2
2

species

 0.00
32391
1
2

6
1
1
0
superfamily

 0.00
7422


 0.00
family
75187
5
1
1
0

0
1
4
1
75190
subfamily

 0.00

84507
genus

 0.00
0
1
3
1

2
1
1
0

 0.00
species
142686

326594
subspecies

 0.00
1
1

178
146
1
2

 0.02
infraorder
7434

2153482
superfamily

 0.00
0
5
1
3

92421

 0.00
family
0
3
1
4


 0.00
subfamily
219387
1
3
3
0

3
1
2
0
genus

 0.00
219389


 0.00
species
330862
3
1

0
1
5
3
1803217

 0.00
superfamily

3
4
1
0

 0.00
family
27515

0
3
3
1
1801551

 0.00
subfamily

0
3
1
2
200613

 0.00
genus

1667466

 0.00
species
3
1

6
87
1
66
34735
superfamily

 0.01

4
1
1
0

 0.00
family
156309


 0.00
subfamily
178044
3
1
1
0

156310
genus

 0.00
0
1
2
1

935657

 0.00
species
1
1

2153468

 0.00
no rank
0
20
1
14

253718

 0.00
family
0
19
1
14

253723
subfamily

 0.00
0
2
4
1

421418

 0.00
tribe
0
3
1
2

0
2
1
2
421423

 0.00
genus

2
1
2495085
species

 0.00

0
4
1
1
216423
subfamily

 0.00

288382

 0.00
tribe
0
1
3
1

288388

 0.00
genus
0
1
2
1

1
1
1167272
species

 0.00

1
6
8
0

 0.00
subfamily
253722

0
8
5
1
288410
tribe

 0.00

0
4
1
8
421285

 0.00
subtribe


 0.00
genus
421297
8
3
1
0

1
5
1126389

 0.00
species

3
1

 0.00
species
2495015

0
3
4
1
288404

 0.00
subfamily

302523

 0.00
tribe
0
3
1
3

3
2
1
0
genus

 0.00
421220

2495127

 0.00
species
3
1


 0.00
family
124286
13
1
9
0

156330

 0.00
subfamily
0
12
1
9

0
4
1
2
156332

 0.00
tribe

1
3
1
2
124287
genus

 0.00

1437190
species

 0.00
0
1
1
2

1
1
1437191
subspecies

 0.00

0
1
1
3
156337
tribe

 0.00

216413
genus

 0.00
0
1
2
1

1
1
2249760
species

 0.00

156331

 0.00
tribe
0
1
4
6

6
1
3
0
genus

 0.00
132116

4
1
481575

 0.00
species


 0.00
species
1542540
2
1

0
1
5
1
48719
family

 0.00

205141
subfamily

 0.00
0
4
1
1

0
1
3
1
48720
genus

 0.00

subgenus

 0.00
205261
2
1
1
0

1
1
1411667
species

 0.00

family

 0.00
77572
1
18
18
0

0
17
1
18
77573

 0.00
subfamily

3
1
6
0

 0.00
tribe
479730

0
2
1
6
88591

 0.00
genus

1
6
species

 0.00
1190790


 0.00
tribe
88545
1
3
1
0


 0.00
genus
115080
1
1
2
0

species

 0.00
115081
1
1

11
10
1
0
tribe

 0.00
88544

88466
genus

 0.00
0
3
2
1

115100
species

 0.00
3
1

0
7
1
8
88467

 0.00
genus

0
1
2
3
88474
subgenus

 0.00

88516
species

 0.00
1
3

0
2
2
1
88475
subgenus

 0.00

species

 0.00
88514
2
1


 0.00
subgenus
88472
3
2
1
0

species

 0.00
88531
1
3

family

 0.00
7458
11
22
1
0

0
2
1
4
78169
subfamily

 0.00

0
3
1
2
95294

 0.00
tribe

0
2
2
1
95295
genus

 0.00

601510

 0.00
species
1
2

subfamily

 0.00
70987
1
17
9
0

tribe

 0.00
83323
1
3
1
0

117248

 0.00
genus
0
1
2
1

1
1

 0.00
species
597456


 0.00
tribe
83311
7
1
10
0

7
9
1
0
genus

 0.00
28641

3
1
3
1
subgenus

 0.00
144703

1
1
species

 0.00
30191

1
1

 0.00
species
30194

0
2
1
1
144700
subgenus

 0.00

65598

 0.00
species
1
1

1
2
1
0
subgenus

 0.00
144704

species

 0.00
85660
1
1

1
2
subgenus

 0.00
144708

83321
tribe

 0.00
0
1
3
1

1
2
1
0
genus

 0.00
7459

1
1

 0.00
species
7463

0
4
1
6
156323
family

 0.00

subfamily

 0.00
253710
6
1
3
0

genus

 0.00
253714
6
2
1
0

6
1
species

 0.00
253715

0
32
18
1
34725
superfamily

 0.00


 0.00
family
7438
32
1
17
0

0
1
4
1
7455

 0.00
subfamily


 0.00
tribe
76984
1
3
1
0


 0.00
genus
7456
1
1
2
0

1
1
30207

 0.00
species

1
9
29
5

 0.00
subfamily
7439

7451
genus

 0.00
2
6
3
1

2
1

 0.00
species
7454

1
2
30212

 0.00
species

5
1
3
16
7443
genus

 0.00

species

 0.00
7445
6
1

1
5
202808
species

 0.00

7440

 0.00
genus
1
2
1
2

85444
species

 0.00
1
1

50638
subfamily

 0.00
0
1
3
2


 0.00
genus
76989
2
1
2
0


 0.00
species
76990
2
1


 0.01
superfamily
2153479
1
30
72
0

0
72
1
29
36668
family

 0.01

1
15
1
64
34695

 0.01
subfamily

3
1
5
0
tribe

 0.00
144017

genus

 0.00
55077
2
2
1
0

307658

 0.00
species
1
2

genus

 0.00
13685
1
2
1
0


 0.00
species
13686
1
1

144003

 0.00
tribe
0
2
3
1

genus

 0.00
369112
2
1
2
0

species

 0.00
606695
1
2

0
1
3
1
144001

 0.00
tribe

0
1
1
2
64792

 0.00
genus

1
1
64793
species

 0.00

144020

 0.01
tribe
0
1
3
57


 0.01
genus
30204
1
2
57
0

species

 0.01
219812
57
1

subfamily

 0.00
213859
5
3
1
0

1
2
5
0
genus

 0.00
2015172

5
1
species

 0.00
2015173

0
1
4
1
7479

 0.00
subfamily

3
1
1
0
tribe

 0.00
72772

genus

 0.00
710235
1
2
1
0

1
1
613905

 0.00
species

2
1
6
0

 0.00
subfamily
43085

2
1
5
0

 0.00
tribe
141711

0
2
1
1
604375

 0.00
genus

610380
species

 0.00
1
1

genus

 0.00
43086
1
2
1
0

species

 0.00
486640
1
1

85772

 0.00
superfamily
0
1
11
13

12
1
7
0

 0.00
family
27532

112287
subfamily

 0.00
0
12
1
6

4
2
1
0
genus

 0.00
1250642


 0.00
species
1385029
1
4

0
8
1
3
112291

 0.00
genus

1
3
species

 0.00
362091

222778
species

 0.00
1
5

0
1
3
1
2982298
family

 0.00


 0.00
genus
37343
1
2
1
0

1
1
37344

 0.00
species

1
4
1
0
superfamily

 0.00
222831

family

 0.00
27528
3
1
1
0

0
1
1
2
27529

 0.00
genus

222816
species

 0.00
1
1


 0.02
order
7041
121
109
1
7

0
16
1
23
41071
suborder

 0.00


 0.00
superfamily
535382
23
15
1
0

41073

 0.00
family
0
1
14
23


 0.00
subfamily
71541
16
8
1
0

0
1
4
10
879230
tribe

 0.00

3
1
10
0
genus

 0.00
41078

2
1
10
0
subgenus

 0.00
484232

767470
species

 0.00
10
1

60759
tribe

 0.00
0
3
1
6

247414

 0.00
genus
0
6
1
2

1
6

 0.00
species
247415

7
5
1
0
subfamily

 0.00
71539

0
4
1
7
60833
tribe

 0.00

genus

 0.00
60836
2
1
4
0

4
1
878056
species

 0.00

1
3
genus

 0.00
60834

41084
suborder

 0.01
5
1
92
91

11
1
13
0
infraorder

 0.00
41087

1
1
5
0
superfamily

 0.00
71192

1
4
1
0

 0.00
family
50527

1
3
1
0

 0.00
subfamily
261156

0
1
2
1
195164
genus

 0.00

1
1
224129
species

 0.00

71193

 0.00
superfamily
0
1
7
10

10
6
1
0
family

 0.00
41097

433502

 0.00
subfamily
0
5
1
10

186072

 0.00
genus
0
1
2
6

6
1
species

 0.00
1553677

genus

 0.00
41098
4
1
2
0

4
1
species

 0.00
195172

1
64
51
3

 0.01
infraorder
41088

5
1
3
0
superfamily

 0.00
71526

0
4
1
3
116151
family

 0.00

3
1
3
0

 0.00
subfamily
577241

1431902
genus

 0.00
0
1
2
3

3
1
species

 0.00
1431903

0
1
16
6
71529
superfamily

 0.00

7042

 0.00
family
0
5
1
11

39814
subfamily

 0.00
0
1
1
4


 0.00
tribe
465383
1
1
3
0

genus

 0.00
122852
1
1
2
0


 0.00
species
202137
1
1

39812

 0.00
subfamily
0
3
1
2


 0.00
genus
7045
2
1
2
0

7048
species

 0.00
2
1

123516

 0.00
subfamily
0
2
1
3

0
2
2
1
201855

 0.00
genus

1
2
467358
species

 0.00

0
4
1
1
122737

 0.00
family

0
1
1
3
701798

 0.00
subfamily

1
1
2
0
genus

 0.00
122772

species

 0.00
201766
1
1

0
7
1
11
2939015

 0.00
superfamily

7
1
10
0

 0.00
family
7080


 0.00
subfamily
7081
9
1
7
0


 0.00
tribe
263632
1
3
1
0

0
1
1
2
347358

 0.00
genus

species

 0.00
347359
1
1

1
5
6
0

 0.00
tribe
263631

115356
genus

 0.00
0
4
1
2

species

 0.00
115357
1
4

7083
genus

 0.00
0
2
1
2

species

 0.00
7084
2
1

0
10
1
8
71527

 0.00
superfamily

0
4
1
3
55098

 0.00
family


 0.00
genus
295984
2
1
4
0

346838

 0.00
species
1
4

0
6
4
1
7065
family

 0.00

0
3
1
6
1304792

 0.00
no rank


 0.00
genus
7069
2
1
6
0

7072
species

 0.00
1
6

71525

 0.00
superfamily
0
5
5
1

186093
family

 0.00
0
4
1
5


 0.00
subfamily
353826
1
3
5
0

genus

 0.00
295699
5
1
2
0

1
5

 0.00
species
295700

71528

 0.00
superfamily
0
17
1
18

27439

 0.00
family
0
17
1
17


 0.00
subfamily
131688
3
1
2
0

1
2
2
0

 0.00
genus
204943

2
1

 0.00
species
204949

6
1
6
0
subfamily

 0.00
63707

tribe

 0.00
63708
5
1
6
0

genus

 0.00
41125
2
1
2
0

2
1
1587174

 0.00
species

1
2
4
0
genus

 0.00
80248


 0.00
species
80249
4
1

9
1
7
0
subfamily

 0.00
63710

131578
tribe

 0.00
0
9
1
6

1
2
6
0
genus

 0.00
294691

1
6
species

 0.00
2598218


 0.00
genus
224132
3
3
1
0

444603

 0.00
species
2
1

1
1
224133

 0.00
species

41085
infraorder

 0.00
0
24
14
1

75543

 0.00
superfamily
1
24
13
1

1
8
15
0
family

 0.00
29026

15
7
1
0
no rank

 0.00
351514

82886
subfamily

 0.00
0
1
6
15

15
5
1
0
tribe

 0.00
295648

9
1
2
0

 0.00
genus
290671

662956
species

 0.00
1
9

0
1
2
6
219450

 0.00
genus

species

 0.00
346820
6
1

family

 0.00
57514
4
1
8
0

subfamily

 0.00
82881
8
3
1
0

0
8
1
2
414933

 0.00
genus


 0.00
species
414934
1
8

15
261
1
198
7147
order

 0.04

7148

 0.01
suborder
1
70
60
1

0
6
1
6
43784

 0.00
infraorder

5
1
6
0

 0.00
superfamily
43790

1
4
6
0

 0.00
family
52729

52730
subfamily

 0.00
0
6
1
3

189978
genus

 0.00
0
6
2
1


 0.00
species
189979
1
6

1
7
1
0
infraorder

 0.00
43787

1
6
1
0
superfamily

 0.00
41831

7197

 0.00
family
0
1
5
1

subfamily

 0.00
7198
4
1
1
0

1
3
1
0

 0.00
genus
7199

252607
subgenus

 0.00
0
2
1
1

1
1

 0.00
species
7200

43786

 0.01
infraorder
0
62
1
46

41828

 0.00
superfamily
0
5
1
13

0
4
6
1
41819

 0.00
family

43801

 0.00
subfamily
0
4
5
1

58262

 0.00
tribe
0
4
4
1

41820
genus

 0.00
0
1
3
4


 0.00
subgenus
58277
1
2
4
0

179676
species

 0.00
1
4

0
1
6
1
7149
family

 0.00

54970

 0.00
subfamily
0
1
5
1

72530
tribe

 0.00
0
1
4
1

7150
genus

 0.00
0
3
1
1

0
1
2
1
1165752
no rank

 0.00

1
1
315576
species

 0.00

superfamily

 0.01
41827
1
32
57
0

family

 0.01
7157
1
31
57
0

3
1
6
0

 0.00
subfamily
43817

0
3
1
5
53550

 0.00
tribe

genus

 0.00
7174
3
4
1
0


 0.00
subgenus
53527
3
3
1
0

no rank

 0.00
518105
3
2
1
1


 0.00
species
7176
1
2

1
24
54
0
subfamily

 0.01
43816

1
23
54
0
genus

 0.01
7164

subgenus

 0.00
44482
4
1
6
0

58247
section

 0.00
0
4
1
5


 0.00
series
58250
4
1
4
0

0
1
3
4
59130

 0.00
species group


 0.00
species
345580
1
3

species

 0.00
139045
1
1

1
8
40
0

 0.01
subgenus
44543

0
39
1
4
44545

 0.01
clade

clade

 0.01
44546
1
3
39
0

44552

 0.01
species group
0
2
1
39

1
39
43151
species

 0.01

44544

 0.00
clade
0
3
1
1

44547

 0.00
clade
0
1
2
1

species

 0.00
7167
1
1


 0.00
subgenus
44534
6
1
8
0


 0.00
clade
59140
1
1
2
0


 0.00
species
1521116
1
1

44535

 0.00
clade
0
7
1
3

1
5

 0.00
species
30069

1
2
1496333

 0.00
species

0
1
2
2
68877
subgenus

 0.00


 0.00
species
68878
1
2

0
137
1
176
7203

 0.02
suborder

0
5
1
1
43734

 0.00
infraorder

34687

 0.00
family
0
1
4
1

1
1
3
0

 0.00
subfamily
343564

1
1
2
0
genus

 0.00
343581


 0.00
species
343691
1
1

2
175
1
131
43733
infraorder

 0.02

superfamily

 0.00
50671
9
1
8
0

4
4
1
0

 0.00
family
50674

4
3
1
0

 0.00
subfamily
50694

1
2
4
0
genus

 0.00
219362

4
1
240869
species

 0.00

4
4
1
0

 0.00
family
50673

subfamily

 0.00
50679
3
1
4
0

0
2
1
4
247604
genus

 0.00

species

 0.00
2794001
1
4

480118
clade

 0.02
0
121
1
165

1
120
165
4
clade

 0.02
480117

no rank

 0.01
43737
38
35
1
0

0
34
1
38
43740

 0.01
superfamily

0
1
29
34
34680

 0.00
family

43838
subfamily

 0.00
0
16
1
13

0
9
1
10
115274
tribe

 0.00


 0.00
genus
414810
1
1
2
0

1
1
species

 0.00
1352479

414800

 0.00
genus
0
2
1
2

1
2
414801
species

 0.00

286458

 0.00
genus
0
2
1
2

2
1
286459
species

 0.00

5
2
1
0
genus

 0.00
219538


 0.00
species
219539
5
1

224219
tribe

 0.00
0
6
3
1

1
2
6
0

 0.00
genus
192444

species

 0.00
414846
1
6

0
15
1
18
115244
subfamily

 0.00

4
4
1
0

 0.00
tribe
192448

4
3
1
0
genus

 0.00
173981

273409

 0.00
species
2
1

1
2
173985
species

 0.00

3
1
3
0

 0.00
tribe
224230

226146

 0.00
genus
0
1
2
3

species

 0.00
226147
3
1

115277

 0.00
tribe
0
1
7
11

1
4
4
0

 0.00
genus
198633

species

 0.00
1124515
1
1

species

 0.00
198635
1
1

2
1

 0.00
species
1572519

7
2
1
0
genus

 0.00
115278

7
1

 0.00
species
2725509

1
4
4
0
family

 0.00
43835

subfamily

 0.00
115302
1
3
4
0

4
1
2
0

 0.00
genus
115303

4
1
566305
species

 0.00

43738

 0.02
no rank
1
123
1
84


 0.02
no rank
43741
119
1
72
0

43752

 0.00
superfamily
0
6
1
19

7211

 0.00
family
0
6
18
1

5
1
2
0
subfamily

 0.00
43867

2
4
1
0
tribe

 0.00
43901

164882

 0.00
subtribe
0
1
3
2

genus

 0.00
28609
1
2
2
0

28612
species

 0.00
2
1

4
1
12
0
subfamily

 0.00
164860

43871

 0.00
tribe
0
1
7
3

genus

 0.00
47833
2
1
3
0


 0.00
subgenus
1987911
1
2
2
0

2
1

 0.00
species
28588

27456
genus

 0.00
0
1
3
1

0
2
1
1
69624

 0.00
subgenus


 0.00
species
104688
1
1

tribe

 0.00
164862
1
4
1
0

1
3
1
0

 0.00
genus
7212

0
1
2
1
474492
subgenus

 0.00

species

 0.00
7213
1
1

1
9
8
0
superfamily

 0.00
43744

115263

 0.00
family
0
1
8
8


 0.00
subfamily
115265
7
1
8
0


 0.00
genus
1219203
2
1
4
0

4
1
species

 0.00
1219204

2
2
1
0

 0.00
genus
305546

1219171

 0.00
species
2
1

2
1
2
0

 0.00
genus
286486

2829445
species

 0.00
1
2

1
39
104
0

 0.01
superfamily
43746

7214

 0.01
family
0
38
1
104


 0.01
subfamily
43845
37
1
104
0

1
36
104
0

 0.01
tribe
46877

13
35
1
104
7215
genus

 0.01

subgenus

 0.00
32280
1
2
6
0


 0.00
species
30019
1
6


 0.01
no rank
504493
6
1
61
0


 0.01
clade
48384
61
1
5
0

48301

 0.01
clade
0
61
1
4

48302

 0.01
species group
0
3
1
61

2
1
61
0

 0.01
species subgroup
32378

61
1
7222

 0.01
species


 0.00
subgenus
32281
8
1
10
0

32335

 0.00
species group
0
2
1
1

1
1
47314
species

 0.00


 0.00
species group
32304
3
1
1
0

0
1
1
2
32307
species subgroup

 0.00

species

 0.00
7291
1
1

0
2
1
8
32320

 0.00
species group

1
8
198719

 0.00
species

32341
subgenus

 0.00
0
14
1
18

0
1
1
3
32365

 0.00
species group

32367
species subgroup

 0.00
0
2
1
1

species

 0.00
7260
1
1

32355
species group

 0.00
0
7
1
6

32358

 0.00
species subgroup
0
1
2
4

species

 0.00
7229
4
1


 0.00
species subgroup
32357
3
3
1
0

7241

 0.00
species
1
1

7266
species

 0.00
2
1

species group

 0.00
32346
6
1
8
0

2
1
3
0
species subgroup

 0.00
65962

3
1
1041015
species

 0.00

species subgroup

 0.00
32351
2
1
3
0

1
1
species

 0.00
7227

species

 0.00
7220
1
1

0
2
1
1
32354

 0.00
species subgroup

29030

 0.00
species
1
1

superfamily

 0.00
43750
1
4
1
0

0
3
1
1
169447
family

 0.00

genus

 0.00
1226614
2
1
1
0

1
1
1226616
species

 0.00

0
1
11
3
43742

 0.00
no rank

0
10
1
3
43755
superfamily

 0.00

2
4
1
0

 0.00
family
7371

0
1
3
2
43912

 0.00
subfamily

670600

 0.00
genus
0
2
1
2

2795671
species

 0.00
2
1

family

 0.00
27474
1
5
1
0

subfamily

 0.00
43917
1
4
1
0

179426
tribe

 0.00
0
3
1
1

0
1
2
1
569039
genus

 0.00

569040

 0.00
species
1
1

31
1
14
0

 0.00
cohort
33342


 0.00
order
7524
30
1
14
1

0
11
1
2
33343
clade

 0.00


 0.00
suborder
33345
1
10
2
0

33347
clade

 0.00
0
9
1
2

0
1
8
2
33349
clade

 0.00

clade

 0.00
33351
2
1
7
0


 0.00
infraorder
33357
6
1
2
0

superfamily

 0.00
38105
5
1
2
0

186376

 0.00
family
0
4
1
2


 0.00
subfamily
2068237
3
1
2
0

1
2
2
0
genus

 0.00
1276926

1545138
species

 0.00
1
2

suborder

 0.00
33373
11
18
1
0

33375
superfamily

 0.00
0
1
4
1

family

 0.00
1585420
1
3
1
0

1
1
2
0

 0.00
genus
121844

1
1
121845

 0.00
species

1
5
4
0
superfamily

 0.00
33377

7036
family

 0.00
0
1
4
4

33379
subfamily

 0.00
0
1
3
4

4
2
1
0
genus

 0.00
7037

species

 0.00
7038
4
1

infraorder

 0.00
33380
6
8
1
0

7
1
6
0

 0.00
superfamily
33385

family

 0.00
27482
6
1
6
1

5
1
5
1
subfamily

 0.00
133076

0
1
4
4
33387

 0.00
tribe

4
1
3
0

 0.00
genus
80764


 0.00
subgenus
464929
4
1
2
0

4
1
80765

 0.00
species

6657
subphylum

 0.01
0
49
1
54

38
32
1
0
superclass

 0.01
2172821


 0.00
class
6681
16
1
16
0

0
15
1
16
72041

 0.00
subclass

6682
superorder

 0.00
0
14
1
16

6683
order

 0.00
0
13
1
16

10
1
6
0

 0.00
suborder
6692

0
5
1
10
6694
infraorder

 0.00

0
10
1
4
115580

 0.00
superfamily


 0.00
family
6695
1
3
10
0

10
2
1
0

 0.00
genus
6696

10
1
159736

 0.00
species


 0.00
suborder
6684
6
1
6
0

111520

 0.00
superfamily
0
1
5
6

6685

 0.00
family
0
6
4
1

133894

 0.00
genus
1
3
1
6


 0.00
species
6687
1
1

4
1
27405

 0.00
species

72037

 0.00
class
0
22
1
15

6830

 0.00
subclass
0
22
14
1

22
1
13
0

 0.00
infraclass
116569

0
1
5
1
116570

 0.00
superorder


 0.00
order
6833
4
1
1
0

1
1
3
0

 0.00
family
88013

genus

 0.00
88014
1
2
1
0

1
1
88015
species

 0.00

0
21
7
1
116571
superorder

 0.00


 0.00
order
72033
21
6
1
0

family

 0.00
72034
21
1
5
0

217164

 0.00
genus
0
14
1
2

217165

 0.00
species
1
14

1
2
7
0
genus

 0.00
72035

species

 0.00
72036
7
1

0
16
1
16
2172819
superclass

 0.00

6670

 0.00
class
0
16
15
1


 0.00
subclass
43953
1
14
16
0

84318
order

 0.00
1
1
13
16

0
1
7
9
84328

 0.00
suborder

84329
superfamily

 0.00
0
6
1
9


 0.00
family
43954
9
1
5
0

2
1
7
0
genus

 0.00
399044

399045

 0.00
species
7
1

genus

 0.00
163713
1
2
2
0

163714
species

 0.00
2
1


 0.00
suborder
116574
1
5
6
0

superfamily

 0.00
116575
1
4
6
0

family

 0.00
69351
6
3
1
0

1
2
6
0
genus

 0.00
69354

1
6
69355
species

 0.00

6843
subphylum

 0.01
0
59
62
1

6854

 0.01
class
1
61
1
59


 0.01
subclass
6933
55
48
1
0


 0.00
superorder
6934
18
16
1
0

0
1
7
1
34634

 0.00
order

0
1
6
1
281668
suborder

 0.00

5
1
1
0

 0.00
infraorder
1723665

superfamily

 0.00
41438
1
4
1
0

3
1
1
0
family

 0.00
109261

1
1
2
0

 0.00
genus
62624

1
1

 0.00
species
62625

8
1
17
0

 0.00
order
6935

17
1
7
0

 0.00
superfamily
297308

17
6
1
0
family

 0.00
6939

1
1
5
17
426437

 0.00
subfamily

genus

 0.00
34630
16
1
4
1

0
15
3
1
426455
subgenus

 0.00

species group

 0.00
578835
2
1
15
1

species

 0.00
34632
14
1

4
37
1
31
6946

 0.01
superorder


 0.00
order
83137
1
22
27
0

8
1
12
0
suborder

 0.00
66551

0
1
7
12
229894
infraorder

 0.00

229794
superfamily

 0.00
0
12
6
1

family

 0.00
229795
12
5
1
0

1979940
genus

 0.00
0
8
2
1

8
1
species

 0.00
1979941

2
1
4
0
genus

 0.00
334624

334625
species

 0.00
1
4

6951
suborder

 0.00
0
15
13
1

0
1
12
15
223472
parvorder

 0.00

10
1
6
0
superfamily

 0.00
83163

6952
family

 0.00
0
1
5
10

10
4
1
0

 0.00
subfamily
474036

6953

 0.00
genus
0
1
3
10

1
8
6956
species

 0.00

6954

 0.00
species
1
2

83158

 0.00
superfamily
0
5
1
5

52281
family

 0.00
0
4
1
5

474019
subfamily

 0.00
0
3
1
5

52282

 0.00
genus
0
2
1
5

5
1

 0.00
species
52283

6
1
8
1

 0.00
order
83136

1
5
7
1
6947
suborder

 0.00


 0.00
infraorder
83138
4
6
1
0

5
1
4
2
clade

 0.00
83141


 0.00
superfamily
92088
1
4
2
0

92251
family

 0.00
1
2
1
3

0
1
1
2
1712447
genus

 0.00

species

 0.00
1712448
1
1

3
12
1
0
order

 0.00
6893

0
3
11
1
6905

 0.00
suborder

0
10
1
3
74971
clade

 0.00

74974

 0.00
clade
0
2
1
5

2
4
1
0
superfamily

 0.00
74975

0
2
3
1
27394
family

 0.00

94025
genus

 0.00
0
2
2
1

2
1
1926196
species

 0.00

4
1
1
0
superfamily

 0.00
175332

0
1
1
3
175333
family

 0.00


 0.00
genus
175340
2
1
1
0

1
1
202533

 0.00
species

52
1
28
0
clade

 0.00
2698737


 0.00
clade
33634
8
18
1
0

1
9
1
4
4762
phylum

 0.00

2
3
1
0

 0.00
order
4763

family

 0.00
4764
2
1
2
1

1
1

 0.00
genus
4769

370421

 0.00
order
0
1
5
1

1
4
1
0
family

 0.00
65355


 0.00
genus
65356
1
1
3
0

0
2
1
1
653948

 0.00
species

1
1
isolate

 0.00
890382

4
8
1
1

 0.00
clade
2696291

1
7
3
1
phylum

 0.00
2836

0
6
1
2
33853
class

 0.00

0
5
1
2
33854

 0.00
subclass


 0.00
order
2108076
2
4
1
0


 0.00
family
2108078
2
3
1
0

genus

 0.00
426668
2
1
2
0

1
2
1436140
species

 0.00

20
1
33
0

 0.00
clade
33630

phylum

 0.00
5878
9
1
3
1

2
1
8
1

 0.00
subphylum
431838

0
1
7
1
6020

 0.00
class

31277
order

 0.00
0
1
6
1

37093
suborder

 0.00
0
5
1
1

1
4
1
0

 0.00
family
291294

5890

 0.00
genus
0
1
3
1

5911
species

 0.00
0
1
2
1

312017

 0.00
strain
1
1

phylum

 0.00
5794
23
1
17
0

0
5
15
1
422676

 0.00
class

1
5
1
0
order

 0.00
5863

4
1
1
0
family

 0.00
32594


 0.00
genus
5864
1
3
1
0

0
2
1
1
5868
species

 0.00

1
1
1133968
strain

 0.00

order

 0.00
5819
4
1
9
0

family

 0.00
1639119
4
1
8
0

0
1
7
4
5820

 0.00
genus

418103
subgenus

 0.00
0
1
2
1

5858

 0.00
species
1
1


 0.00
subgenus
418101
3
1
1
0

5825

 0.00
species
0
2
1
1

1
1
31271

 0.00
subspecies

2
1
418107

 0.00
subgenus

1280412

 0.00
class
0
12
1
7

5796
subclass

 0.00
0
12
1
6

0
12
1
5
75739

 0.00
order

suborder

 0.00
423054
12
1
4
0

12
1
3
0
family

 0.00
5809

1
2
12
0

 0.00
genus
5810

5811
species

 0.00
12
1

clade

 0.00
554915
1
8
1
0

2605435

 0.00
phylum
0
1
1
7


 0.00
class
142796
6
1
1
0

33083

 0.00
clade
0
1
5
1

order

 0.00
2058949
1
4
1
0

family

 0.00
2058185
1
3
1
0

genus

 0.00
5782
1
2
1
0

species

 0.00
5786
1
1

0
26
1
13
2611352
clade

 0.00


 0.00
phylum
5752
6
6
1
0

clade

 0.00
2601529
6
5
1
0

clade

 0.00
2601530
6
1
4
0

6
1
3
0

 0.00
family
5765

6
2
1
0

 0.00
genus
5761

species

 0.00
5763
6
1

33682

 0.00
phylum
0
7
19
1

1
18
7
0

 0.00
class
5653

0
17
1
7
2704647

 0.00
subclass

2704949
order

 0.00
0
16
1
7

1
7
1
15
5654
family

 0.00

7
1
2
0
genus

 0.00
5690

subgenus

 0.00
47570
1
1
3
0

5693

 0.00
species
0
1
2
1

1
1
353153

 0.00
strain

47569

 0.00
subgenus
0
1
3
1

5692
species

 0.00
0
1
2
1

1068625

 0.00
strain
1
1

1286322
subfamily

 0.00
0
4
1
7

0
1
6
4
5658

 0.00
genus

0
1
4
3
38568
subgenus

 0.00

0
3
1
3
38582

 0.00
species group

5665

 0.00
species
0
2
1
3

3
1
strain

 0.00
929439


 0.00
subgenus
37616
1
1


 0.10
kingdom
33090
473
1
737
2

0
9
33
1
3041
phylum

 0.00

33103

 0.00
class
0
1
6
4

1
5
4
0
clade

 0.00
2546215

0
1
4
4
31306

 0.00
order

205394

 0.00
family
0
4
1
3


 0.00
genus
43940
4
1
2
0

43941
species

 0.00
1
4

0
1
1
5
2302911

 0.00
class

order

 0.00
2302912
1
4
1
0

0
1
3
1
2302913

 0.00
family

1
1
2
0
genus

 0.00
2302914

1
1
species

 0.00
1764295

0
4
1
21
2692248

 0.00
clade

5
1
1
0

 0.00
class
75966

75981
no rank

 0.00
0
1
1
4


 0.00
clade
2682734
3
1
1
0

1
2
1
0
genus

 0.00
41299


 0.00
species
41300
1
1

15
1
3
0
class

 0.00
3166

9
1
2
0
clade

 0.00
2812636

35491

 0.00
order
0
1
4
1

1
3
1
0

 0.00
family
1284389

0
1
1
2
50036

 0.00
genus

1
1
species

 0.00
50037

3042

 0.00
order
0
1
4
1

3
1
1
0
family

 0.00
3051

genus

 0.00
3052
1
1
2
0

1
1
species

 0.00
3055

1
5
1
0

 0.00
clade
2546211

1
4
1
0

 0.00
order
35490

0
1
3
1
2682485
family

 0.00

genus

 0.00
55993
1
2
1
0

1
1
321588

 0.00
species

726
439
1
0

 0.10
phylum
35493

1
438
726
0

 0.10
subphylum
131221

131209

 0.00
class
0
5
1
2


 0.00
subclass
2684882
4
1
2
0


 0.00
order
131210
2
3
1
0


 0.00
family
31314
1
2
2
0

genus

 0.00
33100
2
1

clade

 0.10
3193
1
432
724
3

1
414
706
0

 0.10
clade
58023

1
706
413
1
78536
clade

 0.10

clade

 0.10
58024
705
1
412
1

3
1
14
0
clade

 0.00
1437180

3
1
13
0
class

 0.00
58019

12
1
3
0

 0.00
subclass
3313

1
6
1
0
clade

 0.00
2821352

1446380
order

 0.00
0
5
1
1

family

 0.00
3318
1
4
1
0

0
3
1
1
3337

 0.00
genus

subgenus

 0.00
139271
1
1
2
0

3352

 0.00
species
1
1

0
2
1
5
2821351

 0.00
clade

0
4
1
2
1446379
order

 0.00

2
1
3
0
family

 0.00
3367

0
2
1
2
13414

 0.00
genus

1
2
species

 0.00
89191

397
1
701
1
class

 0.10
3398

0
4
1
1
261009

 0.00
order

1
1
3
0

 0.00
family
22097

13332

 0.00
genus
0
1
1
2

species

 0.00
13333
1
1

3
7
1
0
order

 0.00
261007

family

 0.00
4422
1
3
2
0

0
2
1
2
4423
genus

 0.00

1
2
4424

 0.00
species


 0.00
family
4410
1
1
3
0

1
1
2
0

 0.00
genus
4418

species

 0.00
210225
1
1

1
3
1
0
order

 0.00
82956

2
1
1
0

 0.00
family
16733

13673

 0.00
genus
1
1


 0.10
clade
1437183
382
1
695
26


 0.02
clade
4447
171
101
1
0

0
6
1
4
16360
order

 0.00

family

 0.00
4454
4
1
5
0

0
4
1
4
284551

 0.00
subfamily

0
3
1
4
4473
genus

 0.00

51605

 0.00
species
1
2


 0.00
species
29656
1
2

167
1
94
0

 0.02
subclass
1437197


 0.00
order
73496
7
1
6
0

4668

 0.00
family
0
7
5
1

0
7
4
1
40553
subfamily

 0.00

tribe

 0.00
703248
7
3
1
0

genus

 0.00
4678
1
2
7
1


 0.00
species
4679
6
1

5
1
5
0

 0.00
order
40548

0
4
1
5
4671
family

 0.00

4672
genus

 0.00
0
3
1
5


 0.00
species
29710
1
2
5
0

subspecies

 0.00
55577
5
1


 0.02
clade
4734
82
1
155
0

0
1
1
6
40551
order

 0.00

4710

 0.00
family
0
5
1
1

169700

 0.00
subfamily
0
4
1
1

1
3
1
0

 0.00
tribe
169748

1
2
1
0

 0.00
genus
4719

1
1
42345

 0.00
species

38820
order

 0.02
0
142
66
1

0
1
5
9
14101
family

 0.00

46322
genus

 0.00
0
2
1
6

1
6
59018

 0.00
species

0
2
1
3
13578
genus

 0.00

1
3
13579

 0.00
species

128
1
55
0

 0.02
family
4479


 0.00
clade
147370
15
22
1
0

147369

 0.00
subfamily
0
15
21
1

0
2
1
8
1648033

 0.00
no rank

1
7
2
0

 0.00
tribe
147429

1
3
1
0

 0.00
subtribe
1648028

genus

 0.00
4557
2
1
1
0

1
1
4558
species

 0.00

0
1
3
1
1648029
subtribe

 0.00

4575
genus

 0.00
0
1
1
2

1
1

 0.00
species
4577


 0.00
no rank
1648036
12
1
13
0

0
11
1
13
147428

 0.00
tribe

0
3
1
3
1293361

 0.00
subtribe

3
1
2
0

 0.00
genus
4554

1
3
4556
species

 0.00

subtribe

 0.00
1293360
1
3
6
0

2
1
6
0
genus

 0.00
66017

1
6
1010633

 0.00
species


 0.00
subtribe
1293365
4
4
1
0

3
1
4
0
genus

 0.00
4539

0
4
1
2
2100771
section

 0.00

species

 0.00
38727
1
4


 0.02
clade
359160
113
1
32
0

147368
subfamily

 0.01
1
97
24
1

89
1
12
0
no rank

 0.01
1648038

147389

 0.01
tribe
3
89
11
1

2
4
1
0

 0.00
subtribe
1648017

4512
genus

 0.00
0
3
1
2

2
1
2
1
species

 0.00
4513

1
1
subspecies

 0.00
112509

5
84
1
6
1648030

 0.01
subtribe

2
1
1
0
genus

 0.00
4480

1
1

 0.00
species
37682

genus

 0.01
4564
78
3
1
26

1
1

 0.00
species
85692

51
1
4565

 0.01
species

1648037

 0.00
no rank
0
7
11
1

147387
tribe

 0.00
0
7
1
10

2
1
5
0
clade

 0.00
1652081

2
1
4
0
clade

 0.00
2948571

0
2
3
1
640630
subtribe

 0.00

0
2
1
2
4520

 0.00
genus

species

 0.00
4522
2
1

0
5
4
1
1652080
clade

 0.00

0
5
3
1
640623

 0.00
subtribe

genus

 0.00
4496
5
2
1
0

1
5
4498
species

 0.00

0
16
1
7
147367

 0.00
subfamily


 0.00
tribe
147380
16
1
6
0

1648021

 0.00
subtribe
0
1
5
16

4527

 0.00
genus
0
4
1
16

4530
species

 0.00
8
1
2
10

39947
no rank

 0.00
1
2

1
6
4533

 0.00
species

5
1
5
0
family

 0.00
4613

subfamily

 0.00
1909378
4
1
5
0


 0.00
genus
4614
5
1
3
0

5
2
1
2

 0.00
species
4615

1
3
296719

 0.00
varietas

0
12
1
9
4618

 0.00
order

4642
family

 0.00
0
1
3
1

0
2
1
1
4650
genus

 0.00


 0.00
species
94328
1
1

0
11
5
1
4637

 0.00
family

4640
genus

 0.00
1
1
4
11


 0.00
species
4641
9
2
1
0

9
1
subspecies

 0.00
214687

species

 0.00
320322
1
1

71240
clade

 0.07
0
486
1
276

486
1
275
0

 0.07
clade
91827

274
1
486
46

 0.07
clade
1437201

71275
clade

 0.04
17
1
168
280

91836
clade

 0.02
8
1
61
115

0
1
13
17
41938

 0.00
order

family

 0.00
3629
12
1
17
0

14
8
1
0

 0.00
subfamily
214907

3633
genus

 0.00
4
12
1
5

2
1

 0.00
species
3635

34274
species

 0.00
1
1

species

 0.00
29730
1
1

1
4
47622
species

 0.00

0
2
2
1
47614

 0.00
genus

2
1
47615
species

 0.00

214909
subfamily

 0.00
0
3
3
1


 0.00
genus
3640
1
2
3
0

3
1
species

 0.00
3641


 0.01
order
41937
17
1
58
1

1
3
16
0
family

 0.00
23808

0
2
1
16
23809

 0.00
genus

1
16
species

 0.00
2768810

1
3
1
0
family

 0.00
43707

0
1
1
2
43708

 0.00
genus

1
1
155640

 0.00
species

6
1
38
0

 0.01
family
23513

0
1
5
38
1728959

 0.01
subfamily


 0.01
genus
2706
38
1
4
22

1
1
species

 0.00
2709

1
4
85681

 0.00
species

11
1
2711
species

 0.00

family

 0.00
4011
1
4
2
0

23461
genus

 0.00
1
1

1
2
1
0

 0.00
genus
55512

1
1
species

 0.00
55513

0
27
1
21
3699

 0.00
order

0
27
1
20
3700
family

 0.00


 0.00
tribe
981070
3
1
3
0

50451
genus

 0.00
0
3
1
2

50452

 0.00
species
1
3

tribe

 0.00
980083
8
1
7
0


 0.00
genus
71323
3
1
3
0

1
1

 0.00
species
90675

2
1

 0.00
species
883000

3
1
5
0

 0.00
genus
3701

2
1
3702
species

 0.00


 0.00
species
38785
3
1

981071

 0.00
tribe
0
12
6
1

10
3
1
7
genus

 0.00
3705

1
1
species

 0.00
3711

2
1
3712
species

 0.00

0
2
1
2
3725

 0.00
genus

3726
species

 0.00
1
2


 0.00
tribe
981099
4
1
3
0

4
1
2
0
genus

 0.00
13287

4
1
13288

 0.00
species

41944
order

 0.00
0
1
9
5

1
3
1
0
family

 0.00
3928

22662
genus

 0.00
0
1
2
1

22663
species

 0.00
1
1

0
4
5
1
3934
family

 0.00

1585427

 0.00
subfamily
0
1
4
4


 0.00
tribe
1585433
4
3
1
0

238243

 0.00
genus
0
4
2
1

4
1
species

 0.00
13055

142
99
1
4
clade

 0.02
91835


 0.00
order
3744
1
21
12
1

1
20
11
0
family

 0.00
3745

171637
subfamily

 0.00
0
7
1
5

1
3
1
0

 0.00
tribe
721805

1
1
2
0
genus

 0.00
3754


 0.00
species
3755
1
1

0
4
1
3
721813
tribe

 0.00

genus

 0.00
3749
2
1
4
3

3750
species

 0.00
1
1

0
6
1
12
171638
subfamily

 0.00

1
1
3
0

 0.00
no rank
1176516

3764
genus

 0.00
0
1
2
1

species

 0.00
74649
1
1


 0.00
tribe
721789
1
1
5
0

1184124

 0.00
subtribe
0
1
1
4


 0.00
genus
3746
1
3
1
0

57918
species

 0.00
0
2
1
1

101020

 0.00
subspecies
1
1

1
3
4
0

 0.00
tribe
721790

2
1
4
0

 0.00
genus
3761

57919
species

 0.00
4
1

3646

 0.00
order
0
12
1
3


 0.00
family
3977
3
11
1
0

1
6
2
0

 0.00
subfamily
235629


 0.00
tribe
235880
5
1
2
0

0
1
2
1
3987

 0.00
genus

1
1

 0.00
species
3988

0
1
2
1
3984
genus

 0.00


 0.00
species
3986
1
1

4
1
1
0
subfamily

 0.00
235631


 0.00
tribe
235883
1
1
3
0

0
1
2
1
3982

 0.00
genus


 0.00
species
3983
1
1

42
1
75
0

 0.01
order
72025

3803

 0.01
family
0
1
41
75

subfamily

 0.01
3814
1
40
75
0

39
1
75
0

 0.01
clade
2231393

0
27
1
30
2231382
clade

 0.00

clade

 0.00
2233855
1
11
11
0

163735
tribe

 0.00
0
1
10
11


 0.00
genus
3820
2
1
1
0

1
1
species

 0.00
3821

3913

 0.00
genus
0
1
4
9


 0.00
species
3914
5
1
2
0

1
5

 0.00
varietas
157739

3917

 0.00
species
1
4

3
1
1
0

 0.00
genus
3846


 0.00
subgenus
1462606
1
2
1
0

1
1
3847

 0.00
species

0
15
1
19
2233838
clade

 0.00

16
1
10
0
clade

 0.00
2233839

0
10
1
4
163742
tribe

 0.00

3877

 0.00
genus
0
10
3
1

1
7
70936

 0.00
species

3880

 0.00
species
1
3

0
1
2
1
163743

 0.00
tribe

3863

 0.00
genus
1
1

0
3
1
5
163722

 0.00
tribe

0
5
1
2
3826
genus

 0.00

3827

 0.00
species
5
1

2233857

 0.00
clade
0
3
1
4


 0.00
tribe
163747
3
1
3
0

0
2
1
3
3867

 0.00
genus


 0.00
species
34305
3
1

0
3
1
5
2231384
clade

 0.00

clade

 0.00
2231385
4
1
3
0

163729
tribe

 0.00
0
1
3
3

3
1
2
0
genus

 0.00
3869

species

 0.00
3871
1
3


 0.01
clade
2231387
6
1
42
0

163725

 0.01
tribe
0
5
1
42

2231390

 0.01
clade
6
42
1
4

1
3
36
4

 0.01
genus
3817

1
1
281009
species

 0.00

species

 0.00
3818
31
1

0
1
11
21
71239

 0.00
order

1
10
1
21
3650
family

 0.00

0
19
5
1
1003877
tribe

 0.00


 0.00
genus
3655
10
1
2
1

3656
species

 0.00
1
9

1
2
9
0

 0.00
genus
102210

1
9
species

 0.00
102211

1003878
tribe

 0.00
0
1
4
1

3660

 0.00
genus
0
1
3
1


 0.00
species
3663
2
1
1
0

1
1

 0.00
subspecies
3664

1
12
1
27
3502

 0.00
order

0
9
1
5
3514
family

 0.00

0
1
2
7
13450

 0.00
genus

13451
species

 0.00
1
7

0
2
1
2
12989

 0.00
genus

species

 0.00
176864
2
1

0
6
1
17
3503

 0.00
family

0
15
1
3
3511
genus

 0.00

14
1

 0.00
species
38942

1
1
species

 0.00
97700

21024

 0.00
genus
0
2
1
2

2
1
28930
species

 0.00

91834
no rank

 0.00
0
1
7
6

0
6
6
1
403667

 0.00
order

3602

 0.00
family
0
1
5
6

6
1
4
0
tribe

 0.00
2304100


 0.00
genus
3603
3
1
6
2

103349

 0.00
species
2
1

1
2

 0.00
species
96939

4
138
1
91
71274
clade

 0.02

0
1
57
101
91888

 0.01
clade

order

 0.01
4069
23
1
80
0

4118
family

 0.00
0
4
1
8

267213
tribe

 0.00
0
8
3
1


 0.00
genus
4119
8
1
2
3

1
5

 0.00
species
35884


 0.01
family
4070
72
18
1
0

0
1
4
1
424554
subfamily

 0.00

0
3
1
1
424562
tribe

 0.00

1
2
1
0
genus

 0.00
4085

1
1

 0.00
species
4098

0
71
13
1
424551

 0.01
subfamily

tribe

 0.00
424564
6
1
3
0

2
2
1
6
4071
genus

 0.00

4072

 0.00
species
1
4

424574
tribe

 0.01
0
65
9
1

4107

 0.01
genus
14
8
1
65

33
1
species

 0.00
4113

13
3
1
2
subgenus

 0.00
49274

1
2
28526
species

 0.00

9
1
species

 0.00
4081

1
1
50273

 0.00
species

1
1

 0.00
species
315347

3
1
45834

 0.00
species

0
4
8
1
4055

 0.00
order


 0.00
family
24966
7
1
4
0

169618

 0.00
subfamily
0
1
6
4

1968429
clade

 0.00
0
5
1
4

4
4
1
0

 0.00
clade
1968428

169640

 0.00
tribe
0
4
1
3

0
2
1
4
13442

 0.00
genus

49369
species

 0.00
1
4

4143
order

 0.00
1
17
1
25

4180
family

 0.00
0
1
3
1

0
1
1
2
4181

 0.00
genus

1
1

 0.00
species
4182

0
9
1
9
4144
family

 0.00

426105

 0.00
tribe
0
2
3
1

0
2
1
2
4147
genus

 0.00

1
2
species

 0.00
660624

426106
tribe

 0.00
0
5
1
7

genus

 0.00
126428
1
2
1
0

1
1
126429
species

 0.00


 0.00
genus
38871
6
1
2
0

56036

 0.00
species
6
1

family

 0.00
156152
4
1
1
0

tribe

 0.00
216780
1
1
3
0

102598

 0.00
genus
0
1
2
1


 0.00
species
102599
1
1

4136

 0.00
family
0
8
1
5

216702
subfamily

 0.00
0
1
3
2

4139
genus

 0.00
0
2
1
2

1
2
53169

 0.00
species

0
3
4
1
216703
subfamily

 0.00

1
3
3
0
tribe

 0.00
983543

0
1
2
3
155228
genus

 0.00

1
3
species

 0.00
194200

0
11
8
1
41945
order

 0.00


 0.00
family
27065
1
4
7
0

4441
genus

 0.00
1
7
3
1

5
1
4442
species

 0.00

1
1
species

 0.00
385388

0
1
3
4
25692

 0.00
family

2
1
4
0

 0.00
genus
35939

253017
species

 0.00
4
1

0
22
1
25
91882
clade

 0.00

13
1
19
0

 0.00
order
4036

suborder

 0.00
364270
19
1
12
0

0
13
8
1
4037
family

 0.00

241778

 0.00
subfamily
1
7
1
13

12
1
6
0

 0.00
tribe
241789

241799

 0.00
subtribe
0
5
1
12

2
4
1
12
4038

 0.00
genus

1873447

 0.00
section
0
1
3
10

species

 0.00
4039
10
1
2
0

1
10
79200
subspecies

 0.00

6
3
1
0
family

 0.00
4050

0
6
2
1
4051
genus

 0.00


 0.00
species
4052
1
6

3
11
1
0

 0.00
order
4209

0
1
10
3
4210

 0.00
family

3
9
1
0

 0.00
subfamily
102804

102806

 0.00
tribe
0
4
1
1

911294
subtribe

 0.00
0
1
3
1


 0.00
genus
56534
1
1
2
0

species

 0.00
56535
1
1

1
4
2
0
clade

 0.00
911341

1
3
2
0

 0.00
tribe
102814

2
1
2
0
genus

 0.00
4231

1
2
species

 0.00
4232

0
22
14
1
3524

 0.00
order

0
8
1
20
1804623

 0.00
family

7
1
20
0
subfamily

 0.00
1307796

1307775

 0.00
tribe
0
1
1
3

genus

 0.00
3561
2
1
1
0

3562
species

 0.00
1
1

0
19
1
3
1307774
tribe

 0.00

19
1
2
0
genus

 0.00
3558

1
19
63459

 0.00
species

3615
family

 0.00
0
5
1
2

0
1
4
2
1110380

 0.00
subfamily

2
3
1
0
tribe

 0.00
1110385

1
2
2
0
genus

 0.00
46786

2
1

 0.00
species
137693

12
1
4
1

 0.00
order
232378

4401

 0.00
family
0
11
3
1

10
1
2
11
4402

 0.00
genus

1
1
species

 0.00
140101

3208
clade

 0.00
0
1
1
9

404260
clade

 0.00
0
1
1
8


 0.00
class
3214
1
7
1
0

6
1
1
0
subclass

 0.00
114658

superorder

 0.00
404315
5
1
1
0


 0.00
order
13798
4
1
1
0

0
1
1
3
28466
family

 0.00

1
2
1
0
genus

 0.00
28467

28468

 0.00
species
1
1

0
14
8
1
3195
clade

 0.00

14
7
1
0

 0.00
class
186770

6
1
14
0
subclass

 0.00
186774

1
5
14
0
order

 0.00
28908

0
1
4
14
29585

 0.00
family

3196

 0.00
genus
0
14
1
3

0
2
1
14
3197

 0.00
species

1
14
1480154
subspecies

 0.00

3027

 0.00
class
0
5
1
10

589342
order

 0.00
0
5
1
2

0
1
4
2
589343
family

 0.00

55528
genus

 0.00
0
2
1
3

species

 0.00
55529
2
1
2
0

905079
strain

 0.00
2
1


 0.00
order
589350
3
1
4
0


 0.00
family
2896
3
1
3
0


 0.00
genus
3030
3
2
1
2

1
1
species

 0.00
233186

1
577718

 80.46

2
superkingdom

 4.84
1054
2040
1
34572

196
1
1663
0

 0.25
clade
1783270

1
6
1
2
142182

 0.00
phylum

0
1
1
5
219685

 0.00
class

219686
order

 0.00
0
4
1
1

family

 0.00
219687
3
1
1
0

0
1
1
2
173479
genus

 0.00

1
1
1379270
species

 0.00

clade

 0.25
68336
1661
1
189
0

1660
1
185
38

 0.25
phylum
976

0
1
14
22
117747
class

 0.00


 0.00
order
200666
13
1
22
0

84566
family

 0.00
3
22
1
12


 0.00
genus
84567
12
7
1
5

2766984

 0.00
species
1
1

363852

 0.00
species
1
1

2628915
no rank

 0.00
0
5
1
4

1
1
2578106
species

 0.00

1
1
2482728

 0.00
species


 0.00
species
2856523
3
1

genus

 0.00
28453
7
4
1
3

species

 0.00
2886510
1
2


 0.00
species
371142
1
1

28454
species

 0.00
1
1

9
8
1
0
class

 0.00
1853228

9
1
7
0
order

 0.00
1853229

6
1
9
2

 0.00
family
563835


 0.00
genus
1860196
1
1
2
0

661488

 0.00
species
1
1

79328
genus

 0.00
2
6
1
3

1
3

 0.00
species
2725414

1
1
no rank

 0.00
2619133

200643

 0.02
class
0
43
1
160

order

 0.02
171549
160
42
1
11

1
3
1
0
family

 0.00
2005520

307628
genus

 0.00
0
1
2
1

1
1
species

 0.00
1642646

3
1
9
14
815
family

 0.00

6
1
5
3
genus

 0.00
816

1
1
species

 0.00
28113

species

 0.00
371601
2
1
1
0

1
1
657309
strain

 0.00

818

 0.00
species
1
1

909656
genus

 0.00
2
5
1
3

1
1
387090

 0.00
species

357276

 0.00
species
2
1

2005525
family

 0.00
0
5
1
5

2
1
2
0
genus

 0.00
375288


 0.00
species
823
2
1

195950

 0.00
genus
0
1
2
3

1
3
712710

 0.00
species

1
125
16
1
171552

 0.02
family

2974257
1
4
genus


 0.00
species
28127
1
4

1
14
120
13

 0.02
genus
838

1
20
28129
species

 0.00

28135

 0.00
species
7
1

species

 0.00
470565
1
5

28133
species

 0.00
1
2

6
3
1
0
no rank

 0.00
2638335

652716

 0.00
species
0
6
2
1

575614
strain

 0.00
1
6

1
3

 0.00
species
28131

5
1
species

 0.00
1177574

589437
species

 0.00
0
1
2
1

1
1
strain

 0.00
1236518

1
1
282402

 0.00
species

28132

 0.01
species
57
1

6
1
3
0
family

 0.00
171551

0
3
5
1
836
genus

 0.00

1
1
species

 0.00
28124

1
2
1
0
species

 0.00
28123

879243
strain

 0.00
1
1

1
1
322095
species

 0.00

1
2
1
0
family

 0.00
171550

1
1
239759

 0.00
genus

class

 0.02
768503
126
30
1
0

1
29
126
1

 0.02
order
768507

0
79
1
15
1853232
family

 0.01

genus

 0.01
89966
1
14
79
34

22
38
8
1
2615202

 0.01
no rank

1
1
2584940

 0.00
species

2761579

 0.00
species
1
1

2
1
species

 0.00
2932254

1
1
species

 0.00
2714932


 0.00
species
1356852
3
1

1
6

 0.00
species
2932250

2835648
species

 0.00
1
2

1
1

 0.00
species
1484116

0
1
2
1
1446467
species

 0.00

strain

 0.00
1227739
1
1

1705399
species

 0.00
4
1


 0.00
species
497967
1
1

1
1
4
2
2896860
family

 0.00

0
1
1
3
120831
genus

 0.00

2625061

 0.00
no rank
0
1
1
2

1
1
2861765
species

 0.00

44
9
1
0

 0.01
family
89373

8
39
3
1
2676247
genus

 0.01

1
11
2696560
species

 0.00

2516559
species

 0.00
1
20

2
1
5
5
107
genus

 0.00

1
1
species

 0.00
2057025

1
1
species

 0.00
564064


 0.00
species
108
1
2
1
0


 0.00
strain
504472
1
1

1305
89
1
0
class

 0.20
117743

order

 0.20
200644
1
88
1305
11

1
1
2
0
no rank

 0.00
403978

2021391

 0.00
species
1
1

1853230

 0.00
family
0
1
1
4

1
1
3
0
genus

 0.00
332102

species

 0.00
191579
2
1
1
0


 0.00
strain
755732
1
1

1
1
3
0
no rank

 0.00
313602

2
1
1
0

 0.00
genus
336809


 0.00
species
336810
1
1


 0.02
family
2762318
173
32
1
7

308865
genus

 0.00
2
8
1
3

species

 0.00
1756149
1
5

1
1
species

 0.00
1117645

0
2
1
1
1013
genus

 0.00

1
1
1014

 0.00
species

2782232

 0.01
no rank
11
78
19
1

1
4
1
8
2782231

 0.00
genus


 0.00
species
2820270
1
1

421525

 0.00
species
1
4

266749

 0.00
species
2
1


 0.00
genus
2782229
1
3
33
0


 0.00
species
2487072
32
1

1
1

 0.00
species
1416779

genus

 0.00
59732
11
1
26
6

1
7
2754694
species

 0.00

1
1

 0.00
species
1493872

1
3
3
1
2593645
no rank

 0.00


 0.00
species
2039166
1
1

1
1
2864040

 0.00
species

species

 0.00
254
1
1

1
1
species

 0.00
253

4
1
2929799

 0.00
species

2
1
536441

 0.00
species

1
1
246
species

 0.00


 0.00
genus
1433995
1
2
1
0


 0.00
species
1118202
1
1

0
2
1
20
59734

 0.00
genus

1
20

 0.00
species
343874

3
1
58
11

 0.01
genus
501783

237258
species

 0.00
1
13

2004710
species

 0.00
1
34

61
46
1
1118
49546

 0.18
family

2
3
1
0

 0.00
genus
252356

2
1
2
0

 0.00
no rank
2615042

1644130

 0.00
species
2
1

1012
22
1
104

 0.16
genus
237

1
1
96345

 0.00
species

species

 0.00
1355330
1
1

1
252
species

 0.04
1751056

1763534
species

 0.00
1
1

1
1
species

 0.00
2704140

1617283
species

 0.00
1
1

1
1
2
0
species

 0.00
986

376686
strain

 0.00
1
1

species

 0.00
55197
1
2
1
0

1
1
strain

 0.00
1034807

1
1
2895947

 0.00
species

2816357
species

 0.00
1
1

1871076
species

 0.00
1
1

1
1
1492737
species

 0.00

293
1
species

 0.04
1751095

6
1
352
347
no rank

 0.15
196869

1
1
1979344

 0.00
species

1
1

 0.00
species
2937442


 0.00
species
2893886
1
1

1
1
2893883
species

 0.00

1
1
239

 0.00
species

genus

 0.00
143222
2
1

52959
genus

 0.00
0
2
1
1

species

 0.00
2738844
1
1

0
1
1
2
104267

 0.00
genus


 0.00
species
584609
1
1

0
1
3
1
2045416

 0.00
genus

2631961
no rank

 0.00
0
1
2
1

1
1
2529032

 0.00
species

genus

 0.00
76831
4
1
4
0

species

 0.00
76832
1
1


 0.00
species
256
2
1

1
1
702745

 0.00
species

34
1
8
7
genus

 0.00
1016

1
11

 0.00
species
1019

6
1

 0.00
species
1017

3
9
1
4
2640652
no rank

 0.00

1
1
2545799

 0.00
species

species

 0.00
1705617
1
1

4
1
2748316

 0.00
species

1
1
327575

 0.00
species


 0.00
phylum
1090
1
1
3
0


 0.00
no rank
44765
2
1
1
0

1
1
2268192

 0.00
species

49
1
34
0
clade

 0.00
1783257

phylum

 0.00
204428
1
1
7
0

0
1
6
1
204429
class

 0.00

1963360

 0.00
order
0
1
5
1

0
1
4
1
92713
family

 0.00

0
1
3
1
83551

 0.00
genus

1
2
1
0
species

 0.00
83552

1
1
strain

 0.00
765952

phylum

 0.00
74201
10
1
4
1

0
1
2
1
417295
no rank

 0.00

2026799
species

 0.00
1
1

0
2
1
7
203494
class

 0.00

2
6
1
0

 0.00
order
48461

0
1
1
2
1647988

 0.00
family


 0.00
genus
239934
1
1

1
3
1
0
family

 0.00
203557


 0.00
genus
518753
1
2
1
0

2824561
species

 0.00
1
1

phylum

 0.00
203682
29
1
31
1

4
7
1
1
class

 0.00
666505


 0.00
order
666506
3
1
6
0

2
1
1
0

 0.00
no rank
1500946

1
1

 0.00
species
2052180

666507

 0.00
family
0
2
1
3

1385974

 0.00
no rank
0
1
2
2

1
2
2026777
species

 0.00

class

 0.00
203683
24
23
1
3

2691356
order

 0.00
0
1
4
1


 0.00
family
1763524
1
3
1
0

0
2
1
1
2680020
genus

 0.00

1
1
2527974
species

 0.00


 0.00
order
2691354
2
1
7
0

1
6
2
0

 0.00
family
2691357

genus

 0.00
123
1
1
3
0

0
1
2
1
2639138
no rank

 0.00

species

 0.00
1632865
1
1

genus

 0.00
2807414
1
2
1
0

species

 0.00
2528021
1
1

11
1
18
0
order

 0.00
2691355


 0.00
family
1914233
1
10
18
11

2731450
genus

 0.00
0
1
2
1

2598579
species

 0.00
1
1

2807415
genus

 0.00
0
2
1
2

2528023

 0.00
species
1
2

2774146

 0.00
genus
0
2
1
2

1
2
species

 0.00
2774151

113

 0.00
genus
1
1


 0.00
no rank
2052163
1
2
1
0

1
1
species

 0.00
2052164

2323
no rank

 0.00
0
1
16
13

2
13
1
15
1783234

 0.00
clade

2
6
1
5
95818

 0.00
phylum


 0.00
no rank
1895827
4
1
4
0

713051

 0.00
species
1
2

1
1
713059

 0.00
species

1619070
species

 0.00
1
1

1783273
clade

 0.00
0
1
9
5


 0.00
clade
1794811
4
5
1
1

1752727

 0.00
phylum
0
1
1
2

1
1
1618633
species

 0.00

1752729
phylum

 0.00
0
2
1
2

2052152

 0.00
species
1
2

3
1
1
0
clade

 0.00
1794810

0
2
1
1
1752723

 0.00
phylum

1
1

 0.00
species
2282149

341
1
860
17825
1224
phylum

 2.48

738
187
1
33
class

 0.10
28216


 0.00
order
32003
4
1
8
0

32011
family

 0.00
0
4
1
7

2
1
3
0

 0.00
genus
404

2
1
2
0

 0.00
species
405

1
2

 0.00
strain
265072

16

 0.00
genus
0
1
1
2

2588534
species

 0.00
1
1

81682
genus

 0.00
1
1

6
1
4
0

 0.00
no rank
119066

0
6
1
3
327159
genus

 0.00

2954388
species

 0.00
1
5

1
1
2954383

 0.00
species

206389

 0.00
order
1
9
1
11

0
3
4
1
75787

 0.00
family

3
1
3
2

 0.00
genus
146937


 0.00
species
146939
2
1
1
0

1
1
640081

 0.00
strain

4
3
1
0

 0.00
family
2808923

0
4
2
1
2808942
genus

 0.00

1751046

 0.00
species
1
4

family

 0.00
2008795
1
3
1
0

1
1
2
0

 0.00
genus
73029

1
1
species

 0.00
281362

order

 0.08
80840
588
134
1
97

0
1
12
36
506

 0.01
family

1
2
1
0

 0.00
genus
517

1
1
species

 0.00
123899

222

 0.00
genus
4
7
1
3

2
1
1353891

 0.00
species

1
1

 0.00
species
85698

3
1
8
5
genus

 0.00
507

2
1
species

 0.00
511

species

 0.00
323284
1
1

90243
genus

 0.00
0
3
1
20

90244
species

 0.00
1
1

1
19
90245

 0.00
species

family

 0.03
80864
198
1
48
46

6
1
19
9

 0.00
genus
34072

1
2
436515
species

 0.00

663243
no rank

 0.00
0
2
3
1

species

 0.00
2762322
1
1

2774875
species

 0.00
1
1

species

 0.00
34073
6
1

2
1
47420

 0.00
genus

5
3
1
1
genus

 0.00
28065

4
1
2
1

 0.00
no rank
2627954

3
1

 0.00
species
2752316

genus

 0.00
665874
10
1
4
0

2626134

 0.00
no rank
1
10
1
3

1678129
species

 0.00
2
1

1678128

 0.00
species
7
1

3
4
1
0

 0.00
genus
281915

1
2
2
0
no rank

 0.00
2685271

1
2
species

 0.00
1844971


 0.00
species
667019
1
1

2
1
6
10
283

 0.00
genus

225992
species

 0.00
1
2

225991

 0.00
species
1
1

1
1
363952
species

 0.00

species

 0.00
285
1
2

1
2
379895
species

 0.00

0
1
3
1
683756

 0.00
genus

0
1
1
2
2632542

 0.00
no rank

1
1

 0.00
species
2714952

genus

 0.00
219181
2
1
2
0


 0.00
species
2109914
2
1

1
7
12
1

 0.00
genus
12916

1
1

 0.00
species
2743470

no rank

 0.00
2684926
1
4
9
2

species

 0.00
2518343
1
2

species

 0.00
358220
1
4

232721

 0.00
species
1
1

1
1
species

 0.00
553814

0
5
4
1
238749
genus

 0.00

1
2
1288495

 0.00
species

2649760

 0.00
no rank
0
3
2
1

1
3
species

 0.00
2714924

0
2
1
72
83494

 0.01
no rank

2952996
species

 0.01
72
1

1
1
174951

 0.00
genus

10
4
1
6

 0.00
genus
80865

2
1
2
0

 0.00
no rank
2613839

2
1
species

 0.00
1920191

2
1

 0.00
species
80866


 0.01
family
75682
19
1
81
14

0
1
2
1
401469

 0.00
genus

1
1

 0.00
no rank
2630295


 0.01
no rank
2895353
1
10
58
11

2
2
1
0
genus

 0.00
75654

2
1
2728021

 0.00
species

149698
genus

 0.01
30
6
1
43

1141883

 0.00
species
1
1

2609279

 0.00
no rank
8
3
1
11

2
1
species

 0.00
1707785

1
1

 0.00
species
2861282

1
1

 0.00
species
2728020

1522432
genus

 0.00
2
1

1
1
genus

 0.00
963


 0.00
genus
29580
5
1
7
4

29581

 0.00
species
1
1

0
1
2
1
2610881
no rank

 0.00

1537274

 0.00
species
1
1

1
1

 0.00
species
2590869

13
1
14
4

 0.00
family
2975441

0
1
2
3
391952
genus

 0.00

391953

 0.00
species
3
1

1
1
2
0

 0.00
genus
644355

1
1
species

 0.00
392593

3
4
1
0

 0.00
genus
34102

2
1
639200

 0.00
species

1
2
1
0
no rank

 0.00
2628234

1
1
2914710

 0.00
species

0
1
2
1
316612

 0.00
genus

1
1
no rank

 0.00
2633235

genus

 0.00
28067
1
3
1
0

28068

 0.00
species
0
1
2
1

983917

 0.00
strain
1
1

family

 0.02
119060
158
35
1
2

44013
genus

 0.02
25
109
1
17

24
1
9
62
2640945

 0.01
no rank

1
1
2576926

 0.00
species

2576930
species

 0.00
2
1

1855880
species

 0.00
3
1


 0.00
species
2770234
26
1

1
2
species

 0.00
1855619

species

 0.00
2576928
1
1


 0.00
species
2689107
1
2

2081049
species

 0.00
1
1

2
1

 0.00
species
576610

species

 0.00
2527775
1
1

556054
species

 0.00
10
1

1819725
species

 0.00
1
1

1
1
1835254

 0.00
species

1
1
2081042

 0.00
species

576611
species

 0.00
6
1

5
1
17
9
genus

 0.00
32008

1
2
7
5

 0.00
species group
87882

2
1

 0.00
species
488447

0
2
1
1
111527

 0.00
species group

1
1

 0.00
species
28450


 0.00
genus
47670
1
2
15
0

47671

 0.00
species
1
15

1
1
2
2
106589

 0.00
genus

119219
species

 0.00
1
1

5
5
1
9
48736

 0.00
genus

no rank

 0.00
209769
2
1
1
0

1
1
species

 0.00
54061

329
species

 0.00
2
1

1
1

 0.00
species
305

1
1
3
4
1822464

 0.00
genus

1
2
134537

 0.00
species

1
1
species

 0.00
2026199

5
1
5
1
no rank

 0.00
224471

92793
genus

 0.00
0
3
1
2


 0.00
species
1296669
3
1

genus

 0.00
114248
1
1
2
0

1
1
307486
species

 0.00

98
1
29
0

 0.01
order
206351

1
4
1
0
family

 0.00
1499392

187

 0.00
genus
0
1
1
3

no rank

 0.00
2620219
1
1
2
0

1
1
species

 0.00
1938604

481

 0.01
family
13
24
1
97

genus

 0.00
212742
2
1
1
0

1
1
1056807
species

 0.00

genus

 0.00
32257
6
1
3
1

4
1
species

 0.00
505

1
1
502

 0.00
species

1
15
75
29
genus

 0.01
482

species

 0.00
488
1
10

2
1
8
6

 0.00
species
495

2
1
88719
subspecies

 0.00

1
1
species

 0.00
267212

2
1
3
0
species

 0.00
496


 0.00
strain
997348
3
1

0
5
3
1
2623750
no rank

 0.00

641148
species

 0.00
0
2
1
5


 0.00
strain
641149
1
5

1
1

 0.00
species
487

28449

 0.00
species
1
11

species

 0.00
483
1
1


 0.00
species
486
1
1

1
5
490

 0.00
species

0
2
3
1
538

 0.00
genus

1
1

 0.00
species
2528037

539

 0.00
species
1
1

68525
subphylum

 0.01
0
60
46
1

29547

 0.00
class
0
23
1
24


 0.00
order
213849
22
1
24
0

6
1
7
2

 0.00
family
2808963

0
4
6
1
28196

 0.00
genus

1
1
1054033
species

 0.00


 0.00
species
1278212
2
1
2
0

1
2
strain

 0.00
663365

2593671

 0.00
no rank
0
1
1
2

944547
species

 0.00
1
1

2932623
family

 0.00
0
3
1
1

57665

 0.00
genus
0
1
2
1

366522
species

 0.00
1
1

72294

 0.00
family
0
16
8
1

0
16
1
7
194

 0.00
genus

0
1
2
1
827
species

 0.00

1
1

 0.00
strain
1032069

1
9

 0.00
species
199


 0.00
no rank
2593542
1
2
1
0

2517362
species

 0.00
1
1

species

 0.00
824
5
1

1
3
1
0
family

 0.00
72293

1
1
2
0
genus

 0.00
209

1
1
species

 0.00
76936

1
36
22
1
28221
class

 0.01

5
1
1
0

 0.00
order
69541

family

 0.00
213422
1
4
1
0

0
1
1
3
28231

 0.00
genus

0
1
2
1
2627627
no rank

 0.00


 0.00
species
1277350
1
1

29

 0.00
order
1
1
12
33

80811

 0.00
suborder
1
1
7
13

11
1
4
0
family

 0.00
39

2
11
3
1
47

 0.00
genus

1
2
83451
species

 0.00

7
1
species

 0.00
48

1524215
family

 0.00
0
1
2
1

1
1
161492
genus

 0.00

2
1
4
19
80812

 0.00
suborder

0
17
1
3
49

 0.00
family

genus

 0.00
1882917
17
1
2
0

species

 0.00
1882918
1
17

213115
order

 0.00
0
4
1
1

194924

 0.00
family
0
1
1
3

872
genus

 0.00
0
1
2
1

1
1
901

 0.00
species


 0.69
class
1236
4937
1
371
40

1
10
5
0

 0.00
order
135619

0
5
1
9
28256

 0.00
family

204286
genus

 0.00
0
1
1
3

no rank

 0.00
2609414
1
2
1
0

1
1
2758724
species

 0.00

0
1
3
1
42054
genus

 0.00

0
1
2
1
158080

 0.00
species

290398

 0.00
strain
1
1

0
3
2
1
2745

 0.00
genus

1
3
2609666
no rank

 0.00

order

 0.19
91347
106
1
1339
113

0
3
1
7
1903414

 0.00
family

581
genus

 0.00
0
1
2
1

582
species

 0.00
1
1


 0.00
genus
586
1
2
1
0

1
1
587
species

 0.00

genus

 0.00
583
1
2
1
0

1
1
585

 0.00
species

1903409
family

 0.00
11
35
1
16

genus

 0.00
2100764
5
1
4
0

species

 0.00
1615494
1
1

1
1
665913

 0.00
species

0
2
2
1
2628878

 0.00
no rank

2
1
2872648

 0.00
species

1
1
2
0
genus

 0.00
551

1
1
55212

 0.00
species

53335
genus

 0.00
11
1
8
19

2630326

 0.00
no rank
0
1
4
4

1
1
2575375
species

 0.00

1
1
species

 0.00
2886821

2
1
species

 0.00
2970818

66269

 0.00
species
1
1

2
1
3
0
species group

 0.00
1654067

549
species

 0.00
1
3

1903411
family

 0.12
14
829
1
32


 0.00
genus
41201
1
2
1
0

1
1
41202

 0.00
species

482
1
14
802
613
genus

 0.11

1
11

 0.00
species
82996

615
species

 0.00
4
1


 0.00
species
47917
1
2

614

 0.00
species
9
14
1
2

strain

 0.00
1346614
5
1

28151
species

 0.00
6
1

2647522

 0.00
no rank
0
6
1
4

1
2
species

 0.00
2447890

2448483

 0.00
species
3
1

1
1
species

 0.00
2697369

253
1
species

 0.04
82995

137545
species

 0.00
4
1

1
20

 0.00
species
42906

1
2
3
0
genus

 0.00
1565532

1646377

 0.00
species
3
1

1964366
genus

 0.00
0
1
3
1

0
2
1
1
2636213

 0.00
no rank

1
1
2126321
species

 0.00


 0.00
genus
629
5
1
2
0

species

 0.00
29483
2
1
1
0

1
1

 0.00
strain
1453495

1
1
2
0

 0.00
species group
1649845

species

 0.00
633
1
1

2
1
5
6
34037

 0.00
genus

0
2
2
1
34038
species

 0.00

2
1

 0.00
strain
745277

1
1
58169
species

 0.00

1510570
species

 0.00
1
1

3
1
4
1

 0.00
family
1903412

genus

 0.00
82982
2
1
3
0

82983

 0.00
species
1
3

543
family

 0.05
152
355
47
1


 0.00
genus
561
3
3
1
0

2
3
1
2
562
species

 0.00

1
1
244319
serotype

 0.00

1
1
3
5
36866
no rank

 0.00

2045202

 0.00
species
3
1

1
1

 0.00
species
2066051


 0.00
genus
1330545
2
1
3
0


 0.00
species
1907578
1
1

species

 0.00
61646
1
1

1
111
1
7
2890311
no rank

 0.02

160674

 0.00
genus
5
7
1
3

1
1
575

 0.00
species

species

 0.00
54291
1
1

103
1
3
57
genus

 0.01
570

45
1
573
species

 0.01

1
1

 0.00
species
1134687

genus

 0.00
544
14
4
1
8

species group

 0.00
1344959
1
2
3
0

1
3

 0.00
species
67826

1
3

 0.00
no rank
2644389

14
1
7
32
547
genus

 0.00

species group

 0.00
354276
18
6
1
14

1
1
2
2
158836

 0.00
species

1
1
1296536
subspecies

 0.00


 0.00
species
1812935
1
1

no rank

 0.00
2757714
1
1
2
0

1
1
1686399

 0.00
species

14
25
1
11
413496

 0.00
genus

2
1
3
0
no rank

 0.00
2649764

3
1
species

 0.00
1906275

413501

 0.00
species
0
5
1
2

5
1

 0.00
strain
1159613

species

 0.00
413502
2
1
1
0

1
1
693216
strain

 0.00

1
1
species

 0.00
28141

0
3
1
1
413497
species

 0.00

413498

 0.00
subspecies
0
1
2
1

1
1
1159554
strain

 0.00

genus

 0.00
579
1
3
9
0

1
1
51288
species

 0.00

species

 0.00
61648
1
8

590

 0.00
genus
0
1
1
3

0
1
2
1
28901

 0.00
species

1
1
59203

 0.00
subspecies


 0.00
genus
1330547
2
1
1
0


 0.00
species
208223
1
1

226
1
19
0
order

 0.03
135625

7
1
18
226
712
family

 0.03

15
7
1
3

 0.00
genus
416916

1
4
8
2
no rank

 0.00
2639383

3
1
712150

 0.00
species

2820817
species

 0.00
1
1

2
1
species

 0.00
2866570

1
2
4
2

 0.00
species
732

2
1

 0.00
strain
634176


 0.03
genus
724
1
10
204
15


 0.00
species
735
1
1

1
3
2582921

 0.00
species

1
1
2
0

 0.00
no rank
2609962

1
1
712310

 0.00
species

2
1
species

 0.00
727

729
species

 0.02
169
174
1
2

5
1
862965
strain

 0.00

726

 0.00
species
7
1

1
1
species

 0.00
249188

135622

 0.00
order
0
4
1
10

family

 0.00
267888
1
4
1
0

0
1
3
1
53246
genus

 0.00

0
1
2
1
288

 0.00
species

342610
strain

 0.00
1
1

3
5
1
0

 0.00
family
267890

22
genus

 0.00
0
1
4
3

196818

 0.00
no rank
0
2
1
2

2
1
2029986
species

 0.00

1
1
species

 0.00
62322

1
7
4
0
order

 0.00
135623


 0.00
family
641
6
1
4
0

0
2
1
1
662

 0.00
genus

1
1
676
species

 0.00

1
3
3
0
genus

 0.00
657

0
3
1
2
74109
species

 0.00

1
3
strain

 0.00
298386

1
1
2
0
no rank

 0.00
33811


 0.00
species
1778877
1
1

0
38
8
1
135624
order

 0.01

0
1
7
38
84642

 0.01
family

31
38
6
1
642

 0.01
genus

644
species

 0.00
2
1

no rank

 0.00
257493
3
1
3
0

species

 0.00
2033033
2
1

1636606
species

 0.00
1
1

2
1

 0.00
species
648

0
779
43
1
2887326

 0.11
order

468

 0.11
family
3
42
1
779

5
4
1
1

 0.00
genus
497

1
2
2
1
196806
no rank

 0.00

2983299
species

 0.00
1
1

1
2
species

 0.00
861445

469
genus

 0.05
115
354
30
1

species group

 0.00
909768
5
1
27
3

1530123

 0.00
species
1
2

1
1
48296

 0.00
species

20
1
470
species

 0.00

species

 0.00
106654
1
1

2053287

 0.00
species
1
5

40214

 0.00
species
1
29

465797
species

 0.00
4
1


 0.00
no rank
196816
8
1
10
0

1
1
2798861

 0.00
species

1
1
2929509

 0.00
species

2004644

 0.00
species
1
2

1
1
species

 0.00
1646498

2953738

 0.00
species
3
1

2810070
species

 0.00
1
1

species

 0.00
2908637
1
1

species

 0.00
202956
1
2

1
1

 0.00
species
1324350

1
1
106649

 0.00
species

1
1

 0.00
species
108981

1
48
40215

 0.01
species

species

 0.01
40216
2
1
55
52

strain

 0.00
981334
3
1

1
2
202954

 0.00
species


 0.00
species
2715163
1
1

species

 0.00
29430
1
1

1
1
202955

 0.00
species

45
1
108980

 0.01
species

6
1

 0.00
species
28090

54393
no rank

 0.00
0
1
2
1

1
1
1889775
species

 0.00

genus

 0.06
475
416
1
5
5

1
42
478

 0.01
species

309
1
34062

 0.04
species

1
1
29433
species

 0.00

1
59
species

 0.01
480


 0.00
order
135615
6
1
4
0

868
family

 0.00
0
4
5
1

2717

 0.00
genus
1
4
1
4

0
1
1
2
2648856

 0.00
no rank

1
1
2866573
species

 0.00

1
2
species

 0.00
2718

135618
order

 0.00
0
1
4
1

family

 0.00
403
3
1
1
0

genus

 0.00
413
1
2
1
0

1
1
414
species

 0.00

2
1
45
2023
135614
order

 0.28

23
2020
1
41
32033

 0.28
family

3
3
1
1
genus

 0.00
83614

species

 0.00
2901869
1
1

1
1

 0.00
no rank
2629088

1913
18
1
120
genus

 0.27
338

339
species

 0.25
1685
6
1
1772

1
1

 0.00
no rank
92826

79
1
2
81
340

 0.01
no rank

190485
strain

 0.00
2
1

359385

 0.00
no rank
3
1
2
5

1
2

 0.00
strain
990315

2259622

 0.00
species
1
1

2
3
1
2
56454

 0.00
species


 0.00
no rank
2754056
1
1

56448

 0.00
species
1
1
3
8

2
1
195709
no rank

 0.00

1
5
487821

 0.00
no rank

1
2
1
0

 0.00
species
1985254

1
1

 0.00
no rank
317013

2053930

 0.00
species
3
1

species group

 0.00
643453
1
2
5
0


 0.00
species
346
1
5


 0.00
genus
141948
2
1
1
0

1463158

 0.00
species
1
1

83618

 0.00
genus
1
8
1
5

species

 0.00
314722
1
2
2
0

1
2
743721
strain

 0.00

2645906

 0.00
no rank
0
5
1
2

5
1

 0.00
species
2571115

1
1
3
8
68

 0.00
genus

1
6
species

 0.00
69

1
1
435897
species

 0.00

40323

 0.01
genus
31
64
1
9

995085

 0.00
species group
0
21
1
3

40324

 0.00
species
20
21
2
1

391008
strain

 0.00
1
1

216778
species

 0.00
2
1

10
1
4
1
no rank

 0.00
196198

1
1
2962886
species

 0.00

1
1
species

 0.00
2742129

1904944
species

 0.00
7
1

1
1
3
0
family

 0.00
1775411

2
1
1
0
genus

 0.00
2707020

1
1

 0.00
species
2010829

12
11
1
0

 0.00
order
135613

11
6
1
0

 0.00
family
1046

2
11
1
5
67575
genus

 0.00

115860
no rank

 0.00
0
8
1
3

1
1
1869214

 0.00
species

7
1
species

 0.00
1763998

2498451

 0.00
species
1
1

72276
family

 0.00
0
1
4
1

1
3
1
0

 0.00
genus
406099

0
2
1
1
2644645

 0.00
no rank

2740807

 0.00
species
1
1

1
85
452
1
order

 0.06
72274

135621
family

 0.06
4
451
1
84

genus

 0.06
286
68
1
438
262

1
1
47886
species

 0.00

1
1
321846
species

 0.00

2
1

 0.00
species
46677

15
46
1
20
196821

 0.01
no rank

1
1
2749808
species

 0.00

2654238
species

 0.00
1
1

2605424

 0.00
species
2
1


 0.00
species
2049589
3
1

5
1
2895485

 0.00
species

1
1
2861799

 0.00
species

species

 0.00
2879114
1
1

1
1
species

 0.00
2731552

253237

 0.00
species
2
1

2866808
species

 0.00
1
1

2738843
species

 0.00
2
1


 0.00
species
2498848
1
1

2855434
species

 0.00
2
1

1
1

 0.00
species
2901380


 0.00
species
2590776
1
1

1
1

 0.00
species
2201356

1
1

 0.00
species
2083055

1
3
2025658
species

 0.00

1
1
species

 0.00
2745519

1
1
2666183
species

 0.00

136842

 0.00
species group
0
12
1
4

1
1
species

 0.00
587753

1
7
86185
species

 0.00

4
1
species

 0.00
296

2056231

 0.00
species
2
1

1
1
species

 0.00
658629

species

 0.00
101564
2
1
2
0

1
2
741155
strain

 0.00


 0.00
species
2681983
1
1

species

 0.00
216142
1
1

136841

 0.00
species group
0
26
8
1

1
17

 0.00
species
287

1232139

 0.00
species subgroup
0
1
2
4

species

 0.00
301
4
1

2
4
3
1
627141
species subgroup

 0.00


 0.00
species
46680
1
1

1
1
95301
species

 0.00

300
species

 0.00
1
1

species

 0.00
237610
2
1

1
1
702115

 0.00
species

5
34
1
11
136843

 0.00
species group

12
1

 0.00
species
47878

species

 0.00
294
1
2
6
5


 0.00
strain
216595
1
1


 0.00
species
75588
1
2

1
1
129817

 0.00
species

200451
species

 0.00
3
1

species

 0.00
76760
1
1

1
1
76758

 0.00
species

1
2
75612
species

 0.00

1
1

 0.00
species
78543

1
1
species

 0.00
198620

1
4

 0.00
species
122355

1
11
2906062
species

 0.00

2
1
191390

 0.00
species

1
1

 0.00
species group
136849


 0.00
species
95300
1
1

species

 0.00
2725477
3
1

species group

 0.00
136845
1
4
19
0

47885
species

 0.00
1
4

13
1
species

 0.00
303

2
1
76759
species

 0.00


 0.00
species
2842349
1
1

351

 0.00
subfamily
0
2
4
1

352

 0.00
genus
0
3
1
2

353
species

 0.00
0
2
1
2

1328314
strain

 0.00
2
1

0
5
6
1
2901164

 0.00
genus

1176257

 0.00
species
1
1

species group

 0.00
136846
4
1
4
0


 0.00
species subgroup
578833
3
2
1
1

2
1
316
species

 0.00

74829
species

 0.00
1
1

1654787

 0.00
genus
0
2
1
1

1
1
species

 0.00
1697053

3
1
1
0

 0.00
genus
2901189

0
1
2
1
136844
species group

 0.00

1
1
species

 0.00
43306

order

 0.00
118969
1
6
5
0

444
family

 0.00
0
1
2
1

genus

 0.00
445
1
1

0
4
1
3
118968

 0.00
family


 0.00
genus
776
4
2
1
1

1
3

 0.00
species
777

0
4
8
1
1706369
order

 0.00

family

 0.00
1706373
3
1
1
0

2
1
1
0
genus

 0.00
48073


 0.00
species
86173
1
1

0
4
1
3
1706371

 0.00
family

10
genus

 0.00
0
3
3
1

no rank

 0.00
2624793
3
1
2
2

1
1
2964606
species

 0.00

11729
1
240
210
class

 1.63
28211

45
1
133
16

 0.02
order
204457

30
108
40
1
41297

 0.02
family

2
1
1
0
genus

 0.00
2709685


 0.00
species
2806553
1
1

165697
genus

 0.00
1
1
7
7


 0.00
species
33052
2
1
3
0

1
1
2
2
2448440
subspecies

 0.00

1219058
strain

 0.00
1
1

2614943
no rank

 0.00
1
1
2
2

1
1
2968500
species

 0.00

267128
species

 0.00
1
2

17
1
60
19
genus

 0.01
13687

152682

 0.00
species
0
1
1
2

strain

 0.00
621456
1
1

species

 0.00
296544
1
1

1
1
13689

 0.00
species

1
18
species

 0.00
185951

0
1
2
1
397260
species

 0.00

1
1

 0.00
strain
1123269

196159

 0.00
no rank
1
9
6
1

species

 0.00
2565555
1
1

2596913
species

 0.00
1
1

2949093
species

 0.00
1
4

1
1

 0.00
species
1517554


 0.00
species
28214
1
1

1
1
653931

 0.00
species

1
8
2759526

 0.00
species

1
1
363835

 0.00
species

0
1
1
2
2685927

 0.00
genus

1
1
species

 0.00
1921510

165696
genus

 0.00
0
4
1
3

3
3
1
1

 0.00
no rank
2644732

702113

 0.00
species
1
1

1
1
2780074
species

 0.00

0
2
1
1
1434046

 0.00
genus

392610
species

 0.00
1
1

1
1
5
5
165695

 0.00
genus

1
2
13690
species

 0.00

2611147

 0.00
no rank
0
2
3
1

1
1
1315974

 0.00
species

species

 0.00
1843368
1
1

family

 0.00
335929
9
1
4
4

5
3
1
4

 0.00
no rank
2800788


 0.00
genus
1111
1
2
1
0

1
1
2683265

 0.00
no rank

3
1
19
38
204441
order

 0.01

433
family

 0.00
3
1
12
32


 0.00
genus
1706033
1
2
1
0

1
1
1225128
species

 0.00

1
4
5
1
441

 0.00
genus

species

 0.00
318683
1
1

442
species

 0.00
0
1
1
2

1
1

 0.00
strain
290633

1
1
257438

 0.00
species

125216

 0.00
genus
17
24
4
1


 0.00
species
257708
1
2
1
0

subspecies

 0.00
204527
1
1

1
6
207340

 0.00
species

family

 0.00
41295
2
1
4
0

0
1
2
1
13134

 0.00
genus

1
1
no rank

 0.00
2617991

111830
genus

 0.00
1
1

1
2
1
0
family

 0.00
2829815

genus

 0.00
191
1
1

1
4
1
0

 0.00
order
1921002

2100208

 0.00
family
0
3
1
1

0
2
1
1
2100210

 0.00
genus

1
1
species

 0.00
1629334

356

 1.53
order
230
1
113
11008

1
5
6
0

 0.00
family
2831100

85413

 0.00
genus
0
6
1
4

no rank

 0.00
2653178
3
1
6
2


 0.00
species
1842539
3
1

1
1
species

 0.00
1867715

69277
family

 0.04
10
20
1
298


 0.04
genus
68287
1
19
288
98

2725666
species

 0.00
1
1

325217

 0.03
no rank
170
14
1
187

2493676

 0.00
species
1
1


 0.00
species
2589974
1
1

2493680

 0.00
species
1
1

species

 0.00
2493673
1
4

1
1

 0.00
species
2493668

species

 0.00
2744518
1
1


 0.00
species
2493672
1
1

1
1

 0.00
species
2493677

1
1
2589903
species

 0.00

2654248
species

 0.00
1
1

1
1
species

 0.00
2493671

2589967
species

 0.00
1
1

1
2
2865837

 0.00
species

1
1
species

 0.00
1777866

71433

 0.00
species
0
1
1
2

1
1

 0.00
strain
1082933

9
1
3
0

 0.00
family
45404

0
1
2
9
45405
no rank

 0.00

2572036
species

 0.00
1
9

2843305
family

 0.00
0
1
1
2

1
1
1632780

 0.00
genus

2831106

 0.00
family
0
1
4
2

46913

 0.00
genus
1
3
1
2

1
2
1
0

 0.00
no rank
196773


 0.00
species
1736675
1
1


 0.00
family
2036754
5
1
3
0

28209
genus

 0.00
1
3
4
1

1
2
1
0
no rank

 0.00
2638111


 0.00
species
1702325
1
1

1
1

 0.00
species
444444

family

 0.01
119045
82
1
16
13

9
1
6
12
407

 0.00
genus

0
1
1
2
2615210

 0.00
no rank

2984843
species

 0.00
1
1

39956

 0.00
species
0
1
1
2


 0.00
strain
908290
1
1

570505

 0.00
species
1
1

3
4
1
5
2282523
genus

 0.00

species

 0.00
223967
1
1

408

 0.00
species
0
1
2
1

1
1

 0.00
strain
661410

23
52
5
1
186650
genus

 0.01

23
1
3
11

 0.00
no rank
2617746


 0.00
species
2807101
1
5

1
7
2740529

 0.00
species

1882682

 0.00
species
1
6

2
4
1
1
family

 0.00
335928

279

 0.00
genus
0
3
1
1

280
species

 0.00
0
1
2
1

1
1
strain

 0.00
78245

82115
family

 0.00
5
19
12
1

227292

 0.00
no rank
1
3
5
1

2
1
1
0
genus

 0.00
106591

species

 0.00
106592
1
1

0
1
2
1
28105
genus

 0.00

1
1
194963

 0.00
species

6
11
1
6
227290

 0.00
no rank

379

 0.00
genus
2
4
1
2

1
2
240521
species

 0.00

genus

 0.00
357
1
3
1
0

1183400
species group

 0.00
0
1
2
1

358

 0.00
species
1
1

37
10344
1
23
41294

 1.44
family

genus

 0.00
1033
1
2
1
0

1
1
40137
species

 0.00

1
3
3
1
1073
genus

 0.00

1
1
species

 0.00
1076

475937
species

 0.00
1
1

10303
17
1
280

 1.43
genus
374

species

 0.00
993502
1
1

4
1
244734
species

 0.00

2
1

 0.00
species
1355477

13
1
10016
188
no rank

 1.39
2631580

5
1

 0.00
species
2590772

1
1
1521768
species

 0.00

2057741
species

 0.01
1
57

1
1

 0.00
species
288000

2493093

 0.00
species
1
2

11
1
2782665

 0.00
species


 0.00
species
2782641
8
1

species

 0.00
1325100
1
1

2782659

 0.00
species
1
2

1
1
species

 0.00
1325120

376

 0.00
species
11
1

2715960

 1.35
species
1
9728

4
1
6
0
family

 0.00
118882


 0.00
genus
354349
1
2
1
0

1
1
2647013
no rank

 0.00

2
3
3
1
2826938
no rank

 0.00

234
genus

 0.00
0
2
1
1

1
1

 0.00
species
94625

0
5
4
1
119042
no rank

 0.00


 0.00
genus
212791
1
3
5
0

0
5
1
2
2632691

 0.00
no rank

1
5
1894999
species

 0.00

2843308

 0.00
family
0
1
4
2

2
1
3
0

 0.00
genus
261933

no rank

 0.00
2627136
2
1
2
0

2
1
1885025
species

 0.00

1
1
4
0

 0.00
family
2831111


 0.00
genus
166953
3
1
1
0


 0.00
no rank
2649303
1
1
2
0

2795690

 0.00
species
1
1

0
11
1
6
766
order

 0.00


 0.00
family
775
6
10
1
0

3
1
1
0

 0.00
genus
1742636

0
1
2
1
1742707
no rank

 0.00

2866314
species

 0.00
1
1

0
5
1
6
33988

 0.00
tribe

1
1
69474

 0.00
genus

0
4
4
1
780
genus

 0.00

4
1
3
0

 0.00
species group
114277

786

 0.00
species
0
4
1
2

293614
strain

 0.00
1
4

0
20
1
214
204458
order

 0.03

76892

 0.03
family
17
19
1
214

genus

 0.02
75
9
1
170
98

1
53
species

 0.01
88688

1
1
species

 0.00
69666

2708539

 0.00
species
1
1

155892

 0.00
species
9
1

species

 0.00
69395
1
1

0
3
1
7
2648921

 0.00
no rank


 0.00
species
2823693
4
1

69665

 0.00
species
1
3

12
27
1
9
41275

 0.00
genus

2800818

 0.00
species
1
1

2
3
1
4
2622653

 0.00
no rank

1
1
2560058

 0.00
species

1
1
2579977
species

 0.00

species

 0.00
74329
3
1

3
1
172043

 0.00
species

3
1

 0.00
species
41276

1
1

 0.00
species
1312364

204455
order

 0.02
21
119
27
1

2854170
family

 0.00
2
6
1
7

2
1

 0.00
genus
74030

0
2
1
2
266808
genus

 0.00

266809
species

 0.00
1
2


 0.00
genus
983507
1
1
2
0

1
1

 0.00
species
1943633

1
20
91
16
family

 0.01
31989

1
2
1
0

 0.00
genus
2946607

1
1
2807096

 0.00
species

0
2
1
1
2683599
genus

 0.00

2579971
species

 0.00
1
1

1284657
genus

 0.00
0
1
3
1

0
2
1
1
1284658

 0.00
species

1
1

 0.00
strain
666509

71
9
1
16

 0.01
genus
265

1945662

 0.00
species
1
14


 0.00
no rank
2688777
3
1
5
1

3
1

 0.00
species
2589076

species

 0.00
2760307
1
1

26
1

 0.00
species
147645

5
1

 0.00
species
2903900

1
1
2259340
species

 0.00

4
1
species

 0.00
59779

991903
genus

 0.00
0
1
1
3


 0.00
species
991904
1
1
2
0

strain

 0.00
991905
1
1

1
5
17
0
class

 0.00
2008785

17
1
4
0

 0.00
order
119069

family

 0.00
206349
3
1
17
0

17
1
2
0
genus

 0.00
70774


 0.00
species
297
1
17

1
10
3
0
class

 0.00
1553900


 0.00
order
213481
5
1
1
0


 0.00
family
213483
1
4
1
0

1
1
3
0

 0.00
genus
958

0
2
1
1
2633795

 0.00
no rank

1916293
species

 0.00
1
1

2
4
1
0
order

 0.00
2024973

0
2
3
1
2024974

 0.00
family

2698753

 0.00
genus
0
2
1
2

1
2
2493639
species

 0.00


 0.00
phylum
200940
1
9
2
0

0
2
1
8
67799
class

 0.00

order

 0.00
188710
7
1
2
0

188711
family

 0.00
0
1
6
2

0
2
1
1
444090

 0.00
genus

1653476

 0.00
species
1
1


 0.00
genus
1740
1
1
3
0

1
1
2
0
species

 0.00
1741

1
1
289377
strain

 0.00

clade

 1.67
1783272
847
1
12005
39


 0.01
phylum
1297
14
1
52
0

188787
class

 0.01
0
1
13
52

0
1
4
3
68933

 0.00
order


 0.00
family
188786
1
3
3
0

270
genus

 0.00
2
3
2
1

1
1
274

 0.00
species

order

 0.01
118964
1
8
49
0

0
49
7
1
183710

 0.01
family

17
6
1
49
1298
genus

 0.01

species

 0.00
57497
1
1

2623546
no rank

 0.00
3
3
1
28

1
1
2939437
species

 0.00

2652443
species

 0.00
1
24

1
3
1182568

 0.00
species

18
2687
326
1
1239
phylum

 0.37

1
62
1
71
186801

 0.01
class

no rank

 0.00
218638
1
1
2
0


 0.00
species
244328
1
1

16
69
59
1
186802
order

 0.01

31979

 0.00
family
1
11
1
9

1
6
6
1

 0.00
genus
1485

1
1
1491

 0.00
species

0
3
1
2
2614128
no rank

 0.00

1
1
1155388

 0.00
species

1970093
species

 0.00
1
1

2
1
species

 0.00
137838

2
1
1
0
genus

 0.00
1848399


 0.00
species
1286698
1
1

no rank

 0.00
189971
1
1
2
0

2830659
species

 0.00
1
1

1
7
4
0

 0.00
no rank
538999

2
1
1
0
genus

 0.00
1686313

1
1
species

 0.00
938288

0
3
4
1
543314
family

 0.00


 0.00
species
143393
2
1
2
0

888727

 0.00
strain
2
1

genus

 0.00
86331
1
1

216572

 0.00
family
3
6
7
1

2591381

 0.00
genus
0
3
1
1

0
1
1
2
2644575

 0.00
no rank

2830675

 0.00
species
1
1

no rank

 0.00
473772
2
3
1
0

species

 0.00
2485925
1
1

1
1
species

 0.00
1572656

0
11
1
13
186804
family

 0.00

1501226

 0.00
genus
4
3
1
10

2626894
no rank

 0.00
5
1

1
1
1115758

 0.00
species

0
1
2
1
1849828

 0.00
genus


 0.00
species
1505
1
1


 0.00
genus
44259
3
1
1
0

0
1
1
2
143361
species

 0.00

546269

 0.00
strain
1
1

2
1
1
0

 0.00
no rank
1411023


 0.00
species
2081703
1
1

family

 0.00
186803
1
21
20
1

no rank

 0.00
2840493
2
1
1
0

39491

 0.00
species
1
1

7
4
1
0
no rank

 0.00
186928

1
2
712982
species

 0.00

species

 0.00
2709410
1
2

3
1
species

 0.00
2109691

841
genus

 0.00
0
1
3
1

0
2
1
1
166486

 0.00
species

1
1
718255
strain

 0.00


 0.00
genus
2719231
1
3
3
1

1
2
2
0

 0.00
species
29370

2
1
1297793

 0.00
strain

207244
genus

 0.00
0
1
1
2

1
1
species

 0.00
105841

0
1
2
3
572511
genus

 0.00

species

 0.00
418240
3
1

genus

 0.00
2944193
1
1
2
0

2763672

 0.00
species
1
1


 0.00
genus
2569097
1
2
2
0

species

 0.00
39488
1
2

39779

 0.00
no rank
1
1

1737404
class

 0.01
0
41
21
1

1737405
order

 0.01
0
17
1
39

38
13
1
0

 0.01
family
1570339

1
2
1
0
genus

 0.00
543311

species

 0.00
33033
1
1


 0.00
genus
150022
26
2
1
0

1
26
1260
species

 0.00


 0.00
genus
162289
9
1
5
1

54005

 0.00
species
2
1

2637196

 0.00
no rank
0
6
1
3

1
5
species

 0.00
2948573


 0.00
species
2921087
1
1

165779
genus

 0.00
0
2
3
1

1
1

 0.00
species
1287640

species

 0.00
33037
1
1

3
1
1
0

 0.00
family
2992717

2992720
genus

 0.00
0
1
2
1

2507161

 0.00
species
1
1


 0.00
no rank
1737407
2
1
3
0

0
2
1
2
1582879
genus

 0.00

1
2
46507

 0.00
species

0
23
1
154
909932

 0.02
class

1843489
order

 0.02
0
12
1
147


 0.02
family
31977
1
11
147
0

39948
genus

 0.00
0
2
1
2

1
2

 0.00
species
39950


 0.02
genus
29465
145
8
1
19

2682456

 0.00
species
1
2


 0.00
species
248315
1
1

105
1
29466
species

 0.01

species

 0.00
39777
7
1

2630086

 0.00
no rank
0
2
1
6

6
1
2682455
species

 0.00


 0.00
species
39778
5
1

909929
order

 0.00
0
10
1
7

family

 0.00
1843491
7
1
9
0

0
1
2
1
158846
genus

 0.00

1
1

 0.00
species
158847

970
genus

 0.00
0
6
6
1

no rank

 0.00
2637378
1
3
5
2

1
1

 0.00
species
712538

713030

 0.00
species
2
1

0
1
2
1
69823

 0.00
species


 0.00
strain
546271
1
1

91061
class

 0.33
4
212
1
2401

186826

 0.15
order
6
1083
94
1

family

 0.12
1300
852
51
1
0

1357
genus

 0.00
5
12
4
1

1
1
1281486
species

 0.00

4
1

 0.00
species
1358

1363

 0.00
species
1
2

1
46
840
257
genus

 0.12
1301

2608887

 0.01
no rank
15
87
1
14

species

 0.00
2759692
1
28

1
1

 0.00
species
2763068

1
9

 0.00
species
712633

2598453

 0.00
species
1
8

species

 0.00
2610896
1
4

1839799

 0.00
species
1
1

species

 0.00
2972784
7
1

1
1
1902136

 0.00
species

712623

 0.00
species
3
1

2710759

 0.00
species
1
1

1
2
2576376

 0.00
species

1
5
712624

 0.00
species

2
1
species

 0.00
1419814

1
19
species

 0.00
1308

78
84
2
1
1305

 0.01
species

388919

 0.00
strain
6
1


 0.00
species
1313
1
11

9
3
1
1
species group

 0.00
671232

1
3
1338

 0.00
species

1
5
1328

 0.00
species

1
6
species

 0.00
2819619


 0.01
species
1302
77
1

1318

 0.00
species
1
1

94
5
1
119
1303

 0.02
species

3
1
strain

 0.00
927666

1
15
1077464

 0.00
subspecies

subspecies

 0.00
1458253
1
1

1
6
subspecies

 0.00
1891914

0
1
2
1
257758
species

 0.00

1
1
1054460

 0.00
strain


 0.00
species
1307
1
1


 0.00
species
68892
5
1

1
4
1433513

 0.00
species

1304

 0.00
species
27
1

species

 0.00
113107
1
1

1
1
species

 0.00
361101

112
1
2
121
28037
species

 0.02


 0.00
strain
365659
9
1

0
3
1
2
45634

 0.00
species

1
3
889201

 0.00
strain


 0.00
species
1811193
1
1

1156431

 0.00
species
2
1

684066
species

 0.00
2
1

1
1
1309
species

 0.00

186828
family

 0.01
0
56
6
1

25
3
1
0
genus

 0.00
117563

1
7
137732

 0.00
species

18
1

 0.00
species
46124

31
1
2
0
genus

 0.00
29393

31
1
species

 0.00
29394

186827

 0.00
family
0
9
1
5

0
1
2
3
1375
genus

 0.00

1
3
87541
species

 0.00

0
2
1
6
46123
genus

 0.00

46125

 0.00
species
1
6

family

 0.02
81852
131
7
1
1

3
4
1
129
1350
genus

 0.02

1
12
1354

 0.00
species

44008

 0.02
species
1
110

1351

 0.00
species
4
1

2
1
1
0
genus

 0.00
2737

2738
species

 0.00
1
1

family

 0.00
33958
29
24
1
0

0
1
4
2
1243
genus

 0.00

species

 0.00
33968
1
1

3016637
1
species group
2

0
1

 0.00
species
1244

115778

 0.00
species
1
1

genus

 0.00
2742598
2
1
2
1

1613
species

 0.00
1
1

0
2
3
1
2767887
genus

 0.00

1601
species

 0.00
1
1

1624

 0.00
species
1
1

genus

 0.00
2767885
1
1
2
0

1
1
28038
species

 0.00

2759736

 0.00
genus
1
2
1
2

1
1
1597
species

 0.00

4
1
2
0
genus

 0.00
2767842

species

 0.00
1590
4
1

1
1
2
0

 0.00
genus
46255

1583
species

 0.00
1
1

1578
genus

 0.00
0
6
1
15

species

 0.00
1584
3
2
1
2

subspecies

 0.00
1585
1
1

147802
species

 0.00
5
1

47770
species

 0.00
1
5

2
1
1579
species

 0.00

1385

 0.18
order
21
1
117
1314

3
1
3
0

 0.00
family
186820


 0.00
genus
2755
2
1
3
0

species

 0.00
2756
1
3

186822

 0.00
family
0
1
5
3


 0.00
genus
44249
4
1
3
0

1
2
1
2
185978

 0.00
no rank

1
1
1306526

 0.00
species

1
1
253703
species

 0.00

1
14
38
0

 0.01
family
186818


 0.00
genus
1649
1
1
3
0

no rank

 0.00
2644826
1
2
1
0


 0.00
species
1750719
1
1

0
1
1
2
648802
genus

 0.00

1
1
species

 0.00
241244


 0.00
genus
1569
1
3
1
0


 0.00
no rank
2647733
1
1
2
0

1
1
2762563
species

 0.00

1372

 0.00
genus
2
4
1
2

1
2
species

 0.00
459472


 0.00
genus
648800
31
1
3
0

76853

 0.00
species
16
1
2
31


 0.00
strain
1002809
15
1

539002

 0.01
no rank
0
39
1
12

5
1
4
0
no rank

 0.00
539742

4
1
4
1
genus

 0.00
33986

1
2
41170

 0.00
species


 0.00
no rank
2644629
1
2
1
0

1
1
species

 0.00
1224749

539738

 0.00
no rank
0
35
1
6

1378
genus

 0.00
5
1
5
35

24
1

 0.00
species
1379

1
4
species

 0.00
29391

84135
species

 0.00
1
1

1
1
2624949
no rank

 0.00

6
1
36
953
90964

 0.13
family

3
1
102
0

 0.01
genus
2803850

1
1

 0.00
species
42858

species

 0.01
1296
101
1

2
1
1
0

 0.00
genus
489909

1
1
species

 0.00
489910

0
9
4
1
227979

 0.00
genus

2630462
no rank

 0.00
0
3
1
9

species

 0.00
946435
1
1

1
8

 0.00
species
2708346

1
24
833
76

 0.12
genus
1279

1280
species

 0.00
1
2

6
1
33028

 0.00
species

species

 0.00
214473
3
1

45972

 0.01
species
107
1

1282
species

 0.02
152
1
2
153

strain

 0.00
1449752
1
1

29385

 0.00
species
11
1

45
1
1290

 0.01
species

1293
species

 0.00
9
1


 0.00
species
1286
1
6

2815305
species group

 0.00
0
1
1
2

53344

 0.00
species
1
1

1
1
2282419

 0.00
species

1288

 0.00
species
1
1

29379

 0.00
species
1
1

1
2
3
0
no rank

 0.00
91994

species

 0.00
2813777
1
3

61
1
2
62
1292
species

 0.01

1
1
1194526
strain

 0.00

2
1
246432

 0.00
species

1
1
species

 0.00
170573

333
1
2
343
29388

 0.05
species

72758

 0.00
subspecies
1
10

2
1
2
0
genus

 0.00
1955413

1
2

 0.00
species
1817405


 0.04
family
186817
46
1
257
22

genus

 0.00
2675231
1
1
2
0

1
1
279826
species

 0.00

genus

 0.00
2675232
2
1
2
0

220684
species

 0.00
1
2

genus

 0.00
129337
6
1
4
4

0
2
1
3
2642459

 0.00
no rank


 0.00
species
1963024
1
1


 0.00
species
1963026
1
1

genus

 0.00
2675229
2
1
1
0

228899

 0.00
species
1
1

1386
genus

 0.03
88
22
1
197

1
2
species

 0.00
1479

185979
no rank

 0.00
0
5
1
7

352858
species

 0.00
4
1


 0.00
species
2846779
1
1


 0.00
species
1868655
1
1

2499213
species

 0.00
1
1


 0.00
species
1408
2
1

1
2
1
0
species group

 0.00
1792192

1
1
293387

 0.00
species

1
2
1
0

 0.00
species group
653685


 0.00
species
1402
1
1

86661

 0.01
species group
37
96
1
10

1396
species

 0.00
1
5

2
1
34
33
species

 0.00
1405

315730
strain

 0.00
1
1

species

 0.00
2026191
1
1

3
1
species

 0.00
1428

155322
species

 0.00
1
1

1392

 0.00
species
1
3

12
1
2
11

 0.00
species
1890302

2494319

 0.00
no rank
1
1

genus

 0.00
2817139
1
1
2
0

1
1
species

 0.00
1398

12
1
2
0
genus

 0.00
1276290

35841
species

 0.00
12
1

1
2
12
2
genus

 0.00
2800373

1
10
1404
species

 0.00


 0.00
genus
400634
4
1
2
0

1
2
1
0

 0.00
no rank
2636778

1
1
2796169

 0.00
species

species

 0.00
28031
1
1

0
3
1
1
1884449
genus

 0.00

2635229
no rank

 0.00
0
1
1
2

2924031

 0.00
species
1
1

0
2
1
7
526524
class

 0.00

2
6
1
0

 0.00
order
526525

3
1
1
0

 0.00
family
128827

0
1
1
2
1647
genus

 0.00


 0.00
species
225084
1
1

2810281
family

 0.00
0
1
1
2


 0.00
genus
191303
1
1

5
16
1
0
phylum

 0.00
544448


 0.00
order
2790996
1
5
1
0

1
4
1
0

 0.00
family
2895623

0
1
1
3
2895509
genus

 0.00

1
2
1
0
species

 0.00
2098

strain

 0.00
347256
1
1

31969
class

 0.00
0
4
10
1

order

 0.00
2085
9
1
4
0

8
1
4
1

 0.00
family
2092

0
1
1
3
295595
genus

 0.00

1
2
1
0

 0.00
species
295596

1
1
1427984
strain

 0.00

2767358
genus

 0.00
0
1
1
2

1
1
171279

 0.00
species

2
1
1
0

 0.00
genus
2923352


 0.00
species
29562
1
1

phylum

 0.00
200795
2
5
1
1

0
4
1
1
292625
class

 0.00

292629

 0.00
order
0
3
1
1

1674871

 0.00
no rank
0
1
1
2

2073117

 0.00
species
1
1

0
1
24
21
1798711
clade

 0.00

phylum

 0.00
1117
23
1
21
1

3028117
22
20
class

2
1
5
1
order

 0.00
1890424


 0.00
family
1890431
1
4
1
0

217161

 0.00
genus
0
1
3
1

0
1
2
1
1173032

 0.00
species

1173020
strain

 0.00
1
1

1301283

 0.00
subclass
0
1
8
3

1150

 0.00
order
1
3
7
1

0
4
1
1
1892254

 0.00
family

genus

 0.00
1155738
1
1
3
0

0
2
1
1
1155739
species

 0.00

1454205

 0.00
strain
1
1

1
1
2
0
family

 0.00
1892252


 0.00
genus
54304
1
1

3
1
9
15
1161
order

 0.00


 0.00
family
1162
1
1
4
0

1177

 0.00
genus
0
1
1
3

272131

 0.00
species
0
1
1
2

1
1
63737
strain

 0.00

1892263
family

 0.00
0
11
1
4

genus

 0.00
1190
1
3
11
0

494603
no rank

 0.00
0
2
1
11

1191
species

 0.00
11
1

9199
461
1
74

 1.28
phylum
201174

84992
class

 0.00
0
2
1
6

order

 0.00
84993
2
5
1
0

family

 0.00
633392
1
4
2
1

1648491
genus

 0.00
0
3
1
1

0
1
1
2
2633173

 0.00
no rank

1
1
2849779

 0.00
species

class

 1.27
1760
9098
1
426
1316

1
16
48
0
order

 0.01
1643682

85030
family

 0.01
20
48
15
1

no rank

 0.00
234661
1
2
7
0

1
7

 0.00
species
2596920

1860

 0.00
genus
0
1
3
1

1861
species

 0.00
0
1
2
1


 0.00
strain
526225
1
1

88138

 0.00
genus
1
1
4
9

2643866

 0.00
no rank
0
4
2
1

4
1

 0.00
species
2851567

species

 0.00
477641
4
1


 0.00
genus
38501
11
1
5
3

3
1
2
0

 0.00
species
138336

strain

 0.00
1146883
3
1

2619396

 0.00
no rank
0
2
1
5

1
5
2933797

 0.00
species


 0.00
order
85012
14
13
1
5

1
4
1
5
2012

 0.00
family

4
3
1
2

 0.00
genus
1988

1
2
1
2
2626254
no rank

 0.00

species

 0.00
2591108
1
1

3
1
5
0

 0.00
family
83676

2013
genus

 0.00
1
3
4
1

1
1

 0.00
species
2014

1
2
1
0

 0.00
no rank
2649073

2831968

 0.00
species
1
1

3
1
1
0

 0.00
family
2004

1
1
2
0
genus

 0.00
83681

1
1

 0.00
species
2219224

85010

 0.01
order
0
91
1
32

31
1
91
31
family

 0.01
2070

1
4
10
3

 0.00
genus
165301

1
4

 0.00
species
1586287

2643253

 0.00
no rank
0
3
2
1

2951806

 0.00
species
1
3

1
3
4
1
2029
genus

 0.00

species

 0.00
860235
1
1


 0.00
no rank
2637669
1
1
2
0

703222

 0.00
species
1
1

genus

 0.00
1847
4
1
15
4

1
2

 0.00
species
2074

8
9
1
2
2619320

 0.00
no rank

445576

 0.00
species
1
1

1835

 0.00
genus
1
11
1
3

1
9
33914

 0.00
species

1836
species

 0.00
1
1

genus

 0.00
39845
3
1
6
0

no rank

 0.00
2642662
6
1
2
0

2268449

 0.00
species
1
6


 0.00
genus
1813
11
6
1
7

0
1
1
2
2893673

 0.00
species group


 0.00
species
129921
1
1

1
1

 0.00
species
218821

2618356

 0.00
no rank
1
2
1
2

2745196

 0.00
species
1
1


 0.00
genus
65496
1
3
1
0

2
1
1
0

 0.00
no rank
2644606

1
1
species

 0.00
2072503

2071
genus

 0.00
1
3
1
3

2
1
2
0

 0.00
no rank
2593673

1
2
2781735

 0.00
species

order

 0.00
85014
5
1
1
0

1
4
1
0

 0.00
family
85034

1
3
1
0
genus

 0.00
58113

0
1
2
1
2637084

 0.00
no rank

1
1
2867006

 0.00
species

0
5
1
17
85008

 0.00
order

13
1
4
17
28056

 0.00
family

2
3
1
4
1873

 0.00
genus

47853
species

 0.00
1
1


 0.00
species
285665
1
1

0
1
1
4
1217098
order

 0.00


 0.00
family
1217100
1
3
1
0

281472

 0.00
genus
0
1
1
2

species

 0.00
419479
1
1

2039638

 0.00
order
0
20
1
7

20
6
1
0
family

 0.00
2162846

2
1
5
20
622681

 0.00
genus

1
15
species

 0.00
1884904

species

 0.00
1884905
1
1

1
1
1884913

 0.00
species

1
1

 0.00
species
1884914


 0.00
order
85013
1
4
1
0

74712
family

 0.00
0
1
1
3

no rank

 0.00
418002
1
2
1
0

1
1
418003
species

 0.00


 0.01
order
85004
1
10
45
0

family

 0.01
31953
9
1
45
0

0
2
1
3
196082

 0.00
genus

0
2
1
2
78258
species

 0.00

2
1
864564
strain

 0.00

29
1
3
1

 0.00
genus
1678

19
1

 0.00
species
33905

1
9
species

 0.00
1689

14
2
1
0
genus

 0.00
2701

1
14
species

 0.00
2702

340
1
32
0

 0.05
order
2037

340
1
31
3

 0.05
family
2049

2529408
genus

 0.00
0
22
7
1

131110
species

 0.00
1
1


 0.00
species
1660
1
19

species

 0.00
181487
1
2
1
0

1
1
strain

 0.00
888050

no rank

 0.00
2691889
1
1
2
0

1
1

 0.00
species
2709403

5
2
1
0

 0.00
genus
2740557

5
1
species

 0.00
178339

1654

 0.04
genus
92
1
17
304

1
1
55565
species

 0.00

1656
species

 0.00
1
3

26
140
1
11
2609248
no rank

 0.02


 0.00
species
649739
2
1
5
0

5
1

 0.00
strain
649743

712116
species

 0.00
28
1

1
1
2081702
species

 0.00

species

 0.00
2755559
1
23

species

 0.00
2789424
3
1

0
19
1
2
706438
species

 0.00

19
1

 0.00
strain
706439

1
25

 0.00
species
712122

species

 0.00
2789425
10
1

1
31

 0.00
species
1655

1
2
37
33
species

 0.01
544580


 0.00
strain
871541
1
4


 0.00
genus
184869
2
1
2
0

2811781

 0.00
species
2
1

2767327

 0.00
genus
0
4
2
1

4
1
species

 0.00
82135

0
28
1
259
85011
order

 0.04

259
27
1
11

 0.04
family
2062

26
1
248
182
genus

 0.03
1883

1
1

 0.00
species
1889

16
1
56
16

 0.01
no rank
2593676

2964669
species

 0.00
1
3

1
1
species

 0.00
2866715

1
1
2695266
species

 0.00

1
1
349971

 0.00
species

2898276

 0.00
species
1
1

1
2
2563602

 0.00
species


 0.00
species
2742136
1
1

1
1
2721246
species

 0.00

1
1
2801029
species

 0.00

21
1
species

 0.00
2923272

1
1

 0.00
species
2930049

2109593
species

 0.00
1
1

1
3
species

 0.00
2072505

2211357
species

 0.00
1
1

1
1
2662065

 0.00
species

553510
species

 0.00
1
1

species

 0.00
1977088
1
1

1
1
species

 0.00
2174846

1535768

 0.00
species
1
2

67304

 0.00
species
1
1

436397
species

 0.00
1
1

1413221

 0.00
species
1
1

2496836
species

 0.00
1
1

2858
1
36
10
order

 0.40
85009

85015

 0.02
family
21
1
19
116

1839
genus

 0.01
56
12
1
89

4
1
species

 0.00
2894081

18
1
species

 0.00
160826


 0.00
no rank
2615069
7
1
9
3

1
1

 0.00
species
2763008

1
1
2895565
species

 0.00


 0.00
species
2017486
1
1

1
1
2582905

 0.00
species

2714939
species

 0.00
1
1


 0.00
species
2840457
1
1

419476

 0.00
species
1
1

1
1
2714938

 0.00
species

2
1
2
0

 0.00
genus
86795

642780
species

 0.00
2
1

2
1
2
0
genus

 0.00
2044

2
1

 0.00
species
2045

0
2
2
1
53387

 0.00
genus

546871
species

 0.00
2
1

2732
1
16
3

 0.38
family
31957

72763

 0.00
genus
0
2
1
1

2161816
species

 0.00
1
1

1743

 0.00
genus
0
3
3
1

species

 0.00
1744
1
1

1
2

 0.00
species
556499

2801844
genus

 0.00
0
4
1
3

1
1

 0.00
species
1547448

1
3

 0.00
species
1750

1912216
genus

 0.38
48
1
7
2721

7
1
33011

 0.00
species

7
1
species

 0.00
33010

species

 0.00
2559073
1
5

2649
3
1
2654
1747
species

 0.37

5
1
2
2

 0.00
subspecies
1734925

3
1
1114967

 0.00
strain

1320
1
127
68

 0.18
order
85006

0
10
8
1
85020

 0.00
family

genus

 0.00
43668
1
5
5
2

species

 0.00
2017485
1
1


 0.00
species
43669
2
1
1
0

1
1
strain

 0.00
446465

species

 0.00
556288
1
1

0
5
2
1
36739

 0.00
genus

1667168
species

 0.00
1
5

1268

 0.04
family
14
1
32
307

1
6
45
3

 0.01
genus
57493

1
2

 0.00
species
1049583

6
1
71999
species

 0.00

72000

 0.00
species
1
30

446860
species

 0.00
1
1

species

 0.00
1272
3
1

47
82
5
1
1269
genus

 0.01

566027

 0.00
species
1
1

1
33
species

 0.00
1270

0
1
2
1
2620948
no rank

 0.00

1
1
1179670

 0.00
species

1
153
1
7
32207

 0.02
genus

1
21
species

 0.00
172042

43675
species

 0.01
86
93
1
2

1
7

 0.00
strain
680646

9
1
species

 0.00
37923

23
29
2
1
2047

 0.00
species

762948

 0.00
strain
1
6

1742989
genus

 0.00
1
1
4
5


 0.00
species
256701
1
2
1
0

1
1
strain

 0.00
861360

1
3

 0.00
species
162496


 0.00
genus
1663
7
6
1
3

1
1
2895818

 0.00
species

no rank

 0.00
235627
3
4
1
0

2879617
species

 0.00
1
1

1
1
species

 0.00
2972479

1
1
species

 0.00
2831000

1
3
1
0

 0.00
genus
1742993

0
1
1
2
2647000

 0.00
no rank


 0.00
species
2973976
1
1

0
71
9
1
85019
family

 0.01


 0.01
genus
1696
71
1
8
37

1
16
33889

 0.00
species


 0.00
species
199591
1
11

1
1
2898795
species

 0.00

1
3
1703

 0.00
species

0
3
1
3
2614124

 0.00
no rank

1
1
2575923
species

 0.00

2
1

 0.00
species
2823518

2
1
2
0
family

 0.00
2805590


 0.00
genus
125287
1
2

17
1
6
1

 0.00
family
85016

16
5
1
10
genus

 0.00
1707

2819978
species

 0.00
1
1

0
4
2
1
2620175
no rank

 0.00

species

 0.00
2654191
4
1

1
1
76862

 0.00
species

0
1
1
2
85017

 0.00
family

1
1
157920
genus

 0.00

2805426
family

 0.00
0
3
3
1

57499

 0.00
genus
0
2
1
3

1276
species

 0.00
1
3

145357
family

 0.09
0
659
1
5


 0.09
genus
57495
1
4
659
12

no rank

 0.00
2643059
2
1
4
0

1
4
2762331

 0.00
species

1
643
species

 0.09
1274

family

 0.00
85021
7
1
6
0

genus

 0.00
53457
7
5
1
3

262209

 0.00
species
1
2

1
1
53458

 0.00
species

2
1
1
0
no rank

 0.00
2649294

1
1
2761047
species

 0.00

85023
family

 0.02
41
1
53
175

337004

 0.00
genus
1
1
3
2

1
2
1
0
no rank

 0.00
2630066

1
1
2963406
species

 0.00

4
1
15
0
no rank

 0.00
1655488

clade

 0.00
1655489
15
3
1
0

genus

 0.00
529883
1
2
15
8

1
7
species

 0.00
529884


 0.00
genus
33877
5
1
3
4

0
2
1
1
2639701
no rank

 0.00


 0.00
species
2498704
1
1


 0.00
genus
33886
2
2
1
1

1
1
no rank

 0.00
2609250

2034
genus

 0.00
1
3
3
1

no rank

 0.00
257496
2
1
2
1

1
1
1561023
species

 0.00


 0.00
genus
55968
1
2

6
4
1
1

 0.00
genus
46352

399736

 0.00
species
1
1

2615065
no rank

 0.00
1
4
2
1

1
3
2810513

 0.00
species

1
3
1
0
genus

 0.00
355929

2645988
no rank

 0.00
0
1
1
2

2021374
species

 0.00
1
1

37
85
19
1
33882

 0.01
genus

904291
species

 0.00
1
1

1
4
582680

 0.00
species

1
1

 0.00
species
2614638


 0.00
species
370764
1
1

species

 0.00
82380
1
1


 0.00
species
1072463
1
7

species

 0.00
2509458
1
1

162426

 0.00
species
1
2

27
9
1
2
no rank

 0.00
2609290

5
1
2606451

 0.00
species

1
1
species

 0.00
1714373

9
1
1906274
species

 0.00

2014534

 0.00
species
5
1


 0.00
species
2048898
1
1

1
1
2851642

 0.00
species

1
2
species

 0.00
2810535

1
1
1906742
species

 0.00

3
1
species

 0.00
743009

2
1
2
8
1705353
genus

 0.00

1987356
species

 0.00
6
1


 0.00
genus
255204
3
1
3
0

2615177
no rank

 0.00
0
3
1
2

2866588

 0.00
species
1
3

2680004
genus

 0.00
0
1
3
1

2680006
no rank

 0.00
0
1
2
1

1
1
2759943
species

 0.00

1
2
1
0

 0.00
genus
427753

species

 0.00
2599293
1
1

0
3
6
1
2805415

 0.00
order


 0.00
family
2805416
3
1
5
0


 0.00
genus
1434010
3
1
4
0

2038343

 0.00
species
2
1

2629395
no rank

 0.00
0
2
1
1

1
1

 0.00
species
1907575

order

 0.38
85007
100
1
2764
43

family

 0.06
1653
415
36
1
4

1716

 0.06
genus
97
35
1
411

43990
species

 0.00
1
1

species

 0.00
401472
6
1

4
1
161899

 0.00
species

169292

 0.00
species
1
6

43768
species

 0.01
1
63

0
1
1
2
1231000

 0.00
species

1
1
1408189
strain

 0.00


 0.00
species
1717
1
1

1
1
187491
species

 0.00

1
12
species

 0.00
156976

4
1
161879
species

 0.00

species

 0.00
1725
1
1


 0.00
species
156978
5
1

60
1
1979527
species

 0.01

1
3
species

 0.00
2735136

5
1
species

 0.00
38301

2
1

 0.00
species
38303

1
15
43769

 0.00
species

2624378
no rank

 0.00
0
1
3
3

1
1

 0.00
species
702967

2
1

 0.00
species
2778078

2
1
species

 0.00
38290

8
1
species

 0.00
43770

1
10
53374

 0.00
species

9
1
2
8

 0.00
species
38289

306537

 0.00
strain
1
1

18
1

 0.00
species
37637

species

 0.00
38288
19
2
1
0

585529
strain

 0.00
19
1

38304
species

 0.01
48
1


 0.00
species
39791
1
1


 0.00
species
146827
1
5

2
1
1
0
species

 0.00
1404244

1
1
strain

 0.00
1404245

67
26
1
2164
1762

 0.30
family

0
4
1
1
670516
genus

 0.00

0
1
3
1
36809
species

 0.00

319705

 0.00
subspecies
0
1
1
2

1
1
strain

 0.00
1303024

1763
genus

 0.01
18
11
1
44

1775
species

 0.00
1
1

7
1
1389713

 0.00
species

1778
species

 0.00
1
10


 0.00
species
1552759
1
1

2642494
no rank

 0.00
0
1
4
4

1879023
species

 0.00
1
1

373038
species

 0.00
1
1

1
2
species

 0.00
2857059

1
1

 0.00
species
482462


 0.00
species
134601
1
2

1866885

 0.29
genus
46
2052
1
10

2
1
species

 0.00
59813

370526
species

 0.00
1
1


 0.00
species
319706
23
1


 0.00
species
1795
1
10

1962
1
species

 0.27
319707

2
1

 0.00
species
53462

2636767

 0.00
no rank
4
6
1
3

species

 0.00
2606611
1
1

1
1

 0.00
species
1968788

0
26
9
1
85026
family

 0.00

10
1
8
26
2053
genus

 0.00

1
1
36822
species

 0.00

species

 0.00
2055
1
1

species

 0.00
249058
10
1

1
2
84595

 0.00
species

0
1
3
2
2657482

 0.00
no rank

2698900

 0.00
species
1
1

species

 0.00
2878678
1
1

85029
family

 0.01
0
37
7
1

37
1
6
5
genus

 0.01
37914

1
2
322509
species

 0.00

6
1
546160
species

 0.00

2617939

 0.00
no rank
2
1
2
21

1
19
species

 0.00
712270

3
1

 0.00
species
499555


 0.01
family
85025
70
1
15
1

1
4

 0.00
genus
1817

1827
genus

 0.01
47
13
1
65

1
1
species

 0.00
223392

1828

 0.00
species
3
2
1
6


 0.00
strain
1051973
3
1

192944
no rank

 0.00
0
5
1
4

1045808
species

 0.00
3
1

1
1
1564114

 0.00
species

1
1
species

 0.00
2795031

1
1
103816
species

 0.00

2840174
species group

 0.00
1
4
1
5


 0.00
species
1833
3
1
2
2

234621
strain

 0.00
1
1

334542
species

 0.00
1
1

1
3
2
0
family

 0.00
85028

1
2
2
1

 0.00
genus
2060

species

 0.00
47312
1
1

2805586
family

 0.00
0
7
3
1

0
7
2
1
1847725

 0.00
genus

1
7

 0.00
species
1528099

2
6
9
1
1497346

 0.00
class

8
1
4
2
order

 0.00
588673

2758916

 0.00
family
0
1
1
3

0
1
2
1
2758920

 0.00
genus

2949664
species

 0.00
1
1

320583
family

 0.00
0
1
1
4

genus

 0.00
191494
1
1
3
0

2627773

 0.00
no rank
0
1
2
1

2812560

 0.00
species
1
1

1
17
14
1
84998
class

 0.00

3
1
3
0
order

 0.00
1643822

1643826

 0.00
family
2
1
2
3

1
1
2815775
genus

 0.00

13
10
1
0
order

 0.00
84999

2
1
9
13
1643824
family

 0.00

0
8
1
5
133925
genus

 0.00


 0.00
no rank
2638792
7
1
2
0

712411

 0.00
species
7
1

1
2
1
0

 0.00
species
133926


 0.00
strain
633147
1
1

3
3
1
0
genus

 0.00
2767353

1
2
3
2

 0.00
species
1382

1
1
521095
strain

 0.00

84995

 0.00
class
0
2
5
1

84996
order

 0.00
0
1
4
2

0
1
3
2
84997
family

 0.00


 0.00
genus
42255
2
2
1
1

1
1
species

 0.00
42256

0
1
9
3
40117
phylum

 0.00

class

 0.00
203693
8
1
3
0

0
3
1
7
189778
order

 0.00

0
3
6
1
189779
family

 0.00

genus

 0.00
1234
3
5
1
0

1
1
330214
species

 0.00

2
1
1
0

 0.00
no rank
2652172

70125

 0.00
species
1
1

species

 0.00
2652173
1
1


 0.26
phylum
57723
1883
1
11
0

204432
class

 0.26
1
1
10
1883

1882
9
1
0
order

 0.26
204433

8
1
1882
9

 0.26
family
204434

3
1872
4
1
388463
genus

 0.26

0
1
2
1
2637509
no rank

 0.00

2703788

 0.00
species
1
1

1933044

 0.26
species
1868
1

0
3
1
1
940557

 0.00
genus

1
1
2
0

 0.00
no rank
2621151

species

 0.00
2602070
1
1

0
55
1
23
32066

 0.01
phylum

0
22
1
55
203490

 0.01
class

55
1
21
0
order

 0.01
203491

family

 0.00
203492
10
1
31
0

1
9
31
5
genus

 0.00
848

species

 0.00
860
1
1

15
1
4
8

 0.00
species
851

subspecies

 0.00
76859
1
5

1
1

 0.00
subspecies
76857

1
1
155615
subspecies

 0.00

1
2
1
2
1583098
species

 0.00

1307442

 0.00
strain
1
1

1
8
2663009

 0.00
species

1129771

 0.00
family
0
10
1
24

2
1
9
24
32067

 0.00
genus

1
12

 0.00
species
157687

2
1
2
0

 0.00
species
40542

1
2

 0.00
strain
523794

1
1
157688

 0.00
species


 0.00
species
157691
1
2

2633022
no rank

 0.00
1
5
3
1

1
2
712357

 0.00
species

2
1

 0.00
species
712368

3
19
1
5
48479
no rank

 0.00

1393499
species

 0.00
1
1

1
1
1447247
species

 0.00

species

 0.00
1256569
2
1

species

 0.00
77133
1
12

0
16
1
14
203691

 0.00
phylum

0
16
1
13
203692

 0.00
class

0
4
1
1
1643688
order

 0.00

1
1
3
0
family

 0.00
170

1
1
2
0

 0.00
genus
171

1
1
species

 0.00
28182

136
order

 0.00
0
15
1
8


 0.00
family
2845253
3
4
1
0

1
3
3
0

 0.00
genus
157

2638727

 0.00
no rank
1
1


 0.00
species
158
2
1

1643685

 0.00
family
6
12
3
1

4
2
1
6
64895
genus

 0.00

29519

 0.00
species
2
1

2787823
no rank

 0.00
0
10
1
8

1
7
10
0
no rank

 0.00
12908

1
2
2
0
no rank

 0.00
1515699

species

 0.00
1515702
1
2

151659
no rank

 0.00
0
1
4
8

81490

 0.00
no rank
0
1
2
4

species

 0.00
198431
1
4


 0.00
species
155900
4
1

superkingdom

 0.00
2157
22
37
1
0

0
9
1
2
1783275

 0.00
clade

phylum

 0.00
651137
2
8
1
0

31932
order

 0.00
0
7
1
2

338190

 0.00
family
0
1
6
2

0
1
1
3
1007082
genus

 0.00

no rank

 0.00
2641124
1
1
2
0

species

 0.00
2259673
1
1

338191

 0.00
genus
0
1
1
2

1
1
species

 0.00
1229909


 0.00
phylum
28890
27
1
20
0

class

 0.00
183968
1
5
1
0


 0.00
order
2258
1
4
1
0

2259
family

 0.00
0
1
3
1


 0.00
genus
2263
2
1
1
0

1
1
species

 0.00
163003

0
1
14
18
2283794
clade

 0.00

0
18
1
13
183925

 0.00
class


 0.00
order
2158
18
1
12
0

2159

 0.00
family
0
18
11
1

16
1
7
4

 0.00
genus
2172

species

 0.00
83816
2
1
2
0

1
2
strain

 0.00
634498

species

 0.00
230361
1
1

5
1

 0.00
species
294671

2638681
no rank

 0.00
1
4
1
2

1
3
1609968
species

 0.00

2316
genus

 0.00
0
2
1
3

2643926
no rank

 0.00
0
2
1
2

species

 0.00
1789762
1
2

0
1
7
1
2290931
clade

 0.00

1
6
1
0

 0.00
class
183963

5
1
1
0

 0.00
order
1644060

1644061

 0.00
family
0
1
1
4

0
1
3
1
29287
genus

 0.00


 0.00
no rank
2623058
1
2
1
0

species

 0.00
2951804
1
1
