# Supplementary material for: Microbiome and mitogenomics of the chigger mite Pentidionis agamae: potential role as an Orientia vector and associations with divergent clades of Wolbachia and Borrelia
Source: BMC Genomics. 2024 Apr 17;25:380. doi: 10.1186/s12864-024-10301-6 (PMC11025265; doi:10.1186/s12864-024-10301-6)
Supplement: Supplementary file 3 — Additional file 3: Kronagram for Kraken2 output at confidence threshold 0.1 for Pentidionis agamae pool Pa1 [file 12864_2024_10301_MOESM3_ESM.html]

Javascript must be enabled to view this page.

members
magnitude
magnitudeUnassigned
count
unassigned
taxon
rank

CH13\_01\_report

2594487
8463

 11.47 11.17 0.00 0.00 0.00 0.00 0.00 0.00 0.00 0.00 0.00 0.00 0.00 0.00 0.00
14972
15

10239
419

 0.02
206
superkingdom
41
1

12333
22
7

 0.00
no rank
0
1

species
38018
18
1

 0.00

0
1
clade
2
2

 0.00
2693321

species
2
1

 0.00
2693050

1
1

 0.00
2827995
species

1
0
no rank
2

 0.00
1
156614

1

 0.00
1
278008
species

2731342
94
55

 0.00
clade
0
1

kingdom
1
0
2732092
40

 0.00
79


 0.00
17
8
2732415
1
0
phylum

2732421
1

 0.00
7
class
0
1

2732533
6

 0.00
1
order
1
0

1
5

 0.00
151340
0
1
family

1
0
subfamily

 0.00
4
1
2169595


 0.00
3
1
325455
1
0
genus

species
0
1
1513257
1

 0.00
2


 0.00
1
1
2259331
no rank

class
1
0
2732422
9

 0.00
7

order
1
0
2732534
8

 0.00
7

10780

 0.00
7
7
family
1
0

no rank
0
1
535600
1

 0.00
2


 0.00
1
1
2940910
species

0
1
subfamily
6

 0.00
4
40119


 0.00
3
6
207678
1
0
no rank

1341019
6
2

 0.00
species
5
1

1

 0.00
1
1395615
no rank

2732416
71
22

 0.00
phylum
10
1

class
0
1
2732424
6

 0.00
11

order
0
1
2732539
6
10

 0.00

10811
3

 0.00
1
family
1
0

1
2

 0.00
10814
0
1
genus

species
417287

 0.00
1
1

6

 0.00
5
1910928
1
0
family

1985366
1
3

 0.00
genus
0
1

0
1
species
1
2

 0.00
2844550

1985426
1
1

 0.00
no rank

no rank
1
1
1941235
4

 0.00
2

species
2202565
3
1

 0.00

2748377
5

 0.00
51
no rank
1
0

clade
0
1
2202562
49

 0.00
3

species

 0.00
1
7
2202563

species
2656686
1

 0.00
42

2875815
2
1

 0.00
species

2732423
4

 0.00
5
class
0
1

4
4

 0.00
2732536
0
1
order

family
0
1
39724
4
3

 0.00


 0.00
2
4
642248
1
0
no rank

species
1954248
4
1

 0.00

2732090
7

 0.00
2
kingdom
1
0


 0.00
6
2
2732410
1
0
phylum

class
1
0
2732411

 0.00
5
2

2732094
2
4

 0.00
order
0
1

0
1
family
2
3

 0.00
10860


 0.00
2
2
456491
1
0
no rank

2
1

 0.00
2219103
species

0
1
kingdom
13

 0.00
7
2732091

phylum
0
1
2732412
13
6

 0.00

class
0
1
2732413
13

 0.00
5

13

 0.00
4
2732414
0
1
order

1
4
family
3

 0.00
13
10841

1
4
no rank

 0.00
2
9
117574

species
1

 0.00
5
2202644

12877
1
3

 0.00
clade
0
1

1
0
clade

 0.00
2
1
2609695

species
2963853

 0.00
1
1

208
55

 0.01
2731341
0
1
clade

kingdom
1
0
2731360
54

 0.01
208

1
0
phylum

 0.01
53
208
2731618

26
1
class
208

 0.01
52
2731619

1
1
genus
2

 0.00
2
1623304


 0.00
1
1
2231643
no rank


 0.00
3
22
1982251
1
15
genus

no rank
1
6
2079398
2

 0.00
7

2041559
1

 0.00
1
species

no rank
1
0
2100420
2

 0.00
30

species
30

 0.00
1
2100421

0
1
genus
1
3

 0.00
186764

0
1
no rank
1
2

 0.00
2562661

species
1

 0.00
1
1873889

2946166
1

 0.00
3
family

1623305
1

 0.00
1
genus

family
0
1
2560065
3
4

 0.00

2560081
1

 0.00
1
subfamily

no rank
0
1
2572872
2
2

 0.00

2831613

 0.00
1
2
species

genus
0
1
2560124
1
2

 0.00

species
1
1

 0.00
2560430

subfamily
2842528
1
1

no rank
2853007
1
1

species
2686378
1

 0.00
1

2843426

 0.00
3
6
genus
1
4

species
2844294
1

 0.00
1

species
1

 0.00
1
2844298

2731643

 0.00
7
3
family
1
0

2

 0.00
1
2731990
1
0
no rank

species
1
1

 0.00
2693311

1
0
subfamily
4

 0.00
2
2731648

1
0
genus
3

 0.00
2
2732924

1985731

 0.00
2
2
species
1
0

no rank
2

 0.00
1
386793

1
0
genus

 0.00
3
2
2560197

2570374
2
2

 0.00
no rank
0
1

species
2
1

 0.00
2041350

genus
2843418

 0.00
1
4

4

 0.00
2
2169529
1
0
family

0
1
no rank
2

 0.00
3
2175602

species
2831612
1
1

 0.00

species
1

 0.00
1
1651816

2788787
11
99
no rank


 0.00
1
12
2202564
species

species

 0.00
1
1
2828189

species
1

 0.00
68
2170413

2897164

 0.00
1
1
species


 0.00
1
1
2491281
species

species
1

 0.00
1
2825254

species
1

 0.00
1
2973490

2827763
1
1

 0.00
species

1

 0.00
11
2202567
species

1
1

 0.00
2575324
species

1

 0.00
1
2693912
species

1978007
2
3

 0.00
order
0
1

no rank
1
0
2949298
2

 0.00
2

2831617
2

 0.00
1
species

1
0
no rank
11

 0.00
4
12429

4
10

 0.00
2204151
0
1
no rank

35342

 0.00
6
2
no rank
1
0

species

 0.00
1
1
2293298

2058757

 0.00
4
1
family
1
0

0
1
clade
1
3

 0.00
2502018

no rank
0
1
2569970
1
2

 0.00

2761326
1

 0.00
1
species

0
1
no rank
2
3

 0.00
51368

family
0
1
2023203
2
2

 0.00

species
2506597

 0.00
1
2

2732004
26

 0.00
5
clade
1
0

1
0
kingdom
25

 0.00
5
2732005

2732007
4

 0.00
19
phylum
0
1

2732525
5

 0.00
1
class
1
0

order
0
1
2732527
1

 0.00
4

family
1
0
10240
3

 0.00
1

subfamily
1
0
10241

 0.00
2
1

genus
10257
1
1

 0.00

13

 0.00
3
2732523
1
0
class

order
0
1
2732554
2
7

 0.00

1
0
family
6

 0.00
2
549779

2
5

 0.00
985780
0
1
no rank

1
2

 0.00
2094720
0
1
genus

2126984

 0.00
1
1
species

1
0
species
2

 0.00
1
985782

1
1

 0.00
1128131
no rank

order
1
0
2732524
5

 0.00
1

1
0
family
4

 0.00
1
10501

genus
0
1
181083
1
3

 0.00

346674
2

 0.00
1
no rank
1
0

1278261
1
1

 0.00
species

0
1
phylum
1

 0.00
5
2732008

class
0
1
2732529
1
4

 0.00

2732559
3

 0.00
1
order
1
0

10508
1

 0.00
2
family
0
1

genus
1
1

 0.00
10509

3

 0.00
35
186616
1
0
no rank

species
5
1

 0.00
239364

species
340016
1

 0.00
30

clade
1
0
2559587
45

 0.00
9

kingdom
1
0
2732396

 0.00
32
7

2732405
11

 0.00
3
phylum
1
0

class
1
0
2732459

 0.00
5
2

2
4

 0.00
2732541
0
1
order


 0.00
3
2
2946186
1
0
family

genus
0
1
10892
2
2

 0.00

40052
1

 0.00
2
species

5

 0.00
1
2732458
1
0
class

0
1
order
1
4

 0.00
2732540

11006
1
3

 0.00
family
0
1

39756
1
2

 0.00
no rank
0
1

species
1955172
1
1

 0.00

phylum
0
1
2732406
3
13

 0.00

1
5

 0.00
2732463
0
1
class

0
1
order
1
4

 0.00
2732548

family
1
0
39738

 0.00
3
1

137555
1

 0.00
2
no rank
0
1

species
1

 0.00
1
2583950

2732462

 0.00
7
2
class
1
0


 0.00
6
2
2732545
1
0
order

5

 0.00
2
11050
1
0
family

0
1
genus
1
2

 0.00
11051

species
11082
1

 0.00
1

no rank
1
0
38144
2

 0.00
1

species

 0.00
1
1
2602438

2497569
7

 0.00
1
phylum
1
0

1
6

 0.00
2497571
0
1
subphylum

0
1
class
1

 0.00
5
2497577

2499411
1

 0.00
4
order
0
1

1
0
family

 0.00
3
1
11308

2

 0.00
1
197911
1
0
genus

11320
1

 0.00
1
species

1

 0.00
8
2732397
0
1
kingdom

2732409
7

 0.00
1
phylum
1
0

2732514

 0.00
6
1
class
1
0


 0.00
5
1
2169561
1
0
order

1
4

 0.00
11632
0
1
family

1

 0.00
3
327045
0
1
subfamily

1
0
genus
2

 0.00
1
11646

species
1

 0.00
1
11676

no rank
1
0
2585030

 0.00
4
1

439490
1

 0.00
3
no rank
0
1

clade
0
1
35278
1

 0.00
2

340187

 0.00
1
1
species

superkingdom
1
1818
2
3209

 1.00
26001

57723
24

 0.00
15
phylum
1
1

1813735
7

 0.00
6
class
1
0

1
0
order
6

 0.00
6
2910145

2211325
5

 0.00
6
family
1
0

genus
3
1
2004797
6
4

 0.00

species
1855912
1

 0.00
1

2

 0.00
2
2626009
1
0
no rank

species
2

 0.00
1
2802971

class
1
0
1562571
6

 0.00
1

1
0
order
5

 0.00
1
2562241

0
1
family
1
4

 0.00
2562242

genus
1
0
1434048
3

 0.00
1

1
0
no rank

 0.00
2
1
2637716

species
1
1

 0.00
2510637

305072
2

 0.00
1
no rank
1
0

species
1978231
1

 0.00
1

1
0
class

 0.00
8
6
204432

204433
6

 0.00
7
order
0
1

204434
6

 0.00
6
family
1
1

0
1
genus
4

 0.00
3
392733

2

 0.00
4
392734
1
0
species

1

 0.00
4
926566
strain

2608728
1

 0.00
2
genus
0
1

1

 0.00
1
2211140
species

0
1
no rank
32
36

 0.00
2323

32

 0.00
35
1783234
1
1
clade

1783273
12

 0.00
14
clade
0
1


 0.00
3
3
1794810
1
0
clade

1
0
phylum
2

 0.00
3
1752723

2282149

 0.00
1
3
species

1
0
phylum

 0.00
3
1
363464

no rank
1
0
1046947

 0.00
2
1

species

 0.00
1
1
1862133


 0.00
7
8
1794811
1
5
clade

phylum
0
1
1752727
1
2

 0.00

1

 0.00
1
2026716
species

1
0
phylum
2

 0.00
1
1752734

1

 0.00
1
2030810
species

1
2

 0.00
1752729
0
1
phylum

1

 0.00
1
2052152
species

221235

 0.00
3
2
phylum
1
0

2787866
2
2

 0.00
no rank
0
1

species
1643353
2
1

 0.00

95818
17

 0.00
17
phylum
1
1

no rank
1
0
1895827

 0.00
7
10

species
1

 0.00
3
1619070

species
2026720

 0.00
1
1

1

 0.00
1
2572087
species

species
713059
1

 0.00
3

2080739
1
1

 0.00
species

species
1

 0.00
1
713051

1
2

 0.00
2905967
0
1
genus

species
2841263
1
1

 0.00

1
0
class
7

 0.00
5
2093818

2093819

 0.00
6
5
order
1
0

2093822
5

 0.00
5
family
0
1

2093823

 0.00
4
5
genus
1
0

no rank
1
0
2725944
3

 0.00
5

2
1

 0.00
2899133
species

species
2933970

 0.00
1
3

11145
1235

 0.43
1783272
60
1
clade


 0.00
20
39
1297
1
0
phylum

188787

 0.00
19
39
class
1
0

0
1
order
37

 0.00
13
118964


 0.00
12
37
183710
1
0
family

1298
37
11

 0.00
genus
11
1

2623546

 0.00
4
17
no rank
1
0

2080419
11
1

 0.00
species

2652443
1

 0.00
5
species

species
1
1

 0.00
2939437

1
1

 0.00
1182568
species


 0.00
2
1
980427
1
0
species

1288484
1
1

 0.00
strain

species
57497
1

 0.00
1

species
5

 0.00
1
317577


 0.00
1
1
1299
species

68933
2

 0.00
5
order
0
1

2
4

 0.00
188786
0
1
family

2
3

 0.00
270
0
1
genus

1

 0.00
1
456163
species

species
2483360

 0.00
1
1

1
0
clade

 0.01
93
130
1798711

1117
89

 0.00
129
phylum
1
8

121
88
3028117
class

1890505
2
5

 0.00
order
0
1

1
0
family
4

 0.00
2
1890528

genus
1
1
54298

 0.00
3
2

1
0
no rank
2

 0.00
1
2646205

1
1

 0.00
155894
species

1161
29

 0.00
96
order
1
71

1
0
family

 0.00
4
4
1892263

1190

 0.00
3
4
genus
1
0

2

 0.00
4
494603
1
2
no rank

species
1

 0.00
2
1191

family
1
0
1892259
6

 0.00
2

2

 0.00
5
748770
0
1
genus

species
1
0
747522

 0.00
2
1

strain
747523
1

 0.00
1


 0.00
2
1
2622029
1
0
no rank

2529119

 0.00
1
1
species

11

 0.00
12
1162
1
2
family

genus
1
1
1177
10

 0.00
10


 0.00
2
1
374162
1
0
species

strain
1973483
1

 0.00
1

0
1
species
2

 0.00
2
1178

strain
2

 0.00
1
449208

6

 0.00
5
2593658
1
1
no rank

1

 0.00
1
1869241
species

species
1973475
2

 0.00
1

2576902
1
1

 0.00
species

76335
1
1

 0.00
species

7
7

 0.00
2661849
0
1
family

1186

 0.00
6
7
genus
1
0

no rank
0
1
2619626
2

 0.00
3

1954172
1

 0.00
1
species

species
1
1

 0.00
2853227

938406
2

 0.00
5
species
1
0

strain
5
1

 0.00
1973478

1
0
order

 0.00
9
2
2881377

1890438
1

 0.00
4
family
0
1


 0.00
3
1
2664261
1
0
genus

species
1
0
2906798
2

 0.00
1

2835870
1

 0.00
1
strain

1890436
1
4

 0.00
family
0
1

1152
3

 0.00
1
genus
1
0

1
0
no rank
2

 0.00
1
2593292

685565

 0.00
1
1
species

0
1
subclass
15
23

 0.00
1301283

1
0
order
8

 0.00
5
1118

family
1
0
1890464
7

 0.00
5

268175

 0.00
3
1
genus
1
0

2

 0.00
1
2648896
1
0
no rank


 0.00
1
1
2005460
species

genus
0
1
669357
4

 0.00
3

0
1
no rank
4
2

 0.00
2625037


 0.00
1
4
1617448
species


 0.00
14
10
1150
1
3
order

0
1
family
2

 0.00
4
1892254

1158
3

 0.00
2
genus
1
0

0
1
species
2

 0.00
2
482564

strain
179408

 0.00
1
2

0
1
family
5
9

 0.00
1892252

genus
1

 0.00
1
54304

44471

 0.00
4
3
genus
1
0


 0.00
3
3
119532
1
1
species

strain
669365
1

 0.00
1


 0.00
1
1
2303329
strain

1492710

 0.00
3
1
genus
1
0

0
1
species
1
2

 0.00
2874213

strain
2064643
1

 0.00
1

1
0
order
22

 0.00
6
1890424

0
1
family
1
4

 0.00
1890430

1
0
genus

 0.00
3
1
283819

1

 0.00
2
283820
0
1
species

strain

 0.00
1
1
2824559

1
4

 0.00
1213
0
1
family

0
1
genus
1
3

 0.00
2892030

2892031
1
2

 0.00
species
0
1

strain
2829509

 0.00
1
1

2881426
1

 0.00
4
family
0
1

167375
3

 0.00
1
genus
1
0

no rank
1
0
2627006

 0.00
2
1

2831057
1

 0.00
1
species

1890426
5

 0.00
2
family
1
0

4

 0.00
2
1129
1
0
genus

0
1
no rank
2

 0.00
3
2626047

species
1

 0.00
1
1353136

species
1280380
1

 0.00
1

4

 0.00
1
1890429
1
0
family

155977
1

 0.00
3
genus
0
1

1
0
species
2

 0.00
1
155978

1

 0.00
1
329726
strain

1
0
phylum
3

 0.00
1
1798710

1
2

 0.00
1897007
0
1
no rank

species
2052166
1

 0.00
1

67819
3

 0.00
1
phylum
1
0

no rank
1
0
1042316

 0.00
2
1


 0.00
1
1
2033014
species


 0.07
470
1768
1239
1
21
phylum

91061
1376

 0.05
250
class
11
1

127

 0.03
678
186826
1
12
order

0
1
family
22
5

 0.00
186827

1
1
genus

 0.00
2
2
1375

species
51665
1

 0.00
1

20

 0.00
2
46123
0
1
genus

20

 0.00
1
46125
species

1
1
family
72
17

 0.00
186828

genus
1
1
2747
12

 0.00
7

species
2751
5
1

 0.00

147709
1
2

 0.00
species
0
1


 0.00
1
1
1266845
subspecies

0
1
no rank
1

 0.00
2
257487

species
208596

 0.00
1
1

species
1

 0.00
4
174587

0
1
genus
36
2

 0.00
29393

36
1

 0.00
29394
species

1470540
3
4

 0.00
genus
0
1


 0.00
2
1
2621505
1
0
no rank

1903686
1
1

 0.00
species

2

 0.00
1
1868793
species

3

 0.00
20
117563
1
0
genus

species
137732
4
1

 0.00

16

 0.00
1
46124
species

0
1
family
42

 0.00
40
33958

1
2

 0.00
2759736
0
1
genus

species
47715
1
1

 0.00

2742598
1

 0.00
3
genus
0
1

0
1
species
1

 0.00
2
1613

strain
1
1

 0.00
1381124

genus
0
1
2767878
1

 0.00
2

species
1
1

 0.00
2923363

genus
0
1
2767879
1

 0.00
3

species
1
0
1602
2

 0.00
1

strain
1
1

 0.00
1423720

0
1
genus
3

 0.00
3
2767885

species
1

 0.00
2
28038

species
1

 0.00
1
1599

1578
18

 0.00
9
genus
1
1

1

 0.00
7
147802
species

species
1

 0.00
1
1579

1

 0.00
1
2012495
species

species

 0.00
1
2
1584

2269374
1
1

 0.00
species

species
3
1
47770
4
2

 0.00

1381118

 0.00
1
1
strain

species
109790
1
1

 0.00


 0.00
3
3
2767887
1
0
genus

species
1601
2
1

 0.00

species

 0.00
1
1
1624

1253
1
3

 0.00
genus
0
1

1
0
species group
2

 0.00
1
1670797

1
1

 0.00
1254
species

1
1
genus
7

 0.00
7
1243

species
1

 0.00
1
33968

species group
3016637
4
3

0
1
species
1
2

 0.00
1244

subspecies
1
1

 0.00
1607839

1
1
species
2

 0.00
2
115778

1
1

 0.00
1165892
strain

species
1245

 0.00
1
2

46255
6
4

 0.00
genus
0
1

species
1
1

 0.00
1249

species
4

 0.00
1
46256

species
137591

 0.00
1
1

0
1
family
12
7

 0.00
81852

2
1
genus
12

 0.00
6
1350

species
2005703
1

 0.00
1

species
1352
1
1

 0.00

species
44008

 0.00
1
5

53346
1
1

 0.00
species

1354
2

 0.00
1
species

1
1
family
518
57

 0.02
1300

1357
14
4

 0.00
genus
6
1

1
7
species

 0.00
3
8
1358

1

 0.00
2
1360
0
1
subspecies

strain
1

 0.00
1
1046624

503
52

 0.02
1301
156
1
genus

species
1308
20

 0.00
1

species
1335

 0.00
1
5

1303
4

 0.00
71
species
1
67

subspecies
1458253
1

 0.00
1

strain
1

 0.00
1
927666

subspecies
2
1

 0.00
1077464

species
1318
1
1

 0.00

species
1

 0.00
1
1311

1

 0.00
3
2819619
species

species
102684
1

 0.00
1

315405
7

 0.00
3
species
5
1

2

 0.00
2
53354
0
1
subspecies

1

 0.00
2
981539
strain

2382163
1

 0.00
1
species

species
7

 0.00
1
1501662

68892
4

 0.00
1
species

1

 0.00
1
1348
species

species
1302
41
1

 0.00


 0.00
2
2
257758
1
0
species

strain
1054460
1

 0.00
2

1

 0.00
1
684066
species

species
1305

 0.00
1
20


 0.00
1
2
1234680
species

1

 0.00
1
1433513
species

149016

 0.00
1
1
species

1304
2

 0.00
8
species
1
7

strain
1

 0.00
1
347253

45634
10

 0.00
2
species
2
1

8

 0.00
1
889201
strain

46
12

 0.00
2608887
9
1
no rank

species
1

 0.00
4
712633

species
1902136
1

 0.00
1

species
2576376

 0.00
1
4

2
1

 0.00
2610896
species

species
2972784

 0.00
1
2

2
1

 0.00
1419814
species

1

 0.00
7
2598453
species

1

 0.00
7
1759399
species

1

 0.00
1
712623
species

2759692

 0.00
1
6
species

1839799
1

 0.00
1
species

species
113107
5
1

 0.00

28037
73

 0.00
2
species
70
1

strain
3

 0.00
1
365659

species
1307
2

 0.00
1


 0.00
1
7
1313
species

species
1309
1
1

 0.00

4

 0.00
5
671232
1
1
species group

species

 0.00
1
1
1328

3
2

 0.00
1338
2
1
species

862967

 0.00
1
1
strain

1385

 0.03
122
687
order
1
18

0
1
family
6

 0.00
6
186822

6

 0.00
5
44249
1
1
genus

species
1778678
1
1

 0.00

3

 0.00
4
185978
1
1
no rank

2
1

 0.00
2660554
species

species
1
1

 0.00
1536775

530
33

 0.02
90964
5
1
family

69965

 0.00
2
1
genus
1
0

species
1

 0.00
1
2875764

genus
1
0
2803850

 0.00
3
6

1

 0.00
1
42858
species

species
1296
5

 0.00
1

8
3

 0.00
227979
0
1
genus

no rank
1
0
2630462
2

 0.00
8

species
2708346
8
1

 0.00


 0.02
24
510
1279
1
44
genus

29388
2

 0.01
163
species
1
154

72758
9
1

 0.00
subspecies

species
1292
1

 0.00
28

33028
1

 0.00
3
species

1
1

 0.00
1288
species

species
178
1

 0.01
1282

29378

 0.00
1
2
species

3

 0.00
1
29385
species

species
1

 0.00
1
985762

species
5

 0.00
1
1283

3
4

 0.00
91994
0
1
no rank

2929481
1
1

 0.00
species

species
2813777

 0.00
1
1

1715860

 0.00
1
1
species

species
1

 0.00
1
29382

species
1

 0.00
1
170573

species
51

 0.00
1
1290

45972
1

 0.00
17
species

1

 0.00
1
246432
species

1280
2

 0.00
6
species
1
5

strain
523796

 0.00
1
1

species
1
1

 0.00
70255

1
1

 0.00
29379
species

186818
16

 0.00
37
family
1
0

genus
1
0
1649

 0.00
2
2

2

 0.00
1
1650
species

genus
1
0
648802
2

 0.00
1

species

 0.00
1
1
241244

0
1
genus
2

 0.00
2
651660

2320858
1

 0.00
2
species

1569

 0.00
4
28
genus
1
18

no rank
0
1
2647733
4

 0.00
2

species
4
1

 0.00
2762563

6

 0.00
1
1476
species


 0.00
2
1
160795
1
0
genus

species
367743
1
1

 0.00

1372

 0.00
3
3
genus
1
1

1
1

 0.00
414778
species

2233542
1

 0.00
1
species

36

 0.00
15
539002
0
1
no rank

no rank
0
1
539738
25
6

 0.00

1378
5

 0.00
25
genus
1
0

species
84135
1

 0.00
2

1379

 0.00
1
11
species


 0.00
1
11
29391
species

no rank
1

 0.00
1
2624949

11
8

 0.00
539742
0
1
no rank

11

 0.00
7
33986
1
1
genus

340146
1

 0.00
2
species

no rank
1
2
2644629
4

 0.00
7

2829816

 0.00
1
3
species

species
2783796
1

 0.00
1

species
1224749
1

 0.00
1


 0.00
1
1
1428684
species

family
8
1
186817
58

 0.00
48

genus
0
1
2800373
2
2

 0.00

2

 0.00
1
1404
species

5

 0.00
3
2675229
0
1
genus


 0.00
1
4
450367
species

species
1
1

 0.00
421767

1
1

 0.00
2837504
genus


 0.00
2
2
150247
1
1
genus

species

 0.00
1
1
33934

3

 0.00
2
84406
1
0
genus

1
0
no rank

 0.00
2
2
2620237

species
2
1

 0.00
403957

genus
1
2
1386
10

 0.00
21

653685
4

 0.00
3
species group
2
1

1648923

 0.00
1
1
species

species
1402

 0.00
1
1

1
0
no rank

 0.00
3
4
185979

species
1

 0.00
1
1565991

species

 0.00
1
3
2928154

species group
3
1
86661
9
2

 0.00

species
1396
6

 0.00
1

2
1

 0.00
1783501
species

genus
1
0
2817139

 0.00
2
1

species
1
1

 0.00
1398

2837511
1

 0.00
2
genus
0
1

species
79883
1

 0.00
1

genus
1
0
2675234
5

 0.00
3

species
1

 0.00
1
199441

1

 0.00
2
2675276
0
1
no rank

species
2924034
1

 0.00
1


 0.00
1
1
79880
species

2

 0.00
2
2837506
1
0
genus

1397
1

 0.00
2
species

2804231
1
2

 0.00
genus
0
1

1467
1
1

 0.00
species

5

 0.00
5
400634
0
1
genus

species

 0.00
1
1
2115968

species
2

 0.00
1
33935

0
1
no rank
2

 0.00
2
2636778

species
2936682
1

 0.00
2

129337
3

 0.00
1
genus
1
0

2642459
1

 0.00
2
no rank
0
1

1963024

 0.00
1
1
species

1
0
genus

 0.00
2
1
45667

1

 0.00
1
2636472
no rank

1
2

 0.00
74385
0
1
genus

2663022
1

 0.00
1
species

genus
1221880
1
1

 0.00

family
0
1
186824
2

 0.00
3

2023
2

 0.00
2
genus
0
1

2026
2

 0.00
1
species

1
0
class

 0.00
35
52
909932

1843489

 0.00
9
32
order
1
0

0
1
family
32
8

 0.00
31977

0
1
genus
1

 0.00
2
39948

species
2161821
1

 0.00
1

genus
1
5
29465

 0.00
5
31


 0.00
2
1
2630086
1
0
no rank


 0.00
1
1
2682455
species

1

 0.00
2
2682456
species

species
1

 0.00
23
29466

3

 0.00
5
1843488
0
1
order

909930
4

 0.00
3
family
1
0

1
0
genus

 0.00
3
3
33024

species
626940

 0.00
1
1

33025

 0.00
1
2
species

17

 0.00
20
909929
0
1
order

family
1
0
1843491
13

 0.00
15

genus
0
1
52225
1
2

 0.00

1

 0.00
1
52226
species

genus
1
1
158846
10

 0.00
4

species
7
1
158847
8
2

 0.00

strain
1

 0.00
1
657316

species
1
1

 0.00
437897

1
0
genus

 0.00
6
4
970

1
1
no rank
2

 0.00
2
2637378

1
1

 0.00
713030
species

2754044
1
1

 0.00
species

1

 0.00
2
69823
0
1
species


 0.00
1
1
546271
strain

1843490
2

 0.00
6
family
0
1


 0.00
5
2
365348
1
0
genus

2629460
1

 0.00
2
no rank
0
1

species
1
1

 0.00
484770

0
1
species
1

 0.00
2
365349

1192197
1
1

 0.00
strain

class
0
1
1737404
22
13

 0.00


 0.00
3
2
1737407
1
0
no rank

1582879
2

 0.00
2
genus
1
0

species
2

 0.00
1
1852374

order
0
1
1737405
20

 0.00
9

1570339
20
8

 0.00
family
0
1

genus
1
0
150022
2

 0.00
15

species

 0.00
1
15
1260

165779
1

 0.00
1
genus

4
4

 0.00
162289
0
1
genus

species
54005

 0.00
1
2

no rank
1
1
2637196
2

 0.00
2

species
1

 0.00
1
2948573


 0.01
163
293
186801
1
1
class

2

 0.00
3
218638
1
0
no rank

1

 0.00
3
244328
species

order
1
17
186802
143

 0.01
282

186803
116

 0.00
56
family
16
1

1

 0.00
2
1843210
0
1
genus

1

 0.00
1
1727145
species

genus
1
1
841
8
3

 0.00

1

 0.00
6
166486
species

species
1
1

 0.00
301301

6
4

 0.00
186928
0
1
no rank

species
1898203
4

 0.00
1

2109690
1
1

 0.00
species

species
2709410

 0.00
1
1

genus
1
3
572511
8

 0.00
33

6

 0.00
3
40520
0
1
species

strain
1

 0.00
5
411459

strain
1
1

 0.00
657314


 0.00
2
22
418240
1
10
species

strain
12
1

 0.00
1121115

765820
2

 0.00
2
no rank
1
0

species
1

 0.00
2
765821


 0.00
2
3
2944198
1
0
genus

species
2763667
3
1

 0.00

1
0
genus

 0.00
4
3
33042

1
0
no rank

 0.00
2
1
2684943

1
1

 0.00
751585
species

2

 0.00
1
410072
species

0
1
genus
3
2

 0.00
2944193

2763672
1

 0.00
3
species

0
1
genus
1
3

 0.00
2005359

2635385

 0.00
2
1
no rank
1
0

species
2823316
1

 0.00
1

2719313
4

 0.00
4
genus
1
0

2

 0.00
1
333367
1
0
species

strain
1
1

 0.00
518636

species

 0.00
1
3
208479

2719231
1

 0.00
3
genus
0
1

species
1
0
84030

 0.00
2
1

strain
717608
1
1

 0.00

genus
0
1
2569097
8

 0.00
2

1

 0.00
8
39488
species

genus
1
0
2316020
5

 0.00
8

33038
5

 0.00
1
species

46228
2
2

 0.00
species
0
1

1

 0.00
2
471875
strain

33039

 0.00
1
1
species

1
0
genus

 0.00
2
1
1164882

species

 0.00
1
1
617123

no rank
1
0
2840493

 0.00
4
7

1
5
species

 0.00
3
7
39491

strain
657318

 0.00
1
1

657317
1

 0.00
1
strain

3

 0.00
5
189330
1
0
genus

1
1

 0.00
39486
species

species
4
1

 0.00
88431

genus
1
0
28050

 0.00
2
1

39485

 0.00
1
1
species

genus
0
1
207244
7
2

 0.00

species
7
1

 0.00
649756

family
5
1
216572
54
25

 0.00

473772
17

 0.00
2
no rank
0
1

1

 0.00
17
2485925
species


 0.00
10
21
216851
1
7
genus

species
853
2
1

 0.00

2
1
no rank
11

 0.00
7
2646395

1

 0.00
1
2929489
species

4
1

 0.00
2929490
species


 0.00
1
1
2929488
species

1

 0.00
1
2929494
species

species
1
1

 0.00
2929493


 0.00
1
1
2929495
species

411483
1

 0.00
1
species

2

 0.00
1
946234
1
0
genus

species
292800
1

 0.00
1

1
0
genus
2

 0.00
1
35829

species
1

 0.00
1
1677857


 0.00
2
1
459786
1
0
genus

species
1
1

 0.00
2763056

genus
0
1
1263
8

 0.00
6

1
0
no rank

 0.00
3
6
2608920

species
5

 0.00
1
2831966

species
657323
1

 0.00
1


 0.00
1
1
1160721
species

species

 0.00
1
1
2564099

family
1
0
2603322
5

 0.00
3

genus
0
1
1348611
2
2

 0.00


 0.00
1
2
1185412
species

0
1
genus
1
2

 0.00
2603323

species
2173034
1

 0.00
1

1
4

 0.00
186807
0
1
family

genus
0
1
2916693
1
3

 0.00

0
1
species
1

 0.00
2
1564

strain
696281
1

 0.00
1

0
1
family
69
31

 0.00
31979

49082

 0.00
3
1
genus
1
0

no rank
0
1
2638829
1
2

 0.00

1

 0.00
1
1041504
species

1
0
genus
2

 0.00
1
1649459

384636

 0.00
1
1
species

genus
15
1
1485
63
20

 0.00

4
1

 0.00
1492
species

species
46867
2

 0.00
1

7
3

 0.00
2614128
1
1
no rank

species
2849865

 0.00
1
3

1

 0.00
3
411486
species

species

 0.00
1
3
1509

1520
4

 0.00
1
species

species
1497
1
1

 0.00

1513
2

 0.00
1
species

704125

 0.00
1
10
species


 0.00
1
1
169679
species

1501
2

 0.00
1
species
1
0

86416
1
1

 0.00
strain


 0.00
1
1
1491
species

1493
2
2

 0.00
species
0
1


 0.00
1
2
573061
strain

species
1488
1

 0.00
1

238834
9

 0.00
2
species
0
1

1552

 0.00
1
9
subspecies

3

 0.00
1
114627
1
0
genus


 0.00
2
1
461876
1
0
species

350688
1

 0.00
1
strain

3
2

 0.00
1849822
0
1
genus

species
1490

 0.00
1
3

39779
2

 0.00
7
no rank
1
3

species
1898207
4
1

 0.00

1
0
no rank

 0.00
8
3
538999

1686313
1
2

 0.00
genus
0
1

species

 0.00
1
1
2811778

5

 0.00
2
543314
1
0
family

species
1
0
143393

 0.00
2
1

1

 0.00
1
888727
strain


 0.00
2
1
86331
1
0
genus


 0.00
1
1
114527
species

0
1
family
12
11

 0.00
186804

1

 0.00
1
1505652
genus

2
2

 0.00
2743582
0
1
genus

species
2
1

 0.00
89152

2

 0.00
1
1870884
1
0
genus

1

 0.00
1
1496
species

genus
0
1
44259
6
3

 0.00

1
0
species

 0.00
2
6
143361

6
1

 0.00
546269
strain

genus
1
1
1501226

 0.00
2
2

1

 0.00
1
1115758
species

order
0
1
53433
3

 0.00
10

family
1
0
972
9

 0.00
3

1
0
genus
3

 0.00
1
32636

31909
1
2

 0.00
species
0
1

strain
373903
1

 0.00
1

1
0
genus

 0.00
2
1
2899804

1
1

 0.00
2682810
species

genus
0
1
2330
1

 0.00
3

0
1
species
1

 0.00
2
2331

572479
1
1

 0.00
strain

1
0
order

 0.00
7
4
68295

family
0
1
543371
3

 0.00
4

1
1
genus
3

 0.00
3
28895

2622527
2
2

 0.00
no rank
0
1

species
2

 0.00
1
1550240

2

 0.00
1
186814
1
0
family

1

 0.00
1
1754
genus


 0.00
8
4
526524
1
0
class

4
7

 0.00
526525
0
1
order

1
1
family

 0.00
6
4
128827

0
1
genus
1
2

 0.00
2943184

1

 0.00
1
2899121
species

544447
2

 0.00
3
no rank
0
1

species
2676062
1

 0.00
1

2109692
1
1

 0.00
species

9123

 0.35
611
201174
74
1
phylum


 0.00
9
16
84992
1
0
class

16

 0.00
8
84993
4
1
order

633392

 0.00
7
12
family
1
4

1648491

 0.00
3
6
genus
1
0

2

 0.00
6
2633173
1
1
no rank

species
2849779
5

 0.00
1

3

 0.00
2
467975
1
0
genus

2
2

 0.00
2624035
0
1
no rank

1

 0.00
2
2722752
species

1497346

 0.00
18
14
class
1
2

order
1
3
588673
14

 0.00
11

0
1
family
5
7

 0.00
320583


 0.00
6
5
191494
1
1
genus

2627773
3

 0.00
3
no rank
1
1

species
2937801
1
1

 0.00

species
2812560
1

 0.00
1

191495
2

 0.00
1
species
1
0

469383

 0.00
1
1
strain

1759334
3

 0.00
2
family
1
0

1
0
no rank

 0.00
2
2
2898437

species
2898438
2

 0.00
1

1
0
family

 0.00
3
1
2758916

0
1
genus
1
2

 0.00
2758920

1
1

 0.00
2949664
species

1
0
order
3

 0.00
1
2843198

2843199

 0.00
2
1
family
1
0

genus
2843200

 0.00
1
1

1
0
class

 0.00
6
2
84995

5

 0.00
2
84996
1
0
order

family
0
1
84997
2

 0.00
4

42255
2

 0.00
3
genus
0
1

2653852
1

 0.00
1
species

species
1

 0.00
1
2653851

1
844
class
549

 0.35
9003
1760

0
1
order
9
7

 0.00
1643682

family
1
5
85030

 0.00
6
9

2

 0.00
2
88138
0
1
genus

2
1

 0.00
477641
species

genus

 0.00
1
1
38501

1
0
no rank

 0.00
2
1
234661

species
2596920
1
1

 0.00

order
0
1
85008
72

 0.00
20

28056
72
19

 0.00
family
25
1

genus
5
1
1865
10
6

 0.00

species
113562
1

 0.00
1


 0.00
2
3
2626549
1
2
no rank

species
2721544
1
1

 0.00

196914
1
2

 0.00
species
0
1

strain
1246995
1

 0.00
1

1
1
genus

 0.00
3
6
35753

35754
1

 0.00
4
species

species
1
1

 0.00
53360

1873
31

 0.00
9
genus
18
1

species
1

 0.00
1
356852

47850
1

 0.00
1
species


 0.00
1
3
291594
species

6
3

 0.00
2617518
1
1
no rank

species
2201999
1
1

 0.00

species

 0.00
1
4
2583243

species
709883
1

 0.00
1

species
307121
1

 0.00
1

order
1
0
85011
31

 0.00
122

family
1
5
2062

 0.00
30
122

113
26

 0.00
1883
65
1
genus


 0.00
1
1
2014920
species

species
2

 0.00
1
2763006

species
362257
1

 0.00
1


 0.00
1
1
2653200
species

2593676
16

 0.00
37
no rank
1
6

2907545
1

 0.00
4
species

1855352

 0.00
1
1
species

2
1

 0.00
2923272
species


 0.00
1
1
2833628
species


 0.00
1
1
2801033
species

1964449

 0.00
1
1
species

species
2056258
1

 0.00
1

species
2838850
1

 0.00
1

2879427
1

 0.00
1
species

2750025

 0.00
1
2
species

species

 0.00
1
12
2801029

species
1

 0.00
1
1848900

1

 0.00
1
2981134
species

species
2136173

 0.00
1
1

2742136

 0.00
1
1
species

species
1969
1

 0.00
1

1

 0.00
1
1413221
species

species
285558
1

 0.00
1

species
2496836

 0.00
1
2

species
1

 0.00
1
73044

2063
4
3

 0.00
genus
0
1

2633591
2

 0.00
4
no rank
1
0

species
2742135
4

 0.00
1

178

 0.01
31
2037
0
1
order

1
1
family
178

 0.01
30
2049

0
1
genus
4
2

 0.00
2740557

178339
4

 0.00
1
species

1069494

 0.00
2
1
genus
1
0

1661

 0.00
1
1
species

1
2

 0.00
2767327
0
1
genus

82135
1
1

 0.00
species

genus
1
1
2529408

 0.00
4
19

1
0
no rank

 0.00
2
2
2691889

species
2

 0.00
1
2758572

species
16

 0.00
1
1660

genus
0
1
1522056
1

 0.00
2

species
1

 0.00
1
2758573

1
43
genus

 0.01
17
151
1654

1655
7
1

 0.00
species

1
31
species
2

 0.00
36
544580

5
1

 0.00
871541
strain

species
2737173
1
1

 0.00

1656
1

 0.00
1
species

55565
1

 0.00
1
species

9
1
no rank
62

 0.00
10
2609248

species
2789425
6

 0.00
1

0
1
species
32

 0.00
2
706438

strain
32

 0.00
1
706439

species
6

 0.00
1
712116

species
1

 0.00
4
712122

2

 0.00
1
2789424
species

2

 0.00
2
649739
0
1
species

strain

 0.00
1
2
649743

species
1

 0.00
1
2708340

1
0
order

 0.00
5
11
2039638

2162846

 0.00
4
11
family
1
0

3

 0.00
11
622681
1
2
genus

species
1884904
7

 0.00
1

1884914
1

 0.00
2
species

85014
3

 0.00
8
order
0
1

85034
3

 0.00
7
family
0
1

genus
0
1
58113
2
3

 0.00

2

 0.00
2
2637084
0
1
no rank

species
2867006
2
1

 0.00

genus
0
1
283810
1

 0.00
3

species
1
0
283811

 0.00
2
1

446470

 0.00
1
1
strain

18

 0.00
36
85004
1
0
order

31953
17

 0.00
36
family
1
3

1
4
genus
12

 0.00
20
1678

216816

 0.00
1
3
species

species
4
1

 0.00
1680

77635
1
1

 0.00
species

78448
1

 0.00
2
species
0
1

subspecies
1

 0.00
1
78344

28026

 0.00
1
1
species

1

 0.00
1
1685
species

0
1
species
1
2

 0.00
1683

518635

 0.00
1
1
strain

species
1

 0.00
1
1686

3

 0.00
1
33905
species

1
3
genus
4

 0.00
13
2701

species
2792977
1

 0.00
1

2702

 0.00
1
8
species

species
2792978
1

 0.00
1

1526
136

 0.06
85007
34
1
order


 0.00
4
1
85028
1
0
family

2060
3

 0.00
1
genus
1
0

2061
2

 0.00
1
species
1
0

1

 0.00
1
521096
strain

family
1
11
1653

 0.03
52
679

genus
124
1
1716
668

 0.03
51

species
1
0
152794

 0.00
2
8

strain
196164
1

 0.00
8

1
1

 0.00
156978
species


 0.00
1
10
169292
species

1231000
5

 0.00
2
species
1
1

4

 0.00
1
1408189
strain

1

 0.00
36
43768
species

species

 0.00
1
13
156976

38290
7

 0.00
1
species

0
1
species
2

 0.00
2
349751

1224162

 0.00
1
2
strain

38284
30
1

 0.00
species

species
39791
1

 0.00
1

8

 0.00
1
2594913
species

species
1
1

 0.00
1471400

4

 0.00
2
1404244
0
1
species

strain
1

 0.00
4
1404245

8

 0.00
2
38289
5
1
species

3
1

 0.00
306537
strain

species
43765
4

 0.00
1

species
6

 0.00
1
161899


 0.00
1
5
43770
species

species
3

 0.00
1
1705

1717
1

 0.00
2
species
0
1

1

 0.00
1
2315254
subspecies

1979527
75

 0.00
1
species

species
0
1
160386
3

 0.00
2

strain
3
1

 0.00
1285583

species

 0.00
1
7
1725

0
1
no rank
2
3

 0.00
2624378

2778078

 0.00
1
1
species

702967
1

 0.00
1
species

159
1

 0.01
161879
species

1
1

 0.00
2763010
species


 0.00
1
2
38303
species

38301
5

 0.00
1
species

species
1
1

 0.00
2735136

species
0
1
1121358
3
2

 0.00

558173
1

 0.00
3
strain

species
43769
8

 0.00
1

43990

 0.00
1
22
species

1

 0.00
1
2754725
species

53374
9

 0.00
1
species

species
40

 0.00
1
38304

species
34
1

 0.00
37637

401472

 0.00
1
14
species

1

 0.00
2
1230998
0
1
species

strain
1
1

 0.00
1437875

38288
4
2

 0.00
species
0
1

585529
1

 0.00
4
strain

family
0
1
2805586
9
3

 0.00

9

 0.00
2
1847725
0
1
genus

species
1528099
1

 0.00
9

289
35

 0.01
1762
42
1
family

54
1
genus
178
15

 0.01
1866885

258505
15

 0.00
1
species

species

 0.00
1
2
126673

1795
18

 0.00
1
species

2636767
2

 0.00
13
no rank
1
12

species
1

 0.00
1
2908837

species
1
0
36814
2

 0.00
1

710685
1

 0.00
1
strain

species
1

 0.00
1
1286180

1
0
species
2

 0.00
7
56689

strain
7
1

 0.00
1226753


 0.00
1
2
1799
species

58
1

 0.00
319706
species

1
1

 0.00
1802
species

species
319707
6

 0.00
1

1
17
genus

 0.00
12
43
1763

no rank
0
1
2642494
6
5

 0.00

species
2857060
1

 0.00
1

1

 0.00
1
1547487
species

1561223
1
1

 0.00
species

species
1682113
3
1

 0.00

species
1

 0.00
2
1768

318424

 0.00
1
1
species

species
117567
4
1

 0.00

species
1389713

 0.00
1
7

species
5

 0.00
1
1778

1

 0.00
1
482462
species

0
1
genus
2
2

 0.00
1073531

species
1118379
2

 0.00
1

genus
1
1
670516
5

 0.00
24

species
404941
1
1

 0.00

species

 0.00
1
1
1578165

species
1774
20

 0.00
1

species
36809
1

 0.00
1

family
1
0
85029

 0.00
4
11

genus
1
5
37914
3

 0.00
11

1

 0.00
1
2617939
no rank

species
5
1

 0.00
322509

85025
451

 0.02
30
family
1
1

1
1
genus
11

 0.00
5
1817

species
1824
6

 0.00
1

species
1

 0.00
1
209247


 0.00
2
3
2637762
1
0
no rank

species
1047172
3
1

 0.00

439
24

 0.02
1827
259
1
genus

39
1
species
57
4

 0.00
1828

1443894
1

 0.00
3
strain

strain
1051973

 0.00
1
12

strain
1443893

 0.00
1
3

no rank
1
8
192944
10

 0.00
33

6
1

 0.00
1653478
species

1723645
1

 0.00
2
species

1727214
1

 0.00
1
species

species
1805827
1

 0.00
1

species

 0.00
1
1
644410

species
2795031
2

 0.00
1

species

 0.00
1
2
2880880

species
1045808
8

 0.00
1

species
2054902
2

 0.00
1

species
1

 0.00
1
33008

103816

 0.00
1
2
species

2840174
87
7

 0.00
species group
20
1


 0.00
2
40
334542
1
36
species

4
1

 0.00
1303681
strain

27

 0.00
4
1833
23
1
species


 0.00
1
1
234621
strain

strain
1136179
1

 0.00
2

1
1

 0.00
1289591
strain

family
0
1
85026
52
7

 0.00

genus
3
1
2053
52

 0.00
6

species
1

 0.00
8
2054

84595
29

 0.00
1
species

species
36821
2

 0.00
1

species

 0.00
1
2
2055

species
8
1

 0.00
249058


 0.06
192
1462
85006
1
137
order

16
6

 0.00
85019
0
1
family

1696
16

 0.00
5
genus
3
1

species
33889

 0.00
1
4

species
1

 0.00
2
273384


 0.00
2
7
2614124
1
0
no rank

species
2575923
7

 0.00
1

7

 0.00
3
85017
1
0
family

genus
1
0
186188
4

 0.00
2

species
2509459

 0.00
1
1

species
0
1
186189
1

 0.00
2

strain
446471
1

 0.00
1


 0.00
2
1
157920
1
0
genus

1

 0.00
1
1710
species

family
1
0
145357
8

 0.00
46

genus
0
1
57495
45

 0.00
4

no rank
0
1
2643059
1
2

 0.00

1
1

 0.00
2762331
species

species
1274
44

 0.00
1

1
0
genus

 0.00
3
1
908935

1
0
no rank

 0.00
2
1
2649892

1

 0.00
1
2770551
species

46

 0.00
10
85021
3
1
family

2
3

 0.00
367298
0
1
genus

no rank
0
1
2637926
2

 0.00
2

species
2714941
1

 0.00
2

53457

 0.00
6
41
genus
1
16

species
262209

 0.00
1
2

857417
19

 0.00
1
species

2649294
3

 0.00
4
no rank
1
0

species
2963431

 0.00
1
3

1

 0.00
1
2761047
species

family
1
0
85016

 0.00
4
4

3

 0.00
4
1707
1
1
genus

no rank
1
1

 0.00
2620175

species
2729175
2

 0.00
1

145360
3
4

 0.00
family
0
1

genus
1
0
60919

 0.00
3
3

60920

 0.00
2
3
species
1
0

446469

 0.00
1
3
strain

1
0
family

 0.00
9
22
85020

36739

 0.00
2
5
genus
1
2


 0.00
1
3
1667168
species

43668
17
6

 0.00
genus
4
1

1331682
1

 0.00
1
species

1
0
no rank

 0.00
2
9
2623841

species
9

 0.00
1
2759167

species

 0.00
1
2
2017485

1

 0.00
1
2017484
species

1
2
family

 0.00
6
6
2805590

125287
4
5

 0.00
genus
1
1

1

 0.00
1
2283195
species

2615080
1
2

 0.00
no rank
0
1

species

 0.00
1
1
2934161

species
1
1

 0.00
2594265

1
0
family
3

 0.00
5
85022

5

 0.00
2
43673
0
1
genus

species
43674

 0.00
1
5

487
86

 0.02
85023
276
1
family

55968
2

 0.00
4
genus
1
2

2621730
2

 0.00
1
no rank

4

 0.00
5
2034
1
2
genus

species
2035

 0.00
1
1

257496
2

 0.00
2
no rank
1
0

2862880
1

 0.00
2
species

110932
4

 0.00
3
genus
1
0

1

 0.00
1
150026
species

1
1
no rank
2
2

 0.00
2663824

1798223
1

 0.00
1
species

genus
1
0
337004
4

 0.00
7

2

 0.00
2
2630066
0
1
no rank

2963406

 0.00
1
2
species

1804990
5
1

 0.00
species

no rank
0
1
90316
2

 0.00
2

2880260
2

 0.00
1
species

69578
6

 0.00
5
genus
3
1

2649013
1

 0.00
2
no rank
0
1

species
1897061
1

 0.00
1

2220095

 0.00
1
1
species

species
670052
1

 0.00
1

1
3

 0.00
1649454
0
1
genus

1
0
no rank

 0.00
2
1
2645362

species

 0.00
1
1
2596912

1
1
genus
3

 0.00
2
33877

1
0
no rank

 0.00
2
1
2639701

species
2929802
1

 0.00
1

genus
0
1
96492
3

 0.00
3

2

 0.00
3
2627005
1
0
no rank

2596916
1

 0.00
3
species

18
5

 0.00
190323
4
1
genus

no rank
3
1
2624265
12
3

 0.00

4
1

 0.00
2583822
species

species
1

 0.00
5
2480625

150123
2

 0.00
1
species

0
1
genus
1
2

 0.00
447237

no rank
2626248
1

 0.00
1

genus
0
1
881616
1
3

 0.00

0
1
no rank
1
2

 0.00
2618217

species
2905871
1

 0.00
1

1705353

 0.00
2
1
genus
1
0

species
1
1

 0.00
1987356

110934
14
3

 0.00
genus
0
1

2641148
2

 0.00
14
no rank
1
0


 0.00
1
14
2773266
species

genus
1
0
120212
3

 0.00
1

1
0
no rank
2

 0.00
1
2648727


 0.00
1
1
2846775
species

genus
2
1
33886
8

 0.00
4

no rank
4
1
2609250
5
2

 0.00

1
1

 0.00
2609252
species

110937
1

 0.00
1
species

255204

 0.00
2
1
genus
1
0

species

 0.00
1
1
2820673

11

 0.00
2
1573
5
1
genus

species

 0.00
1
6
1401995


 0.00
22
116
33882
1
54
genus

2782168

 0.00
1
1
species

no rank
1
12
2609290
9

 0.00
35

1906742
1
1

 0.00
species

66354
1
1

 0.00
species

species
2909587
1
1

 0.00

species
1906274
1

 0.00
1

2861281
1

 0.00
1
species

species

 0.00
1
1
2782166

species
2810535
1
1

 0.00

species
16

 0.00
1
2014534

904291
1

 0.00
1
species

species
2
1

 0.00
370764

species

 0.00
1
1
582680

162426
2
1

 0.00
species

species
1
1

 0.00
104336

species
273677
1

 0.00
1

2

 0.00
1
199592
species

species

 0.00
1
8
84292

1526412
1

 0.00
1
species

species
1

 0.00
6
1072463

species
2614638
1

 0.00
1

genus
0
1
235888
2
3

 0.00

no rank
1
1

 0.00
2632331

2079791
1

 0.00
1
species

46352

 0.00
2
1
genus
1
0

1

 0.00
1
684552
species

3
2

 0.00
2680004
1
1
genus

species
1

 0.00
2
2592654

687
48

 0.03
1268
36
1
family

1
2
genus

 0.00
4
4
1742992

no rank
1
0
2634190
3

 0.00
2

species
2979324
1

 0.00
1

species
2735317
1
1

 0.00


 0.01
7
240
1269
1
124
genus

species
566027
2

 0.00
1

1

 0.00
102
1270
species

no rank
1
0
2620948

 0.00
4
12

1

 0.00
1
1179670
species

1
1

 0.00
936902
species

2856555
1

 0.00
10
species


 0.00
10
26
1663
1
7
genus

199136
1

 0.00
1
species

37921
1

 0.00
1
species

5

 0.00
15
235627
1
9
no rank

1
1

 0.00
2830997
species

1

 0.00
1
2017685
species

species
1

 0.00
2
1546536

species
1477518
2
1

 0.00


 0.00
1
1
656366
species

species
1

 0.00
1
2895818


 0.00
5
13
1742989
1
4
genus

2

 0.00
1
1933880
species

species
0
1
256701
6

 0.00
2

strain
6
1

 0.00
861360


 0.00
1
1
37929
species

32207
129
8

 0.00
genus
3
1

species
37923
1

 0.00
7

169480
1

 0.00
1
species

species
12
1

 0.00
172042

species
24
1
2047
29
2

 0.00

strain
5

 0.00
1
762948


 0.00
2
77
43675
1
75
species

680646

 0.00
1
2
strain

1742993
3

 0.00
5
genus
0
1

0
1
no rank
2

 0.00
3
2647000

2590775
1

 0.00
1
species

species
1
1

 0.00
2772401

species
1

 0.00
1
728066

27
1
genus
236
8

 0.01
57493

155

 0.01
1
72000
species

species
71999
1

 0.00
28

1272
13

 0.00
1
species

1049583

 0.00
1
5
species

1
0
no rank
3

 0.00
8
2649579

species
1702043

 0.00
1
1

species
2719588
7
1

 0.00

9
1
order
4675

 0.18
54
85009

27

 0.01
161
85015
1
26
family

genus
0
1
86795
3

 0.00
2

species

 0.00
1
3
642780

1
1
genus
2
2

 0.00
2040

species
1

 0.00
1
2041

2

 0.00
1
116071
1
0
genus

1

 0.00
1
75385
species

0
1
genus
4

 0.00
2
53387

546871
1

 0.00
4
species

genus
1
84
1839
18

 0.00
125

species
1643322

 0.00
1
1

2712223
1
1

 0.00
species

species
1
1

 0.00
2849501


 0.00
1
1
2894081
species

27

 0.00
8
2615069
12
1
no rank

species
1

 0.00
1
196162

1

 0.00
1
2840457
species

2714939
1

 0.00
2
species

2017486

 0.00
1
6
species

2931390
1

 0.00
1
species

2582905
1

 0.00
1
species

2895565

 0.00
1
3
species

402297
1
1

 0.00
species

species
160826
6
1

 0.00

1

 0.00
1
2558918
species

200618
1
1

 0.00
species

2518370

 0.00
1
1
species


 0.17
26
4505
31957
1
3
family

1
1
genus

 0.00
2
2
29404

546874
1

 0.00
1
species

1743
3

 0.00
4
genus
0
1

2

 0.00
2
1744
1
1
species

subspecies
1

 0.00
1
1752

556499
1

 0.00
1
species

1912216

 0.17
11
4490
genus
1
101

1

 0.00
1
1574624
species

1

 0.00
49
33011
species

species
1

 0.00
11
2559073

species
33010
1

 0.00
6

species
4317
1
1747
4322

 0.17
6

strain
1234380
1

 0.00
1

subspecies
1
0
1734925
2

 0.00
2

strain
1114967

 0.00
1
2

0
1
subspecies
2
2

 0.00
1905725

2
1

 0.00
1091045
strain


 0.00
2
1
2801844
1
0
genus

1750

 0.00
1
1
species

genus
1
0
1085622
4

 0.00
4

no rank
1
0
2642922
2

 0.00
3

species
1

 0.00
3
2760308

species
2714937
1
1

 0.00

1912215
2
2

 0.00
genus
1
1

species
1749
1
1

 0.00

order
5
1
85012
21

 0.00
18

2012
8

 0.00
6
family
2
1

1988
6
5

 0.00
genus
1
1

species
46165
1

 0.00
1

2750812
2
1

 0.00
species

2

 0.00
2
2626254
1
1
no rank

1

 0.00
1
2742128
species

2

 0.00
4
2004
1
1
family

2000

 0.00
3
1
genus
1
0

species
0
1
2001
1
2

 0.00

479432
1
1

 0.00
strain

6
7

 0.00
83676
0
1
family

2013
4

 0.00
5
genus
1
1

2014
2
1

 0.00
species

species
1
1

 0.00
2831970

2649073

 0.00
1
1
no rank

104204
2

 0.00
1
genus
1
0

species
2498135

 0.00
1
1

1
5

 0.00
1643684
0
1
order

85031
4

 0.00
1
family
1
0

0
1
genus
1

 0.00
3
53460

1

 0.00
2
53461
0
1
species

strain
479431
1
1

 0.00

0
1
order
43

 0.00
23
85010

43

 0.00
22
2070
15
1
family

7
6

 0.00
1847
1
1
genus

species
2736640

 0.00
1
1

2619320

 0.00
3
4
no rank
1
0

1

 0.00
2
2865833
species

species
445576
2
1

 0.00

37331
1

 0.00
1
species

genus
1
0
43356

 0.00
3
1

1

 0.00
2
2621979
0
1
no rank

1

 0.00
1
2802641
species


 0.00
3
1
1835
1
0
genus

no rank
1
0
2646250

 0.00
2
1

species
2719023
1

 0.00
1

genus
165301
3
1

 0.00

genus
1
1
1813
4

 0.00
4

no rank
1
0
2618356

 0.00
3
3

species

 0.00
1
2
2745196

1
1

 0.00
2653857
species

2071
12

 0.00
4
genus
5
1

species
1
1

 0.00
103733

2

 0.00
6
2593673
1
0
no rank

6
1

 0.00
2781735
species

class
1
0
84998
26

 0.00
13

84999
8
16

 0.00
order
1
1


 0.00
11
4
1643824
1
0
family

genus
1
0
2767353

 0.00
2
1

species
1382

 0.00
1
1

1

 0.00
3
1380
0
1
genus

1
0
no rank

 0.00
2
1
2643817

species
712157
1
1

 0.00


 0.00
5
2
133925
1
0
genus

1

 0.00
2
133926
0
1
species

strain
633147

 0.00
1
1

1
0
no rank

 0.00
2
1
2638792

species
1

 0.00
1
712411

84107
3

 0.00
4
family
0
1

1
0
genus
3

 0.00
3
102106


 0.00
2
3
74426
1
2
species

strain
1

 0.00
1
411903

1643822
5
9

 0.00
order
0
1

1643826

 0.00
8
5
family
1
0

1

 0.00
2
84111
0
1
genus

species
1

 0.00
1
84112

genus
1
1
644652
3

 0.00
4

species
1
0
471189

 0.00
2
1

1
1

 0.00
657308
strain

species
1335613
1
1

 0.00

genus
1
1

 0.00
2815775

1752188
1
2

 0.00
no rank
0
1

species
1

 0.00
1
1848255

544448

 0.00
37
24
phylum
1
0

2790996
1
1
order

family
1
1
2895623

genus
2995234
1
1


 0.00
1
1
2096
species

class
0
1
31969
23
35

 0.00

2085
14

 0.00
11
order
1
0

2092
13

 0.00
11
family
1
0

genus
1
0
2129

 0.00
2
1


 0.00
1
1
2130
species

genus
0
1
2923352
2
4

 0.00

species

 0.00
1
1
29562

2

 0.00
1
2128
1
0
species

1

 0.00
1
743971
strain

1
0
genus
4

 0.00
7
2093

species

 0.00
1
6
754517

65123
1
2

 0.00
species
0
1


 0.00
1
1
1197325
strain

0
1
genus
1

 0.00
2
2767358

species
33923
1
1

 0.00

1
0
order
7

 0.00
3
186328

family
0
1
33925
1

 0.00
3

46239
1
2

 0.00
genus
0
1

species
216427
1

 0.00
1

2131

 0.00
3
2
family
1
0

2132
2
2

 0.00
genus
1
1

216934
1

 0.00
1
species

1
0
order

 0.00
13
9
186329

2146
9
12

 0.00
family
0
1

3

 0.00
3
2147
1
0
genus

2148
1

 0.00
1
species

species

 0.00
1
2
35623

2

 0.00
2
2903107
1
0
genus

species
61635

 0.00
1
2

0
1
genus
4
6

 0.00
33926

species group
1
0
85630
2

 0.00
2

37692

 0.00
1
2
species

85632
3

 0.00
2
species group
1
0

species
59748
1
1

 0.00


 0.00
1
1
69896
species


 0.00
24
36
32066
1
0
phylum


 0.00
2
1
69842
1
0
no rank

2970471
1
1

 0.00
species

0
1
class
35
21

 0.00
203490

order
1
0
203491
20

 0.00
35

203492

 0.00
10
23
family
1
0

genus
1
8
848
9

 0.00
23

860
1

 0.00
1
species


 0.00
3
7
851
1
5
species

1

 0.00
1
155615
subspecies

subspecies
76857
1

 0.00
1

861

 0.00
1
1
species

5

 0.00
1
2663009
species

0
1
species
1
2

 0.00
850

strain
1
1

 0.00
469616

1129771

 0.00
9
12
family
1
0

1
0
genus
8

 0.00
12
32067


 0.00
1
3
157687
species

species

 0.00
1
1
109328

species
157688
2
1

 0.00

2

 0.00
1
40542
1
0
species

strain
1
1

 0.00
523794


 0.00
2
5
2633022
1
0
no rank

1

 0.00
5
712357
species

phylum
1
0
200918

 0.00
6
2

188708

 0.00
5
2
class
1
0

order
1
0
2419

 0.00
4
2

1643950
3

 0.00
2
family
1
0

2

 0.00
2
2422
1
0
genus

species

 0.00
1
2
548918

40117
6

 0.00
1
phylum
1
0

1
5

 0.00
2811502
0
1
class


 0.00
4
1
2811503
1
0
order

family
1
0
2811505

 0.00
3
1

1
2

 0.00
2811506
0
1
genus

species
2715679
1
1

 0.00

2179
418

 0.08
1783270
2
1
clade


 0.00
2
1
62680
1
0
phylum

1
1

 0.00
2026760
species

65842
1
3

 0.00
phylum
0
1

1

 0.00
2
1805148
0
1
no rank

species
2052160
1
1

 0.00

142182
6

 0.00
2
phylum
1
0

0
1
no rank
1
2

 0.00
234665

species

 0.00
1
1
2026742

1

 0.00
3
219685
0
1
class

0
1
order
1
2

 0.00
219686

family
219687

 0.00
1
1

68336
2171

 0.08
402
clade
5
1

2150

 0.08
380
976
114
1
phylum

353
37

 0.01
117747
0
1
class


 0.01
36
353
200666
1
0
order

84566

 0.01
35
353
family
1
22

423349
11

 0.00
52
genus
1
30

species
1

 0.00
1
2305508

3

 0.00
1
652787
species

862126
3

 0.00
1
species

species
1

 0.00
2
2740462

species
1550579
1
1

 0.00

species
1234841
2

 0.00
1


 0.00
3
9
2617802
1
2
no rank

species

 0.00
1
3
2703789

4
1

 0.00
1300914
species

species

 0.00
1
1
2027860

7

 0.00
5
28453
2
1
genus

258
1

 0.00
2
species

1
1

 0.00
28454
species

1
0
no rank

 0.00
2
2
2609468

1538644
2

 0.00
1
species

1
0
genus
3

 0.00
2
376469

0
1
no rank
2
2

 0.00
2632301

2592345
2

 0.00
1
species

84567
15

 0.01
270
genus
1
25

188932
110
1

 0.00
species

species

 0.00
1
2
332999

species
430522
1

 0.00
1


 0.00
1
10
336820
species


 0.00
1
1
425514
species

2628915
6

 0.00
117
no rank
1
15

2

 0.00
1
1727164
species

2714940
3

 0.00
1
species

2856523
1

 0.00
78
species

1

 0.00
16
2578106
species

3
1

 0.00
2762321
species

species
363852
1

 0.00
1

species
2605747
1
1

 0.00

2
1

 0.00
2766984
species

0
1
class
1268

 0.05
167
117743

1268

 0.05
166
200644
35
1
order

1
0
family

 0.00
3
1
1333713

1

 0.00
2
2828338
0
1
genus

1

 0.00
1
2761580
species

0
1
family
1
3

 0.00
39782

34098
1
2

 0.00
genus
0
1

species
1

 0.00
1
1653831

2762318
504

 0.02
52
family
11
1

3

 0.01
369
501783
1
76
genus

1

 0.01
241
2004710
species


 0.00
1
52
237258
species

1778601

 0.00
3
1
genus
1
0

1
2

 0.00
2630820
0
1
no rank

1

 0.00
1
2704651
species

0
1
genus
4

 0.00
2
1013

species
4
1

 0.00
1014

2
1
genus
6
4

 0.00
308865

1756149

 0.00
1
1
species

1117645
1

 0.00
2
species

species
1
1

 0.00
238

genus
0
1
34084
2

 0.00
2

2

 0.00
1
34085
species

9

 0.00
3
59734
0
1
genus


 0.00
1
1
247
species

343874

 0.00
1
8
species

2782232
102
34

 0.00
no rank
12
1

2782229

 0.00
4
13
genus
1
0


 0.00
1
1
421072
species


 0.00
1
10
2487072
species

species
1416779
1

 0.00
2

genus
1
29
59732

 0.00
25
67

2898076

 0.00
1
1
species

species
2852098
1
1

 0.00

2547600

 0.00
1
1
species

species
1

 0.00
1
2754694

species

 0.00
1
1
536441

1124835
1

 0.00
1
species

2593645
10

 0.00
22
no rank
1
6

species
2713414
4
1

 0.00

1721091
1

 0.00
2
species

species
2478663

 0.00
1
4

2825845
1

 0.00
1
species

species
1

 0.00
1
2724619

2879939
1

 0.00
1
species

species
1

 0.00
1
2879938

species
2838877

 0.00
1
1

species
2487065
1

 0.00
1

species
254
1

 0.00
1

2956784

 0.00
1
1
species

2929800
1

 0.00
2
species

1
1

 0.00
1493872
species

253

 0.00
1
1
species

species
1
1

 0.00
2497456

species
1685010
1

 0.00
2

species
1

 0.00
1
2929799


 0.00
4
10
2782231
1
1
genus

species
2
1

 0.00
421525

1241979

 0.00
1
1
species

species
1

 0.00
6
266748

0
1
family
1

 0.00
3
1853230


 0.00
2
1
332102
1
0
genus

species
1798018

 0.00
1
1

family
134
1
49546
726
104

 0.03

1016
19
9

 0.00
genus
4
1

species
1017
2

 0.00
1

species
1

 0.00
1
28188

3

 0.00
3
2640652
0
1
no rank

species
1

 0.00
2
1316596

1

 0.00
1
2545799
species

45243

 0.00
1
1
species

species

 0.00
1
5
1019

species

 0.00
1
3
327575

1

 0.00
2
61432
0
1
no rank

1
1

 0.00
531844
species

3
2

 0.00
76831
0
1
genus

3
1

 0.00
256
species

0
1
genus
1

 0.00
3
225842

0
1
no rank
1
2

 0.00
2644710

species
1798225
1

 0.00
1


 0.00
2
3
252356
1
0
genus

species
320912
3

 0.00
1

111500

 0.00
2
1
genus
1
0

species
1

 0.00
1
1383885

417127
3
3

 0.00
genus
0
1

1
0
no rank

 0.00
2
3
2632541

2779359

 0.00
1
3
species

1
2

 0.00
358023
0
1
genus


 0.00
1
1
1622118
species

genus
1
1

 0.00
2058174

genus
1
0
326319

 0.00
3
1

no rank
1
0
2615033
2

 0.00
1

983548

 0.00
1
1
species


 0.00
4
2
286104
1
0
genus

2615021
3

 0.00
2
no rank
1
0

species

 0.00
1
1
754409


 0.00
1
1
2929510
species

genus
393005
1
1

 0.00

1

 0.00
3
283735
0
1
genus

1

 0.00
2
2615029
0
1
no rank

2719912
1

 0.00
1
species

genus
0
1
104267
7
4

 0.00

species
104268
2

 0.00
1

species
1850252
4

 0.00
1

no rank
1
1

 0.00
2635139

290174

 0.00
4
2
genus
1
0

no rank
1
0
2627091

 0.00
3
2

species
1714860
1

 0.00
1

species
1
1

 0.00
1714849


 0.00
3
1
291183
1
0
genus

2647285
2

 0.00
1
no rank
1
0

species
983544
1

 0.00
1

genus
0
1
261827
1
2

 0.00

species
1
1

 0.00
1736674


 0.00
4
4
143222
1
1
genus

1
0
no rank

 0.00
3
3
2633436

1729720
1
1

 0.00
species

species
1903072
1

 0.00
2

283785
3

 0.00
1
genus
1
0

no rank
1
0
2626393
2

 0.00
1

species
1

 0.00
1
2675331

genus
1
256
237
41

 0.02
532

species
1
1

 0.00
2895947

2547394
1

 0.00
3
species

species
1

 0.00
1
1751056

species
1114867
4
1

 0.00

species
1
1

 0.00
1871076

55197
1
2

 0.00
species
0
1

1

 0.00
1
1034807
strain

species
998845
1

 0.00
1

2654843
1

 0.00
2
species

2816357
4
1

 0.00
species

species

 0.00
1
1
1114861

species
3
1

 0.00
1306519

species
1751095
4

 0.00
1

2

 0.00
2
312277
0
1
species

strain
1094466
2

 0.00
1

1

 0.00
1
683124
species

1

 0.00
2
2518177
species


 0.01
21
237
196869
1
84
no rank

2981990
1

 0.00
2
species

species
239
1

 0.00
4

species
2746225
1

 0.00
1

species

 0.00
1
14
2739062

species
2845819
1

 0.00
3

2893884
2

 0.00
1
species


 0.00
1
5
2871165
species

2893885
1
1

 0.00
species

species
1

 0.00
1
907046

species
1

 0.00
13
2893886

species
1
1

 0.00
2294119

1

 0.00
4
1979344
species

species
1
1

 0.00
2893883

1

 0.00
9
2724135
species

species
1

 0.00
1
2748319

species
10
1

 0.00
2478552

species

 0.00
1
6
2665645

species
1
1

 0.00
2654325

species
59
1

 0.00
1179672

935222

 0.00
1
15
species

1

 0.00
1
2704140
species

96345
7
1

 0.00
species

3

 0.00
3
153265
1
0
genus

species
1
0
101385

 0.00
2
3

1

 0.00
3
746697
strain

2
1
genus
3

 0.00
2
52959


 0.00
1
1
1908341
species

class
1
0
1853228
17

 0.00
16

16
16

 0.00
1853229
0
1
order

563835
16

 0.00
15
family
6
1

3
3

 0.00
79328
1
1
genus

1
1
no rank
2
2

 0.00
2619133

1

 0.00
1
2853437
species

1
2

 0.00
2698688
0
1
genus


 0.00
1
1
2315862
species

1
2

 0.00
2836216
0
1
genus

species
1

 0.00
1
2676868

genus
0
1
504481
1
3

 0.00

no rank
0
1
2635961
1

 0.00
2

1
1

 0.00
575378
species

1874621
2

 0.00
2
genus
1
0

species
1813871

 0.00
1
2

569836

 0.00
2
2
no rank
1
0

species
1869212

 0.00
1
2

0
1
class
91
52

 0.00
768503

51

 0.00
91
768507
1
5
order

family
0
1
563798
3

 0.00
7

2

 0.00
3
232244
0
1
genus

1
0
species

 0.00
2
2
232259

strain
1

 0.00
2
866536

0
1
genus
1
3

 0.00
68288

1
0
species

 0.00
2
1
104

strain
880070
1
1

 0.00


 0.00
2
1
1751870
1
0
no rank

1945892
1

 0.00
1
species

1
0
family

 0.00
9
5
2896860

1
0
genus

 0.00
3
2
105

106
2

 0.00
2
species
1
0

strain
1

 0.00
2
761193

genus
0
1
2173039
1
2

 0.00

species
1

 0.00
1
1784714

120831
2
3

 0.00
genus
0
1

2625061
2

 0.00
2
no rank
1
1

species
1

 0.00
1
2909339

1
2
family
12

 0.00
48
1853232

18
1
genus
46
11

 0.00
89966

1

 0.00
1
1484116
species

species
1
1

 0.00
1850093

1705399
1

 0.00
1
species

species
3
1

 0.00
2502781

14
1
no rank
22

 0.00
6
2615202

2932250

 0.00
1
2
species

species

 0.00
1
1
2932248


 0.00
1
3
1356852
species

species
2584940
1
1

 0.00

species
2675878
1

 0.00
1

89373
25
14

 0.00
family
0
1


 0.00
4
10
2676247
1
3
genus

species
2

 0.00
1
2696560

1

 0.00
3
2516557
species

species
1

 0.00
2
2516559

1

 0.00
3
978
0
1
genus

0
1
species
1

 0.00
2
985

1

 0.00
1
269798
strain

1
4
genus

 0.00
6
14
107

species
564064

 0.00
1
3

1
1

 0.00
2666025
species

1379870
1

 0.00
1
species

2621999

 0.00
2
5
no rank
1
2

species

 0.00
1
3
2710596

0
1
family
4

 0.00
6
2762286

2

 0.00
1
1433993
1
0
genus

2321403
1
1

 0.00
species

396811
3

 0.00
3
genus
1
0

no rank
0
1
2685541
3
2

 0.00

2904245
3

 0.00
1
species

303
98

 0.01
200643
0
1
class

2

 0.00
5
1970189
0
1
order

2

 0.00
4
558415
0
1
family

0
1
genus
2

 0.00
3
286729

2
2

 0.00
2644571
0
1
no rank

species
2949635
1

 0.00
2

order
1
11
171549

 0.01
92
301

1
0
family
6

 0.00
5
171551

no rank
1
0
361210
2

 0.00
2

species
1

 0.00
2
2049046

1
1
genus
3

 0.00
3
836

species
1
1

 0.00
322095

species
1

 0.00
1
28124

0
1
family
6
6

 0.00
171550

1
1
genus

 0.00
5
6
239759

328813
1

 0.00
3
species

no rank
0
1
2608932
1

 0.00
2

2662363

 0.00
1
1
species

1
1

 0.00
1288121
species

family
0
1
2005525
15
8

 0.00

195950

 0.00
2
1
genus
1
0

712710

 0.00
1
1
species

375288
14

 0.00
5
genus
3
1

species
1
5
823

 0.00
2
6

999416

 0.00
1
1
strain

species
1
1

 0.00
387661

species
46503
1

 0.00
4

815
153

 0.01
25
family
1
1

genus
9
1
909656
59

 0.00
7


 0.00
1
3
387090
species

12
2

 0.00
357276
11
1
species

strain
997877
1

 0.00
1

species
1
0
376805
2

 0.00
1

strain
667015
1

 0.00
1

821

 0.00
1
34
species

816
93
17

 0.00
genus
28
1

species

 0.00
1
13
246787

1

 0.00
2
329854
species

species
2650157
2

 0.00
1

1

 0.00
3
371601
species

species
674529

 0.00
1
2

11
1
species
13

 0.00
2
820

2

 0.00
1
997890
strain

2646097
2

 0.00
1
no rank
1
0

species
1
1

 0.00
556259


 0.00
1
10
818
species

46506

 0.00
2
4
species
1
3

strain
1

 0.00
1
449673

species
28116
7

 0.00
1

47678

 0.00
1
4
species

817
3
1

 0.00
species

species
1

 0.00
1
28111

no rank
1
0
185291

 0.00
2
3

1400053
1

 0.00
3
species

171552
74
18

 0.00
family
0
1

genus
5
1
838
74
17

 0.00

species
3
1

 0.00
28133

470565
1

 0.00
1
species

species
18

 0.00
1
28132

species

 0.00
1
1
28137

species
28131
1

 0.00
1

2
1

 0.00
1177574
species

2

 0.00
1
28129
species

2

 0.00
3
2638335
0
1
no rank


 0.00
2
2
652716
1
0
species

strain
2
1

 0.00
575614

species
1

 0.00
1
839

1
0
species
2

 0.00
3
52227

strain
908937

 0.00
1
3

1
20
species
2

 0.00
31
165179

strain
537011
1

 0.00
11

species
4

 0.00
1
28135

2005523
2
4

 0.00
family
0
1

346096
2

 0.00
3
genus
0
1

0
1
species
2

 0.00
2
185300

2
1

 0.00
694427
strain

2005520
11

 0.00
23
family
1
1

2

 0.00
4
307628
1
0
genus

species
4

 0.00
1
1642646

2
2

 0.00
1784836
0
1
genus

1562970
2

 0.00
1
species

0
1
no rank
12

 0.00
2
2811463


 0.00
1
12
2811465
species

294702

 0.00
4
4
genus
1
0

species

 0.00
1
2
2829812

species
1642647
1

 0.00
2

no rank

 0.00
1
0
2622718

family
0
1
1853231
7
6

 0.00

574697
3

 0.00
2
genus
1
0

species
1
1

 0.00
2093856

1
1

 0.00
544645
species

283168
5

 0.00
2
genus
0
1

28118

 0.00
1
5
species

0
1
family
2

 0.00
5
2005473


 0.00
2
1
2518495
1
0
genus

1

 0.00
1
2649562
no rank

0
1
no rank
1
2

 0.00
2006847

species
1
1

 0.00
2767078

class
0
1
1937959
4

 0.00
6

1936988
4

 0.00
5
order
0
1

1937961

 0.00
4
4
family
1
0

2349
4

 0.00
3
genus
0
1

1
0
species
2

 0.00
4
2350

760192
1

 0.00
4
strain


 0.00
2
1
37452
1
0
no rank

1690483
1

 0.00
1
species

0
1
phylum
5
11

 0.00
1134404

795747
5
10

 0.00
class
0
1

1
0
no rank

 0.00
2
1
1798416

1
1

 0.00
2053306
species

1
0
order
7

 0.00
4
795748

0
1
no rank
3
2

 0.00
1951140

3

 0.00
1
2049428
species

0
1
family
1
4

 0.00
795749

genus
1
0
795750
3

 0.00
1

591197

 0.00
2
1
species
1
0

1

 0.00
1
945713
strain

11
10

 0.00
1090
0
1
phylum

191410

 0.00
7
2
class
1
0

2

 0.00
6
191411
0
1
order

191412
2

 0.00
5
family
0
1


 0.00
4
2
274493
1
0
no rank

1
0
genus
3

 0.00
2
1091


 0.00
2
2
1096
1
0
species

strain

 0.00
1
2
290317

no rank
1
0
44765

 0.00
2
9

species
1

 0.00
9
2268192

phylum
0
1
456828
2
4

 0.00

0
1
genus
2
3

 0.00
456826

456827
2

 0.00
2
species
1
0

459349

 0.00
1
2
strain

1
1
no rank
11

 0.00
51
48479

1181531
1
1

 0.00
species

species
1
1

 0.00
1623951

1

 0.00
1
136703
species

species
1447235

 0.00
1
1

species
1

 0.00
1
1805587

1781351
1

 0.00
1
species

1

 0.00
1
1256569
species

species
1624413

 0.00
1
1

species
506514
1

 0.00
1

species
77133

 0.00
1
41

0
1
phylum
1

 0.00
3
74152

0
1
no rank
1
2

 0.00
1797919

2030800
1

 0.00
1
species

10563
1328

 0.41
1224
953
1
phylum

298
1
class
1701
330

 0.07
28211


 0.02
64
475
204457
1
80
order

123
1
family
388

 0.01
57
41297

1
0
genus
3

 0.00
1
1649486

1
0
species
2

 0.00
1
160791

1

 0.00
1
392499
strain

8
6

 0.00
165696
3
1
genus

158500
1

 0.00
2
species

3

 0.00
4
2644732
0
1
no rank

species
2571749

 0.00
1
1

702113

 0.00
1
1
species

1

 0.00
1
2025349
species

0
1
no rank
4
3

 0.00
122612

2
1

 0.00
2952887
species

2
1

 0.00
2952888
species

genus
87
1
13687
212

 0.01
27

species
1
1

 0.00
2714944

species
1

 0.00
1
1549858

363835
1

 0.00
1
species

species
11

 0.00
1
185951

1

 0.00
4
2759526
species

2938948
1

 0.00
1
species

species
1327635
2

 0.00
1


 0.00
1
1
653931
species

species

 0.00
1
1
1609977

1560345

 0.00
1
6
species

1
2
species

 0.00
2
4
152682

strain

 0.00
1
2
621456

no rank
36
1
196159
91
12

 0.00

2872652
1
1

 0.00
species

1390395
1

 0.00
1
species

species
1517551

 0.00
1
1


 0.00
1
9
1523415
species

species
3

 0.00
1
2972485

1
1

 0.00
2937787
species

species
1

 0.00
1
1961362

species
2
1

 0.00
2653203

species
1

 0.00
1
2596913

10
1

 0.00
1517554
species

species

 0.00
1
25
2565555

0
1
species
1
2

 0.00
397260

1

 0.00
1
1123269
strain

165695
23

 0.00
10
genus
7
1

2611147
6
6

 0.00
no rank
1
1

species
1
1

 0.00
2733867

species
2082188
1
1

 0.00

species
2676077
1

 0.00
1

1

 0.00
1
2565554
species

species
520734

 0.00
1
1

species
76947
1

 0.00
1

13690

 0.00
1
8
species

species
1

 0.00
1
121428

8
1
genus
15

 0.00
5
165697

0
1
no rank
5
3

 0.00
2614943


 0.00
1
1
2486273
species

4
1

 0.00
2976533
species

1515612

 0.00
1
2
species

1434046
2

 0.00
2
genus
1
0


 0.00
1
2
392610
species

7
6

 0.00
335929
4
1
family

0
1
genus
1

 0.00
2
1295327

1348774
1

 0.00
1
species

2

 0.00
3
361177
0
1
genus

1
0
no rank
2

 0.00
2
2614945

2067415
1

 0.00
2
species

order
162
1
356
571
156

 0.02

0
1
family
1

 0.00
3
45404

1156568

 0.00
2
1
genus
1
0

species
1
1

 0.00
569860

2036754
3

 0.00
1
family
1
0

genus
0
1
28209
1

 0.00
2

species
444444
1

 0.00
1

2831100
5

 0.00
6
family
1
0

0
1
genus
6
4

 0.00
85413

species

 0.00
1
2
1526658

4
2

 0.00
2653178
3
1
no rank

species
2015316
1

 0.00
1

family
1
0
31993

 0.00
3
1

1
2

 0.00
133
0
1
genus

173366
1

 0.00
1
species

82115
50

 0.00
24
family
14
1

0
1
genus
1

 0.00
2
323620

species
1

 0.00
1
2871820

227290
31
16

 0.00
no rank
7
1

6
1
genus
16
7

 0.00
379

451876
1

 0.00
1
species

species
1

 0.00
1
384

2613769
8
4

 0.00
no rank
4
1

species

 0.00
1
1
2895568

species
1

 0.00
1
1435607

2
1

 0.00
2815360
species

genus
1
1
357
7
5

 0.00

species
2735528
3

 0.00
1

species
1

 0.00
1
28099

2

 0.00
2
1183400
0
1
species group

species
358

 0.00
1
2

1
0
genus

 0.00
3
1
1525371

2629175

 0.00
2
1
no rank
1
0

species
1825976
1
1

 0.00

227292

 0.00
5
4
no rank
1
0

1
1
genus
4
4

 0.00
28105

194963
1

 0.00
2
species

2

 0.00
1
663276
1
0
species group

species
380

 0.00
1
1


 0.00
7
5
255475
1
0
family

1
0
genus
3

 0.00
1
182269

no rank
1
0
2638230
2

 0.00
1

2906072
1

 0.00
1
species

genus
3
1
414371
4

 0.00
3

0
1
no rank
1
2

 0.00
2615206

2816454
1

 0.00
1
species

0
1
family
1

 0.00
2
772

773
1

 0.00
1
genus

43
12

 0.00
118882
0
1
family

43

 0.00
11
2826938
31
1
no rank

234
10

 0.00
6
genus
1
1

no rank
0
1
2632610
1

 0.00
2

1
1

 0.00
2986424
species

94625

 0.00
1
1
species

271865
3

 0.00
1
species

species
529
4
1

 0.00

528
2

 0.00
4
genus
0
1

0
1
no rank
2
3

 0.00
239106

2708351
1

 0.00
1
species

species
2696486
1

 0.00
1

655351
1
4

 0.00
family
0
1

1
0
genus
3

 0.00
1
655352

no rank
1
0
2631913
2

 0.00
1

1798205

 0.00
1
1
species

7

 0.00
3
45401
1
0
family

3

 0.00
2
29407
1
0
genus

2619116
2

 0.00
2
no rank
0
1

species
674703

 0.00
1
2

81
3

 0.00
1
genus
1
0

species
0
1
53399
1

 0.00
2

strain

 0.00
1
1
582899

1
0
family

 0.00
3
2
2831090

genus
0
1
59282
2
2

 0.00

species
1079

 0.00
1
2

1
0
family

 0.00
4
2
335928

genus
1
1
556257
2
3

 0.00

no rank
1
0
2638514

 0.00
2
1

species
2562284
1

 0.00
1

1

 0.00
4
2813035
0
1
family

1
0
genus
3

 0.00
1
444432

0
1
no rank
1

 0.00
2
2614933

1922226

 0.00
1
1
species

1
40
family

 0.01
25
168
41294

3
1
genus
6
3

 0.00
1395974

no rank
2631404
1

 0.00
2

1
1

 0.00
943830
species

374
15

 0.00
118
genus
1
84

2631580
23

 0.00
7
no rank
13
1

species
2898149

 0.00
1
3

species

 0.00
1
1
2782609

2782641
1
1

 0.00
species

2

 0.00
1
2782665
species

species
2
1

 0.00
376

1

 0.00
1
1404888
species

species
722472
1

 0.00
2

1404864

 0.00
1
1
species

species

 0.00
1
1
1437360

375
1

 0.00
1
species

species
2840469
3
1

 0.00

species
244734
1

 0.00
2

species
1

 0.00
1
1325107

3

 0.00
4
1073
0
1
genus

species
475937
1
1

 0.00


 0.00
2
2
2638247
1
0
no rank

2
1

 0.00
340268
species

no rank
0
1
81426
1
2

 0.00

species

 0.00
1
1
2072420

0
1
no rank
2

 0.00
4
119042

genus
0
1
212791
2

 0.00
3

no rank
0
1
2632691
2

 0.00
2


 0.00
1
2
1894999
species

1
13
family
23

 0.00
81
119045

407
52

 0.00
16
genus
24
1

species
0
1
31998
2
2

 0.00

426355
2
1

 0.00
strain

species
374432

 0.00
1
1

418223

 0.00
1
2
species

0
1
species
1

 0.00
2
39956

strain
908290
1
1

 0.00

1
0
species

 0.00
2
1
334852

1

 0.00
1
693986
strain

species

 0.00
1
1
269660

14
5

 0.00
2615210
4
1
no rank

2603276
1

 0.00
1
species

species
2984843
2
1

 0.00

1

 0.00
1
426117
species

2953579
6

 0.00
1
species

species
570505

 0.00
1
6

genus
1
3
186650
3

 0.00
12

species

 0.00
1
8
2651334

1882682

 0.00
1
1
species

2282523
4

 0.00
3
genus
1
1

1
1
species
2

 0.00
3
223967

strain
441620
1

 0.00
2

family
1
1
2831106
23

 0.00
7

46913
6

 0.00
22
genus
1
14

no rank
1
1
196773
5

 0.00
4

1

 0.00
2
1736675
species

2917991

 0.00
1
1
species

2806348
1

 0.00
1
species

species
3

 0.00
1
2801335

69277
15

 0.00
18
family
1
3

1

 0.00
3
31988
0
1
genus

0
1
no rank
1
2

 0.00
2644704

species
374606
1

 0.00
1

genus
1
0
245876

 0.00
3
2

no rank
1
0
2641084
2

 0.00
2

2599600
1

 0.00
2
species

5
1
genus
10

 0.00
6
68287

1
1
species
2
2

 0.00
381

935548
1

 0.00
1
strain

3

 0.00
3
325217
1
1
no rank

species
2744523
1

 0.00
1

species
1

 0.00
1
2483404

genus
1
1
28100
2

 0.00
2

species
1867719

 0.00
1
1

no rank
0
1
47925
1
2

 0.00

species

 0.00
1
1
40872

54526

 0.00
4
2
order
1
0

0
1
family
2
3

 0.00
1655514

1
0
no rank
2

 0.00
2
1655516


 0.00
1
2
2026775
species

766
18

 0.00
19
order
0
1

5

 0.00
5
942
1
0
family

4

 0.00
2
952
0
1
tribe

genus
4

 0.00
1
953

no rank
0
1
84676
1

 0.00
2

1

 0.00
1
2704616
species

1699067

 0.00
2
1
no rank
1
0

species

 0.00
1
1
1528098

1
0
family

 0.00
11
12
775

0
1
tribe
12
10

 0.00
33988

12
9

 0.00
780
0
1
genus

114295
1

 0.00
2
no rank
0
1

species
1
1

 0.00
931099

2

 0.00
1
1129742
1
0
species group


 0.00
1
1
33990
species

1
0
species group
4

 0.00
10
114277

786
8

 0.00
2
species
0
1

293614
8

 0.00
1
strain

35788

 0.00
1
2
species

25

 0.00
99
204458
1
0
order

99

 0.00
24
76892
13
1
family

75

 0.00
8
39
genus
1
27

no rank

 0.00
1
1
2648921

2010972

 0.00
1
1
species

species
1

 0.00
2
1679497

species
69395

 0.00
1
1

2708539
1

 0.00
1
species

1

 0.00
1
155892
species

species
1

 0.00
5
88688

20

 0.00
6
8
genus
1
2

2640670
1

 0.00
2
no rank
0
1

2903901
1

 0.00
1
species

species
1
0
284016
2

 0.00
1

strain
450851
1

 0.00
1

species
1

 0.00
4
2803784

23
1
genus
39
9

 0.00
41275

species
1325724
1

 0.00
1

1

 0.00
1
172043
species

2774190
3

 0.00
1
species

species
41276

 0.00
1
1

1
0
species
2

 0.00
3
74313

3

 0.00
1
633149
strain

1

 0.00
2
293
species

5
1

 0.00
2622653
no rank

order
0
1
2800060
1

 0.00
4

family
1
0
69657

 0.00
3
1

genus
1
0
2892997
2

 0.00
1

1

 0.00
1
1759059
species

order
18
1
204455
113
23

 0.00

31989
91

 0.00
14
family
12
1

265
78
11

 0.00
genus
20
1

10

 0.00
1
147645
species

1
1
no rank
17
3

 0.00
2688777

species
1

 0.00
1
2589076

species
15
1

 0.00
2500532

16
1

 0.00
59779
species

2560053

 0.00
1
4
species

1
1

 0.00
1945662
species


 0.00
1
1
135740
species

1545044
1

 0.00
7
species

species
2903900
2

 0.00
1

2

 0.00
1
1209946
1
0
genus


 0.00
1
1
564137
species

1
0
family
8

 0.00
4
2854170

60136
1
3

 0.00
genus
0
1

no rank
1
0
196795
2

 0.00
1


 0.00
1
1
2070369
species

2
1

 0.00
74030
genus

1
3

 0.00
263377
0
1
genus

2

 0.00
1
2640570
1
0
no rank

species
1

 0.00
1
1792508

order
1
1
204441
123

 0.00
32

no rank
1
0
941843
2

 0.00
2

2026786
2

 0.00
1
species

family
0
1
41295
3

 0.00
5

genus

 0.00
1
1
2705399

genus
1

 0.00
1
13134

0
1
no rank
1

 0.00
2
41296

species

 0.00
1
1
1898112

5
1
family
52

 0.00
16
433

1434011

 0.00
2
7
genus
1
0

species
1

 0.00
7
265959

1
21
genus
7

 0.00
35
125216

2

 0.00
1
257708
1
0
species

subspecies
204527
1

 0.00
1

2768162
1

 0.00
1
species

no rank
0
1
2617492
1
2

 0.00

2897332
1

 0.00
1
species

207340
11
1

 0.00
species

434
5
6

 0.00
genus
2
1

1
0
subgenus

 0.00
3
1
151157

species
0
1
435
1

 0.00
2

strain
1

 0.00
1
887700

1

 0.00
1
446692
species

species
1

 0.00
1
438

family
1
1
2829815
65

 0.00
8

62
5

 0.00
204447
6
1
genus

2632462
51
2

 0.00
no rank
0
1

1

 0.00
51
2775420
species

species
1789672

 0.00
1
3

species
1817965
2

 0.00
1

1
1
genus
2

 0.00
2
191

species

 0.00
1
1
2970906

68525
88

 0.01
189
subphylum
1
0

0
1
class
164
51

 0.01
29547


 0.01
50
164
213849
1
4
order

0
1
family
4

 0.00
5
72293


 0.00
4
4
209
1
0
genus

123841
1

 0.00
1
species

species
76936
2
1

 0.00

1

 0.00
1
210
species

family
22
1
2808963
135

 0.01
23

genus
33
1
28196
60
9

 0.00

no rank
0
1
2593671
1
2

 0.00

species
2961520
1
1

 0.00

1849015

 0.00
1
8
species

species
1

 0.00
1
913109

0
1
species
10

 0.00
2
1278212

strain
663365
10

 0.00
1

species
1

 0.00
1
1054034

1054033
1

 0.00
6
species

1
2

 0.00
2321115
0
1
genus

1

 0.00
1
1462615
species

3

 0.00
2
2321114
1
1
genus

species
1
0
1032072
2

 0.00
1

1
1

 0.00
870501
strain

8

 0.00
50
2321111
1
3
genus

species
28197
1

 0.00
2

species
255507
1

 0.00
2

species
2

 0.00
1
1564138

species
1
30
28198
2

 0.00
34

strain
1032070
4
1

 0.00

7
2

 0.00
28200
6
1
species

strain
1032239

 0.00
1
1

0
1
family
3
4

 0.00
2932623

57665
3

 0.00
3
genus
2
1

species
0
1
194424
1

 0.00
2

1
1

 0.00
1193502
strain

0
1
family
1
4

 0.00
2771472

genus
0
1
265570
1
3

 0.00

no rank
0
1
2646778
1
2

 0.00

species
1

 0.00
1
387093

72294

 0.00
13
17
family
1
0

genus
1
1
194

 0.00
12
17

197

 0.00
1
1
species

species
1

 0.00
3
204

1

 0.00
1
827
species

species
195
1
1

 0.00

2

 0.00
1
2593542
no rank

species
201

 0.00
1
1

196
2
2

 0.00
species
0
1


 0.00
1
2
32020
subspecies

species
3
1

 0.00
199

species
824
1
1

 0.00

76517

 0.00
1
1
species

1
3
class
36

 0.00
25
28221

order
1
4
29
19

 0.00
16

6

 0.00
5
80811
1
3
suborder

family
31
1

 0.00
1

family
0
1
39
1
4

 0.00

genus
1
0
44
3

 0.00
1

species
1
0
83453
2

 0.00
1

1
1

 0.00
1294270
strain

80812

 0.00
12
7
suborder
1
2

0
1
no rank
2
3

 0.00
215910

1
0
genus
2

 0.00
2
1649470

species
888845
1

 0.00
2

family
0
1
1524216
1
3

 0.00

1524217
2

 0.00
1
genus
1
0

species
1391654

 0.00
1
1


 0.00
5
2
49
1
0
family

1

 0.00
2
55
0
1
genus

1

 0.00
1
2567896
species

1
2

 0.00
39643
0
1
genus

species
56
1
1

 0.00

order
1
0
213113
5

 0.00
1

117942

 0.00
4
1
family
1
0

84404
1

 0.00
3
genus
0
1

1
0
species
2

 0.00
1
84405

1
1

 0.00
760142
strain

4
8

 0.00
69541
0
1
order

1
2
family
7

 0.00
4
213422

3

 0.00
1
2910589
1
0
genus

0
1
species
1
2

 0.00
351604

1

 0.00
1
351605
strain

1

 0.00
3
28231
0
1
genus

1
0
no rank
2

 0.00
1
2627627

species
1
1

 0.00
2597769

1
0
order
3

 0.00
1
213115

family
0
1
194924
1

 0.00
2

1
1

 0.00
2910984
genus

550

 0.19
4977
1236
1
206
class

135615

 0.00
6
6
order
1
0

1
0
family
5

 0.00
6
868

0
1
genus
6
4

 0.00
2717

species
2718
1

 0.00
5

no rank
0
1
2648856
1

 0.00
2

2866573
1
1

 0.00
species

order
1
0
2887326
70

 0.08
2142

468
69

 0.08
2142
family
1
38

2

 0.00
1
54393
1
0
no rank

species
1
1

 0.00
1889775

2824158
1
2

 0.00
genus
0
1

2283318
1

 0.00
1
species

506
36

 0.02
469
274
1
genus

species group
1
15
909768
5

 0.00
53

species
48296
4
1

 0.00

species

 0.00
1
32
470


 0.00
1
1
1530123
species

species
106654
1
1

 0.00

1
1

 0.00
2006115
species

106649
3
1

 0.00
species

5
1
no rank
30
14

 0.00
196816

species

 0.00
1
1
1758189

species
2905879
1
1

 0.00

1

 0.00
1
2079596
species

species
1
1

 0.00
2563897

2798861
1
1

 0.00
species

species
1407071

 0.00
1
2

species
1646498
6
1

 0.00

species
2853158
1

 0.00
3

5
1

 0.00
2743575
species

2925837

 0.00
1
1
species

species

 0.00
1
1
2004644


 0.00
1
1
2545797
species

species
2725684
1

 0.00
1

2053287

 0.00
1
6
species

species
28090
15
1

 0.00

species
51
1
40214
55

 0.00
2

strain
1242245
1

 0.00
4

1

 0.00
1
52133
species

1789224
1

 0.00
1
species

5

 0.00
1
108981
species

13

 0.00
1
108980
species

465797
15

 0.00
1
species

12
1

 0.00
29430
species

1839785
1

 0.00
2
species


 0.00
1
8
756892
species

species
40216
4
1

 0.00

species
8
1

 0.00
40215

5
1
genus
974

 0.04
7
475

34062
845

 0.03
1
species


 0.00
2
1
2685852
1
0
no rank

species
2904122

 0.00
1
1

116

 0.00
2
480
115
1
species

strain
1236608

 0.00
1
1


 0.00
1
7
478
species

622
21

 0.02
497
260
1
genus

species
1

 0.00
6
45610

1
187
no rank

 0.01
14
323
196806

1

 0.00
7
571800
species

species
349857

 0.00
1
1

species
1028420
1
1

 0.00

species
1

 0.00
5
1699624

2565531
1
1

 0.00
species

species
18
1

 0.00
1028416

2607668
1
1

 0.00
species

species
2733866

 0.00
1
1

23
1

 0.00
56811
species


 0.00
1
58
2708350
species

2983299
1

 0.00
8
species

1

 0.00
2
2772254
species

species
1699622

 0.00
1
10


 0.00
1
20
330922
species

species

 0.00
1
1
256325

0
1
species
10
2

 0.00
334543

10

 0.00
1
259536
strain

1

 0.00
2
861445
species


 0.00
6
2
118884
1
0
no rank


 0.00
3
1
32036
1
0
clade

genus
0
1
2732587
1
2

 0.00


 0.00
1
1
2763321
no rank

1

 0.00
2
1076628
0
1
clade

1076629
1

 0.00
1
species

135619
114
30

 0.00
order
1
1

4

 0.00
1
135620
1
0
family

188907
3

 0.00
1
genus
1
0

1
0
species

 0.00
2
1
188908

1

 0.00
1
698738
strain

1
15
family
22

 0.00
111
28256

1
49
genus

 0.00
18
95
2745

species
1

 0.00
1
507626

115555

 0.00
1
1
species

1

 0.00
2
2746
0
1
species

strain
1

 0.00
1
768066

2497861
9
1

 0.00
species

29
8

 0.00
2609666
20
1
no rank

species
2982692
1
1

 0.00

1
1

 0.00
2982694
species

2854257
1

 0.00
1
species

species
2

 0.00
1
2730360

species
1
1

 0.00
2855441

species
2306583
2
1

 0.00

species
2733487
1

 0.00
1

2733488

 0.00
1
3
species

97916
1

 0.00
2
no rank
0
1

173971
1

 0.00
1
species

species
1

 0.00
1
1897729

1
0
genus

 0.00
3
1
42054

0
1
species
1
2

 0.00
158080

290398

 0.00
1
1
strain


 0.00
3
1
224372
1
0
family

1
1
3020832
genus

1

 0.00
1
1094342
species


 0.00
1
0
59753
genus

135624
39

 0.00
16
order
0
1

84642
39

 0.00
15
family
0
1

1
15
genus

 0.00
11
38
642

species
644
2
1

 0.00

9
1
species
10

 0.00
3
645

subspecies
1
0
96473

 0.00
2
1

1324960
1

 0.00
1
strain

73010
1

 0.00
1
species

species
1114880
1

 0.00
1

species
648
1

 0.00
3

species
4
1

 0.00
651

2

 0.00
2
257493
1
1
no rank

species

 0.00
1
1
2778058

1
0
genus
3

 0.00
1
43947

43948
1

 0.00
2
species
0
1

strain
1
1

 0.00
595494

1692040
1
4

 0.00
order
0
1

1
3

 0.00
1692041
0
1
family

2

 0.00
1
1692042
1
0
genus

species
1

 0.00
1
1675686

order
0
1
72273
3

 0.00
8

34064
1
3

 0.00
family
0
1

262
1
2

 0.00
genus
0
1

species
573570

 0.00
1
1

135616
2

 0.00
4
family
0
1

1

 0.00
1
34067
genus

genus
0
1
933
1
2

 0.00

species
406020
1

 0.00
1

33811

 0.00
2
1
no rank
1
0

650377
1

 0.00
1
species

order
0
1
135623
20
15

 0.00

20
14

 0.00
641
0
1
family

genus
1
0
511678

 0.00
2
2

species
2

 0.00
1
80852

662
15

 0.00
8
genus
1
1

no rank
1
0
2614977
2

 0.00
1

1

 0.00
1
2883075
species

1
1

 0.00
296199
species

212663
1

 0.00
1
species

676
5
1

 0.00
species

species
672
1

 0.00
5

1

 0.00
1
675
species

genus
0
1
657
3

 0.00
3

species
1
0
74109
2

 0.00
3

298386
3

 0.00
1
strain

156

 0.07
1723
72274
1
3
order

3

 0.00
1
2887365
1
0
family

1
0
genus

 0.00
2
1
2742

species

 0.00
1
1
1033846


 0.07
152
1719
135621
1
33
family

8
4

 0.00
2901189
1
1
genus

136844
1
2

 0.00
species group
0
1

1

 0.00
1
43306
species

species
797277
6

 0.00
1

2901261
1
2

 0.00
genus
0
1

1
1

 0.00
521720
species

2901164
17

 0.00
5
genus
0
1

1
1
species group
17

 0.00
4
136846

0
1
species subgroup
16
3

 0.00
578833

1
15
species
2

 0.00
16
316

1

 0.00
1
644801
strain

1
893
genus
136

 0.06
1658
286

species
2745514

 0.00
1
2

no rank
0
1
62104
1

 0.00
2

species
1

 0.00
1
114707

species
2745510
1

 0.00
1


 0.00
9
24
136849
1
6
species group

36746
1
1

 0.00
species

species
33069

 0.00
1
8

6
4

 0.00
251695
0
1
species subgroup

317
6

 0.00
3
species
4
1

1
1

 0.00
1332075
strain

no rank
1
1

 0.00
199201


 0.00
1
1
46257
species

2
1

 0.00
1190415
species


 0.00
1
1
702115
species

species
0
1
101564
1

 0.00
2

strain

 0.00
1
1
741155

1691904

 0.00
1
2
species

1

 0.00
1
2842354
species

1
1

 0.00
658629
species

46677
1

 0.00
1
species

species
359110
1

 0.00
8

species
53407

 0.00
1
2

species
47886

 0.00
1
1

species
2842349

 0.00
1
4

species
2493633

 0.00
1
2

515393
4

 0.00
1
species

2906062
1

 0.00
3
species

237610
1

 0.00
27
species

1

 0.00
2
216142
species

158627
1
1

 0.00
species

species group
1
1
136841
29

 0.00
7

1
1

 0.00
300
species

species
14

 0.00
1
287

1

 0.00
2
53408
species


 0.00
2
9
1232139
1
0
species subgroup

species
9
1

 0.00
301

species

 0.00
1
2
43263

136843
275
22

 0.01
species group
27
1

200452

 0.00
1
1
species

75588

 0.00
1
2
species

185

 0.01
4
294
133
1
species

1
1

 0.00
1037911
strain

216595

 0.00
1
50
strain

strain
1

 0.00
1
1221522

651740
1

 0.00
1
species

species

 0.00
1
1
380021

species
76758
26

 0.00
1

species
75612
1
1

 0.00

species
169669

 0.00
1
1

2
1

 0.00
47878
species

species
1
3
76761

 0.00
2
6

3
1

 0.00
1295141
strain

species
1

 0.00
7
200451

4
1

 0.00
76760
species

species
1

 0.00
2
47883

2

 0.00
1
29442
species

species
1

 0.00
1
78543

species
129817
3
1

 0.00

species
200450
3
1

 0.00

136842
19

 0.00
3
species group
0
1

species
1

 0.00
1
86185

1

 0.00
18
296
species

species
1

 0.00
2
198620

2745518

 0.00
1
23
species

species group
1
2
136845
6

 0.00
54

species
303
32

 0.00
1

2
1
species
3
2

 0.00
47880

strain
1
1

 0.00
743720

47885
15
1

 0.00
species

species
2
1

 0.00
76759

species
1

 0.00
1
319939

1

 0.00
2
47884
species


 0.00
1
2
1274359
species

196821
53

 0.01
241
no rank
1
78

1206777
1

 0.00
1
species

1
1

 0.00
2866278
species

2870750
1
1

 0.00
species

2052956

 0.00
1
1
species

species
1
1

 0.00
1207075

species
1886807
1
1

 0.00

species

 0.00
1
1
2018067

species
349859
1

 0.00
1

3

 0.00
1
2590776
species

species
2605424
23

 0.00
1

1283291
1

 0.00
5
species

2898483
2
1

 0.00
species

species
2604941
21

 0.00
1

1573711
2
1

 0.00
species

species
1

 0.00
1
1294143

species
2974552
8

 0.00
1

species
2
1

 0.00
1344094

species
1855380

 0.00
1
1

species

 0.00
1
2
2054914

species
2825975

 0.00
1
1

species
2083054

 0.00
1
1

2479392
1
1

 0.00
species

1534110

 0.00
1
1
species

species
2862945

 0.00
1
1

species
1

 0.00
1
1573718

species
1

 0.00
1
2895487

2730847

 0.00
1
1
species

species
2735906
1

 0.00
1

2498848
1

 0.00
4
species

species
658644

 0.00
1
1

1

 0.00
3
2054919
species

1930532

 0.00
1
1
species

species
2219057

 0.00
1
2

species
1

 0.00
6
1827300


 0.00
1
1
1636610
species

species

 0.00
1
7
253237

species

 0.00
1
1
1573720

species
2749807

 0.00
1
2

species
1758730
1

 0.00
3

2895486
1
1

 0.00
species

species
1

 0.00
2
2654238

2320270
1

 0.00
1
species

2025658
1

 0.00
1
species

306
1

 0.00
1
species

species
2804761

 0.00
1
3

2926671
1
1

 0.00
species

2879114
1
1

 0.00
species


 0.00
1
1
1573712
species

species
2971912
3

 0.00
1

species
2745519
1

 0.00
28

species
1

 0.00
1
2073078

species
2895473
1

 0.00
1

7
1

 0.00
2867264
species


 0.00
1
2
1853130
species

86265

 0.00
1
1
species

1
1

 0.00
437900
species

species
321846
2
1

 0.00

1

 0.00
1
489632
species

1

 0.00
1
2961896
species

species
2745511
1

 0.00
1

species
12

 0.00
1
122355

1
0
subfamily

 0.00
4
2
351

352
3

 0.00
2
genus
1
0


 0.00
2
2
353
1
1
species

1328314

 0.00
1
1
strain

1

 0.00
5
2887327
0
1
order

0
1
family
1

 0.00
4
1920240

genus
1
0
261963
3

 0.00
1

species
0
1
261964
1

 0.00
2

strain
523791
1

 0.00
1

135613

 0.00
12
4
order
1
0

72276

 0.00
4
1
family
1
0

genus
0
1
1335745
1

 0.00
3

0
1
no rank
1

 0.00
2
2629425

1
1

 0.00
2666185
species

1046
3

 0.00
1
family
1
0

no rank
0
1
82569
1

 0.00
2

2049432
1
1

 0.00
species

family
0
1
255526
2

 0.00
4

109262

 0.00
3
2
genus
1
0

species
1
0
927
2

 0.00
2

2

 0.00
1
555778
strain

135618
6
10

 0.00
order
0
1

9

 0.00
6
403
1
0
family

genus
0
1
429
1
3

 0.00

0
1
no rank
1
2

 0.00
2635283

1

 0.00
1
2839024
species

genus
0
1
762296
3

 0.00
2

3

 0.00
1
1704499
species

413
1

 0.00
2
genus
0
1

1

 0.00
1
414
species

416
1

 0.00
1
genus

7

 0.00
2
118969
1
0
order


 0.00
3
1
118968
1
0
family

no rank
0
1
134284
1
2

 0.00

species
1
1

 0.00
2026727

0
1
family
1
3

 0.00
444

genus
1
0
445
2

 0.00
1

1

 0.00
1
446
species

33

 0.00
40
135622
0
1
order

1
0
family

 0.00
5
2
267891


 0.00
4
2
58050
1
0
genus

2

 0.00
1
2637987
1
0
no rank

2746230

 0.00
1
1
species

species
80854
1

 0.00
1

1
0
family

 0.00
5
2
72275

1
0
no rank

 0.00
2
1
2903219

1

 0.00
1
226
genus

1172191
1
2

 0.00
genus
0
1

species
1

 0.00
1
1740262

family
0
1
267889
6

 0.00
6

28228

 0.00
5
6
genus
1
0

196834
6
4

 0.00
no rank
0
1

2497879
2
1

 0.00
species

species

 0.00
1
1
1816219

species
1

 0.00
3
2583805

267893
1
3

 0.00
family
0
1

135575

 0.00
2
1
genus
1
0

species
1

 0.00
1
1940690

267890
18

 0.00
12
family
0
1

22

 0.00
11
18
genus
1
7

species

 0.00
1
1
2590884

1

 0.00
1
43661
species

332186

 0.00
1
1
species

species
518738
1

 0.00
1

1

 0.00
1
38313
species

6
5

 0.00
196818
0
1
no rank

species
1
1

 0.00
2989727

species
351745
1
1

 0.00

1

 0.00
1
2864212
species

2029986
3
1

 0.00
species

267894
1

 0.00
4
family
0
1

genus
0
1
67572
1

 0.00
3

357794
1
2

 0.00
species
0
1

strain
1
1

 0.00
357804

267888

 0.00
4
3
family
1
0

1
2
genus

 0.00
3
3
53246

no rank
1
0
194690
2

 0.00
1

species

 0.00
1
1
2822846

1
0
order
9

 0.00
5
1706369


 0.00
3
3
1706371
1
1
family

10
2

 0.00
2
genus
0
1

2
1

 0.00
2624793
no rank

family
1
0
1706373
5

 0.00
2

48073
4

 0.00
2
genus
1
0

1
0
no rank
2

 0.00
1
2619833

species
2587856

 0.00
1
1

1

 0.00
1
48074
species

135614
44

 0.01
227
order
1
14

family
50
1
32033
208
41

 0.01

genus
37
1
40323
88
13

 0.00

3
1
species group
39
7

 0.00
995085

1
0
no rank

 0.00
2
1
2961925

species
1

 0.00
1
2072405

species
31
1
40324
35
4

 0.00


 0.00
1
2
391008
strain

strain
1163399

 0.00
1
1

1

 0.00
1
868597
strain

species
7
1

 0.00
216778

196198
5
4

 0.00
no rank
0
1

species
2770322
1
1

 0.00


 0.00
1
3
1904944
species

1
1

 0.00
2962886
species

genus
1
1
83614

 0.00
4
8

species
2006110

 0.00
1
2

species
1176533

 0.00
1
4

2901869
1

 0.00
1
species

genus
1
11
338

 0.00
7
39

5
1

 0.00
2643310
no rank

species
56455

 0.00
1
1

339
2

 0.00
17
species
1
15

340
2

 0.00
1
no rank

3
1
species
5
2

 0.00
343

no rank
1

 0.00
2
227946

genus
0
1
2370
1

 0.00
2

species
1
1

 0.00
2371

1
3
genus

 0.00
4
6
141948

1463158
2
1

 0.00
species

0
1
no rank
1
2

 0.00
2633315

species
2771436
1
1

 0.00


 0.00
4
7
68
1
3
genus

2698682
1
1

 0.00
species

435897

 0.00
1
2
species

species
453783
1
1

 0.00

4
1
genus
9
6

 0.00
83618

2645906
3
3

 0.00
no rank
0
1

species
2
1

 0.00
2758562


 0.00
1
1
2571115
species

2

 0.00
2
314722
1
1
species

743721
1
1

 0.00
strain

1
4
family

 0.00
2
5
1775411

242605
1

 0.00
1
genus

1
21
order
91

 0.01
376
91347

family
1
0
1903412
3

 0.00
1

1
0
genus
2

 0.00
1
568

1

 0.00
1
569
species

543
261
46

 0.01
family
180
1

genus
1
0
579
2

 0.00
1

1

 0.00
1
61648
species

561
3

 0.00
10
genus
1
1

species
208962
1
1

 0.00

562
1

 0.00
8
species

2055880
2

 0.00
2
genus
0
1

566
2

 0.00
1
species

genus
1
0
1081630

 0.00
2
1

1081631

 0.00
1
1
species

2

 0.00
1
447792
1
0
genus

species
1972431
1

 0.00
1

2815296
2

 0.00
1
genus
1
0

species
2579935
1
1

 0.00

4

 0.00
3
83654
1
1
genus

2627398
2

 0.00
3
no rank
1
0

species
3

 0.00
1
2714951

12

 0.00
4
544
5
1
genus

1

 0.00
2
1344959
0
1
species group

species

 0.00
1
1
546

species
6
1

 0.00
545

191675
3
7

 0.00
no rank
0
1

clade
0
1
84563
3

 0.00
6

1449912

 0.00
2
1
genus
1
0

1345115
1

 0.00
1
species

1
0
clade
3

 0.00
2
84564

genus
0
1
1699619
2
2

 0.00

2
1

 0.00
1594731
species

82976

 0.00
2
1
genus
1
0

species
1

 0.00
1
82977

158483

 0.00
3
1
genus
1
0

1
0
no rank
2

 0.00
1
2649846

2545798

 0.00
1
1
species

2890311
23

 0.00
8
no rank
1
1

genus
1
1
160674
2

 0.00
2

577
1
1

 0.00
species

20

 0.00
5
570
13
1
genus

species
1
1

 0.00
1463165

1

 0.00
1
1134687
species

no rank
2608929
1

 0.00
1

4
1

 0.00
573
species

547
21

 0.00
5
genus
12
1


 0.00
4
9
354276
1
6
species group

species
1

 0.00
1
550

species
158836
1

 0.00
1

1

 0.00
1
208224
species

11

 0.00
12
1903414
0
1
family

2
2

 0.00
581
0
1
genus

species
582

 0.00
1
2

genus
0
1
626
1
2

 0.00

species
628
1

 0.00
1

genus
1
1
583
5
4

 0.00


 0.00
1
3
183417
species

2

 0.00
1
257482
1
0
no rank

1
1

 0.00
2883107
species

1
1
genus
3
3

 0.00
586

587
1

 0.00
1
species

species
126385
1

 0.00
1

family
1
1
1903409
31
16

 0.00

53335
9

 0.00
21
genus
1
8

2
1

 0.00
472695
species

1
1

 0.00
470934
species

species
1

 0.00
1
553

59814

 0.00
1
4
species

species group
1
0
1654067

 0.00
2
4

species
549

 0.00
1
4


 0.00
2
1
2630326
1
0
no rank

592316
1

 0.00
1
species

32199
1
2

 0.00
genus
0
1

species
9
1

 0.00
1

genus
1
1
3026546

1619313
1
1

 0.00
species

2
1
genus
7

 0.00
3
551

species
55212
3
1

 0.00

182337
2

 0.00
1
species

1903411
51

 0.00
13
family
2
1

genus
4
1
34037
6

 0.00
4

0
1
no rank
1
2

 0.00
2635087

species
1
1

 0.00
657337


 0.00
1
1
34038
species

39
5

 0.00
613
28
1
genus


 0.00
1
5
615
species

300181
1
1

 0.00
species

1

 0.00
2
28151
species

2597702

 0.00
1
3
species

4
3

 0.00
629
2
1
genus

species
1

 0.00
1
630

631
1

 0.00
1
species

1
0
order

 0.00
13
64
135625

family
1
1
712
64
12

 0.00

genus
1
4
724
5

 0.00
51

species
727

 0.00
1
1

species
42
1
729
45
2

 0.00

strain

 0.00
1
3
862965

species
726
1

 0.00
1

genus
2
1
416916
12

 0.00
6

2

 0.00
3
732
1
2
species

strain
1
1

 0.00
985008

2639383
3

 0.00
7
no rank
1
1

species
3
1

 0.00
712150

2866570

 0.00
1
3
species

1240482

 0.00
5
2
order
1
0

1240483
2
4

 0.00
family
0
1

genus
0
1
1193503
2
3

 0.00

2
2

 0.00
2685620
0
1
no rank

species
2704654

 0.00
1
2

580370
1

 0.00
5
class
0
1

0
1
order
1

 0.00
4
580371

0
1
family
1

 0.00
3
580372

0
1
genus
1

 0.00
2
377315

1

 0.00
1
1921087
species

349

 0.11
2735
28216
1
157
class

2381
235

 0.09
80840
471
1
order


 0.03
83
749
80864
1
391
family

665874

 0.00
4
9
genus
1
0

9

 0.00
3
2626134
0
1
no rank

species
1678128
1

 0.00
6

species
1678129
1

 0.00
3

genus
0
1
364316
2
3

 0.00

364317
2
2

 0.00
species
0
1

391735

 0.00
1
2
strain

genus
1
26
80865
4

 0.00
36

species
558537
1
1

 0.00

species
80866
7

 0.00
1

species
180282
2
1

 0.00

1
0
no rank
3

 0.00
17
83494

species
2893879
1
1

 0.00

1

 0.00
16
2952996
species

1
9
genus
8

 0.00
25
283

1

 0.00
1
363952
species

species
225991
6
1

 0.00

2638500
1

 0.00
2
no rank
0
1

species
2966554

 0.00
1
1

379895
3
1

 0.00
species

1
4
species

 0.00
2
5
285

strain
1
1

 0.00
1392005

10

 0.00
33
28065
1
11
genus

species
1484693
5

 0.00
1

2509614
2

 0.00
1
species

species
1842727
1

 0.00
5

2527691

 0.00
1
1
species

2627954
9
5

 0.00
no rank
0
1

2741720
1

 0.00
1
species

species

 0.00
1
5
2752316

species
2798804
1
1

 0.00

2822760
2
1

 0.00
species

6
1
genus
18

 0.00
6
47420

2610897
10
4

 0.00
no rank
7
1

1842537
1
1

 0.00
species

species
795665
1
1

 0.00

species
2806347
1

 0.00
1

species

 0.00
1
2
47421

genus
1
1
238749
17
6

 0.00

species
1288495
9
1

 0.00


 0.00
4
7
2649760
1
2
no rank

species

 0.00
1
2
2792224

species
1

 0.00
1
2735554

species
2714924
1

 0.00
2

1
16
genus

 0.00
12
47
12916

species
1

 0.00
1
2743470

553814
1

 0.00
4
species

1
6
no rank

 0.00
8
25
2684926

species
1858609
1

 0.00
1

358220

 0.00
1
5
species

species

 0.00
1
2
232721

species
2478662
1

 0.00
2

1842533
1

 0.00
3
species

species
2518343
1

 0.00
5

2714923

 0.00
1
1
species

1
1

 0.00
758826
species

genus
0
1
352450
10

 0.00
2

2109915
1

 0.00
10
species

1

 0.00
3
281915
genus

219181
9

 0.00
4
genus
0
1

2109914
8

 0.00
1
species

2645081

 0.00
2
1
no rank
1
0

1658672

 0.00
1
1
species

0
1
genus
1
3

 0.00
174951

2617605
1
2

 0.00
no rank
0
1

species
1

 0.00
1
2732511

genus
1
5
52972
8

 0.00
33

no rank
11
1
2638319
15
5

 0.00

species
2895564
1

 0.00
1


 0.00
1
1
2895563
species

1

 0.00
1
296591
species

species
1840293

 0.00
1
1

2

 0.00
13
216465
1
0
species

13
1

 0.00
365044
strain

1
39
genus

 0.00
8
98
34072

34073

 0.00
1
18
species

species
436515

 0.00
1
21

no rank
1
12
663243

 0.00
5
20

species
1795631
2
1

 0.00

2774875
4

 0.00
1
species

species
2762322
1

 0.00
1

species
1

 0.00
1
2681552

1
0
family
5

 0.00
2
995019

1
0
genus
4

 0.00
2
40544

286132
2

 0.00
1
no rank
1
0

286133
1

 0.00
1
species

species

 0.00
1
1
40545

224471
9

 0.00
12
no rank
1
2

3

 0.00
1
318147
1
0
genus

0
1
no rank
1
2

 0.00
2642959

1768242

 0.00
1
1
species

0
1
genus
2
3

 0.00
212743

no rank
1
0
2640088

 0.00
2
2

species

 0.00
1
2
2753607

genus
0
1
114248
7

 0.00
2

7

 0.00
1
307486
species

1
161
family

 0.03
54
660
75682

1
6
genus
3

 0.00
9
202907


 0.00
2
3
158899
1
2
species

strain
1005048
1
1

 0.00

29580
210
12

 0.01
genus
145
1

no rank
1
9
2610881

 0.00
6
25

3

 0.00
1
1644131
species

species
1938606
2

 0.00
1

species
1

 0.00
3
2861284


 0.00
1
1
375286
species

species

 0.00
1
7
1537274

13

 0.00
1
2590869
species

species

 0.00
1
10
368607

29581

 0.00
1
7
species

species
5
1
55508
10

 0.00
2

1349767

 0.00
1
5
strain

2895353
140
19

 0.01
no rank
41
1

genus
1
0
75654

 0.00
2
4

species
2728021
4

 0.00
1

1522432
2

 0.00
3
genus
1
2

species
1

 0.00
1
2072590

1
54
genus

 0.00
14
92
149698

1
19
no rank
9

 0.00
31
2609279

species
1707785

 0.00
1
1

2861283

 0.00
1
1
species

1

 0.00
1
2970464
species

species
1

 0.00
1
1337838

2769491
1
1

 0.00
species

1

 0.00
1
1593482
species

species
1
1

 0.00
2852099

1

 0.00
5
2861282
species

species
2765360

 0.00
1
1

4
1

 0.00
2045208
species

species
1

 0.00
1
2728020

species
1

 0.00
1
945844

0
1
genus
5

 0.00
5
963

0
1
no rank
3

 0.00
2
2624150

species
2025949
3

 0.00
1

341045
2

 0.00
2
species
1
0

1

 0.00
2
1262470
strain

genus
1

 0.00
1
303379


 0.00
4
85
119059
1
67
no rank

2

 0.00
1
2955018
species

species
4
1

 0.00
2955019

2955020
1

 0.00
12
species

0
1
genus
1
3

 0.00
1229970

1
2

 0.00
2644401
0
1
no rank

species
1
1

 0.00
2807626

genus
3
1
401469
48
6

 0.00

2630295

 0.00
3
16
no rank
1
7


 0.00
1
5
2058624
species

2058625
4

 0.00
1
species

401471
14
1

 0.00
species


 0.00
1
15
2495591
species

119060
390
45

 0.02
family
19
1

genus
15
1
44013
36
10

 0.00

species
2

 0.00
1
1743165


 0.00
7
16
2640945
1
9
no rank

1

 0.00
1
2770234
species

1

 0.00
1
2081052
species

2576926
1

 0.00
1
species

species

 0.00
1
2
1855910

species

 0.00
1
1
1855880

species
1758390
1

 0.00
1

1

 0.00
3
576610
species

genus
1
2
32008
10

 0.00
11

1
0
species group

 0.00
8
8
87882

species
1
0
60552

 0.00
2
1


 0.00
1
1
269482
strain

species
1

 0.00
1
179879

1
1

 0.00
488729
species

species
3
1

 0.00
87883

species
95485
1
1

 0.00

species
482957
1
1

 0.00

species
1

 0.00
1
337

genus
3
1
106589
7
5

 0.00

68895
1

 0.00
2
species

0
1
no rank
1
2

 0.00
2640874

species
876364
1

 0.00
1

96344

 0.00
1
1
species

1
0
no rank
2

 0.00
1
132490

2030806
1
1

 0.00
species

1
0
genus

 0.00
2
1
93217

1

 0.00
1
445709
species

8

 0.00
3
1822464
5
1
genus

species
134537

 0.00
1
2

2026199

 0.00
1
1
species

0
1
genus
8
2

 0.00
47670

species

 0.00
1
8
47671

155
2

 0.01
157932
0
1
genus

species

 0.01
1
155
29443

genus
6
1
48736
142
6

 0.01

105219
3

 0.00
1
species

305
1
1

 0.00
species

126

 0.00
1
190721
species

species
1
5
329

 0.00
2
6

strain
1366050
1

 0.00
1


 0.00
2
2
240411
1
0
genus

species
356302
2
1

 0.00

2975441
13
17

 0.00
family
4
1

genus
1
0
316612
2

 0.00
1

2633235
1
1

 0.00
no rank

1
1
genus

 0.00
3
2
93681

2

 0.00
1
2626991
1
0
no rank

species
1

 0.00
1
2978473

0
1
genus
2
2

 0.00
36862

species
2

 0.00
1
36863

3

 0.00
1
28067
1
0
genus

28068
1
2

 0.00
species
0
1

983917
1

 0.00
1
strain

1
1
196013
genus

species
1

 0.00
1
215580

391952
1

 0.00
2
genus
0
1

1

 0.00
1
391953
species

1

 0.00
2
34102
0
1
genus

1
1

 0.00
639200
species

genus
1

 0.00
0
215579

family
1
1
506
84

 0.00
21

genus
1
0
1148163

 0.00
3
1


 0.00
2
1
2631622
1
0
no rank

species
1
1

 0.00
2901142

507
2
3

 0.00
genus
0
1

259357
2
2

 0.00
no rank
0
1

species
2813780
2

 0.00
1

4
3

 0.00
90243
0
1
genus

species
90244
1

 0.00
1

3
1

 0.00
90245
species

222
68

 0.00
6
genus
32
1

17
1
species
18
2

 0.00
85698

strain
762376

 0.00
1
1

1
1

 0.00
217203
species

species
1353891
1

 0.00
16

species
32002
1
1

 0.00

0
1
genus
6
2

 0.00
152267

1940612

 0.00
1
6
species

2
3

 0.00
290425
0
1
genus

1
0
species
2

 0.00
2
302406


 0.00
1
2
1247726
strain

order
1
0
206351

 0.00
40
89

family
1
1
1499392
8

 0.00
13


 0.00
1
1
230666
genus

0
1
genus
2
3

 0.00
187

no rank
0
1
2620219
2

 0.00
2

species
2
1

 0.00
1938604

885864
3

 0.00
1
genus
1
0

no rank
1
0
2619593
2

 0.00
1

species
1009682
1
1

 0.00


 0.00
3
2
90153
1
0
no rank

2
2

 0.00
32014
0
1
genus

2

 0.00
1
537
species


 0.00
2
1
57479
1
0
genus


 0.00
1
1
57480
species

481
81
26

 0.00
family
8
1

0
1
genus
1
2

 0.00
2593687

1

 0.00
1
2067065
species

1
0
genus

 0.00
2
1
59

63
1
1

 0.00
species

genus
0
1
32257
1

 0.00
2


 0.00
1
1
502
species

4
2

 0.00
538
0
1
genus

1

 0.00
4
539
species

212742

 0.00
2
1
genus
1
0

species
1

 0.00
1
1056807

genus
1
29
482

 0.00
15
65

28449
13
1

 0.00
species

4
1

 0.00
488
species

species
1
0
487

 0.00
3
1

serogroup
1
0
135720

 0.00
2
1

strain
1
1

 0.00
374833

2623750
3
4

 0.00
no rank
0
1

species
655307

 0.00
1
1

2
2

 0.00
641148
0
1
species

641149
2
1

 0.00
strain

1

 0.00
5
490
species

species
1
0
496
2

 0.00
3

997348
1

 0.00
3
strain

7

 0.00
2
495
6
1
species

88719
1

 0.00
1
subspecies

1
5
order
26

 0.00
71
206389

family
1
1
2008795
8

 0.00
7

73029
5

 0.00
5
genus
1
0

0
1
no rank
3
3

 0.00
2633351

species

 0.00
1
2
2654218

1

 0.00
1
2231055
species

species
281362

 0.00
1
2

0
1
genus
1

 0.00
2
88875

76259
1

 0.00
1
species


 0.00
6
2
75787
1
0
family

no rank
0
1
90628
1
2

 0.00

151985
1
1

 0.00
species

1

 0.00
3
146937
0
1
genus

2

 0.00
1
146939
1
0
species

strain
640081
1
1

 0.00

2808923
3

 0.00
47
family
1
0

1
0
genus
2

 0.00
47
2808942

species
47
1

 0.00
1751046

10

 0.00
8
2008794
1
1
family

12960

 0.00
3
4
genus
1
0

no rank
1
1
2629479
4

 0.00
2


 0.00
1
3
2027405
species

4

 0.00
5
33057
1
2
genus

no rank
1
0
2609274
3

 0.00
3

1

 0.00
2
85643
species

2005884

 0.00
1
1
species

no rank
0
1
33809
7
4

 0.00

species
2
1

 0.00
1904640

species
1

 0.00
4
543913

species
1

 0.00
1
1891241

39

 0.00
28
32003
1
1
order

3

 0.00
1
2803844
1
0
family

0
1
genus
1
2

 0.00
2803845

species
2732067

 0.00
1
1

2008793
3

 0.00
7
family
0
1

0
1
genus
2
3

 0.00
1054211

748811
2

 0.00
2
species
0
1

strain
2
1

 0.00
1223802

1
0
genus

 0.00
3
1
378210

no rank
1
0
2639971

 0.00
2
1

species
1

 0.00
1
1842540

6
7

 0.00
90627
1
1
family

genus
2
1
453161
4
3

 0.00

species
453162
1

 0.00
1

species
2182327

 0.00
1
1

3

 0.00
1
314343
1
0
genus

1
2

 0.00
2642294
0
1
no rank

species
1
1

 0.00
2600596

32011
10

 0.00
11
family
1
0

119067

 0.00
2
1
no rank
1
0

2975065
1

 0.00
1
species


 0.00
2
1
1679002
1
0
genus

species
1

 0.00
1
1581557

genus
0
1
2872156
2
2

 0.00

2778876
1

 0.00
2
species

genus
0
1
359407
7
3

 0.00


 0.00
2
7
1055487
1
0
species

strain
666681
7
1

 0.00

206379

 0.00
5
4
family
1
0

genus
0
1
914
4
4

 0.00

2609265
3

 0.00
2
no rank
0
1

3
1

 0.00
42353
species

species
44577
1
1

 0.00

2772226

 0.00
6
2
family
1
0

1778653
3

 0.00
1
genus
1
0

no rank
0
1
2637784
1
2

 0.00

species
1985873
1

 0.00
1

2

 0.00
1
2772198
1
0
genus

2715678
1

 0.00
1
species

119066
2

 0.00
4
no rank
0
1

1
0
genus

 0.00
3
2
327159

1

 0.00
1
2954383
species

species
1

 0.00
1
2954388

5

 0.00
7
2008785
1
0
class

7

 0.00
4
119069
0
1
order

206349
3

 0.00
7
family
1
0


 0.00
2
7
70774
1
0
genus

species
7
1

 0.00
297

0
1
phylum
23

 0.00
30
203691

0
1
class
23
29

 0.00
203692

2
4

 0.00
1643688
0
1
order

1
0
family

 0.00
3
2
170

171
2

 0.00
2
genus
1
1

1

 0.00
1
28182
species

order
1
0
1643687

 0.00
3
1

1
0
no rank

 0.00
2
1
2614240

2979017

 0.00
1
1
species

1643686
8

 0.00
5
order
0
1

family
1
0
143786
4

 0.00
8

29521

 0.00
3
8
genus
1
4

1

 0.00
1
159
species

52584

 0.00
1
3
species

12

 0.00
16
136
0
1
order

family
0
1
2845253
3

 0.00
7

1
0
genus
6

 0.00
3
157

0
1
species
1
2

 0.00
53419

subspecies
69713
1

 0.00
1

1
0
species
2

 0.00
1
88058

1

 0.00
1
545694
strain

species
1

 0.00
1
221027

5
5

 0.00
2791015
0
1
family

5
4

 0.00
399320
0
1
genus

species
3

 0.00
1
1129264

2

 0.00
2
1131703
0
1
species

strain
158189
1

 0.00
2

3
1
family
4
3

 0.00
1643685

0
1
genus
1
2

 0.00
138

species
1

 0.00
1
140

131
85

 0.01
1783257
4
1
clade

phylum
1
4
203682
44

 0.00
78

666505
3
4

 0.00
class
0
1

order
0
1
666506
3
3

 0.00

0
1
no rank
3
2

 0.00
1500946

species
2052180
1

 0.00
3

2
7

 0.00
2517206
0
1
class


 0.00
6
2
1127829
1
0
order

1
0
family
5

 0.00
2
1127830

1
0
genus
4

 0.00
2
380240

1
0
no rank
2

 0.00
1
2685353

2172549

 0.00
1
1
species

species
2608983

 0.00
1
1

473814
1
2

 0.00
no rank
0
1

species
2528008
1
1

 0.00

30

 0.00
68
203683
1
19
class

order
1
1
2691354

 0.00
13
10

2691359
4

 0.00
3
family
0
1

2795783
2

 0.00
4
genus
1
0

4

 0.00
1
2528009
species

family
0
1
2691357
5

 0.00
9

2
2

 0.00
2807414
0
1
genus

1

 0.00
2
2528021
species

2795973

 0.00
2
1
genus
1
0

species
1

 0.00
1
2527979

1

 0.00
2
1579506
0
1
genus

species
980254
1

 0.00
1

2795605
2

 0.00
1
genus
1
0

species

 0.00
1
1
2527968

0
1
order
33
8

 0.00
2691355

family
1
15
1914233

 0.00
7
33


 0.00
2
1
2774146
1
0
genus

2774151

 0.00
1
1
species


 0.00
2
12
2052163
1
0
no rank

species
12

 0.00
1
2052164

genus
1
0
2807415
2

 0.00
5

species
5
1

 0.00
2528023

3
6

 0.00
112
0
1
order

1
1
family
3

 0.00
5
126

genus
1
0
2795774

 0.00
2
1

species
1

 0.00
1
2528007

1649453
1

 0.00
2
genus
0
1


 0.00
1
1
2527964
species


 0.00
2
3
2691356
1
0
order

family

 0.00
1
3
1763524

1
0
phylum

 0.00
9
3
204428

0
1
class
3

 0.00
8
204429

1963360
7

 0.00
3
order
1
1

689704
1

 0.00
3
family
0
1

genus
1
0
292833

 0.00
2
1

species
2720720
1

 0.00
1

family
0
1
92713
1
3

 0.00


 0.00
2
1
282132
1
0
genus


 0.00
1
1
389348
species

1
0
phylum
4

 0.00
1
256845

class
1
0
1313211

 0.00
3
1

1368584
1

 0.00
2
no rank
0
1

species
2053569
1
1

 0.00


 0.00
27
45
74201
1
4
phylum

414999
35
14

 0.00
class
0
1

order
1
0
415000

 0.00
13
35

family
1
15
134623
12

 0.00
35

1

 0.00
2
278955
0
1
no rank

794903
1

 0.00
1
species

1
0
genus

 0.00
2
9
1961799

species
9

 0.00
1
1838286

2

 0.00
3
2576890
1
0
genus

3

 0.00
1
2576891
species

1
1
genus
7
5

 0.00
178440

2

 0.00
5
2649488
1
0
no rank

1882749
1

 0.00
5
species

0
1
species
1

 0.00
2
107709

strain
1

 0.00
1
452637


 0.00
2
1
417295
1
0
no rank

species
1637999
1
1

 0.00

no rank
1
0
74202
2

 0.00
1

species
156588
1

 0.00
1

203494
4
8

 0.00
class
0
1

order
1
0
48461
7

 0.00
4

3

 0.00
3
1647988
1
0
family

3
2

 0.00
239934
0
1
genus

species
239935
3

 0.00
1


 0.00
3
1
203557
1
0
family

518753
1

 0.00
2
genus
0
1

species
1

 0.00
1
2728835

no rank
1
0
49928

 0.00
2
4

1869227

 0.00
1
4
species

4908

 9.87
256070
2759
1
17049
superkingdom

554915
15

 0.00
17
clade
1
0

phylum
0
1
2605435
17
14

 0.00

142796

 0.00
7
13
class
1
0

33083
6

 0.00
13
clade
1
0

order
0
1
2058181
13

 0.00
5

2058183
13

 0.00
4
family
0
1

13
3

 0.00
2058189
0
1
genus

species
0
1
2086695
13

 0.00
2

13

 0.00
1
670386
strain

clade
0
1
555406
4

 0.00
6

order
1
0
2682482

 0.00
5
4

family
1
0
33084

 0.00
4
4


 0.00
3
4
5758
1
3
genus

2

 0.00
1
46681
1
0
species

strain
1

 0.00
1
370354

1

 0.00
8
2608109
0
1
clade

1

 0.00
7
2830
0
1
phylum

clade
1
0
2608131
6

 0.00
1

order
0
1
73020
1
5

 0.00

418966
1

 0.00
4
family
0
1

genus
0
1
2902
1

 0.00
3

2903

 0.00
2
1
species
1
0

strain
1

 0.00
1
280463

1
2846
clade

 9.12
3981
236488
33154

1
1
class
8

 0.00
5
28009

4
7

 0.00
1924738
0
1
order

4

 0.00
6
81529
0
1
family

86017
2

 0.00
3
genus
1
0

species
946362

 0.00
1
3

genus
1
0
81525

 0.00
3
1

1
2

 0.00
81824
0
1
species

strain
431895
1

 0.00
1

kingdom
1
5
4751

 0.03
578
854

0
1
no rank
17

 0.00
15
112252

phylum
0
1
1913637
17

 0.00
14

15

 0.00
7
214504
0
1
subphylum

0
1
class
15
6

 0.00
214506

1
0
order
5

 0.00
15
36750

1
0
family

 0.00
4
15
36751

genus
1
1
1129544
3

 0.00
15

species
1
13
588596
2

 0.00
14

747089
1
1

 0.00
strain

2

 0.00
6
451507
0
1
subphylum

class
0
1
2212703
2

 0.00
5


 0.00
4
2
4827
1
0
order

1
0
family
3

 0.00
2
499202


 0.00
2
2
688353
1
0
genus

2

 0.00
1
688394
species

subkingdom
1
17
451864

 0.03
560
831

419
407

 0.02
4890
0
1
phylum

subphylum
0
1
451866
25

 0.00
7

1
0
class
6

 0.00
25
147554

0
1
order
25

 0.00
5
34346

1
0
family
4

 0.00
25
4894

3

 0.00
25
4895
1
0
genus

0
1
species
25
2

 0.00
866546

strain
1

 0.00
25
653667

716545
394
399

 0.02
clade
5
1

340
322

 0.01
147538
2
1
subphylum

class
1
0
147549

 0.00
7
4


 0.00
6
4
5185
1
0
order

1
0
family
5

 0.00
4
5192


 0.00
4
4
5193
1
0
genus

4

 0.00
3
1051054
0
1
section

species
1174677

 0.00
1
3

species
1174673
1
1

 0.00

334

 0.01
314
716546
16
1
clade

clade
1
4
715989

 0.01
139
133

15
32

 0.00
147548
0
1
class

order
1
0
5120
5

 0.00
1

0
1
family
1
4

 0.00
34371

34372
1
3

 0.00
genus
0
1

species
1
0
34373

 0.00
2
1

forma specialis
62690
1
1

 0.00

order
1
0
5178
22

 0.00
13

1
5

 0.00
2793945
0
1
family

2081418
4

 0.00
1
genus
1
0

species
0
1
698440
1

 0.00
3

0
1
forma specialis
1

 0.00
2
698441

1072389
1
1

 0.00
strain

1
0
family

 0.00
4
1
2907085

genus
0
1
5100
1
3

 0.00

species
1
0
5101

 0.00
2
1

strain

 0.00
1
1
857342

28983
10

 0.00
9
family
0
1


 0.00
2
6
38447
1
0
genus

61207

 0.00
1
6
species

genus
0
1
33196
3

 0.00
3

species
2
1
40559
3

 0.00
2

strain
1

 0.00
1
332648

5179

 0.00
3
1
genus
1
0

1
0
species

 0.00
2
1
5180

isolate
665079
1
1

 0.00

1
0
family
3

 0.00
1
2656784


 0.00
2
1
2656786
1
0
genus

2656787

 0.00
1
1
species

4

 0.00
1
221903
1
0
no rank

family
0
1
34379
1
3

 0.00

genus
1
0
78156
2

 0.00
1

1
1

 0.00
655981
species

147550
106

 0.00
114
class
1
7

subclass
1
0
222544

 0.00
25
10

order
1
0
5139
10

 0.00
6

0
1
family
1
3

 0.00
42302

42303
1

 0.00
2
genus
0
1

species
1899523
1

 0.00
1

family
0
1
2609812
3
2

 0.00

5144
1

 0.00
3
genus

family
1
0
35718

 0.00
4
2

1
0
genus

 0.00
3
2
2609811

2

 0.00
2
2587410
0
1
species

strain
578455
2
1

 0.00

1
0
order

 0.00
4
1
639021

1

 0.00
3
2528436
0
1
family

48558
1

 0.00
2
genus
0
1

species
318829
1

 0.00
1

1
0
order
10

 0.00
3
5114

767018
1

 0.00
2
family
0
1

genus

 0.00
1
1
36922


 0.00
7
2
399129
1
0
family

1276216
1

 0.00
2
genus
0
1

species
1
1

 0.00
1276217

1
0
no rank
4

 0.00
1
218105

genus
0
1
305399
1

 0.00
3

no rank
2625752
0
1

 0.00

species

 0.00
1
1
2029752


 0.00
14
17
222545
1
0
subclass

order
1
0
37989

 0.00
10
15

0
1
family
2

 0.00
3
106263

0
1
genus
2
2

 0.00
1811811

species
335852
2

 0.00
1

2033035
6

 0.00
13
family
1
0

11

 0.00
2
42308
0
1
genus

species
63214

 0.00
1
11

3

 0.00
2
42360
1
0
genus

species
103429
1

 0.00
1

species
326647
1
1

 0.00

1
0
no rank
3

 0.00
2
1830229

2

 0.00
2
2983819
1
0
genus

1

 0.00
2
1658444
species

1
4
subclass
62

 0.00
78
222543

0
1
order
6

 0.00
14
1028384

1
0
family

 0.00
4
1
1033978

3

 0.00
1
1401161
1
0
genus

2

 0.00
1
1302862
1
0
species

strain
1314773

 0.00
1
1

681950
5

 0.00
9
family
0
1

8

 0.00
5
5455
1
0
genus

3

 0.00
1
2707350
1
0
no rank

1
0
species
2

 0.00
1
80884

1

 0.00
1
759273
strain

no rank
0
1
2707335
1

 0.00
2

145971
1

 0.00
1
species

1
0
no rank

 0.00
2
3
2872341

3

 0.00
1
1543685
species

6
1
order
68

 0.00
47
5125

110618

 0.00
20
23
family
1
0

genus
0
1
140106
2
2

 0.00

78403
2
1

 0.00
species


 0.00
3
2
57138
1
0
genus

no rank
0
1
2779503
2

 0.00
2


 0.00
1
2
182845
species

5506
19

 0.00
14
genus
0
1

species group
1
0
569360
4

 0.00
4

1
1

 0.00
56646
species


 0.00
1
1
5518
species

101028
1

 0.00
2
species

3

 0.00
5
171627
1
0
species group

species
1

 0.00
1
192010

4

 0.00
1
5127
species

450425
5
3

 0.00
species group
1
1

2675880
1

 0.00
2
species

231269
2

 0.00
1
species

1
0
species group
3

 0.00
5
232080

species
1

 0.00
4
2747967

species
169388
1

 0.00
1

5129

 0.00
6
10
family
1
0

5543

 0.00
5
10
genus
1
0

species
51453

 0.00
1
7

1
0
species

 0.00
2
1
29875

strain
1
1

 0.00
1331945

species
63577
2

 0.00
1

12

 0.00
22
34397
1
0
family

124426
3
2

 0.00
genus
0
1

species
1159556
3

 0.00
1

1
0
genus

 0.00
2
2
5529

species
2
1

 0.00
500148

1
0
genus
7

 0.00
17
5112

species
42805

 0.00
1
8

species

 0.00
1
2
47801

1
1
species
3
3

 0.00
5113

2570311
1

 0.00
1
subspecies

1616224
1

 0.00
1
subspecies

species
55200
1

 0.00
4

2
1
family
6

 0.00
5
474943

genus
1
1
45234

 0.00
2
3


 0.00
1
2
73501
species

1
0
genus
2

 0.00
1
5581

species
1

 0.00
1
176275

1
0
family

 0.00
3
1
474942

genus
1
0
98402

 0.00
2
1

1

 0.00
1
98403
species

147551
2

 0.00
4
no rank
0
1

265081
2

 0.00
3
family
0
1


 0.00
2
2
265082
1
0
genus

1093900

 0.00
1
2
species

147545

 0.00
79
96
class
1
2

0
1
subclass
84
59

 0.00
451871

0
1
order
78
47

 0.00
5042

1
1
family
76
40

 0.00
1131492

genus
1
1
5073
23

 0.00
6

species

 0.00
1
1
60172

1
0
no rank

 0.00
3
20
254878

species
0
1
1108849
20

 0.00
2

1

 0.00
20
500485
strain

1

 0.00
1
5074
species

genus
1
0
70110

 0.00
3
3

3

 0.00
2
41063
0
1
species

strain
1073090
3

 0.00
1


 0.00
30
49
5052
1
7
genus

2

 0.00
2
51019
1
0
species

1

 0.00
2
1448321
strain

1

 0.00
2
1069201
species

1
0
species
2

 0.00
2
979771

1450539
2

 0.00
1
strain


 0.00
1
2
41047
species

2
1

 0.00
132259
species


 0.00
1
2
1341132
species

1
0
species
2

 0.00
1
1196635

strain
1

 0.00
1
1448310

1

 0.00
1
138277
species

1
0
species

 0.00
2
1
1191702

strain
1
1

 0.00
1448319

319627
2

 0.00
1
species
1
0

1450535
1
1

 0.00
strain


 0.00
6
17
2720871
1
0
subgenus

5053
2

 0.00
1
species
1
0


 0.00
1
1
690307
strain

1
0
species
2

 0.00
2
33178

341663
2

 0.00
1
strain

species

 0.00
1
14
5061

1
0
species

 0.00
2
2
446911

1448315
2

 0.00
1
strain

species
61420
2

 0.00
1

species
1
1

 0.00
109264

species
1220207

 0.00
1
2

species
0
1
34381
1

 0.00
2


 0.00
1
1
1448312
strain

species
1
1

 0.00
182096

2

 0.00
6
28568
0
1
family

genus
1
0
5094

 0.00
5
2

2

 0.00
1
2752543
1
0
section

1

 0.00
1
1131652
species

section
0
1
2752537
1

 0.00
2

37727
1

 0.00
1
species


 0.00
11
6
33183
1
0
order

1
0
family

 0.00
8
5
299071

genus
0
1
229219
2
3

 0.00

species
1
0
5039

 0.00
2
2

strain
559297

 0.00
1
2

1
0
genus

 0.00
4
3
5036

1
0
species
3

 0.00
3
5037

447093
2

 0.00
1
strain

1

 0.00
1
544711
strain

family
1
0
34384
2

 0.00
1

5550

 0.00
1
1
genus

0
1
subclass
10

 0.00
19
451870

0
1
order
1
5

 0.00
146291

0
1
family
1
4

 0.00
146292

364710
3

 0.00
1
genus
1
0

1
0
species

 0.00
2
1
364733

1
1

 0.00
1263415
strain

1
0
order

 0.00
13
9
34395

43219
9
12

 0.00
family
1
1

1
0
genus

 0.00
3
1
5587

86056

 0.00
2
1
species
1
0

strain
1442369
1

 0.00
1

5583
6
7

 0.00
genus
0
1

species
1
0
5970
2

 0.00
2

strain
2

 0.00
1
858893


 0.00
2
1
1033840
1
0
species

1
1

 0.00
1182545
strain

species
1

 0.00
1
212818

species
1

 0.00
2
91925

genus

 0.00
1
1
82105

715962
86

 0.00
82
clade
0
1


 0.00
81
86
147541
1
1
class

1
1
subclass
16
22

 0.00
451867

5014

 0.00
4
5
order
1
0

family
1
0
1570301
3

 0.00
5

5579
5

 0.00
2
genus
0
1

species
46634
5

 0.00
1

order
1
0
2726946
3

 0.00
2

1
0
family
2

 0.00
2
452563

genus
1

 0.00
2
5498

order
0
1
2726947
8

 0.00
14

668547
2
6

 0.00
family
0
1

483074

 0.00
3
1
genus
1
0

0
1
species
1

 0.00
2
1709381

1

 0.00
1
717646
strain

2

 0.00
1
91942
1
0
genus

species
91943
1

 0.00
1

93133
7

 0.00
6
family
1
0


 0.00
2
1
2897311
1
0
genus

5499
1

 0.00
1
species

genus
0
1
39702
1

 0.00
2

215465
1

 0.00
1
species

genus
0
1
1047167
4

 0.00
2

species

 0.00
1
4
1047171

159987

 0.00
14
10
no rank
1
0

2810619

 0.00
3
3
genus
1
0

3

 0.00
2
61459
0
1
species

strain
1168221
3

 0.00
1

7

 0.00
10
451869
0
1
order

2
4

 0.00
1450293
0
1
family


 0.00
3
2
462253
1
0
genus

species
0
1
462254
2

 0.00
2


 0.00
1
2
1176127
strain

5

 0.00
5
45131
1
0
family

66739
1

 0.00
2
genus
0
1

species
45133

 0.00
1
1

407951
2

 0.00
4
genus
1
0

310453
4

 0.00
1
species

subclass
1
1
451868
59
44

 0.00

order
1
1
92860

 0.00
43
58

1

 0.00
4
147498
0
1
no rank

1450170

 0.00
3
1
genus
1
0

species
1
0
1450171

 0.00
2
1

strain
1450172
1
1

 0.00

1
0
suborder
4

 0.00
1
1255046

family
1
0
221678

 0.00
3
1

125369

 0.00
2
1
genus
1
0

species
1460663
1

 0.00
1

1
0
family
4

 0.00
1
548648

genus
1
0
548651

 0.00
3
1

1
0
species
2

 0.00
1
548649

1392245

 0.00
1
1
strain

7
1
suborder
54
30

 0.00
715340

1
1
family

 0.00
21
42
28556

1
0
genus
2

 0.00
1
95729

183478
1
1

 0.00
species

genus
1
0
5027

 0.00
4
2

53485
2

 0.00
3
species
0
1

97480

 0.00
1
1
forma

forma

 0.00
1
1
97479

37

 0.00
12
5598
2
1
genus

section
1
0
2499237
3

 0.00
2


 0.00
2
2
187734
1
0
species group

2

 0.00
1
5599
species

2

 0.00
2
2499270
0
1
section

1187941

 0.00
1
2
species

section
22
1
2499258
31

 0.00
6

species
297637
1

 0.00
2

430562
1
1

 0.00
species

283354
1

 0.00
4
species

566460

 0.00
1
1
species

1

 0.00
1
1187951
species

0
1
genus
1

 0.00
2
91493

species
1
1

 0.00
93612

34374
4
5

 0.00
family
0
1

genus
118259
4
3

3
1
no rank
4

 0.00
3
220671

0
1
species
1

 0.00
2
220672

no rank
1

 0.00
1
225338

genus
0

 0.00
1
5021

0
1
family
1

 0.00
3
683158

1

 0.00
2
749461
0
1
genus

species
749465
1

 0.00
1

147547

 0.00
13
3
class
1
0

clade
0
1
1520881
3
12

 0.00

3
11

 0.00
388435
0
1
subclass

5

 0.00
1
388450
1
0
order

1
0
suborder

 0.00
4
1
157824

48860

 0.00
3
1
family
1
0

48861
1

 0.00
2
genus
0
1

52884
1

 0.00
1
species

order
1
0
5197
5

 0.00
2

1
0
suborder
4

 0.00
2
157822


 0.00
3
2
56478
1
0
family

93111
2

 0.00
2
genus
0
1

species
2732470
2

 0.00
1

subphylum
0
1
147537
49
76

 0.00

class
0
1
4891
49
75

 0.00

1
1
order
49

 0.00
74
4892

4893
24

 0.00
14
family
1
0

genus
1
0
4930
3

 0.00
2

4932

 0.00
1
1
species

species
27291

 0.00
1
1

0
1
genus
1

 0.00
2
4948

4950
1

 0.00
1
species

374468
2

 0.00
2
genus
1
0

5478
2

 0.00
1
species

3

 0.00
2
4910
0
1
genus


 0.00
1
3
4911
species

0
1
genus
1
3

 0.00
113604

1

 0.00
2
113608
0
1
species

strain
1

 0.00
1
1071381

1
0
genus

 0.00
5
2
33170

species
1
0
45285

 0.00
2
1

strain
1

 0.00
1
931890

species
0
1
33169
1

 0.00
2

1034331

 0.00
1
1
strain

genus
0
1
374469
2

 0.00
3

2

 0.00
2
36033
1
0
species

436907
2
1

 0.00
strain

genus
0
1
71245
1
3

 0.00

species
1
0
432096

 0.00
2
1


 0.00
1
1
1071382
strain

34353

 0.00
3
4
family
1
0

2

 0.00
4
4951
1
0
genus

species
4952
4
1

 0.00

2926619
6

 0.00
2
clade
1
0

family
1
0
34366

 0.00
5
2

genus
0
1
4943
2

 0.00
4

no rank
0
1
2636529
1
2

 0.00

1

 0.00
1
2826930
species

4944

 0.00
1
1
species

410830
3

 0.00
1
family
1
0

45787

 0.00
2
1
genus
1
0


 0.00
1
1
45607
species

1156497
2

 0.00
4
family
0
1

0
1
genus
2
3

 0.00
4919

0
1
species
2
2

 0.00
4926

763406
2
1

 0.00
strain

20
25

 0.00
2916678
0
1
clade

family
1
0
766764

 0.00
19
16

genus
1
0
766502

 0.00
3
1

4920
1

 0.00
2
species
0
1

strain
559304
1

 0.00
1

clade
1
0
1535325
6

 0.00
11

5475

 0.00
5
11
genus
1
0

5476

 0.00
1
5
species

5
1

 0.00
5480
species

species
1
0
42374

 0.00
2
1

573826
1

 0.00
1
strain

1
3

 0.00
1539666
0
1
genus

46583
2

 0.00
1
species
1
0

984487
1

 0.00
1
strain

4958
2

 0.00
3
genus
0
1


 0.00
2
2
4959
1
0
species

2
1

 0.00
284592
strain

3

 0.00
1
766728
1
0
genus

species
1
0
4929

 0.00
2
1

strain
294746
1

 0.00
1

4

 0.00
5
27319
0
1
family

0
1
no rank
4
4

 0.00
2937349

2964429
3

 0.00
4
genus
1
2

45354
1

 0.00
1
species

species
1

 0.00
1
418784

1
0
family
5

 0.00
2
115784

genus
1
0
599737

 0.00
2
1

species

 0.00
1
1
1041607

460517

 0.00
2
1
genus
1
0

4922
1
1

 0.00
species

no rank
0
1
241407
3
3

 0.00

genus
0
1
2952368
3

 0.00
2

3
1

 0.00
312227
species

phylum
1
1
5204
152

 0.02
395

452284
269
24

 0.01
subphylum
0
1

class
0
1
5257
3

 0.00
9

5267

 0.00
8
3
order
1
0


 0.00
7
3
5268
1
0
family

0
1
genus
1
2

 0.00
63265

species
1
1

 0.00
72558

1
2

 0.00
63261
0
1
genus

1

 0.00
1
84753
species


 0.00
2
1
5269
1
0
genus

species
307758

 0.00
1
1

class
0
1
452283
1

 0.00
5

order
0
1
5404
1

 0.00
4

family
1
0
190068
3

 0.00
1

2

 0.00
1
215251
1
0
genus

species

 0.00
1
1
1280837

class
0
1
1538075
265

 0.01
9

162474
265
8

 0.01
order
0
1

family
0
1
742845
265
7

 0.01

265
6

 0.01
55193
0
1
genus

55194
1

 0.00
1
species

1

 0.00
28
76777
species

species
54

 0.00
1
76773

182

 0.01
2
76775
157
1
species

strain
25
1

 0.00
425264

5302

 0.00
99
114
subphylum
1
1

class
0
1
155616
16
20

 0.00

90886

 0.00
4
2
order
1
0

5408

 0.00
3
2
family
1
0

genus
1
0
5209
2

 0.00
2


 0.00
1
2
5210
species

4

 0.00
1
1851469
1
0
order

3

 0.00
1
1759442
1
0
family

105983
1
2

 0.00
genus
0
1

species
1

 0.00
1
252803

order
1
0
90883

 0.00
4
1

family
0
1
1851551
1

 0.00
3

0
1
genus
1
2

 0.00
107449

species

 0.00
1
1
264483

0
1
order
12
7

 0.00
5234

12
6

 0.00
1884633
0
1
family

genus
1
0
490731

 0.00
2
2

1

 0.00
2
1734106
species

10

 0.00
3
5206
0
1
genus

species group
1
0
1897064
2

 0.00
10

species

 0.00
1
10
5207

class
1
5
155619
78

 0.00
97

no rank
1
5
355688

 0.00
49
70

order
1
0
452338
5

 0.00
1

family
1
0
908827

 0.00
4
1

0
1
genus
1

 0.00
3
133746

202698

 0.00
2
1
species
1
0

strain
741275
1

 0.00
1

36064
1
3

 0.00
order
0
1

0
1
family
1

 0.00
2
5250

1322061
1

 0.00
1
genus

1
0
order
7

 0.00
2
139380

family
1
0
40424
3

 0.00
1

genus
1
0
40468

 0.00
2
1

167356
1

 0.00
1
species

3

 0.00
1
574935
1
0
family

2

 0.00
1
1213737
1
0
genus

27342
1

 0.00
1
species

452342
31

 0.00
13
order
0
1

3

 0.00
3
103393
1
0
family

genus
1
0
40463
2

 0.00
3

3
1

 0.00
154539
species

0
1
family
2

 0.00
5
40420

1
0
genus

 0.00
4
2
13562

1
0
no rank

 0.00
3
2
256003

984962
2
2

 0.00
species
1
1

747525

 0.00
1
1
strain

1
0
family

 0.00
4
26
103376

1
0
genus
3

 0.00
26
5644

species
0
1
40492
26
2

 0.00

721885

 0.00
1
26
strain

1
2
order

 0.00
20
30
5303

1
0
family

 0.00
4
2
2028212

genus
1
0
5629

 0.00
3
2

2

 0.00
2
5630
1
1
species

1314785
1

 0.00
1
strain

1769247
8

 0.00
5
family
0
1

81045
1

 0.00
2
genus
0
1

species
81046
1

 0.00
1

1
0
genus

 0.00
2
7
2983002

species
139415
7
1

 0.00

1
1
family

 0.00
5
16
5317

5324
4

 0.00
15
genus
1
0


 0.00
1
2
5327
species

5325
13
2

 0.00
species
1
1

strain
717944
1

 0.00
12


 0.00
2
1
2028214
1
0
family

1
1

 0.00
234811
genus

1
3

 0.00
81064
0
1
family

0
1
genus
1
2

 0.00
5307

5308
1
1

 0.00
species

22

 0.00
28
452333
0
1
subclass

68889

 0.00
6
2
order
1
0


 0.00
5
2
227332
1
0
suborder

family
1
0
227336

 0.00
4
2

2
3

 0.00
5379
0
1
genus

species
1

 0.00
1
48587


 0.00
1
1
116603
species

order
1
2
5338
21

 0.00
20


 0.00
4
4
2983527
1
0
suborder

104366
4

 0.00
3
family
0
1

5320
2

 0.00
4
genus
1
0

species

 0.00
1
4
5322

2982316
5

 0.00
4
suborder
0
1

1
0
family

 0.00
3
5
862241

genus
0
1
38944
5
2

 0.00

species
38945
5
1

 0.00


 0.00
4
6
2982303
1
0
suborder

1
0
family
3

 0.00
6
5351

40144
6
2

 0.00
genus
0
1

1

 0.00
6
40145
species

2982305
3

 0.00
8
suborder
0
1

0
1
family
2

 0.00
3
5339

genus
1
0
5340
2

 0.00
2

5341
1

 0.00
2
species

family
1
0
184208

 0.00
4
1

184431
1
3

 0.00
genus
0
1

species
0
1
5346
1
2

 0.00

240176
1

 0.00
1
strain

subphylum
1
0
29000
22

 0.00
10

1

 0.00
5
162481
0
1
class

order
0
1
231213
1
4

 0.00

1799696
3

 0.00
1
family
1
0

genus
1
0
5533
2

 0.00
1

1

 0.00
1
5286
species

class
0
1
162484
9
16

 0.00

1
0
order

 0.00
15
9
5258

5262
4
7

 0.00
family
0
1

6

 0.00
4
5296
1
0
genus

5297
3

 0.00
1
species
1
0

1
0
forma specialis

 0.00
2
1
56615

1

 0.00
1
418459
strain

species
1
0
27350
2

 0.00
3

forma specialis
168172
3

 0.00
1

2

 0.00
4
5259
0
1
family

2

 0.00
3
5260
0
1
genus

2

 0.00
2
203908
0
1
species

747676
1

 0.00
2
strain

3
3

 0.00
2792465
0
1
family

3

 0.00
2
203903
0
1
genus

species
203904

 0.00
1
3

1
0
subphylum

 0.00
6
1
2204096

5

 0.00
1
431957
1
0
class

431958
4

 0.00
1
order
1
0


 0.00
3
1
431959
1
0
family

genus
0
1
148959
1

 0.00
2

species
148960
1
1

 0.00

0
1
no rank
1

 0.00
2
57731

species
175245
1
1

 0.00

kingdom
217
1
33208
232782

 8.97
3390

232549

 8.96
3377
6072
539
1
clade

72
52

 0.00
6073
3
1
phylum

class
1
0
6101

 0.00
34
58

6102
56

 0.00
29
subclass
0
1

order
0
1
6125
14
14

 0.00

9

 0.00
7
123757
1
0
suborder

family
3
1
46729
6

 0.00
5

genus
1
0
50428
2

 0.00
2

2

 0.00
1
50429
species

1
2

 0.00
46730
0
1
genus

1

 0.00
1
46731
species

1
0
family
3

 0.00
1
6126

genus
0
1
6127
1
2

 0.00

1

 0.00
1
45264
species

123760
7
4

 0.00
suborder
0
1

family
0
1
46736
7
3

 0.00

1
0
genus
2

 0.00
7
1920453

species
48498
7

 0.00
1


 0.00
14
42
6103
1
0
order

0
1
suborder
21
4

 0.00
86626

3

 0.00
21
478428
1
0
family

21
2

 0.00
478394
0
1
genus

species
21

 0.00
1
1789172

42823
4

 0.00
3
family
0
1

1720308
4

 0.00
2
genus
0
1


 0.00
1
4
2652724
species

1
0
family
3

 0.00
2
42822

genus
1
0
6104

 0.00
2
2

6105

 0.00
1
2
species

family
0
1
45349
15

 0.00
3

0
1
genus
15
2

 0.00
45350

species
15
1

 0.00
45351

2

 0.00
4
6132
0
1
subclass

order
3028843
3
2

51108

 0.00
3
2
family
1
0

2
2

 0.00
51109
0
1
genus

species
1

 0.00
2
151771

1927913
6

 0.00
6
class
1
0

37528
5

 0.00
6
order
1
0


 0.00
4
6
1927915
1
0
suborder

family
1
0
1927917
3

 0.00
6

genus
1
0
37533

 0.00
2
6

313498
1

 0.00
6
species

5

 0.00
11
6074
0
1
class

0
1
subclass
5

 0.00
10
37516

406427
9

 0.00
5
order
1
0

0
1
suborder
4

 0.00
4
1612408

1
0
family

 0.00
3
4
6080


 0.00
2
4
6083
1
0
genus

species

 0.00
1
4
6087

0
1
suborder
1

 0.00
4
406428


 0.00
3
1
6094
1
0
family

genus
0
1
6095
1
2

 0.00

species

 0.00
1
1
6096

33213

 8.94
3324
231938
clade
1
27227

1287

 6.39
165797
33511
1
119
clade

165635
1246

 6.38
7711
131
1
phylum

subphylum
0
1
89593
165461
1219

 6.38

clade
1
14
7742

 6.38
1218
165461

1476529
12

 0.00
8
clade
1
0

6

 0.00
1
117565
1
0
class

1
0
order

 0.00
5
1
7761

1
0
family

 0.00
4
1
7762

1

 0.00
3
30309
0
1
subfamily

2

 0.00
1
7763
1
0
genus

1

 0.00
1
7764
species

1
0
class

 0.00
5
7
117569

order
0
1
7745
7
4

 0.00

7746

 0.00
3
7
family
1
0

2

 0.00
7
7756
1
0
genus

species
7757

 0.00
1
7

1
71
clade

 6.38
1205
165439
7776

1
0
clade

 6.37
1173
165323
117570

117571
1172

 6.37
165323
clade
1
4293

1
7
superclass
490

 0.18
4551
7898

5

 0.00
56
1338366
1
0
class

56

 0.00
4
8288
0
1
order

3

 0.00
56
8289
1
1
family

1
0
genus
2

 0.00
55
27686

species
27687
55

 0.00
1

186623
4488

 0.17
484
class
98
1

41665

 0.17
470
4318
subclass
1
2

32443
4312

 0.17
464
infraclass
8
1

1
0
clade

 0.00
11
5
1489340

186624
5
10

 0.00
clade
0
1

32521
5
9

 0.00
clade
0
1

0
1
order
4

 0.00
4
7925

1
0
family
3

 0.00
4
7930

4

 0.00
2
7931
0
1
genus

species
4
1

 0.00
118141

order
1
0
7933
4

 0.00
1

1
3

 0.00
7934
0
1
family

genus
1
0
7935

 0.00
2
1

1

 0.00
1
7936
species

clade
76
1
1489341
4299

 0.17
452

1
420
no rank

 0.16
440
4187
186625

186634
795

 0.03
93
cohort
9
1

subcohort
1
0
282425
14

 0.01
166

order
1
0
32446
13

 0.01
166

1489460
125

 0.00
8
suborder
0
1

55118

 0.00
7
125
family
1
4

115

 0.00
3
7948
0
1
subfamily

genus
0
1
7949
115
2

 0.00

species
115
1

 0.00
7950

0
1
subfamily
6
3

 0.00
55119

1
3
genus
2

 0.00
6
34772

34773
3

 0.00
1
species

suborder
0
1
1489459
41

 0.00
4

299319
3

 0.00
41
family
1
0

1
0
genus

 0.00
2
41
299320

species
299321

 0.00
1
41

1
9
subcohort

 0.02
78
620
32519

186633
37
6

 0.00
clade
0
1


 0.00
5
37
29140
1
0
order

4

 0.00
37
186632
1
0
suborder

family
0
1
29142
37

 0.00
3

1
0
genus

 0.00
2
37
29143

species
29144
37

 0.00
1

186626
574
71

 0.02
clade
11
1

superorder
1
0
186627

 0.02
38
470

37

 0.02
470
7952
1
5
order

0
1
suborder
11
4

 0.00
30725

family
1
0
278171
3

 0.00
11

genus
0
1
160394
11

 0.00
2

1

 0.00
11
135647
species


 0.02
32
454
30727
1
12
suborder

family
1
0
2743709

 0.01
6
291

2743711

 0.01
5
291
subfamily
1
0

291
4

 0.01
7954
32
1
genus

species
1

 0.00
101
7955

1142201
71
1

 0.00
species

species
242068
87
1

 0.00

family
1
0
2743714
5

 0.00
1

1

 0.00
4
2743715
0
1
subfamily

1

 0.00
3
328542
0
1
genus

no rank
0
1
2624887
1

 0.00
2

species
1

 0.00
1
1664672

4

 0.00
1
2743745
1
0
family

subfamily
1
0
2743747

 0.00
3
1

1
0
genus
2

 0.00
1
75351

1
1

 0.00
75352
species

12

 0.01
146
7953
1
7
family

subfamily
1
1
2743694
97

 0.00
8

genus
1
0
75365
3

 0.00
3

species
1

 0.00
1
75366

2

 0.00
1
307959
species

7961
2

 0.00
89
genus
1
0

species
7962

 0.00
1
89

0
1
genus
4

 0.00
2
7956

1

 0.00
4
7957
species


 0.00
3
42
2743693
1
0
subfamily

2

 0.00
42
40829
1
0
genus

42
1

 0.00
40830
species

4

 0.00
3
2743726
1
0
family

2743731
3

 0.00
3
subfamily
1
0

51137

 0.00
2
3
genus
1
0

90988

 0.00
1
3
species

0
1
superorder
93

 0.00
32
186628

1
0
order
15

 0.00
77
7995

1
1
suborder
77
14

 0.00
1489793

30989
61

 0.00
4
family
0
1

genus
1
10
94992
3

 0.00
61

1

 0.00
3
175797
species

933932
48

 0.00
1
species

family
0
1
31013
1

 0.00
3

0
1
genus
1
2

 0.00
641818

species
1234273
1

 0.00
1

family
0
1
7999
10

 0.00
3

genus
0
1
30992
10
2

 0.00

1

 0.00
10
310915
species

family
1
0
7996

 0.00
3
4

genus
1
0
7997

 0.00
2
4


 0.00
1
4
7998
species

order
1
0
8002
5

 0.00
3

0
1
suborder
3
4

 0.00
1489620

30771
3

 0.00
3
family
1
0

genus
0
1
8004
3

 0.00
2

species
8005
1

 0.00
3

order
1
0
7991
11

 0.00
13

suborder
0
1
1489739
13

 0.00
10


 0.00
5
4
42495
1
0
family

1
0
genus

 0.00
2
1
42513

species
42514

 0.00
1
1

42525
3
2

 0.00
genus
0
1

species

 0.00
1
3
42526

family
0
1
7992
9
4

 0.00

subfamily
1
0
42595

 0.00
3
9

genus
1
0
7993

 0.00
2
9

7994
9
1

 0.00
species

1489388
2972
346

 0.11
cohort
536
1

1
0
clade

 0.02
21
552
41705

8007
5

 0.00
4
order
0
1

family
1
0
8008

 0.00
3
5

genus
0
1
8009
5

 0.00
2

species
1

 0.00
5
8010

8006
16

 0.02
547
order
1
0

15

 0.02
547
8015
1
21
family

504568
9

 0.01
141
subfamily
1
1

0
1
genus
136
3

 0.01
8028

species
8032

 0.01
1
133

8030
3
1

 0.00
species

1
1
genus
3

 0.00
3
8016

1

 0.00
1
74940
species

species
8019
1
1

 0.00

2

 0.00
1
8033
1
0
genus

species
1

 0.00
1
8040

subfamily
1
0
504567
5

 0.01
385

27772
4

 0.01
385
genus
1
0


 0.01
2
383
2649731
1
0
no rank

1

 0.01
383
861768
species


 0.00
1
2
59861
species


 0.07
318
1882
123365
1
0
clade


 0.07
317
1882
123366
1
0
clade

clade
0
1
123367
1882

 0.07
316

315

 0.07
1882
123368
1
36
clade

1489838

 0.00
8
50
clade
1
0

1
0
clade
7

 0.00
50
1489841

1489843
50

 0.00
6
clade
0
1

order
0
1
8043
50

 0.00
5

1
0
suborder

 0.00
4
50
1489845

1
0
family

 0.00
3
50
8045

8048
50
2

 0.00
genus
0
1

1

 0.00
50
8049
species

1796

 0.07
306
123369
56
1
clade

54

 0.00
6
181483
0
1
clade

5

 0.00
54
1490028
1
0
order

1
0
family
4

 0.00
54
47697

1
0
subfamily
3

 0.00
54
47698

47699
2

 0.00
54
genus
1
0

54

 0.00
1
586833
species

1489872

 0.06
299
1686
clade
1
339

0
1
clade
17
10

 0.00
1489883


 0.00
9
17
129912
1
0
order

1489884
17
8

 0.00
suborder
0
1

17

 0.00
7
72045
0
1
family

1
0
subfamily

 0.00
3
9
129917

genus
0
1
72046
9

 0.00
2

109280
9
1

 0.00
species

subfamily
0
1
129914
8
3

 0.00

103719
8

 0.00
2
genus
1
1


 0.00
1
7
161584
species

0
1
clade
42
13

 0.00
1489875

1489876

 0.00
6
41
order
1
0

5

 0.00
41
1489877
1
0
suborder

0
1
family
41
4

 0.00
83881

475176

 0.00
3
41
subfamily
1
0

1
0
genus
2

 0.00
41
375763

375764
41
1

 0.00
species

order
0
1
1489878
1
6

 0.00

8219
1

 0.00
5
suborder
0
1

1
0
family
4

 0.00
1
8220

497678
1
3

 0.00
subfamily
0
1

1

 0.00
2
86204
0
1
genus

1

 0.00
1
409849
species

clade
1
0
1489885

 0.00
8
87

order
1
0
1489894

 0.00
7
87

8224

 0.00
6
87
family
1
0

87
5

 0.00
186745
0
1
subfamily

186749
4

 0.00
87
tribe
1
0

87
3

 0.00
8234
58
1
genus

species
8240

 0.00
1
14

8236
15
1

 0.00
species

1
3
clade
44

 0.01
192
1489904

1489907
11

 0.00
87
order
1
0

8157
57

 0.00
7
family
0
1

8160
2
4

 0.00
genus
0
1

species
1
0
302047
2

 0.00
1

1
1

 0.00
1841481
subspecies


 0.00
1
1
41447
species

1
0
genus
2

 0.00
55
36211

36212
1

 0.00
55
species

0
1
family
30

 0.00
3
173245

genus
0
1
173246
30

 0.00
2

species
30

 0.00
1
173247

8252
88

 0.00
18
order
0
1

88
17

 0.00
30942
12
1
suborder

0
1
family
3

 0.00
3
8256

8266
2

 0.00
3
genus
1
1

2

 0.00
1
195615
species

family
0
1
171414
2
3

 0.00

2

 0.00
2
8254
1
0
genus


 0.00
1
2
8255
species

52902
37

 0.00
3
family
0
1


 0.00
2
37
52903
1
0
genus

species
52904
37
1

 0.00

family
0
1
30948
19
3

 0.00

19

 0.00
2
28828
0
1
genus

19

 0.00
1
28829
species

1
0
family

 0.00
4
15
30947

0
1
subfamily
15
3

 0.00
603456

2

 0.00
15
106173
1
0
genus

244447
15
1

 0.00
species

0
1
no rank
12

 0.00
10
1489905

family
1
0
27769
3

 0.00
1

1
0
genus

 0.00
2
1
13691

1

 0.00
1
443726
species

30876
5
3

 0.00
family
0
1

5
2

 0.00
270536
0
1
genus


 0.00
1
5
941984
species

family
0
1
8184
6

 0.00
3

8186

 0.00
2
6
genus
1
0

1

 0.00
6
8187
species

2

 0.00
4
1489906
0
1
order

2

 0.00
3
8243
0
1
family

2
2

 0.00
8244
0
1
genus

2
1

 0.00
8245
species

1489908
95

 0.01
339
clade
1
16

superorder
1
0
1489910

 0.00
19
44

order
1
0
1489911
18

 0.00
44

8113
44
17

 0.00
family
2
1

319056

 0.00
5
35
clade
1
0

subfamily
1
0
318559

 0.00
4
35

318529
35

 0.00
3
tribe
0
1

61816
35

 0.00
2
genus
0
1

species
35

 0.00
1
63155

clade
0
1
319095
7
11

 0.00

10

 0.00
7
318546
1
3
subfamily

319058
1
3

 0.00
tribe
0
1

195936

 0.00
2
1
genus
1
0

1

 0.00
1
303518
species

2
3

 0.00
319057
0
1
tribe

8155
2

 0.00
2
genus
0
1

species
2
1

 0.00
43689

tribe
1
0
319069

 0.00
3
1


 0.00
2
1
32506
1
0
genus

1

 0.00
1
32507
species

clade
1
0
1489920

 0.00
12
70

order
1
1
1489921
70
11

 0.00

suborder
0
1
123349
38

 0.00
5

family
1
0
63826

 0.00
4
38

3

 0.00
38
557415
1
0
subfamily

210581
2

 0.00
38
genus
1
0

species
38

 0.00
1
441366

1
0
suborder
5

 0.00
31
56717

56718
4

 0.00
31
family
1
0


 0.00
3
31
703913
1
0
subfamily

genus
0
1
94311
31
2

 0.00

31
1

 0.00
181472
species

clade
1
0
1489919

 0.00
5
2

2

 0.00
4
41872
0
1
order


 0.00
3
2
8189
1
0
family

2

 0.00
2
8190
0
1
genus

species
48193
2
1

 0.00

1489909
9

 0.00
27
no rank
1
0

family
1
0
30863
5

 0.00
3

genus
1
0
80969
2

 0.00
2

1

 0.00
2
80972
species

genus
0
1
80965
1

 0.00
2

1

 0.00
1
80966
species

205120
24

 0.00
3
family
0
1

24

 0.00
2
210631
0
1
genus

species

 0.00
1
24
210632

superorder
3
1
1489913
180
49

 0.01

order
0
1
28738
57
34

 0.00


 0.00
11
6
45443
1
0
suborder

5

 0.00
7
28771
0
1
family

1
2

 0.00
326431
0
1
genus

species
1
1

 0.00
37003

genus
0
1
942014
1

 0.00
2

species
451745
1
1

 0.00

genus
1
0
52669

 0.00
2
3

52670
1

 0.00
3
species

1
0
family

 0.00
3
1
405002

genus
0
1
28779
1

 0.00
2

1

 0.00
1
105023
species

8087
22

 0.00
51
suborder
1
0

8076
2
5

 0.00
family
0
1

subfamily
1
0
136836
4

 0.00
2

136838
3

 0.00
2
tribe
1
0

2

 0.00
2
28741
1
0
genus


 0.00
1
2
28743
species

0
1
family
2

 0.00
3
28758

1
0
genus
2

 0.00
2
208332

species
208333
2

 0.00
1

28756
5

 0.00
3
family
1
0


 0.00
2
1
232976
1
0
genus

species

 0.00
1
1
232977

2
2

 0.00
8077
0
1
genus

species

 0.00
1
2
8078

family
0
1
8079
44

 0.00
8

7

 0.00
44
586240
1
1
subfamily

8080
3

 0.00
26
genus
1
0

species
8081

 0.00
1
24

species
1

 0.00
2
48701

3

 0.00
1
8082
genus

33527
14

 0.00
2
genus
0
1

species
14

 0.00
1
33528

7

 0.00
54
8075
1
1
order

238703
33

 0.00
3
family
0
1

270533

 0.00
2
33
genus
1
0

species
446457

 0.00
1
33

1
0
family
3

 0.00
20
270656

300305
20
2

 0.00
genus
0
1

species
20
1

 0.00
300306

66

 0.00
7
76071
0
1
order

66

 0.00
6
28781
0
1
suborder

1
0
family

 0.00
5
66
47757


 0.00
4
66
8088
1
0
subfamily

0
1
genus
66

 0.00
3
8089

30732
1

 0.00
3
species

species

 0.00
1
63
8090

42
1
clade
572

 0.02
99
1489922

order
0
1
1489939
96
4

 0.00

1204718
96

 0.00
3
family
0
1

genus
1
0
8163

 0.00
2
96

species
315492

 0.00
1
96

order
1
0
1545895
4

 0.00
3

3
3

 0.00
30828
0
1
family

genus
1
0
109904
2

 0.00
3


 0.00
1
3
109905
species

order
1
5
8111

 0.01
56
265

1
0
suborder
8

 0.00
3
8112

8165
3
7

 0.00
family
0
1

641308

 0.00
3
1
subfamily
1
0

1
0
genus

 0.00
2
1
283033

species
283035

 0.00
1
1

subfamily
1
0
641307
3

 0.00
2


 0.00
2
2
8166
1
1
genus

1

 0.00
1
8167
species

8205
10

 0.00
60
suborder
1
1

family
0
1
36203
23
3

 0.00

23

 0.00
2
56715
0
1
genus

23
1

 0.00
56716
species

8206
3

 0.00
1
family
1
0

genus
0
1
35729
1

 0.00
2


 0.00
1
1
40690
species

family
1
0
30806

 0.00
3
35

35
2

 0.00
52238
0
1
genus

species
52239
35
1

 0.00

suborder
0
1
8107
5

 0.00
5

1
0
family

 0.00
4
5
274692

274705
5

 0.00
3
subfamily
0
1

genus
1
0
34820

 0.00
2
5

species
72105
5
1

 0.00

suborder
0
1
1489943
124

 0.00
9


 0.00
8
124
30871
1
0
family

7

 0.00
124
274794
1
0
subfamily

1
0
tribe

 0.00
6
124
1505891

3

 0.00
53
94231
1
2
genus

species
1
1

 0.00
310571

293821
1

 0.00
50
species

genus
0
1
134629
71
2

 0.00

71
1

 0.00
160734
species

suborder
2
1
8100
68

 0.00
23

1
0
infraorder
8

 0.00
33
8192

family
0
1
56724
32

 0.00
4

3

 0.00
32
181468
1
0
subfamily

56725

 0.00
2
32
genus
1
0

32

 0.00
1
56726
species

1
0
family
3

 0.00
1
8202

1
0
genus
2

 0.00
1
433404

433405

 0.00
1
1
species

1490020
7

 0.00
6
infraorder
0
1


 0.00
5
7
69291
1
0
family

0
1
genus
2

 0.00
2
69292

species
69293
1

 0.00
2

1
0
genus
2

 0.00
5
134919

134920
5
1

 0.00
species

infraorder
1
0
1490021
8

 0.00
26

1
4

 0.00
8101
0
1
family

1
3

 0.00
181456
0
1
subfamily

genus
0
1
8102
1

 0.00
2

species
8103
1
1

 0.00

0
1
family
25

 0.00
3
8092

61642

 0.00
2
25
genus
1
0

61643
25
1

 0.00
species

11

 0.00
4
1489928
0
1
order

1
0
family
3

 0.00
11
8247

genus
0
1
98381
11
2

 0.00

species
1

 0.00
11
1203425

1
0
order
9

 0.00
7
1489940

1545897

 0.00
8
7
superfamily
1
0

0
1
family
1
3

 0.00
119486

1
2

 0.00
119487
0
1
genus


 0.00
1
1
119488
species

1
0
family

 0.00
4
6
8180

27705
6
3

 0.00
genus
2
1

27706
1

 0.00
3
species

147949

 0.00
1
1
species

order
1
0
1489931
6

 0.00
60

family
2
1
8169
60
5

 0.00

genus
1
0
8174
2

 0.00
37

species
8175
1

 0.00
37

2

 0.00
21
8176
1
0
genus

21

 0.00
1
8177
species

no rank
1
0
1489923
9

 0.00
74

1
0
family
3

 0.00
6
30869

1
0
genus

 0.00
2
6
75037

6
1

 0.00
75038
species


 0.00
5
68
30870
1
2
family


 0.00
2
39
215357
1
0
genus

species

 0.00
1
39
215358

27

 0.00
2
215359
0
1
genus

species
240163
27

 0.00
1

31022
6

 0.00
14
order
1
0

31028

 0.00
5
14
suborder
1
0


 0.00
4
14
32517
1
0
superfamily

family
0
1
31031
14
3

 0.00

genus
1
0
31032
2

 0.00
14

14

 0.00
1
31033
species

clade
0
1
1489874
31

 0.00
6

8064
31
5

 0.00
order
0
1

8065
4

 0.00
31
family
1
0

subfamily
1
0
390319
3

 0.00
31


 0.00
2
31
289381
1
0
genus

1

 0.00
31
390379
species

1489892
67
23

 0.00
clade
0
1

48

 0.00
13
1489900
0
1
order

1
0
suborder

 0.00
8
46
50370

8
4

 0.00
270602
0
1
family

158449
3

 0.00
8
subfamily
1
0


 0.00
2
8
158455
1
0
genus

1

 0.00
8
158456
species

38

 0.00
3
64142
0
1
family


 0.00
2
38
64143
1
0
genus

species
38
1

 0.00
64144

1
0
suborder

 0.00
4
2
50374

family
0
1
30910
2
3

 0.00

2

 0.00
2
33789
1
0
genus

species
215402

 0.00
1
2

1
0
order

 0.00
9
19
43697

suborder
1
0
129920
4

 0.00
16

1
0
family
3

 0.00
16
94233

16
2

 0.00
94234
0
1
genus

205130
16

 0.00
1
species

suborder
0
1
129918
3
4

 0.00

43698

 0.00
3
3
family
1
0

3

 0.00
2
43699
0
1
genus

species
43700
3
1

 0.00

clade
1
0
1489798

 0.00
6
2

order
1
0
41711
5

 0.00
2

8012

 0.00
4
2
family
1
0

1
0
subfamily
3

 0.00
2
182238

0
1
genus
2

 0.00
2
137519

137520
2
1

 0.00
species

clade
1
0
1489343

 0.00
11
36

clade
0
1
31089
36

 0.00
10

36
9

 0.00
41712
0
1
order

32
3

 0.00
27723
0
1
family

27726

 0.00
2
32
genus
1
0

species
1

 0.00
32
113540

0
1
family
4
5

 0.00
31092

91732
2
2

 0.00
genus
0
1

1676925
1

 0.00
2
species

0
1
genus
2

 0.00
2
42634

2
1

 0.00
42636
species

0
1
infraclass
4

 0.00
5
1489100

0
1
order
4
4

 0.00
7914

7915
4

 0.00
3
family
0
1

0
1
genus
4
2

 0.00
7916

species
4

 0.00
1
7918

0
1
subclass
72
13

 0.00
32440

7899

 0.00
12
72
order
1
0

1
0
suborder
11

 0.00
72
186622

family
1
0
7900

 0.00
5
68

subfamily
0
1
124129
68

 0.00
4

tribe
1
0
124130
3

 0.00
68

68

 0.00
2
7901
0
1
genus

species
7906
68
1

 0.00

family
0
1
7911
4

 0.00
5

subfamily
1
0
186619

 0.00
4
4

0
1
tribe
4

 0.00
3
186621

7912
4
2

 0.00
genus
0
1

1

 0.00
4
7913
species

8287
156479

 6.03
681
superclass
3
1

clade
1
3
1338369

 6.03
674
156472

1
81
clade
673

 6.03
156469
32523

8292
33

 0.00
37
class
1
0

27

 0.00
10
8445
2
1
order

264006
9
3

 0.00
family
0
1

0
1
genus
9
2

 0.00
260994

species
260995

 0.00
1
9

3

 0.00
5
1277737
1
0
family

1
0
genus

 0.00
2
5
264009

5

 0.00
1
1415580
species

11
3

 0.00
30380
0
1
family

11

 0.00
2
194407
0
1
genus

194408

 0.00
1
11
species

22

 0.00
10
41666
1
0
superorder

1
0
order
21

 0.00
10
8342

1
0
superfamily
8

 0.00
2
30319

family
1
0
8352
7

 0.00
2


 0.00
6
2
8360
1
0
subfamily

8353
5

 0.00
2
genus
1
0

subgenus
0
1
262014
1

 0.00
2


 0.00
1
1
8355
species

8363
1
2

 0.00
subgenus
0
1

1

 0.00
1
8364
species

suborder
0
1
8416
8
12

 0.00

superfamily
1
0
8417

 0.00
11
8

3
3

 0.00
8382
0
1
family

8383
3
2

 0.00
genus
2
1

8384
1

 0.00
1
species

611790
1
4

 0.00
family
0
1

0
1
subfamily
1
3

 0.00
611792

1

 0.00
2
449235
0
1
genus

448617
1
1

 0.00
species

4
3

 0.00
192735
0
1
family

8419
4

 0.00
2
genus
0
1

species

 0.00
1
4
248795


 6.03
639
156351
32524
1
624
clade

1
11
class

 5.97
402
154931
40674

clade
1
45
32525

 5.97
393
154916

9347

 5.97
366
154825
clade
1
66

superorder
1
0
9348
5

 0.00
4

order
0
1
948951
4

 0.00
4


 0.00
3
4
9359
1
0
family

2

 0.00
4
9360
1
0
genus

9361
4

 0.00
1
species

14
24

 0.00
311790
0
1
superorder


 0.00
4
3
9369
1
0
family

176113
3

 0.00
3
subfamily
0
1

genus
1
0
9370
2

 0.00
3

3

 0.00
1
9371
species

order
1
0
9774
5

 0.00
4

0
1
family
4
4

 0.00
9775

9776

 0.00
3
4
genus
1
0

0
1
species
4

 0.00
2
9778

subspecies
4
1

 0.00
127582


 0.00
5
1
9815
1
0
order

family
1
0
9816
4

 0.00
1

9817
3

 0.00
1
genus
1
0

9818
1

 0.00
2
species
0
1

subspecies
1230840
1

 0.00
1

28734
4

 0.00
1
order
1
0

28735
1

 0.00
3
family
0
1

28736
1

 0.00
2
genus
0
1

species

 0.00
1
1
28737

order
1
0
9779
5

 0.00
5

5

 0.00
4
9780
0
1
family

9782
5
3

 0.00
genus
0
1

2

 0.00
5
9783
1
2
species

99487
3
1

 0.00
subspecies

1021
1
clade
154741

 5.96
336
1437010

314145
1275
147

 0.05
superorder
69
1

order
1
0
9362

 0.00
6
3

0
1
family
3
5

 0.00
9373

genus
1
0
9374

 0.00
2
2

species
1

 0.00
2
50954

genus
0
1
143301
1
2

 0.00

species
1

 0.00
1
143302

order
1
0
9971

 0.00
4
4

4

 0.00
3
9972
0
1
family

1
1
genus

 0.00
2
4
9973

species
9974

 0.00
1
3

9787

 0.00
6
33
order
1
0

9788
33
5

 0.00
family
0
1

1
4
genus

 0.00
4
33
9789

1

 0.00
6
9793
species

9796
22

 0.00
1
species

species
1
1

 0.00
9798

order
1
0
9397
41

 0.00
74

0
1
suborder
62

 0.00
32
30560

family
0
1
186994
1

 0.00
3

1

 0.00
2
58068
0
1
genus

186990

 0.00
1
1
species

49
11

 0.00
9431
0
1
family

1
0
subfamily

 0.00
3
2
981671

9432
2
2

 0.00
genus
0
1

species
291302
1

 0.00
2

genus
1
1
9434
3

 0.00
5

species
1

 0.00
3
51298


 0.00
1
1
109478
species

29077

 0.00
2
1
genus
1
0

species
29078
1

 0.00
1

0
1
genus
41
2

 0.00
27671

species
1

 0.00
41
59474

5

 0.00
5
58055
1
0
family

5

 0.00
4
186995
0
1
subfamily

1
0
genus
3

 0.00
5
49442

species
1

 0.00
3
89399

2

 0.00
1
59479
species

3

 0.00
2
9436
1
0
family

1
0
genus

 0.00
2
2
27621

2

 0.00
1
27622
species

0
1
family
5
9

 0.00
9415

40234
2
5

 0.00
subfamily
0
1

genus
0
1
9416
1
2

 0.00

9417
1
1

 0.00
species

genus
1
0
27659

 0.00
2
1

species
1

 0.00
1
192404

subfamily
1
0
40238

 0.00
3
3

3

 0.00
2
9422
0
1
genus

9423
1

 0.00
3
species

30559
8

 0.00
12
suborder
1
0

family
0
1
9398
12

 0.00
7

77225
6

 0.00
12
subfamily
1
0

9

 0.00
3
9401
0
1
genus

species
9402
1

 0.00
1

143291

 0.00
1
8
species

genus
1
0
9406
2

 0.00
3

9407
1

 0.00
3
species

1
14
order

 0.03
46
652
91561

9845
527
21

 0.02
suborder
0
1

infraorder
64
1
35500
527

 0.02
20


 0.00
4
84
9850
1
2
family

34878

 0.00
3
82
subfamily
1
0

genus
1
1
9859

 0.00
2
82

species
1

 0.00
81
9860

family
1
43
9895

 0.01
15
379

subfamily
7
1
27592
211

 0.01
6

genus
1
145
9903
5

 0.01
204

1705790
5
1

 0.00
species


 0.00
1
24
72004
species

9913
1

 0.00
29
species

species
1

 0.00
1
30522

0
1
subfamily
1

 0.00
3
9959

0
1
genus
1

 0.00
2
9957

species
1

 0.00
1
59534

0
1
subfamily
124

 0.00
5
9963

9935
4

 0.00
124
genus
1
5

species
11

 0.00
1
9940

species
0
1
37174
108
2

 0.00

subspecies
112262
108
1

 0.00

suborder
0
1
9834
7
6

 0.00


 0.00
5
7
9835
1
1
family

2
2

 0.00
30539
0
1
genus

30538

 0.00
1
2
species

1
0
genus

 0.00
2
4
9836

4
1

 0.00
419612
species

0
1
suborder
37
13

 0.00
2653789

infraorder
1
0
9721

 0.00
12
37

9722
32
7

 0.00
parvorder
3
1

1
1
family
28
3

 0.00
9726

genus
1
0
9732
2

 0.00
27

species
27
1

 0.00
9733

119500
1
3

 0.00
family
0
1

118796
1

 0.00
2
genus
0
1

1

 0.00
1
118797
species

9761
5
4

 0.00
parvorder
0
1

1
0
family
3

 0.00
5
9765


 0.00
2
5
9766
1
0
genus

species
9771
5

 0.00
1

35497

 0.00
5
67
suborder
1
0

9821
4

 0.00
67
family
1
1

genus
0
1
9822
66
3

 0.00

9823
66
2

 0.00
species
39
1

subspecies
27
1

 0.00
415978

33554

 0.02
43
440
order
1
5

15

 0.00
77
379583
1
0
suborder

1
4
family

 0.00
8
69
9681

subfamily
1
2
338152

 0.00
3
61

9682

 0.00
2
59
genus
1
0

species
9685

 0.00
1
59

4

 0.00
4
338153
0
1
subfamily


 0.00
3
4
9688
1
0
genus

species
29064

 0.00
1
3

9691
1
1

 0.00
species

6

 0.00
3
9697
0
1
family

1
0
genus
2

 0.00
6
37031

37032
6
1

 0.00
species

3

 0.00
2
9676
1
0
family

1
0
genus
2

 0.00
2
95911

species
95912
2
1

 0.00

27

 0.01
358
379584
1
78
suborder

2

 0.00
3
9702
1
1
family

1
0
genus

 0.00
2
1
34883

species
1
1

 0.00
34884

4

 0.01
197
9608
1
3
family

9611

 0.01
3
194
genus
1
0

1
176
species
2

 0.01
194
9612


 0.00
1
18
9615
subspecies

73
11

 0.00
9655
12
1
family

subfamily
0
1
1008252
30
3

 0.00

30

 0.00
2
9661
0
1
genus

1

 0.00
30
9662
species

subfamily
0
1
169417
28

 0.00
3

9656

 0.00
2
28
genus
1
0

species
28
1

 0.00
9657

0
1
subfamily
3

 0.00
4
169418


 0.00
3
3
9665
1
0
genus

species
1
0
9668

 0.00
2
3

subspecies
1

 0.00
3
9669

9709
3

 0.00
3
family
1
2

1
0
genus

 0.00
2
1
9714

species
1

 0.00
1
9715

9632
5

 0.00
5
family
0
1

1
0
genus

 0.00
2
2
9645

species
9646
2

 0.00
1

genus
1
2
9639
2

 0.00
3

29073
1

 0.00
1
species

1
776
superorder
188

 5.88
152445
314146

16
1
clade
135401
117

 5.22
314147

order
782
1
9989
135380

 5.22
108


 0.00
17
32
33553
1
0
suborder

55153

 0.00
16
32
family
1
0

subfamily
1
0
337726
10

 0.00
5

0
1
tribe
5
9

 0.00
337730

1
0
genus
2

 0.00
1
1141640

1

 0.00
1
43179
species

genus
0
1
1141645
2
2

 0.00

9999
1

 0.00
2
species

0
1
genus
2
4

 0.00
9992

0
1
species
1
2

 0.00
9993

subspecies
1
1

 0.00
9994

species
1

 0.00
1
9995

subfamily
1
0
9991

 0.00
5
27

tribe
1
0
337752

 0.00
4
27

1
6
genus
3

 0.00
27
10001

species
1

 0.00
8
55149

species
30640

 0.00
1
13

1963757

 0.00
11
7
suborder
1
0

family
1
0
10015

 0.00
7
5

1
0
subfamily
2

 0.00
4
38662

genus
1

 0.00
4
10016

0
1
subfamily
1

 0.00
4
38663


 0.00
3
1
37442
1
0
genus

2

 0.00
1
38669
1
0
species

214514

 0.00
1
1
subspecies

family
1
0
29132

 0.00
3
2

10184
2
2

 0.00
genus
0
1

species
51338
1

 0.00
2

134445
66

 5.18
1963758
100
1
suborder

6730
1
clade
134345
65

 5.18
337687


 4.91
34
127315
10066
1
5374
family

10045
3

 0.00
6
subfamily
1
0

genus
0
1
10046
6
2

 0.00

species
6
1

 0.00
10047

326408
121115
12

 4.67
subfamily
0
1

24781
1
genus
121115

 4.67
11
10067

63
1

 0.00
10068
species

92878
38

 0.00
1
species

3
2

 0.00
2642879
0
1
no rank


 0.00
1
3
60743
species

species
645539
1

 0.00
1

species
1

 0.00
1
2866131

83527
1279

 0.05
1
species


 0.55
1
14269
60744
species

60746
70901
1

 2.73
species


 0.38
1
9779
83762
species


 0.03
18
820
39107
1
116
subfamily

genus
0
1
30639
3
2

 0.00

35658
1

 0.00
3
species

66

 0.00
3
10128
0
1
genus

66
2

 0.00
400053
0
1
species group

species
10129
66

 0.00
1

genus
0
1
61153
4

 0.00
2

4
1

 0.00
61156
species

10088
6

 0.02
590
genus
1
3

70
1
subgenus
587

 0.02
5
862507

10096
11

 0.00
1
species

501
1
species
506

 0.02
3
10090

subspecies

 0.00
1
3
10091

subspecies

 0.00
1
2
57486

4

 0.00
2
121588
0
1
genus

4

 0.00
1
491861
species

2
1
genus
37
2

 0.00
10114

species
10116
35

 0.00
1

337677

 0.01
26
299
family
1
23

subfamily
8
1
39087
48
7

 0.00

30
2

 0.00
10049
0
1
genus

species
1047088

 0.00
1
30

447134
5
2

 0.00
genus
0
1

447135
5

 0.00
1
species

10053
5

 0.00
2
genus
2
1

1

 0.00
3
100897
species

1
3

 0.00
40141
0
1
subfamily

10069

 0.00
2
1
genus
1
0

species

 0.00
1
1
10074

1
0
subfamily
7

 0.00
17
10026

genus
1
0
10035
2

 0.00
5

species

 0.00
1
5
10036

genus
0
1
10028
4

 0.00
2

species
10029
4

 0.00
1

genus
0
1
10043
8

 0.00
2

109678
8
1

 0.00
species

subfamily
4
1
337963
210

 0.01
8

0
1
genus
195
2

 0.01
38667

195
1

 0.01
38674
species

10040
11

 0.00
5
genus
1
1

species
0
1
10042
3
3

 0.00

subspecies

 0.00
1
1
44239

subspecies
230844

 0.00
1
2

10041

 0.00
1
7
species

0
1
family
1
4

 0.00
337664

1
0
subfamily

 0.00
3
1
10061

30636
1
2

 0.00
genus
0
1

species
1026970
1

 0.00
1

33550
114

 0.00
13
suborder
1
1

10161
1

 0.00
3
family
0
1

genus
1
0
10162

 0.00
2
1

species
1

 0.00
1
10163

1
0
family
3

 0.00
1
10150

1
0
genus

 0.00
2
1
10151

34839

 0.00
1
1
species

0
1
family
110
3

 0.00
10167

10180
110
2

 0.00
genus
0
1

species
110

 0.00
1
10181

1
3

 0.00
10158
0
1
family

0
1
genus
1
2

 0.00
10159

species
1

 0.00
1
10160

1
0
order

 0.00
8
5
9975

0
1
family
4

 0.00
4
9976

9977
4
3

 0.00
genus
0
1

130825
1

 0.00
3
species

9978

 0.00
1
1
species

family
1
0
9979
3

 0.00
1

9984

 0.00
2
1
genus
1
0

9986
1

 0.00
1
species

30656
2
4

 0.00
order
0
1

30657
2

 0.00
3
family
1
1

genus
0
1
482536
1

 0.00
2

species
482537

 0.00
1
1

1
0
order

 0.00
4
3
9392

3
3

 0.00
9393
0
1
family

9394
3

 0.00
2
genus
0
1

37347

 0.00
1
3
species

9443
62

 0.63
16263
order
1
0

376911
12

 0.00
5
suborder
1
0

1
0
infraorder

 0.00
7
4
376915

1
0
family

 0.00
3
3
9445

3
2

 0.00
9446
0
1
genus

1

 0.00
3
9447
species

30615

 0.00
3
1
family
1
0

1
2

 0.00
13149
0
1
genus

species
1

 0.00
1
30608

1

 0.00
4
376917
0
1
infraorder

3

 0.00
1
40297
1
0
family

genus
1
0
30610

 0.00
2
1

30611
1
1

 0.00
species

suborder
0
1
376913
16258

 0.63
49

314293
48

 0.63
16258
infraorder
1
21

9479

 0.00
6
1
parvorder
1
0

9498
1

 0.00
5
family
0
1

9480
4

 0.00
1
subfamily
1
0

1
3

 0.00
9481
0
1
genus


 0.00
2
1
1965096
1
0
subgenus

species
9483

 0.00
1
1

1
71
parvorder

 0.63
41
16236
9526

superfamily
0
1
314294
19
21

 0.00

19

 0.00
20
9527
0
1
family

9528

 0.00
11
15
subfamily
1
0

genus
1
1
9539

 0.00
6
11

species

 0.00
1
3
9541

species
9545
2

 0.00
1

4

 0.00
1
9544
species

1
2

 0.00
54602
0
1
species

subspecies
257877

 0.00
1
1

1
0
genus
2

 0.00
1
9529

1

 0.00
1
9531
species

3
2

 0.00
392815
0
1
genus

3
1

 0.00
9534
species

1
0
subfamily
8

 0.00
4
9569

2

 0.00
1
591932
1
0
genus

species
1
1

 0.00
591936

1
3

 0.00
9570
0
1
genus

2

 0.00
1
54131
1
0
species

1
1

 0.00
336983
subspecies

2

 0.00
2
54136
1
0
genus

54180
2

 0.00
1
species

16146

 0.62
19
314295
31
1
superfamily

9577
5

 0.00
5
family
1
0

9578
2
2

 0.00
genus
0
1

1

 0.00
2
81572
species


 0.00
2
3
325165
1
0
genus


 0.00
1
3
61853
species

59
1
family
16110
13

 0.62
9604

607660
3

 0.00
10
subfamily
1
0

genus
1
0
9599
2

 0.00
10

9601
10

 0.00
1
species

subfamily
1008
1
207598
16041

 0.62
9

14990

 0.58
2
9605
0
1
genus

species
14990

 0.58
1
9606


 0.00
3
3
9592
1
0
genus

1
1
species

 0.00
2
3
9593

subspecies

 0.00
1
2
9595

40
3

 0.00
9596
1
1
genus

9597
2
1

 0.00
species

species
37

 0.00
1
9598


 0.00
26
46
9263
1
0
clade

order
0
1
38609
17

 0.00
10


 0.00
3
2
9307
1
0
family

1
0
genus
2

 0.00
2
1960649

species
9315
2

 0.00
1

0
1
family
12
3

 0.00
38624

12

 0.00
2
38625
0
1
genus

38626

 0.00
1
12
species

3

 0.00
3
9338
1
0
family

0
1
genus
3
2

 0.00
29138

3

 0.00
1
29139
species


 0.00
7
4
38605
1
0
order

1
0
family
6

 0.00
4
9265

1
0
subfamily

 0.00
5
4
126287

126288
1
2

 0.00
genus
0
1

species
1
1

 0.00
191870

1
0
genus
2

 0.00
3
13615

species
3
1

 0.00
13616

38608
24

 0.00
4
order
0
1

family
1
0
9277
3

 0.00
24

genus
0
1
9304
24

 0.00
2

species
9305
24
1

 0.00

1
4

 0.00
38607
0
1
order

0
1
family
1
3

 0.00
30660

genus
1
0
33561

 0.00
2
1

33562
1

 0.00
1
species

8

 0.00
4
9254
1
0
clade

4

 0.00
7
9255
0
1
order

3

 0.00
3
9256
1
0
family

genus
1
0
9257
2

 0.00
3

species
1

 0.00
3
9258

family
0
1
9259
1
3

 0.00

9260
1
2

 0.00
genus
0
1

9261
1
1

 0.00
species

clade
1
0
8457

 0.03
236
796

32561
796
235

 0.03
clade
1
1

0
1
class
30

 0.00
53
8504

52

 0.00
30
8509
1
0
order

1329961
30
51

 0.00
clade
0
1

infraorder
0
1
8560
1
5

 0.00

4

 0.00
1
8561
1
0
family

385256
1
3

 0.00
subfamily
0
1

0
1
genus
1
2

 0.00
401550

1

 0.00
1
1277458
species

1329950
29
45

 0.00
clade
0
1

0
1
clade
29

 0.00
44
1329912

1329911
24
37

 0.00
clade
0
1


 0.00
23
17
8570
1
0
infraorder

0
1
superfamily
2

 0.00
4
34979

2
3

 0.00
34984
0
1
family

0
1
genus
2
2

 0.00
37579

1

 0.00
2
176946
species

1
0
superfamily

 0.00
18
15
34989

6

 0.00
2
8602
1
0
family

subfamily
1
0
42167

 0.00
5
2

1

 0.00
2
8661
0
1
genus

8663
1

 0.00
1
species


 0.00
2
1
8672
1
0
genus

1

 0.00
1
8673
species

1
0
family
7

 0.00
8
8689

0
1
subfamily
1
3

 0.00
8710

8728
1

 0.00
2
genus
0
1

1

 0.00
1
88082
species

3

 0.00
7
8690
1
0
subfamily

0
1
genus
7

 0.00
2
8703

7
1

 0.00
103942
species

5
4

 0.00
8578
0
1
family

5
3

 0.00
169862
0
1
subfamily

34999
5

 0.00
2
genus
1
1

35019
1

 0.00
4
species

13

 0.00
7
8511
1
0
suborder

5

 0.00
4
2024743
0
1
family

5

 0.00
3
42425
0
1
subfamily

8518

 0.00
2
5
genus
1
0

8520
5

 0.00
1
species

clade
1
0
83232
5

 0.00
1

1

 0.00
4
81953
0
1
family

0
1
subfamily
1

 0.00
3
145349


 0.00
2
1
52201
1
0
genus

1

 0.00
1
103695
species


 0.00
3
1
2024747
1
0
family

genus
1
0
28376

 0.00
2
1

species

 0.00
1
1
28377

1329976
5

 0.00
6
clade
0
1

clade
0
1
1329975
5

 0.00
5

8522

 0.00
4
5
family
1
0

subfamily
1
1
162266
5
3

 0.00

0
1
genus
4

 0.00
2
42163

species
64176
4
1

 0.00

clade
1
1
1329799
181

 0.03
765

clade
1
1
8492
749
150

 0.03

149

 0.03
748
436486
1
0
clade

436489
748
148

 0.03
clade
0
1

1
0
clade

 0.03
147
748
436491


 0.03
146
748
436492
1
0
clade

8782
145

 0.03
748
class
1
6

infraclass
1
33
8825
130

 0.03
708


 0.00
3
3
8948
1
0
order

8949
3

 0.00
2
family
0
1

genus
8952
3
1

 0.00

order
1
0
2558200

 0.00
8
56

56259
7

 0.00
56
family
1
0

1
18
subfamily

 0.00
6
56
8955

8956
21

 0.00
2
genus
0
1

8957
21
1

 0.00
species

8960
3

 0.00
17
genus
1
0

species
0
1
8962
17

 0.00
2


 0.00
1
17
223781
subspecies

7
7

 0.00
9223
0
1
order


 0.00
3
3
1545690
1
0
family

0
1
genus
3
2

 0.00
13145

species
13146
1

 0.00
3

9224
4

 0.00
3
family
0
1

35547
4

 0.00
2
genus
0
1

1

 0.00
4
2489341
species

1
0
order
4

 0.00
6
8920

56295

 0.00
3
6
family
1
0

1
0
genus
2

 0.00
6
56296

240206

 0.00
1
6
species

order
1
0
8936

 0.00
4
1

0
1
family
1
3

 0.00
57383

1
2

 0.00
57405
0
1
genus

1

 0.00
1
176938
species

112
40

 0.00
9126
20
1
order

superfamily
0
1
192204
10
7

 0.00

family
0
1
28725
10

 0.00
6


 0.00
2
1
34923
1
0
genus


 0.00
1
1
34924
species

30420
3

 0.00
9
genus
1
4

species
1

 0.00
2
1196302

species
3
1

 0.00
68294

4

 0.00
13
175121
0
1
superfamily

family
1
0
9158
3

 0.00
1

187421
2

 0.00
1
genus
1
0

species

 0.00
1
1
44316

family
1
0
37611

 0.00
5
2

1
0
subfamily
4

 0.00
2
40155

2
3

 0.00
40156
0
1
genus

40157

 0.00
2
2
species
1
0

299123

 0.00
1
2
subspecies

0
1
family
1

 0.00
4
36256


 0.00
3
1
45806
1
0
genus

45807
2

 0.00
1
species
1
0

1094192
1

 0.00
1
subspecies

family
0
1
9183
49
5

 0.00

2

 0.00
48
36283
1
0
genus

48

 0.00
1
37610
species

1
0
genus

 0.00
2
1
9184

species
1
1

 0.00
91951

2
1
family
5
3

 0.00
400783

87174

 0.00
2
3
genus
1
0

87175
3

 0.00
1
species

0
1
family
3

 0.00
5
114313

1
0
genus

 0.00
2
1
196036

328815
1

 0.00
1
species

196026
2

 0.00
2
genus
0
1

species
1

 0.00
2
296741

21

 0.00
6
2116661
0
1
superfamily

family
1
0
36270
5

 0.00
21

4

 0.00
21
330750
1
0
subfamily

0
1
genus
21
3

 0.00
39620

0
1
species
21
2

 0.00
48156

subspecies
126889
1

 0.00
21

clade
0
1
2607030
15

 0.00
10

8902
14

 0.00
5
order
0
1

family
0
1
48283
14

 0.00
4

subfamily
0
1
48286
14
3

 0.00

48284
14

 0.00
2
genus
0
1

species
111811
1

 0.00
14

8892
1
4

 0.00
order
0
1

9242
3

 0.00
1
family
1
0

2

 0.00
1
9243
1
0
genus

species
1

 0.00
1
9244

order
0
1
8906
1
4

 0.00


 0.00
3
1
8907
1
0
family

1
2

 0.00
50391
0
1
genus

species
1

 0.00
1
50402

0
1
superorder
168

 0.01
32
1549675

8826
33

 0.00
11
order
0
1

33

 0.00
10
8830
3
1
family

subfamily
1
0
2068716
3

 0.00
20


 0.00
2
20
8835
1
0
genus

20

 0.00
1
8839
species

1
3
subfamily

 0.00
6
10
2068722

8842
2

 0.00
4
genus
1
0

8845

 0.00
1
4
species

genus
0
1
8867
3
3

 0.00

species
2
1

 0.00
8868

species
8869
1

 0.00
1

order
0
1
8976
135

 0.01
20

4

 0.00
3
8990
0
1
family

genus
0
1
8995
4

 0.00
2

species
1

 0.00
4
8996

family
1
1
9005
131
16

 0.01


 0.00
3
7
466544
1
0
subfamily

9090
7

 0.00
2
genus
0
1

species
93934

 0.00
1
7

1
0
subfamily
5

 0.00
81
9072

9030

 0.00
2
76
genus
1
0

9031
76

 0.00
1
species


 0.00
2
5
9053
1
0
genus

9054
1

 0.00
5
species

subfamily
1
0
466552

 0.00
3
40

1
0
genus

 0.00
2
40
9102

species
40
1

 0.00
9103

2
4

 0.00
466585
0
1
subfamily

2

 0.00
3
30409
0
1
genus

species
64668
1

 0.00
1

30410
1
1

 0.00
species

order
1
0
8929

 0.01
6
299

family
1
0
8930

 0.01
5
299

genus
0
1
36242
297
2

 0.01

species

 0.01
1
297
177155

2

 0.00
2
8931
0
1
genus

species
1

 0.00
2
8932

0
1
order
3
7

 0.00
9205

30445
3

 0.00
1
family
1
0

0
1
genus
1
2

 0.00
37044

species
1

 0.00
1
97097

family
0
1
33574
2

 0.00
3


 0.00
2
2
128389
1
0
genus

species
128390
1

 0.00
2

30458
4

 0.00
4
order
0
1

1
0
family
3

 0.00
4
30462

2

 0.00
4
56312
1
0
genus

species
56313
4

 0.00
1

superorder
1
0
8783
14

 0.00
34

4

 0.00
1
8784
1
0
order

family
0
1
8788
1

 0.00
3

8789

 0.00
2
1
genus
1
0

species
8790

 0.00
1
1

0
1
order
29
5

 0.00
8819


 0.00
4
29
8820
1
0
family

genus
1
0
8821

 0.00
3
29

2696672

 0.00
2
29
species
1
0

202946
29

 0.00
1
subspecies

order
1
0
8802

 0.00
4
4

4
3

 0.00
8803
0
1
family

8806
4
2

 0.00
genus
0
1

1

 0.00
4
30464
species

0
1
subclass
15

 0.00
30
2841271

15
29

 0.00
8459
0
1
order

1
3
suborder

 0.00
28
15
8464

1
2
clade

 0.00
23
11
1579337

5

 0.00
14
8486
0
1
superfamily

8476
1

 0.00
4
family
0
1

0
1
genus
1

 0.00
3
34902

34903
1
2

 0.00
species
0
1

subspecies
31138
1

 0.00
1

5

 0.00
3
8487
1
0
family

0
1
genus
2

 0.00
3
904181

no rank
1
0
1137846

 0.00
2
2

2

 0.00
1
106734
species

genus

 0.00
1
1
38771

family
1
0
328320
4

 0.00
1

subfamily
0
1
328321
1
3

 0.00

2

 0.00
1
74925
1
0
genus

74926
1

 0.00
1
species

1579336
8

 0.00
4
clade
1
0


 0.00
7
4
27791
1
1
superfamily

0
1
family
1

 0.00
3
8465

8466

 0.00
2
1
genus
1
0

species
8467
1
1

 0.00

27792
2
3

 0.00
family
0
1

27793

 0.00
2
2
genus
1
0

1

 0.00
2
27794
species

1
0
superfamily
4

 0.00
1
1579275

1

 0.00
3
34907
0
1
family

genus
0
1
204969
1
2

 0.00

1

 0.00
1
13735
species

clade
0
1
118072
4

 0.00
6

7894

 0.00
5
4
order
1
0

4

 0.00
4
7895
0
1
family

0
1
genus
4
3

 0.00
7896

species
1

 0.00
2
7897

106881
2
1

 0.00
species

7777
45
31

 0.00
class
0
1

subclass
0
1
7863
2
5

 0.00

0
1
order
2

 0.00
4
7864

7865
2
3

 0.00
family
0
1

0
1
genus
2

 0.00
2
7866

species
7868
1

 0.00
2

7778

 0.00
25
43
subclass
1
0

22

 0.00
5
117893
0
1
superorder

0
1
order
22

 0.00
4
7858

family
1
0
30475
3

 0.00
22

0
1
genus
22
2

 0.00
117853


 0.00
1
22
386614
species

21

 0.00
19
119203
0
1
infraclass

21

 0.00
18
119197
0
1
clade

119195
21
17

 0.00
superorder
0
1

30503
9

 0.00
7
order
0
1

0
1
family
4

 0.00
3
378069

1
0
genus

 0.00
2
4
378070

species
378071
1

 0.00
4

40580
3

 0.00
5
family
1
0

34767
5

 0.00
2
genus
0
1

36176
5

 0.00
1
species

0
1
order
1
5

 0.00
30496

family
1
0
7850

 0.00
4
1

subfamily
0
1
7844
1
3

 0.00

genus
1
0
13396
2

 0.00
1

species
1

 0.00
1
13397

1
0
order
4

 0.00
11
30483

family
1
0
7826
3

 0.00
11

11
2

 0.00
7829
0
1
genus

7830

 0.00
1
11
species

7712
29
19

 0.00
subphylum
0
1

0
1
class
11
5

 0.00
30302

0
1
order
11

 0.00
4
2507557

11
3

 0.00
41302
0
1
family

0
1
genus
11
2

 0.00
34763

34765
11

 0.00
1
species

class
1
0
7713

 0.00
13
18

4

 0.00
15
32436
1
0
order

1
0
family

 0.00
3
15
201955

201956
15
2

 0.00
genus
0
1

species
15

 0.00
1
2771288

7720
2
4

 0.00
order
0
1

7721
2

 0.00
3
family
0
1

7724
2

 0.00
2
genus
1
0

7725
2

 0.00
1
species

order
1
0
7716

 0.00
4
1

7717

 0.00
3
1
family
1
0

1
2

 0.00
7718
0
1
genus

7719
1

 0.00
1
species

0
1
subphylum
14
7

 0.00
7735

0
1
class
14

 0.00
6
2682552

0
1
order
14

 0.00
5
2682553

7736
14

 0.00
4
family
0
1

7737
3

 0.00
14
genus
1
0

species
7741
1

 0.00
1

1

 0.00
13
7740
species


 0.00
35
41
7586
1
0
phylum

1
0
clade

 0.00
8
3
133550

3
7

 0.00
35069
0
1
class

subclass
0
1
35070
3

 0.00
6

3
5

 0.00
7581
0
1
order

106121
3
4

 0.00
family
0
1

subfamily
1
0
1529449

 0.00
3
3

3

 0.00
2
1529434
0
1
genus

1529436
3

 0.00
1
species

clade
1
0
133551

 0.00
26
38

7624
8

 0.00
4
superclass
1
0

class
1
0
7625

 0.00
7
4

subclass
1
0
7638
6

 0.00
4


 0.00
5
4
7674
1
0
superorder

order
1
0
31184
4

 0.00
4

31185
4
3

 0.00
family
0
1

4

 0.00
2
7652
0
1
genus

species
7654
1

 0.00
4

34
17

 0.00
7587
0
1
superclass

class
0
1
7588
34
16

 0.00

superorder
0
1
41243
2

 0.00
8

1
0
order

 0.00
7
2
41166

0
1
family
1
3

 0.00
7592

1
2

 0.00
35076
0
1
genus


 0.00
1
1
46514
species

1
3

 0.00
133432
0
1
family

1
0
genus
2

 0.00
1
133433

1

 0.00
1
133434
species

41242
7

 0.00
32
superorder
1
0

order
0
1
7599
32
6

 0.00

7600
32

 0.00
5
family
0
1

7608

 0.00
2
6
genus
1
0

species
7609

 0.00
1
6

0
1
genus
26

 0.00
2
7601

species
1

 0.00
26
7604

5

 0.00
2
10219
1
0
phylum


 0.00
4
2
10220
1
0
class

family
0
1
10221
2
3

 0.00

genus
1
0
10222

 0.00
2
2

species
10224
2
1

 0.00

1
5629
clade

 1.50
2036
38914
33317

clade
2
1
2697495
1624

 0.06
246

clade
0
1
2697496
19

 0.00
8

phylum
1
0
10190
7

 0.00
19

class
1
0
2816136
6

 0.00
19


 0.00
5
19
44578
1
0
subclass

104779

 0.00
4
19
order
1
0

family
0
1
104780
19

 0.00
3

genus
0
1
104781
19

 0.00
2

species
19

 0.00
1
104782

1206795
237

 0.06
1603
clade
1
114

phylum
1
36
6447
121

 0.04
1064

6605
3
8

 0.00
class
0
1

6606
3

 0.00
7
subclass
0
1

superorder
0
1
215451
3

 0.00
6

6638
3
5

 0.00
order
0
1

4

 0.00
3
6646
1
0
suborder

3

 0.00
3
6647
0
1
family

genus
0
1
6643
3
2

 0.00

species
2607531
3

 0.00
1

6448
269

 0.01
60
class
4
1

subclass
1
0
216305
21

 0.00
50

216307
50

 0.00
20
clade
0
1

2836391
3
6

 0.00
clade
0
1

6497

 0.00
5
3
order
1
0

1
0
superfamily
4

 0.00
3
216318

1
0
family
3

 0.00
3
6498

3

 0.00
2
6499
0
1
genus

species
6500
1

 0.00
3

13

 0.00
47
977775
1
0
clade


 0.00
5
46
977779
1
0
clade

superfamily
0
1
216441
46
4

 0.00

3

 0.00
46
6524
1
0
family

2

 0.00
46
6525
1
0
genus

species
6526
1

 0.00
46

120490

 0.00
7
1
superorder
1
0

order
0
1
6527
1
6

 0.00

216366
5

 0.00
1
clade
1
0

4

 0.00
1
87871
1
0
superfamily

family
1
0
37859

 0.00
3
1

genus
1
0
1338343

 0.00
2
1

species
1338344

 0.00
1
1

1

 0.00
5
2219556
0
1
subclass

1
0
superfamily

 0.00
4
1
216274

55007
3

 0.00
1
family
1
0

1735270
1
2

 0.00
genus
0
1

species
1735272
1

 0.00
1

subclass
1
0
216275

 0.01
17
140

14
6

 0.00
2315723
0
1
order

216276

 0.00
5
14
superfamily
1
0

1
0
family

 0.00
4
14
6451

6452
14
3

 0.00
genus
2
1

6454
8

 0.00
1
species

species

 0.00
1
4
36100

order
1
0
2315720
10

 0.00
126

9

 0.00
126
216285
1
0
superfamily

126

 0.00
8
6466
0
1
family

7

 0.00
126
1955429
1
5
subfamily

148341
35
2

 0.00
genus
0
1

species
1

 0.00
35
703304

1
0
genus

 0.00
2
48
2072689

216125

 0.00
1
48
species

0
1
genus
38

 0.00
2
1093071

38

 0.00
1
1620919
species

subclass
0
1
69555
2

 0.00
6

1
0
order
5

 0.00
2
75116

1
0
superfamily

 0.00
4
2
6475

1
0
family
3

 0.00
2
54973


 0.00
2
2
72702
1
0
genus

species
400727
1

 0.00
2

69675
72
10

 0.00
subclass
1
1

1
0
superfamily
5

 0.00
64
146277

64

 0.00
4
6462
0
1
family

64

 0.00
3
6463
1
1
genus

29

 0.00
1
88005
species

6465

 0.00
1
34
species

7

 0.00
4
216260
0
1
superfamily

family
0
1
69676
7

 0.00
3

72691

 0.00
2
7
genus
1
0

225164
7

 0.00
1
species

6544
52

 0.03
756
class
1
0

subclass
1
18
2785011
51

 0.03
756


 0.02
29
542
6599
1
0
clade

infraclass
0
1
735337
542

 0.02
28

superorder
1
0
2785015
27

 0.02
542

44
1
clade
542

 0.02
26
2908833

order
0
1
6580
49
9

 0.00

4

 0.00
2
74489
1
0
superfamily

6592
2
3

 0.00
family
0
1

6595
2

 0.00
2
genus
1
0

species
6596
2

 0.00
1

47
4

 0.00
106231
0
1
superfamily

3

 0.00
47
6581
1
0
family

genus
1
0
6582
2

 0.00
47

31201

 0.00
1
47
species

2783445

 0.02
16
449
order
1
16

105710
11

 0.01
334
superfamily
1
0

10
1
family
334
10

 0.01
55708

2787994
245

 0.01
6
subfamily
4
1

genus
0
1
80817
23

 0.00
2

23

 0.00
1
80818
species

genus
1
0
52939

 0.01
3
218

80829
28

 0.00
1
species

species
80833
190

 0.01
1

0
1
subfamily
79

 0.00
3
2787989

80820

 0.00
2
79
genus
1
0

80821
79

 0.00
1
species

0
1
superfamily
99
4

 0.00
98297

1
0
family

 0.00
3
99
61354

99
2

 0.00
457750
0
1
genus

species

 0.00
1
99
2589376

1
3
clade

 0.01
21
196
6545

6546
50
6

 0.00
order
0
1

106220

 0.00
5
50
superfamily
1
0

50
4

 0.00
6547
0
1
family

subfamily
0
1
2899742
50

 0.00
3

genus
0
1
356392
50
2

 0.00

50

 0.00
1
356393
species

order
1
0
6562

 0.00
7
98

1
0
superfamily
6

 0.00
98
98302

family
0
1
6563
98
5

 0.00

4
2

 0.00
37858
0
1
genus


 0.00
1
4
37623
species

1
0
genus

 0.00
2
94
6564

29159
1

 0.00
94
species

0
1
order
45
7

 0.00
106218

106219

 0.00
6
45
superfamily
1
0

1
0
family

 0.00
5
45
6566

1
0
genus
2

 0.00
43
6578

species
6579
1

 0.00
43

genus
1
0
186466

 0.00
2
2

species
1

 0.00
2
6573

phylum
1
0
6217
6

 0.00
16

6218

 0.00
5
16
class
1
0

16

 0.00
4
6219
0
1
order

6222
16
3

 0.00
family
0
1

16

 0.00
2
6223
0
1
genus

species
16

 0.00
1
88925

10205
17

 0.00
41
phylum
1
0

class
1
0
10206
16

 0.00
41

1
2
order

 0.00
15
41
10207

suborder
0
1
558764
31
9

 0.00

1
0
superfamily
4

 0.00
20
193246

family
0
1
558762
20
3

 0.00

1
0
genus

 0.00
2
20
558754

species
20

 0.00
1
558755

0
1
superfamily
11

 0.00
4
193209

family
0
1
10210
11
3

 0.00

1970207
11
2

 0.00
genus
0
1

species
11

 0.00
1
192920

suborder
1
0
193205
5

 0.00
8

193206
4

 0.00
8
superfamily
1
0

8

 0.00
3
192924
0
1
family

95169
8
2

 0.00
genus
0
1


 0.00
1
8
95170
species

6340

 0.01
32
134
phylum
1
1

6341
72
12

 0.00
class
0
1

1
0
subclass
11

 0.00
72
105390

72

 0.00
10
6348
0
1
order

46593
49

 0.00
3
family
0
1


 0.00
2
49
222002
1
0
genus

species

 0.00
1
49
1210411

family
0
1
104728
3

 0.00
3

55701

 0.00
2
3
genus
1
0

1

 0.00
3
1210413
species

1
0
family

 0.00
3
20
39820

genus
0
1
868094
20
2

 0.00

880429
20

 0.00
1
species

0
1
class
61
19

 0.00
42113

subclass
0
1
55824
17
10

 0.00

order
0
1
6406
1

 0.00
4

1
0
family
3

 0.00
1
6407

6411
1

 0.00
2
genus
0
1

species
1
1

 0.00
6412

0
1
order
16

 0.00
5
2218736

0
1
suborder
16

 0.00
4
2218739

16
3

 0.00
60930
0
1
family

16
2

 0.00
60957
0
1
genus

species
60958
16
1

 0.00

0
1
subclass
44
8

 0.00
6381


 0.00
7
44
2803884
1
0
order

suborder
0
1
6391
44

 0.00
6

1
0
family
5

 0.00
44
6392

1046325
44
4

 0.00
subfamily
0
1

6397
44

 0.00
3
genus
0
1

no rank
0
1
1050932
44

 0.00
2

species
35632
44

 0.00
1


 0.00
8
1
7568
1
0
phylum

subphylum
1
0
115360

 0.00
7
1

class
1
0
115361

 0.00
6
1


 0.00
5
1
7570
1
0
order

1
0
superfamily
4

 0.00
1
115362

33491
1

 0.00
3
family
0
1


 0.00
2
1
7571
1
0
genus

1

 0.00
1
7574
species


 0.01
52
233
6157
1
3
phylum

6199
33
10

 0.00
class
0
1


 0.00
9
33
6200
1
0
subclass

order
0
1
6201
8

 0.00
4

family
1
0
6214
3

 0.00
8

0
1
genus
8

 0.00
2
6215


 0.00
1
8
85433
species

1224679
25
4

 0.00
order
0
1

28843
25

 0.00
3
family
0
1

genus
0
1
46580
25
2

 0.00

species
99802

 0.00
1
25

0
1
class
1

 0.00
8
147100

166126
1

 0.00
7
clade
0
1

0
1
order
1

 0.00
6
6159


 0.00
5
1
1292243
1
0
suborder

1
4

 0.00
1292248
0
1
superfamily

27896
1

 0.00
3
family
0
1

27899
1

 0.00
2
genus
0
1

1051007

 0.00
1
1
species

class
0
1
6178
196

 0.01
33

6179
196

 0.01
32
subclass
1
1

order
1
0
27871

 0.00
11
22

3

 0.00
5
27841
0
1
suborder

1
0
superfamily
4

 0.00
3
404429

family
0
1
27843
3
3

 0.00

6191

 0.00
2
3
genus
1
0

1

 0.00
3
6192
species

suborder
0
1
27872
19
5

 0.00

superfamily
0
1
1776223
19
4

 0.00

73421
3

 0.00
19
family
1
0

1
0
genus

 0.00
2
19
57077

species

 0.00
1
19
57078

6180
173
20

 0.01
order
0
1

0
1
superfamily
173
19

 0.01
31244

1
1
family
173
18

 0.01
31245

39198
2

 0.00
15
genus
1
0

species
15
1

 0.00
39320

2

 0.00
4
100601
1
0
genus

species
4
1

 0.00
157070

genus
1
49
6181
13

 0.01
153

1

 0.00
1
6182
species

4

 0.00
1
6187
species

1163369

 0.00
1
11
species

6186

 0.00
1
2
species


 0.00
1
7
393876
species

species
6

 0.00
1
31246

1

 0.00
14
6188
species


 0.00
1
21
6184
species

48269
17

 0.00
1
species

species
6183

 0.00
1
5

6

 0.00
1
6185
species

species
6189
1

 0.00
10

411
1
clade
31661
1789

 1.22
1206794

31140

 1.20
1732
88770
3
1
clade

1
0
phylum

 0.00
7
1
42241

0
1
class
1

 0.00
6
42242

1
0
order

 0.00
5
1
149990

2558949
4

 0.00
1
superfamily
1
0

1
3

 0.00
58669
0
1
family

genus
1
0
58670
2

 0.00
1

species
232323
1

 0.00
1

phylum
1
683
6656

 1.20
1724
31136

91

 0.01
152
6843
1
0
subphylum

5

 0.00
5
6844
1
0
class

6845
5
4

 0.00
order
0
1

1
0
family

 0.00
3
5
6846

6849
2

 0.00
5
genus
1
0

species
5

 0.00
1
6850

6
1
class
147
85

 0.01
6854

0
1
subclass
108

 0.00
58
6933

92

 0.00
35
6946
1
1
superorder

8
12

 0.00
83136
0
1
order

1
1
suborder
8

 0.00
11
6947

1
0
infraorder
4

 0.00
5
83138


 0.00
3
5
83141
1
2
clade

92088
3

 0.00
2
superfamily
0
1

family
92251
3

 0.00
1

infraorder
1
0
83145

 0.00
6
2

no rank
0
1
188550
2
5

 0.00

superfamily
1
0
83146
4

 0.00
2

3

 0.00
2
32262
1
0
family

32263
2

 0.00
2
genus
1
0


 0.00
1
2
32264
species

83137
83

 0.00
22
order
1
1

suborder
0
1
6951
37
13

 0.00

parvorder
1
2
223472

 0.00
12
37

superfamily
1
0
83163
6

 0.00
16

0
1
family
16
5

 0.00
6952

subfamily
1
0
474036

 0.00
4
16

0
1
genus
16
3

 0.00
6953

species

 0.00
1
13
6956

species
6954
3

 0.00
1

0
1
superfamily
19
5

 0.00
83158


 0.00
4
19
52281
1
0
family

3

 0.00
19
474019
1
0
subfamily

0
1
genus
19

 0.00
2
52282

species
52283

 0.00
1
19

66551
8

 0.00
45
suborder
1
0

229894
45
7

 0.00
infraorder
0
1

229794
45

 0.00
6
superfamily
0
1

family
1
1
229795
45
5

 0.00

334624
2

 0.00
28
genus
1
0

species

 0.00
1
28
334625

1979940
16
2

 0.00
genus
0
1

species
1979941

 0.00
1
16

6934
16

 0.00
22
superorder
0
1

6935
14

 0.00
9
order
1
0

297308
9

 0.00
13
superfamily
0
1

1
0
family

 0.00
12
9
6939

subfamily
0
1
426442
4

 0.00
3

0
1
genus
4

 0.00
2
6944

species

 0.00
1
4
6945

1
1
subfamily
4
5

 0.00
426437

34630
4

 0.00
3
genus
1
0

subgenus
0
1
426455
3

 0.00
3

578835
3

 0.00
2
species group
0
1


 0.00
1
3
34632
species

subfamily
1
0
426441

 0.00
3
1

6942
1

 0.00
2
genus
0
1

1

 0.00
1
34610
species

0
1
order
7

 0.00
7
34634

7
6

 0.00
281668
0
1
suborder

infraorder
1
0
1723665

 0.00
5
7

41438
7
4

 0.00
superfamily
0
1

family
1
0
109261
3

 0.00
7

62624
2

 0.00
7
genus
1
1

species
6
1

 0.00
62625

6855
6

 0.00
6
order
1
0

parvorder
0
1
259437
6
5

 0.00

superfamily
0
1
70336
6

 0.00
4

family
0
1
6856
6
3

 0.00

0
1
genus
6

 0.00
2
6875


 0.00
1
6
218467
species

27

 0.00
20
6893
0
1
order

0
1
suborder
27
19

 0.00
6905

clade
0
1
74971
27
18

 0.00

175332
1

 0.00
4
superfamily
0
1

family
1
0
175333
3

 0.00
1

genus
0
1
175340
1

 0.00
2


 0.00
1
1
202533
species

4

 0.00
5
94020
0
1
clade

clade
1
0
94015
4

 0.00
4

1
0
family

 0.00
3
4
152923

2

 0.00
4
152924
1
0
genus

2926465

 0.00
1
4
species

74974
22
8

 0.00
clade
0
1

22

 0.00
7
74975
0
1
superfamily

1
0
family
3

 0.00
6
34643

449632
6

 0.00
2
genus
0
1

6
1

 0.00
114398
species

16

 0.00
3
27394
0
1
family

1
0
genus

 0.00
2
16
94025

species
1926196
1

 0.00
16

clade
2
1
197563
30301
1632

 1.17

clade
1
1267
197562

 1.17
1631
30299

1
99
subphylum
1541

 1.11
28725
6960

class
0
1
30001
27
11

 0.00

79705
23
4

 0.00
order
0
1

family
0
1
39131
23

 0.00
3

39132
2

 0.00
23
genus
1
0

species
39272

 0.00
1
23

730330

 0.00
6
4
order
1
0

730333

 0.00
5
4
superfamily
1
0

family
1
0
36141

 0.00
4
4

subfamily
0
1
187620
4
3

 0.00

158440
4
2

 0.00
genus
0
1

species
1

 0.00
4
158441

50557
28599

 1.10
1529
class
0
1

28599

 1.10
1528
85512
0
1
clade

7496

 1.10
1527
28599
subclass
1
31

33339
10

 0.00
8
infraclass
0
1


 0.00
6
9
6961
1
0
order

1
0
suborder
5

 0.00
9
50488

9
4

 0.00
70894
0
1
superfamily

9

 0.00
3
70895
0
1
family

79456
9
2

 0.00
genus
0
1

species

 0.00
1
9
197161

order
30073

 0.00
1
1

28558
1518

 1.10
33340
2997
1
infraclass


 0.02
67
485
33341
1
11
cohort

order
1
0
6993
20

 0.00
19

6994

 0.00
7
2
suborder
1
0

1
0
superfamily

 0.00
6
2
1069438

family
1
0
62768

 0.00
5
2

subfamily
1
0
114931

 0.00
4
2

62769
2
3

 0.00
genus
0
1

0
1
no rank
2
2

 0.00
2606691

species
1821528
2
1

 0.00

17

 0.00
12
7001
0
1
suborder


 0.00
11
17
1955150
1
0
infraorder

1
0
no rank
10

 0.00
17
70910

92621
17
9

 0.00
superfamily
0
1

family
1
3
7002

 0.00
8
17

1
0
subfamily
7

 0.00
14
37267

14

 0.00
6
7008
8
1
genus

1

 0.00
1
7009
species

species
0
1
2023354
1
2

 0.00

2023355
1
1

 0.00
subspecies


 0.00
1
3
7010
species

1
1

 0.00
7011
species

5
11

 0.00
6970
0
1
superorder

order
0
1
85823
5

 0.00
10

1
0
superfamily

 0.00
9
5
1049657

8

 0.00
5
1912919
1
0
no rank

family
0
1
46569
2

 0.00
3

62955

 0.00
2
2
subfamily
1
0


 0.00
1
2
46572
genus

46562

 0.00
4
3
family
1
0

subfamily
1
0
105801

 0.00
3
3

60568
3
2

 0.00
genus
0
1

3
1

 0.00
105785
species

7020

 0.01
14
369
order
1
0

1
0
suborder

 0.01
13
369
523712

superfamily
0
1
213545
369

 0.01
12

213546

 0.01
11
369
family
1
0


 0.01
10
369
61471
1
164
genus


 0.00
1
15
61484
species

species
61476
1

 0.00
37

species
170557
18

 0.00
1

species
61474
1

 0.00
11

species
21
1

 0.00
61472

species
11
1

 0.00
629360

61478
16
1

 0.00
species

629358
1

 0.00
49
species

species
27
1

 0.00
170555

order
1
1
50622
21

 0.00
81

50
15

 0.00
70405
0
1
superfamily

15
4

 0.00
143733
0
1
family

1
0
subfamily
3

 0.00
15
143769

0
1
genus
15

 0.00
2
143734

1

 0.00
15
143735
species

62802
18

 0.00
6
family
0
1

1
1
subfamily
5

 0.00
18
143773

0
1
genus
7

 0.00
2
62803

2014036
7

 0.00
1
species

2

 0.00
10
143721
1
0
genus

143722
10
1

 0.00
species

143727

 0.00
4
17
family
1
0

subfamily
0
1
143771
17
3

 0.00

0
1
genus
17
2

 0.00
143731


 0.00
1
17
2065413
species

30

 0.00
5
70404
0
1
superfamily

1
0
family
4

 0.00
30
50623


 0.00
3
30
466866
1
0
subfamily

143767
2

 0.00
30
genus
1
0

552050
30

 0.00
1
species

1
7494
cohort
1355

 0.96
24944
33392

superorder
1
736
85604
599

 0.57
14850

220
13

 0.01
30263
0
1
order

220

 0.01
12
93875
0
1
suborder

1683728
11

 0.01
220
infraorder
1
0

superfamily
0
1
41033
220

 0.01
10

220

 0.01
9
50645
0
1
family

0
1
subfamily
220
8

 0.01
177669

177673
7

 0.01
220
tribe
1
15


 0.00
2
50
1271741
1
0
genus

1271742
50
1

 0.00
species


 0.01
4
155
177674
1
25
genus

1271730
1

 0.00
37
species


 0.00
1
31
1218281
species

692089
1

 0.00
62
species

order
113
1
7088
13894

 0.54
585

0
1
suborder
35

 0.00
4
41024

41025
35
3

 0.00
family
0
1

1
0
genus
2

 0.00
35
41026

species
1

 0.00
35
1042620

41191
580

 0.53
13746
suborder
1
0

0
1
infraorder
13746
579

 0.53
41196

parvorder
1
131
41197

 0.53
578
13746

superfamily
0
1
37584
44
7

 0.00

family
1
0
98958

 0.00
3
20

0
1
genus
20

 0.00
2
101736

species

 0.00
1
20
101737

3

 0.00
24
30222
1
0
family

753374

 0.00
2
24
genus
1
0

species
24

 0.00
1
753375

1
2643
clade
570

 0.52
13571
37567

clade
45
1
104430
668

 0.03
68

31
5

 0.00
104432
0
1
superfamily

186108
31
4

 0.00
family
0
1

subfamily
1
0
1556158

 0.00
3
31

0
1
genus
31
2

 0.00
655084

31

 0.00
1
1101072
species

182

 0.01
15
104434
0
1
superfamily

106496
182
14

 0.01
family
0
1

106499

 0.01
13
182
subfamily
1
3

301641
8

 0.00
98
tribe
1
0

106500
76

 0.00
5
genus
3
1

301036

 0.00
1
19
species

species
23
1

 0.00
1108570

1

 0.00
20
1108569
species

species
1660703
11
1

 0.00

genus
0
1
300855
22
2

 0.00

22
1

 0.00
301037
species

81

 0.00
4
301638
0
1
tribe

genus
1
10
287191
3

 0.00
81

24
1

 0.00
287197
species

species
1

 0.00
47
748215

superfamily
1
0
104435

 0.00
8
60

115354
17

 0.00
4
family
0
1

1
0
subfamily
3

 0.00
17
287187

287110

 0.00
2
17
genus
1
0

1

 0.00
17
287375
species

43

 0.00
3
115353
0
1
family

1
0
genus

 0.00
2
43
287107

287200
43

 0.00
1
species

104437

 0.00
4
20
superfamily
1
0

20
3

 0.00
252293
0
1
family

1
0
genus
2

 0.00
20
252294

species

 0.00
1
20
252295

35

 0.01
330
37568
1
0
superfamily

family
1
13
7139

 0.01
34
330

69
9

 0.00
65022
0
1
subfamily

19
3

 0.00
81689
0
1
tribe

19

 0.00
2
192190
0
1
genus

species

 0.00
1
19
753214

0
1
tribe
50
5

 0.00
173709

572799
50
4

 0.00
genus
0
1

1

 0.00
13
758706
species

20
1

 0.00
1100899
species

758717
1

 0.00
17
species

81687
248

 0.01
24
subfamily
7
1

581387
8

 0.00
86
tribe
1
2

3

 0.00
43
581588
1
2
genus

species
1870148
1

 0.00
19


 0.00
1
22
1101027
species

0
1
genus
16

 0.00
2
293340


 0.00
1
16
192188
species

genus
1
0
82599

 0.00
2
25

species
1100963
25
1

 0.00

7

 0.00
93
581385
1
2
tribe

29054
61
4

 0.00
genus
4
1

species
1594294
1

 0.00
19

989769

 0.00
1
24
species

species
1

 0.00
14
1594293

30

 0.00
2
581513
0
1
genus

species
1594315
30

 0.00
1

tribe
1
0
581389

 0.00
8
62

genus
1
1
572704
39

 0.00
3

1

 0.00
11
1100915
species

species
27

 0.00
1
1100916


 0.00
2
11
572852
1
0
genus

11

 0.00
1
1869985
species

0
1
genus
12

 0.00
2
581658

species
12
1

 0.00
1100989

0
1
superfamily
97
12

 0.00
37582

1
0
family
3

 0.00
13
51653

51654
13

 0.00
2
genus
0
1

species
51655
1

 0.00
13

687156
62

 0.00
4
family
0
1

1
0
genus
3

 0.00
62
687147

1870435
35

 0.00
1
species

1870436
27
1

 0.00
species

33464
22

 0.00
4
family
0
1

subfamily
1
0
397427
3

 0.00
22

0
1
genus
22

 0.00
2
33465

1

 0.00
22
263933
species


 0.01
10
131
41011
1
0
superfamily

family
1
0
41012
9

 0.01
131

1181440
1
4

 0.00
subfamily
0
1

1
3

 0.00
1181405
0
1
genus

2637683
1

 0.00
2
no rank
0
1

species

 0.00
1
1
1181406

4

 0.01
130
236781
1
0
subfamily

41013
130
3

 0.01
genus
0
1

1594354
22
1

 0.00
species

species
108
1

 0.00
1101063

superfamily
1
2
37581
17

 0.01
134

0
1
family
20

 0.00
4
57992

116123
3

 0.00
20
subfamily
1
0

688988

 0.00
2
20
genus
1
0

20

 0.00
1
2870497
species


 0.00
8
52
2681869
1
0
family

1
0
subfamily
4

 0.00
29
116119

1
3
genus

 0.00
3
29
262437

1857958
16
1

 0.00
species

10

 0.00
1
1594222
species

no rank
1
0
2925401

 0.00
3
23

genus
1
0
116120

 0.00
2
23

species
116121
23
1

 0.00

173649
60

 0.00
4
family
0
1

655692
60

 0.00
3
genus
6
1

34
1

 0.00
2561016
species

20
1

 0.00
1869501
species

104431

 0.38
462
9898
clade
1
3998

40092
138
18

 0.01
superfamily
0
1

17

 0.01
138
40093
1
3
family

218718

 0.00
3
20
subfamily
1
0

genus
0
1
218719
20
2

 0.00

20

 0.00
1
218720
species

40096
66
8

 0.00
subfamily
0
1

2839394

 0.00
7
66
tribe
1
0

22

 0.00
2
218743
0
1
genus

species
876063

 0.00
1
22

genus
0
1
218734
20
2

 0.00

species
20

 0.00
1
291688

1
0
genus
2

 0.00
24
218770

species
272628

 0.00
1
24

40100
49
5

 0.00
subfamily
0
1

76212
11

 0.00
2
genus
0
1

species
520884
11

 0.00
1

0
1
genus
38

 0.00
2
40102

species
1

 0.00
38
218760

superfamily
157
1
37572
1511

 0.06
123

199
21

 0.01
27544
1
1
family

subfamily
16
1
42297
183
17

 0.01

3

 0.00
28
91737
1
16
genus

91738

 0.00
1
4
species

species
91739
1

 0.00
8

genus
1
0
42298
2

 0.00
15

species
203782

 0.00
1
15

2

 0.00
34
203780
1
0
genus

species
34

 0.00
1
203781

1
0
genus

 0.00
2
22
242266


 0.00
1
22
242267
species

genus
1
0
1821620
2

 0.00
17

1

 0.00
17
988025
species

265382
24

 0.00
2
genus
0
1

1

 0.00
24
265386
species

genus
8
1
138069
27
3

 0.00

species
268709
1

 0.00
10

species

 0.00
1
9
138070

124406

 0.00
3
15
subfamily
1
0

265359
15
2

 0.00
genus
0
1

species
282391
1

 0.00
15

1
0
family

 0.00
9
46
7143

1
1
subfamily
46

 0.00
8
42289

0
1
tribe
27
3

 0.00
189314

genus
0
1
157396
27
2

 0.00

110791
27
1

 0.00
species

4

 0.00
18
189315
1
0
tribe

18
3

 0.00
7145
2
1
genus

species

 0.00
1
2
66420


 0.00
1
14
76193
species

1
2
family

 0.01
19
213
7114

0
1
subfamily
17
3

 0.00
151208

1
0
genus

 0.00
2
17
189907

189913
17
1

 0.00
species

subfamily
1
0
42450
5

 0.00
27

1
0
genus
2

 0.00
24
42295

species

 0.00
1
24
72248

33411
2

 0.00
3
genus
1
0

3
1

 0.00
33412
species

42449
167
10

 0.01
subfamily
0
1

152601
167
9

 0.01
tribe
3
1

25
2

 0.00
129396
0
1
genus

species
129397
25

 0.00
1

16
2

 0.00
72244
0
1
genus

16

 0.00
1
227532
species

5
1
genus
123

 0.00
4
7115

species

 0.00
1
40
78633


 0.00
1
24
7116
species


 0.00
1
54
64459
species

family
1
70
33415

 0.03
73
896

subfamily
0
1
127218
82
14

 0.00


 0.00
7
80
42315
1
0
tribe

344711
80

 0.00
6
subtribe
0
1

genus
20
1
64444
80

 0.00
5

species
1
0
331299
2

 0.00
39

subspecies
331333
1

 0.00
39

21
2

 0.00
304554
0
1
species

subspecies
1

 0.00
21
331302

30248
2
6

 0.00
tribe
0
1

2

 0.00
5
344705
0
1
subtribe

genus
1
0
13036

 0.00
4
2

subgenus
1
0
151542
3

 0.00
2

13037
2
2

 0.00
species
0
1

subspecies
278856
2

 0.00
1


 0.01
17
282
40040
1
4
subfamily

10

 0.01
193
171576
1
3
tribe

76218
92
6

 0.00
genus
1
1

44

 0.00
2
442324
0
1
subgenus

1

 0.00
44
171594
species

111880

 0.00
3
47
subgenus
1
2

species
111881
1

 0.00
22

species
171585

 0.00
1
23

1
1
genus
3

 0.00
98
42274

species
54
1

 0.00
42275

1

 0.00
43
171605
species

171578

 0.00
6
85
tribe
1
0

2
1
subtribe
85

 0.00
5
171580

genus
0
1
596672
28
2

 0.00

28
1

 0.00
113330
species

2

 0.00
55
104514
1
0
genus

species

 0.00
1
55
113334

40037
155

 0.01
11
subfamily
1
1

1
0
tribe

 0.00
3
31
127322

genus
1
0
33416

 0.00
2
31

species
33443
1

 0.00
31

7
1
tribe
123
7

 0.00
127312

genus
1
0
405031
2

 0.00
51

species
405034

 0.00
1
51

1
0
genus
2

 0.00
25
127313

25
1

 0.00
191398
species


 0.00
2
40
525812
1
0
genus


 0.00
1
40
405009
species

279

 0.01
26
42282
2
1
subfamily

tribe
1
0
1664845

 0.00
4
48

genus
5
1
111897
48
3

 0.00

species
447833
1

 0.00
24

species

 0.00
1
19
111903

127320
229

 0.01
21
tribe
3
1


 0.00
6
64
150883
1
0
subtribe

genus
1
0
111919
5

 0.00
64

13
2

 0.00
111885
0
1
subgenus

species
2795564
13
1

 0.00

111950
51
2

 0.00
no rank
0
1


 0.00
1
51
191418
species

0
1
subtribe
37
3

 0.00
167180

1
0
genus

 0.00
2
37
110367

37
1

 0.00
110368
species

3

 0.00
23
150886
1
0
subtribe

genus
0
1
111908
23
2

 0.00

species

 0.00
1
23
111912

0
1
subtribe
32
3

 0.00
150884

0
1
genus
32
2

 0.00
111922

species
32

 0.00
1
111923

1
0
subtribe

 0.00
5
70
366209

genus
1
0
111915
2

 0.00
21

species
1

 0.00
21
111917

49
2

 0.00
111932
0
1
genus

116150

 0.00
1
49
species

28
4

 0.00
100750
0
1
subfamily

1
0
tribe

 0.00
3
28
215788

124410

 0.00
2
28
genus
1
0

species
270466
1

 0.00
28

119

 0.00
19
37569
0
1
superfamily

4

 0.00
36
7089
1
0
family

36

 0.00
3
475327
0
1
subfamily

36
2

 0.00
7090
2
1
genus

species

 0.00
1
34
7091

family
1
1
7128
14

 0.00
83

subfamily
0
1
469321
50

 0.00
6

523180
50

 0.00
5
tribe
0
1

522835

 0.00
2
28
genus
1
0

species
28
1

 0.00
522836

genus
0
1
522847
22
2

 0.00

1

 0.00
22
522848
species

subfamily
1
0
82617
7

 0.00
32

18
3

 0.00
523176
0
1
tribe

283833
18
2

 0.00
genus
0
1

644661
18
1

 0.00
species

523174
14

 0.00
3
tribe
0
1

genus
0
1
82622
14

 0.00
2

14

 0.00
1
987953
species

0
1
superfamily
69
12

 0.00
104423

0
1
family
69

 0.00
11
104425

29

 0.00
5
219490
0
1
subfamily

2

 0.00
18
219491
1
0
genus

species
721137
1

 0.00
18

11
2

 0.00
721162
0
1
genus

species
721163
11
1

 0.00

104465

 0.00
5
40
subfamily
1
0

721164

 0.00
2
14
genus
1
0

721165
14
1

 0.00
species

104426
26

 0.00
2
genus
0
1

species

 0.00
1
26
104428

1
422
superfamily

 0.10
176
2703
37570

family
1
1
319783
61
7

 0.00

753688
3

 0.00
33
subfamily
1
0

1
0
genus
2

 0.00
33
572722

988004

 0.00
1
33
species

27

 0.00
3
95245
0
1
subfamily

1
0
genus
2

 0.00
27
56393

species
27
1

 0.00
987977

37571
20

 0.01
132
family
1
3

subfamily
1
2
319765

 0.00
7
50

214308
3

 0.00
22
genus
1
2

988019

 0.00
1
16
species

species
988018
1

 0.00
4

1
1
genus
26
3

 0.00
214280

species
11
1

 0.00
753204

species
14
1

 0.00
988002

319762
15

 0.00
3
subfamily
0
1

214089
15

 0.00
2
genus
0
1

15
1

 0.00
987902
species

subfamily
0
1
319766
25
3

 0.00

genus
0
1
56587
25
2

 0.00

species
987943
25

 0.00
1

319770
10

 0.00
3
subfamily
0
1

0
1
genus
10

 0.00
2
987448

species
987449
10

 0.00
1

319773
29
3

 0.00
subfamily
0
1


 0.00
2
29
13633
1
0
genus

753216

 0.00
1
29
species

family
1
30
695564
39

 0.02
552

86
7

 0.00
27548
2
1
subfamily

genus
1
0
13122
2

 0.00
42

species

 0.00
1
42
78897

24

 0.00
2
319798
0
1
genus


 0.00
1
24
987935
species

33413
2

 0.00
18
genus
1
0

species
335469

 0.00
1
18

77
5

 0.00
95217
0
1
subfamily

genus
1
0
95218
2

 0.00
41

753189
41

 0.00
1
species

36
2

 0.00
411962
0
1
genus

411963
36
1

 0.00
species

50
3

 0.00
95222
0
1
subfamily

0
1
genus
50
2

 0.00
705937

species
50
1

 0.00
987440

16

 0.01
209
30225
1
4
subfamily

tribe
9
1
132199
114
8

 0.00

464722
35
3

 0.00
genus
1
1

987424
1

 0.00
21
species

species
13

 0.00
1
987419

genus
1
0
694848

 0.00
2
37

species
987918
37

 0.00
1

0
1
genus
33

 0.00
2
694657

987980

 0.00
1
33
species

tribe
7
1
1945703
91
7

 0.00

214310
29
2

 0.00
genus
0
1

214311
29
1

 0.00
species

genus
1
0
695183
2

 0.00
29

species
875881
1

 0.00
29

genus
0
1
214365
26

 0.00
2

species

 0.00
1
26
875880

1
1
subfamily
7

 0.00
100
1583079

genus
0
1
292570
34
2

 0.00

species

 0.00
1
34
938167

0
1
genus
22
2

 0.00
938237


 0.00
1
22
938238
species

56375
2

 0.00
43
genus
1
0

423510
43

 0.00
1
species

family
1
297
7100

 0.06
109
1536

95179
28

 0.01
300
subfamily
1
10

320076

 0.00
2
16
genus
1
0

species
987895
16
1

 0.00

15

 0.01
186
2555566
1
9
tribe

214276
47
4

 0.00
genus
5
1

species
1

 0.00
12
214277

species
987995
21
1

 0.00

species

 0.00
1
9
753202

0
1
genus
20
2

 0.00
47766

1857961

 0.00
1
20
species

2

 0.00
14
320033
1
0
genus

14
1

 0.00
987925
species

320016

 0.00
4
77
genus
1
1

species
997545
26

 0.00
1

species

 0.00
1
25
988049

1

 0.00
25
987431
species


 0.00
2
19
214282
1
0
genus

19

 0.00
1
320037
species

tribe
8
1
2555556
88

 0.00
10

0
1
genus
21
3

 0.00
95189

species

 0.00
1
10
875885

11
1

 0.00
689061
species

0
1
genus
23
2

 0.00
688395

1

 0.00
23
987876
species

1
0
genus
2

 0.00
20
882791

species
988174

 0.00
1
20

16
2

 0.00
214170
0
1
genus

species
214171
16
1

 0.00


 0.01
13
161
95182
1
3
subfamily

56365
53
3

 0.00
genus
1
1

1

 0.00
20
689058
species

species
32
1

 0.00
987877

genus
1
1
7106
5

 0.00
45

species
7109
11

 0.00
1

1

 0.00
30
7107
species

species
1

 0.00
2
7108

69820

 0.00
1
1
species

1
0
genus
2

 0.00
25
988055

species
25

 0.00
1
988056

0
1
genus
35

 0.00
2
1430964

species
1870430

 0.00
1
35

subfamily
8
1
572922
202
18

 0.01

2492374
22

 0.00
2
genus
0
1

2492375
22

 0.00
1
species

0
1
genus
37
2

 0.00
988063

1337163
37
1

 0.00
species

997550
15
2

 0.00
genus
0
1

species
997551

 0.00
1
15

20
2

 0.00
988080
0
1
genus

20
1

 0.00
988081
species

genus
0
1
988070
15

 0.00
2

15
1

 0.00
988071
species

0
1
genus
17

 0.00
2
1101105

species
1101106
17

 0.00
1

0
1
genus
43
3

 0.00
689302

species
987872

 0.00
1
20

species
987866
1

 0.00
23

genus
0
1
988105
25

 0.00
2

species
988106
1

 0.00
25

0
1
subfamily
12

 0.00
3
95178

7112

 0.00
2
12
genus
1
0

species
7113

 0.00
1
12

95244
260

 0.01
17
subfamily
14
1

320089
23

 0.00
2
genus
0
1

species
875884
23

 0.00
1

genus
0
1
55056
23

 0.00
2

species
55057
23
1

 0.00

1
0
genus

 0.00
2
44
946290


 0.00
1
44
988041
species

genus
8
1
103830
78
4

 0.00

24
1

 0.00
987983
species


 0.00
1
26
987985
species


 0.00
1
20
997540
species

1
0
genus

 0.00
2
24
988059

1

 0.00
24
988060
species

genus
0
1
988123
17

 0.00
2

species
988125

 0.00
1
17

genus
0
1
320087
37
2

 0.00

species
987933

 0.00
1
37

subfamily
1
0
1430885

 0.00
3
22

2

 0.00
22
708062
1
0
genus

species
708063
22
1

 0.00

95186
11

 0.01
130
subfamily
1
2

254364
3

 0.00
34
genus
1
3

254365
21

 0.00
1
species

10
1

 0.00
938171
species

3

 0.00
46
254361
1
2
genus

species
1

 0.00
21
254363

species
987893
23
1

 0.00

genus
0
1
254717
13

 0.00
2

species
13

 0.00
1
689277

35

 0.00
2
179673
0
1
genus

35

 0.00
1
179674
species

95214
3

 0.00
35
subfamily
1
0

1
0
genus
2

 0.00
35
753440

species
35

 0.00
1
753441

116124
6

 0.00
53
subfamily
1
0

genus
1
2
116125
3

 0.00
39

23
1

 0.00
987909
species

species
116126
14
1

 0.00

0
1
genus
14
2

 0.00
116129

species
1

 0.00
14
116130

subfamily
1
0
95175
6

 0.00
64

3

 0.00
45
56362
1
0
genus

species
987865
22

 0.00
1

species

 0.00
1
23
987859

genus
0
1
938225
19

 0.00
2

938226

 0.00
1
19
species

1
0
superfamily
71

 0.03
908
82592

82593

 0.03
70
908
family
1
56

subfamily
1
0
393382
3

 0.00
32


 0.00
2
32
104485
1
0
genus

species
32
1

 0.00
104486

17

 0.00
3
104442
0
1
subfamily

0
1
genus
17
2

 0.00
104446

species
17
1

 0.00
104447

82596
461
29

 0.02
subfamily
17
1

2

 0.00
14
104473
1
0
genus

species
104474
14
1

 0.00

genus
1
0
82597

 0.00
2
28

species
934813
1

 0.00
28

104475
3

 0.00
48
genus
1
2


 0.00
1
22
104476
species

24

 0.00
1
190331
species

genus
1
0
214128

 0.00
3
38


 0.00
1
20
875883
species

18
1

 0.00
722662
species

genus
1
0
393392
2

 0.00
18

species
688445
1

 0.00
18

0
1
genus
31

 0.00
2
190355

31

 0.00
1
190356
species

genus
0
1
692034
91

 0.00
2

species
934875
91

 0.00
1

1
0
genus
2

 0.00
9
722672

722673
9

 0.00
1
species

1
0
genus

 0.00
2
37
934914

species
1

 0.00
37
934915

64

 0.00
2
572919
0
1
genus

64
1

 0.00
934894
species

21

 0.00
2
82594
0
1
genus

82595

 0.00
1
21
species

genus
1
0
704699

 0.00
2
12

species
934829

 0.00
1
12

33
2

 0.00
704816
0
1
genus

species
33
1

 0.00
934882

104461
12
3

 0.00
subfamily
0
1

1
0
genus

 0.00
2
12
934941

934942
12

 0.00
1
species


 0.01
31
330
104450
1
22
subfamily

190368
14
2

 0.00
genus
0
1

species
14

 0.00
1
934904

24

 0.00
2
934939
0
1
genus

species
934940
24

 0.00
1

genus
1
0
326956

 0.00
2
5

5

 0.00
1
987013
species

1
0
genus

 0.00
2
28
934916


 0.00
1
28
934917
species

0
1
genus
22
2

 0.00
214392

species
934828
22
1

 0.00

genus
1
7
214137

 0.00
6
92

17

 0.00
1
934866
species

934847

 0.00
1
22
species

species
15
1

 0.00
934840

1

 0.00
26
934844
species

934845
5

 0.00
1
species

0
1
genus
13

 0.00
2
214189

species
13
1

 0.00
934876

934935

 0.00
2
16
genus
1
0

16
1

 0.00
934936
species

genus
1
0
104451
2

 0.00
25

104452
1

 0.00
25
species

1
0
genus
2

 0.00
11
104456

104457
11

 0.00
1
species

0
1
genus
14

 0.00
2
214132

species
934839
14

 0.00
1

1
0
genus
2

 0.00
16
104458

16

 0.00
1
104460
species

2

 0.00
28
873511
1
0
genus

934888

 0.00
1
28
species

37573
33

 0.01
380
superfamily
1
10

268499
18

 0.01
262
family
1
8

190
12

 0.01
40081
8
1
subfamily

1
1
genus
3

 0.00
57
572808

species
22
1

 0.00
1660579

1594226
34
1

 0.00
species

genus
0
1
572825
16

 0.00
2

1594250
16
1

 0.00
species

genus
1
0
168630

 0.00
2
40

40

 0.00
1
168631
species

1368975
31
2

 0.00
genus
0
1

species
1371681

 0.00
1
31

40084

 0.00
2
38
genus
1
0

species
1

 0.00
38
40085

1
0
subfamily

 0.00
5
64
299362

687116
21
2

 0.00
genus
0
1

species
1

 0.00
21
1594321

genus
0
1
1666817
43
2

 0.00

species
1666818
1

 0.00
43

family
1
0
7135

 0.00
14
108

subfamily
2
1
40083
41
5

 0.00

2

 0.00
9
989881
1
0
genus

9
1

 0.00
1666458
species

687068
30

 0.00
2
genus
0
1

species

 0.00
1
30
1857951

1
0
subfamily

 0.00
5
66
299347

1101094

 0.00
2
13
genus
1
0

species
13
1

 0.00
1101095

genus
1
0
1101109

 0.00
2
53

species
1101110
1

 0.00
53

3

 0.00
1
40082
1
0
subfamily

7136
1
2

 0.00
genus
0
1

species
1

 0.00
1
7137

0
1
superfamily
72
9

 0.00
104493

0
1
family
72

 0.00
8
186111

3
1
subfamily
72
7

 0.00
186112

1
0
genus

 0.00
2
17
467773

17
1

 0.00
467774
species

genus
1
0
1594452
2

 0.00
21

species
1

 0.00
21
1594453


 0.00
2
31
753424
1
0
genus

1660692
1

 0.00
31
species

7041
729
146

 0.03
order
37
1


 0.02
125
527
41084
1
24
suborder

infraorder
0
1
41087
55
23

 0.00

71192

 0.00
5
1
superfamily
1
0

0
1
family
1

 0.00
4
50527

3

 0.00
1
261156
1
0
subfamily

0
1
genus
1
2

 0.00
195164

species
224129
1

 0.00
1

54

 0.00
17
71193
0
1
superfamily

0
1
family
2

 0.00
4
7049

1
0
subfamily
3

 0.00
2
433514

7053

 0.00
2
2
genus
1
0

7054

 0.00
1
2
species

1
0
family

 0.00
4
4
30009

3

 0.00
4
116139
1
0
subfamily

4

 0.00
2
292457
0
1
genus

4
1

 0.00
292458
species

41097
8

 0.00
48
family
1
0

48

 0.00
7
433502
1
1
subfamily

25

 0.00
2
41098
0
1
genus


 0.00
1
25
195172
species

12

 0.00
2
186072
0
1
genus

species

 0.00
1
12
1553677

2

 0.00
10
41100
1
0
genus

species
1

 0.00
10
41101

11
1
infraorder
345

 0.01
80
41088

71527
24

 0.00
9
superfamily
0
1


 0.00
5
10
7065
1
1
family

1304792
9

 0.00
4
no rank
0
1

3

 0.00
9
7069
1
0
genus

species
1
1

 0.00
7070

1

 0.00
8
7072
species

3

 0.00
14
55098
1
0
family

295984

 0.00
2
14
genus
1
0

1

 0.00
14
346838
species

superfamily
1
0
2939015
13

 0.00
57

0
1
family
57

 0.00
12
7080

7081
11

 0.00
57
subfamily
1
0

tribe
1
1
263631

 0.00
7
48

10
2

 0.00
41138
0
1
genus

species
1

 0.00
10
41139

genus
0
1
115356
22

 0.00
2


 0.00
1
22
115357
species

genus
0
1
7083
15
2

 0.00

species
1

 0.00
15
7084

263632
9

 0.00
3
tribe
0
1

genus
0
1
347358
9
2

 0.00

species
9
1

 0.00
347359

60
19

 0.00
71529
0
1
superfamily

family
1
0
7042

 0.00
14
33

1
0
subfamily
3

 0.00
15
123516

genus
1
0
201855
2

 0.00
15

species
15
1

 0.00
467358

55867
2

 0.00
3
subfamily
0
1

0
1
genus
2

 0.00
2
77156

species
77166
1

 0.00
2

subfamily
0
1
39812
3

 0.00
3

0
1
genus
3

 0.00
2
7045

species
3

 0.00
1
7048

39814
4

 0.00
13
subfamily
1
0

tribe
0
1
465383
13
3

 0.00

genus
0
1
122852
13

 0.00
2

1

 0.00
13
202137
species

0
1
family
27
4

 0.00
122737

27

 0.00
3
701798
0
1
subfamily

2

 0.00
27
122772
1
0
genus

species
201766
27

 0.00
1

71528

 0.01
25
138
superfamily
1
0

family
1
1
27439
138
24

 0.01

6

 0.00
69
63707
1
0
subfamily

63708
69

 0.00
5
tribe
0
1

80248
23

 0.00
2
genus
0
1

23
1

 0.00
80249
species

0
1
genus
46
2

 0.00
41125

species
1

 0.00
46
1587174

42
14

 0.00
63710
0
1
subfamily

tribe
1
0
131578

 0.00
6
32


 0.00
3
8
224132
1
0
genus

species
5

 0.00
1
224133

species
3
1

 0.00
444603

genus
0
1
294691
24
2

 0.00

species

 0.00
1
24
2598218

63711
7

 0.00
10
tribe
1
0

subtribe
1
0
226742
6

 0.00
10

226749
10

 0.00
5
no rank
0
1

10

 0.00
4
50385
0
1
genus

species
1
0
50389
2

 0.00
1

subspecies
50390

 0.00
1
1

species
1

 0.00
9
107213


 0.00
3
26
131688
1
0
subfamily

204943
2

 0.00
26
genus
1
0

204949
1

 0.00
26
species

superfamily
0
1
71526
29
8

 0.00

0
1
family
29

 0.00
7
116151

4
3

 0.00
577242
0
1
subfamily

genus
0
1
116152
4

 0.00
2

116153
4
1

 0.00
species

577241

 0.00
3
25
subfamily
1
0

1431902

 0.00
2
25
genus
1
0

1431903
1

 0.00
25
species

71525
5

 0.00
26
superfamily
1
0

26

 0.00
4
186093
0
1
family

353826
26
3

 0.00
subfamily
0
1

295699
26

 0.00
2
genus
0
1

species
1

 0.00
26
295700

41086
3
7

 0.00
infraorder
0
1

75546
3

 0.00
6
superfamily
0
1

3
5

 0.00
7055
0
1
family

0
1
subfamily
3
4

 0.00
41142

3
3

 0.00
569643
0
1
no rank


 0.00
2
3
166331
1
0
genus

species
3

 0.00
1
166361

infraorder
0
1
41085
100

 0.00
14


 0.00
13
100
75543
1
2
superfamily

8

 0.00
53
29026
1
0
family

351514
7

 0.00
53
no rank
1
0

82886
53

 0.00
6
subfamily
0
1

tribe
1
1
295648

 0.00
5
53

219450
17
2

 0.00
genus
0
1

346820
17

 0.00
1
species

2

 0.00
35
290671
1
0
genus

35
1

 0.00
662956
species

57514
45
4

 0.00
family
0
1

1
0
subfamily
3

 0.00
45
82881

414933
2

 0.00
45
genus
1
0

414934
1

 0.00
45
species

suborder
0
1
41071
165
20

 0.01

superfamily
0
1
535382
165
19

 0.01

3
1
family
165

 0.01
18
41073

subfamily
1
0
71539

 0.00
9
70

1
0
tribe
8

 0.00
70
60833


 0.00
2
24
60836
1
0
genus

species
24

 0.00
1
878056

genus
5
1
60834
46

 0.00
5

33

 0.00
2
186524
0
1
subgenus

1

 0.00
33
878211
species

subgenus
0
1
186517
8
2

 0.00

species
8
1

 0.00
110024

subfamily
11
1
71541
92
8

 0.00

879230

 0.00
4
30
tribe
1
0

genus
0
1
41078
30
3

 0.00

1
0
subgenus

 0.00
2
30
484232

30
1

 0.00
767470
species

tribe
0
1
60759
51
3

 0.00

1
0
genus

 0.00
2
51
247414

51
1

 0.00
247415
species

order
1
52
7147
314

 0.03
906

7148
272
96

 0.01
suborder
4
1

43784
38
10

 0.00
infraorder
0
1

43790

 0.00
5
37
superfamily
1
0

0
1
family
37

 0.00
4
52729

subfamily
0
1
52730
37

 0.00
3

189978
37

 0.00
2
genus
0
1

species
189979

 0.00
1
37

1
0
superfamily

 0.00
4
1
41830

1
0
family
3

 0.00
1
7184

35571
2

 0.00
1
genus
1
0

38358
1

 0.00
1
species

1
6

 0.00
43789
0
1
infraorder

1
5

 0.00
41829
0
1
superfamily

1
0
family
4

 0.00
1
41042

52735
1

 0.00
3
subfamily
0
1

46210
2

 0.00
1
genus
1
0

species
2719080
1
1

 0.00

43787
8
9

 0.00
infraorder
0
1

8

 0.00
8
41831
0
1
superfamily

7

 0.00
8
7197
1
0
family

1
0
subfamily

 0.00
6
8
7198

genus
0
1
7199
7
3

 0.00

0
1
subgenus
7

 0.00
2
252607

species
7

 0.00
1
7200

252611
1

 0.00
2
genus
0
1

268290
1

 0.00
1
species

221
70

 0.01
43786
0
1
infraorder

1
0
superfamily
56

 0.01
166
41827

2
1
family
166

 0.01
55
7157

subfamily
0
1
43817
39

 0.00
11

1
0
tribe
5

 0.00
24
1056966

0
1
genus
24

 0.00
4
7158

1
1
subgenus
24
3

 0.00
53541

7160
19

 0.00
1
species

7159
4

 0.00
1
species

15
5

 0.00
53550
0
1
tribe

genus
0
1
7174
15
4

 0.00

0
1
subgenus
15
3

 0.00
53527

9
1
no rank
15

 0.00
2
518105

7176

 0.00
1
6
species

0
1
subfamily
125
43

 0.00
43816

1
6
genus
42

 0.00
125
7164

44534

 0.00
18
44
subgenus
1
2


 0.00
3
12
44535
1
0
clade

1496333
5

 0.00
1
species

species

 0.00
1
7
30069

0
1
clade
11
5

 0.00
44537

1
3
no rank
4

 0.00
11
44542

1518534
2
1

 0.00
species

1

 0.00
5
7165
species

30066
1

 0.00
1
species

15
5

 0.00
59140
2
1
clade

59142
4
2

 0.00
species group
0
1

62324
4

 0.00
1
species


 0.00
1
7
1521116
species

186751
1

 0.00
2
species

4
4

 0.00
44536
0
1
clade

3

 0.00
4
185573
1
0
species group

1
0
no rank
2

 0.00
4
185574

species

 0.00
1
4
185578

1
0
subgenus
3

 0.00
5
68877

species

 0.00
1
4
139047

68878
1
1

 0.00
species

subgenus
1
0
44482

 0.00
8
19

section
0
1
58247
19

 0.00
7

1
0
series
6

 0.00
19
58250

1
0
species group
2

 0.00
1
59131

species
74873

 0.00
1
1

1
6
species group
3

 0.00
18
59130

species
345580
6
1

 0.00

139045

 0.00
1
6
species

12

 0.00
51
44543
1
1
subgenus

clade
0
1
44545
34
4

 0.00

0
1
clade
34
3

 0.00
44546

species group
1
0
44552

 0.00
2
34

43151
34
1

 0.00
species

0
1
clade
16

 0.00
7
44544

1
0
clade
2

 0.00
3
44547

7167
3
1

 0.00
species

1
0
clade

 0.00
4
13
44548


 0.00
3
13
44549
1
0
species group

0
1
species subgroup
13

 0.00
2
44550

species
42839

 0.00
1
13

13

 0.00
55
41828
1
0
superfamily

41819
33
6

 0.00
family
0
1

1
0
subfamily

 0.00
5
33
43801

0
1
tribe
33

 0.00
4
58262

0
1
genus
33
3

 0.00
41820

0
1
subgenus
33
2

 0.00
58277

179676
33
1

 0.00
species

6

 0.00
22
7149
1
0
family

1
0
subfamily
5

 0.00
22
54970

tribe
1
0
72530
4

 0.00
22

genus
0
1
7150
22
3

 0.00

no rank
1
0
1165752
2

 0.00
22

species
315576
22

 0.00
1

suborder
0
1
7203
582
217

 0.02

578
211

 0.02
43733
14
1
infraorder

200

 0.02
504
480118
1
0
clade

480117
199

 0.02
504
clade
1
16

no rank
1
0
43737
51

 0.01
176

1
2
superfamily

 0.01
50
176
43740

family
1
3
34680

 0.01
45
161

0
1
subfamily
63

 0.00
19
43838

115274
51

 0.00
13
tribe
3
1

414873
3

 0.00
2
genus
0
1

species
414876
3

 0.00
1

0
1
genus
9
2

 0.00
414800

species
9
1

 0.00
414801

219538

 0.00
2
19
genus
1
0


 0.00
1
19
219539
species

genus
0
1
414810
8
2

 0.00

species
1352479
8
1

 0.00

genus
1
0
286458
2

 0.00
4

species
286459

 0.00
1
4


 0.00
2
5
323311
1
0
genus

1

 0.00
5
323312
species

1
0
tribe
5

 0.00
12
224219

1
0
genus
2

 0.00
3
92597

species
653684
1

 0.00
3

genus
0
1
192444
9
2

 0.00

species

 0.00
1
9
414846

115244
25

 0.00
95
subfamily
1
0

192448
28
5

 0.00
tribe
0
1

173981
28

 0.00
4
genus
1
1

species
273407
5

 0.00
1

273409
8

 0.00
1
species

1

 0.00
14
173985
species

115284
1
3

 0.00
tribe
0
1

226148

 0.00
2
1
genus
1
0

species
226149
1

 0.00
1

9

 0.00
41
115277
1
0
tribe

0
1
genus
2
2

 0.00
2714348

species
2714349
1

 0.00
2


 0.00
2
18
115278
1
0
genus

18
1

 0.00
2725509
species

198633

 0.00
4
21
genus
1
0

species
198635
7
1

 0.00

species
1124515

 0.00
1
9

species
1572519
1

 0.00
5

tribe
0
1
224230
25

 0.00
7

9
2

 0.00
34681
0
1
genus

9
1

 0.00
34682
species

10

 0.00
2
226146
0
1
genus

226147

 0.00
1
10
species

0
1
genus
6

 0.00
2
224240

species
1

 0.00
6
374264

0
1
family
13

 0.00
4
43835

13
3

 0.00
115302
0
1
subfamily

genus
1
0
115303

 0.00
2
13

566305
13
1

 0.00
species

no rank
1
4
43738

 0.01
147
312

no rank
1
6
43741

 0.01
95
224

superfamily
1
0
43752
19

 0.00
21


 0.00
18
21
7211
1
0
family

1
0
subfamily
12

 0.00
14
164860

43871
14
11

 0.00
tribe
0
1

genus
1
0
47833
3

 0.00
4

0
1
subgenus
4

 0.00
2
1987911

species
28588
4
1

 0.00

27456
10
7

 0.00
genus
0
1

1
0
subgenus

 0.00
4
6
47832

174628

 0.00
1
4
species

no rank
1
1
98808
2
2

 0.00

species
98809
1
1

 0.00

1
0
subgenus

 0.00
2
4
69624

species
104688
4
1

 0.00

7
5

 0.00
43867
0
1
subfamily

1
0
tribe

 0.00
4
7
43901

1
0
subtribe
3

 0.00
7
164882

28609

 0.00
2
7
genus
1
1

species
6
1

 0.00
28612

43745
2

 0.00
4
superfamily
0
1

family
0
1
139644
2
3

 0.00

2

 0.00
2
139679
1
0
genus

species
139649
1

 0.00
2

56

 0.01
150
43746
1
0
superfamily

7214
55

 0.01
150
family
1
0

43845
150
54

 0.01
subfamily
0
1


 0.00
4
2
1861795
1
0
tribe

1
0
genus
3

 0.00
2
7354

species group
0
1
32386
2

 0.00
2

7225
2
1

 0.00
species


 0.01
49
148
46877
1
0
tribe

7215

 0.01
48
148
genus
1
17

7
2

 0.00
32280
0
1
subgenus

30019
1

 0.00
7
species

504493
51

 0.00
6
no rank
0
1

1
0
clade
5

 0.00
51
48384

4

 0.00
51
48301
1
0
clade

0
1
species group
51
3

 0.00
48302

32378

 0.00
2
51
species subgroup
1
0

1

 0.00
51
7222
species

32341
63
27

 0.00
subgenus
3
1

1
1
species group
32

 0.00
16
32346

2
1
species subgroup
4

 0.00
2
32353

2

 0.00
1
1486046
species

32348

 0.00
2
1
species subgroup
1
0

1

 0.00
1
30023
species

32351
15
3

 0.00
species subgroup
4
1

species
10

 0.00
1
7227

species

 0.00
1
1
7220

3

 0.00
4
32352
0
1
species subgroup

no rank
1
0
446045

 0.00
2
1

species
7274

 0.00
1
1

30033
2

 0.00
1
species

0
1
species subgroup
2
2

 0.00
32350

species
30025
2

 0.00
1

0
1
species subgroup
6
2

 0.00
65962

1041015

 0.00
1
6
species

0
1
species group
3
3

 0.00
32365

2

 0.00
3
32367
1
0
species subgroup


 0.00
1
3
7260
species

species group
1
0
32355

 0.00
7
25

3

 0.00
7
32357
1
0
species subgroup

7241
3

 0.00
1
species

4

 0.00
1
7266
species

0
1
species subgroup
18

 0.00
3
32358

14
1

 0.00
7229
species

4
1

 0.00
7237
species

0
1
subgenus
10
12

 0.00
32281

0
1
species group
2

 0.00
4
32321


 0.00
3
2
32324
1
0
species subgroup

1
0
no rank

 0.00
2
2
198037

7230

 0.00
1
2
species

32304
3

 0.00
3
species group
1
0

0
1
species subgroup
3
2

 0.00
32307

3
1

 0.00
7291
species

1
1
species group

 0.00
2
2
32335

1

 0.00
1
7244
species

32320
3

 0.00
2
species group
0
1

3
1

 0.00
198719
species

2

 0.00
6
43750
0
1
superfamily

169447
5

 0.00
2
family
1
0

1096076
1
2

 0.00
genus
0
1


 0.00
1
1
1096077
species

1
2

 0.00
1226614
0
1
genus

1
1

 0.00
1226616
species


 0.00
9
43
43744
1
0
superfamily

115263
8

 0.00
43
family
1
0

1
0
subfamily

 0.00
7
43
115265

2

 0.00
10
305546
1
0
genus

species
1219171
1

 0.00
10

286486

 0.00
2
21
genus
1
0

species
2829445
21

 0.00
1


 0.00
2
12
1219203
1
0
genus

species
1

 0.00
12
1219204

43742
84
51

 0.00
no rank
0
1

4

 0.00
5
43753
0
1
superfamily

0
1
family
4
4

 0.00
7392

7393

 0.00
3
4
genus
1
0

0
1
subgenus
4
2

 0.00
44051


 0.00
1
4
7396
species

1
0
superfamily
7

 0.00
4
43754


 0.00
6
4
7366
1
0
family

5

 0.00
4
43910
1
0
subfamily


 0.00
4
4
57894
1
0
tribe


 0.00
3
4
7369
1
0
genus

44052
2

 0.00
4
subgenus
1
0

species
7370
1

 0.00
4

43755
76

 0.00
38
superfamily
3
1


 0.00
3
1
43758
1
0
family

genus
1
0
1262320
2

 0.00
1

1262321
1
1

 0.00
species


 0.00
8
6
7371
1
0
family

1
0
subfamily

 0.00
3
1
43912

genus
0
1
7372
1
2

 0.00

species
27454
1

 0.00
1

5

 0.00
4
43914
0
1
subfamily

0
1
genus
5
3

 0.00
7374

species
1
1

 0.00
7375

13632
1

 0.00
4
species

34
9

 0.00
7381
0
1
family

1
0
subfamily
8

 0.00
34
43916

7384
7

 0.00
34
genus
1
4

2

 0.00
2
226134
0
1
subgenus


 0.00
1
2
236850
species


 0.00
2
6
321190
1
0
subgenus

596942
1

 0.00
6
species

1
0
subgenus
2

 0.00
22
236878

22
1

 0.00
1206372
species

1
0
family

 0.00
4
5
54279

genus
1
0
229639

 0.00
3
5

species

 0.00
1
3
1266490

1

 0.00
2
670595
species

27474

 0.00
13
27
family
1
0

subfamily
0
1
54286
1

 0.00
4

3

 0.00
1
569105
1
0
tribe

569045
1

 0.00
2
genus
0
1

species
1

 0.00
1
569046

subfamily
0
1
43917
25

 0.00
4

1
0
tribe

 0.00
3
25
179426

genus
1
0
569039

 0.00
2
25

species
1

 0.00
25
569040

1

 0.00
4
54288
0
1
subfamily

1
3

 0.00
141256
0
1
tribe

1

 0.00
2
141257
0
1
genus

species
631329
1

 0.00
1

1
0
superfamily

 0.00
10
60
50671

family
0
1
50674
43
5

 0.00

1
0
subfamily

 0.00
4
43
50694

4
1
genus
43
3

 0.00
219362

species
240869

 0.00
1
16

23

 0.00
1
2741128
species

50673
17
4

 0.00
family
0
1

subfamily
0
1
50679
17
3

 0.00

2

 0.00
17
247604
1
0
genus

2794001

 0.00
1
17
species

43734

 0.00
5
4
infraorder
1
0

4

 0.00
4
34687
1
0
family

343564
4
3

 0.00
subfamily
0
1

4

 0.00
2
343581
0
1
genus

343691
4

 0.00
1
species

0
1
superorder
28
7

 0.00
85817


 0.00
6
28
7516
1
0
order

2029065

 0.00
5
28
suborder
1
0

family
0
1
7520
28
4

 0.00

3

 0.00
28
2029106
1
0
subfamily

7521
28
2

 0.00
genus
0
1

189513
28

 0.00
1
species

936
281

 0.04
7399
13
1
order

1
0
superfamily

 0.00
4
3
222831

27528

 0.00
3
3
family
1
0

27529

 0.00
2
3
genus
1
0

3

 0.00
1
222816
species

1
1
superfamily

 0.00
15
100
85772

0
1
family
92

 0.00
7
27532

subfamily
0
1
112287
92
6

 0.00

1250642
2

 0.00
12
genus
1
0

species
12
1

 0.00
1385029

112291
3

 0.00
80
genus
1
0

species
222778
53

 0.00
1

27

 0.00
1
362091
species

1
0
family
3

 0.00
3
2982298

1
0
genus

 0.00
2
3
37343

37344
1

 0.00
3
species

52632

 0.00
4
4
family
1
0

3

 0.00
4
410274
1
1
subfamily

270857
2

 0.00
3
genus
1
2

2872261
1

 0.00
1
species

20
1
suborder
819

 0.03
257
7400

689
192

 0.03
7434
39
1
infraorder


 0.00
5
7
2153482
1
0
superfamily

family
1
0
92421
4

 0.00
7

3

 0.00
7
219387
1
0
subfamily

genus
0
1
219389
7

 0.00
2

7
1

 0.00
330862
species

1803217
34

 0.00
5
superfamily
0
1


 0.00
4
34
27515
1
0
family

subfamily
0
1
1801551
34

 0.00
3

genus
0
1
200613
34
2

 0.00

1667466

 0.00
1
34
species

0
1
superfamily
125
21

 0.00
34725

1
1
family

 0.00
20
125
7438

109
11

 0.00
7439
3
1
subfamily

7440
25

 0.00
4
genus
0
1

85444

 0.00
1
9
species

species
881891

 0.00
1
12

species
85443
4
1

 0.00

7451

 0.00
3
55
genus
1
30

species
7454

 0.00
1
6

species
30212
19
1

 0.00

26

 0.00
3
7443
3
1
genus

species

 0.00
1
14
7445


 0.00
1
9
202808
species

subfamily
1
0
50638

 0.00
3
11

0
1
genus
11

 0.00
2
76989

11

 0.00
1
76990
species

5

 0.00
4
7455
1
0
subfamily

4

 0.00
4
76984
0
1
tribe

genus
1
1
7456
4

 0.00
3

species
91411
2
1

 0.00

1

 0.00
1
743375
species

2153479
50

 0.00
96
superfamily
1
0

1
1
family

 0.00
49
96
36668

43085
6

 0.00
2
subfamily
1
0

2

 0.00
5
141711
0
1
tribe

43086
1
2

 0.00
genus
0
1

species
1

 0.00
1
486640

0
1
genus
1
2

 0.00
604375

species
610380

 0.00
1
1

4
1
subfamily
80
21

 0.00
34695

143999
1
3

 0.00
tribe
0
1

1
0
genus
2

 0.00
1
34699

species
1
1

 0.00
456900

144004

 0.00
3
1
tribe
1
0

300110

 0.00
2
1
genus
1
0

species
1

 0.00
1
300111

tribe
1
0
144001
3

 0.00
2

0
1
genus
2

 0.00
2
64792

species
64793
1

 0.00
2

144020

 0.00
3
60
tribe
1
0

0
1
genus
60

 0.00
2
30204

60
1

 0.00
219812
species

1932955
1
3

 0.00
tribe
0
1

144030
1
2

 0.00
genus
0
1


 0.00
1
1
144034
species

1
0
tribe
5

 0.00
11
144017

1
0
genus
2

 0.00
6
13685

species

 0.00
1
6
13686

0
1
genus
5
2

 0.00
55077

species
307658
5
1

 0.00


 0.00
3
1
40138
1
0
subfamily

1

 0.00
2
56621
0
1
genus

219809

 0.00
1
1
species

subfamily
0
1
7479
7

 0.00
12

tribe
1
0
72771
5

 0.00
2

0
1
genus
1

 0.00
2
72766

species
72781

 0.00
1
1

genus
1
0
47732

 0.00
2
1

1086592
1

 0.00
1
species

72772
3

 0.00
1
tribe
1
0

1

 0.00
2
710235
0
1
genus

species
1
1

 0.00
613905

72773

 0.00
3
4
tribe
1
0

genus
1
0
13390

 0.00
2
4

104421
4

 0.00
1
species

subfamily
1
0
40139

 0.00
3
1

genus
0
1
83484
1

 0.00
2

1
1

 0.00
83485
species

subfamily
0
1
213859
4

 0.00
3

0
1
genus
4

 0.00
2
2015172

2015173
1

 0.00
4
species

superfamily
1
34
34735

 0.01
110
388

1
0
family
13

 0.00
60
124286

subfamily
0
1
156330
60

 0.00
12

156337

 0.00
3
18
tribe
1
0


 0.00
2
18
216413
1
0
genus

species
18
1

 0.00
2249760

19
4

 0.00
156332
0
1
tribe

1
5
genus

 0.00
3
19
124287

1437190
2

 0.00
14
species
1
0

subspecies
1437191
14

 0.00
1


 0.00
4
23
156331
1
0
tribe

2
1
genus
23

 0.00
3
132116

1542540
15
1

 0.00
species

6
1

 0.00
481575
species

family
0
1
7458
72
40

 0.00

78170

 0.00
5
4
subfamily
1
0

78171
4

 0.00
4
tribe
1
0

4
3

 0.00
78173
0
1
genus

1
0
subgenus

 0.00
2
4
236025

species
1

 0.00
4
156304

64

 0.00
30
70987
1
1
subfamily

354149

 0.00
3
1
tribe
1
0

354152
2

 0.00
1
genus
1
0

889126
1
1

 0.00
species

83311
50

 0.00
18
tribe
0
1

12
1
genus
50

 0.00
17
28641

144704
3
2

 0.00
subgenus
0
1

85660
1

 0.00
3
species

0
1
subgenus
2
2

 0.00
144700

species
65598
2

 0.00
1

17
5

 0.00
144703
6
1
subgenus

species
207650
1

 0.00
1

132113
2
1

 0.00
species

30191
4

 0.00
1
species

4

 0.00
1
30194
species

subgenus
2
1
28653
7
3

 0.00

species
30201
1
1

 0.00

species
1

 0.00
4
207624

144715

 0.00
2
1
subgenus
1
0

396416
1

 0.00
1
species

144708
8
2

 0.00
subgenus
3
1

5

 0.00
1
30195
species


 0.00
5
10
83321
1
0
tribe

4

 0.00
10
7459
1
3
genus

species
7461
1
1

 0.00

7460
1

 0.00
4
species

1

 0.00
2
7462
species

83319
2

 0.00
3
tribe
0
1

genus
1
0
166413
2

 0.00
2

species
561572

 0.00
1
2

4

 0.00
4
78169
0
1
subfamily

4
3

 0.00
95294
0
1
tribe

genus
0
1
95295
4
2

 0.00


 0.00
1
4
601510
species

15

 0.00
84
77572
1
0
family

1
0
subfamily

 0.00
14
84
77573

63

 0.00
10
88544
3
1
tribe

88467
44

 0.00
7
genus
4
1

0
1
subgenus
14
2

 0.00
88474


 0.00
1
14
88516
species

88475

 0.00
2
10
subgenus
1
0

species

 0.00
1
10
88514

1
0
subgenus

 0.00
2
16
88472

species
88531

 0.00
1
16

genus
1
0
88466
2

 0.00
16

species
16
1

 0.00
115100

0
1
tribe
21
3

 0.00
479730

88591
21
2

 0.00
genus
0
1

species
1190790
1

 0.00
21


 0.00
25
67
2153468
1
0
no rank

253718
24

 0.00
67
family
1
0

subfamily
0
1
253722
20

 0.00
6

tribe
0
1
288410
20

 0.00
5


 0.00
4
20
421285
1
0
subtribe

genus
0
1
421297
20

 0.00
3

species

 0.00
1
11
1126389

species
2495015
1

 0.00
9

0
1
subfamily
10

 0.00
9
253723

6

 0.00
3
421418
0
1
tribe

1
0
genus
2

 0.00
6
421423

species
6
1

 0.00
2495085


 0.00
5
4
302062
1
0
tribe

subtribe
0
1
421392
4
4

 0.00

genus
1
0
421402
3

 0.00
4

species
1889546

 0.00
1
1

3

 0.00
1
2495172
species

subfamily
1
0
288404

 0.00
4
24

302523
24

 0.00
3
tribe
0
1

421220

 0.00
2
24
genus
1
0

species
2495127
24

 0.00
1

13

 0.00
4
216423
0
1
subfamily

0
1
tribe
13

 0.00
3
288382

288388

 0.00
2
13
genus
1
0

species
1167272
13
1

 0.00

family
0
1
48719
56
12

 0.00


 0.00
11
56
205141
1
0
subfamily

56

 0.00
10
48720
5
1
genus

1
0
subgenus
2

 0.00
20
205271

species
20
1

 0.00
444401

no rank
1
1
1126400

 0.00
3
25

species

 0.00
1
9
1126402

1190802
15
1

 0.00
species

0
1
subgenus
1

 0.00
2
205272

1411666
1

 0.00
1
species

subgenus
1
0
205261
2

 0.00
5

5

 0.00
1
1411667
species

family
1
0
156323
4

 0.00
15

253710
15
3

 0.00
subfamily
0
1

genus
1
0
253714

 0.00
2
15

species
253715
15

 0.00
1

1
5
infraorder

 0.00
64
110
1955251

1
0
superfamily

 0.00
13
9
7422

family
0
1
29051
1

 0.00
4

1
0
subfamily

 0.00
3
1
272199

genus
0
1
29052
1

 0.00
2

species
29053
1

 0.00
1

family
1
0
75187

 0.00
5
4

subfamily
0
1
75190
4

 0.00
4

0
1
genus
4

 0.00
3
84507

142686
4

 0.00
2
species
2
1

subspecies
326594
1

 0.00
2

1
0
family

 0.00
3
4
7489

genus
1
0
7490
2

 0.00
4

species
7493
4

 0.00
1

1
1
superfamily
92
40

 0.00
7401

1
2
family

 0.00
29
71
7408

subfamily
1
0
65167

 0.00
5
8

231867
8

 0.00
4
tribe
0
1

genus
0
1
65288
8
3

 0.00

494744
8

 0.00
2
subgenus
0
1

1

 0.00
8
2866289
species

65149
1
4

 0.00
subfamily
0
1

no rank
1
0
273877
3

 0.00
1

92443
1
2

 0.00
genus
0
1

32260
1
1

 0.00
species

subfamily
1
0
65163

 0.00
4
19

176302
3

 0.00
19
tribe
1
0

genus
1
0
493657
2

 0.00
19

2870495
19
1

 0.00
species

65170
11

 0.00
6
subfamily
0
1

tribe
1
3
172511
5

 0.00
11

231886

 0.00
2
3
genus
1
0

species
231887
3

 0.00
1

1
0
genus
2

 0.00
5
27520

2795680
1

 0.00
5
species

5

 0.00
6
65171
1
0
subfamily

tribe
1
0
172377

 0.00
4
6

231877
6

 0.00
3
subtribe
0
1

1
0
genus

 0.00
2
6
231878

1419289
6

 0.00
1
species

0
1
subfamily
24

 0.00
4
65140

0
1
no rank
24
3

 0.00
288186

1
0
genus
2

 0.00
24
29048

species
1539398
1

 0.00
24

7402
10

 0.00
20
family
1
0

0
1
subfamily
4
3

 0.00
68882


 0.00
2
4
37852
1
0
genus

species
684658
4
1

 0.00

0
1
subfamily
8
3

 0.00
65207

1
0
genus

 0.00
2
8
51538

69319
8

 0.00
1
species

subfamily
0
1
65197
8

 0.00
3

0
1
genus
8

 0.00
2
144405

2738948
1

 0.00
8
species

40307

 0.00
10
4
superfamily
1
0

44353
3

 0.00
4
family
0
1

303412
3
3

 0.00
subfamily
0
1

0
1
genus
3

 0.00
2
63429

species
63436
1

 0.00
3

1
0
family

 0.00
5
1
73401


 0.00
4
1
1159319
1
0
subfamily

75144
1
3

 0.00
tribe
0
1

0
1
genus
1
2

 0.00
1159320

species
1

 0.00
1
2817044

superfamily
1
0
222823
4

 0.00
1

1
0
family

 0.00
3
1
27524

1
0
genus

 0.00
2
1
173784

211228
1

 0.00
1
species

7509
1
7

 0.00
order
0
1

1
6

 0.00
140693
0
1
suborder

superfamily
1
0
129369
5

 0.00
1

4

 0.00
1
7511
1
0
family

subfamily
1
0
476429
3

 0.00
1

genus
0
1
7514
1

 0.00
2

7515
1
1

 0.00
species

132

 0.01
95
33342
0
1
cohort

1
0
order
9

 0.00
6
30262

suborder
0
1
38130
6

 0.00
8

superfamily
0
1
45049
6
7

 0.00

45053
6

 0.00
6
family
0
1

153976
6
5

 0.00
subfamily
0
1

45057

 0.00
2
1
genus
1
0

species
161013
1

 0.00
1

genus
0
1
45059
5

 0.00
2

species
133901
5
1

 0.00

order
1
0
7524

 0.00
85
126


 0.00
21
8
1955247
1
0
suborder

6

 0.00
14
33365
0
1
infraorder

0
1
superfamily
1
6

 0.00
33367

0
1
family
1

 0.00
5
7033

subfamily
1
0
145353
4

 0.00
1

tribe
0
1
1137203
1
3

 0.00

0
1
genus
1
2

 0.00
2218832

2218833

 0.00
1
1
species

5

 0.00
7
33368
0
1
superfamily

6

 0.00
5
30102
1
0
family

0
1
subfamily
3

 0.00
4
33370

0
1
tribe
3
3

 0.00
565685

139475
3

 0.00
2
genus
0
1

species
3
1

 0.00
197043

subfamily
33371
2
1

 0.00

infraorder
0
1
33361
2

 0.00
6

1
0
superfamily

 0.00
5
2
36151

33362
2
4

 0.00
family
0
1

2
3

 0.00
130551
0
1
subfamily


 0.00
2
2
108930
1
0
genus

species
1

 0.00
2
108931

suborder
1
1
33373
82

 0.00
38


 0.00
28
59
33380
1
0
infraorder

8

 0.00
3
33382
1
0
superfamily

1
0
family
4

 0.00
1
33383


 0.00
3
1
38118
1
0
genus

749396
1
2

 0.00
subgenus
0
1

1

 0.00
1
133065
species

family
1
0
33384

 0.00
3
2

2
2

 0.00
58001
0
1
genus

58002

 0.00
1
2
species

superfamily
0
1
33385
56

 0.00
19

family
1
0
27482
18

 0.00
56

3

 0.00
2
805116
1
0
subfamily

2

 0.00
2
143949
0
1
genus

143950
1

 0.00
2
species

subfamily
1
1
133076
54

 0.00
14

0
1
tribe
45
8

 0.00
33387

1
0
genus

 0.00
2
1
345571

1
1

 0.00
742174
species

2
2

 0.00
40931
0
1
genus

species
2
1

 0.00
43146

genus
0
1
80764
42

 0.00
3

42
2

 0.00
464929
0
1
subgenus

1

 0.00
42
80765
species

8
5

 0.00
33386
0
1
tribe

13163

 0.00
2
2
genus
1
0

species
13164

 0.00
1
2

6
2

 0.00
7028
0
1
genus

species
7029

 0.00
1
6

1
0
superfamily

 0.00
4
1
33375

family
0
1
1585420
1
3

 0.00

121844
1

 0.00
2
genus
0
1


 0.00
1
1
121845
species

0
1
superfamily
21
5

 0.00
33377

family
1
0
7036

 0.00
4
21

0
1
subfamily
21

 0.00
3
33379

genus
0
1
7037
21
2

 0.00

1

 0.00
21
7038
species

clade
0
1
33343
36
25

 0.00

33345
24

 0.00
36
suborder
1
0

clade
0
1
33347
36

 0.00
23

22

 0.00
36
33349
1
0
clade

clade
0
1
33351
36

 0.00
21

1
0
infraorder

 0.00
5
1
33354

33355
1

 0.00
4
superfamily
0
1

30078

 0.00
3
1
family
1
0

0
1
genus
1

 0.00
2
30079

1

 0.00
1
79782
species

infraorder
1
0
33357

 0.00
15
35

superfamily
1
0
38105

 0.00
5
25

1
0
family

 0.00
4
25
186376

25
3

 0.00
2068237
0
1
subfamily

genus
0
1
1276926
25
2

 0.00

25
1

 0.00
1545138
species

0
1
superfamily
10
9

 0.00
33358

160513
10

 0.00
8
family
0
1

10

 0.00
7
286710
0
1
subfamily

0
1
genus
1

 0.00
2
85309

species
85310
1
1

 0.00

0
1
genus
8
2

 0.00
631388

species
1511221

 0.00
1
8

1
0
genus
2

 0.00
1
286705

species
286706

 0.00
1
1

307
89

 0.01
6657
0
1
subphylum

52

 0.00
16
2172819
0
1
superclass

6670
52
15

 0.00
class
0
1

52

 0.00
14
43953
0
1
subclass

order
1
1
84318
52

 0.00
13

24

 0.00
5
116574
0
1
suborder

116575

 0.00
4
24
superfamily
1
0

69351

 0.00
3
24
family
1
0


 0.00
2
24
69354
1
0
genus

69355
1

 0.00
24
species

suborder
0
1
84328
27
7

 0.00

27
6

 0.00
84329
0
1
superfamily

27

 0.00
5
43954
1
1
family


 0.00
2
15
399044
1
0
genus

15

 0.00
1
399045
species

genus
0
1
163713
11

 0.00
2

species
163714

 0.00
1
11

1
0
class

 0.00
9
10
6658

1
0
subclass
8

 0.00
10
116557

1
0
order

 0.00
7
10
84337

6

 0.00
10
6665
1
0
suborder

116561
10
5

 0.00
infraorder
0
1

1
0
family
4

 0.00
10
77658

3

 0.00
10
6668
1
3
genus

6669

 0.00
1
6
species

35525
1

 0.00
1
species

1
1
superclass
245

 0.01
63
2172821

72037

 0.00
10
126
class
1
0

subclass
1
0
6830
9

 0.00
126

infraclass
0
1
116569
126

 0.00
8

116571

 0.00
7
126
superorder
1
0

6

 0.00
126
72033
1
0
order

5

 0.00
126
72034
1
1
family

0
1
genus
64

 0.00
2
217164

species
217165

 0.00
1
64

0
1
genus
61

 0.00
2
72035


 0.00
1
61
72036
species

105

 0.00
46
6681
0
1
class

105

 0.00
45
72041
0
1
subclass

6820
3
12

 0.00
superorder
0
1

6821
11

 0.00
3
order
1
0

1
0
suborder

 0.00
10
3
1732196

0
1
infraorder
3
9

 0.00
1732206

0
1
superfamily
1

 0.00
4
199476

family
0
1
199477
1
3

 0.00

199487

 0.00
2
1
genus
1
0

species
294128
1

 0.00
1

0
1
superfamily
2

 0.00
4
2992673

family
0
1
199478
2

 0.00
3

genus
0
1
280676
2
2

 0.00

species
2
1

 0.00
317513


 0.00
32
102
6682
1
0
superorder

6683
102

 0.00
31
order
0
1

0
1
suborder
96

 0.00
23
6692

6694
86
5

 0.00
infraorder
0
1

115580

 0.00
4
86
superfamily
1
0

family
1
0
6695
3

 0.00
86

genus
1
0
6696

 0.00
2
86

1

 0.00
86
159736
species

infraorder
1
0
6712
10

 0.00
9

4
5

 0.00
6724
0
1
superfamily

1
0
family
4

 0.00
4
6725

4

 0.00
3
72430
0
1
subfamily


 0.00
2
4
6726
1
0
genus

species
6728

 0.00
1
4

37849
4

 0.00
5
superfamily
1
0

6704
5
3

 0.00
family
0
1

6705

 0.00
2
5
genus
1
0

species
5
1

 0.00
6706

6752
7

 0.00
1
infraorder
1
0

no rank
0
1
116704
1
6

 0.00

116707

 0.00
5
1
no rank
1
0

29962
1
4

 0.00
superfamily
0
1

72876
1
3

 0.00
family
0
1

genus
0
1
95601
1

 0.00
2

95602
1
1

 0.00
species

suborder
0
1
6684
6
7

 0.00

111520
6

 0.00
6
superfamily
0
1

family
0
1
6685
6
5

 0.00

133894
6

 0.00
4
genus
0
1

species
139456

 0.00
1
1

2

 0.00
1
6687
species

1

 0.00
3
27405
species

class
0
1
116172
13

 0.00
6

13
5

 0.00
6675
0
1
subclass

infraclass
1
0
37909
4

 0.00
13

1
0
family
3

 0.00
13
38011

1
0
genus
2

 0.00
13
51649

51650
13

 0.00
1
species

phylum
2
1
6231
110

 0.00
56

119088
3
6

 0.00
class
0
1

subclass
0
1
1457286
3

 0.00
5

0
1
order
3
4

 0.00
6329

1
0
family

 0.00
3
3
6332

genus
0
1
6333
3

 0.00
2

species
6334
3

 0.00
1

class
1
0
119089

 0.00
49
105

order
1
0
6308

 0.00
5
27

1
0
superfamily
4

 0.00
27
6314


 0.00
3
27
126387
1
0
family

6288

 0.00
2
27
genus
1
0

species
6289
1

 0.00
27

43

 0.00
78
6236
1
0
order

2301116

 0.00
18
57
suborder
1
0

2301119
57

 0.00
17
infraorder
0
1

1
0
superfamily
16

 0.00
57
55879

15

 0.00
57
6243
1
0
family

55885
22

 0.00
6
subfamily
0
1

6237
22
5

 0.00
genus
0
1

31234
2
1

 0.00
species

species
6239
3
1

 0.00

species
10

 0.00
1
1978547

6238
7
1

 0.00
species

subfamily
1
0
55887

 0.00
8
35

35

 0.00
7
42476
0
1
genus

141969
20

 0.00
1
species

species
473156

 0.00
1
10

species
1559960
1

 0.00
1

no rank
1
1
2613844

 0.00
3
4

species
2879419

 0.00
1
1


 0.00
1
2
2879420
species

suborder
1
0
6300

 0.00
15
18

2082223
14

 0.00
18
infraorder
1
0

superfamily
1
0
2082224
13

 0.00
18

8

 0.00
15
6246
1
0
family

131309

 0.00
2
3
genus
1
0

species
1

 0.00
3
131310


 0.00
5
12
6247
1
0
genus

75913
1

 0.00
2
species

174720
3

 0.00
1
species

6

 0.00
1
34506
species

species
6248
1
1

 0.00

1
0
family
4

 0.00
3
114888


 0.00
3
3
114889
1
0
genus

2629767
3
2

 0.00
no rank
0
1

114890
3

 0.00
1
species

1
0
suborder
9

 0.00
3
6274


 0.00
8
3
2072716
1
0
infraorder

7

 0.00
3
6295
1
0
superfamily

1

 0.00
3
6296
0
1
family

6278
2

 0.00
1
genus
1
0

species
6279
1

 0.00
1

2

 0.00
3
48791
0
1
family

genus
1
1
48796
2
2

 0.00

108094
1

 0.00
1
species

6040
16

 0.00
12
phylum
0
1

0
1
class
16

 0.00
11
6042

1779148
7

 0.00
5
subclass
0
1

order
1
0
1779149

 0.00
4
7

0
1
family
7

 0.00
3
129258

genus
1
0
68573
2

 0.00
7

species
7

 0.00
1
68574

9

 0.00
5
1779146
0
1
subclass

9
4

 0.00
6049
0
1
order

68562
9
3

 0.00
family
0
1

9

 0.00
2
68563
0
1
genus

species
9

 0.00
1
68564

2687318
1
4

 0.00
class
0
1

1
0
genus

 0.00
3
1
192874

species
1
0
192875

 0.00
2
1

strain

 0.00
1
1
595528

1
0
clade
7

 0.00
1
2611341

5719

 0.00
6
1
phylum
1
0

1

 0.00
5
37104
0
1
order

4

 0.00
1
181550
1
0
family

1
0
genus

 0.00
3
1
5721

species
0
1
5722
1
2

 0.00

isolate
1

 0.00
1
412133

1
0
clade
30

 0.00
18
2611352

5752
7

 0.00
6
phylum
1
0

2601529

 0.00
6
6
clade
1
0

clade
0
1
2601530
6

 0.00
5

6

 0.00
4
5765
0
1
family

1
0
genus

 0.00
3
6
5761

species
1

 0.00
1
5762

species
5
1

 0.00
5763

1
0
phylum
22

 0.00
12
33682

12
21

 0.00
5653
0
1
class

2704647
20

 0.00
12
subclass
1
0


 0.00
16
11
2704949
1
0
order

0
1
family
11
15

 0.00
5654

1286322

 0.00
7
5
subfamily
1
0

5658

 0.00
6
5
genus
1
0

38568
5

 0.00
5
subgenus
1
1

2

 0.00
2
38582
1
1
species group


 0.00
1
1
5659
species

1
1
species group
2

 0.00
2
38574

5661
1

 0.00
1
species

5690
4

 0.00
5
genus
1
0

1
0
subgenus
3

 0.00
5
47569

1
0
species

 0.00
2
5
5692

strain
5

 0.00
1
1068625

1581334
1

 0.00
3
subfamily
0
1

1003337
1

 0.00
2
genus
0
1

1

 0.00
1
59799
species

1
0
order
3

 0.00
1
2704649

genus
0
1
5709
1
2

 0.00

5710
1

 0.00
1
species


 0.01
177
221
2698737
1
4
clade

33630

 0.00
80
120
clade
1
0

5794
109
55

 0.00
phylum
0
1

23

 0.00
20
1280412
0
1
class

0
1
subclass
23

 0.00
19
5796

1
0
order
18

 0.00
23
75739

suborder
0
1
423054
23
17

 0.00

35082
4

 0.00
2
family
1
0

genus
0
1
5806
2
3

 0.00

857276
1
1

 0.00
species

1

 0.00
1
5807
species

0
1
family
17

 0.00
8
5809


 0.00
4
8
5810
1
0
genus

8
3

 0.00
5811
7
1
species

0
1
biotype
1

 0.00
2
398031

432359
1
1

 0.00
strain

genus
0
1
29175
9

 0.00
3

species
1
0
29176

 0.00
2
9

9

 0.00
1
572307
strain

5799
4

 0.00
4
family
1
0

1
0
genus
3

 0.00
4
5800

species
3
1

 0.00
5802

44415
1

 0.00
1
species

0
1
class
86

 0.00
34
422676

10
1
order
85
29

 0.00
5819

0
1
family
1

 0.00
3
1639122

genus
0
1
195944
1

 0.00
2

no rank
257649
1

 0.00
1

1
1
family
4

 0.00
26
1639121

77521
25

 0.00
3
genus
0
1

0
1
no rank
25

 0.00
2
219820

191679
25
1

 0.00
species

48
21

 0.00
1639119
0
1
family


 0.00
20
48
5820
1
6
genus

0
1
subgenus
12

 0.00
6
418103

species
2

 0.00
1
5850

species
5855

 0.00
1
1

species
4

 0.00
1
5858

36330
3
1

 0.00
species

species
2
1

 0.00
5827

subgenus
7
1
418107
11

 0.00
4


 0.00
1
2
720590
species

species
880535
1
1

 0.00

5833

 0.00
1
1
species

2

 0.00
3
418104
1
0
subgenus

5849
1

 0.00
3
species

418101

 0.00
6
15
subgenus
1
7

5860

 0.00
2
1
species
1
0

subspecies
119398

 0.00
1
1

1

 0.00
6
5821
species

5825
2

 0.00
1
species
1
0

1

 0.00
1
5826
subspecies

1

 0.00
1
208452
species

5863

 0.00
4
1
order
1
0

family
0
1
27994
1

 0.00
3

1

 0.00
2
5873
0
1
genus

species
1
1

 0.00
68886

5

 0.00
1
2497438
1
0
phylum

clade
0
1
27997
1
4

 0.00

1

 0.00
3
27998
0
1
order

2

 0.00
1
27999
1
0
family

genus
28000

 0.00
1
1

phylum
1
0
5878
19

 0.00
10

subphylum
1
1
431838
10
18

 0.00

6020

 0.00
17
9
class
1
2

subclass
1
0
6021

 0.00
5
1

0
1
order
1

 0.00
4
1974272

1
0
family

 0.00
3
1
85904

0
1
genus
1

 0.00
2
168243

168244
1

 0.00
1
species

order
0
1
31277
4
6

 0.00

suborder
0
1
37093
4

 0.00
5

family
0
1
291294
4

 0.00
4

4
3

 0.00
5890
0
1
genus

species
1
0
5911

 0.00
2
4

strain
312017
4
1

 0.00

1
1
order
5

 0.00
2
33825

1

 0.00
4
340080
0
1
family

5884
1

 0.00
3
genus
0
1

1
2

 0.00
5888
0
1
species

strain
1
1

 0.00
412030

clade
1
1
33634

 0.00
87
94

2696291
87

 0.00
68
clade
21
1

1
0
class
5

 0.00
2
5747

425074

 0.00
4
2
order
1
0

2
3

 0.00
425072
0
1
family

1
0
genus

 0.00
2
2
5748

145522

 0.00
1
2
species

2836

 0.00
40
51
phylum
1
8

1
6

 0.00
33853
0
1
class

subclass
0
1
33854
1

 0.00
5

order
1
0
33855
4

 0.00
1

family
0
1
33856
1
3

 0.00

1
0
genus
2

 0.00
1
35129

species
210441
1

 0.00
1

class
1
0
33849
17

 0.00
28

16

 0.00
28
33850
1
6
clade


 0.00
4
2
245176
1
0
order

245177

 0.00
3
2
family
1
0

2
2

 0.00
420972
0
1
genus

2809013
2

 0.00
1
species

order
2
1
38748
20
11

 0.00

10

 0.00
18
67474
1
10
family

genus
2
1
50949
5

 0.00
4

1
0
no rank

 0.00
2
1
655776

species
1923966
1

 0.00
1

species
1

 0.00
2
355634


 0.00
2
1
286098
1
0
genus

568900
1

 0.00
1
species

67475
2
3

 0.00
genus
0
1

species
2018706
1

 0.00
1

1

 0.00
1
2018708
species

33858
1
2

 0.00
no rank
0
1


 0.00
1
1
659498
species


 0.00
14
13
33836
1
1
class

33846
8

 0.00
11
subclass
1
4

1

 0.00
4
2910910
0
1
order

1365474

 0.00
3
1
family
1
0

1
0
genus

 0.00
2
1
29206

29207
1
1

 0.00
species

order
1
1
33847
6
3

 0.00

1
2
family

 0.00
2
5
29202

genus
35127

 0.00
1
3

1
0
subclass

 0.00
5
1
420266

order
0
1
265576
1
4

 0.00

family
0
1
265577
1

 0.00
3

1
2

 0.00
265578
0
1
genus

451786
1

 0.00
1
species


 0.00
6
2
35675
1
0
class

order
0
1
54409
2

 0.00
5

genus
0
1
35676
1
2

 0.00

species
1

 0.00
1
35677

1
2

 0.00
44055
0
1
genus

species
44056
1

 0.00
1

class
1
0
33859
6

 0.00
4

order
0
1
420622
4

 0.00
5

1
1
family
4

 0.00
4
420617

genus
2988

 0.00
1
1

2

 0.00
2
2991
1
1
genus

species
1

 0.00
1
52557

class
2
1
2825
7
10

 0.00

0
1
order
2
4

 0.00
420604

98655
2

 0.00
3
family
0
1

2995

 0.00
2
2
genus
1
0

1

 0.00
2
2996
species

order
1
1
96792
3

 0.00
5

family
1
1
98651

 0.00
4
2

3

 0.00
1
2985
1
0
genus

no rank
1
0
2635268

 0.00
2
1

species
420557
1

 0.00
1

3
12

 0.00
4762
0
1
phylum

order
1
0
4776

 0.00
4
1

4777
1

 0.00
3
family
0
1

4780
1
2

 0.00
genus
0
1

species
1

 0.00
1
4781

370421
4

 0.00
1
order
1
0


 0.00
3
1
65355
1
0
family

1
0
genus

 0.00
2
1
65356

species
653948
1
1

 0.00

121069
1

 0.00
3
order
0
1

1

 0.00
2
4782
0
1
family

genus
1448052
1

 0.00
1

1
0
class
6

 0.00
3
2683628

0
1
clade
3

 0.00
5
2683629

3

 0.00
4
42740
0
1
order

2547934
3

 0.00
3
family
0
1

12967

 0.00
2
3
genus
1
0

12968

 0.00
1
3
species

9

 0.00
3
543769
1
0
clade

0
1
phylum
3

 0.00
8
136419

29197
2
3

 0.00
class
0
1

2

 0.00
2
227085
0
1
genus

1

 0.00
2
227086
species

188941
1
4

 0.00
order
0
1

3

 0.00
1
45105
1
0
family


 0.00
2
1
45106
1
0
genus

species
45107
1
1

 0.00

class
1
0
3027

 0.00
6
8

order
0
1
589342
8
5

 0.00

589343
4

 0.00
8
family
1
0

55528
8

 0.00
3
genus
0
1

8

 0.00
2
55529
2
1
species

strain
6

 0.00
1
905079

2763
4

 0.00
15
phylum
0
1

2797
3

 0.00
9
class
0
1

29216
2
4

 0.00
order
0
1


 0.00
3
2
31345
1
0
family

2
2

 0.00
1034309
0
1
genus


 0.00
1
2
1134705
species

1
0
order
4

 0.00
1
2798

0
1
family
1
3

 0.00
2799

1
2

 0.00
2791
0
1
genus

species
2891951

 0.00
1
1

class
0
1
446134
1
5

 0.00

0
1
order
1
4

 0.00
282338

family
1
0
446133
3

 0.00
1

446135
2

 0.00
1
genus
1
0

1

 0.00
1
446136
species

kingdom
4
1
33090
2263
668

 0.09

phylum
1
0
35493

 0.09
606
2218

0
1
subphylum
2218
605

 0.09
131221

1
22
clade
604

 0.09
2218
3193

clade
0
1
3208
22

 0.00
26

1
0
clade
25

 0.00
22
404260

3214

 0.00
24
22
class
1
0

114658

 0.00
14
20
subclass
1
0

404315

 0.00
8
19
superorder
1
0

13798
19

 0.00
7
order
0
1

1
0
family

 0.00
3
10
61526

1
0
genus

 0.00
2
10
67427

species
1

 0.00
10
67428

0
1
family
9

 0.00
3
28466


 0.00
2
9
28467
1
0
genus

species
9
1

 0.00
28468

5

 0.00
1
404297
1
0
superorder


 0.00
4
1
3226
1
0
order


 0.00
3
1
37411
1
0
family

genus
1
0
37412

 0.00
2
1

species

 0.00
1
1
37413

1
0
subclass
4

 0.00
1
114657

1
0
order
3

 0.00
1
38585

2

 0.00
1
38586
1
0
family

1

 0.00
1
200749
genus

subclass
1
0
114656

 0.00
5
1

4

 0.00
1
3215
1
0
order

0
1
family
1

 0.00
3
3216

37414
2

 0.00
1
genus
1
0

1

 0.00
1
3218
species

clade
0
1
58023
2154
569

 0.08

78536
2153
563

 0.08
clade
2
1

2151
562

 0.08
58024
3
1
clade

class
1
1
3398
2134
545

 0.08

261009
4

 0.00
2
order
1
0

0
1
family
2

 0.00
3
22097

genus
1
0
13332

 0.00
2
2

1

 0.00
2
13333
species

2126

 0.08
536
1437183
113
1
clade

1
0
order
7

 0.00
2
232378

1
0
family
3

 0.00
1
4429

4430

 0.00
2
1
genus
1
0

1

 0.00
1
4432
species

1
0
family
3

 0.00
1
4328

1
0
genus
2

 0.00
1
4329

species
1

 0.00
1
60698

0
1
order
2
5

 0.00
41768

0
1
family
2
4

 0.00
3465

2
3

 0.00
1462614
0
1
subfamily

2

 0.00
2
3468
0
1
genus

species
1

 0.00
2
3469

0
1
clade
1652
409

 0.06
71240

clade
1
0
91827
408

 0.06
1652

168
1
clade
1652
407

 0.06
1437201


 0.02
142
429
71274
1
7
clade

0
1
order
32
14

 0.00
41945

family
1
0
3623

 0.00
4
1

1

 0.00
3
78230
0
1
genus

1

 0.00
2
448037
0
1
species

2486852

 0.00
1
1
subspecies

19955

 0.00
3
5
family
1
0

genus
1
0
13492

 0.00
2
5

1

 0.00
5
35925
species

26

 0.00
6
25692
0
1
family

genus
0
1
35939
26
5

 0.00

species
253017
25
1

 0.00


 0.00
3
1
1758100
1
0
subgenus

1758102
1

 0.00
2
section
0
1

1

 0.00
1
253027
species

76

 0.00
46
91882
1
1
clade

4209
30
32

 0.00
order
1
1


 0.00
31
29
4210
1
1
family

1
0
subfamily

 0.00
7
3
219103


 0.00
6
3
102818
1
0
tribe

742010
3
5

 0.00
subtribe
0
1

1
0
genus

 0.00
4
3
4264

1
0
species

 0.00
3
3
4265

1
0
subspecies

 0.00
2
3
309979

varietas
1

 0.00
3
59895

0
1
subfamily
1

 0.00
4
219102

0
1
tribe
1
3

 0.00
495130


 0.00
2
1
40564
1
0
genus

1
1

 0.00
711043
species

219120
4

 0.00
5
subfamily
0
1

219121

 0.00
4
4
tribe
1
0

745062
4
3

 0.00
subtribe
0
1

4235

 0.00
2
4
genus
1
0

4
1

 0.00
4236
species

0
1
subfamily
20

 0.00
14
102804

102806
13
4

 0.00
tribe
0
1

911294

 0.00
3
13
subtribe
1
0

13

 0.00
2
56534
0
1
genus

species

 0.00
1
13
56535

1
0
tribe
5

 0.00
3
102809

877976
4

 0.00
3
clade
1
0

2841728
3

 0.00
3
subtribe
0
1


 0.00
2
3
41574
1
0
genus

3
1

 0.00
72917
species

4

 0.00
4
911341
1
0
clade

102814
4

 0.00
3
tribe
0
1

0
1
genus
4
2

 0.00
4231

4232
4
1

 0.00
species

45
13

 0.00
4036
0
1
order

1
0
suborder

 0.00
12
45
364270

0
1
family
3

 0.00
8
4037

241778
7

 0.00
3
subfamily
1
0

241789
3
6

 0.00
tribe
0
1

241799
3
5

 0.00
subtribe
0
1

4038
3

 0.00
4
genus
1
1

2
3

 0.00
1873447
0
1
section

4039

 0.00
2
2
species
1
1

subspecies
79200

 0.00
1
1

family
1
0
4050
3

 0.00
42

4051

 0.00
2
42
genus
1
0

species

 0.00
1
42
4052

clade
5
1
91888
314

 0.01
81

4069
28

 0.01
170
order
1
3

family
0
1
4118
25

 0.00
5

tribe
0
1
267213
25

 0.00
4


 0.00
3
25
4119
1
6
genus

35885
1

 0.00
6
species

13
1

 0.00
35884
species

4070

 0.01
22
142
family
1
1

424554

 0.00
5
2
subfamily
1
0

1
0
tribe

 0.00
4
2
424562

2
3

 0.00
4085
0
1
genus

1

 0.00
1
49451
species

4096
1

 0.00
1
species

16

 0.01
139
424551
1
0
subfamily


 0.00
4
11
424564
1
0
tribe


 0.00
3
11
4071
1
1
genus

4072
10
2

 0.00
species
9
1

varietas
165789

 0.00
1
1

1
0
tribe
8

 0.00
127
424574

genus
1
9
4107
7

 0.00
127

species
45834

 0.00
1
27

1
5
subgenus

 0.00
3
47
49274

species
19
1

 0.00
28526

species
4081
23
1

 0.00

17

 0.00
1
50273
species


 0.00
1
27
4113
species

1
0
tribe

 0.00
3
1
424573


 0.00
2
1
126908
1
0
genus


 0.00
1
1
126910
species

4143
38

 0.00
125
order
1
4

family
1
0
156152
4

 0.00
19

0
1
tribe
19

 0.00
3
216780

genus
1
0
102598

 0.00
2
19

species
102599
19

 0.00
1

1
0
family
14

 0.00
48
4136

subfamily
1
0
216703

 0.00
4
9

1
0
tribe

 0.00
3
9
983543

genus
1
0
155228
2

 0.00
9

194200
9

 0.00
1
species

subfamily
1
0
216706

 0.00
6
2

5

 0.00
2
216718
1
0
tribe

subtribe
1
0
2836339

 0.00
4
2

21880
3

 0.00
2
genus
1
1

0
1
no rank
1

 0.00
2
2291027

49212
1

 0.00
1
species

subfamily
0
1
216702
37
3

 0.00

genus
0
1
4139
37
2

 0.00

species
53169
1

 0.00
37

1
0
family
5

 0.00
2
4185

216691
2

 0.00
4
subfamily
0
1

1
0
tribe

 0.00
3
2
216695

2
2

 0.00
175693
0
1
genus


 0.00
1
2
175694
species

family
1
0
41399
3

 0.00
1

1
0
genus
2

 0.00
1
1502711

species
1

 0.00
1
4155

family
1
0
4144

 0.00
11
51

1
0
tribe

 0.00
7
27
426106

genus
1
0
4145

 0.00
4
2

3

 0.00
2
4146
1
0
species

1
0
subspecies

 0.00
2
2
158383

158386

 0.00
1
2
varietas

1
0
genus
2

 0.00
25
38871

species
56036
25

 0.00
1

24

 0.00
3
426105
0
1
tribe

24
2

 0.00
4147
0
1
genus

660624
24

 0.00
1
species


 0.00
14
14
4055
1
0
order

24966
13
8

 0.00
family
0
1

169618
7

 0.00
13
subfamily
1
0

clade
0
1
1968429
13

 0.00
6


 0.00
5
13
1968428
1
0
clade

tribe
1
0
169640

 0.00
4
13

0
1
genus
13
3

 0.00
13442

species
49369
12

 0.00
1

species
1
1

 0.00
13443


 0.00
5
1
21472
1
0
family

0
1
tribe
1

 0.00
4
303185

1
0
subtribe

 0.00
3
1
2546018

1
0
genus

 0.00
2
1
50807

1

 0.00
1
50808
species

1
0
order

 0.00
19
64
3524

family
1
0
1804623

 0.00
11
45

0
1
subfamily
44
7

 0.00
1307796


 0.00
3
2
1307775
1
0
tribe

2

 0.00
2
3561
0
1
genus

3562
1

 0.00
2
species

1307774
42

 0.00
3
tribe
0
1

0
1
genus
42
2

 0.00
3558


 0.00
1
42
63459
species

0
1
subfamily
1

 0.00
3
1804621

2

 0.00
1
3554
1
0
genus

species

 0.00
1
1
161934

0
1
family
19
7

 0.00
3615

1110380
19
6

 0.00
subfamily
0
1

1110384
2

 0.00
1
tribe
1
0

1
1

 0.00
61508
genus

tribe
0
1
1110385
18
3

 0.00

18

 0.00
2
46786
0
1
genus

18
1

 0.00
137693
species


 0.04
245
991
71275
1
83
clade

91835
517
152

 0.02
clade
31
1

72025
170

 0.01
51
order
0
1

50

 0.01
170
3803
1
0
family

0
1
subfamily
169
44

 0.01
3814

0
1
clade
169

 0.01
43
2231393

2231384
5

 0.00
39
clade
1
0

clade
0
1
2231385
39
4

 0.00

39
3

 0.00
163729
0
1
tribe


 0.00
2
39
3869
1
0
genus

39
1

 0.00
3871
species

clade
1
1
2231382

 0.00
31
118

1
3
clade
12

 0.00
42
2233855

tribe
1
0
163715

 0.00
3
2

3815
2

 0.00
2
genus
0
1

species
3816
2

 0.00
1

163735
37

 0.00
8
tribe
0
1

3846
3

 0.00
3
genus
0
1


 0.00
2
3
1462606
1
1
subgenus

species
2
1

 0.00
3847

3913
34
4

 0.00
genus
10
1

2

 0.00
11
3914
1
0
species

157739
11

 0.00
1
varietas

species
1

 0.00
13
3917

75

 0.00
18
2233838
0
1
clade

2233857
14

 0.00
4
clade
0
1

1
0
tribe

 0.00
3
14
163747

genus
0
1
3867
14

 0.00
2

species
34305
1

 0.00
14

61

 0.00
13
2233839
0
1
clade

0
1
tribe
43

 0.00
6
163742

1
1
genus
3

 0.00
41
3877

species
70936
27

 0.00
1

3880
1

 0.00
13
species

3898
2
2

 0.00
genus
0
1

species
2
1

 0.00
57577

1
0
tribe

 0.00
3
1
163743

3887
1

 0.00
2
genus
0
1

3888
1

 0.00
1
species

0
1
tribe
17

 0.00
3
163722

1
0
genus

 0.00
2
17
3826

species
3827
17
1

 0.00

2231387

 0.00
6
12
clade
1
0

0
1
tribe
12

 0.00
5
163725

clade
1
0
2231390
4

 0.00
12

genus
2
1
3817
12

 0.00
3


 0.00
1
8
3818
species

species
130454
2
1

 0.00

3804
1

 0.00
5
subfamily
0
1


 0.00
4
1
3807
1
0
clade

tribe
1
0
163487
3

 0.00
1

genus
0
1
35715
1

 0.00
2

species
1

 0.00
1
207710

order
1
1
3744
143
34

 0.01

family
0
1
3481
3
3

 0.00

1
0
genus

 0.00
2
3
3482

species
3

 0.00
1
3483

3745
27

 0.01
138
family
1
3

171638
60

 0.00
15
subfamily
1
1

10
8

 0.00
721789
0
1
tribe

no rank
0
1
1184125
6

 0.00
3

0
1
genus
6
2

 0.00
23204

species
6
1

 0.00
57926

0
1
subtribe
4
4

 0.00
1184124

0
1
genus
4

 0.00
3
3746

4

 0.00
2
57918
0
1
species

subspecies
101020
1

 0.00
4

5
3

 0.00
1176516
0
1
no rank

3764

 0.00
2
5
genus
1
0

species
74649
5

 0.00
1

44

 0.00
3
721790
0
1
tribe

44

 0.00
2
3761
0
1
genus

species
44

 0.00
1
57919

171637
75
11

 0.00
subfamily
1
1

tribe
0
1
721805
15

 0.00
4

genus
1
3
3754
3

 0.00
15

11
1

 0.00
3755
species

1

 0.00
1
42229
species

1
1
tribe
6

 0.00
59
721813

1
1
genus
3

 0.00
2
3766

species
1

 0.00
2
225117

genus
1
28
3749
3

 0.00
55

3752
1

 0.00
4
species

species

 0.00
1
23
3750

3487

 0.00
3
1
family
1
0

3497
2

 0.00
1
genus
1
0

1
1

 0.00
981085
species

1
4

 0.00
233875
0
1
order

1
0
family

 0.00
3
1
4305


 0.00
2
1
123484
1
0
genus


 0.00
1
1
458696
species

order
1
0
3502
19

 0.00
81

1
1
family
9

 0.00
5
16714


 0.00
3
7
16718
1
1
genus

species
2

 0.00
1
51240

species
2249226
1

 0.00
4

91223

 0.00
1
1
genus

1
0
family
6

 0.00
52
3503

1
0
genus

 0.00
2
13
21024

28930
1

 0.00
13
species

0
1
genus
39
3

 0.00
3511

species

 0.00
1
38
38942

1

 0.00
1
97700
species

family
1
0
3514

 0.00
7
20

genus
1
0
12989
2

 0.00
9

9
1

 0.00
176864
species

genus
0
1
3504
1

 0.00
2

1
1

 0.00
3508
species


 0.00
2
10
13450
1
0
genus

species
1

 0.00
10
13451

54

 0.00
28
3646
1
1
order

1
0
family

 0.00
3
1
3683

1
2

 0.00
3684
0
1
genus

species
78168
1
1

 0.00

3

 0.00
10
4004
1
0
family


 0.00
2
10
4005
1
0
genus

4006

 0.00
1
10
species

3977
37

 0.00
17
family
0
1

0
1
subfamily
22

 0.00
6
235629

tribe
0
1
235880
22
5

 0.00


 0.00
2
15
3984
1
0
genus

15
1

 0.00
3986
species

genus
1
0
3987
2

 0.00
7

species
3988
1

 0.00
7

235631
10

 0.00
15
subfamily
1
0

0
1
tribe
3

 0.00
3
235883

3982
2

 0.00
3
genus
1
0

species
3

 0.00
1
3983

tribe
0
1
235882
5
3

 0.00

3980
2

 0.00
5
genus
1
0

species
3981
1

 0.00
5

tribe
1
0
235887
3

 0.00
7

2

 0.00
7
3995
1
0
genus

180498

 0.00
1
7
species

3688
5

 0.00
4
family
0
1


 0.00
3
5
238069
1
0
tribe

3689
5

 0.00
2
genus
1
1

species
3694
1

 0.00
4

1
0
order
15

 0.00
37
71239

family
1
1
3650
37

 0.00
14

1003878
5

 0.00
6
tribe
1
0

6
4

 0.00
3660
3
1
genus

3662
1

 0.00
1
species

2

 0.00
2
3663
1
1
species

3664
1

 0.00
1
subspecies

1003877

 0.00
8
30
tribe
1
0

3

 0.00
23
3655
1
0
genus

species
3656
1

 0.00
18


 0.00
1
5
3659
species

3653
1

 0.00
2
genus
0
1

1

 0.00
1
3654
species


 0.00
2
6
102210
1
0
genus

species
102211
1

 0.00
6

91836
362
84

 0.01
clade
24
1

128

 0.00
32
3699
0
1
order

family
1
3
3700
31

 0.00
128

980083
24
8

 0.00
tribe
0
1

2

 0.00
4
71323
1
0
genus


 0.00
1
4
883000
species

3718
4
2

 0.00
genus
0
1

species

 0.00
1
4
81985

genus
2
1
3701
16

 0.00
3

1

 0.00
10
3702
species

species
38785
4
1

 0.00

981099
14

 0.00
3
tribe
0
1

genus
0
1
13287
14
2

 0.00

species
13288
14
1

 0.00

981070
21

 0.00
3
tribe
0
1

genus
0
1
50451
21
2

 0.00


 0.00
1
21
50452
species

1
0
tribe

 0.00
3
3
981100

2

 0.00
3
98005
1
1
genus


 0.00
1
2
72664
species

2
1
tribe
59

 0.00
7
981071

2

 0.00
27
3725
1
0
genus

species
3726
1

 0.00
27

genus
13
1
3705
30
4

 0.00

species
3708

 0.00
1
7

5

 0.00
1
3711
species

species
3712
5

 0.00
1

1
0
tribe

 0.00
3
3
980085

0
1
genus
3

 0.00
2
76872

115917
3

 0.00
1
species

1

 0.00
3
980193
0
1
tribe

0
1
genus
1

 0.00
2
50460

1

 0.00
1
558126
species

0
1
order
52
14

 0.00
41937

family
0
1
4011
2
3

 0.00

23461
2

 0.00
2
genus
0
1

species
29780
2

 0.00
1

0
1
family
46

 0.00
3
23808

46

 0.00
2
23809
0
1
genus

species
2768810
46

 0.00
1

0
1
family
1
3

 0.00
91851

43874
1

 0.00
2
genus
0
1

species

 0.00
1
1
357929

23513
4

 0.00
3
family
1
0

subfamily
0
1
1728959
3
3

 0.00

2

 0.00
3
2706
1
2
genus

species
85681
1

 0.00
1

41944
17

 0.00
29
order
1
0

3928
3

 0.00
3
family
0
1

genus
0
1
22662
3
2

 0.00

species
3

 0.00
1
22663

5
8

 0.00
3931
0
1
family

5
7

 0.00
1699513
0
1
subfamily

3
3

 0.00
1699522
0
1
tribe

178174
2

 0.00
3
genus
1
0

species
3

 0.00
1
219896

1
0
tribe

 0.00
3
2
1699524

0
1
genus
2
2

 0.00
3932

species
2

 0.00
1
71139

3934

 0.00
5
21
family
1
0

1585427

 0.00
4
21
subfamily
1
0

21

 0.00
3
1585433
0
1
tribe

0
1
genus
21
2

 0.00
238243

21

 0.00
1
13055
species

41938
20

 0.00
129
order
1
0

family
4
1
3629
129
19

 0.00

1
0
subfamily
3

 0.00
1
214915

1

 0.00
2
66655
0
1
genus

66656
1

 0.00
1
species

subfamily
0
1
214909
11

 0.00
5

1
0
genus
2

 0.00
1
108869

1
1

 0.00
108875
species

1
0
genus
2

 0.00
10
3640

species
1

 0.00
10
3641

subfamily
1
2
214907

 0.00
10
113

7

 0.00
92
3633
1
21
genus

species
4

 0.00
1
47622

16

 0.00
1
34274
species

29730

 0.00
1
19
species

species
34284
22

 0.00
1

3635
8
1

 0.00
species

species
29729
2

 0.00
1

47614
19

 0.00
2
genus
0
1

1

 0.00
19
47615
species

0
1
no rank
29
8

 0.00
91834

29

 0.00
7
403667
0
1
order

1
0
family

 0.00
6
29
3602

29
5

 0.00
2304100
0
1
tribe

3603

 0.00
4
29
genus
1
5

103349

 0.00
1
14
species

29760

 0.00
1
9
species

1

 0.00
1
96939
species

114

 0.01
357
4447
1
1
clade

342
107

 0.01
1437197
0
1
subclass

91

 0.01
325
4734
1
1
clade


 0.01
74
288
38820
1
1
order

1
0
family
5

 0.00
33
14101

genus
1
0
13578
2

 0.00
15

species
13579

 0.00
1
15

46322
18

 0.00
2
genus
0
1

59018
18
1

 0.00
species

5

 0.00
17
4613
1
0
family

1909378
17
4

 0.00
subfamily
0
1

genus
1
0
4614

 0.00
3
17

1
13
species

 0.00
2
17
4615

4

 0.00
1
296719
varietas

237
63

 0.01
4479
3
1
family

clade
1
0
147370

 0.00
30
26

subfamily
6
1
147369
26

 0.00
29


 0.00
14
16
1648036
1
0
no rank

0
1
tribe
16

 0.00
13
147428

1
0
subtribe

 0.00
4
5
1293365


 0.00
3
5
4539
1
0
genus

section
1
0
2100771

 0.00
2
5

1

 0.00
5
38727
species

subtribe
0
1
1293360
6

 0.00
3

genus
0
1
66017
6
2

 0.00

1010633

 0.00
1
6
species

1293361

 0.00
5
5
subtribe
1
0

genus
1
0
4554
2

 0.00
3

1

 0.00
3
4556
species


 0.00
2
2
4583
1
0
genus

species
2

 0.00
1
154765

1
0
no rank
14

 0.00
4
1648033


 0.00
13
4
147429
1
0
tribe

1648026
6

 0.00
2
subtribe
1
0

4546
5

 0.00
2
genus
1
0

1
0
no rank
4

 0.00
2
286192

2

 0.00
1
128810
1
0
species

no rank
131158
1
1

 0.00

62335

 0.00
1
1
species

subtribe
0
1
1648028
1
3

 0.00

2

 0.00
1
4557
1
0
genus

species
4558
1

 0.00
1

0
1
subtribe
1
3

 0.00
1648029

0
1
genus
1

 0.00
2
4575

species

 0.00
1
1
4577

359160
208

 0.01
32
clade
0
1

0
1
subfamily
28
8

 0.00
147367

tribe
0
1
147380
28
7

 0.00

subtribe
0
1
1648021
28

 0.00
6

4527
28
5

 0.00
genus
0
1

1
16
species

 0.00
3
26
4530

3
1

 0.00
39946
no rank

39947
7

 0.00
1
no rank

4533
2

 0.00
1
species

3
1
subfamily
180

 0.01
23
147368

9

 0.00
110
1648038
1
0
no rank

110

 0.00
8
147389
10
1
tribe

1648030
100
7

 0.00
subtribe
28
1

3

 0.00
70
4564
1
21
genus

4565

 0.00
1
47
species

2

 0.00
1
85692
species

genus
1
0
4480
3

 0.00
2

1
0
species
2

 0.00
2
37682

subspecies
2

 0.00
1
200361

67

 0.00
13
1648037
0
1
no rank

1
1
tribe
67

 0.00
12
147387

1652080

 0.00
4
49
clade
1
1

subtribe
0
1
640623
48
3

 0.00

4496
2

 0.00
48
genus
1
0

species

 0.00
1
48
4498

1
0
clade
7

 0.00
17
1652081

2948571
17

 0.00
6
clade
0
1

subtribe
0
1
640630
17

 0.00
5

genus
1
2
4520

 0.00
2
16

species

 0.00
1
14
4522

1
2

 0.00
4605
0
1
genus

species
1

 0.00
1
4608

4618

 0.00
9
35
order
1
0


 0.00
5
32
4637
1
0
family

4640
32
4

 0.00
genus
3
1

species
1
0
4641
2

 0.00
20

subspecies

 0.00
1
20
214687

species
320322
1

 0.00
9


 0.00
3
3
4642
1
0
family

1
0
genus

 0.00
2
3
4650

species
94328
3

 0.00
1

1
0
order

 0.00
7
1
40551

1
6

 0.00
4710
0
1
family

169697
5

 0.00
1
subfamily
1
0

tribe
0
1
169705
1
4

 0.00

0
1
subtribe
1
3

 0.00
169729

1
0
genus

 0.00
2
1
51952

1

 0.00
1
51953
species


 0.00
10
11
73496
1
0
order

family
0
1
4668
7

 0.00
5

subfamily
1
0
40553
4

 0.00
7

tribe
1
0
703248

 0.00
3
7


 0.00
2
7
4678
1
2
genus

species
5

 0.00
1
4679

40552
4

 0.00
4
family
0
1

703533

 0.00
3
4
subfamily
1
0

4
2

 0.00
4685
0
1
genus

species

 0.00
1
4
4686

1
0
order

 0.00
5
6
40548

6
4

 0.00
4671
0
1
family

1
0
genus
3

 0.00
6
4672

6

 0.00
2
29710
0
1
species

subspecies
55577

 0.00
1
6

order
1
0
16360

 0.00
6
14

family
0
1
4454
14

 0.00
5

subfamily
1
0
284551
4

 0.00
14

genus
1
0
4473

 0.00
3
14

1

 0.00
10
29656
species

species
51605
4

 0.00
1

261007

 0.00
4
5
order
1
0

4410
3

 0.00
5
family
1
0

0
1
genus
5

 0.00
2
4418

species
1

 0.00
5
210225

16

 0.00
14
1437180
1
0
clade

58019
14

 0.00
15
class
0
1


 0.00
14
14
3313
1
0
subclass


 0.00
13
14
2821352
1
0
clade

14

 0.00
12
1446380
0
1
order

3318

 0.00
11
14
family
1
2

2

 0.00
2
3321
0
1
genus

species
3322
2

 0.00
1

1
0
genus

 0.00
3
2
3328

species
1

 0.00
1
3330

species
1

 0.00
1
3329

genus
0
1
3337
8
5

 0.00

1
1
subgenus
2

 0.00
7
139271

species

 0.00
1
6
3352

subgenus
1
0
139272
2

 0.00
1

species
3348

 0.00
1
1

1
0
class
5

 0.00
1
1521260

4

 0.00
1
3244
1
0
order

family
1
0
3245

 0.00
3
1

3246
1
2

 0.00
genus
0
1

species
88036
1

 0.00
1

clade
1
0
3195
8

 0.00
20

186770
20
7

 0.00
class
0
1

0
1
subclass
20
6

 0.00
186774

28908
20
5

 0.00
order
0
1

29585
4

 0.00
20
family
1
0

0
1
genus
20
3

 0.00
3196

0
1
species
20

 0.00
2
3197

subspecies
1

 0.00
20
1480154

phylum
1
1
3041

 0.00
61
41

1
0
class

 0.00
12
6
1035538

13792

 0.00
11
6
order
1
0

1
0
family
7

 0.00
3
1525212

2

 0.00
2
41874
0
1
genus

species
41875

 0.00
1
2

4

 0.00
1
70447
1
0
genus

1
0
no rank
3

 0.00
1
2268852

0
1
species
1

 0.00
2
242159

strain
436017

 0.00
1
1

41873
3

 0.00
3
family
0
1

1
0
genus

 0.00
2
3
38832

species
3

 0.00
1
296587

1
0
no rank
4

 0.00
3
34155

family
1
0
41878
3

 0.00
3

genus
1
0
41879

 0.00
2
3

species
1

 0.00
3
41880

30

 0.00
22
2692248
1
0
clade

4

 0.00
10
75966
0
1
class

1

 0.00
4
2507901
0
1
order

1

 0.00
3
2507902
0
1
family

114064
1
2

 0.00
genus
0
1

species
1

 0.00
1
3171

no rank
1
0
75981
5

 0.00
3

2511161
3
4

 0.00
clade
0
1

41891
3

 0.00
3
genus
1
0

1
0
no rank
2

 0.00
3
2688356

3

 0.00
1
2315456
species

class
1
1
3166
18

 0.00
19

2812636
18

 0.00
17
clade
1
1

order
0
1
35491
1
4

 0.00

0
1
family
1

 0.00
3
35466


 0.00
2
1
39954
1
0
genus

species
81817

 0.00
1
1

1
0
order
13

 0.00
15
3042

3043
5

 0.00
3
family
0
1

5

 0.00
2
3044
0
1
genus

species
257627
5

 0.00
1

family
1
0
3051

 0.00
5
4

40525
2

 0.00
2
genus
1
0

40532
2

 0.00
1
species

1
0
genus

 0.00
2
2
3052

3055
2
1

 0.00
species

3065

 0.00
4
6
family
1
0


 0.00
3
6
3066
1
0
genus

species
1
0
3067
2

 0.00
6

6
1

 0.00
3068
forma

4
9

 0.00
33103
0
1
class

clade
0
1
2546215
4

 0.00
8

31306
7

 0.00
4
order
1
1

2682567
3

 0.00
1
family
1
0

2045111
2

 0.00
1
genus
1
0

species
1

 0.00
1
160070

family
1
0
205394
3

 0.00
2

0
1
genus
2
2

 0.00
43940

species
43941

 0.00
1
2

0
1
class
5

 0.00
5
2302911

2302912
5

 0.00
4
order
0
1

family
1
0
2302913

 0.00
3
5

5
2

 0.00
2302914
0
1
genus

5

 0.00
1
1764295
species

0
1
no rank
9

 0.00
11
2787854

0
1
no rank
9

 0.00
10
28384

1
1
no rank
7

 0.00
7
81077

no rank
1
0
29278
3

 0.00
2

species
1238570
1
1

 0.00

2797847
1

 0.00
1
species

3
1

 0.00
32630
species

1

 0.00
2
111786
0
1
no rank

species
111789
1

 0.00
1

2

 0.00
2
36549
1
0
no rank

2
1

 0.00
45202
species

1

 88.53
2296941

2157
61

 0.00
106
superkingdom
1
1


 0.00
66
47
28890
1
0
phylum

3

 0.00
5
183968
0
1
class

order
1
0
2258
4

 0.00
3

2259
3
3

 0.00
family
0
1

0
1
genus
3

 0.00
2
2263

1

 0.00
3
1505907
species

1
0
clade
39

 0.00
25
2290931

17

 0.00
15
183963
1
0
class


 0.00
5
1
1644060
1
0
order

1644061

 0.00
4
1
family
1
0

genus
1
0
88723
3

 0.00
1


 0.00
2
1
69525
1
0
species

strain
797303

 0.00
1
1

14

 0.00
11
2235
0
1
order

1
0
family

 0.00
4
1
1963268

genus
0
1
63743
1

 0.00
3

no rank
1
0
2634096
2

 0.00
1

1
1

 0.00
2961939
species

1
0
family
6

 0.00
13
2236

12
2

 0.00
2239
0
1
genus

2039234
1

 0.00
12
species

1070314
1

 0.00
3
genus
0
1

1

 0.00
2
2643768
0
1
no rank

species
1

 0.00
1
2953749


 0.00
21
10
224756
1
0
class

570264
1
5

 0.00
order
0
1

570265
4

 0.00
1
family
1
0

570266
3

 0.00
1
genus
1
0

1
2

 0.00
1175444
0
1
species


 0.00
1
1
1041930
strain

6

 0.00
4
94695
1
0
order

family
1
0
2206
5

 0.00
4

genus
0
1
2207
4
4

 0.00

1

 0.00
2
2644672
no rank

species
2208

 0.00
1
1

1

 0.00
1
2209
species

9

 0.00
5
2191
1
0
order

family
0
1
196137
3

 0.00
3

1
0
genus

 0.00
2
3
2202

species
1

 0.00
3
2203

family
0
1
2194
2

 0.00
5

4

 0.00
2
45989
1
0
genus

2

 0.00
1
2198
1
0
species

strain
1

 0.00
1
368407

species
1

 0.00
1
83986

18
19

 0.00
2283794
1
1
clade

class
0
1
183925
16

 0.00
12


 0.00
11
16
2158
1
0
order

1
0
family

 0.00
10
16
2159

4
1
genus
7

 0.00
2
145260

species
145261

 0.00
1
3

genus
1
2
2160
3

 0.00
6

2627676
2

 0.00
4
no rank
1
0


 0.00
1
4
1379702
species

1
1
genus
4

 0.00
3
2172

1
1

 0.00
230361
species

0
1
no rank
1

 0.00
2
2638681

1609968
1
1

 0.00
species

183939

 0.00
6
1
class
1
0


 0.00
5
1
2182
1
0
order

4

 0.00
1
196117
1
0
family

0
1
genus
1
3

 0.00
196118

73913
1
2

 0.00
species
0
1

strain
1

 0.00
1
579137

1
0
no rank
2

 0.00
1
68359

1457825
1

 0.00
1
species

phylum
1
0
2283796
7

 0.00
1

1
6

 0.00
183967
0
1
class


 0.00
5
1
2301
1
0
order

family
1
0
46630
4

 0.00
1

46631

 0.00
3
1
genus
1
0

0
1
species
1

 0.00
2
82076


 0.00
1
1
263820
strain

1
0
clade

 0.00
28
10
1783275

phylum
0
1
928852
2
3

 0.00

1700837
2

 0.00
2
no rank
1
0

2

 0.00
1
2026714
species

0
1
phylum
4
11

 0.00
651137

0
1
class
1

 0.00
5
1643678

order
1
0
1033996

 0.00
4
1

family
1
0
1033997
3

 0.00
1

1826864
1
2

 0.00
genus
0
1

species
1

 0.00
1
1826872

1

 0.00
2
651140
0
1
no rank

species
1
1

 0.00
1167203

2

 0.00
3
651142
0
1
no rank

2

 0.00
2
1078904
0
1
genus

2
1

 0.00
1078905
species

4

 0.00
13
28889
0
1
phylum

1
0
class
12

 0.00
4
183924

1
5

 0.00
114380
0
1
order

family
0
1
2272
1

 0.00
4

1
0
genus

 0.00
3
1
2279

2280
1

 0.00
2
species
0
1

399550
1

 0.00
1
strain

3

 0.00
6
2281
0
1
order

0
1
family
3

 0.00
5
118883

0
1
genus
2
2

 0.00
2100760

2286
2
1

 0.00
species

1

 0.00
2
2284
0
1
genus

no rank
2641160
1

 0.00
1

1935183
2
4

 0.00
clade
0
1

1
0
phylum
3

 0.00
2
1936272

species
2026747
1

 0.00
1

2876572
1
1

 0.00
species


 0.00
7
14
2787823
1
0
no rank

14

 0.00
6
12908
0
1
no rank


 0.00
4
13
151659
1
0
no rank

no rank
1
0
81490

 0.00
2
8


 0.00
1
8
198431
species

5

 0.00
1
155900
species

species
32644
1

 0.00
1
