# Supplementary material for: Microbiome and mitogenomics of the chigger mite Pentidionis agamae: potential role as an Orientia vector and associations with divergent clades of Wolbachia and Borrelia
Source: BMC Genomics. 2024 Apr 17;25:380. doi: 10.1186/s12864-024-10301-6 (PMC11025265; doi:10.1186/s12864-024-10301-6)
Supplement: Supplementary file 4 — Additional file 4: Kronagram for Kraken2 output at confidence threshold 0.1 for Pentidionis agamae pool Pa2 [file 12864_2024_10301_MOESM4_ESM.html]

Javascript must be enabled to view this page.

members
magnitude
magnitudeUnassigned
count
unassigned
taxon
rank

Pagamae\_01\_report

4035562
15

 20.14 20.01 0.00 0.00 0.00 0.00 0.00 0.00 0.00 0.00 0.00 0.00 0.00 0.00 0.00
8781
17627

1
210
192
10239
20

 0.01
superkingdom

0
2732004
33
8
1
clade

 0.00

0
2732005
32
8
1
kingdom

 0.00

11
1
3
0
2732008

 0.00
phylum


 0.00
class
1
2
5
2732529
0

1
2
4
2732557
0

 0.00
order


 0.00
family
2
1
3
0
1511858


 0.00
genus
1
2
2
0
1511859

754004
1
2
species

 0.00

class

 0.00
0
2732528
5
1
1

4
1
1
2732556
0

 0.00
order

family

 0.00
0
1914302
3
1
1

0
2268405
2
1
1
no rank

 0.00

species

 0.00
2420055
1
1

1
5
20
0
2732007

 0.00
phylum

class

 0.00
0
2732523
1
4
12

order

 0.00
0
2732524
4
1
1

3
1
1
10501
0

 0.00
family

2
1
1
0
455363

 0.00
no rank


 0.00
species
1
1
2023057

order

 0.00
2732554
0
1
3
7


 0.00
family
6
3
1
549779
0

no rank

 0.00
0
985780
5
3
1


 0.00
subfamily
3
2
1
1977630
0

1
2
2
1977636
0

 0.00
genus


 0.00
species
1
2
1977638

species

 0.00
2487768
1
1

7
1
1
0
2732525

 0.00
class

order

 0.00
0
2732527
1
1
6


 0.00
family
1
1
5
0
10240

subfamily

 0.00
10241
0
4
1
1


 0.00
genus
1
1
3
2005509
0

2025358
0
2
1
1
no rank

 0.00

2025360
1
1
species

 0.00

1
12
51
0
2559587

 0.00
clade

3
1
10
2732397
0

 0.00
kingdom

phylum

 0.00
2732409
0
9
1
3

0
2732514
1
3
8
class

 0.00

0
2169561
7
1
3
order

 0.00

11632
0
6
1
3
family

 0.00

no rank

 0.00
35276
0
2
1
1

1435008
1
1
species

 0.00


 0.00
subfamily
3
2
1
0
327045


 0.00
genus
2
2
1
11646
0

1
2
11676

 0.00
species

40
1
9
0
2732396

 0.00
kingdom

0
2497569
1
7
23
phylum

 0.00

0
2497571
5
1
12
subphylum

 0.00

0
2497576
6
4
1
class

 0.00

order

 0.00
0
1980410
5
1
4


 0.00
family
4
1
4
1980412
0

genus

 0.00
675845
0
4
1
3

species

 0.00
2845511
0
2
4
1

4
1
2719116

 0.00
no rank


 0.00
class
1
1
5
0
2497577

4
1
1
0
2499411

 0.00
order

3
1
1
11308
0

 0.00
family

0
197911
1
1
2
genus

 0.00

1
1
11320

 0.00
species

no rank

 0.00
0
2613794
2
1
1

2746656
1
1
species

 0.00


 0.00
subphylum
1
1
8
2497570
0


 0.00
class
7
1
1
0
2497574

order

 0.00
11157
0
6
1
1

family

 0.00
0
11158
5
1
1

1
1
4
0
2560076

 0.00
subfamily

0
2560155
1
1
3
genus

 0.00


 0.00
no rank
1
1
2
2686069
0


 0.00
species
1
1
2877485

phylum

 0.00
2732408
0
10
1
1

0
2732506
1
1
9
class

 0.00


 0.00
order
8
1
1
76804
0

2499399
0
1
1
7
suborder

 0.00

family

 0.00
0
11118
1
1
6

subfamily

 0.00
2501931
0
5
1
1


 0.00
genus
1
1
4
694002
0

1
1
3
0
2509511

 0.00
subgenus

1
1
2
694009
0

 0.00
species

2697049
1
1
no rank

 0.00

0
2732406
1
1
6
phylum

 0.00

5
1
1
0
2732462

 0.00
class

2732545
0
4
1
1
order

 0.00

0
11050
1
1
3
family

 0.00

1
1
2
0
11102

 0.00
genus

11103
1
1
species

 0.00

0
2731341
134
1
53
clade

 0.00

2731360
0
52
1
134
kingdom

 0.00

phylum

 0.00
0
2731361
8
1
2

class

 0.00
2731363
0
7
1
2

6
1
2
548681
0

 0.00
order


 0.00
family
5
1
2
0
10292

2
1
4
10357
0

 0.00
subfamily

0
10365
3
2
1
genus

 0.00


 0.00
species
1
2
2
10366
0

1
2
524651

 0.00
no rank


 0.00
phylum
1
132
43
2731618
0

42
1
132
22
2731619

 0.00
class


 0.00
genus
2
1
2843418

3
1
7
0
2946170

 0.00
family

1
1
1198137

 0.00
genus

2
1
1
0
1198136

 0.00
subfamily

1913653
1
1
genus

 0.00

3
1
1
2842517
0

 0.00
subfamily

1
1
2
2842766
0

 0.00
genus

species

 0.00
260149
1
1

0
2731691
5
1
1
family

 0.00

0
1910999
4
1
1
subfamily

 0.00


 0.00
genus
3
1
1
0
187217


 0.00
species
2
1
1
2734021
0

no rank

 0.00
2175169
1
1

genus

 0.00
16
1982251
4
1
18

0
1982264
2
1
1
species

 0.00

no rank

 0.00
1229792
1
1

1
1
2079398

 0.00
no rank

1
6
2
2100420
0

 0.00
no rank


 0.00
species
6
1
2100421

no rank
2788787
9
71

species

 0.00
2202567
1
9

2828115
1
1
species

 0.00

species

 0.00
2827580
1
1

2170413
47
1
species

 0.00

1
1
1636270

 0.00
species

1
1
2825508

 0.00
species

1
1
2686311

 0.00
species

1
9
2202564

 0.00
species

species

 0.00
331278
1
1

order

 0.00
1978007
0
1
1
3

no rank

 0.00
2949298
0
1
1
2

species

 0.00
2831617
1
1


 0.00
genus
2
2
1
2843426
1

1
1
2844294

 0.00
species

subfamily

 0.00
2946646
0
1
1
2


 0.00
genus
1
1
2733124


 0.00
genus
1
1
2948791

2560131
2
1
genus

 0.00

family
2946160
1
1

1
1
2960609
no rank

1
1
2783673

 0.00
species

1
1
3
2731643
0

 0.00
family

1
1
2
2731646
0

 0.00
subfamily


 0.00
genus
1
1
2731674

1
17
41
0
2731342

 0.00
clade

kingdom

 0.00
2732091
0
7
1
3


 0.00
phylum
6
3
1
0
2732412


 0.00
class
5
3
1
0
2732413

order

 0.00
0
2732414
3
1
4

1
10841
3
1
3
family

 0.00

no rank

 0.00
117574
0
2
1
2

species

 0.00
2202644
1
2

0
2732092
33
1
14
kingdom

 0.00

0
2732415
13
1
3
phylum

 0.00

1
2
6
2732422
0

 0.00
class

order

 0.00
2732534
0
5
2
1


 0.00
family
4
2
1
10780
0


 0.00
subfamily
2
1
3
40119
0

no rank

 0.00
207678
0
2
2
1


 0.00
species
2
1
1341019

6
1
1
2732421
0

 0.00
class

1
1
5
2732533
0

 0.00
order

family

 0.00
0
151340
4
1
1

333774
0
3
1
1
no rank

 0.00

173087
0
1
1
2
clade

 0.00


 0.00
species
1
1
10566

11
1
19
2732416
2

 0.00
phylum

class

 0.00
2732423
0
5
1
2

order

 0.00
0
2732536
1
2
4

3
2
1
39724
0

 0.00
family

642248
0
1
2
2
no rank

 0.00

1954248
1
2
species

 0.00

0
2748377
4
5
1
no rank

 0.00

clade

 0.00
0
2202562
1
3
2

3
1
2656686

 0.00
species

2875815
2
1
species

 0.00

class

 0.00
0
2732424
2
1
9

0
2732539
8
2
1
order

 0.00


 0.00
family
7
1
2
0
1910928

no rank

 0.00
1941235
0
2
1
1

1
1
2202565

 0.00
species

genus

 0.00
1542744
0
1
1
4

0
2844427
3
1
1
species

 0.00


 0.00
no rank
1
1
2
0
1985376

no rank

 0.00
1843740
1
1

no rank

 0.00
0
12333
1
6
2

6
1
38018

 0.00
species

186616
0
3
9
1
no rank

 0.00

species

 0.00
239364
1
1


 0.00
species
8
1
340016

12429
0
1
4
8
no rank

 0.00

no rank

 0.00
0
2204151
4
1
7

no rank

 0.00
0
51368
4
1
6

2
1
1
0
2060084

 0.00
genus

species

 0.00
1349410
1
1

species

 0.00
2591644
1
1

2023203
0
2
2
1
family

 0.00

species

 0.00
2506597
1
2

0
2787823
4
1
6
no rank

 0.00

12908
0
5
4
1
no rank

 0.00


 0.00
no rank
4
4
1
151659
0

species

 0.00
360281
1
1

358574
1
1
species

 0.00


 0.00
species
2
1
155900

5740
783223
1
2759
38101

 19.41
superkingdom

0
554915
30
11
1
clade

 0.00


 0.00
phylum
1
1
7
0
555280

1
1
6
0
1485168

 0.00
order

5
1
1
555407
0

 0.00
clade

0
33677
4
1
1
family

 0.00

0
5754
3
1
1
genus

 0.00

2
1
1
0
5755

 0.00
species

strain

 0.00
1257118
1
1


 0.00
phylum
1
10
22
0
2605435

1
3
8
555406
0

 0.00
clade

0
2682482
3
1
7
order

 0.00


 0.00
family
1
3
6
33084
0

5758
1
5
3
1
genus

 0.00

species

 0.00
412467
0
1
1
2


 0.00
strain
1
1
1076696

0
33085
1
1
2
species

 0.00

1
1
370355

 0.00
strain

class

 0.00
0
142796
13
7
1

33083
0
12
7
1
clade

 0.00

2058181
0
1
3
5
order

 0.00

0
2058183
4
3
1
family

 0.00

genus

 0.00
133407
0
3
1
3


 0.00
species
3
1
2
361139
0

1410327
3
1
strain

 0.00


 0.00
order
4
1
6
0
2058949

family

 0.00
2058185
0
5
4
1

genus

 0.00
0
5782
1
4
4

3
1
5786

 0.00
species

2
1
1
0
44689

 0.00
species

1
1
352472

 0.00
strain


 0.00
phylum
35
8
1
2763
0

5
1
22
2806
0

 0.00
class

6
1
2
1443949
0

 0.00
subclass

order

 0.00
31491
0
5
2
1

family

 0.00
31492
0
4
2
1

genus

 0.00
0
31493
1
2
3

2645983
0
2
1
2
no rank

 0.00


 0.00
species
2
1
2485823

0
2045258
5
1
1
subclass

 0.00

4
1
1
0
2807

 0.00
order


 0.00
family
1
1
3
73096
0


 0.00
genus
1
1
2
268565
0

268567
1
1
species

 0.00

0
2045261
1
2
10
subclass

 0.00

order

 0.00
0
2802
1
1
5

1
1
4
2803
0

 0.00
family

2008390
0
1
1
3
tribe

 0.00

228261
0
2
1
1
genus

 0.00

228262
1
1
species

 0.00

4
1
1
31468
0

 0.00
order

family

 0.00
0
31469
3
1
1

1
1
2
2774
0

 0.00
genus

species

 0.00
2510778
1
1

class

 0.00
2797
0
12
3
1


 0.00
order
7
2
1
265318
0

0
265316
6
1
2
family

 0.00

45156
0
1
1
3
genus

 0.00


 0.00
species
2
1
1
45157
0

1
1
280699

 0.00
strain

genus

 0.00
0
83373
1
1
2


 0.00
species
1
1
130081

4
1
1
0
29216

 0.00
order

family

 0.00
31345
0
1
1
3


 0.00
genus
2
1
1
2784
0


 0.00
species
1
1
2786

10
4
1
3027
0

 0.00
class


 0.00
order
5
1
2
589342
0

4
2
1
589343
0

 0.00
family

3
2
1
0
55528

 0.00
genus

species

 0.00
0
55529
1
2
2

905079
1
2
strain

 0.00


 0.00
order
4
2
1
0
589350

3
1
2
0
589351

 0.00
family


 0.00
genus
2
2
1
77924
0

2
1
464988

 0.00
species

8
1
6
2608109
0

 0.00
clade

7
6
1
2830
0

 0.00
phylum

clade

 0.00
0
2608131
6
1
6


 0.00
order
1
6
5
0
73020

0
418966
6
1
4
family

 0.00

0
2902
1
6
3
genus

 0.00

1
6
2
2903
0

 0.00
species

280463
6
1
strain

 0.00

clade

 18.36
6892
33154
740886
1
4648

class

 0.00
28009
0
5
1
8

0
1924738
5
1
7
order

 0.00

81529
0
5
1
6
family

 0.00

0
81525
3
1
1
genus

 0.00


 0.00
species
1
1
2
0
81824

strain

 0.00
431895
1
1

genus

 0.00
86017
0
2
1
4


 0.00
species
4
1
946362

3872
1
732729
446
33208

 18.16
kingdom

clade

 18.15
953
6072
3856
732253
1


 0.00
phylum
2
1
11
10197
0

10
2
1
0
140493

 0.00
class

140471
0
1
1
5
order

 0.00

140472
0
4
1
1
family

 0.00


 0.00
genus
3
1
1
140473
0

2
1
1
0
2619977

 0.00
no rank

1
1
1532212

 0.00
species


 0.00
order
4
1
1
0
27921


 0.00
family
3
1
1
51105
0

1
1
2
0
27922

 0.00
genus

27923
1
1
species

 0.00

clade

 18.12
33213
65144
3783
731188
1


 1.88
clade
1
75858
2277
33317
10494

1206794
940
2005
1
62140
clade

 1.54

7
1
1
1215728
0

 0.00
superphylum

33467
0
6
1
1
phylum

 0.00

0
2082883
1
1
5
class

 0.00

1
1
4
2082909
0

 0.00
order

1
1
3
0
37891

 0.00
family


 0.00
genus
2
1
1
37847
0


 0.00
species
1
1
37621


 1.51
clade
1929
60928
1
2
88770

42241
0
7
1
1
phylum

 0.00


 0.00
class
6
1
1
42242
0


 0.00
order
1
1
5
0
149990


 0.00
superfamily
1
1
4
0
2558949


 0.00
family
1
1
3
0
58669

0
58670
2
1
1
genus

 0.00

1
1
232323

 0.00
species

1
60925
1921
1279
6656

 1.51
phylum

57817
1
1803
2
197563

 1.43
clade

clade

 1.43
2517
197562
1802
57815
1

180
6960
1695
54679
1
subphylum

 1.35

0
30001
11
53
1
class

 0.00

79705
0
1
46
4
order

 0.00


 0.00
family
3
46
1
0
39131

genus

 0.00
39132
0
1
46
2

species

 0.00
39272
1
46


 0.00
order
1
7
6
0
730330


 0.00
superfamily
5
1
7
730333
0

0
36141
4
7
1
family

 0.00

subfamily

 0.00
0
187620
7
1
3

1
7
2
0
158440

 0.00
genus

species

 0.00
158441
1
7

54446
1
1683
0
50557

 1.35
class

54446
1
1682
1
85512

 1.35
clade


 1.35
subclass
54445
1
1681
7496
55

17
21
1
33339
0

 0.00
infraclass

30073
0
7
1
1
order

 0.00


 0.00
suborder
6
1
1
185812
0


 0.00
infraorder
1
1
5
170974
0

4
1
1
0
170976

 0.00
superfamily

family

 0.00
0
219473
1
1
3

genus

 0.00
0
650628
2
1
1


 0.00
species
1
1
2680887

20
1
9
6961
0

 0.00
order

8
20
1
50488
0

 0.00
suborder


 0.00
superfamily
7
20
1
0
70894

0
70895
19
1
3
family

 0.00


 0.00
genus
19
1
2
79456
0

197161
1
19
species

 0.00

3
1
1
126177
0

 0.00
family

genus

 0.00
126230
0
2
1
1


 0.00
species
1
1
126231

infraclass

 1.35
33340
5298
1663
1
54369

66
1
1154
27
33341

 0.03
cohort


 0.00
order
1
20
12
6993
0

20
1
11
0
7001

 0.00
suborder

0
1955150
10
20
1
infraorder

 0.00

9
1
20
70910
0

 0.00
no rank

superfamily

 0.00
92621
0
8
1
20

family

 0.00
4
7002
1
20
7


 0.00
subfamily
1
16
6
37267
0

genus

 0.00
7008
1
5
1
16

species

 0.00
0
2023354
1
1
2

subspecies

 0.00
2023355
1
1


 0.00
species
1
3
7011

7010
11
1
species

 0.00

1
246
21
2
50622

 0.01
order

superfamily

 0.00
70405
0
15
185
1

143727
0
4
1
56
family

 0.00


 0.00
subfamily
1
56
3
0
143771

56
1
2
0
143731

 0.00
genus

species

 0.00
2065413
56
1

143733
0
4
45
1
family

 0.00

1
45
3
0
143769

 0.00
subfamily

genus

 0.00
0
143734
1
45
2


 0.00
species
45
1
143735

6
84
1
0
62802

 0.00
family


 0.00
subfamily
1
84
5
0
143773

genus

 0.00
0
62803
2
16
1

16
1
2014036

 0.00
species

genus

 0.00
143721
0
2
68
1

143722
68
1
species

 0.00

superfamily

 0.00
70404
0
1
59
5

4
59
1
0
50623

 0.00
family

0
466866
3
59
1
subfamily

 0.00

143767
0
2
1
59
genus

 0.00

552050
1
59
species

 0.00

order

 0.02
0
7020
19
839
1


 0.00
suborder
1
1
5
213547
0

infraorder

 0.00
0
523721
1
1
4


 0.00
superfamily
1
1
3
0
523866


 0.00
family
2
1
1
58556
0

524121
1
1
subfamily

 0.00


 0.02
suborder
13
1
838
0
523712

superfamily

 0.02
213545
0
838
1
12

family

 0.02
213546
0
1
838
11


 0.02
genus
1
838
10
61471
417


 0.00
species
55
1
61476

1
134
629358

 0.00
species

species

 0.00
170557
21
1

species

 0.00
629360
1
40

33
1
61478

 0.00
species

1
13
61484

 0.00
species

33
1
61472

 0.00
species


 0.00
species
1
49
170555


 0.00
species
43
1
61474

0
6970
13
22
1
superorder

 0.00

order

 0.00
0
85823
12
1
22

0
1049657
1
22
11
superfamily

 0.00

0
1912919
22
1
10
no rank

 0.00

family

 0.00
0
7501
1
6
5


 0.00
subfamily
6
1
4
127820
0


 0.00
tribe
3
6
1
0
127821

genus

 0.00
0
7502
2
1
6

6
1
136037

 0.00
species


 0.00
family
1
16
4
46562
0

0
105801
3
1
16
subfamily

 0.00

genus

 0.00
0
60568
2
16
1

1
16
105785

 0.00
species


 1.18
cohort
47649
1
1493
13497
33392

7509
0
7
1
7
order

 0.00


 0.00
suborder
1
7
6
0
140693

5
7
1
129369
0

 0.00
superfamily

family

 0.00
7511
0
4
7
1


 0.00
subfamily
7
1
3
0
476429

2
7
1
7514
0

 0.00
genus

species

 0.00
7515
1
7

85604
1250
28099
1
608
superorder

 0.70


 0.01
order
13
1
479
0
30263

93875
0
479
1
12
suborder

 0.01

479
1
11
1683728
0

 0.01
infraorder


 0.01
superfamily
10
1
479
41033
0

1
479
9
50645
0

 0.01
family


 0.01
subfamily
1
479
8
0
177669

7
479
1
177673
16

 0.01
tribe

4
1
343
177674
41

 0.01
genus

70
1
1218281

 0.00
species

species

 0.00
1271730
1
93

species

 0.00
692089
139
1

0
1271741
2
120
1
genus

 0.00

1271742
1
120
species

 0.00


 0.65
order
26370
1
594
7088
210

suborder

 0.00
0
41024
1
70
4

family

 0.00
41025
0
3
1
70


 0.00
genus
1
70
2
0
41026

70
1
1042620

 0.00
species

589
26090
1
0
41191

 0.65
suborder

infraorder

 0.65
0
41196
1
26090
588

587
26090
1
271
41197

 0.65
parvorder


 0.00
superfamily
7
114
1
1
37584

family

 0.00
98958
0
3
45
1

0
101736
1
45
2
genus

 0.00

species

 0.00
101737
45
1

3
1
68
30222
0

 0.00
family

753374
0
2
68
1
genus

 0.00

68
1
753375

 0.00
species

clade

 0.64
4805
37567
579
1
25705

98
104430
1
1438
70
clade

 0.04

8
131
1
0
104435

 0.00
superfamily

4
37
1
0
115354

 0.00
family


 0.00
subfamily
37
1
3
287187
0

287110
0
1
37
2
genus

 0.00

37
1
287375

 0.00
species

family

 0.00
0
115353
3
1
94

2
1
94
287107
0

 0.00
genus

1
94
287200

 0.00
species


 0.00
superfamily
4
18
1
0
104437

family

 0.00
252293
0
18
1
3

252294
0
2
1
18
genus

 0.00

252295
18
1
species

 0.00

superfamily

 0.00
0
104432
5
1
98

0
186108
98
1
4
family

 0.00


 0.00
subfamily
98
1
3
0
1556158

2
98
1
0
655084

 0.00
genus


 0.00
species
98
1
1101072

15
333
1
0
104434

 0.01
superfamily


 0.01
family
14
1
333
0
106496

13
1
333
106499
3

 0.01
subfamily

8
1
217
5
301641

 0.01
tribe


 0.00
genus
144
1
5
5
106500

species

 0.00
1108569
1
27

1108570
1
36
species

 0.00

45
1
1660703

 0.00
species


 0.00
species
31
1
301036

2
68
1
300855
0

 0.00
genus


 0.00
species
68
1
301037


 0.00
tribe
1
113
4
301638
0

3
113
1
4
287191

 0.00
genus

287197
1
37
species

 0.00

72
1
748215

 0.00
species

37
1
760
37568
0

 0.02
superfamily

family

 0.02
21
7139
1
760
36

1
571
26
12
81687

 0.01
subfamily

8
1
179
9
581389

 0.00
tribe

80
1
3
10
572704

 0.00
genus

1
26
1100915

 0.00
species

species

 0.00
1100916
44
1


 0.00
genus
43
1
2
572852
0

43
1
1869985

 0.00
species


 0.00
genus
1
47
2
0
581658


 0.00
species
1
47
1100989

581385
0
7
1
203
tribe

 0.01


 0.00
genus
4
1
144
17
29054


 0.00
species
1
42
989769

42
1
1594294

 0.00
species

43
1
1594293

 0.00
species


 0.00
genus
59
1
2
0
581513

species

 0.00
1594315
1
59

10
1
177
6
581387

 0.00
tribe

1
86
3
0
581588

 0.00
genus

1101027
42
1
species

 0.00

1870148
1
44
species

 0.00

82599
0
1
19
2
genus

 0.00

1100963
1
19
species

 0.00

0
581577
2
1
8
genus

 0.00

8
1
1035111

 0.00
species

2
1
58
0
293340

 0.00
genus

192188
1
58
species

 0.00


 0.00
subfamily
168
1
9
1
65022

tribe

 0.00
0
173709
5
128
1

genus

 0.00
572799
4
4
1
128

1100899
1
38
species

 0.00

46
1
758717

 0.00
species

40
1
758706

 0.00
species

0
81689
3
39
1
tribe

 0.00

39
1
2
0
192190

 0.00
genus


 0.00
species
39
1
753214

superfamily

 0.01
0
37582
1
221
12

4
40
1
0
33464

 0.00
family

397427
0
3
40
1
subfamily

 0.00

genus

 0.00
33465
0
40
1
2


 0.00
species
1
40
263933

4
158
1
687156
0

 0.00
family

genus

 0.00
1
687147
1
158
3

78
1
1870436

 0.00
species

1
79
1870435

 0.00
species


 0.00
family
23
1
3
0
51653

51654
0
1
23
2
genus

 0.00


 0.00
species
23
1
51655

0
41011
1
85
6
superfamily

 0.00


 0.00
family
5
1
85
0
41012

subfamily

 0.00
0
236781
85
1
4


 0.00
genus
85
1
3
0
41013


 0.00
species
1
40
1594354

1101063
1
45
species

 0.00

superfamily

 0.01
1
37581
1
330
21

0
57992
4
58
1
family

 0.00

116123
0
3
58
1
subfamily

 0.00

2
58
1
688988
0

 0.00
genus

species

 0.00
2870497
58
1

family

 0.00
0
173647
1
1
4

3
1
1
0
248746

 0.00
subfamily

1
1
2
0
545366

 0.00
genus

1477025
1
1
species

 0.00

1
120
4
0
173649

 0.00
family

3
1
120
35
655692

 0.00
genus


 0.00
species
41
1
1869501


 0.00
species
44
1
2561016

1
150
8
2681869
0

 0.00
family

78
1
4
0
116119

 0.00
subfamily


 0.00
genus
3
1
78
6
262437

1594222
45
1
species

 0.00

species

 0.00
1857958
27
1

no rank

 0.00
2925401
0
3
72
1

1
72
2
0
116120

 0.00
genus

1
72
116121

 0.00
species

clade

 0.47
6983
104431
18826
1
469

superfamily

 0.13
37570
779
1
5445
179

family

 0.00
0
319783
78
1
7

subfamily

 0.00
753688
0
3
44
1

2
44
1
572722
0

 0.00
genus

988004
1
44
species

 0.00

0
95245
3
34
1
subfamily

 0.00

56393
0
2
34
1
genus

 0.00

species

 0.00
987977
34
1

1
281
20
37571
2

 0.01
family

319762
0
36
1
3
subfamily

 0.00

2
36
1
0
214089

 0.00
genus

987902
36
1
species

 0.00

0
319773
69
1
3
subfamily

 0.00

2
69
1
13633
0

 0.00
genus

species

 0.00
753216
1
69

0
319766
41
1
3
subfamily

 0.00

56587
0
2
41
1
genus

 0.00

987943
1
41
species

 0.00


 0.00
subfamily
1
101
7
1
319765


 0.00
genus
3
52
1
2
214280

21
1
988002

 0.00
species

753204
29
1
species

 0.00


 0.00
genus
48
1
3
4
214308

988018
1
21
species

 0.00

988019
23
1
species

 0.00


 0.00
subfamily
32
1
3
0
319770

0
987448
2
1
32
genus

 0.00

species

 0.00
987449
32
1

3204
1
112
498
7100

 0.08
family


 0.00
subfamily
1
49
3
1430885
0


 0.00
genus
1
49
2
708062
0

49
1
708063

 0.00
species

1
267
13
7
95186

 0.01
subfamily

genus

 0.00
1
254364
3
56
1

254365
1
23
species

 0.00


 0.00
species
1
32
938171

genus

 0.00
3
254361
3
1
79

1
46
987893

 0.00
species

species

 0.00
254363
30
1

0
254717
1
64
2
genus

 0.00


 0.00
species
64
1
689277

179673
0
2
1
58
genus

 0.00

179674
58
1
species

 0.00

0
7110
2
1
3
genus

 0.00

species

 0.00
7111
1
3

13
1
308
95182
7

 0.01
subfamily

0
988055
1
41
2
genus

 0.00

species

 0.00
988056
1
41

genus

 0.00
0
1430964
1
65
2

species

 0.00
1870430
65
1


 0.00
genus
106
1
5
3
7106

56
1
7107

 0.00
species

1
1
69820

 0.00
species

7109
1
39
species

 0.00


 0.00
species
7
1
7108

1
56365
3
1
89
genus

 0.00

1
45
689058

 0.00
species

987877
1
43
species

 0.00

95179
16
28
645
1
subfamily

 0.02

tribe

 0.01
2555566
16
15
1
404


 0.00
genus
34
1
2
47766
0

1857961
34
1
species

 0.00

genus

 0.00
4
214276
4
131
1

1
41
753202

 0.00
species


 0.00
species
1
42
987995


 0.00
species
1
44
214277

4
150
1
1
320016

 0.00
genus

1
45
988049

 0.00
species


 0.00
species
60
1
987431


 0.00
species
44
1
997545


 0.00
genus
1
35
2
0
320033

1
35
987925

 0.00
species

214282
0
2
1
38
genus

 0.00

38
1
320037

 0.00
species

0
320076
2
1
36
genus

 0.00


 0.00
species
1
36
987895


 0.00
tribe
1
189
10
2555556
3

882791
0
29
1
2
genus

 0.00

species

 0.00
988174
29
1

1
61
2
0
688395

 0.00
genus

species

 0.00
987876
61
1


 0.00
genus
2
30
1
0
214170


 0.00
species
30
1
214171

1
66
3
95189
3

 0.00
genus


 0.00
species
1
31
689061


 0.00
species
32
1
875885

17
502
1
95244
25

 0.01
subfamily


 0.00
genus
2
1
55
320089
0

1
55
875884

 0.00
species

genus

 0.00
0
946290
2
91
1


 0.00
species
1
91
988041

genus

 0.00
103830
12
4
138
1

39
1
987983

 0.00
species

987985
1
53
species

 0.00


 0.00
species
34
1
997540


 0.00
genus
40
1
2
0
55056

55057
40
1
species

 0.00

988059
0
1
70
2
genus

 0.00

70
1
988060

 0.00
species

2
1
28
988123
0

 0.00
genus

988125
28
1
species

 0.00

320087
0
55
1
2
genus

 0.00

1
55
987933

 0.00
species

546
1
18
29
572922

 0.01
subfamily

2
1
41
997550
0

 0.00
genus

997551
41
1
species

 0.00

1
689302
3
74
1
genus

 0.00


 0.00
species
1
39
987872


 0.00
species
1
34
987866

130
1
2
988105
0

 0.00
genus

1
130
988106

 0.00
species

1
56
2
0
2492374

 0.00
genus

2492375
56
1
species

 0.00


 0.00
genus
2
70
1
988070
0


 0.00
species
1
70
988071

genus

 0.00
0
988063
52
1
2


 0.00
species
52
1
1337163

1101105
0
1
41
2
genus

 0.00

species

 0.00
1101106
41
1

53
1
2
988080
0

 0.00
genus

species

 0.00
988081
1
53

144
1
6
95175
15

 0.00
subfamily

genus

 0.00
56362
0
3
78
1


 0.00
species
1
35
987859

43
1
987865

 0.00
species

genus

 0.00
938225
0
2
1
51


 0.00
species
1
51
938226

subfamily

 0.00
116124
1
6
168
1

56
1
2
116129
0

 0.00
genus

species

 0.00
116130
1
56


 0.00
genus
1
111
3
116125
9


 0.00
species
1
52
987909

50
1
116126

 0.00
species

43
1
3
95214
0

 0.00
subfamily


 0.00
genus
1
43
2
0
753440


 0.00
species
1
43
753441

1
34
4
0
95178

 0.00
subfamily

genus

 0.00
1
7112
3
1
34

species

 0.00
29058
2
1

31
1
7113

 0.00
species

1103
1
39
695564
55

 0.03
family

95222
0
3
50
1
subfamily

 0.00


 0.00
genus
2
1
50
705937
0


 0.00
species
50
1
987440

1
392
16
5
30225

 0.01
subfamily

10
1945703
1
165
7
tribe

 0.00


 0.00
genus
2
1
51
0
695183

875881
51
1
species

 0.00

genus

 0.00
0
214365
2
1
56

56
1
875880

 0.00
species

2
1
48
214310
0

 0.00
genus

214311
48
1
species

 0.00

9
132199
222
1
8
tribe

 0.01


 0.00
genus
80
1
3
2
464722

987424
1
30
species

 0.00


 0.00
species
1
48
987419

2
59
1
0
694848

 0.00
genus

1
59
987918

 0.00
species

0
694657
2
1
74
genus

 0.00

987980
1
74
species

 0.00

1583079
4
7
1
244
subfamily

 0.01

0
938237
56
1
2
genus

 0.00


 0.00
species
1
56
938238

292570
0
2
1
113
genus

 0.00

113
1
938167

 0.00
species

genus

 0.00
0
56375
71
1
2

species

 0.00
423510
1
71

95217
1
126
1
5
subfamily

 0.00

2
49
1
0
95218

 0.00
genus

species

 0.00
753189
49
1

genus

 0.00
0
411962
76
1
2

species

 0.00
411963
1
76


 0.01
subfamily
1
236
7
4
27548


 0.00
genus
2
114
1
0
13122

species

 0.00
78897
1
114


 0.00
genus
59
1
2
0
33413

335469
59
1
species

 0.00

genus

 0.00
0
319798
2
59
1

1
59
987935

 0.00
species

118
3003
1
37572
272

 0.07
superfamily

3
27544
404
1
21
family

 0.01

124406
0
43
1
3
subfamily

 0.00

2
43
1
0
265359

 0.00
genus

282391
43
1
species

 0.00

1
358
17
42297
28

 0.01
subfamily

genus

 0.00
0
1821620
2
1
41

988025
41
1
species

 0.00

genus

 0.00
265382
0
2
1
33


 0.00
species
33
1
265386

138069
21
49
1
3
genus

 0.00

1
16
138070

 0.00
species

1
12
268709

 0.00
species

1
50
2
0
203780

 0.00
genus

1
50
203781

 0.00
species


 0.00
genus
2
67
1
42298
0

203782
67
1
species

 0.00

242266
0
1
42
2
genus

 0.00

242267
1
42
species

 0.00


 0.00
genus
48
1
3
91737
18


 0.00
species
22
1
91739

8
1
91738

 0.00
species

family

 0.05
148
33415
69
1
1848


 0.01
subfamily
1
548
19
40040
11

2
5
1
311092
0

 0.00
tribe

genus

 0.00
39707
5
1

tribe

 0.00
171578
0
6
1
187


 0.00
subtribe
5
187
1
171580
7

genus

 0.00
0
104514
100
1
2

113334
100
1
species

 0.00

0
596672
2
1
80
genus

 0.00

species

 0.00
113330
80
1

14
171576
10
1
345
tribe

 0.01

genus

 0.00
3
42274
3
1
145

species

 0.00
42275
63
1

79
1
171605

 0.00
species

genus

 0.00
76218
12
6
1
186

subgenus

 0.00
17
111880
3
1
97


 0.00
species
1
36
111881

44
1
171585

 0.00
species

0
442324
2
77
1
subgenus

 0.00

77
1
171594

 0.00
species

98
1
4
100750
0

 0.00
subfamily

215788
0
98
1
3
tribe

 0.00

genus

 0.00
124410
0
2
98
1


 0.00
species
1
98
270466

subfamily

 0.01
40037
0
11
262
1

0
127322
1
61
3
tribe

 0.00


 0.00
genus
2
61
1
33416
0

species

 0.00
33443
1
61

tribe

 0.00
10
127312
1
201
7

genus

 0.00
127313
0
42
1
2

191398
42
1
species

 0.00

1
103
2
405031
0

 0.00
genus


 0.00
species
1
103
405034

genus

 0.00
525812
0
2
46
1


 0.00
species
1
46
405009


 0.00
subfamily
1
200
8
0
127218

tribe

 0.00
0
42315
7
1
200

0
344711
6
200
1
subtribe

 0.00


 0.00
genus
5
200
1
40
64444

1
73
2
304554
0

 0.00
species

331302
1
73
subspecies

 0.00

2
87
1
331299
0

 0.00
species

87
1
331333

 0.00
subspecies

1
592
26
42282
5

 0.01
subfamily

tribe

 0.00
1664845
0
60
1
4


 0.00
genus
3
60
1
4
111897

species

 0.00
447833
1
29

species

 0.00
111903
27
1

127320
13
1
527
21
tribe

 0.01

subtribe

 0.00
366209
2
5
97
1

genus

 0.00
111932
0
46
1
2

116150
46
1
species

 0.00

111915
0
49
1
2
genus

 0.00

111917
1
49
species

 0.00

subtribe

 0.00
167180
0
1
96
3

0
110367
2
1
96
genus

 0.00


 0.00
species
1
96
110368

150883
0
1
194
6
subtribe

 0.00


 0.00
genus
5
1
194
111919
1

111950
0
2
154
1
no rank

 0.00

species

 0.00
191418
1
154


 0.00
subgenus
1
39
2
0
111885

39
1
2795564

 0.00
species

150884
0
99
1
3
subtribe

 0.00

111922
0
2
99
1
genus

 0.00

99
1
111923

 0.00
species

1
28
3
150886
0

 0.00
subtribe

0
111908
2
1
28
genus

 0.00

111912
1
28
species

 0.00

406
1
17
2
7114

 0.01
family


 0.00
subfamily
3
1
60
0
42450


 0.00
genus
2
1
60
0
42295


 0.00
species
60
1
72248

subfamily

 0.00
151208
0
80
1
3

0
189907
2
1
80
genus

 0.00

1
80
189913

 0.00
species


 0.01
subfamily
10
1
264
0
42449

5
152601
9
264
1
tribe

 0.01

genus

 0.00
7115
8
160
1
4

7116
1
26
species

 0.00

64459
52
1
species

 0.00


 0.00
species
74
1
78633

2
1
48
0
129396

 0.00
genus

129397
1
48
species

 0.00


 0.00
genus
2
1
51
72244
0


 0.00
species
51
1
227532

10
1
73
7143
0

 0.00
family


 0.00
subfamily
9
73
1
0
42289

26
1
3
0
189314

 0.00
tribe


 0.00
genus
2
1
26
157396
0

species

 0.00
110791
1
26

5
1
47
189315
0

 0.00
tribe

7145
0
47
1
4
genus

 0.00

2
1
76194

 0.00
species

76193
37
1
species

 0.00


 0.00
species
8
1
66420

superfamily

 0.00
0
104423
136
1
12

family

 0.00
104425
0
136
1
11

2
104465
5
69
1
subfamily

 0.00


 0.00
genus
2
1
25
0
721164

species

 0.00
721165
1
25

genus

 0.00
0
104426
2
42
1

1
42
104428

 0.00
species


 0.00
subfamily
1
67
5
219490
1

0
219491
1
36
2
genus

 0.00

1
36
721137

 0.00
species

721162
0
2
1
30
genus

 0.00

species

 0.00
721163
30
1


 0.00
superfamily
1
196
9
0
104493

family

 0.00
0
186111
1
196
8

1
186112
196
1
7
subfamily

 0.00

467773
0
34
1
2
genus

 0.00


 0.00
species
1
34
467774

81
1
2
0
1594452

 0.00
genus

1
81
1594453

 0.00
species

genus

 0.00
753424
0
80
1
2

species

 0.00
1660692
80
1


 0.01
superfamily
18
326
1
40092
0

40093
5
1
326
17
family

 0.01

1
159
8
0
40096

 0.00
subfamily


 0.00
tribe
7
1
159
1
2839394

genus

 0.00
218770
0
51
1
2

species

 0.00
272628
51
1

2
34
1
218734
0

 0.00
genus

34
1
291688

 0.00
species


 0.00
genus
1
73
2
218743
0

73
1
876063

 0.00
species

5
1
111
40100
0

 0.00
subfamily


 0.00
genus
71
1
2
0
40102


 0.00
species
1
71
218760

2
40
1
0
76212

 0.00
genus

1
40
520884

 0.00
species

3
51
1
218718
0

 0.00
subfamily


 0.00
genus
51
1
2
218719
0

218720
51
1
species

 0.00

superfamily

 0.01
37569
2
258
1
23

family

 0.00
7089
0
4
1
47


 0.00
subfamily
3
47
1
475327
0

4
7090
2
47
1
genus

 0.00


 0.00
species
1
43
7091


 0.01
family
209
1
18
7128
3

82617
3
1
111
7
subfamily

 0.00

1
60
3
523176
0

 0.00
tribe

genus

 0.00
283833
0
1
60
2


 0.00
species
60
1
644661

3
48
1
523174
0

 0.00
tribe


 0.00
genus
2
48
1
0
82622


 0.00
species
1
48
987953

0
469321
93
1
6
subfamily

 0.00


 0.00
tribe
93
1
5
523180
0


 0.00
genus
1
39
2
522847
0

species

 0.00
522848
1
39


 0.00
genus
54
1
2
522835
0


 0.00
species
1
54
522836

82616
0
4
2
1
subfamily

 0.00


 0.00
tribe
1
2
3
0
523164

genus

 0.00
0
7129
2
2
1

7130
1
2
species

 0.00

37573
19
38
1
839
superfamily

 0.02


 0.01
family
16
265
1
7135
2

subfamily

 0.00
299347
0
148
1
5

genus

 0.00
0
1101109
1
97
2

1
97
1101110

 0.00
species

51
1
2
0
1101094

 0.00
genus

51
1
1101095

 0.00
species

7
114
1
2
40083

 0.00
subfamily

genus

 0.00
680682
0
2
1
2

680683
1
2
species

 0.00


 0.00
genus
2
1
35
0
989881


 0.00
species
35
1
1666458


 0.00
genus
2
75
1
687068
0


 0.00
species
1
75
1857951

3
1
1
40082
0

 0.00
subfamily


 0.00
genus
2
1
1
7136
0

species

 0.00
7137
1
1

family

 0.01
12
268499
21
555
1

3
1
3
0
40080

 0.00
subfamily

genus

 0.00
29056
0
2
3
1


 0.00
species
3
1
93504


 0.01
subfamily
428
1
12
40081
16

genus

 0.00
0
1368975
98
1
2

1
98
1371681

 0.00
species

genus

 0.00
572825
0
1
50
2

1594250
50
1
species

 0.00

genus

 0.00
168630
0
1
86
2

168631
86
1
species

 0.00

572808
5
3
1
93
genus

 0.00

1
37
1660579

 0.00
species

1594226
51
1
species

 0.00

genus

 0.00
0
40084
85
1
2

species

 0.00
40085
1
85

299362
0
5
112
1
subfamily

 0.00

2
1
55
0
687116

 0.00
genus


 0.00
species
1
55
1594321

genus

 0.00
0
1666817
1
57
2


 0.00
species
1
57
1666818

1640
1
71
82592
0

 0.04
superfamily


 0.04
family
1
1640
70
82593
103

subfamily

 0.00
0
104442
1
34
3

genus

 0.00
0
104446
34
1
2

species

 0.00
104447
1
34

subfamily

 0.02
29
104450
31
681
1


 0.00
genus
21
1
2
0
214132

species

 0.00
934839
21
1

2
54
1
934939
0

 0.00
genus

54
1
934940

 0.00
species

934935
0
2
19
1
genus

 0.00


 0.00
species
1
19
934936

0
873511
62
1
2
genus

 0.00

62
1
934888

 0.00
species

genus

 0.00
0
104456
2
1
21

104457
21
1
species

 0.00


 0.00
genus
36
1
2
326956
0

987013
1
36
species

 0.00

214137
9
6
1
194
genus

 0.00

934844
1
42
species

 0.00

1
26
934840

 0.00
species

934845
1
21
species

 0.00

62
1
934847

 0.00
species


 0.00
species
34
1
934866

1
44
2
934916
0

 0.00
genus

species

 0.00
934917
1
44

2
56
1
104451
0

 0.00
genus


 0.00
species
1
56
104452

0
214392
1
41
2
genus

 0.00

934828
1
41
species

 0.00

2
1
45
104458
0

 0.00
genus

104460
45
1
species

 0.00

1
35
2
0
214189

 0.00
genus

1
35
934876

 0.00
species

24
1
2
190368
0

 0.00
genus


 0.00
species
1
24
934904


 0.00
subfamily
3
40
1
104461
0

934941
0
2
40
1
genus

 0.00


 0.00
species
40
1
934942

subfamily

 0.02
28
82596
29
1
734

genus

 0.00
104473
0
2
48
1

104474
48
1
species

 0.00


 0.00
genus
58
1
2
704816
0


 0.00
species
58
1
934882

41
1
2
0
82594

 0.00
genus


 0.00
species
1
41
82595

704699
0
2
30
1
genus

 0.00

species

 0.00
934829
1
30

1
18
2
0
722672

 0.00
genus

species

 0.00
722673
1
18


 0.00
genus
2
1
61
934914
0


 0.00
species
61
1
934915

2
1
36
82597
0

 0.00
genus

1
36
934813

 0.00
species

genus

 0.00
692034
0
1
47
2

934875
47
1
species

 0.00

0
214128
1
61
3
genus

 0.00


 0.00
species
33
1
722662

species

 0.00
875883
28
1

0
393392
1
34
2
genus

 0.00


 0.00
species
1
34
688445

genus

 0.00
0
572919
1
110
2

934894
1
110
species

 0.00

3
1
118
3
104475

 0.00
genus

61
1
190331

 0.00
species

species

 0.00
104476
1
54


 0.00
genus
1
44
2
0
190355

species

 0.00
190356
1
44


 0.00
subfamily
3
1
48
0
393382


 0.00
genus
48
1
2
0
104485

104486
1
48
species

 0.00

0
85817
1
49
15
superorder

 0.00

50553
0
1
1
5
order

 0.00

4
1
1
50554
0

 0.00
family

85815
0
3
1
1
subfamily

 0.00

genus

 0.00
1569766
0
1
1
2

1
1
2027374

 0.00
species

7516
0
9
1
48
order

 0.00

suborder

 0.00
2029065
0
8
1
48

7520
0
7
1
48
family

 0.00

0
2029107
3
1
1
subfamily

 0.00

2
1
1
0
373838

 0.00
genus


 0.00
species
1
1
2028661

subfamily

 0.00
0
2029106
47
1
3

0
7521
47
1
2
genus

 0.00

species

 0.00
189513
1
47

1574
1
178
7041
51

 0.04
order

suborder

 0.01
41071
0
20
382
1


 0.01
superfamily
19
382
1
535382
0

18
1
382
41073
4

 0.01
family

0
71541
8
1
160
subfamily

 0.00

tribe

 0.00
879230
0
4
76
1

genus

 0.00
41078
0
3
1
76


 0.00
subgenus
1
76
2
0
484232

1
76
767470

 0.00
species

0
60759
84
1
3
tribe

 0.00

0
247414
2
84
1
genus

 0.00

species

 0.00
247415
84
1

subfamily

 0.01
0
71539
9
218
1

0
60833
1
218
8
tribe

 0.01

2
1
44
60836
0

 0.00
genus

878056
44
1
species

 0.00

1
174
5
60834
10

 0.00
genus


 0.00
subgenus
2
1
155
186524
0

species

 0.00
878211
155
1


 0.00
subgenus
9
1
2
186517
0

1
9
110024

 0.00
species


 0.03
suborder
157
1141
1
49
41084

33
41088
99
1
754
infraorder

 0.02

13
1
112
0
2939015

 0.00
superfamily

family

 0.00
0
7080
12
1
112

1
7081
112
1
11
subfamily

 0.00

0
263631
7
95
1
tribe

 0.00

genus

 0.00
41138
0
2
30
1


 0.00
species
1
30
41139


 0.00
genus
2
37
1
0
115356

1
37
115357

 0.00
species


 0.00
genus
2
1
28
0
7083


 0.00
species
1
28
7084

3
16
1
0
263632

 0.00
tribe


 0.00
genus
2
16
1
347358
0

347359
1
16
species

 0.00

5
64
1
71525
0

 0.00
superfamily

family

 0.00
0
186093
1
64
4

1
64
3
0
353826

 0.00
subfamily

1
64
2
0
295699

 0.00
genus


 0.00
species
1
64
295700


 0.00
superfamily
62
1
9
71527
0

family

 0.00
0
7065
1
30
5

1
30
4
0
1304792

 0.00
no rank

30
1
3
2
7069

 0.00
genus


 0.00
species
1
1
7070

species

 0.00
7072
27
1

3
1
32
0
55098

 0.00
family


 0.00
genus
2
1
32
0
295984

1
32
346838

 0.00
species

8
1
51
0
71526

 0.00
superfamily

family

 0.00
0
116151
7
1
51

3
48
1
0
577241

 0.00
subfamily

genus

 0.00
1431902
0
1
48
2


 0.00
species
1
48
1431903

1
3
3
577242
0

 0.00
subfamily

2
1
3
116152
0

 0.00
genus

species

 0.00
116153
3
1

superfamily

 0.01
71528
0
37
321
1

5
3
1
34667
0

 0.00
family


 0.00
subfamily
4
1
3
0
79514


 0.00
tribe
3
1
3
192383
0


 0.00
genus
1
3
2
157304
0

species

 0.00
217634
1
3

318
1
31
6
27439

 0.01
family

subfamily

 0.00
0
131688
56
1
3

genus

 0.00
204943
0
56
1
2

204949
56
1
species

 0.00

subfamily

 0.00
63707
0
9
117
1

63712
0
1
4
3
tribe

 0.00

genus

 0.00
7538
0
4
1
2


 0.00
species
4
1
7539


 0.00
tribe
5
1
113
1
63708

80248
0
1
51
2
genus

 0.00

1
51
80249

 0.00
species

0
41125
1
61
2
genus

 0.00

1587174
1
61
species

 0.00

subfamily

 0.00
63710
7
1
139
18

4
1
1
200827
0

 0.00
tribe

3
1
1
0
226725

 0.00
clade

1
1
2
0
226615

 0.00
genus

226666
1
1
species

 0.00

0
131578
6
116
1
tribe

 0.00


 0.00
genus
28
1
3
224132
1

15
1
224133

 0.00
species


 0.00
species
1
12
444603

genus

 0.00
294691
0
1
88
2

88
1
2598218

 0.00
species

0
63711
7
1
15
tribe

 0.00


 0.00
subtribe
15
1
6
0
226742


 0.00
no rank
5
15
1
0
226749


 0.00
genus
4
1
15
2
50385

107213
1
6
species

 0.00

species

 0.00
0
50389
2
1
7

subspecies

 0.00
50390
7
1

26
1
111
0
71529

 0.00
superfamily

4
33
1
122737
0

 0.00
family

33
1
3
701798
0

 0.00
subfamily

0
122772
2
33
1
genus

 0.00


 0.00
species
1
33
201766


 0.00
family
21
78
1
1
7042


 0.00
subfamily
3
27
1
0
123516

genus

 0.00
0
201855
2
1
27


 0.00
species
27
1
467358

subfamily

 0.00
0
39812
3
8
1


 0.00
genus
8
1
2
0
7045

8
1
7048

 0.00
species

subfamily

 0.00
54544
0
1
1
5

tribe

 0.00
0
365782
4
1
1

genus

 0.00
0
1482260
1
1
3

1
1
2
2633171
0

 0.00
no rank

1
1
1682456

 0.00
species

0
39816
1
3
5
subfamily

 0.00


 0.00
tribe
4
1
3
0
123517

3
1
3
0
7043

 0.00
genus

0
7044
3
1
2
species

 0.00

3
1
2921223

 0.00
subspecies

subfamily

 0.00
39814
0
38
1
4

tribe

 0.00
465383
0
3
38
1


 0.00
genus
1
38
2
0
122852

202137
38
1
species

 0.00


 0.00
infraorder
1
8
13
0
41086


 0.00
superfamily
12
8
1
0
75546

0
7055
8
1
11
family

 0.00

7
6
1
41142
0

 0.00
subfamily

569643
0
1
4
3
no rank

 0.00

1
4
2
0
166331

 0.00
genus

species

 0.00
166361
4
1

569639
0
2
1
3
tribe

 0.00


 0.00
genus
2
1
2
0
166327

species

 0.00
166328
1
2


 0.00
subfamily
3
2
1
0
7059

2
2
1
7060
0

 0.00
genus

2
1
7061

 0.00
species


 0.00
infraorder
27
1
144
41087
0

5
1
3
71192
0

 0.00
superfamily

1
3
4
0
50527

 0.00
family

subfamily

 0.00
261156
0
3
1
3

0
195164
3
1
2
genus

 0.00

1
3
224129

 0.00
species

141
1
21
0
71193

 0.00
superfamily


 0.00
family
5
4
1
7049
0

1
1
433515

 0.00
subfamily

0
433514
1
3
3
subfamily

 0.00

genus

 0.00
7053
0
3
1
2


 0.00
species
3
1
7054

1
26
4
0
30009

 0.00
family


 0.00
subfamily
1
26
3
116139
0

genus

 0.00
0
292457
2
1
26

292458
26
1
species

 0.00

family

 0.00
0
41097
8
110
1

subfamily

 0.00
5
433502
110
1
7


 0.00
genus
1
48
2
41098
0

48
1
195172

 0.00
species


 0.00
genus
2
1
26
0
186072

species

 0.00
1553677
26
1

2
31
1
41100
0

 0.00
genus

1
31
41101

 0.00
species


 0.00
family
1
1
3
71195
0

1
1
2
2043422
0

 0.00
subfamily


 0.00
genus
1
1
343838


 0.00
infraorder
1
186
17
0
41085


 0.00
superfamily
16
1
186
75543
1

8
114
1
0
29026

 0.00
family

1
114
7
351514
0

 0.00
no rank


 0.00
subfamily
6
1
114
0
82886

295648
2
114
1
5
tribe

 0.00

2
71
1
290671
0

 0.00
genus

species

 0.00
662956
1
71

2
41
1
219450
0

 0.00
genus

1
41
346820

 0.00
species

family

 0.00
0
57514
71
1
7

subfamily

 0.00
82881
0
3
1
67

genus

 0.00
414933
0
1
67
2

414934
1
67
species

 0.00

0
82882
3
4
1
subfamily

 0.00

genus

 0.00
57515
0
2
4
1

110193
4
1
species

 0.00

334
2203
1
29
7399

 0.05
order

85772
0
1
154
20
superfamily

 0.00

27532
0
7
1
136
family

 0.00

subfamily

 0.00
112287
2
6
136
1

genus

 0.00
112291
1
119
1
3

1
41
362091

 0.00
species


 0.00
species
77
1
222778

genus

 0.00
0
1250642
15
1
2

1
15
1385029

 0.00
species

2982298
0
3
10
1
family

 0.00

genus

 0.00
0
37343
2
1
10

1
10
37344

 0.00
species

family

 0.00
0
52632
6
1
7

0
410274
1
7
5
subfamily

 0.00

6
1
2
2
270857

 0.00
genus


 0.00
species
1
4
2961670

0
52633
2
1
1
genus

 0.00


 0.00
species
1
1
362088


 0.00
family
3
1
1
85774
0

genus

 0.00
0
222776
1
1
2

1
1
2950364

 0.00
species

0
222823
1
1
4
superfamily

 0.00

0
27524
1
1
3
family

 0.00

0
173784
1
1
2
genus

 0.00

1
1
211228

 0.00
species

3
1
4
0
222831

 0.00
superfamily

0
27528
3
1
3
family

 0.00


 0.00
genus
2
1
3
27529
0

species

 0.00
222816
3
1

2016
1
305
7400
57

 0.05
suborder

1955251
1
79
242
1
infraorder

 0.01

superfamily

 0.01
7401
1
50
1
204

7408
2
1
157
29
family

 0.00

0
65170
6
1
17
subfamily

 0.00


 0.00
tribe
17
1
5
172511
0


 0.00
genus
2
13
1
0
27520

2795680
1
13
species

 0.00

genus

 0.00
0
231886
2
1
4

species

 0.00
231887
4
1

0
65140
4
1
33
subfamily

 0.00

288186
0
1
33
3
no rank

 0.00

33
1
2
0
29048

 0.00
genus

1
33
1539398

 0.00
species

3
1
4
65149
0

 0.00
subfamily

0
273877
3
3
1
no rank

 0.00


 0.00
genus
2
3
1
0
92443

32260
3
1
species

 0.00

subfamily

 0.00
0
65167
1
26
5

231867
0
1
26
4
tribe

 0.00

65288
0
3
26
1
genus

 0.00

subgenus

 0.00
0
494744
26
1
2

2866289
1
26
species

 0.00

65171
0
25
1
5
subfamily

 0.00


 0.00
tribe
4
1
25
0
172377

subtribe

 0.00
231877
0
3
1
25

1
25
2
0
231878

 0.00
genus


 0.00
species
1
25
1419289

4
51
1
0
65163

 0.00
subfamily

tribe

 0.00
176302
0
3
51
1


 0.00
genus
1
51
2
493657
0

2870495
1
51
species

 0.00

family

 0.00
0
7402
20
1
46


 0.00
subfamily
3
1
1
0
65210

0
7405
2
1
1
genus

 0.00

species

 0.00
460826
1
1


 0.00
subfamily
3
1
18
0
65197

0
144405
1
18
2
genus

 0.00

2738948
1
18
species

 0.00


 0.00
subfamily
11
1
7
0
65207

genus

 0.00
51538
0
8
1
2


 0.00
species
8
1
69319

genus

 0.00
32390
0
1
2
2

2
1
32391

 0.00
species

genus

 0.00
0
169869
1
1
2


 0.00
species
1
1
1911502

3
1
1
0
68883

 0.00
subfamily

2
1
1
0
454922

 0.00
genus

1
1
454923

 0.00
species

1
15
3
68882
0

 0.00
subfamily

genus

 0.00
0
37852
15
1
2

species

 0.00
684658
1
15

superfamily

 0.00
0
40307
11
10
1

1
9
5
44353
0

 0.00
family

0
303412
1
9
4
subfamily

 0.00

2
63429
9
1
3
genus

 0.00

species

 0.00
63433
4
1

species

 0.00
63436
1
3

0
73401
5
1
1
family

 0.00

0
1159319
1
1
4
subfamily

 0.00

75144
0
3
1
1
tribe

 0.00

1
1
2
0
1159320

 0.00
genus


 0.00
species
1
1
2817044

17
27
1
0
7422

 0.00
superfamily

family

 0.00
0
75187
5
1
9

75190
0
9
1
4
subfamily

 0.00

9
1
3
0
84507

 0.00
genus

3
142686
2
9
1
species

 0.00


 0.00
subspecies
1
6
326594

3
5
1
0
7489

 0.00
family

5
1
2
0
7490

 0.00
genus

7493
1
5
species

 0.00

2
1
4
0
29051

 0.00
family

subfamily

 0.00
0
272199
1
2
3


 0.00
genus
2
2
1
29052
0

29053
1
2
species

 0.00

1
11
4
7423
0

 0.00
family

3
1
11
0
272242

 0.00
subfamily

genus

 0.00
7424
0
11
1
2

species

 0.00
7425
1
11

76
7434
1717
1
225
infraorder

 0.04


 0.00
superfamily
17
1
5
0
2153482

17
1
4
92421
0

 0.00
family

subfamily

 0.00
219387
0
3
17
1

17
1
2
0
219389

 0.00
genus


 0.00
species
17
1
330862


 0.01
superfamily
1
338
22
0
34725

1
338
21
8
7438

 0.01
family


 0.00
subfamily
25
1
3
0
50638

genus

 0.00
0
76989
2
1
25

76990
25
1
species

 0.00

5
17
1
0
7455

 0.00
subfamily


 0.00
tribe
17
1
4
0
76984

6
7456
3
17
1
genus

 0.00

species

 0.00
743375
7
1

species

 0.00
91411
1
4

subfamily

 0.01
11
7439
12
288
1

7440
12
4
1
84
genus

 0.00


 0.00
species
1
22
85443

species

 0.00
881891
1
23


 0.00
species
27
1
85444

7451
21
106
1
3
genus

 0.00

7454
21
1
species

 0.00

30212
1
64
species

 0.00


 0.00
genus
87
1
4
7443
13

species

 0.00
7445
32
1

species

 0.00
7446
1
1

41
1
202808

 0.00
species


 0.00
superfamily
66
201
1
2153479
0

65
1
201
36668
0

 0.00
family

subfamily

 0.00
4
34695
34
147
1

tribe

 0.00
0
144003
1
1
3

genus

 0.00
0
369112
2
1
1

1
1
606695

 0.00
species

144020
0
3
1
112
tribe

 0.00

2
1
112
0
30204

 0.00
genus

species

 0.00
219812
112
1

tribe

 0.00
144001
0
1
1
3

2
1
1
0
64792

 0.00
genus

64793
1
1
species

 0.00


 0.00
tribe
5
4
1
0
1932955

3
1
2
144030
0

 0.00
genus

species

 0.00
144034
3
1

genus

 0.00
604392
0
2
1
1


 0.00
species
1
1
610408


 0.00
tribe
13
1
5
144017
0

genus

 0.00
0
55077
1
7
2


 0.00
species
7
1
307658

genus

 0.00
13685
0
2
6
1

13686
6
1
species

 0.00

8
1
6
0
143999

 0.00
tribe

12956
0
1
2
3
genus

 0.00

1
1
520822

 0.00
species

12957
1
1
species

 0.00


 0.00
genus
1
3
2
34699
0

456900
3
1
species

 0.00

genus

 0.00
34717
0
1
1
2

1
1
471704

 0.00
species

3
1
1
144004
0

 0.00
tribe

300110
0
1
1
2
genus

 0.00


 0.00
species
1
1
300111


 0.00
tribe
5
1
3
144019
0

0
369248
1
5
2
genus

 0.00


 0.00
species
5
1
411798

213859
0
3
9
1
subfamily

 0.00

genus

 0.00
0
2015172
2
1
9


 0.00
species
1
9
2015173

0
40138
4
1
5
subfamily

 0.00

genus

 0.00
56621
0
5
1
3

219809
1
2
species

 0.00


 0.00
species
1
3
1929244

8
16
1
0
43085

 0.00
subfamily

0
141711
1
16
7
tribe

 0.00


 0.00
genus
8
1
2
0
369122

609295
1
8
species

 0.00

0
43086
3
1
2
genus

 0.00

1
3
486640

 0.00
species

genus

 0.00
0
604375
2
1
5

5
1
610380

 0.00
species

subfamily

 0.00
7479
0
1
19
12


 0.00
tribe
1
4
3
0
72772


 0.00
genus
2
1
4
710235
0


 0.00
species
4
1
613905

0
72773
3
7
1
tribe

 0.00


 0.00
genus
1
7
2
0
13390

104421
1
7
species

 0.00

1
72771
8
1
5
tribe

 0.00


 0.00
genus
1
4
2
0
47732

1
4
1086592

 0.00
species

0
72766
3
1
2
genus

 0.00

3
1
72781

 0.00
species


 0.00
subfamily
1
5
3
0
40139

2
1
5
83484
0

 0.00
genus


 0.00
species
5
1
83485


 0.03
superfamily
1019
1
126
34735
37

0
124286
1
160
14
family

 0.00

subfamily

 0.00
0
156330
160
1
13

tribe

 0.00
0
156332
27
1
4

genus

 0.00
124287
3
3
1
27

0
1437190
1
24
2
species

 0.00

subspecies

 0.00
1437191
24
1


 0.00
tribe
1
45
3
0
156337

genus

 0.00
216413
0
2
1
45

45
1
2249760

 0.00
species

tribe

 0.00
156331
0
88
1
5

4
88
1
132116
28

 0.00
genus

481575
17
1
species

 0.00

143995
3
1
species

 0.00

1
40
1542540

 0.00
species


 0.01
family
245
1
22
3
77572

subfamily

 0.00
0
156312
4
1
2

2
1
3
0
178049

 0.00
genus

2
1
2
2448183
0

 0.00
subgenus

2448451
2
1
species

 0.00


 0.01
subfamily
237
1
14
6
77573

tribe

 0.00
0
479730
64
1
3

genus

 0.00
0
88591
2
64
1


 0.00
species
1
64
1190790

88544
1
10
1
167
tribe

 0.00

7
140
1
88467
5

 0.00
genus

1
35
2
88475
0

 0.00
subgenus

species

 0.00
88514
35
1

subgenus

 0.00
88474
0
1
54
2

88516
1
54
species

 0.00

46
1
2
88472
0

 0.00
subgenus

1
46
88531

 0.00
species

1
26
2
88466
0

 0.00
genus

species

 0.00
115100
26
1


 0.00
subfamily
3
1
3
178025
0

2
3
1
0
178032

 0.00
genus

species

 0.00
178035
3
1


 0.00
family
132
1
12
48719
0

subfamily

 0.00
0
205141
1
132
11


 0.00
genus
10
132
1
48720
16


 0.00
no rank
3
41
1
1126400
1

1190802
1
17
species

 0.00

1
23
1126402

 0.00
species

205271
0
2
50
1
subgenus

 0.00


 0.00
species
50
1
444401

1
11
2
0
205272

 0.00
subgenus

1
11
1411666

 0.00
species

2
14
1
205261
0

 0.00
subgenus

species

 0.00
1411667
1
14


 0.00
family
4
59
1
156323
0


 0.00
subfamily
3
1
59
0
253710

253714
0
2
59
1
genus

 0.00

253715
1
59
species

 0.00

no rank

 0.00
2153468
0
194
1
24

0
253718
194
1
23
family

 0.00

subfamily

 0.00
253722
0
6
1
83

tribe

 0.00
288410
0
5
1
83

83
1
4
0
421285

 0.00
subtribe


 0.00
genus
1
83
3
421297
0

29
1
1126389

 0.00
species

species

 0.00
2495015
1
54

288404
0
4
1
41
subfamily

 0.00


 0.00
tribe
3
41
1
302523
0

2
1
41
421220
0

 0.00
genus

species

 0.00
2495127
1
41

4
40
1
216423
0

 0.00
subfamily

0
288382
3
1
40
tribe

 0.00

288388
0
2
1
40
genus

 0.00

species

 0.00
1167272
1
40

253723
0
8
30
1
subfamily

 0.00

3
15
1
0
421418

 0.00
tribe


 0.00
genus
2
1
15
0
421423

1
15
2495085

 0.00
species

tribe

 0.00
0
302062
4
15
1

subtribe

 0.00
421392
0
15
1
3

0
421402
15
1
2
genus

 0.00

species

 0.00
2495172
1
15


 0.00
family
45
190
1
7458
0


 0.00
subfamily
1
172
35
70987
4

tribe

 0.00
83323
0
3
1
2

genus

 0.00
117248
0
1
2
2

species

 0.00
597456
1
2


 0.00
tribe
4
1
3
83319
0

2
4
1
0
166413

 0.00
genus

561572
1
4
species

 0.00

tribe

 0.00
83321
0
25
1
7


 0.00
genus
1
25
6
10
7459

species

 0.00
7461
1
1


 0.00
species
1
5
7460

1
1
7462

 0.00
species

1
4
7463

 0.00
species

species

 0.00
183418
4
1

tribe

 0.00
0
83310
3
1
3


 0.00
genus
2
1
3
0
28644

1
3
516756

 0.00
species


 0.00
tribe
1
134
18
83311
0

134
1
17
29
28641

 0.00
genus

3
1
2
144715
0

 0.00
subgenus


 0.00
species
3
1
396416

2
1
14
0
144700

 0.00
subgenus

65598
1
14
species

 0.00


 0.00
subgenus
26
1
3
7
144703

1
7
30194

 0.00
species

30191
1
12
species

 0.00

144708
4
2
20
1
subgenus

 0.00

16
1
30195

 0.00
species

0
144704
2
20
1
subgenus

 0.00


 0.00
species
20
1
85660

4
28653
3
1
21
subgenus

 0.00


 0.00
species
1
7
207624

species

 0.00
30201
10
1


 0.00
subgenus
1
1
2
309957
0

species

 0.00
309958
1
1

5
1
3
0
78170

 0.00
subfamily

tribe

 0.00
0
78171
4
3
1

0
78173
1
3
3
genus

 0.00

0
236025
2
1
3
subgenus

 0.00


 0.00
species
3
1
156304

78169
0
4
15
1
subfamily

 0.00


 0.00
tribe
15
1
3
0
95294

genus

 0.00
95295
0
2
1
15

601510
1
15
species

 0.00

family

 0.00
0
156309
4
1
2

subfamily

 0.00
178044
0
3
1
2


 0.00
genus
2
1
2
156310
0

935657
1
2
species

 0.00

1
66
5
1803217
0

 0.00
superfamily


 0.00
family
66
1
4
27515
0

subfamily

 0.00
1801551
0
3
66
1

genus

 0.00
200613
0
66
1
2

66
1
1667466

 0.00
species

2220
1
350
7147
152

 0.06
order

7148
6
104
1
612
suborder

 0.02


 0.00
infraorder
1
2
6
0
43789

2
1
5
41829
0

 0.00
superfamily

family

 0.00
0
41042
4
2
1

3
2
1
0
52735

 0.00
subfamily

0
46210
1
2
2
genus

 0.00


 0.00
species
2
1
2719080


 0.01
infraorder
75
501
1
2
43786

16
71
1
0
41828

 0.00
superfamily


 0.00
family
6
1
45
41819
0

5
1
45
43801
0

 0.00
subfamily

tribe

 0.00
0
58262
4
1
45

3
1
45
41820
0

 0.00
genus


 0.00
subgenus
2
45
1
0
58277

species

 0.00
179676
1
45

9
26
1
7149
0

 0.00
family

subfamily

 0.00
0
43807
1
1
3


 0.00
genus
2
1
1
338156
0


 0.00
no rank
1
1
2643826

25
1
5
54970
0

 0.00
subfamily


 0.00
tribe
4
1
25
72530
0

3
1
25
7150
0

 0.00
genus

25
1
2
0
1165752

 0.00
no rank


 0.00
species
25
1
315576

41827
0
58
428
1
superfamily

 0.01


 0.01
family
57
1
428
6
7157

subfamily

 0.00
0
43817
25
1
13

tribe

 0.00
1056966
0
1
14
5

7158
0
4
14
1
genus

 0.00


 0.00
subgenus
3
1
14
2
53541

6
1
7159

 0.00
species

7160
6
1
species

 0.00

0
53550
7
1
11
tribe

 0.00

0
7174
6
11
1
genus

 0.00

subgenus

 0.00
53527
0
5
11
1

no rank

 0.00
518105
8
1
11
4

species

 0.00
7176
1
1

species

 0.00
0
7175
2
1
2

42434
1
2
subspecies

 0.00

43816
0
397
1
43
subfamily

 0.01


 0.01
genus
1
397
42
7164
7

subgenus

 0.00
2
44534
142
1
20

clade

 0.00
44535
1
3
57
1

species

 0.00
30069
1
44

1
12
1496333

 0.00
species

1
29
5
59140
0

 0.00
clade

species

 0.00
1521116
13
1

2
1
6
0
59142

 0.00
species group

species

 0.00
62324
1
6

species

 0.00
186751
1
10

7
33
1
44537
0

 0.00
clade


 0.00
no rank
6
33
1
17
44542

1
2
30066

 0.00
species

species

 0.00
7173
1
1

2
1
5
7165
3

 0.00
species

180454
1
2
strain

 0.00

species

 0.00
1518534
8
1


 0.00
clade
1
21
4
0
44536

3
21
1
185573
0

 0.00
species group

no rank

 0.00
185574
0
2
1
21

species

 0.00
185578
1
21

subgenus

 0.00
0
44482
6
46
1

58247
0
1
46
5
section

 0.00


 0.00
series
4
1
46
0
58250


 0.00
species group
1
46
3
8
59130

18
1
139045

 0.00
species


 0.00
species
20
1
345580

2
44543
1
175
12
subgenus

 0.00


 0.00
clade
4
153
1
44545
0

clade

 0.00
44546
0
153
1
3

44552
0
1
153
2
species group

 0.00

43151
1
153
species

 0.00

clade

 0.00
0
44544
1
20
7

2
1
6
44547
0

 0.00
clade

6
1
7167

 0.00
species

clade

 0.00
0
44548
4
1
14

44549
0
3
14
1
species group

 0.00


 0.00
species subgroup
2
14
1
0
44550

42839
1
14
species

 0.00

27
1
3
0
68877

 0.00
subgenus

species

 0.00
68878
22
1

1
5
139047

 0.00
species

43787
0
7
1
15
infraorder

 0.00

1
15
6
41831
0

 0.00
superfamily

7197
0
1
15
5
family

 0.00

subfamily

 0.00
0
7198
15
1
4

7199
0
1
15
3
genus

 0.00

252607
0
1
15
2
subgenus

 0.00

1
15
7200

 0.00
species

1
88
15
43784
0

 0.00
infraorder

superfamily

 0.00
0
41830
9
1
3


 0.00
family
1
3
8
0
33406

subfamily

 0.00
43793
0
5
1
2

0
52723
4
1
2
no rank

 0.00

3
2
1
71814
0

 0.00
tribe


 0.00
genus
1
2
2
153220
0


 0.00
species
1
2
265458


 0.00
no rank
2
1
1
0
329961

species

 0.00
2908770
1
1

43790
0
85
1
5
superfamily

 0.00

family

 0.00
52729
0
4
1
85


 0.00
subfamily
1
85
3
0
52730


 0.00
genus
2
85
1
0
189978


 0.00
species
1
85
189979

1
1456
245
7203
1

 0.04
suborder


 0.04
infraorder
244
1
1455
43733
15

480118
0
233
1305
1
clade

 0.03


 0.03
clade
232
1
1305
39
480117

43737
0
396
1
60
no rank

 0.01

1
396
59
43740
3

 0.01
superfamily


 0.01
family
378
1
54
7
34680

subfamily

 0.00
0
43838
1
108
26

1
115274
95
1
20
tribe

 0.00

0
414873
2
1
2
genus

 0.00

414876
1
2
species

 0.00


 0.00
genus
3
1
2
0
290403


 0.00
subgenus
1
2
2
414732
0

1
2
1124558

 0.00
species


 0.00
genus
2
16
1
323311
0

1
16
323312

 0.00
species

2
1
11
414810
0

 0.00
genus

species

 0.00
1352479
1
11


 0.00
genus
2
10
1
0
414813

414814
10
1
species

 0.00

9
1
2
0
414800

 0.00
genus


 0.00
species
1
9
414801


 0.00
genus
1
14
2
286458
0

species

 0.00
286459
14
1

genus

 0.00
219538
0
2
1
28

219539
1
28
species

 0.00

2
1
2
226179
0

 0.00
genus

species

 0.00
2735243
2
1


 0.00
tribe
1
13
5
224219
0

1
5
2
0
92597

 0.00
genus

653684
5
1
species

 0.00

192444
0
1
8
2
genus

 0.00

414846
1
8
species

 0.00

115244
1
27
263
1
subfamily

 0.01

7
86
1
1
224230

 0.00
tribe

genus

 0.00
224240
0
40
1
2

40
1
374264

 0.00
species

1
18
2
0
34681

 0.00
genus

18
1
34682

 0.00
species

0
226146
27
1
2
genus

 0.00

species

 0.00
226147
1
27

tribe

 0.00
115284
0
3
1
1

2
1
1
0
226148

 0.00
genus


 0.00
species
1
1
226149


 0.00
tribe
7
1
44
0
192448


 0.00
genus
2
1
2
192449
0

2
1
226161

 0.00
species

genus

 0.00
173981
0
4
1
42

17
1
173985

 0.00
species

species

 0.00
273407
15
1


 0.00
species
10
1
273409

1
131
9
115277
1

 0.00
tribe

115278
0
2
1
51
genus

 0.00

species

 0.00
2725509
1
51

68
1
4
198633
0

 0.00
genus

species

 0.00
198635
1
21

species

 0.00
1572519
22
1

1
25
1124515

 0.00
species

2
1
11
0
2714348

 0.00
genus

species

 0.00
2714349
11
1

0
43835
4
15
1
family

 0.00


 0.00
subfamily
3
15
1
115302
0


 0.00
genus
2
15
1
115303
0

species

 0.00
566305
15
1

43738
19
171
870
1
no rank

 0.02

no rank

 0.00
2
43742
175
1
55

superfamily

 0.00
0
43753
5
1
5


 0.00
family
4
5
1
7392
0

7393
0
5
1
3
genus

 0.00

subgenus

 0.00
44051
0
5
1
2

1
5
7396

 0.00
species

1
153
39
43755
0

 0.00
superfamily

family

 0.00
0
7381
9
56
1

subfamily

 0.00
0
43916
56
1
8

0
7384
7
1
56
genus

 0.00

2
12
1
0
321190

 0.00
subgenus

596942
1
12
species

 0.00

1
41
2
0
236878

 0.00
subgenus

species

 0.00
1206372
41
1

3
1
2
0
226134

 0.00
subgenus

236850
1
3
species

 0.00


 0.00
family
9
14
1
0
7371

9
1
4
43914
0

 0.00
subfamily

1
9
3
7374
0

 0.00
genus

7375
1
3
species

 0.00

6
1
13632

 0.00
species


 0.00
subfamily
4
5
1
43913
0

tribe

 0.00
0
54282
1
5
3

genus

 0.00
0
142901
2
5
1

5
1
226133

 0.00
species

54279
0
12
1
4
family

 0.00

3
12
1
229639
0

 0.00
genus

species

 0.00
670595
1
8


 0.00
species
4
1
1266490

0
27474
71
1
16
family

 0.00


 0.00
subfamily
6
1
5
0
54288


 0.00
tribe
1
6
4
0
141256

genus

 0.00
0
141257
3
6
1


 0.00
species
4
1
631328

2
1
631329

 0.00
species

subfamily

 0.00
43917
0
6
1
64

0
179426
1
64
5
tribe

 0.00

genus

 0.00
0
569039
1
62
2


 0.00
species
1
62
569040

1918309
0
2
2
1
genus

 0.00

1918310
1
2
species

 0.00


 0.00
subfamily
4
1
1
0
54286

1
1
3
569110
0

 0.00
tribe


 0.00
genus
2
1
1
1918220
0


 0.00
species
1
1
1918221

15
1
10
43754
0

 0.00
superfamily

family

 0.00
0
7366
9
1
15


 0.00
subfamily
8
15
1
0
43910

4
10
1
0
57894

 0.00
tribe


 0.00
genus
3
10
1
7369
0

44052
0
10
1
2
subgenus

 0.00

7370
1
10
species

 0.00

tribe

 0.00
43911
0
3
1
5


 0.00
genus
5
1
2
35569
0


 0.00
species
1
5
35570

1
676
115
43741
24

 0.02
no rank


 0.00
superfamily
1
4
6
43750
0

0
169447
4
1
5
family

 0.00

genus

 0.00
1226614
0
1
1
2

species

 0.00
1226616
1
1


 0.00
genus
2
1
3
1096076
0

1096077
3
1
species

 0.00


 0.01
superfamily
1
486
71
0
43746

family

 0.01
0
7214
486
1
70

69
486
1
43845
0

 0.01
subfamily

64
1
484
46877
0

 0.01
tribe


 0.01
genus
63
484
1
54
7215

206
1
37
9
32341

 0.01
subgenus

32346
8
25
1
109
species group

 0.00


 0.00
species subgroup
8
58
1
2
32351

2
1
129105

 0.00
species


 0.00
species
1
3
7245

species

 0.00
7243
1
3

1
18
7240

 0.00
species

species

 0.00
7220
4
1

1
20
7227

 0.00
species

1
6
7226

 0.00
species

species subgroup

 0.00
0
32348
5
1
2

30023
5
1
species

 0.00

0
32349
1
6
2
species subgroup

 0.00

species

 0.00
29029
1
6

species subgroup

 0.00
65962
0
2
1
15

15
1
1041015

 0.00
species

species subgroup

 0.00
1
32347
3
4
1

0
186282
3
1
2
no rank

 0.00


 0.00
species
1
3
42026


 0.00
species subgroup
3
6
1
1
32353


 0.00
species
4
1
1486046

1
1
125945

 0.00
species

32350
0
1
2
2
species subgroup

 0.00

30025
2
1
species

 0.00

species subgroup

 0.00
0
32354
1
5
2


 0.00
species
1
5
29030


 0.00
species group
8
85
1
0
32355

species subgroup

 0.00
7
32358
4
61
1

47
1
7229

 0.00
species


 0.00
species
1
4
7237

3
1
7234

 0.00
species

species subgroup

 0.00
32357
12
1
24
3

species

 0.00
7241
3
1


 0.00
species
9
1
7266

3
1
3
32365
0

 0.00
species group

0
32367
1
3
2
species subgroup

 0.00

3
1
7260

 0.00
species

32281
2
17
1
30
subgenus

 0.00

32335
1
8
1
3
species group

 0.00


 0.00
species
4
1
7244

3
1
47314

 0.00
species


 0.00
species group
1
7
3
32304
0

species subgroup

 0.00
32307
0
1
7
2

7291
1
7
species

 0.00

species group

 0.00
32321
0
6
1
6

40364
0
2
1
1
species subgroup

 0.00

1
1
7224

 0.00
species

1
5
3
32324
0

 0.00
species subgroup

198037
1
2
1
5
no rank

 0.00

4
1
7232

 0.00
species


 0.00
species group
2
1
1
0
66367

66368
1
1
species

 0.00

species group

 0.00
0
32320
1
6
2

198719
6
1
species

 0.00

no rank

 0.00
504493
0
1
143
6

clade

 0.00
0
48384
1
143
5

48301
0
1
143
4
clade

 0.00

143
1
3
0
48302

 0.00
species group

143
1
2
32378
0

 0.00
species subgroup

7222
1
143
species

 0.00

subgenus

 0.00
0
32280
2
1
51


 0.00
species
1
51
30019

1861795
0
4
1
2
tribe

 0.00

7354
0
2
1
3
genus

 0.00


 0.00
species group
2
2
1
32386
0

species

 0.00
7225
2
1

43744
0
9
107
1
superfamily

 0.00

family

 0.00
115263
0
107
1
8

7
107
1
8
115265

 0.00
subfamily


 0.00
genus
2
39
1
286486
0


 0.00
species
39
1
2829445

1219203
0
1
31
2
genus

 0.00

1
31
1219204

 0.00
species

1
29
2
0
305546

 0.00
genus

species

 0.00
1219171
29
1

3
1
4
0
43745

 0.00
superfamily


 0.00
family
3
1
3
139644
0

139679
0
2
1
3
genus

 0.00

species

 0.00
139649
3
1


 0.00
superfamily
52
1
24
43752
0

23
1
52
7211
0

 0.00
family


 0.00
subfamily
6
1
27
0
43867


 0.00
tribe
1
27
5
43901
0

subtribe

 0.00
164882
0
27
1
4

genus

 0.00
1
28609
3
1
27


 0.00
species
1
3
28610

28612
23
1
species

 0.00

subfamily

 0.00
0
164860
1
25
16

11
20
1
0
43871

 0.00
tribe

genus

 0.00
0
27456
7
10
1


 0.00
subgenus
2
4
1
0
69624

species

 0.00
104688
4
1


 0.00
subgenus
4
1
6
47832
3

174628
1
1
species

 0.00

0
98808
2
2
1
no rank

 0.00

species

 0.00
98809
2
1

1
10
3
0
47833

 0.00
genus

1
10
2
0
1987911

 0.00
subgenus

28588
1
10
species

 0.00

0
164862
1
5
4
tribe

 0.00


 0.00
genus
3
1
5
0
7212

subgenus

 0.00
0
474492
2
1
5

species

 0.00
7213
5
1

50671
2
10
135
1
superfamily

 0.00


 0.00
family
31
1
4
50673
0


 0.00
subfamily
31
1
3
0
50679

2
31
1
0
247604

 0.00
genus

species

 0.00
2794001
31
1

50674
0
5
1
102
family

 0.00

4
1
102
0
50694

 0.00
subfamily

102
1
3
10
219362

 0.00
genus

1
53
2741128

 0.00
species

species

 0.00
240869
1
39

103
268
1
33342
0

 0.01
cohort

9
1
9
0
30262

 0.00
order

0
38130
1
9
8
suborder

 0.00

superfamily

 0.00
0
45049
7
1
9


 0.00
family
9
1
6
45053
0

0
153976
5
9
1
subfamily

 0.00

genus

 0.00
0
45059
1
4
2

1
4
133901

 0.00
species

genus

 0.00
0
45057
2
5
1

161013
1
5
species

 0.00


 0.00
superorder
11
10
1
1930602
0


 0.00
order
10
10
1
85819
0

suborder

 0.00
0
30005
7
1
5

family

 0.00
0
121221
4
7
1

1
7
3
121222
0

 0.00
genus

0
121225
2
7
1
species

 0.00


 0.00
subspecies
7
1
121224

3
1
4
35634
0

 0.00
suborder

family

 0.00
0
66236
1
3
3

1
3
2
186208
0

 0.00
genus

species

 0.00
382674
1
3

249
1
82
3
7524

 0.01
order

33373
1
166
1
42
suborder

 0.00


 0.00
infraorder
137
1
28
0
33380

19
1
124
0
33385

 0.00
superfamily


 0.00
family
18
124
1
0
27482

subfamily

 0.00
7
133076
1
122
14


 0.00
tribe
42
1
7
0
33386

genus

 0.00
143947
0
1
2
2


 0.00
species
1
2
143948


 0.00
genus
20
1
2
0
13163

species

 0.00
13164
1
20

0
7028
2
1
20
genus

 0.00

20
1
7029

 0.00
species

0
33387
6
1
73
tribe

 0.00

3
72
1
80764
0

 0.00
genus

1
72
2
464929
0

 0.00
subgenus


 0.00
species
72
1
80765

1
1
2
40931
0

 0.00
genus

1
1
43146

 0.00
species

3
1
2
805116
0

 0.00
subfamily

genus

 0.00
143949
0
1
2
2

species

 0.00
143950
2
1

superfamily

 0.00
0
33382
8
1
13

4
1
3
0
33384

 0.00
family

4
1
2
58001
0

 0.00
genus

58002
1
4
species

 0.00

family

 0.00
33383
0
4
9
1


 0.00
genus
3
1
9
0
38118

0
749396
2
1
9
subgenus

 0.00


 0.00
species
9
1
133065

5
1
25
33377
0

 0.00
superfamily

7036
0
25
1
4
family

 0.00

0
33379
3
25
1
subfamily

 0.00

2
1
25
7037
0

 0.00
genus

7038
25
1
species

 0.00

33381
0
4
1
1
superfamily

 0.00


 0.00
family
1
1
3
0
30189

genus

 0.00
0
1661414
1
1
2


 0.00
species
1
1
1661415

0
33375
2
1
4
superfamily

 0.00


 0.00
family
2
1
3
1585420
0


 0.00
genus
1
2
2
121844
0

species

 0.00
121845
1
2

25
1
72
33343
0

 0.00
clade


 0.00
suborder
24
1
72
33345
0

0
33347
23
72
1
clade

 0.00

22
1
72
0
33349

 0.00
clade


 0.00
clade
72
1
21
0
33351

infraorder

 0.00
0
33357
15
1
68

superfamily

 0.00
38105
0
5
36
1

0
186376
4
36
1
family

 0.00

subfamily

 0.00
2068237
0
3
36
1

36
1
2
0
1276926

 0.00
genus

species

 0.00
1545138
1
36

1
32
9
33358
0

 0.00
superfamily

8
32
1
160513
0

 0.00
family


 0.00
subfamily
7
32
1
286710
0

85309
0
16
1
2
genus

 0.00

85310
1
16
species

 0.00

genus

 0.00
0
286705
2
1
6

species

 0.00
286706
6
1

10
1
2
631388
0

 0.00
genus


 0.00
species
10
1
1511221


 0.00
infraorder
5
4
1
33354
0

superfamily

 0.00
0
33355
4
1
4

family

 0.00
0
30078
3
1
4


 0.00
genus
2
4
1
30079
0

79782
1
4
species

 0.00


 0.00
suborder
1
8
14
0
1955247

infraorder

 0.00
33361
0
6
1
2


 0.00
superfamily
1
2
5
36151
0

33362
0
4
1
2
family

 0.00


 0.00
subfamily
3
1
2
130551
0

genus

 0.00
108930
0
2
2
1


 0.00
species
2
1
108931

0
33365
7
1
6
infraorder

 0.00


 0.00
superfamily
6
1
6
0
33368

family

 0.00
30102
0
5
6
1

1
6
4
0
33370

 0.00
subfamily

tribe

 0.00
0
565685
6
1
3

0
139475
6
1
2
genus

 0.00


 0.00
species
6
1
197043


 0.02
subphylum
1
619
106
3
6657

6
2172821
76
511
1
superclass

 0.01


 0.00
class
1
26
6
0
116172


 0.00
subclass
5
26
1
0
6675

infraclass

 0.00
0
37909
26
1
4

3
26
1
38011
0

 0.00
family

genus

 0.00
0
51649
2
1
26

species

 0.00
51650
26
1

1
225
15
72037
0

 0.01
class

0
6830
225
1
14
subclass

 0.01

13
1
225
0
116569

 0.01
infraclass

116570
0
5
1
1
superorder

 0.00

order

 0.00
0
6833
1
1
4

family

 0.00
0
88013
1
1
3


 0.00
genus
2
1
1
0
88014

species

 0.00
88015
1
1

superorder

 0.01
0
116571
7
224
1

6
224
1
72033
0

 0.01
order


 0.01
family
5
1
224
2
72034

1
102
2
72035
0

 0.00
genus


 0.00
species
102
1
72036

2
1
120
0
217164

 0.00
genus

species

 0.00
217165
120
1


 0.01
class
54
1
254
0
6681

53
254
1
0
72041

 0.01
subclass

0
6820
1
5
14
superorder

 0.00

order

 0.00
0
6821
5
1
13

0
1732196
12
1
5
suborder

 0.00

infraorder

 0.00
0
1732206
5
1
1

0
2992673
4
1
1
superfamily

 0.00


 0.00
family
1
1
3
0
199478

genus

 0.00
0
280676
2
1
1

species

 0.00
317513
1
1

0
1732204
6
4
1
infraorder

 0.00


 0.00
parvorder
4
1
5
0
1732303

4
1
4
0
1732305

 0.00
superfamily

family

 0.00
1041812
0
1
4
3

genus

 0.00
1041813
0
4
1
2

species

 0.00
2211525
4
1

6682
0
38
1
249
superorder

 0.01

order

 0.01
1
6683
37
1
249


 0.00
suborder
8
19
1
0
6684

superfamily

 0.00
0
111520
1
19
7


 0.00
family
19
1
6
0
6685

5
1
19
1
133894

 0.00
genus


 0.00
species
1
7
6687

6689
1
6
species

 0.00

139456
1
2
species

 0.00

species

 0.00
27405
1
3

suborder

 0.01
6692
1
28
1
229


 0.00
infraorder
10
8
1
0
6712

0
37849
4
7
1
superfamily

 0.00


 0.00
family
3
7
1
0
6704


 0.00
genus
1
7
2
0
6705

1
7
6706

 0.00
species


 0.00
superfamily
5
1
1
0
6724


 0.00
family
1
1
4
0
6725

3
1
1
0
72430

 0.00
subfamily


 0.00
genus
1
1
2
6726
0

6728
1
1
species

 0.00

6752
0
23
1
12
infraorder

 0.00

23
1
11
116704
0

 0.00
no rank


 0.00
no rank
1
17
5
116707
0

4
17
1
0
29962

 0.00
superfamily

1
17
3
0
72876

 0.00
family

genus

 0.00
0
95601
2
17
1

1
17
95602

 0.00
species

5
1
6
116706
0

 0.00
no rank

4
6
1
0
6774

 0.00
superfamily

6
1
3
6757
0

 0.00
family

subfamily
600346
2
6


 0.00
genus
2
1
6
0
80835

6
1
210409

 0.00
species

1
197
5
0
6694

 0.00
infraorder

4
197
1
115580
0

 0.00
superfamily

0
6695
3
1
197
family

 0.00

197
1
2
0
6696

 0.00
genus


 0.00
species
197
1
159736

class

 0.00
6658
0
1
14
13


 0.00
subclass
12
1
14
116557
0

1
12
7
84337
0

 0.00
order

0
6665
12
1
6
suborder

 0.00


 0.00
infraorder
5
12
1
116561
0


 0.00
family
4
1
12
77658
0


 0.00
genus
12
1
3
6668
0

species

 0.00
6669
11
1


 0.00
species
1
1
35523

order

 0.00
58774
0
4
2
1

family

 0.00
63729
0
1
2
3

0
58776
2
1
2
genus

 0.00

58777
1
2
species

 0.00

16
1
91
2172819
0

 0.00
superclass

0
6670
91
1
15
class

 0.00


 0.00
subclass
14
1
91
43953
0


 0.00
order
13
91
1
0
84318


 0.00
suborder
7
64
1
84328
0

superfamily

 0.00
0
84329
1
64
6

5
64
1
43954
0

 0.00
family

2
1
32
0
399044

 0.00
genus

32
1
399045

 0.00
species


 0.00
genus
2
1
32
163713
0

163714
1
32
species

 0.00

suborder

 0.00
116574
0
1
27
5

0
116575
27
1
4
superfamily

 0.00


 0.00
family
3
1
27
69351
0

2
1
27
69354
0

 0.00
genus

69355
1
27
species

 0.00

6843
2
117
1
1829
subphylum

 0.05

111
1
1819
6854
3

 0.05
class

75
1
1754
1
6933

 0.04
subclass

superorder

 0.04
6934
0
32
1
1534

order

 0.00
34634
0
1
9
9

suborder

 0.00
281668
0
9
1
8


 0.00
infraorder
9
1
7
1723665
2

superfamily

 0.00
41438
2
6
7
1

3
3
1
0
109261

 0.00
family

genus

 0.00
62624
1
2
1
3

2
1
62625

 0.00
species

family

 0.00
48831
1
2
1
2


 0.00
genus
1
1
797477


 0.04
order
1525
1
22
0
6935

1525
1
21
1
297308

 0.04
superfamily

20
1
1524
6939
7

 0.04
family

426437
77
13
1
1508
subfamily

 0.04

41
1
3
3
34619

 0.00
genus

543639
1
34
species

 0.00


 0.00
species
1
4
34620

9
1390
1
94
34630

 0.03
genus

subgenus

 0.03
426455
47
6
1264
1

species

 0.00
72861
4
1


 0.03
species group
1186
1
2
578835
0

34632
1
1186
species

 0.03

2
1
578836

 0.00
species

25
1
34631

 0.00
species

subgenus

 0.00
0
6940
32
1
2

6941
32
1
species

 0.00

3
8
1
0
426442

 0.00
subfamily

8
1
2
0
6944

 0.00
genus

species

 0.00
6945
8
1

1
1
3
0
426441

 0.00
subfamily

genus

 0.00
0
6942
1
1
2

1
1
34610

 0.00
species

1
219
42
4
6946

 0.01
superorder

19
16
1
0
83136

 0.00
order

18
16
1
6947
3

 0.00
suborder

83138
0
1
10
11
infraorder

 0.00


 0.00
clade
10
10
1
1
83141

0
92068
1
1
5
clade

 0.00


 0.00
superfamily
1
1
4
0
257012


 0.00
family
1
1
3
1633871
0

genus

 0.00
1633861
0
1
1
2

species

 0.00
1633862
1
1

4
1
8
92088
0

 0.00
superfamily


 0.00
family
3
8
1
7
92251

1
1
2
768112
0

 0.00
no rank

species

 0.00
2979943
1
1

infraorder

 0.00
0
83145
3
1
6

0
188550
5
3
1
no rank

 0.00

0
83146
3
1
4
superfamily

 0.00

family

 0.00
32262
0
3
1
3

genus

 0.00
0
32263
2
1
3

1
3
32264

 0.00
species


 0.00
order
22
199
1
83137
0

66551
0
1
136
8
suborder

 0.00

infraorder

 0.00
229894
0
1
136
7

0
229794
136
1
6
superfamily

 0.00

229795
2
5
1
136
family

 0.00

65
1
2
1979940
0

 0.00
genus

1
65
1979941

 0.00
species

genus

 0.00
334624
0
69
1
2


 0.00
species
1
69
334625


 0.00
suborder
13
63
1
0
6951

parvorder

 0.00
223472
1
12
1
63

0
83158
5
45
1
superfamily

 0.00

family

 0.00
0
52281
45
1
4


 0.00
subfamily
3
1
45
474019
0

genus

 0.00
52282
0
1
45
2

52283
1
45
species

 0.00

superfamily

 0.00
83163
0
6
1
17

6952
0
5
1
17
family

 0.00

0
474036
17
1
4
subfamily

 0.00


 0.00
genus
17
1
3
1
6953


 0.00
species
6
1
6954


 0.00
species
10
1
6956

order

 0.00
0
6855
6
1
6


 0.00
parvorder
6
1
5
259437
0

superfamily

 0.00
0
70336
6
1
4

6856
0
1
6
3
family

 0.00

2
6
1
6875
0

 0.00
genus


 0.00
species
6
1
218467

56
1
29
0
6893

 0.00
order

28
56
1
6905
0

 0.00
suborder


 0.00
clade
27
56
1
0
74971

0
74974
8
1
46
clade

 0.00

superfamily

 0.00
0
74975
46
1
7

3
11
1
34643
0

 0.00
family

0
449632
2
1
11
genus

 0.00

1
11
114398

 0.00
species

0
27394
35
1
3
family

 0.00


 0.00
genus
1
35
2
94025
0

species

 0.00
1926196
1
35

0
175332
4
1
5
superfamily

 0.00


 0.00
family
3
1
5
0
175333

175340
0
1
5
2
genus

 0.00


 0.00
species
1
5
202533

0
94020
5
1
14
clade

 0.00

clade

 0.00
94015
0
1
5
13

3
3
1
152923
0

 0.00
family

3
1
2
152924
0

 0.00
genus


 0.00
species
3
1
2926465


 0.00
family
1
2
9
0
94017


 0.00
subfamily
4
1
1
1222166
0

genus

 0.00
0
1190772
3
1
1


 0.00
no rank
2
1
1
2677611
0

1
1
1222095

 0.00
species

1
1
4
243881
0

 0.00
clade

1
1
3
0
243884

 0.00
clade

2
1
1
153384
0

 0.00
subfamily


 0.00
genus
1
1
130929


 0.00
class
5
8
1
0
6844


 0.00
order
8
1
4
6845
0


 0.00
family
3
8
1
6846
0


 0.00
genus
2
1
8
0
6849

1
8
6850

 0.00
species


 0.01
phylum
68
1
271
2
6231


 0.01
class
259
1
61
1
119089

1
212
46
0
6236

 0.01
order


 0.00
suborder
103
1
19
0
2301116

infraorder

 0.00
2301119
0
18
103
1


 0.00
superfamily
17
103
1
0
55879

1
103
16
0
6243

 0.00
family

subfamily

 0.00
0
55885
7
1
30

6
30
1
6237
0

 0.00
genus

5
1
31234

 0.00
species

9
1
6239

 0.00
species


 0.00
species
1
11
1978547

1
1
2306311

 0.00
species

6238
4
1
species

 0.00

8
1
73
0
55887

 0.00
subfamily

genus

 0.00
0
42476
7
73
1


 0.00
species
1
5
473156


 0.00
species
1
56
141969

no rank

 0.00
0
2613844
1
9
3


 0.00
species
3
1
2879420

2879419
6
1
species

 0.00

1559960
3
1
species

 0.00

0
6274
5
1
1
suborder

 0.00

1
1
4
0
2072716

 0.00
infraorder


 0.00
superfamily
3
1
1
0
6295

1
1
2
0
48791

 0.00
family


 0.00
genus
1
1
48796

0
6300
21
108
1
suborder

 0.00


 0.00
infraorder
93
1
14
2082223
0

superfamily

 0.00
2082224
0
13
1
93

family

 0.00
6246
0
87
1
8

1
16
2
131309
0

 0.00
genus

1
16
131310

 0.00
species


 0.00
genus
1
71
5
3
6247

species

 0.00
6248
9
1


 0.00
species
1
20
75913

18
1
174720

 0.00
species


 0.00
species
21
1
34506


 0.00
family
4
1
6
114888
0


 0.00
genus
3
6
1
0
114889


 0.00
no rank
2
6
1
2629767
0


 0.00
species
1
6
114890


 0.00
infraorder
1
15
6
0
33283

superfamily

 0.00
33284
0
5
1
15

1
15
4
0
6301

 0.00
family

33286
0
1
15
3
subfamily

 0.00

0
34509
15
1
2
genus

 0.00

51029
1
15
species

 0.00

1
46
14
0
6308

 0.00
order

5
1
1
2572558
0

 0.00
superfamily

0
33278
4
1
1
family

 0.00

subfamily

 0.00
0
53477
1
1
3

51030
0
2
1
1
genus

 0.00

51031
1
1
species

 0.00

4
1
43
6314
0

 0.00
superfamily

family

 0.00
126387
0
3
1
43

6288
0
1
43
2
genus

 0.00

6289
1
43
species

 0.00

4
1
2
27829
0

 0.00
superfamily


 0.00
family
3
2
1
0
321367

0
321368
2
1
2
genus

 0.00

species

 0.00
321369
1
2

10
1
6
0
119088

 0.00
class


 0.00
subclass
5
10
1
0
1457286

order

 0.00
6329
0
4
10
1


 0.00
family
10
1
3
0
6332

2
10
1
6333
0

 0.00
genus

species

 0.00
6334
10
1

1
3224
271
7
2697495

 0.08
clade


 0.00
clade
40
1
14
0
2697496

0
10190
40
1
13
phylum

 0.00


 0.00
class
12
40
1
2816136
0

0
10191
1
1
6
subclass

 0.00

superorder

 0.00
1709201
0
5
1
1

1
1
4
84394
0

 0.00
order

family

 0.00
10193
0
1
1
3

genus

 0.00
10194
0
2
1
1

species

 0.00
10195
1
1

1
39
5
44578
0

 0.00
subclass

39
1
4
0
104779

 0.00
order

1
39
3
0
104780

 0.00
family


 0.00
genus
2
39
1
0
104781

104782
39
1
species

 0.00

256
3177
1
191
1206795

 0.08
clade


 0.00
phylum
1
68
20
0
10205

class

 0.00
10206
0
19
68
1

order

 0.00
10207
0
1
68
18


 0.00
suborder
5
1
19
193205
0

0
193206
4
1
19
superfamily

 0.00

0
192924
19
1
3
family

 0.00

19
1
2
95169
0

 0.00
genus

1
19
95170

 0.00
species

12
1
49
0
558764

 0.00
suborder

superfamily

 0.00
193209
0
7
20
1


 0.00
family
1
19
3
0
10210


 0.00
genus
1
19
2
1970207
0

species

 0.00
192920
1
19

3
1
1
0
97286

 0.00
family

2
1
1
0
2724385

 0.00
genus

species

 0.00
2724386
1
1

0
193246
1
29
4
superfamily

 0.00

0
558762
3
29
1
family

 0.00

genus

 0.00
0
558754
1
29
2

1
29
558755

 0.00
species

6
43
1
0
6217

 0.00
phylum


 0.00
class
5
43
1
0
6218

43
1
4
6219
0

 0.00
order

6222
0
1
43
3
family

 0.00

2
1
43
6223
0

 0.00
genus

43
1
88925

 0.00
species

1
4
8
0
7568

 0.00
phylum

1
4
7
115360
0

 0.00
subphylum

6
1
4
115361
0

 0.00
class

7570
0
5
1
4
order

 0.00

115362
0
4
4
1
superfamily

 0.00

3
1
4
33491
0

 0.00
family

7571
0
2
1
4
genus

 0.00

7574
4
1
species

 0.00

523
1
58
2
6157

 0.01
phylum


 0.00
class
1
3
14
147100
0


 0.00
clade
13
3
1
0
166126


 0.00
order
12
1
3
6159
0

3
1
11
0
1292243

 0.00
suborder


 0.00
superfamily
4
1
1
1292248
0

0
27896
3
1
1
family

 0.00


 0.00
genus
1
1
2
27899
0

1
1
1051007

 0.00
species


 0.00
superfamily
2
1
6
0
1292253

31262
0
2
1
5
family

 0.00


 0.00
genus
1
1
2
0
6160

1
1
6161

 0.00
species

genus

 0.00
55270
0
2
1
1

species

 0.00
79327
1
1

103
1
14
6199
0

 0.00
class

subclass

 0.00
6200
0
103
1
13

0
1224679
4
62
1
order

 0.00

3
1
62
0
28843

 0.00
family

62
1
2
0
46580

 0.00
genus


 0.00
species
1
62
99802


 0.00
order
1
41
8
0
6201


 0.00
family
34
1
3
0
6214

6215
0
2
34
1
genus

 0.00


 0.00
species
1
34
85433

family

 0.00
0
6208
4
1
7

genus

 0.00
0
6209
1
7
3

0
2212966
1
7
2
species group

 0.00

6210
7
1
species

 0.00


 0.01
class
29
415
1
6178
0

6179
0
28
1
415
subclass

 0.01


 0.01
order
21
1
383
0
6180


 0.01
superfamily
383
1
20
31244
0

19
383
1
6
31245

 0.01
family


 0.00
genus
3
1
40
100601
0

25
1
157069

 0.00
species


 0.00
species
1
15
157070

13
306
1
95
6181

 0.01
genus

6183
15
1
species

 0.00

11
1
6186

 0.00
species


 0.00
species
1
23
6188

species

 0.00
6182
1
1

6185
1
9
species

 0.00

6184
1
26
species

 0.00

1163369
28
1
species

 0.00

393876
5
1
species

 0.00

16
1
31246

 0.00
species

48269
1
36
species

 0.00


 0.00
species
10
1
6187

31
1
6189

 0.00
species


 0.00
genus
2
31
1
39198
0

31
1
39320

 0.00
species

27871
0
6
32
1
order

 0.00

5
1
32
27872
0

 0.00
suborder


 0.00
superfamily
4
1
32
0
1776223


 0.00
family
32
1
3
73421
0

57077
0
32
1
2
genus

 0.00


 0.00
species
1
32
57078


 0.05
phylum
1
2061
129
71
6447

1
6
15
6605
0

 0.00
class


 0.00
subclass
6
1
14
6606
0


 0.00
superorder
6
1
1
215450
0

order

 0.00
551287
0
5
1
1

34531
0
4
1
1
family

 0.00

subfamily

 0.00
0
2094723
3
1
1

1
1
2
34536
0

 0.00
genus

70197
1
1
species

 0.00

5
1
7
215451
0

 0.00
superorder

order

 0.00
0
6638
1
5
6

6646
0
5
1
5
suborder

 0.00

family

 0.00
6647
0
4
1
5

1
6643
3
5
1
genus

 0.00

species

 0.00
2607531
2
1

species

 0.00
37653
2
1


 0.01
class
573
1
61
6448
10

2219556
0
3
1
5
subclass

 0.00


 0.00
superfamily
4
3
1
216274
0

1
3
3
55007
0

 0.00
family

genus

 0.00
1735270
0
1
3
2

1
3
1735272

 0.00
species

0
216275
1
302
17
subclass

 0.01

order

 0.01
2315720
0
10
1
285


 0.01
superfamily
9
285
1
216285
0

family

 0.01
6466
0
285
1
8


 0.01
subfamily
1
285
7
1955429
6


 0.00
genus
1
86
2
0
148341

species

 0.00
703304
86
1

76
1
2
1093071
0

 0.00
genus

1620919
76
1
species

 0.00


 0.00
genus
1
117
2
2072689
0

species

 0.00
216125
117
1

order

 0.00
2315723
0
6
1
17

0
216276
1
17
5
superfamily

 0.00


 0.00
family
4
1
17
6451
0

genus

 0.00
6452
2
3
1
17

species

 0.00
36100
1
8


 0.00
species
1
7
6454

subclass

 0.00
0
69555
6
10
1


 0.00
order
10
1
5
75116
0

4
10
1
6475
0

 0.00
superfamily

family

 0.00
0
54973
3
10
1

72702
0
2
10
1
genus

 0.00


 0.00
species
1
10
400727


 0.00
subclass
134
1
11
0
69675

superfamily

 0.00
216260
0
5
9
1

1
9
4
69676
0

 0.00
family

genus

 0.00
72691
0
9
1
3


 0.00
species
8
1
225164

351231
1
1
species

 0.00

superfamily

 0.00
146277
0
1
125
5

6462
0
125
1
4
family

 0.00

genus

 0.00
6463
3
125
1
3


 0.00
species
1
62
6465

species

 0.00
88005
60
1


 0.00
subclass
114
1
21
216305
0

20
114
1
216307
0

 0.00
clade

977775
0
13
106
1
clade

 0.00


 0.00
clade
5
104
1
0
977779

superfamily

 0.00
0
216441
4
104
1


 0.00
family
1
104
3
0
6524


 0.00
genus
2
1
104
6525
0

6526
1
104
species

 0.00

2
1
7
0
120490

 0.00
superorder

6
1
2
6527
0

 0.00
order

5
1
2
0
216366

 0.00
clade

0
87871
4
1
2
superfamily

 0.00

0
37859
2
1
3
family

 0.00


 0.00
genus
2
1
2
0
1338343

species

 0.00
1338344
1
2

6
1
8
2836391
0

 0.00
clade

5
8
1
0
6497

 0.00
order

superfamily

 0.00
216318
0
4
1
8

family

 0.00
6498
0
8
1
3

genus

 0.00
6499
0
2
8
1

6500
8
1
species

 0.00


 0.03
class
52
1411
1
6544
1

51
1410
1
36
2785011

 0.03
subclass

clade

 0.03
0
6599
1
1047
29

1
1047
28
735337
0

 0.03
infraclass

superorder

 0.03
2785015
0
27
1047
1


 0.03
clade
26
1047
1
2908833
16

order

 0.02
2783445
9
1
935
16


 0.02
superfamily
698
1
11
105710
0

family

 0.02
4
55708
698
1
10

1
85
3
0
2787989

 0.00
subfamily

2
1
85
80820
0

 0.00
genus

species

 0.00
80821
1
85


 0.02
subfamily
609
1
6
4
2787994

0
52939
3
1
517
genus

 0.01


 0.00
species
90
1
80829


 0.01
species
427
1
80833

genus

 0.00
0
80817
2
1
88

80818
1
88
species

 0.00

superfamily

 0.01
0
98297
4
228
1


 0.01
family
228
1
3
0
61354


 0.01
genus
228
1
2
0
457750

species

 0.01
2589376
228
1


 0.00
order
96
1
9
6580
0

0
74489
1
6
4
superfamily

 0.00

family

 0.00
0
6592
6
1
3

2
6
1
6595
0

 0.00
genus

6596
1
6
species

 0.00

4
90
1
0
106231

 0.00
superfamily

6581
0
1
90
3
family

 0.00

2
1
90
0
6582

 0.00
genus

31201
1
90
species

 0.00


 0.01
clade
327
1
21
6545
2

0
106218
7
123
1
order

 0.00

0
106219
123
1
6
superfamily

 0.00

family

 0.00
6566
1
5
1
123


 0.00
genus
2
57
1
0
6578

species

 0.00
6579
57
1

186466
0
2
1
65
genus

 0.00

65
1
6573

 0.00
species

order

 0.00
6546
0
6
1
72

1
72
5
106220
0

 0.00
superfamily

0
6547
4
1
72
family

 0.00


 0.00
subfamily
3
72
1
2899742
0

72
1
2
356392
0

 0.00
genus


 0.00
species
72
1
356393

7
130
1
0
6562

 0.00
order

superfamily

 0.00
0
98302
1
130
6

family

 0.00
6563
0
5
130
1


 0.00
genus
2
1
125
6564
0


 0.00
species
1
125
29159

0
37858
2
5
1
genus

 0.00

5
1
37623

 0.00
species

34
287
1
7
6340

 0.01
phylum

6341
0
136
1
12
class

 0.00

11
1
136
105390
0

 0.00
subclass

6348
0
136
1
10
order

 0.00


 0.00
family
1
97
3
46593
0

0
222002
2
1
97
genus

 0.00

97
1
1210411

 0.00
species

39820
0
1
33
3
family

 0.00

genus

 0.00
0
868094
2
1
33


 0.00
species
33
1
880429

0
104728
6
1
3
family

 0.00

55701
0
2
1
6
genus

 0.00

6
1
1210413

 0.00
species

1
144
21
42113
0

 0.00
class

subclass

 0.00
0
55824
1
42
10

order

 0.00
0
6406
4
1
4

1
4
3
6407
0

 0.00
family

0
6411
2
4
1
genus

 0.00

1
4
6412

 0.00
species

5
38
1
2218736
0

 0.00
order

suborder

 0.00
2218739
0
1
38
4

60930
0
38
1
3
family

 0.00

genus

 0.00
0
60957
2
38
1

species

 0.00
60958
38
1

1
102
10
0
6381

 0.00
subclass

9
102
1
0
2803884

 0.00
order

suborder

 0.00
6391
0
102
1
8

7
1
102
0
6392

 0.00
family

1
102
6
1046325
0

 0.00
subfamily

genus

 0.00
27388
0
1
2
2

2
1
1150270

 0.00
species

genus

 0.00
6397
0
3
100
1

2
100
1
1050932
0

 0.00
no rank


 0.00
species
1
100
35632

269
33511
590186
1
1505
clade

 14.62


 0.00
phylum
32
77
1
7586
0

clade

 0.00
133550
0
8
1
1

class

 0.00
35069
0
1
1
7

1
1
6
35070
0

 0.00
subclass

7581
0
1
1
5
order

 0.00

1
1
4
0
106121

 0.00
family

subfamily

 0.00
0
1529449
3
1
1

2
1
1
1529434
0

 0.00
genus

1
1
1529436

 0.00
species


 0.00
clade
23
1
76
0
133551


 0.00
superclass
9
54
1
7587
0

0
7588
1
54
8
class

 0.00


 0.00
superorder
1
54
7
0
41242

order

 0.00
7599
0
6
1
54

1
54
5
1
7600

 0.00
family

genus

 0.00
0
7608
1
24
2


 0.00
species
1
24
7609


 0.00
genus
29
1
2
7601
0

species

 0.00
7604
1
29

22
1
13
0
7624

 0.00
superclass

12
22
1
0
7625

 0.00
class

subclass

 0.00
7638
0
11
22
1

superorder

 0.00
0
7674
22
1
10

0
2785018
5
1
6
order

 0.00


 0.00
infraorder
6
1
4
7675
0

6
1
3
0
31181

 0.00
family

genus

 0.00
7664
0
2
6
1

species

 0.00
7668
6
1

0
31184
4
16
1
order

 0.00

1
16
3
31185
0

 0.00
family

7652
0
2
16
1
genus

 0.00

1
16
7654

 0.00
species

phylum

 0.00
10219
0
5
1
1


 0.00
class
4
1
1
10220
0

1
1
3
0
10221

 0.00
family

1
1
2
10222
0

 0.00
genus


 0.00
species
1
1
10224

356
7711
1467
589839
1
phylum

 14.62

subphylum

 0.00
0
7712
1
38
22

0
7713
34
1
16
class

 0.00

order

 0.00
32436
0
1
28
4

3
1
28
201955
0

 0.00
family

genus

 0.00
201956
0
28
1
2

2771288
1
28
species

 0.00

2
1
4
7720
0

 0.00
order

1
2
3
0
7721

 0.00
family


 0.00
genus
2
1
2
0
7724

species

 0.00
7725
2
1

order

 0.00
7716
0
7
4
1

0
7717
3
1
2
family

 0.00


 0.00
genus
2
1
2
0
7718


 0.00
species
1
2
7719

0
30274
3
2
1
family

 0.00

genus

 0.00
0
56696
1
2
2

59560
2
1
species

 0.00

30302
0
4
1
5
class

 0.00

4
1
4
2507557
0

 0.00
order

3
1
4
0
41302

 0.00
family


 0.00
genus
4
1
2
34763
0

34765
4
1
species

 0.00


 0.00
subphylum
8
27
1
7735
0


 0.00
class
1
27
7
0
2682552

order

 0.00
2682553
0
6
27
1

27
1
5
7736
0

 0.00
family

genus

 0.00
0
7737
27
1
4

3
1
7739

 0.00
species

species

 0.00
7741
1
1

species

 0.00
7740
23
1

subphylum

 14.61
0
89593
1436
1
589418


 14.61
clade
1
589418
1435
44
7742

1422
589360
1
7776
220

 14.60
clade

0
117570
589018
1
1385
clade

 14.60


 14.60
clade
589018
1
1384
117571
13280

8287
11
866
1
564396
superclass

 13.99

clade

 0.00
118072
0
5
1
5

7894
0
4
5
1
order

 0.00

family

 0.00
7895
0
3
5
1

genus

 0.00
0
7896
5
1
2


 0.00
species
1
5
7897

clade

 13.99
1338369
6
564380
1
860

subclass

 0.00
0
7878
7
1
2

6
1
2
0
118077

 0.00
superorder

1
2
5
0
2823314

 0.00
order

0
7879
2
1
4
suborder

 0.00

family

 0.00
7884
0
3
1
2

genus

 0.00
7885
0
2
1
2

species

 0.00
7888
2
1


 13.98
clade
1
564372
852
259
32523

47
1
98
0
8292

 0.00
class

0
8445
10
79
1
order

 0.00

30380
0
3
1
43
family

 0.00

genus

 0.00
0
194407
2
1
43

194408
43
1
species

 0.00

family

 0.00
1277737
0
16
1
3

264009
0
1
16
2
genus

 0.00

1415580
1
16
species

 0.00

family

 0.00
264006
0
3
1
20


 0.00
genus
1
20
2
260994
0

species

 0.00
260995
20
1


 0.00
superorder
36
1
19
0
41666

order

 0.00
8342
0
16
1
29

7
1
8
0
30319

 0.00
superfamily

family

 0.00
0
8352
1
7
7


 0.00
subfamily
6
1
7
0
8360

7
1
5
8353
1

 0.00
genus


 0.00
subgenus
2
2
1
262014
0

species

 0.00
8355
1
2

subgenus

 0.00
8363
0
4
1
2

species

 0.00
8364
1
4


 0.00
suborder
20
1
9
8416
0

0
8417
10
6
1
superfamily

 0.00


 0.00
family
1
3
3
0
192735

genus

 0.00
8419
0
2
3
1

3
1
248795

 0.00
species

8382
0
2
2
1
family

 0.00

8383
1
2
genus

 0.00

family

 0.00
611790
0
4
1
1


 0.00
subfamily
1
1
3
611792
0


 0.00
genus
2
1
1
0
449102

448589
1
1
species

 0.00

superfamily

 0.00
0
30352
9
3
1

family

 0.00
0
8397
2
1
4

8399
0
3
1
2
genus

 0.00

2
1
2
0
121175

 0.00
subgenus


 0.00
species
1
2
8407

0
685130
1
1
4
family

 0.00

0
1191377
1
1
3
subfamily

 0.00

0
30356
1
1
2
genus

 0.00


 0.00
species
1
1
30357

order

 0.00
0
8293
6
1
3


 0.00
superfamily
1
3
5
30364
0

4
3
1
30366
0

 0.00
family

3
1
3
36311
0

 0.00
genus

0
1527435
2
1
3
subgenus

 0.00

1
3
429345

 0.00
species

2044
32524
804
1
564015
clade

 13.98

1
560099
489
40674
41

 13.88
class


 13.88
clade
1
560051
480
120
32525

clade

 13.87
9347
315
559885
1
457

superorder

 0.00
9348
0
1
12
10

5
10
1
948950
0

 0.00
order

0
948953
10
1
4
suborder

 0.00

1
10
3
227508
0

 0.00
family

1
10
2
9357
0

 0.00
genus

27675
1
10
species

 0.00

order

 0.00
948951
0
1
2
4

9359
0
3
2
1
family

 0.00

2
1
2
0
9360

 0.00
genus

9361
1
2
species

 0.00

clade

 13.87
3921
1437010
421
1
559539

209
3437
1
207
314145

 0.09
superorder


 0.05
order
1838
1
70
56
91561

21
143
1
2653789
0

 0.00
suborder

9721
6
20
1
143
infraorder

 0.00

36
1
4
9761
0

 0.00
parvorder


 0.00
family
36
1
3
9765
0

0
9766
1
36
2
genus

 0.00

1
36
9771

 0.00
species

15
101
1
9722
10

 0.00
parvorder


 0.00
family
3
6
1
0
9740


 0.00
genus
2
6
1
0
9741


 0.00
species
1
6
42100

9747
0
1
1
3
family

 0.00

1
1
2
0
9748

 0.00
genus


 0.00
species
1
1
9749

family

 0.00
119500
0
3
1
3

118796
0
1
3
2
genus

 0.00

species

 0.00
118797
3
1

9726
0
81
1
5
family

 0.00

0
9738
3
1
2
genus

 0.00

species

 0.00
9739
3
1

0
9732
2
78
1
genus

 0.00

1
78
9733

 0.00
species

suborder

 0.03
9845
0
1
1318
33

infraorder

 0.03
35500
168
32
1318
1

22
912
1
114
9895

 0.02
family

subfamily

 0.00
0
9959
3
1
1

genus

 0.00
9957
0
1
1
2


 0.00
species
1
1
59534

subfamily

 0.01
6
9963
1
448
7


 0.00
genus
2
1
3
9922
0

9925
1
3
species

 0.00

9935
7
4
439
1
genus

 0.01


 0.01
species
2
409
1
0
37174

112262
1
409
subspecies

 0.01

9940
23
1
species

 0.00

subfamily

 0.01
9
27592
11
349
1


 0.01
genus
1
329
5
9903
235


 0.00
species
4
1
1705790

species

 0.00
30522
3
1

46
1
9913

 0.00
species

72004
41
1
species

 0.00

0
9900
3
1
5
genus

 0.00

0
9901
5
1
2
species

 0.00

subspecies

 0.00
43346
5
1


 0.00
genus
2
6
1
9918
0


 0.00
species
6
1
89462

family

 0.01
1
9850
238
1
9

subfamily

 0.00
0
9881
1
2
4

2
1
3
0
9871

 0.00
genus


 0.00
species
2
1
2
9874
0

1
2
9880

 0.00
subspecies

34878
0
1
235
4
subfamily

 0.01


 0.01
genus
1
235
3
9859
3


 0.00
species
1
1
1574408

species

 0.01
9860
1
231

suborder

 0.00
9834
0
1
17
8

family

 0.00
1
9835
7
1
17

genus

 0.00
0
30539
1
6
2

30538
6
1
species

 0.00

1
10
4
9836
1

 0.00
genus

9838
1
3
species

 0.00


 0.00
species
1
1
9837


 0.00
species
1
5
419612


 0.01
suborder
1
304
7
1
35497


 0.01
family
303
1
6
9821
11

0
9822
3
1
291
genus

 0.01


 0.01
species
291
1
2
169
9823


 0.00
subspecies
122
1
415978

1
1
2
0
41425

 0.00
genus

species

 0.00
41426
1
1

4
6
1
9971
0

 0.00
order

family

 0.00
0
9972
6
1
3

9973
0
2
6
1
genus

 0.00

143292
1
6
species

 0.00

21
33554
1094
1
65
order

 0.03

suborder

 0.00
379583
0
21
126
1

3
9681
1
114
14
family

 0.00

subfamily

 0.00
338153
0
3
4
1


 0.00
genus
2
1
4
9688
0

species

 0.00
29064
4
1


 0.00
subfamily
1
105
7
0
338152

genus

 0.00
0
46841
1
1
2

1
1
46844

 0.00
species

genus

 0.00
0
37028
1
1
2

species

 0.00
61388
1
1

1
103
2
0
9682

 0.00
genus

9685
1
103
species

 0.00

1
2
3
338151
0

 0.00
subfamily

2
2
1
32535
0

 0.00
genus

species

 0.00
32536
2
1


 0.00
family
3
5
1
0
9697

0
37031
2
1
5
genus

 0.00

1
5
37032

 0.00
species

3
1
7
0
9676

 0.00
family


 0.00
genus
2
7
1
0
95911

95912
7
1
species

 0.00


 0.02
suborder
947
1
43
172
379584

family

 0.00
0
9632
11
1
4

genus

 0.00
9639
5
3
1
11

species

 0.00
29073
3
1

3
1
9643

 0.00
species

family

 0.00
9702
0
2
1
3

9703
0
1
2
2
genus

 0.00


 0.00
species
1
2
9704


 0.01
family
21
261
1
9655
35

subfamily

 0.00
0
169418
1
12
5

genus

 0.00
0
9665
12
1
4

1
9
2
0
9668

 0.00
species

subspecies

 0.00
9669
9
1


 0.00
species
3
1
36723

0
2892069
11
1
4
subfamily

 0.00

genus

 0.00
0
48419
3
1
11

1
11
2
0
48420

 0.00
species

11
1
2888765

 0.00
subspecies

0
169417
8
1
75
subfamily

 0.00


 0.00
genus
1
72
2
9656
0

9657
1
72
species

 0.00


 0.00
genus
1
1
2
71112
0


 0.00
species
1
1
76717

2
1
3
34881
0

 0.00
genus

2
1
2
0
34882

 0.00
species


 0.00
subspecies
1
2
391180

subfamily

 0.00
1008252
0
3
128
1

1
128
2
9661
0

 0.00
genus

species

 0.00
9662
1
128

family

 0.01
3
9608
1
488
5


 0.00
genus
1
1
9625

0
9611
3
484
1
genus

 0.01


 0.01
species
1
484
2
9612
445

subspecies

 0.00
9615
1
39

7
9709
5
1
12
family

 0.00


 0.00
genus
2
4
1
0
9712

9713
4
1
species

 0.00

2
1
1
0
9710

 0.00
genus

1
1
9711

 0.00
species

4
1
1
9705
0

 0.00
family

3
1
1
0
9706

 0.00
genus

2
1
1
0
9707

 0.00
species


 0.00
subspecies
1
1
9708

9362
0
1
19
13
order

 0.00

0
9376
7
13
1
family

 0.00

183663
0
3
1
10
subfamily

 0.00

9379
0
2
10
1
genus

 0.00

species

 0.00
42254
10
1

3
1
3
183662
0

 0.00
subfamily


 0.00
genus
2
3
1
0
9377

109475
1
3
species

 0.00

family

 0.00
0
9373
1
6
5

143301
0
2
1
3
genus

 0.00

143302
1
3
species

 0.00

1
3
2
9374
0

 0.00
genus

species

 0.00
50954
3
1


 0.00
order
1
43
12
1
9787

7
40
1
9788
0

 0.00
family


 0.00
genus
6
40
1
9789
6

species

 0.00
9796
1
29

species

 0.00
9798
1
2


 0.00
species
2
1
9793

89248
0
2
1
1
species

 0.00


 0.00
subspecies
1
1
89252

2
1
4
9803
0

 0.00
family

9806
0
3
2
1
genus

 0.00

species

 0.00
9807
0
1
2
2

1
2
73337

 0.00
subspecies


 0.01
order
230
1
44
1
9397

9
24
1
0
30559

 0.00
suborder

9398
0
8
1
24
family

 0.00

77225
2
24
1
7
subfamily

 0.00

9406
0
2
1
6
genus

 0.00


 0.00
species
6
1
9407

genus

 0.00
9401
4
1
16
4

species

 0.00
132908
3
1


 0.00
species
7
1
143291

species

 0.00
9402
1
2

2
30560
1
205
34
suborder

 0.01

1
170
12
2
9431

 0.00
family

0
27671
3
1
146
genus

 0.00

144
1
59474

 0.00
species

species

 0.00
59472
1
2


 0.00
genus
2
1
2
29077
0

species

 0.00
29078
2
1

0
981671
1
9
3
subfamily

 0.00

1
9
2
0
9432

 0.00
genus

291302
1
9
species

 0.00

genus

 0.00
6
9434
11
1
3

1
2
59463

 0.00
species

51298
1
3
species

 0.00


 0.00
family
13
1
21
2
9415

3
4
1
0
40237

 0.00
subfamily

2
4
1
0
9429

 0.00
genus

4
1
9430

 0.00
species


 0.00
subfamily
5
6
1
1
40234


 0.00
genus
4
1
2
9416
0


 0.00
species
4
1
9417

genus

 0.00
0
27659
1
1
2

1
1
192404

 0.00
species

0
40238
9
1
4
subfamily

 0.00

genus

 0.00
0
9422
3
9
1

1
7
89673

 0.00
species


 0.00
species
1
2
9423

5
1
8
0
58055

 0.00
family

4
8
1
0
186995

 0.00
subfamily

1
8
3
49442
2

 0.00
genus

4
1
89399

 0.00
species

2
1
59479

 0.00
species

family

 0.00
186994
0
4
1
3

genus

 0.00
0
58068
4
1
2

4
1
186990

 0.00
species

superorder

 13.68
314146
2660
1
552181
211

74
5360
1
9443
3

 0.13
order

5334
1
58
0
376913

 0.13
suborder

314293
26
53
5333
1
infraorder

 0.13

40
1
5296
32
9526

 0.13
parvorder

18
5242
1
15
314295

 0.13
superfamily

2
1
3
0
9577

 0.00
family

genus

 0.00
9578
0
2
2
1


 0.00
species
2
1
81572

family

 0.13
35
9604
5225
1
14


 0.13
subfamily
9
1
5182
207598
286

2
1
4848
9605
0

 0.12
genus

4848
1
9606

 0.12
species

2
1
3
0
9592

 0.00
genus

species

 0.00
9593
0
2
1
2

9595
2
1
subspecies

 0.00

9596
0
46
1
3
genus

 0.00

7
1
9597

 0.00
species

species

 0.00
9598
1
39

subfamily

 0.00
0
607660
4
8
1

9599
0
3
8
1
genus

 0.00


 0.00
species
1
1
9600

species

 0.00
9601
1
7

22
1
21
314294
0

 0.00
superfamily


 0.00
family
20
22
1
0
9527

1
20
13
9528
6

 0.00
subfamily

genus

 0.00
9529
0
2
1
1

9531
1
1
species

 0.00


 0.00
genus
1
3
2
9554
0

species

 0.00
9555
3
1

0
392815
3
1
5
genus

 0.00

9534
1
1
species

 0.00

60711
4
1
species

 0.00

genus

 0.00
9539
0
1
5
5

species

 0.00
54602
0
2
1
1

257877
1
1
subspecies

 0.00

1
1
9541

 0.00
species


 0.00
species
1
3
9544

6
1
2
9569
0

 0.00
subfamily


 0.00
genus
1
1
2
0
54136

54180
1
1
species

 0.00


 0.00
genus
1
1
3
9570
0


 0.00
species
1
1
2
54131
0

336983
1
1
subspecies

 0.00

parvorder

 0.00
3
9479
11
1
12

8
6
1
1
9498

 0.00
family


 0.00
subfamily
1
3
4
0
9480

0
9481
3
1
3
genus

 0.00

subgenus

 0.00
0
1965096
1
3
2

1
3
9483

 0.00
species


 0.00
subfamily
3
2
1
1
38070


 0.00
genus
2
1
1
1532884
0

9515
1
1
species

 0.00

376918
0
1
2
3
family

 0.00

genus

 0.00
9504
0
2
1
2


 0.00
species
2
1
37293

0
376912
1
1
4
infraorder

 0.00

family

 0.00
0
9475
1
1
3

1
1
2
0
1868481

 0.00
genus

1
1
1868482

 0.00
species


 0.00
suborder
15
1
23
376911
0

infraorder

 0.00
0
376915
10
1
16

family

 0.00
0
30599
3
1
1

2
1
1
30600
0

 0.00
genus


 0.00
species
1
1
379532

1
9
3
0
9445

 0.00
family

genus

 0.00
9446
0
1
9
2

1
9
9447

 0.00
species


 0.00
family
6
1
3
30615
0


 0.00
genus
1
6
2
0
13149

1
6
30608

 0.00
species

0
376917
4
1
7
infraorder

 0.00


 0.00
family
1
7
3
40297
0

7
1
2
0
30610

 0.00
genus

species

 0.00
30611
1
7

1
544152
127
54
314147

 13.48
clade

8
1
8
9975
0

 0.00
order

4
6
1
0
9976

 0.00
family

genus

 0.00
0
9977
3
6
1

9978
1
4
species

 0.00

130825
2
1
species

 0.00

1
2
3
9979
0

 0.00
family

2
1
2
9984
0

 0.00
genus

species

 0.00
9986
1
2

544090
1
118
2729
9989

 13.48
order

0
33553
1
113
17
suborder

 0.00

55153
2
1
113
16
family

 0.00

80
1
5
9991
0

 0.00
subfamily

337752
1
1
80
4
tribe

 0.00

19
10001
3
79
1
genus

 0.00

30640
1
44
species

 0.00


 0.00
species
16
1
55149

subfamily

 0.00
337726
0
10
31
1


 0.00
tribe
9
1
31
6
337730

11
1
2
1141640
0

 0.00
genus


 0.00
species
1
11
43179

genus

 0.00
0
1141645
6
1
2

6
1
9999

 0.00
species

4
1
8
9992
1

 0.00
genus

9993
0
6
1
2
species

 0.00


 0.00
subspecies
6
1
9994

9995
1
1
species

 0.00

suborder

 13.40
331
1963758
72
540925
1


 0.00
superfamily
5
2
1
1963761
0

family

 0.00
30648
0
2
1
4

1
2
3
0
35737

 0.00
subfamily

2
1
2
0
48867

 0.00
genus

51337
1
2
species

 0.00

66
540592
1
337687
25645

 13.40
clade


 0.02
family
23
1
985
98
337677

8
148
1
39087
19

 0.00
subfamily

15
1
3
1
10053

 0.00
genus

species

 0.00
79684
5
1

100897
9
1
species

 0.00

1
102
2
10049
0

 0.00
genus


 0.00
species
102
1
1047088

genus

 0.00
447134
0
1
12
2

species

 0.00
447135
12
1

subfamily

 0.00
7
10026
45
1
7

2
1
10
10043
0

 0.00
genus

1
10
109678

 0.00
species

10035
0
1
11
2
genus

 0.00

11
1
10036

 0.00
species


 0.00
genus
2
17
1
10028
0

species

 0.00
10029
17
1

7
1
694
6
337963

 0.02
subfamily

10040
2
1
29
4
genus

 0.00

2
1
16
1
10042

 0.00
species

subspecies

 0.00
230844
1
15


 0.00
species
1
11
10041

0
38667
2
1
659
genus

 0.02

species

 0.02
38674
1
659

10066
20931
513947
1
38
family

 12.74


 0.00
subfamily
3
1
17
0
10045


 0.00
genus
2
17
1
10046
0

10047
17
1
species

 0.00

406
39107
2476
1
23
subfamily

 0.06

genus

 0.00
10114
6
3
75
1


 0.00
species
1
1
10117

68
1
10116

 0.00
species


 0.00
genus
5
1
2
0
61153

5
1
61156

 0.00
species

30639
0
14
1
2
genus

 0.00

14
1
35658

 0.00
species

10
1
1763
5
10088

 0.04
genus

0
862508
2
1
4
subgenus

 0.00

10093
1
4
species

 0.00

1754
1
7
228
862507

 0.04
subgenus

1
21
10096

 0.00
species

1
1490
4
10090
1478

 0.04
species

subspecies

 0.00
10092
1
5

subspecies

 0.00
57486
1
4

10091
3
1
subspecies

 0.00

10089
15
1
species

 0.00

0
10128
3
1
209
genus

 0.01

1
209
2
0
400053

 0.01
species group


 0.01
species
209
1
10129

genus

 0.00
121588
0
1
4
2

491861
4
1
species

 0.00


 12.16
subfamily
11
490523
1
1
326408

genus

 12.15
10067
91536
10
1
490522

1
3136
83527

 0.08
species


 0.00
species
4
1
2866131


 0.00
species
1
105
92878


 0.00
species
201
1
10068

60744
1
69050
species

 1.71

32520
1
83762

 0.81
species

1
1
2
0
2642879

 0.00
no rank


 0.00
species
1
1
60743

60746
293969
1
species

 7.28


 0.00
family
4
15
1
0
337664

0
10061
1
15
3
subfamily

 0.00

0
30636
15
1
2
genus

 0.00

species

 0.00
1026970
15
1

suborder

 0.00
1963757
1
13
54
1

3
10015
9
1
34
family

 0.00


 0.00
subfamily
4
1
5
0
38663

3
1
5
0
37442

 0.00
genus


 0.00
species
5
1
2
0
38669

214514
1
5
subspecies

 0.00

4
1
26
38662
0

 0.00
subfamily

genus

 0.00
21
10016
3
1
26

species

 0.00
10020
1
1

105255
1
4
species

 0.00

0
29132
19
1
3
family

 0.00

10184
0
1
19
2
genus

 0.00


 0.00
species
19
1
51338

suborder

 0.01
33550
1
269
1
15

258
1
5
0
10167

 0.01
family

256
1
2
10180
0

 0.01
genus

10181
256
1
species

 0.01

423606
0
1
2
2
genus

 0.00


 0.00
species
1
2
885580

family

 0.00
10139
0
3
1
3

0
10140
3
1
2
genus

 0.00


 0.00
species
1
3
10141

10150
0
1
5
3
family

 0.00

10151
0
2
5
1
genus

 0.00

1
5
34839

 0.00
species

10158
0
1
2
3
family

 0.00

0
10159
2
1
2
genus

 0.00

2
1
10160

 0.00
species


 0.00
order
5
8
1
9392
0


 0.00
family
4
1
8
0
9393

genus

 0.00
0
9394
3
8
1

1
6
37347

 0.00
species


 0.00
species
2
1
246437

30656
0
4
1
1
order

 0.00

1
1
3
30657
0

 0.00
family

482536
0
2
1
1
genus

 0.00


 0.00
species
1
1
482537

19
1
25
311790
0

 0.00
superorder

0
28734
1
1
4
order

 0.00

3
1
1
0
28735

 0.00
family

genus

 0.00
28736
0
1
1
2

species

 0.00
28737
1
1

order

 0.00
9774
0
3
1
5

1
3
4
0
9775

 0.00
family

9776
0
3
1
3
genus

 0.00

2
3
1
9778
0

 0.00
species

127582
3
1
subspecies

 0.00

9779
0
7
1
4
order

 0.00

1
9780
6
1
4
family

 0.00


 0.00
genus
2
1
1
0
9784


 0.00
species
1
1
9785

0
9782
1
2
3
genus

 0.00

species

 0.00
0
9783
2
1
2

subspecies

 0.00
99487
1
2

family

 0.00
0
9369
10
1
4

10
1
3
176113
0

 0.00
subfamily

genus

 0.00
9370
0
2
10
1

9371
10
1
species

 0.00

order

 0.00
0
9815
1
1
4

9816
0
3
1
1
family

 0.00


 0.00
genus
2
1
1
9817
0

species

 0.00
9818
1
1

22
1
46
9263
1

 0.00
clade

order

 0.00
0
38609
1
13
10

9335
0
3
2
1
family

 0.00

9336
0
2
1
2
genus

 0.00

2
1
9337

 0.00
species

family

 0.00
0
9338
3
6
1


 0.00
genus
2
1
6
0
29138

6
1
29139

 0.00
species

38624
0
5
1
3
family

 0.00

0
38625
2
1
5
genus

 0.00

species

 0.00
38626
5
1

38608
0
4
1
21
order

 0.00

9277
0
3
1
21
family

 0.00


 0.00
genus
21
1
2
0
9304

1
21
9305

 0.00
species

order

 0.00
0
38605
11
1
7

9265
0
6
11
1
family

 0.00

126287
0
5
11
1
subfamily

 0.00

13615
0
1
10
2
genus

 0.00

species

 0.00
13616
1
10

genus

 0.00
0
126288
2
1
1


 0.00
species
1
1
191870


 0.00
clade
7
1
8
0
9254

7
7
1
0
9255

 0.00
order

family

 0.00
0
9256
3
1
4

2
4
1
0
9257

 0.00
genus


 0.00
species
1
4
9258


 0.00
family
3
1
3
9259
0


 0.00
genus
1
3
2
9260
0


 0.00
species
3
1
9261

8457
0
314
1
1872
clade

 0.05

clade

 0.05
32561
32
313
1
1872


 0.04
clade
1687
1
249
6
1329799

clade

 0.04
8492
1
216
1
1640

436486
0
209
1631
1
clade

 0.04

0
436489
1
1631
208
clade

 0.04

clade

 0.04
436491
0
1
1631
207

0
436492
1
1631
206
clade

 0.04

1631
1
205
8782
21

 0.04
class

infraclass

 0.04
8825
54
185
1506
1

0
30458
7
3
1
order

 0.00

0
30462
2
1
3
family

 0.00

56312
0
2
1
2
genus

 0.00


 0.00
species
2
1
56313

3
1
1
30459
0

 0.00
family

2
1
1
0
57440

 0.00
genus

species

 0.00
57441
1
1


 0.00
order
7
1
3
0
8906

1
2
3
0
8907

 0.00
family

genus

 0.00
50391
0
2
2
1

50402
2
1
species

 0.00

3
1
1
8917
0

 0.00
family

2
1
1
8918
0

 0.00
genus

198806
1
1
species

 0.00

0
2607030
10
50
1
clade

 0.00

0
8892
4
1
1
order

 0.00


 0.00
family
1
1
3
9242
0

genus

 0.00
0
9243
2
1
1


 0.00
species
1
1
9244

0
8902
5
1
49
order

 0.00

1
49
4
48283
0

 0.00
family

3
49
1
48286
0

 0.00
subfamily

2
49
1
0
48284

 0.00
genus

111811
49
1
species

 0.00

9108
0
4
1
1
order

 0.00

family

 0.00
0
54375
1
1
3

172679
0
2
1
1
genus

 0.00

species

 0.00
187382
1
1

56308
0
2
1
4
order

 0.00


 0.00
family
1
2
3
0
56309


 0.00
genus
2
1
2
0
57396


 0.00
species
2
1
57397

10
1
106
2558200
0

 0.00
order

9
106
1
56259
0

 0.00
family

106
1
8
29
8955

 0.00
subfamily

44
1
2
0
8956

 0.00
genus

8957
44
1
species

 0.00

0
8968
2
1
1
genus

 0.00

52644
1
1
species

 0.00

8960
0
1
32
3
genus

 0.00

8962
0
2
32
1
species

 0.00

32
1
223781

 0.00
subspecies

4
1
1
56301
0

 0.00
order

family

 0.00
56302
0
1
1
3

genus

 0.00
0
56303
2
1
1


 0.00
species
1
1
121530

30449
0
4
7
1
order

 0.00

subfamily

 0.00
0
85545
1
7
3

52123
0
1
7
2
genus

 0.00

52124
1
7
species

 0.00

0
9223
7
1
9
order

 0.00


 0.00
family
7
1
3
9224
0

7
1
2
0
35547

 0.00
genus

1
7
2489341

 0.00
species

family

 0.00
1545690
0
1
2
3

0
13145
2
1
2
genus

 0.00

species

 0.00
13146
2
1

order

 0.01
9126
13
226
1
68

superfamily

 0.00
175121
10
1
27
19

3
4
1
0
9158

 0.00
family


 0.00
genus
4
1
2
0
9159


 0.00
species
1
4
9160

family

 0.00
0
9133
4
1
7

subfamily

 0.00
37599
0
7
1
3

0
9134
2
1
7
genus

 0.00

species

 0.00
9135
7
1

family

 0.00
0
37611
7
2
1


 0.00
subfamily
6
1
2
40155
0

genus

 0.00
40156
0
1
1
3

40157
0
2
1
1
species

 0.00

subspecies

 0.00
299123
1
1


 0.00
genus
1
1
2
0
59728

species

 0.00
59729
1
1


 0.00
family
4
1
4
36256
0


 0.00
genus
3
4
1
0
45806


 0.00
species
2
4
1
45807
0

4
1
1094192

 0.00
subspecies

family

 0.00
9170
0
3
1
3


 0.00
genus
1
3
2
0
9171

species

 0.00
9172
1
3

22
1
9
114313
3

 0.00
family

0
114328
2
1
1
genus

 0.00


 0.00
species
1
1
114329

0
415027
7
1
2
genus

 0.00

species

 0.00
415028
1
7

88178
0
1
1
2
genus

 0.00

649802
1
1
species

 0.00

196026
0
1
10
2
genus

 0.00

296741
1
10
species

 0.00


 0.00
family
5
5
1
0
28728

genus

 0.00
0
28729
1
2
2

1
2
164674

 0.00
species

2
3
1
0
478634

 0.00
genus

478635
1
3
species

 0.00

family

 0.00
36291
0
1
2
3

36292
0
2
2
1
genus

 0.00

59894
1
2
species

 0.00

superfamily

 0.00
2116661
0
6
1
51

5
51
1
0
36270

 0.00
family


 0.00
subfamily
4
51
1
0
330750

0
39620
1
51
3
genus

 0.00

51
1
2
0
48156

 0.00
species

126889
51
1
subspecies

 0.00

family

 0.00
0
9183
85
1
5

83
1
2
0
36283

 0.00
genus

37610
1
83
species

 0.00

genus

 0.00
9184
0
1
2
2

2
1
91951

 0.00
species


 0.00
superfamily
6
1
6
192204
0

family

 0.00
0
28725
1
5
2

5
1
30420

 0.00
genus

1
1
3
0
9143

 0.00
family


 0.00
genus
2
1
1
0
9144

1
1
9145

 0.00
species

3
3
1
0
1729112

 0.00
family

0
44387
3
1
2
genus

 0.00

species

 0.00
44394
3
1

8
1
5
3
9153

 0.00
family

1
3
2
9154
0

 0.00
genus

9157
3
1
species

 0.00

2
1
2
0
156562

 0.00
genus

2
1
156563

 0.00
species

0
400783
3
1
1
family

 0.00


 0.00
genus
1
1
2
0
87174

87175
1
1
species

 0.00

order

 0.00
8948
0
4
7
1

8949
0
3
1
7
family

 0.00

genus

 0.00
6
8952
2
7
1


 0.00
species
1
1
148594

order

 0.02
0
8929
6
752
1

8930
2
5
752
1
family

 0.02

2
747
1
0
36242

 0.02
genus

1
747
177155

 0.02
species

genus

 0.00
0
8931
3
1
2

1
3
8932

 0.00
species

7
1
4
0
8920

 0.00
order

3
1
7
56295
0

 0.00
family


 0.00
genus
2
7
1
56296
0

7
1
240206

 0.00
species


 0.01
superorder
31
240
1
5
1549675


 0.00
order
1
166
20
3
8976

0
8990
2
1
3
family

 0.00

genus

 0.00
8995
0
1
2
2

2
1
8996

 0.00
species

16
1
161
6
9005

 0.00
family

1
32
3
0
466544

 0.00
subfamily

genus

 0.00
0
9090
32
1
2

93934
32
1
species

 0.00

1
79
3
0
466552

 0.00
subfamily

1
79
2
9102
0

 0.00
genus

species

 0.00
9103
1
79

subfamily

 0.00
9072
0
5
1
37

genus

 0.00
9053
0
2
1
1

species

 0.00
9054
1
1


 0.00
genus
2
36
1
0
9030


 0.00
species
1
36
9031

4
7
1
466585
1

 0.00
subfamily

3
1
6
30409
1

 0.00
genus


 0.00
species
4
1
30410

species

 0.00
64668
1
1

order

 0.00
8826
0
10
1
69

8830
3
9
1
69
family

 0.00

subfamily

 0.00
2068722
5
1
26
5

1
1
2
8867
0

 0.00
genus

species

 0.00
8868
1
1

8842
0
2
20
1
genus

 0.00

8845
1
20
species

 0.00

40
1
3
2068716
0

 0.00
subfamily

8835
0
1
40
2
genus

 0.00


 0.00
species
40
1
8839

0
9205
36
1
7
order

 0.00

0
30444
1
1
3
family

 0.00

genus

 0.00
0
33617
2
1
1

species

 0.00
36300
1
1

family

 0.00
33574
0
35
1
3

35
1
2
128389
0

 0.00
genus

1
35
128390

 0.00
species


 0.00
order
1
2
7
0
8936

3
1
1
57383
0

 0.00
family

genus

 0.00
0
57405
1
1
2

1
1
176938

 0.00
species

57386
0
3
1
1
family

 0.00

57420
0
2
1
1
genus

 0.00

57421
1
1
species

 0.00

19
1
104
8783
0

 0.00
superorder


 0.00
order
4
1
4
0
8784

0
8788
1
4
3
family

 0.00

genus

 0.00
8789
0
1
4
2

species

 0.00
8790
4
1

order

 0.00
8802
0
4
1
1


 0.00
family
3
1
1
8803
0

1
1
2
0
8806

 0.00
genus

30464
1
1
species

 0.00

order

 0.00
8798
0
5
4
1

8799
0
1
4
4
family

 0.00

0
8800
4
1
3
genus

 0.00

4
1
2
0
8801

 0.00
species


 0.00
subspecies
4
1
441894


 0.00
order
1
95
5
0
8819

family

 0.00
8820
0
4
95
1


 0.00
genus
3
1
95
8821
4

2696672
0
91
1
2
species

 0.00


 0.00
subspecies
91
1
202946

order

 0.00
1294634
1
8
1
6

5
1
7
0
1294636

 0.00
family

subfamily

 0.00
0
34915
4
1
7


 0.00
genus
3
1
7
0
8495

species

 0.00
38654
4
1


 0.00
species
3
1
8496

0
2841271
41
1
32
subclass

 0.00

31
41
1
8459
0

 0.00
order

0
8464
30
1
41
suborder

 0.00

4
1
8
0
1579275

 0.00
superfamily


 0.00
family
1
8
3
0
34907

204969
0
8
1
2
genus

 0.00

1
8
13735

 0.00
species


 0.00
clade
25
33
1
1
1579337

1579336
0
21
1
8
clade

 0.00

4
27791
1
21
7
superfamily

 0.00

3
1
14
0
8465

 0.00
family

2
14
1
0
8468

 0.00
genus

8469
14
1
species

 0.00

family

 0.00
27792
0
1
3
3

2
1
3
0
27793

 0.00
genus

1
3
27794

 0.00
species

0
8486
16
11
1
superfamily

 0.00

1
5
6
8487
1

 0.00
family

2
2
1
38771
1

 0.00
genus

species

 0.00
286002
1
1

904181
0
1
2
3
genus

 0.00

no rank

 0.00
1137846
0
2
1
2

106734
1
2
species

 0.00

1
4
4
3
8476

 0.00
family

8477
0
3
1
1
genus

 0.00

8479
0
1
1
2
species

 0.00

1
1
8478

 0.00
subspecies


 0.00
family
5
1
2
0
328320


 0.00
subfamily
4
2
1
328321
0

genus

 0.00
0
74925
2
1
3

1
1
260615

 0.00
species

species

 0.00
74926
1
1

153
1
63
8504
0

 0.00
class

62
1
153
0
8509

 0.00
order

clade

 0.00
0
1329961
1
153
61


 0.00
infraorder
4
3
1
8560
0


 0.00
family
3
1
3
1329929
0


 0.00
genus
1
3
2
53271
0

species

 0.00
933632
1
3

1329950
0
56
1
150
clade

 0.00

1329912
1
150
1
55
clade

 0.00

1329976
0
40
1
8
clade

 0.00

40
1
7
0
1329975

 0.00
clade

family

 0.00
8522
0
40
1
6

40
1
5
162266
1

 0.00
subfamily

genus

 0.00
42163
0
1
37
2

1
37
64176

 0.00
species


 0.00
genus
2
1
2
8523
0


 0.00
species
2
1
80427

1329911
0
46
1
109
clade

 0.00


 0.00
infraorder
93
1
25
0
8570

34979
0
1
12
4
superfamily

 0.00

34984
0
3
1
12
family

 0.00


 0.00
genus
2
12
1
0
37579


 0.00
species
1
12
176946

81
1
20
34989
0

 0.00
superfamily

family

 0.00
8602
0
4
3
1

42167
2
3
1
3
subfamily

 0.00


 0.00
genus
1
1
2
8672
0

8673
1
1
species

 0.00


 0.00
family
7
1
56
8689
0

subfamily

 0.00
0
8690
3
1
46

genus

 0.00
0
8703
2
1
46

species

 0.00
103942
1
46

0
8710
3
10
1
subfamily

 0.00

genus

 0.00
8728
0
2
1
10

88082
10
1
species

 0.00

family

 0.00
0
8578
1
22
8

1
19
4
169862
0

 0.00
subfamily


 0.00
genus
1
19
3
34999
2

species

 0.00
35005
1
5

35019
1
12
species

 0.00

169863
0
3
1
3
subfamily

 0.00

201800
0
1
3
2
genus

 0.00


 0.00
species
3
1
94885

suborder

 0.00
8511
1
13
1
14

0
2024743
1
3
5
family

 0.00

42425
0
4
3
1
subfamily

 0.00

3
1
3
0
8518

 0.00
genus


 0.00
species
1
2
59702


 0.00
species
1
1
8520

6
1
5
83232
0

 0.00
clade


 0.00
family
6
1
4
81953
0

subfamily

 0.00
145349
0
3
6
1

1
6
2
0
52201

 0.00
genus

103695
6
1
species

 0.00

3
3
1
2024747
0

 0.00
family

genus

 0.00
0
28376
3
1
2

3
1
28377

 0.00
species

infraorder

 0.00
0
8548
1
3
6


 0.00
clade
5
3
1
1330544
0

0
1329920
4
3
1
superfamily

 0.00

3
1
3
0
8555

 0.00
family

8556
0
2
1
3
genus

 0.00

species

 0.00
61221
3
1

superclass

 0.28
22
7898
517
11342
1

5
25
1
1338366
0

 0.00
class

0
8288
4
1
25
order

 0.00

family

 0.00
1
8289
25
1
3

genus

 0.00
27686
0
1
24
2

27687
1
24
species

 0.00

class

 0.28
291
186623
1
11295
511

0
32440
13
133
1
subclass

 0.00

order

 0.00
0
7899
1
133
12

suborder

 0.00
186622
0
1
133
11


 0.00
family
5
3
1
7911
0


 0.00
subfamily
4
3
1
0
186619

3
1
3
186621
0

 0.00
tribe

7912
0
1
3
2
genus

 0.00

1
3
7913

 0.00
species

7900
0
5
130
1
family

 0.00

0
124129
130
1
4
subfamily

 0.00

0
124130
130
1
3
tribe

 0.00


 0.00
genus
2
130
1
0
7901

1
130
7906

 0.00
species


 0.27
subclass
1
10871
497
7
41665


 0.27
infraclass
10841
1
491
32443
11

152
1489341
10824
1
479
clade

 0.27

0
1489343
11
1
98
clade

 0.00

clade

 0.00
0
31089
1
98
10

9
1
98
41712
0

 0.00
order


 0.00
family
3
85
1
27723
0

genus

 0.00
0
27726
1
85
2


 0.00
species
1
85
113540

31092
0
13
1
5
family

 0.00


 0.00
genus
8
1
2
0
91732

species

 0.00
1676925
1
8

5
1
2
0
42634

 0.00
genus

5
1
42636

 0.00
species

1
10574
467
186625
1222

 0.26
no rank

186634
18
97
1876
1
cohort

 0.05

subcohort

 0.00
0
282425
14
126
1

13
126
1
32446
0

 0.00
order

suborder

 0.00
1489459
0
4
95
1

1
95
3
0
299319

 0.00
family

299320
0
1
95
2
genus

 0.00

species

 0.00
299321
95
1

suborder

 0.00
0
1489460
8
31
1

family

 0.00
55118
0
7
31
1

subfamily

 0.00
0
7948
3
15
1


 0.00
genus
1
15
2
0
7949


 0.00
species
15
1
7950

0
55119
3
1
16
subfamily

 0.00

1
16
2
34772
4

 0.00
genus

12
1
34773

 0.00
species


 0.04
subcohort
1
1732
82
18
32519

186626
33
75
1580
1
clade

 0.04


 0.00
superorder
32
1
190
186628
0

0
7991
11
1
28
order

 0.00

suborder

 0.00
3
1489739
10
1
28

1
42495
5
1
7
family

 0.00


 0.00
genus
5
1
2
42513
0

42514
1
5
species

 0.00


 0.00
genus
2
1
1
42525
0


 0.00
species
1
1
42526

0
7992
4
18
1
family

 0.00

subfamily

 0.00
0
42595
18
1
3

genus

 0.00
0
7993
18
1
2

species

 0.00
7994
1
18

1
12
5
0
8002

 0.00
order

1489620
0
4
1
12
suborder

 0.00

family

 0.00
30771
0
3
12
1

genus

 0.00
8004
0
2
12
1


 0.00
species
12
1
8005

150
1
15
0
7995

 0.00
order

150
1
14
1489793
1

 0.00
suborder

1
118
4
0
30989

 0.00
family

118
1
3
5
94992

 0.00
genus

3
1
175797

 0.00
species


 0.00
species
1
110
933932


 0.00
family
4
1
3
0
7996

2
1
4
7997
0

 0.00
genus

1
4
7998

 0.00
species

0
31013
8
1
3
family

 0.00

genus

 0.00
641818
0
8
1
2

species

 0.00
1234273
8
1

7999
0
1
19
3
family

 0.00

19
1
2
30992
0

 0.00
genus

species

 0.00
310915
1
19


 0.03
superorder
1357
1
42
0
186627


 0.03
order
1
1357
41
7952
7

suborder

 0.00
0
30725
4
49
1

1
49
3
0
278171

 0.00
family

160394
0
2
1
49
genus

 0.00

species

 0.00
135647
1
49

suborder

 0.03
37
30727
1301
1
36

0
2743745
6
6
1
family

 0.00

5
6
1
2743747
0

 0.00
subfamily

0
7958
1
3
2
genus

 0.00

7959
3
1
species

 0.00

2
1
3
75351
0

 0.00
genus

species

 0.00
75352
1
3

19
1
445
23
7953

 0.01
family

1
1
3
2743705
0

 0.00
subfamily

0
1606679
2
1
1
genus

 0.00

1606681
1
1
species

 0.00

4
2
1
2743695
0

 0.00
subfamily

3
2
1
2743697
0

 0.00
tribe

genus

 0.00
0
84644
2
1
2


 0.00
species
2
1
84645

3
1
113
0
2743693

 0.00
subfamily


 0.00
genus
113
1
2
40829
0

species

 0.00
40830
1
113

1
2743694
8
306
1
subfamily

 0.01

2
1
2
0
7956

 0.00
genus

7957
2
1
species

 0.00

genus

 0.01
7961
0
1
290
2


 0.01
species
290
1
7962


 0.00
genus
13
1
3
75365
0

11
1
75366

 0.00
species

1
2
307959

 0.00
species

family

 0.02
2743709
0
6
1
803


 0.02
subfamily
5
1
803
0
2743711

genus

 0.02
7954
104
1
803
4

162
1
1142201

 0.00
species

282
1
7955

 0.01
species


 0.01
species
1
255
242068

0
2743726
10
1
4
family

 0.00

subfamily

 0.00
0
2743731
3
10
1

genus

 0.00
51137
0
2
1
10

10
1
90988

 0.00
species


 0.00
clade
134
1
6
0
186633

1
134
5
0
29140

 0.00
order

186632
0
1
134
4
suborder

 0.00


 0.00
family
134
1
3
29142
0

2
134
1
0
29143

 0.00
genus

29144
134
1
species

 0.00

1489388
1306
369
1
7476
cohort

 0.19

123365
0
339
1
4854
clade

 0.12

338
4854
1
123366
0

 0.12
clade

0
123367
1
4854
337
clade

 0.12

1
4854
336
123368
65

 0.12
clade

8
1
107
0
1489838

 0.00
clade

clade

 0.00
0
1489841
7
1
107


 0.00
clade
6
1
107
1489843
0

5
1
107
0
8043

 0.00
order

suborder

 0.00
1489845
0
4
107
1

3
107
1
8045
0

 0.00
family

0
8048
2
107
1
genus

 0.00

8049
107
1
species

 0.00


 0.12
clade
1
4682
327
132
123369

969
1489872
320
1
4431
clade

 0.11

clade

 0.02
28
1489908
99
802
1

no rank

 0.00
0
1489909
11
79
1

30863
3
7
1
10
family

 0.00

0
80965
1
3
2
genus

 0.00

1
3
80966

 0.00
species


 0.00
genus
2
2
1
80992
0

144197
1
2
species

 0.00


 0.00
genus
2
1
2
0
80969

2
1
80972

 0.00
species

205120
0
3
69
1
family

 0.00

210631
0
2
69
1
genus

 0.00


 0.00
species
69
1
210632

0
1489910
103
1
20
superorder

 0.00

order

 0.00
1489911
0
19
103
1

103
1
18
6
8113

 0.00
family


 0.00
clade
71
1
5
319056
0

1
71
4
318559
0

 0.00
subfamily

1
71
3
318529
0

 0.00
tribe

1
71
2
0
61816

 0.00
genus


 0.00
species
71
1
63155

319095
0
26
1
12
clade

 0.00

318546
15
26
1
11
subfamily

 0.00


 0.00
tribe
4
1
5
1315725
0


 0.00
genus
5
1
3
8139
3

47969
1
1
species

 0.00


 0.00
species
1
1
8128


 0.00
tribe
3
3
1
0
319058

genus

 0.00
195936
0
3
1
2

3
1
303518

 0.00
species


 0.00
tribe
3
1
3
319069
0

3
1
2
0
32506

 0.00
genus

species

 0.00
32507
3
1


 0.01
superorder
386
1
50
1489913
7

10
1
97
0
8075

 0.00
order

0
30700
7
1
3
family

 0.00

32459
0
2
1
7
genus

 0.00

1250792
7
1
species

 0.00

family

 0.00
0
270656
1
41
3

genus

 0.00
300305
0
2
1
41

species

 0.00
300306
41
1

238703
0
3
49
1
family

 0.00


 0.00
genus
2
1
49
0
270533

446457
1
49
species

 0.00

0
76071
1
127
7
order

 0.00

1
127
6
28781
0

 0.00
suborder

47757
0
5
127
1
family

 0.00

subfamily

 0.00
0
8088
4
127
1

genus

 0.00
0
8089
127
1
3


 0.00
species
4
1
30732

8090
123
1
species

 0.00

32
155
1
1
28738

 0.00
order

11
11
1
45443
0

 0.00
suborder

7
8
1
0
28771

 0.00
family

genus

 0.00
942014
0
2
1
1

1
1
451745

 0.00
species

0
52669
2
1
2
genus

 0.00


 0.00
species
2
1
52670

326431
0
5
1
2
genus

 0.00

37003
5
1
species

 0.00

0
405002
3
1
3
family

 0.00

genus

 0.00
28779
0
2
3
1

105023
3
1
species

 0.00

suborder

 0.00
8087
0
143
1
20

8076
0
2
1
5
family

 0.00

subfamily

 0.00
0
136836
2
1
4


 0.00
tribe
2
1
3
136838
0

2
2
1
1
28741

 0.00
genus

species

 0.00
28743
1
1

5
1
3
28756
0

 0.00
family


 0.00
genus
2
5
1
0
8077

species

 0.00
8078
5
1


 0.00
family
1
128
8
0
8079

subfamily

 0.00
6
586240
7
128
1

2
1
41
0
33527

 0.00
genus

33528
1
41
species

 0.00

8082
9
1
12
2
genus

 0.00

1
3
8083

 0.00
species


 0.00
genus
2
1
69
4
8080

1
65
8081

 0.00
species

3
1
8
0
28758

 0.00
family

8
1
2
0
208332

 0.00
genus

species

 0.00
208333
1
8

1
3
5
1489919
0

 0.00
clade

0
41872
3
1
4
order

 0.00

8189
0
3
1
3
family

 0.00

0
8190
1
3
2
genus

 0.00


 0.00
species
3
1
48193

0
1489920
12
1
203
clade

 0.01

order

 0.01
1
1489921
1
203
11

0
56717
109
1
5
suborder

 0.00


 0.00
family
1
109
4
56718
0

subfamily

 0.00
0
703913
3
1
109

1
109
2
0
94311

 0.00
genus


 0.00
species
109
1
181472


 0.00
suborder
5
1
93
0
123349


 0.00
family
93
1
4
0
63826

1
93
3
0
557415

 0.00
subfamily


 0.00
genus
93
1
2
0
210581

species

 0.00
441366
93
1

1489922
129
1
1639
116
clade

 0.04


 0.00
no rank
1
173
14
2
1489923


 0.00
family
1
159
5
10
30870


 0.00
genus
2
46
1
215359
0

species

 0.00
240163
1
46

103
1
2
215357
0

 0.00
genus


 0.00
species
103
1
215358


 0.00
family
5
7
1
42148
0

0
13488
1
3
2
genus

 0.00

13489
3
1
species

 0.00


 0.00
genus
2
4
1
34815
0

34816
1
4
species

 0.00

family

 0.00
0
30869
3
5
1


 0.00
genus
5
1
2
0
75037

1
5
75038

 0.00
species


 0.01
order
384
1
4
0
1489939


 0.01
family
1
384
3
0
1204718

genus

 0.01
2
8163
1
384
2

315492
1
382
species

 0.01

order

 0.00
0
1489931
6
1
171

family

 0.00
8169
5
5
171
1


 0.00
genus
1
103
2
0
8176

1
103
8177

 0.00
species

8174
0
63
1
2
genus

 0.00

63
1
8175

 0.00
species


 0.00
order
9
6
1
1489940
0

1545897
0
6
1
8
superfamily

 0.00

family

 0.00
119486
0
3
1
2


 0.00
genus
1
2
2
119487
0

119488
2
1
species

 0.00

4
1
4
8180
0

 0.00
family

27705
2
4
1
3
genus

 0.00

27706
1
1
species

 0.00

1
1
147949

 0.00
species

31022
0
11
1
47
order

 0.00

10
1
47
0
31028

 0.00
suborder

superfamily

 0.00
32517
0
9
1
47


 0.00
family
47
1
8
0
31031

genus

 0.00
39109
0
2
1
1

species

 0.00
39110
1
1

3
1
45
1
31032

 0.00
genus

1
43
31033

 0.00
species

1
1
433685

 0.00
species


 0.00
genus
1
1
2
47144
0

species

 0.00
99883
1
1


 0.00
order
4
11
1
0
1545895

3
1
11
0
30828

 0.00
family

2
11
1
109904
0

 0.00
genus


 0.00
species
11
1
109905

14
8111
691
1
59
order

 0.02

0
1489943
9
268
1
suborder

 0.01

family

 0.01
0
30871
268
1
8


 0.01
subfamily
268
1
7
0
274794

1
268
6
12
1505891

 0.01
tribe

0
134629
2
157
1
genus

 0.00

157
1
160734

 0.00
species

3
99
1
94231
2

 0.00
genus


 0.00
species
1
1
300413

293821
1
96
species

 0.00

suborder

 0.01
8100
0
24
1
207


 0.00
infraorder
1
9
7
0
1490020

family

 0.00
0
69291
6
9
1

2
5
1
134919
0

 0.00
genus

134920
1
5
species

 0.00

genus

 0.00
69292
0
3
1
4

1
69293
2
1
4
species

 0.00

1
3
481459

 0.00
subspecies

infraorder

 0.00
1490021
0
8
1
89

4
1
4
8101
0

 0.00
family

0
181456
3
4
1
subfamily

 0.00

1
4
2
0
8102

 0.00
genus

8103
4
1
species

 0.00

0
8092
1
85
3
family

 0.00


 0.00
genus
85
1
2
61642
0

1
85
61643

 0.00
species

109
1
8
8192
1

 0.00
infraorder

0
56724
104
1
4
family

 0.00

0
181468
1
104
3
subfamily

 0.00

genus

 0.00
0
56725
104
1
2


 0.00
species
1
104
56726

family

 0.00
0
8202
1
4
3


 0.00
genus
1
4
2
433404
0


 0.00
species
4
1
433405


 0.00
suborder
22
1
13
8112
0

family

 0.00
1
8165
1
22
12

subfamily

 0.00
0
698016
1
5
4

54318
1
1
5
3
genus

 0.00

2
4
1
909700
0

 0.00
subgenus


 0.00
species
1
4
417921

3
5
1
641308
0

 0.00
subfamily

genus

 0.00
0
283033
2
5
1

species

 0.00
283035
5
1

1
11
4
0
641307

 0.00
subfamily

8166
0
3
11
1
genus

 0.00

species

 0.00
8167
8
1

species

 0.00
8168
1
3

8205
6
12
1
180
suborder

 0.00

8206
0
5
9
1
family

 0.00

35729
0
8
1
2
genus

 0.00

species

 0.00
40690
1
8


 0.00
genus
2
1
1
8207
0


 0.00
species
1
1
8208

30806
0
81
1
3
family

 0.00

52238
0
2
81
1
genus

 0.00

52239
1
81
species

 0.00

36203
0
84
1
3
family

 0.00

2
84
1
56715
0

 0.00
genus

84
1
56716

 0.00
species

0
1489928
8
1
27
order

 0.00

27
1
7
8247
0

 0.00
family


 0.00
genus
1
15
2
30799
0

56723
15
1
species

 0.00


 0.00
genus
1
3
2
202584
0

241271
3
1
species

 0.00

0
98381
1
9
2
genus

 0.00


 0.00
species
9
1
1203425


 0.01
clade
44
1
391
1489904
5

order

 0.00
0
1489906
4
5
1


 0.00
family
1
5
3
8243
0

8244
0
1
5
2
genus

 0.00


 0.00
species
1
5
8245


 0.00
no rank
10
32
1
1489905
0

family

 0.00
8184
0
3
1
17


 0.00
genus
2
17
1
8186
0

8187
1
17
species

 0.00

0
30876
14
1
3
family

 0.00

genus

 0.00
0
270536
1
14
2


 0.00
species
1
14
941984

family

 0.00
27769
0
1
1
3

13691
0
2
1
1
genus

 0.00


 0.00
species
1
1
443726

order

 0.00
8252
0
1
188
18

suborder

 0.00
30942
0
1
188
17

family

 0.00
30947
0
4
11
1

603456
0
11
1
3
subfamily

 0.00

genus

 0.00
106173
0
1
11
2

species

 0.00
244447
11
1

family

 0.00
30948
0
3
88
1


 0.00
genus
2
1
88
28828
0

28829
1
88
species

 0.00


 0.00
family
3
82
1
52902
0


 0.00
genus
2
82
1
0
52903


 0.00
species
1
82
52904

3
1
2
0
8256

 0.00
family


 0.00
genus
1
2
2
0
8266

species

 0.00
195615
1
2


 0.00
family
3
5
1
171414
0

8254
0
2
1
5
genus

 0.00

8255
5
1
species

 0.00

1489907
0
11
161
1
order

 0.00

3
59
1
173245
0

 0.00
family

173246
0
2
59
1
genus

 0.00

173247
1
59
species

 0.00

7
102
1
8157
0

 0.00
family

genus

 0.00
36211
0
94
1
2


 0.00
species
94
1
36212


 0.00
genus
4
8
1
3
8160

2
1
2
302047
0

 0.00
species

2
1
1841481

 0.00
subspecies

species

 0.00
41447
3
1

clade

 0.00
0
1489874
1
73
6

8064
0
5
1
73
order

 0.00

0
8065
1
73
4
family

 0.00

73
1
3
0
390319

 0.00
subfamily

73
1
2
289381
0

 0.00
genus

1
73
390379

 0.00
species

clade

 0.00
0
1489883
8
1
29

0
129912
7
29
1
order

 0.00

6
29
1
1489884
0

 0.00
suborder


 0.00
family
5
1
29
0
72045


 0.00
subfamily
4
1
29
129914
0

genus

 0.00
0
103719
3
29
1

161584
26
1
species

 0.00

species

 0.00
161590
1
3

clade

 0.00
1489875
0
97
1
15

order

 0.00
1489876
0
6
91
1


 0.00
suborder
91
1
5
0
1489877

1
91
4
0
83881

 0.00
family

3
91
1
0
475176

 0.00
subfamily

375763
0
2
91
1
genus

 0.00

species

 0.00
375764
1
91


 0.00
order
6
1
8
0
1489878

6
1
7
8219
0

 0.00
suborder

family

 0.00
8220
0
6
1
6

subfamily

 0.00
497678
0
1
6
5

genus

 0.00
86204
0
1
4
2

species

 0.00
409849
4
1


 0.00
genus
1
2
2
150287
0

150288
1
2
species

 0.00

8
250
1
1489885
0

 0.01
clade


 0.01
order
7
1
250
0
1489894

6
250
1
0
8224

 0.01
family

1
250
5
0
186745

 0.01
subfamily

tribe

 0.01
0
186749
250
1
4


 0.01
genus
3
1
250
8234
170


 0.00
species
1
37
8236

8240
43
1
species

 0.00

clade

 0.00
2
1489892
1
181
23

67
1
9
0
43697

 0.00
order

129918
0
2
1
4
suborder

 0.00

family

 0.00
0
43698
2
1
3

1
2
2
43699
0

 0.00
genus

43700
2
1
species

 0.00

65
1
4
129920
0

 0.00
suborder

65
1
3
94233
0

 0.00
family

94234
0
2
1
65
genus

 0.00

1
65
205130

 0.00
species

112
1
13
0
1489900

 0.00
order

0
50370
1
111
8
suborder

 0.00

64142
0
78
1
3
family

 0.00

64143
0
2
78
1
genus

 0.00


 0.00
species
78
1
64144


 0.00
family
1
33
4
270602
0

0
158449
1
33
3
subfamily

 0.00


 0.00
genus
2
33
1
0
158455

33
1
158456

 0.00
species

4
1
1
50374
0

 0.00
suborder


 0.00
family
1
1
3
0
30910


 0.00
genus
2
1
1
33789
0

215402
1
1
species

 0.00

119
1
6
0
181483

 0.00
clade

119
1
5
1490028
0

 0.00
order

family

 0.00
0
47697
4
1
119

47698
0
3
1
119
subfamily

 0.00

1
119
2
0
47699

 0.00
genus

species

 0.00
586833
1
119


 0.03
clade
1295
1
23
0
41705

order

 0.00
8007
0
1
7
4

8008
0
3
1
7
family

 0.00

genus

 0.00
0
8009
7
1
2

7
1
8010

 0.00
species

1288
1
18
0
8006

 0.03
order

56
8015
17
1288
1
family

 0.03


 0.02
subfamily
5
1
852
0
504567

1
852
4
0
27772

 0.02
genus

4
1
59861

 0.00
species

no rank

 0.02
0
2649731
848
1
2

1
848
861768

 0.02
species

subfamily

 0.01
504568
6
380
1
11


 0.00
genus
5
26
1
16
8016

5
1
8022

 0.00
species

2
1
74940

 0.00
species

species

 0.00
8018
1
1

8019
1
2
species

 0.00

genus

 0.01
8028
2
2
1
330


 0.01
species
328
1
8032


 0.00
genus
3
18
1
8033
15

8040
1
1
species

 0.00

species

 0.00
8036
1
2


 0.00
clade
21
1
6
1489798
0

41711
0
21
1
5
order

 0.00

8012
0
4
21
1
family

 0.00

subfamily

 0.00
182238
0
3
1
21


 0.00
genus
1
21
2
137519
0

species

 0.00
137520
1
21

0
1489340
11
6
1
clade

 0.00

0
186624
1
6
10
clade

 0.00

clade

 0.00
0
32521
1
6
9

order

 0.00
7933
0
4
3
1


 0.00
family
3
1
3
0
7934


 0.00
genus
2
3
1
7935
0


 0.00
species
1
3
7936

0
7925
3
1
4
order

 0.00

3
1
3
7930
0

 0.00
family

0
7931
2
1
3
genus

 0.00

118141
3
1
species

 0.00

1
23
5
1489100
0

 0.00
infraclass

7914
0
23
1
4
order

 0.00

0
7915
3
1
23
family

 0.00

7916
0
1
23
2
genus

 0.00

species

 0.00
7918
1
23

7777
0
1
122
36
class

 0.00


 0.00
subclass
30
1
121
7778
0

superorder

 0.00
117893
0
7
56
1

7858
0
56
1
6
order

 0.00


 0.00
family
5
56
1
30475
0

117861
0
1
1
2
genus

 0.00

species

 0.00
7782
1
1


 0.00
genus
2
55
1
0
117853

386614
55
1
species

 0.00


 0.00
infraclass
1
65
22
0
119203

0
119197
1
65
21
clade

 0.00

1
65
20
0
119195

 0.00
superorder


 0.00
order
5
2
1
0
30496


 0.00
family
4
2
1
7850
0

subfamily

 0.00
0
7844
3
1
2

1
2
2
13396
0

 0.00
genus


 0.00
species
2
1
13397

order

 0.00
30503
0
1
35
10

3
6
1
0
378069

 0.00
family

0
378070
1
6
2
genus

 0.00

species

 0.00
378071
6
1

40580
0
3
27
1
family

 0.00

genus

 0.00
0
34767
1
27
2

36176
27
1
species

 0.00


 0.00
family
2
1
3
259919
0

2
2
1
1849765
0

 0.00
genus


 0.00
species
1
2
259920

30483
0
1
28
4
order

 0.00

3
1
28
7826
0

 0.00
family

2
28
1
0
7829

 0.00
genus


 0.00
species
1
28
7830


 0.00
subclass
5
1
1
7863
0

1
1
4
0
7864

 0.00
order


 0.00
family
3
1
1
0
7865

genus

 0.00
7866
0
1
1
2

1
1
7868

 0.00
species

1476529
0
14
1
12
clade

 0.00

3
1
6
117565
0

 0.00
class

1
3
5
7761
0

 0.00
order

family

 0.00
0
7762
3
1
4


 0.00
subfamily
1
3
3
0
30309

2
1
3
7763
0

 0.00
genus

3
1
7764

 0.00
species

117569
0
5
1
11
class

 0.00

order

 0.00
0
7745
4
11
1


 0.00
family
1
11
3
7746
0

genus

 0.00
0
7756
2
1
11

1
11
7757

 0.00
species


 0.00
phylum
110
1
61
1
6073

0
6101
1
78
38
class

 0.00

subclass

 0.00
6102
0
29
1
72


 0.00
order
1
55
14
6103
0


 0.00
family
4
1
3
0
42822

2
1
4
6104
0

 0.00
genus


 0.00
species
4
1
6105


 0.00
family
16
1
3
0
45349


 0.00
genus
1
16
2
0
45350

species

 0.00
45351
1
16

1
4
3
0
42823

 0.00
family

1
4
2
1720308
0

 0.00
genus


 0.00
species
4
1
2652724


 0.00
suborder
1
31
4
86626
0

0
478428
1
31
3
family

 0.00


 0.00
genus
2
31
1
478394
0

1789172
1
31
species

 0.00


 0.00
order
14
1
17
0
6125


 0.00
suborder
4
1
7
123760
0


 0.00
family
3
7
1
0
46736


 0.00
genus
7
1
2
0
1920453


 0.00
species
1
7
48498

123757
0
10
1
9
suborder

 0.00

0
46729
7
1
5
family

 0.00


 0.00
genus
2
2
1
50428
0

2
1
50429

 0.00
species


 0.00
genus
2
5
1
0
46730

46731
5
1
species

 0.00

6126
0
3
3
1
family

 0.00

genus

 0.00
0
6127
2
1
3

45264
1
3
species

 0.00

1
6
8
6132
0

 0.00
subclass

3028843
order
6
7


 0.00
family
4
1
3
51108
0


 0.00
genus
2
1
4
0
51109

151771
4
1
species

 0.00

family

 0.00
86538
0
2
1
4

86539
0
3
1
2
genus

 0.00

no rank

 0.00
2653460
0
1
2
2


 0.00
species
1
2
2897299

class

 0.00
1927913
0
23
1
6

1
23
5
37528
0

 0.00
order


 0.00
suborder
23
1
4
0
1927915

0
1927917
3
1
23
family

 0.00

2
23
1
37533
0

 0.00
genus

23
1
313498

 0.00
species

8
1
16
6074
0

 0.00
class

37516
0
10
1
7
subclass

 0.00


 0.00
order
1
7
9
406427
0

4
1
4
1612408
0

 0.00
suborder

family

 0.00
6080
0
3
1
4

2
4
1
0
6083

 0.00
genus

species

 0.00
6087
1
4


 0.00
suborder
4
1
3
406428
0

3
3
1
6094
0

 0.00
family


 0.00
genus
1
3
2
6095
0

1
3
6096

 0.00
species

37521
0
1
1
5
subclass

 0.00


 0.00
order
1
1
4
0
128122


 0.00
family
1
1
3
128125
0

128123
0
2
1
1
genus

 0.00


 0.00
species
1
1
128124


 0.00
phylum
15
1
30
0
6040

30
1
14
6042
0

 0.00
class

0
1779148
5
1
3
subclass

 0.00

1
3
4
0
1779149

 0.00
order


 0.00
family
3
1
3
129258
0

genus

 0.00
0
68573
1
3
2

1
3
68574

 0.00
species


 0.00
subclass
27
1
8
1779146
0

27
1
7
6049
0

 0.00
order

0
178475
15
1
3
family

 0.00


 0.00
genus
2
1
15
0
178513

species

 0.00
400682
1
15

3
1
12
68562
0

 0.00
family

genus

 0.00
0
68563
2
1
12

12
1
68564

 0.00
species

758
1
1258
4751
7

 0.03
kingdom


 0.00
no rank
2
1
1
57731
0

1
1
175245

 0.00
species


 0.00
no rank
56
1
42
112252
0

8
1
1
0
4761

 0.00
phylum


 0.00
no rank
1
1
7
2683659
0

1
1
6
0
451435

 0.00
class


 0.00
order
1
1
5
451442
0


 0.00
no rank
1
1
4
1142503
0

genus

 0.00
0
100474
1
1
3

109871
0
1
1
2
species

 0.00

1
1
684364

 0.00
strain


 0.00
phylum
1
1
6
6029
0


 0.00
no rank
5
1
1
0
469895

family

 0.00
0
174683
4
1
1


 0.00
genus
1
1
3
0
174684

2
1
1
174685
0

 0.00
species


 0.00
strain
1
1
1354746

1913637
1
1
54
27
phylum

 0.00


 0.00
subphylum
6
1
14
0
451507

class

 0.00
2212703
0
6
1
13

4827
0
12
6
1
order

 0.00


 0.00
suborder
2
1
8
1344963
0


 0.00
family
3
1
1
0
34489

1
1
2
4830
0

 0.00
genus

105697
1
1
species

 0.00

1344955
0
1
1
4
family

 0.00

0
4842
3
1
1
genus

 0.00


 0.00
species
1
1
2
58291
0

1
1
1340429

 0.00
strain


 0.00
family
3
1
4
499202
0

2
4
1
688353
0

 0.00
genus

species

 0.00
688394
4
1

0
214504
46
1
6
subphylum

 0.00


 0.00
class
5
1
46
0
214506


 0.00
order
4
46
1
0
36750

0
36751
46
1
3
family

 0.00

2
46
1
0
1129544

 0.00
genus

species

 0.00
588596
46
1


 0.00
subphylum
1
1
6
0
1137986

1
1
5
0
2212732

 0.00
class

1
1
4
214503
0

 0.00
order

family

 0.00
4854
0
1
1
3


 0.00
genus
1
1
2
0
299330

1
1
64571

 0.00
species

713
1194
1
39
451864

 0.03
subkingdom


 0.02
phylum
1
868
526
0
4890

0
451866
1
15
12
subphylum

 0.00

147553
0
5
1
1
class

 0.00

1
1
4
37987
0

 0.00
order

1
1
3
44281
0

 0.00
family

genus

 0.00
4753
0
2
1
1

species

 0.00
38082
1
1

class

 0.00
147554
0
6
1
14

0
34346
14
1
5
order

 0.00


 0.00
family
4
1
14
4894
0


 0.00
genus
14
1
3
4895
0

species

 0.00
0
866546
2
14
1

strain

 0.00
653667
1
14


 0.02
clade
513
1
853
27
716545

709
1
423
147538
1

 0.02
subphylum

697
1
409
716546
35

 0.02
clade

clade

 0.00
0
715962
78
1
69


 0.00
class
69
1
77
147541
1


 0.00
subclass
1
27
23
451867
0

0
5014
6
1
9
order

 0.00

5
1
9
1570301
0

 0.00
family


 0.00
genus
4
9
1
5579
0

species

 0.00
5580
0
2
1
1

1
1
1043002

 0.00
strain

46634
1
8
species

 0.00

2726947
1
13
17
1
order

 0.00


 0.00
family
1
15
9
0
93133

112497
0
3
1
2
genus

 0.00

species

 0.00
112498
3
1

genus

 0.00
0
1047167
6
1
2


 0.00
species
6
1
1047171

0
2897311
1
4
2
genus

 0.00

4
1
5499

 0.00
species


 0.00
genus
2
1
2
39702
0


 0.00
species
2
1
215465

family

 0.00
668547
0
3
1
1


 0.00
genus
2
1
1
0
2072583

1
1
245834

 0.00
species

3
1
1
0
2726946

 0.00
order

452563
0
2
1
1
family

 0.00

genus

 0.00
5498
1
1

0
451868
35
1
44
subclass

 0.00

order

 0.00
0
92860
1
35
43

715340
0
1
29
32
suborder

 0.00

family

 0.00
1
28556
21
1
20

0
33194
4
1
6
genus

 0.00

1
2
2
0
101162

 0.00
species

strain

 0.00
930090
2
1

species

 0.00
45130
1
1

species

 0.00
5016
0
2
1
1

strain

 0.00
665024
1
1

genus

 0.00
5598
6
11
13
1

0
2499262
2
1
2
section

 0.00

species

 0.00
48100
1
2


 0.00
section
1
1
2
0
2499266

119953
1
1
species

 0.00

0
2499237
4
1
2
section

 0.00

species group

 0.00
187734
0
1
1
2


 0.00
species
1
1
5599

1187904
1
1
species

 0.00

1
2499258
2
2
1
section

 0.00


 0.00
species
1
1
181014

genus

 0.00
5027
0
1
2
3

0
53485
1
2
2
species

 0.00

2
1
97479

 0.00
forma


 0.00
family
1
6
7
683158
0

2
1
1
749461
0

 0.00
genus

749465
1
1
species

 0.00


 0.00
genus
2
1
1
301206
0

species

 0.00
301207
1
1

genus

 0.00
0
749880
2
1
4

species

 0.00
2802321
1
4

5020
0
1
3
3
family

 0.00

3
1
2
0
1351751

 0.00
genus


 0.00
species
1
3
13684


 0.00
suborder
1
2
7
0
1255046


 0.00
family
1
1
3
0
221678


 0.00
genus
1
1
2
125369
0

species

 0.00
1460663
1
1

family

 0.00
0
1208339
1
1
3

0
100048
1
1
2
genus

 0.00


 0.00
species
1
1
390896

3
1
4
0
717954

 0.00
family


 0.00
genus
2
1
4
741162
0

673940
4
1
species

 0.00

no rank

 0.00
159987
0
9
6
1

1
6
8
451869
0

 0.00
order

family

 0.00
1450293
0
4
1
2


 0.00
genus
3
2
1
462253
0

462254
0
2
2
1
species

 0.00

strain

 0.00
1176127
1
2

family

 0.00
0
45131
1
4
3


 0.00
genus
2
1
4
407951
0

1
4
310453

 0.00
species

194
1
380
715989
3

 0.01
clade

class

 0.00
147548
0
1
32
29

order

 0.00
0
1484953
5
1
5

4
5
1
66516
0

 0.00
family


 0.00
genus
1
5
3
0
334516

1484957
1
3
species

 0.00

species

 0.00
334518
1
2

3
1
5
5120
0

 0.00
order

family

 0.00
34371
0
4
1
3

1
3
3
0
34372

 0.00
genus


 0.00
species
2
3
1
34373
0

62690
1
3
forma specialis

 0.00

no rank

 0.00
221903
0
2
1
4


 0.00
family
1
2
3
34379
0


 0.00
genus
2
1
2
78156
0

species

 0.00
342668
2
1


 0.00
order
1
22
14
0
5178

0
2589077
3
1
1
family

 0.00

47830
0
1
1
2
genus

 0.00

1316788
1
1
species

 0.00

1
21
10
28983
0

 0.00
family

1
33196
4
16
1
genus

 0.00

12
40559
1
13
2
species

 0.00


 0.00
strain
1
1
332648

species

 0.00
1964551
2
1

3
1
2
0
38447

 0.00
genus


 0.00
species
1
3
61207

genus

 0.00
0
5179
3
2
1

species

 0.00
0
5180
1
2
2

isolate

 0.00
665079
2
1

class

 0.01
15
147550
164
1
345

0
222544
49
33
1
subclass

 0.00

0
5114
6
1
11
order

 0.00


 0.00
family
1
3
3
767018
0


 0.00
genus
2
1
3
36922
1


 0.00
species
1
2
83186

family

 0.00
399129
0
3
1
7

0
218105
1
1
4
no rank

 0.00


 0.00
genus
3
1
1
0
305399

2625752
1
0
no rank

 0.00

1
1
2029752

 0.00
species

1276216
0
2
1
2
genus

 0.00

1276217
1
2
species

 0.00


 0.00
order
10
1
11
0
639021


 0.00
family
1
1
4
0
81093


 0.00
genus
3
1
1
29849
0

0
36779
2
1
1
species

 0.00

strain

 0.00
644352
1
1

0
2528436
5
10
1
family

 0.00

0
48558
4
10
1
genus

 0.00

species

 0.00
148305
1
2

5
1
318829

 0.00
species

1578925
1
3
species

 0.00

1
11
15
5139
1

 0.00
order

family

 0.00
2609812
0
2
1
1

5144
1
1
genus

 0.00

0
35718
1
4
7
family

 0.00

1
1
3
0
1920207

 0.00
genus


 0.00
species
2
1
1
0
78579


 0.00
strain
1
1
573729

genus

 0.00
2609811
0
3
1
3

2587410
0
2
1
3
species

 0.00


 0.00
strain
1
3
578455

0
5148
1
5
5
family

 0.00

1
5
4
5140
0

 0.00
genus

40127
0
2
1
3
species

 0.00

3
1
510951

 0.00
strain

1
2
5141

 0.00
species

5
2
1
1775898
0

 0.00
order

0
1756146
1
2
4
family

 0.00

1
2
3
0
65412

 0.00
genus

1
2
2
223192
0

 0.00
species

strain

 0.00
1286976
2
1

5151
0
7
3
1
order

 0.00

6
3
1
0
5152

 0.00
family

genus

 0.00
29907
0
3
1
5


 0.00
species
1
1
2
0
29908

1
1
1397361

 0.00
strain

545650
0
2
2
1
species

 0.00

2
1
1398154

 0.00
strain

1
47
19
222545
0

 0.00
subclass

3
1
3
0
1830229

 0.00
no rank

2983819
0
2
3
1
genus

 0.00

3
1
1658444

 0.00
species

order

 0.00
0
37989
1
44
15


 0.00
family
2
1
3
1812770
0


 0.00
genus
1
2
2
0
1812772


 0.00
species
2
1
1141098


 0.00
family
8
1
41
2033035
0


 0.00
genus
1
19
3
42360
0

326647
17
1
species

 0.00

species

 0.00
326644
2
1

42308
0
20
1
2
genus

 0.00


 0.00
species
20
1
63214

326606
0
1
2
2
genus

 0.00

1
2
327061

 0.00
species

0
37990
3
1
1
family

 0.00

genus

 0.00
0
37991
2
1
1


 0.00
species
1
1
326684

no rank

 0.00
147551
0
4
1
6


 0.00
family
3
1
6
0
265081

6
1
2
0
265082

 0.00
genus

1093900
1
6
species

 0.00

11
222543
1
244
91
subclass

 0.01

21
5125
214
1
73
order

 0.01

5129
0
7
1
34
family

 0.00

5543
1
6
1
34
genus

 0.00


 0.00
species
1
1
101201


 0.00
species
1
3
317029

29875
1
3
species

 0.00

species

 0.00
63577
12
1


 0.00
species
1
14
51453

0
103887
1
2
3
family

 0.00

2
1
2
241409
0

 0.00
genus

1094350
2
1
species

 0.00

162454
0
5
1
3
no rank

 0.00


 0.00
genus
2
1
2
0
159075


 0.00
species
2
1
5044

genus

 0.00
0
45244
1
1
2

2614577
1
1
species

 0.00


 0.00
family
1
21
8
0
474942

1
1
2
42367
0

 0.00
genus

species

 0.00
111463
1
1

genus

 0.00
1052105
0
3
18
1

1
1
33203

 0.00
species


 0.00
species
1
17
2060973

1
2
2
0
474995

 0.00
genus

1
2
1119999

 0.00
species

family

 0.00
4
474943
8
1
15

5581
0
1
6
3
genus

 0.00


 0.00
species
1
6
2
176275
5

1
1
655819

 0.00
strain


 0.00
genus
4
1
5
1
45234

1
1
73501

 0.00
species

114497
0
3
1
2
species

 0.00

strain

 0.00
1081104
1
3

1
110618
35
1
25
family

 0.00


 0.00
genus
2
1
1
0
1079112

1079257
1
1
species

 0.00

3
1
1
57138
0

 0.00
genus

0
2779503
2
1
1
no rank

 0.00

1
1
182845

 0.00
species

genus

 0.00
5506
5
17
28
1

0
232080
3
1
2
species group

 0.00


 0.00
species
1
1
2747967


 0.00
species
1
1
169388

species group

 0.00
0
171631
3
1
1

species

 0.00
0
5507
2
1
1

1
1
660027

 0.00
strain

species group

 0.00
1
569360
3
1
4


 0.00
species
2
1
101028

1
1
5516

 0.00
species

species group

 0.00
0
171627
1
14
4

192010
1
3
species

 0.00

1567544
1
5
species

 0.00


 0.00
species
6
1
5127


 0.00
species group
3
2
1
450425
0

species

 0.00
231269
1
1

species

 0.00
2675880
1
1

0
140106
4
1
2
genus

 0.00


 0.00
species
4
1
78403

2
34397
16
83
1
family

 0.00

9
1
2
124426
0

 0.00
genus


 0.00
species
1
9
1159556

genus

 0.00
2
5112
51
1
11

species

 0.00
2
5113
3
1
12


 0.00
subspecies
1
1
1616224

1
9
2570311

 0.00
subspecies

47801
1
1
species

 0.00


 0.00
species
8
1
55200


 0.00
species
1
18
447254

3
1
2
2
35717

 0.00
species

1
1
877507

 0.00
strain

species

 0.00
79589
2
1

species

 0.00
42805
1
5


 0.00
genus
2
21
1
0
5529

1
21
500148

 0.00
species


 0.00
order
19
1
17
1028384
0

3
1
4
0
1033978

 0.00
family


 0.00
genus
3
3
1
0
1401161

species

 0.00
1302862
0
2
3
1


 0.00
strain
3
1
1314773

12
16
1
0
681950

 0.00
family

0
5455
1
16
11
genus

 0.00

2707335
0
2
4
1
no rank

 0.00

species

 0.00
145971
4
1

1209926
1
1
species

 0.00


 0.00
no rank
2
1
1
2872341
0

1
1
1543685

 0.00
species

2
1
1
2707339
0

 0.00
no rank


 0.00
species
1
1
5467


 0.00
no rank
1
9
3
2707338
4

species

 0.00
690256
1
4


 0.00
species
1
1
474922

1
4
10
147547
0

 0.00
class


 0.00
clade
9
1
4
1520881
0


 0.00
subclass
1
4
8
0
388435

7
1
4
5197
0

 0.00
order

157822
0
6
4
1
suborder

 0.00

0
78060
1
1
2
family

 0.00

genus

 0.00
112415
1
1

0
56478
3
1
3
family

 0.00

0
93111
2
1
3
genus

 0.00

species

 0.00
2732470
1
3


 0.01
class
126
209
1
147545
1

subclass

 0.00
451871
0
100
183
1

167
1
77
3
5042

 0.00
order

61
1
147
1131492
0

 0.00
family


 0.00
genus
49
1
125
23
5052

2
1
75553

 0.00
species

species

 0.00
446911
0
2
1
1

1
1
1448315

 0.00
strain

34381
0
3
1
2
species

 0.00

1448312
1
3
strain

 0.00


 0.00
species
1
5
1287682


 0.00
species
1
1
1220207


 0.00
species
1
1
41058

0
319626
1
2
2
species

 0.00

1
2
1450537

 0.00
strain

0
979771
6
1
2
species

 0.00

strain

 0.00
1450539
1
6

1069201
2
1
species

 0.00

2
1
209559

 0.00
species

subgenus

 0.00
2720872
1
5
8
1

0
5057
2
1
5
species

 0.00

344612
5
1
strain

 0.00

36630
0
1
2
2
species

 0.00


 0.00
strain
1
2
331117

2
1
1
319627
0

 0.00
species

1
1
1450535

 0.00
strain

0
340412
1
1
2
species

 0.00

1
1
1392255

 0.00
strain


 0.00
species
2
1
1
301854
0

strain

 0.00
1448316
1
1

11
1
2
0
51019

 0.00
species

11
1
1448321

 0.00
strain

319631
0
15
1
2
species

 0.00

1448317
1
15
strain

 0.00

species

 0.00
41067
2
1

1
1
41047

 0.00
species

subgenus

 0.00
3
2720871
16
1
5

species

 0.00
5061
2
1

0
306088
1
9
2
species

 0.00

9
1
1392250

 0.00
strain

species

 0.00
5059
2
1


 0.00
species
4
1
61420


 0.00
species
2
1
1220188


 0.00
subgenus
4
1
5
2720870
0

2
3
1
0
162425

 0.00
species

3
1
227321

 0.00
strain

species

 0.00
75750
0
2
1
1

strain

 0.00
1036612
1
1

2
1
1
1191702
0

 0.00
species

strain

 0.00
1448319
1
1

2
1
4
0
1196635

 0.00
species

1448310
4
1
strain

 0.00

6
1
138277

 0.00
species

0
5073
8
1
17
genus

 0.00

species

 0.00
69781
1
1

species

 0.00
36651
1
2

3
9
1
0
254878

 0.00
no rank

2
9
1
1108849
0

 0.00
species

strain

 0.00
500485
1
9

species

 0.00
60172
1
1


 0.00
species
1
4
27334

3
1
5
70110
0

 0.00
genus

species

 0.00
41063
0
2
5
1

1073090
5
1
strain

 0.00

family

 0.00
28568
0
12
1
16


 0.00
genus
11
1
16
5094
0

section

 0.00
0
2752543
2
1
2


 0.00
species
2
1
1131652


 0.00
section
4
1
2
2752542
0


 0.00
species
4
1
121627

2752537
4
1
8
4
section

 0.00


 0.00
species
1
2
28572


 0.00
species
1
1
128442

species

 0.00
198730
1
1

section

 0.00
2752540
0
2
2
1


 0.00
species
2
1
1441469

family

 0.00
0
1131624
1
1
3

genus

 0.00
0
33202
2
1
1

1
1
264951

 0.00
species

22
1
16
0
33183

 0.00
order

34384
0
7
1
2
family

 0.00

5550
0
3
1
1
genus

 0.00


 0.00
species
2
1
1
63400
0

strain

 0.00
663331
1
1


 0.00
genus
1
1
3
0
1915381

63402
0
2
1
1
species

 0.00

535722
1
1
strain

 0.00

8
1
12
0
299071

 0.00
family

5
1
4
5036
0

 0.00
genus

3
5
1
5037
1

 0.00
species

strain

 0.00
447093
2
1


 0.00
strain
2
1
544711

3
1
7
4
229219

 0.00
genus

0
5039
1
3
2
species

 0.00

strain

 0.00
559297
1
3

0
1593277
6
1
2
no rank

 0.00

genus

 0.00
38946
0
5
2
1

121759
0
2
1
1
species

 0.00

502780
1
1
strain

 0.00


 0.00
species
1
1
2
1048829
0

1
1
502779

 0.00
strain

25
1
25
451870
0

 0.00
subclass


 0.00
order
24
1
19
0
34395

18
24
1
43219
1

 0.00
family

10
1
3
1
5583

 0.00
genus


 0.00
species
2
1
215243

212818
7
1
species

 0.00

genus

 0.00
0
5587
3
1
1

1
1
2
86056
0

 0.00
species

1
1
1442369

 0.00
strain

3
1
2
0
82105

 0.00
genus

86049
0
2
1
2
species

 0.00

strain

 0.00
1279043
1
2

0
43220
3
8
1
genus

 0.00


 0.00
species
2
1
8
0
43228

8
1
1182542

 0.00
strain


 0.00
genus
2
1
5
0
40354

species

 0.00
979981
0
1
1
2

1442371
1
1
strain

 0.00


 0.00
species
2
1
1
0
40355


 0.00
strain
1
1
1442368

1
1
5
146291
0

 0.00
order

146292
0
4
1
1
family

 0.00

0
364710
3
1
1
genus

 0.00


 0.00
species
1
1
2
0
364733


 0.00
strain
1
1
1263415

6
1
1
0
189478

 0.00
class


 0.00
order
1
1
5
189479
0

4
1
1
0
47021

 0.00
family

1
1
3
47022
0

 0.00
genus

2813651
0
2
1
1
species

 0.00

strain

 0.00
756982
1
1

7
1
10
0
147549

 0.00
class

5185
0
10
1
6
order

 0.00


 0.00
family
5
10
1
0
5192


 0.00
genus
4
10
1
5193
0

section

 0.00
0
1051054
3
1
10


 0.00
species
1
1
1174673

9
1
1174677

 0.00
species


 0.00
subphylum
89
1
117
0
147537

class

 0.00
0
4891
88
1
117

4892
0
87
1
117
order

 0.00


 0.00
family
3
1
1
0
34353

genus

 0.00
1232588
0
2
1
1

species

 0.00
2606893
1
1

1156497
0
4
1
4
family

 0.00


 0.00
genus
1
4
3
0
4919

2
1
4
4926
0

 0.00
species

strain

 0.00
763406
4
1

12
1
8
0
2926619

 0.00
clade


 0.00
family
12
1
7
0
34366

4943
3
12
1
6
genus

 0.00

4944
1
2
species

 0.00

species

 0.00
44092
1
3

1725355
2
1
species

 0.00

2636529
0
1
2
2
no rank

 0.00

2826930
1
2
species

 0.00

no rank

 0.00
241407
0
3
1
6

0
2952368
6
1
2
genus

 0.00


 0.00
species
6
1
312227

0
4893
1
41
22
family

 0.00

20
1
3
0
4910

 0.00
genus


 0.00
species
15
1
4911


 0.00
species
1
5
28985

genus

 0.00
33170
0
1
3
5


 0.00
species
1
1
33169

1
1
45286

 0.00
species

species

 0.00
0
45285
2
1
1


 0.00
strain
1
1
931890

1
1
3
4948
0

 0.00
genus

no rank

 0.00
2629994
0
1
1
2

2792677
1
1
species

 0.00

3
1
2
71245
0

 0.00
genus

species

 0.00
61262
3
1


 0.00
genus
2
9
1
4930
3


 0.00
species
6
1
4932

genus

 0.00
0
113604
3
1
3

species

 0.00
1071379
0
2
1
3


 0.00
strain
3
1
1071380

2
1
3
278028
0

 0.00
genus


 0.00
species
1
2
2
27289
0

strain

 0.00
1071378
2
1

9
9
1
115784
0

 0.00
family

genus

 0.00
0
460517
3
1
6


 0.00
species
1
3
460519

species

 0.00
4922
3
1


 0.00
genus
3
2
1
599737
0

4927
0
2
2
1
species

 0.00

1
2
683960

 0.00
strain

604195
0
1
1
2
genus

 0.00

species

 0.00
36022
1
1

family

 0.00
0
34365
1
1
3

36034
0
1
1
2
genus

 0.00

36035
1
1
species

 0.00


 0.00
clade
1
39
27
0
2916678


 0.00
family
15
29
1
766764
2


 0.00
genus
2
2
1
507510
0

717740
2
1
species

 0.00

8
22
1
0
1535325

 0.00
clade

5475
0
1
22
7
genus

 0.00

273371
0
4
1
2
species

 0.00

1136231
1
4
strain

 0.00

species

 0.00
5480
4
1

species

 0.00
0
42374
2
1
4

strain

 0.00
573826
1
4

species

 0.00
5476
10
1

genus

 0.00
0
4958
4
1
3

species

 0.00
58627
1
2

1
1
2
4959
0

 0.00
species

1
1
284592

 0.00
strain

family

 0.00
27319
0
10
1
11

2937349
0
1
9
6
no rank

 0.00


 0.00
genus
5
1
9
2964429
1

4
1
418784

 0.00
species

1
1
45357

 0.00
species


 0.00
species
1
1
45354


 0.00
species
1
2
498019

genus

 0.00
0
27320
4
1
1

3
1
1
27322
0

 0.00
species

2
1
1
280587
0

 0.00
varietas

strain

 0.00
869754
1
1

7
4
1
410830
0

 0.00
family


 0.00
genus
2
1
1
410829
0

1
1
796027

 0.00
species

genus

 0.00
43971
0
1
2
2

species

 0.00
44072
2
1

genus

 0.00
45787
0
1
1
2


 0.00
species
1
1
45607

phylum

 0.01
5204
1
186
1
287

29000
0
1
7
7
subphylum

 0.00

6
1
7
0
162484

 0.00
class


 0.00
order
1
7
5
5258
0

family

 0.00
0
5262
4
1
7

3
7
1
5296
0

 0.00
genus


 0.00
species
2
7
1
27350
0

forma specialis

 0.00
168172
7
1

subphylum

 0.00
0
452284
162
1
36

14
5
1
452283
0

 0.00
class

order

 0.00
0
62913
1
1
5

62919
0
4
1
1
family

 0.00

genus

 0.00
5280
0
1
1
3


 0.00
species
2
1
1
0
5281


 0.00
strain
1
1
1037660

4
1
1
0
5404

 0.00
order

0
5405
1
1
3
family

 0.00


 0.00
genus
2
1
1
0
5406

271129
1
1
species

 0.00

162475
0
4
1
3
order

 0.00

3
1
3
162477
0

 0.00
no rank

561108
0
2
3
1
genus

 0.00

1569628
3
1
species

 0.00

1
143
8
1538075
0

 0.00
class

7
1
143
162474
0

 0.00
order

742845
0
143
1
6
family

 0.00

1
143
5
55193
0

 0.00
genus

species

 0.00
76773
19
1

76777
4
1
species

 0.00

76775
96
120
1
2
species

 0.00

425264
24
1
strain

 0.00

13
14
1
5257
0

 0.00
class

0
5267
12
1
14
order

 0.00

family

 0.00
0
5268
1
14
11


 0.00
genus
1
2
2
0
1392992

1
2
249478

 0.00
species

3
1
1
63298
0

 0.00
genus

species

 0.00
0
84751
1
1
2

1
1
1277687

 0.00
strain

5269
0
3
1
2
genus

 0.00

species

 0.00
307758
1
3

3
1
8
63265
0

 0.00
genus


 0.00
species
2
1
49012


 0.00
species
6
1
280036

subphylum

 0.00
0
5302
142
117
1


 0.00
class
102
83
1
155619
0

1
22
43
355688
0

 0.00
no rank


 0.00
order
20
1
14
0
5303


 0.00
family
1
4
5
0
1769247


 0.00
genus
2
1
2
2066992
0

2
1
34475

 0.00
species

genus

 0.00
0
2983002
2
1
2

species

 0.00
139415
2
1

0
2983427
3
1
1
family

 0.00

genus

 0.00
0
599838
2
1
1


 0.00
species
1
1
599839

0
2028212
3
1
1
family

 0.00

genus

 0.00
5629
0
1
1
2

species

 0.00
5630
1
1

8
1
8
0
5317

 0.00
family


 0.00
genus
1
1
3
0
114154

114155
0
2
1
1
species

 0.00


 0.00
strain
1
1
732165

5324
0
7
1
4
genus

 0.00


 0.00
species
4
1
5327

species

 0.00
0
5325
2
3
1


 0.00
strain
1
3
717944


 0.00
order
2
1
5
452338
0

family

 0.00
908827
0
2
1
4

3
2
1
133746
0

 0.00
genus

1
2
2
0
202698

 0.00
species

741275
1
2
strain

 0.00

7
3
1
0
36064

 0.00
order


 0.00
family
6
3
1
5250
0


 0.00
genus
1
1
3
0
5251


 0.00
no rank
2
1
1
2600200
0

species

 0.00
170446
1
1

2
2
1
1322061
0

 0.00
genus

species

 0.00
456999
1
2

0
452342
3
1
10
order

 0.00

family

 0.00
0
103376
4
1
1

genus

 0.00
5644
0
1
1
3

2
1
1
0
40492

 0.00
species

1
1
721885

 0.00
strain

5
2
1
0
40420

 0.00
family


 0.00
genus
4
2
1
13562
0

3
2
1
256003
0

 0.00
no rank


 0.00
species
2
1
2
984962
0

strain

 0.00
747525
1
2

subclass

 0.00
452333
0
58
61
1

7
1
6
68889
0

 0.00
order

1
6
6
0
227332

 0.00
suborder

227336
0
5
6
1
family

 0.00

genus

 0.00
5379
1
4
6
1

48587
1
1
species

 0.00

species

 0.00
48578
1
2


 0.00
species
2
1
1904413


 0.00
order
1
55
50
0
5338


 0.00
suborder
11
1
9
0
2982305


 0.00
family
3
3
1
0
40562

71950
0
2
3
1
genus

 0.00

species

 0.00
181762
1
3


 0.00
family
1
1
4
184208
0

1
1
3
184431
0

 0.00
genus

1
1
2
0
5346

 0.00
species

1
1
240176

 0.00
strain


 0.00
family
3
1
5
0
5339

genus

 0.00
0
5340
2
1
5


 0.00
species
5
1
5341

0
2982303
13
14
1
suborder

 0.00

0
71934
2
1
3
family

 0.00


 0.00
genus
2
1
2
0
71935

species

 0.00
648681
2
1

family

 0.00
930979
0
6
5
1

4
1
3
71927
0

 0.00
genus

1
4
2
2634893
0

 0.00
no rank


 0.00
species
4
1
1916073

2
1
1
47720
0

 0.00
genus

1
1
64660

 0.00
species

family

 0.00
5351
0
3
7
1

genus

 0.00
0
40144
2
1
7

40145
7
1
species

 0.00

0
2982316
16
29
1
suborder

 0.00


 0.00
family
2
1
3
72117
0


 0.00
genus
2
1
2
5352
0

5353
1
2
species

 0.00

family

 0.00
0
2024004
4
4
1

genus

 0.00
0
41247
1
4
3

species

 0.00
2126181
3
1

1033013
1
1
species

 0.00

5
12
1
862241
0

 0.00
family

genus

 0.00
0
38944
2
1
1

species

 0.00
38945
1
1

2
11
1
47424
0

 0.00
genus

11
1
47428

 0.00
species

3
1
11
654128
0

 0.00
family

2
1
11
34448
0

 0.00
genus

181124
11
1
species

 0.00

suborder

 0.00
2985477
0
1
2
5

4
2
1
5332
0

 0.00
family

0
5333
1
2
3
genus

 0.00

1
2
2
5334
0

 0.00
species

strain

 0.00
578458
2
1

4
1
1
2983527
0

 0.00
suborder

1
1
3
104366
0

 0.00
family

genus

 0.00
0
5320
1
1
2


 0.00
species
1
1
5322

1
34
39
155616
0

 0.00
class


 0.00
order
23
1
18
5234
0

0
1910893
1
1
3
family

 0.00

genus

 0.00
0
4998
1
1
2

species

 0.00
4999
1
1

0
1884633
15
1
15
family

 0.00


 0.00
genus
9
1
8
0
5206


 0.00
species group
7
1
2
0
1897064

species

 0.00
5207
7
1

0
1884637
3
1
1
species group

 0.00


 0.00
species
2
1
1
0
37769

1
1
367775

 0.00
strain


 0.00
species
1
1
2
104669
0

strain

 0.00
1295533
1
1

genus

 0.00
490731
0
1
6
6

species

 0.00
324769
0
2
1
2

strain

 0.00
1296100
2
1

species

 0.00
0
453459
1
1
2

1296096
1
1
strain

 0.00


 0.00
species
3
1
1734106


 0.00
family
1
2
4
0
5215

105767
0
1
2
3
genus

 0.00

species

 0.00
5217
0
1
2
2

1
2
578456

 0.00
strain


 0.00
order
7
1
7
1851469
0

0
1759442
6
7
1
family

 0.00

genus

 0.00
0
105983
3
1
6


 0.00
species
1
5
252803


 0.00
species
1
1
105984

2
1
1
0
1838142

 0.00
genus

879819
1
1
species

 0.00

0
90886
5
1
4
order

 0.00

family

 0.00
0
5408
5
1
3

2
5
1
5209
0

 0.00
genus


 0.00
species
5
1
5210

order

 0.00
90883
0
4
1
4

family

 0.00
1851551
0
4
1
3

genus

 0.00
0
107449
2
4
1

species

 0.00
264483
1
4

1
1
4
0
2687318

 0.00
class

3
1
1
192874
0

 0.00
genus

1
1
2
192875
0

 0.00
species

1
1
595528

 0.00
strain

class

 0.00
0
127916
1
1
5


 0.00
order
4
1
1
198625
0


 0.00
genus
1
1
3
72018
0

2
1
1
0
72019

 0.00
species

667725
1
1
strain

 0.00

clade

 0.00
2611341
0
7
1
2

phylum

 0.00
5719
0
6
1
2

5
2
1
37104
0

 0.00
order

family

 0.00
181550
0
1
2
4

genus

 0.00
0
5721
1
2
3

5722
0
2
2
1
species

 0.00


 0.00
isolate
1
2
412133


 0.00
clade
1
50
50
2611352
0


 0.00
phylum
8
7
1
5752
0

0
2601529
7
7
1
clade

 0.00

6
7
1
0
2601530

 0.00
clade


 0.00
family
5
1
7
5765
0


 0.00
genus
4
1
7
0
5761

5763
2
1
species

 0.00

51637
1
1
species

 0.00

1
4
5762

 0.00
species

phylum

 0.00
0
33682
1
43
41

4
5
1
0
191814

 0.00
order

3
5
1
0
2603949

 0.00
family

genus

 0.00
0
160598
2
1
5


 0.00
species
5
1
2016123

0
5653
1
38
36
class

 0.00

0
2704647
35
38
1
subclass

 0.00


 0.00
order
38
1
34
2704949
0

38
1
33
5654
0

 0.00
family

5690
0
13
1
8
genus

 0.00

0
47570
3
1
2
subgenus

 0.00

species

 0.00
5693
0
1
2
2


 0.00
subspecies
1
2
85057


 0.00
subgenus
1
1
39700

669453
0
2
2
1
clade

 0.00

species

 0.00
83891
2
1

47569
0
1
1
3
subgenus

 0.00

species

 0.00
0
5692
2
1
1

1068625
1
1
strain

 0.00

39701
0
3
1
2
subgenus

 0.00

1
1
5698

 0.00
species

species

 0.00
5695
1
1

30
1
19
0
1286322

 0.00
subfamily

1
1
2
5683
0

 0.00
genus

species

 0.00
157538
1
1

genus

 0.00
5658
0
16
1
29


 0.00
subgenus
5
1
6
2
37616


 0.00
species group
1
1
38579

37617
0
4
2
1
species group

 0.00

species

 0.00
0
5660
1
1
2

420245
1
1
strain

 0.00

5681
1
1
species

 0.00

9
1
24
38568
0

 0.00
subgenus

1
38574
2
3
1
species group

 0.00


 0.00
species
2
1
5661

0
38581
2
1
2
species group

 0.00

5664
1
2
species

 0.00

38582
3
4
1
19
species group

 0.00

2
1
14
0
5665

 0.00
species

929439
14
1
strain

 0.00

5659
2
1
species

 0.00


 0.00
clade
149
176
1
2698737
1

1
121
87
33630
0

 0.00
clade

8
1
17
5878
0

 0.00
phylum

431838
0
16
8
1
subphylum

 0.00


 0.00
class
1
8
15
0
6020

33825
0
5
3
1
order

 0.00


 0.00
family
4
1
3
0
340080

5884
0
3
1
3
genus

 0.00

species

 0.00
5888
0
2
3
1

412030
1
3
strain

 0.00

9
5
1
31277
0

 0.00
order


 0.00
suborder
1
3
5
0
37093

291294
0
1
3
4
family

 0.00


 0.00
genus
3
3
1
0
5890

2
1
3
0
5911

 0.00
species

strain

 0.00
312017
3
1

3
1
2
0
37090

 0.00
suborder


 0.00
genus
2
2
1
5931
0

species

 0.00
5932
1
2

phylum

 0.00
5794
0
69
1
113

class

 0.00
1280412
0
54
1
23

1
54
22
5796
0

 0.00
subclass

order

 0.00
75739
0
21
54
1

423054
0
20
54
1
suborder

 0.00

35082
0
4
1
5
family

 0.00

0
5806
3
1
5
genus

 0.00


 0.00
no rank
1
1
203864

1
4
857276

 0.00
species

5809
0
8
1
33
family

 0.00


 0.00
genus
2
1
1
94642
0

94643
1
1
species

 0.00

3
10
1
29175
0

 0.00
genus

0
29176
2
1
10
species

 0.00

strain

 0.00
572307
10
1

genus

 0.00
5810
0
2
1
22

5811
22
1
species

 0.00

family

 0.00
0
5799
1
16
7

1
3
2
44417
0

 0.00
genus

88456
1
3
species

 0.00


 0.00
genus
1
13
4
5800
0

5804
1
3
species

 0.00

5802
8
1
species

 0.00


 0.00
species
1
2
44415

class

 0.00
422676
0
45
1
59

2735539
0
1
1
4
order

 0.00


 0.00
genus
3
1
1
0
873537

2637010
0
2
1
1
no rank

 0.00


 0.00
species
1
1
2544991


 0.00
order
30
1
52
5819
1

family

 0.00
0
1639119
25
50
1

0
5820
24
1
50
genus

 0.00


 0.00
subgenus
15
1
8
3
418107

880536
1
4
species

 0.00

species

 0.00
5854
1
1


 0.00
species
3
1
2
1
5833

36329
2
1
isolate

 0.00


 0.00
species
1
1
647221


 0.00
species
1
2
720590


 0.00
species
1
1
880535

418101
4
6
11
1
subgenus

 0.00


 0.00
species
2
1
5861

5821
3
1
species

 0.00

5860
0
3
1
2
species

 0.00

1
1
54757

 0.00
subspecies

1
1
119398

 0.00
subspecies

subgenus

 0.00
418104
0
4
1
2

species

 0.00
85471
1
4

208452
1
3
species

 0.00

418103
0
6
17
1
subgenus

 0.00

1
3
77519

 0.00
species

species

 0.00
5858
4
1

2
1
5827

 0.00
species

species

 0.00
5850
2
1


 0.00
species
1
6
36330

0
1639121
1
1
4
family

 0.00


 0.00
genus
1
1
3
0
77521

no rank

 0.00
219820
0
2
1
1

191679
1
1
species

 0.00

order

 0.00
0
5863
1
6
10

32594
0
5
1
3
family

 0.00


 0.00
genus
4
1
3
5864
0

189622
1
1
species

 0.00

0
5868
2
1
2
species

 0.00


 0.00
strain
2
1
1133968

4
1
3
27994
0

 0.00
family

genus

 0.00
0
5873
1
3
3

5872
1
1
species

 0.00


 0.00
species
1
2
68886

clade

 0.00
1
33634
61
1
54

12
9
1
0
4762

 0.00
phylum


 0.00
order
7
1
4
4776
0

7
1
3
0
4777

 0.00
family

4783
0
7
1
2
genus

 0.00

species

 0.00
67593
1
7

7
1
2
4763
0

 0.00
order


 0.00
family
6
2
1
0
4764

4769
0
3
1
1
genus

 0.00

0
112098
1
1
2
species

 0.00


 0.00
strain
1
1
1156394


 0.00
genus
1
1
2
100860
0

112090
1
1
species

 0.00

class

 0.00
0
2683628
6
1
6

6
1
5
2683629
0

 0.00
clade

0
42740
1
6
4
order

 0.00

family

 0.00
2547934
0
6
1
3

0
12967
2
6
1
genus

 0.00

12968
1
6
species

 0.00

2696291
0
42
38
1
clade

 0.00

clade

 0.00
0
569578
1
1
5


 0.00
class
1
1
4
2870
0

3
1
1
0
2886

 0.00
order

family

 0.00
33636
0
1
1
2


 0.00
genus
1
1
35121

2836
0
6
1
21
phylum

 0.00

1
1
7
33836
0

 0.00
class

subclass

 0.00
33846
0
6
1
1

order

 0.00
33847
0
1
1
5

4
1
1
0
29202

 0.00
family

3
1
1
0
35127

 0.00
genus


 0.00
species
2
1
1
0
35128

strain

 0.00
296543
1
1

13
1
5
0
33849

 0.00
class

33850
1
1
5
12
clade

 0.00

0
245176
4
1
1
order

 0.00

family

 0.00
245177
0
1
1
3

1
1
2
420972
0

 0.00
genus

2809013
1
1
species

 0.00


 0.00
order
7
1
3
0
38748

0
38749
3
1
2
family

 0.00


 0.00
genus
2
2
1
0
2849

species

 0.00
2850
2
1

67474
0
3
1
1
family

 0.00

880756
0
1
1
2
genus

 0.00


 0.00
species
1
1
1519565

5747
0
5
1
24
class

 0.00

1
24
4
425074
0

 0.00
order

family

 0.00
425072
0
3
24
1


 0.00
genus
1
24
2
0
5748


 0.00
species
1
24
145522

7
6
1
35675
0

 0.00
class

order

 0.00
54409
0
6
1
6

0
44055
2
1
3
genus

 0.00

1
3
44056

 0.00
species

3
3
1
35676
0

 0.00
genus

1
3
2
660916
0

 0.00
no rank

660917
1
3
species

 0.00

3
1
1
33859
0

 0.00
class

order

 0.00
0
420622
1
1
2

1
1
420617

 0.00
family

23
33090
3979
1
802
kingdom

 0.10

1
3919
730
35493
0

 0.10
phylum


 0.10
subphylum
729
1
3919
0
131221

3919
1
728
32
3193

 0.10
clade

43
1
8
0
3195

 0.00
clade

186770
0
43
1
7
class

 0.00

6
1
43
186774
0

 0.00
subclass

order

 0.00
28908
0
43
1
5


 0.00
family
43
1
4
0
29585

1
43
3
3196
0

 0.00
genus

species

 0.00
0
3197
2
43
1


 0.00
subspecies
43
1
1480154

17
47
1
0
3208

 0.00
clade

47
1
16
0
404260

 0.00
clade

class

 0.00
0
3214
47
1
15

subclass

 0.00
0
114658
1
39
9

39
1
8
0
404315

 0.00
superorder


 0.00
order
7
39
1
13798
5

1
16
3
0
61526

 0.00
family

genus

 0.00
0
67427
1
16
2

1
16
67428

 0.00
species

0
28466
3
18
1
family

 0.00

0
28467
2
1
18
genus

 0.00

species

 0.00
28468
18
1

subclass

 0.00
114656
0
1
8
5


 0.00
order
1
8
4
3215
0


 0.00
family
3
8
1
0
3216

genus

 0.00
37414
0
8
1
2

8
1
3218

 0.00
species

702
1
3797
2
58023

 0.09
clade


 0.00
class
5
1
7
0
1521260

order

 0.00
0
3244
1
7
4

3245
0
1
7
3
family

 0.00


 0.00
genus
2
1
7
0
3246


 0.00
species
7
1
88036


 0.09
clade
696
1
3788
2
78536


 0.00
class
6
1
19
0
241806

1521262
0
18
6
1
subclass

 0.00

693762
0
1
2
4
order

 0.00

0
693766
1
2
3
family

 0.00

2
1
2
12939
0

 0.00
genus

species

 0.00
12940
1
2

order

 0.00
0
3268
4
1
13

5
2
1
0
2003540

 0.00
suborder


 0.00
family
1
2
4
0
13819

2003543
0
3
2
1
subfamily

 0.00

genus

 0.00
0
29595
2
1
2

species

 0.00
49495
1
2

4
1
1
1203512
0

 0.00
suborder

family

 0.00
29629
0
1
1
3

genus

 0.00
32155
0
1
1
2


 0.00
species
1
1
449867

0
1203511
1
1
3
suborder

 0.00

family

 0.00
41972
0
1
1
2

1
1
32071

 0.00
genus


 0.09
clade
676
3780
1
58024
1

class

 0.09
6
3398
3759
1
657

2
1
4
261009
0

 0.00
order

22097
0
3
1
2
family

 0.00

genus

 0.00
0
13332
2
1
2


 0.00
species
1
2
13333

clade

 0.09
1437183
157
648
3747
1


 0.01
clade
435
1
151
0
4447


 0.00
order
9
1
22
16360
0

4454
0
5
21
1
family

 0.00

0
284551
4
1
21
subfamily

 0.00

genus

 0.00
4473
0
1
21
3

29656
1
11
species

 0.00


 0.00
species
10
1
51605

family

 0.00
0
27254
1
1
3


 0.00
genus
2
1
1
0
27257

species

 0.00
29655
1
1

0
1437197
141
413
1
subclass

 0.01

3
1
1
0
4667

 0.00
order


 0.00
family
1
1
2
0
50362

49669
1
1
genus

 0.00


 0.00
order
7
1
5
40548
0

family

 0.00
4671
0
4
7
1

1
7
3
0
4672

 0.00
genus

7
1
2
29710
0

 0.00
species

55577
1
7
subspecies

 0.00


 0.01
clade
114
1
399
4734
0

order

 0.00
4618
0
9
1
43

42
1
5
4637
0

 0.00
family

0
4640
42
1
4
genus

 0.00

21
1
2
2
4641

 0.00
species

214687
1
19
subspecies

 0.00

320322
21
1
species

 0.00

4642
0
1
1
3
family

 0.00


 0.00
genus
1
1
2
4650
0

1
1
94328

 0.00
species

0
38820
93
1
350
order

 0.01

5
1
48
0
14101

 0.00
family

2
27
1
0
46322

 0.00
genus


 0.00
species
27
1
59018


 0.00
genus
1
21
2
13578
0

species

 0.00
13579
1
21

4613
0
32
1
5
family

 0.00

1909378
0
4
32
1
subfamily

 0.00


 0.00
genus
3
1
32
4614
0


 0.00
species
1
32
2
22
4615

varietas

 0.00
296719
10
1

7
1
1
0
4609

 0.00
family

0
986140
6
1
1
subfamily

 0.00

tribe

 0.00
986152
0
1
1
5


 0.00
genus
1
1
4
0
76510

subgenus

 0.00
0
1916803
3
1
1


 0.00
section
2
1
1
1916808
0


 0.00
species
1
1
1735547

269
1
75
4
4479

 0.01
family

0
147370
1
103
27
clade

 0.00

147369
1
26
103
1
subfamily

 0.00


 0.00
no rank
1
5
8
0
1648033

147429
0
7
5
1
tribe

 0.00

subtribe

 0.00
1648029
0
3
3
1

2
1
3
0
4575

 0.00
genus

species

 0.00
4577
1
3

0
1648028
2
1
3
subtribe

 0.00


 0.00
genus
2
1
2
4557
0


 0.00
species
2
1
4558


 0.00
no rank
97
1
17
1648036
0

16
1
97
0
147428

 0.00
tribe

1293361
0
5
26
1
subtribe

 0.00

2
1
18
4
4554

 0.00
genus

4556
14
1
species

 0.00

0
4583
1
8
2
genus

 0.00

8
1
154765

 0.00
species

2
1
3
0
1293363

 0.00
subtribe

genus

 0.00
0
416176
2
1
2


 0.00
species
2
1
435756

subtribe

 0.00
1293365
0
1
15
4


 0.00
genus
1
15
3
0
4539

section

 0.00
0
2100771
15
1
2

38727
15
1
species

 0.00

3
1
54
0
1293360

 0.00
subtribe

0
66017
54
1
2
genus

 0.00

species

 0.00
1010633
1
54

47
1
162
359160
1

 0.00
clade

147367
0
11
69
1
subfamily

 0.00

69
1
10
147380
0

 0.00
tribe

9
1
69
1648021
0

 0.00
subtribe

genus

 0.00
1
4527
8
1
69

species

 0.00
4537
1
4

57
1
3
4530
38

 0.00
species


 0.00
no rank
12
1
39947


 0.00
no rank
1
7
39946

species

 0.00
4535
1
1

1
2
4532

 0.00
species

63629
4
1
species

 0.00

1
92
35
147368
6

 0.00
subfamily

no rank

 0.00
1648038
0
56
1
14


 0.00
tribe
13
56
1
4
147389

6
1
4
0
1648017

 0.00
subtribe

0
4512
3
6
1
genus

 0.00

species

 0.00
0
4513
1
6
2

6
1
112509

 0.00
subspecies


 0.00
subtribe
8
1
46
8
1648030

genus

 0.00
4480
0
3
1
1

species

 0.00
37682
0
1
1
2

200361
1
1
subspecies

 0.00

1
37
4
11
4564

 0.00
genus


 0.00
species
1
23
4565

1
1
4571

 0.00
species

4572
1
2
species

 0.00


 0.00
no rank
26
1
16
1648037
0


 0.00
tribe
1
26
15
147387
5

clade

 0.00
0
1652081
1
10
10

subtribe

 0.00
2948572
0
2
1
1

15452
1
1
genus

 0.00

clade

 0.00
0
2948571
1
9
7


 0.00
subtribe
6
9
1
0
640630

genus

 0.00
3
4520
1
8
3

2
1
89674

 0.00
species

4522
3
1
species

 0.00

genus

 0.00
0
4605
1
1
2

98750
1
1
species

 0.00

4
1
11
0
1652080

 0.00
clade

3
1
11
640623
0

 0.00
subtribe

11
1
2
4496
0

 0.00
genus


 0.00
species
11
1
4498

1
4
4
0
2822797

 0.00
no rank

147385
0
3
4
1
tribe

 0.00


 0.00
genus
2
1
4
15367
0

species

 0.00
15368
4
1

11
6
1
40551
0

 0.00
order

0
4710
6
1
10
family

 0.00

subfamily

 0.00
169697
0
3
1
5

3
1
4
0
169705

 0.00
tribe

169729
0
3
3
1
subtribe

 0.00

51952
0
2
3
1
genus

 0.00

1
3
51953

 0.00
species

subfamily

 0.00
0
169700
3
1
4

1
3
3
169748
0

 0.00
tribe

genus

 0.00
4719
0
3
1
2

42345
3
1
species

 0.00

order

 0.00
0
73496
18
6
1

7
3
1
0
4668

 0.00
family


 0.00
subfamily
1
1
2
703251
0

2807454
1
1
tribe

 0.00

4
2
1
0
40553

 0.00
subfamily

703248
0
3
1
2
tribe

 0.00

0
4678
2
1
2
genus

 0.00

species

 0.00
105339
1
2

0
4747
1
1
6
family

 0.00

0
158332
1
1
5
subfamily

 0.00

4
1
1
0
158393

 0.00
tribe

subtribe

 0.00
0
158406
1
1
3


 0.00
genus
2
1
1
0
37818

1
1
906689

 0.00
species

family

 0.00
0
40552
4
2
1

703533
0
3
2
1
subfamily

 0.00

genus

 0.00
4685
0
2
2
1

4686
1
2
species

 0.00

232378
0
10
1
9
order

 0.00

1
7
5
0
4328

 0.00
family

4329
0
2
3
1
genus

 0.00

species

 0.00
60698
3
1

54954
0
2
4
1
genus

 0.00

4
1
54955

 0.00
species

family

 0.00
0
4429
3
3
1

3
1
2
0
4430

 0.00
genus

3
1
4432

 0.00
species


 0.08
clade
467
1
3131
71240
0


 0.08
clade
3131
1
466
0
91827


 0.08
clade
465
1
3131
1437201
294

71274
27
749
1
155
clade

 0.02

clade

 0.00
91882
0
46
1
139

0
4209
1
56
28
order

 0.00

27
56
1
4
4210

 0.00
family

219103
0
7
10
1
subfamily

 0.00

1
10
6
0
102818

 0.00
tribe

5
1
10
742010
0

 0.00
subtribe

0
4264
4
10
1
genus

 0.00

4265
0
3
10
1
species

 0.00

2
10
1
0
309979

 0.00
subspecies

59895
10
1
varietas

 0.00

5
1
13
0
219120

 0.00
subfamily

13
1
4
219121
0

 0.00
tribe


 0.00
subtribe
3
13
1
0
745062

genus

 0.00
1
4235
13
1
2


 0.00
species
12
1
4236

subfamily

 0.00
102804
0
1
29
14

102809
0
1
2
5
tribe

 0.00

877976
0
4
1
2
clade

 0.00


 0.00
subtribe
3
2
1
2841728
0

41574
0
2
1
2
genus

 0.00

72917
1
2
species

 0.00


 0.00
tribe
4
1
22
0
102806

911294
0
3
22
1
subtribe

 0.00

22
1
2
0
56534

 0.00
genus

22
1
56535

 0.00
species

911341
0
4
1
5
clade

 0.00


 0.00
tribe
3
5
1
0
102814


 0.00
genus
2
5
1
4231
0

5
1
4232

 0.00
species

1
83
17
0
4036

 0.00
order

16
1
83
364270
0

 0.00
suborder

1
79
3
0
4050

 0.00
family

2
79
1
4051
0

 0.00
genus

species

 0.00
4052
1
79

family

 0.00
4037
0
1
4
12


 0.00
subfamily
1
2
4
241779
0

tribe

 0.00
0
507443
2
1
3

0
90470
2
1
2
genus

 0.00

90471
1
2
species

 0.00

subfamily

 0.00
241778
0
2
1
7


 0.00
tribe
6
2
1
0
241789

2
1
5
241799
0

 0.00
subtribe

genus

 0.00
4038
0
2
1
4

0
1873447
3
2
1
section

 0.00

2
1
2
0
4039

 0.00
species


 0.00
subspecies
2
1
79200


 0.00
order
12
1
40
0
41945

family

 0.00
4345
0
1
1
5

217037
0
4
1
1
subfamily

 0.00

1
1
3
217062
0

 0.00
tribe

0
13749
1
1
2
genus

 0.00

13750
1
1
species

 0.00

family

 0.00
27065
0
3
6
1

genus

 0.00
0
4441
6
1
2

4442
6
1
species

 0.00


 0.00
family
1
33
3
0
25692


 0.00
genus
33
1
2
35939
0


 0.00
species
1
33
253017


 0.01
clade
96
543
1
8
91888


 0.00
order
49
1
174
4143
1

41399
0
1
4
3
family

 0.00


 0.00
genus
4
1
2
1502711
0


 0.00
species
4
1
4155


 0.00
family
1
49
18
4136
1

1
15
5
0
216703

 0.00
subfamily

tribe

 0.00
983535
1
1

3
1
14
0
983543

 0.00
tribe

genus

 0.00
0
155228
14
1
2

1
14
194200

 0.00
species

subfamily

 0.00
216706
0
9
4
1

216718
0
8
1
4
tribe

 0.00

0
2836339
4
1
7
subtribe

 0.00

genus

 0.00
21880
0
6
4
1

3
3
1
2026555
0

 0.00
subgenus


 0.00
clade
3
1
2
2026556
0


 0.00
species
1
3
180675

no rank

 0.00
2291027
0
2
1
1

49212
1
1
species

 0.00

0
216702
3
29
1
subfamily

 0.00

genus

 0.00
4139
0
1
29
2


 0.00
species
1
29
53169


 0.00
family
1
14
3
4180
0

genus

 0.00
4181
0
2
1
14

1
14
4182

 0.00
species

1
4144
81
1
11
family

 0.00

tribe

 0.00
426105
0
3
43
1

genus

 0.00
0
4147
1
43
2

1
43
660624

 0.00
species


 0.00
tribe
1
37
7
426106
0

38871
0
2
1
36
genus

 0.00


 0.00
species
36
1
56036

0
4145
4
1
1
genus

 0.00

0
4146
3
1
1
species

 0.00

subspecies

 0.00
0
158383
2
1
1

1
1
158386

 0.00
varietas

4185
0
1
3
5
family

 0.00

subfamily

 0.00
0
216691
4
3
1

3
1
3
0
216695

 0.00
tribe

0
175693
3
1
2
genus

 0.00

species

 0.00
175694
1
3

0
91896
4
1
1
family

 0.00


 0.00
tribe
1
1
3
0
216770

87753
0
2
1
1
genus

 0.00

species

 0.00
1937030
1
1

156152
0
4
21
1
family

 0.00

tribe

 0.00
0
216780
1
21
3


 0.00
genus
2
1
21
0
102598

species

 0.00
102599
1
21

2
4069
29
313
1
order

 0.01


 0.00
family
6
1
52
4118
0


 0.00
tribe
5
1
52
267213
0

genus

 0.00
11
4119
4
52
1

35885
1
11
species

 0.00

species

 0.00
35883
7
1

species

 0.00
35884
23
1

4070
2
259
1
22
family

 0.01

424551
2
16
254
1
subfamily

 0.01

0
424564
3
1
1
tribe

 0.00

genus

 0.00
4071
0
1
1
2

1
1
4072

 0.00
species

0
424574
250
1
9
tribe

 0.01

4107
14
8
1
250
genus

 0.01

50273
21
1
species

 0.00


 0.00
species
50
1
4113

49274
18
4
102
1
subgenus

 0.00


 0.00
species
1
1
4084

1
40
28526

 0.00
species

species

 0.00
4081
43
1

1
63
45834

 0.00
species


 0.00
tribe
3
1
1
424566
0

genus

 0.00
24609
0
1
1
2

1
1
33113

 0.00
species

3
1
5
0
424554

 0.00
subfamily

424562
0
4
3
1
tribe

 0.00


 0.00
genus
1
3
3
1
4085

1
1
4096

 0.00
species

4100
1
1
species

 0.00


 0.00
order
48
1
17
0
4055

5
2
1
4056
0

 0.00
family


 0.00
subfamily
2
1
4
0
167484

0
167488
2
1
3
tribe

 0.00


 0.00
subtribe
2
1
2
1498477
0

4065
1
2
genus

 0.00

family

 0.00
0
24966
11
1
46

4
1
2
0
169619

 0.00
subfamily


 0.00
tribe
3
1
2
0
169629


 0.00
genus
2
1
2
0
43473


 0.00
species
1
2
43474

44
1
6
169618
0

 0.00
subfamily

clade

 0.00
1968429
0
5
1
44

clade

 0.00
1968428
0
1
44
4


 0.00
tribe
44
1
3
169640
0


 0.00
genus
44
1
2
13442
1

49369
1
43
species

 0.00

288
1971
1
117
71275

 0.05
clade


 0.03
clade
172
1097
1
52
91835

order

 0.01
3744
1
36
1
250


 0.01
family
240
1
27
1
3745

0
171638
1
71
15
subfamily

 0.00

no rank

 0.00
0
1176516
1
3
3


 0.00
genus
2
1
3
3764
0

species

 0.00
74649
3
1

721790
0
55
1
3
tribe

 0.00

genus

 0.00
3761
1
2
55
1


 0.00
species
54
1
57919

tribe

 0.00
721789
0
13
1
8

1
12
3
1184125
0

 0.00
no rank


 0.00
genus
1
12
2
23204
0

12
1
57926

 0.00
species


 0.00
subtribe
1
1
4
0
1184124

3746
0
3
1
1
genus

 0.00

species

 0.00
0
57918
1
1
2

101020
1
1
subspecies

 0.00

0
171637
1
168
11
subfamily

 0.00

tribe

 0.00
721805
0
1
13
4

genus

 0.00
3
3754
3
1
13

species

 0.00
42229
1
1

9
1
3755

 0.00
species


 0.00
tribe
6
1
155
721813
5


 0.00
genus
3
1
145
61
3749


 0.00
species
1
77
3750

3752
7
1
species

 0.00

3766
0
5
1
2
genus

 0.00

species

 0.00
225117
1
5

0
3608
5
1
3
family

 0.00

325284
0
4
1
3
tribe

 0.00

72171
0
3
1
3
genus

 0.00

1
3
2
0
326968

 0.00
species


 0.00
varietas
3
1
714518

6
1
3
3481
0

 0.00
family


 0.00
genus
6
1
2
3482
0

3483
6
1
species

 0.00


 0.01
order
1
206
18
3502
0

7
1
140
1
3503

 0.00
family

genus

 0.00
21024
1
2
48
1

species

 0.00
28930
1
47


 0.00
genus
91
1
4
0
3511

38942
86
1
species

 0.00


 0.00
species
1
1
58331

1
4
97700

 0.00
species

3514
0
1
55
5
family

 0.00

13450
0
2
1
29
genus

 0.00

13451
29
1
species

 0.00


 0.00
genus
2
1
26
0
12989

176864
1
26
species

 0.00

family

 0.00
5
16714
5
11
1

genus

 0.00
13402
0
1
1
2


 0.00
species
1
1
32201

0
16718
5
1
2
genus

 0.00

5
1
51240

 0.00
species

order

 0.00
71239
0
19
100
1

1
100
18
0
3650

 0.00
family


 0.00
tribe
6
22
1
1003878
0

22
1
5
3660
2

 0.00
genus


 0.00
species
6
1
3661

2
5
1
3663
0

 0.00
species

subspecies

 0.00
3664
1
5

species

 0.00
3662
1
9

1003871
0
3
6
1
tribe

 0.00

3671
0
2
6
1
genus

 0.00


 0.00
species
1
6
3673


 0.00
tribe
1
72
8
0
1003877

2
1
1
3653
0

 0.00
genus

1
1
3654

 0.00
species

3655
0
3
1
50
genus

 0.00

3659
1
7
species

 0.00

43
1
3656

 0.00
species

102210
0
1
21
2
genus

 0.00

1
21
102211

 0.00
species


 0.00
order
4
1
1
0
233875


 0.00
family
3
1
1
4305
0

genus

 0.00
123484
0
2
1
1

1
1
458696

 0.00
species


 0.01
order
64
1
390
0
72025

1
389
60
0
3803

 0.01
family


 0.00
subfamily
5
5
1
3804
0

1
5
4
3807
2

 0.00
clade

0
163487
3
3
1
tribe

 0.00


 0.00
genus
1
3
2
35715
0

species

 0.00
207710
3
1

50
383
1
0
3814

 0.01
subfamily

1
383
49
4
2231393

 0.01
clade


 0.00
clade
1
49
5
2231384
0

0
2231385
49
1
4
clade

 0.00

3
1
49
163729
0

 0.00
tribe

3869
0
2
1
49
genus

 0.00


 0.00
species
1
49
3871

clade

 0.01
4
2231382
1
257
37


 0.00
clade
115
1
18
2233838
1

2233857
0
34
1
4
clade

 0.00


 0.00
tribe
34
1
3
163747
0

0
3867
1
34
2
genus

 0.00

species

 0.00
34305
34
1

clade

 0.00
2233839
0
80
1
13


 0.00
tribe
50
1
6
0
163742

genus

 0.00
0
3898
3
1
2

species

 0.00
57577
3
1

1
47
3
2
3877

 0.00
genus

9
1
3880

 0.00
species


 0.00
species
1
36
70936


 0.00
tribe
3
3
1
0
163743

2
3
1
3887
0

 0.00
genus

3
1
3888

 0.00
species

tribe

 0.00
163722
0
1
27
3

genus

 0.00
3826
0
27
1
2

27
1
3827

 0.00
species

2233855
0
138
1
18
clade

 0.00


 0.00
tribe
14
135
1
163735
2

3846
0
1
21
4
genus

 0.00


 0.00
subgenus
3
21
1
1462606
15

3847
1
5
species

 0.00

species

 0.00
3848
1
1

6
3913
1
104
5
genus

 0.00

species

 0.00
87088
1
1

3917
1
43
species

 0.00

3914
0
2
54
1
species

 0.00


 0.00
varietas
1
54
157739


 0.00
genus
1
3
2
3820
0

3821
3
1
species

 0.00

genus

 0.00
0
3883
1
5
2

species

 0.00
3885
5
1


 0.00
tribe
3
3
1
163715
0


 0.00
genus
2
1
3
0
3815

species

 0.00
3816
3
1

73
1
6
0
2231387

 0.00
clade

tribe

 0.00
163725
0
1
73
5


 0.00
clade
1
73
4
3
2231390

70
1
3
12
3817

 0.00
genus


 0.00
species
4
1
130454

1
54
3818

 0.00
species

0
1978182
1
1
4
subfamily

 0.00

163093
0
3
1
1
tribe

 0.00


 0.00
genus
1
1
2
0
162874

species

 0.00
2723574
1
1

family

 0.00
0
4274
3
1
1

0
1562315
1
1
2
genus

 0.00

species

 0.00
1562316
1
1

1
3646
30
98
1
order

 0.00


 0.00
family
1
1
3
0
233880


 0.00
subfamily
1
1
2
1583283
0

235595
1
1
tribe

 0.00

family

 0.00
4004
0
3
1
21

genus

 0.00
0
4005
21
1
2

21
1
4006

 0.00
species

family

 0.00
3688
0
6
1
18

238069
0
5
18
1
tribe

 0.00

3689
0
4
18
1
genus

 0.00

43335
1
1
species

 0.00

species

 0.00
75702
1
1


 0.00
species
16
1
3694


 0.00
family
17
1
57
3977
0

10
1
23
235631
0

 0.00
subfamily

0
235882
3
11
1
tribe

 0.00


 0.00
genus
2
1
11
3980
0

species

 0.00
3981
1
11


 0.00
tribe
9
1
3
235887
0

2
1
9
3995
0

 0.00
genus

1
9
180498

 0.00
species

tribe

 0.00
235883
0
3
3
1


 0.00
genus
2
3
1
3982
0

1
3
3983

 0.00
species

235629
0
6
34
1
subfamily

 0.00


 0.00
tribe
1
34
5
0
235880

3987
0
4
1
2
genus

 0.00

3988
1
4
species

 0.00

1
30
2
0
3984

 0.00
genus

species

 0.00
3986
30
1

91836
32
1
705
107
clade

 0.02


 0.00
order
1
1
4
0
41943


 0.00
family
1
1
3
0
4027

4028
0
2
1
1
genus

 0.00


 0.00
species
1
1
28964

41944
0
26
71
1
order

 0.00

3931
0
12
1
14
family

 0.00

11
1
14
2
1699513

 0.00
subfamily

tribe

 0.00
1699522
0
3
1
3


 0.00
genus
3
1
2
178174
0


 0.00
species
1
3
219896

tribe

 0.00
0
1699524
3
1
5


 0.00
genus
2
1
5
3932
0

species

 0.00
71139
1
5

1699523
0
4
1
4
tribe

 0.00

3
4
1
0
1705102

 0.00
clade

2
4
1
178132
0

 0.00
genus

178133
1
4
species

 0.00

0
3934
5
47
1
family

 0.00


 0.00
subfamily
4
1
47
1585427
0

1585433
0
1
47
3
tribe

 0.00


 0.00
genus
1
47
2
238243
0

47
1
13055

 0.00
species

family

 0.00
0
39998
1
1
5


 0.00
subfamily
1
1
4
0
2653904

1
1
3
2653918
0

 0.00
tribe

0
113478
2
1
1
genus

 0.00

species

 0.00
1160597
1
1

family

 0.00
3928
0
1
9
3


 0.00
genus
1
9
2
22662
0

22663
9
1
species

 0.00


 0.01
order
271
1
38
3699
0


 0.00
family
1
1
3
3647
0


 0.00
genus
1
1
2
3648
0

1
1
3649

 0.00
species

family

 0.01
3700
5
30
1
261

981071
1
125
1
9
tribe

 0.00

92
1
6
47
3705

 0.00
genus

species

 0.00
3712
3
2
1
4

varietas

 0.00
109376
1
1

1
16
2
13
3711

 0.00
species

1
3
51351

 0.00
subspecies

25
1
3708

 0.00
species

2
32
1
0
3725

 0.00
genus

3726
32
1
species

 0.00

3
1
15
0
981070

 0.00
tribe

50451
0
1
15
2
genus

 0.00

species

 0.00
50452
1
15

11
1
71
0
980083

 0.00
tribe

2
3701
54
1
5
genus

 0.00

1
29
3702

 0.00
species

1
18
38785

 0.00
species

species

 0.00
59689
0
2
1
5

1
5
81972

 0.00
subspecies


 0.00
genus
2
1
5
3718
0


 0.00
species
5
1
81985

3
1
12
71323
2

 0.00
genus

species

 0.00
883000
1
6

species

 0.00
90675
4
1


 0.00
tribe
3
1
3
981100
0


 0.00
genus
2
1
3
1
98005

72664
1
2
species

 0.00

3
1
42
981099
0

 0.00
tribe

0
13287
2
42
1
genus

 0.00

species

 0.00
13288
1
42

family

 0.00
301454
0
4
9
1

0
2768677
9
1
3
clade

 0.00

1168313
0
2
1
9
genus

 0.00

1
9
28532

 0.00
species


 0.01
order
220
1
25
0
41938

family

 0.01
2
3629
1
219
21

214909
0
23
1
5
subfamily

 0.00

genus

 0.00
0
108869
1
1
2


 0.00
species
1
1
108875

2
22
1
0
3640

 0.00
genus

species

 0.00
3641
22
1

0
214915
2
1
3
subfamily

 0.00


 0.00
genus
1
2
2
0
66655


 0.00
species
1
2
66656

192
1
12
214907
6

 0.00
subfamily


 0.00
genus
1
46
2
47614
0


 0.00
species
1
46
47615

4
1
2
0
47605

 0.00
genus

4
1
106335

 0.00
species

1
136
7
33
3633

 0.00
genus

species

 0.00
29730
26
1

3635
7
1
species

 0.00

5
1
29729

 0.00
species

32
1
34284

 0.00
species

species

 0.00
47622
9
1

24
1
34274

 0.00
species

39987
0
3
1
1
family

 0.00

genus

 0.00
142700
0
1
1
2

2213114
1
1
species

 0.00


 0.00
order
13
110
1
41937
0

1
104
3
0
23808

 0.00
family

0
23809
2
104
1
genus

 0.00

2768810
1
104
species

 0.00


 0.00
family
1
3
5
4011
0

2
1
2
55512
0

 0.00
genus


 0.00
species
2
1
55513

genus

 0.00
23461
0
1
1
2

29780
1
1
species

 0.00

3
1
4
0
23513

 0.00
family


 0.00
subfamily
3
1
3
0
1728959

2
2706
3
1
2
genus

 0.00

species

 0.00
85681
1
1

no rank

 0.00
91834
0
52
1
8

0
403667
52
1
7
order

 0.00

family

 0.00
3602
0
6
52
1

52
1
5
2304100
0

 0.00
tribe

genus

 0.00
3603
7
4
1
52


 0.00
species
1
5
96939

103349
1
28
species

 0.00

12
1
29760

 0.00
species

0
3524
1
117
21
order

 0.00


 0.00
family
5
1
30
3615
0


 0.00
subfamily
4
1
30
1110380
0


 0.00
tribe
3
1
30
0
1110385

46786
0
1
30
2
genus

 0.00

30
1
137693

 0.00
species

1804623
0
12
86
1
family

 0.00


 0.00
subfamily
1
1
4
0
1804621


 0.00
genus
3
1
1
0
3554


 0.00
species
2
1
1
161934
0

1
1
3555

 0.00
subspecies

0
1307796
85
1
7
subfamily

 0.00

tribe

 0.00
0
1307775
1
3
3

genus

 0.00
3561
0
1
3
2

3562
1
3
species

 0.00


 0.00
tribe
3
1
82
0
1307774

genus

 0.00
0
3558
1
82
2

1
82
63459

 0.00
species

0
3563
3
1
1
family

 0.00

0
3564
2
1
1
genus

 0.00

1
1
107608

 0.00
species

0
41768
1
14
20
order

 0.00

1
9
7
0
3465

 0.00
family

3
1
8
0
1462614

 0.00
subfamily


 0.00
genus
2
1
8
3468
0

8
1
3469

 0.00
species

0
41766
3
1
1
subfamily

 0.00

0
3466
2
1
1
genus

 0.00

species

 0.00
3467
1
1

3440
0
1
4
8
family

 0.00

1
1
4
1463138
0

 0.00
subfamily

tribe

 0.00
1463144
0
3
1
1


 0.00
genus
2
1
1
3448
0


 0.00
species
1
1
3449

subfamily

 0.00
1463137
0
3
3
1

genus

 0.00
3450
0
2
3
1

218851
1
3
species

 0.00


 0.00
family
4
1
1
0
41773

1461176
0
3
1
1
subfamily

 0.00

2976111
0
1
1
2
tribe

 0.00

1
1
63350

 0.00
genus


 0.00
order
4
4
1
261007
0

family

 0.00
0
4410
4
1
3

2
4
1
4418
0

 0.00
genus

4
1
210225

 0.00
species


 0.00
clade
1
20
18
1437180
0

17
1
20
0
58019

 0.00
class

16
20
1
3313
0

 0.00
subclass

0
2821352
17
1
8
clade

 0.00

17
1
7
0
1446380

 0.00
order

17
1
6
3318
0

 0.00
family

3337
1
11
1
3
genus

 0.00

139271
0
1
10
2
subgenus

 0.00


 0.00
species
10
1
3352

3321
0
2
1
6
genus

 0.00

1
6
3322

 0.00
species

clade

 0.00
2821351
0
7
3
1

0
1446379
3
1
6
order

 0.00

2
1
1
0
25623

 0.00
family

25628
1
1
genus

 0.00


 0.00
family
2
1
3
3367
0


 0.00
genus
2
1
2
13414
0

1
2
89191

 0.00
species

phylum

 0.00
3041
1
71
1
37

4
8
1
34155
0

 0.00
no rank


 0.00
family
3
8
1
41878
0

genus

 0.00
41879
0
2
1
8

species

 0.00
41880
8
1

0
2692248
15
1
38
clade

 0.00

1
4
18
0
75966

 0.00
class

5
1
1
0
2507901

 0.00
order


 0.00
family
1
1
4
2507902
0

0
13786
3
1
1
genus

 0.00

no rank

 0.00
0
285719
2
1
1

1
1
1825957

 0.00
species

no rank

 0.00
0
75981
8
1
2

2682734
0
1
1
3
clade

 0.00


 0.00
genus
1
1
2
0
41299

species

 0.00
41300
1
1

0
2511161
1
1
4
clade

 0.00

genus

 0.00
41891
0
3
1
1

no rank

 0.00
2688356
0
1
1
2


 0.00
species
1
1
2315456

order

 0.00
35460
0
1
1
4


 0.00
family
3
1
1
0
35461

0
2511126
1
1
2
clade

 0.00

genus

 0.00
3071
1
1

class

 0.00
0
3166
11
1
19

11
1
18
2812636
0

 0.00
clade


 0.00
order
4
1
2
35491
0

family

 0.00
0
52675
1
2
3


 0.00
genus
2
2
1
0
52687

52688
1
2
species

 0.00


 0.00
order
13
9
1
0
3042

3
1
4
0
3065

 0.00
family


 0.00
genus
1
3
3
3066
0

2
3
1
0
3067

 0.00
species

3068
1
3
forma

 0.00


 0.00
family
5
3
1
3051
0

genus

 0.00
0
47780
2
1
1

47781
1
1
species

 0.00


 0.00
genus
2
2
1
3052
0

1
2
3055

 0.00
species


 0.00
family
3
3
1
0
3043

0
3044
2
3
1
genus

 0.00


 0.00
species
1
3
257627


 0.00
class
5
1
18
33103
0

17
1
5
0
2546214

 0.00
clade


 0.00
order
5
1
16
33104
0

suborder

 0.00
2791031
0
11
1
4

family

 0.00
379500
0
2
1
6

2791007
0
5
1
2
tribe

 0.00

0
170436
1
1
2
genus

 0.00

1
1
170437

 0.00
species


 0.00
genus
2
1
1
0
325644

325645
1
1
species

 0.00

35432
0
1
2
4
family

 0.00

0
76312
3
2
1
genus

 0.00


 0.00
species
2
1
2
0
589157

2
1
177073

 0.00
varietas


 0.00
suborder
4
1
1
0
2791030


 0.00
family
1
1
3
35434
0

1
1
2
121087
0

 0.00
genus

species

 0.00
121088
1
1


 0.00
class
5
1
1
0
1035538

order

 0.00
13792
0
1
1
4

family

 0.00
0
1525212
3
1
1

1
1
2
0
70447

 0.00
genus


 0.00
species
1
1
70448

0
2302911
7
1
5
class

 0.00

2302912
0
1
7
4
order

 0.00

7
1
3
0
2302913

 0.00
family

2
7
1
2302914
0

 0.00
genus


 0.00
species
7
1
1764295

no rank

 0.00
0
2787854
1
5
8


 0.00
no rank
7
1
5
28384
0

2
81077
4
1
4
no rank

 0.00

1
1
32630

 0.00
species


 0.00
no rank
1
1
2
29278
0


 0.00
species
1
1
2797846


 0.00
no rank
2
1
1
0
36549

species

 0.00
45202
1
1


 79.86
3222922
1

superkingdom

 0.00
2157
0
111
71
1

clade

 0.00
1935183
0
1
1
3

phylum

 0.00
0
1936272
1
1
2


 0.00
species
1
1
2876573

8
3
1
0
2283796

 0.00
phylum

class

 0.00
183967
0
7
1
3


 0.00
no rank
1
1
2
1234666
0

species

 0.00
1495144
1
1

order

 0.00
2301
0
4
2
1

3
2
1
0
90142

 0.00
family

74968
0
1
2
2
genus

 0.00


 0.00
species
1
2
74969

2
1
6
48510
0

 0.00
no rank

115547
1
6
species

 0.00

clade

 0.00
1783276
0
7
3
1

6
3
1
0
192989

 0.00
phylum


 0.00
class
3
1
5
2885752
0

0
1890940
4
1
3
order

 0.00

0
1890942
1
3
3
family

 0.00


 0.00
genus
1
3
2
1890407
0


 0.00
species
1
3
1577684

phylum

 0.00
28890
0
48
1
41

1
25
16
0
2283794

 0.00
clade


 0.00
class
15
25
1
183925
0

order

 0.00
2158
0
14
1
25


 0.00
family
25
1
13
2159
2

3
1
2
0
2316

 0.00
genus

no rank

 0.00
0
2643926
1
2
2


 0.00
species
2
1
1789762

1
2160
1
2
2
genus

 0.00

1
1
2731220

 0.00
species

6
2172
7
19
1
genus

 0.00

6
1
230361

 0.00
species

species

 0.00
294671
1
2

3
4
1
2638681
0

 0.00
no rank

species

 0.00
1609968
3
1

species

 0.00
1452351
1
1

species

 0.00
2173
1
1

0
2290931
31
16
1
clade

 0.00

class

 0.00
0
224756
1
7
11

7
1
10
0
94695

 0.00
order

family

 0.00
0
2206
6
1
7

genus

 0.00
2207
0
6
1
6


 0.00
species
2
2
1
0
170861


 0.00
strain
2
1
1434111


 0.00
no rank
2
1
2644672


 0.00
species
2
2
1
2208
1

1434107
1
1
strain

 0.00

2
1
1
0
588815

 0.00
no rank

1
1
2759912

 0.00
species

class

 0.00
183963
1
19
9
1

0
1644060
11
5
1
order

 0.00

1644061
0
10
5
1
family

 0.00

1
1
3
1699375
0

 0.00
genus


 0.00
no rank
2
1
1
0
2643314


 0.00
species
1
1
2953881

0
2256
1
3
3
genus

 0.00

44930
0
2
3
1
species

 0.00

797304
3
1
strain

 0.00

0
88723
3
1
1
genus

 0.00

2
1
1
0
69525

 0.00
species

797303
1
1
strain

 0.00

1
2235
1
3
7
order

 0.00


 0.00
family
1
1
3
1963270
0


 0.00
genus
1
1
2
2249
0


 0.00
species
1
1
224402

family

 0.00
0
2236
1
1
3

genus

 0.00
0
2239
1
1
2


 0.00
species
1
1
2039234

clade

 0.00
0
1783275
1
17
42

10
1
24
28889
0

 0.00
phylum

0
183924
1
10
23
class

 0.00

order

 0.00
2281
0
12
8
1

11
8
1
118883
0

 0.00
family

4
1
3
0
2284

 0.00
genus

2641160
0
1
4
2
no rank

 0.00

2512240
1
4
species

 0.00

0
12914
3
1
1
genus

 0.00

species

 0.00
563177
0
1
1
2

933801
1
1
strain

 0.00

genus

 0.00
2017961
0
2
1
1

species

 0.00
1670455
1
1


 0.00
genus
2
1
2
41674
0

species

 0.00
2824673
2
1


 0.00
order
1
1
5
0
2266


 0.00
family
4
1
1
2267
0


 0.00
genus
3
1
1
164450
0

species

 0.00
164451
0
2
1
1

1
1
572478

 0.00
strain

order

 0.00
0
685948
5
1
1


 0.00
family
1
1
4
685949
0

3
1
1
0
685950

 0.00
genus

species

 0.00
683846
0
2
1
1

1163730
1
1
strain

 0.00

14
1
5
0
651137

 0.00
phylum

5
2
1
31932
0

 0.00
order

family

 0.00
1
338190
1
2
4


 0.00
genus
1
1
3
0
1007082

no rank

 0.00
0
2641124
1
1
2

species

 0.00
2259673
1
1

no rank

 0.00
0
651142
1
1
3

2
1
1
1078904
0

 0.00
genus


 0.00
species
1
1
1078905

class

 0.00
0
1643678
1
2
5

0
1033996
4
2
1
order

 0.00


 0.00
family
1
2
3
0
1033997


 0.00
genus
1
1
1826864

1
1
497726

 0.00
genus

1
2
3
928852
0

 0.00
phylum

2
1
2
0
1700837

 0.00
no rank

1
2
2026714

 0.00
species

superkingdom

 0.28
1024
2
11500
1
2708


 0.00
phylum
1
28
40
0
203691

39
28
1
203692
0

 0.00
class

8
1
5
0
1643686

 0.00
order


 0.00
family
1
5
7
0
143786

genus

 0.00
0
29521
1
5
6


 0.00
species
1
1
2
52584
0

strain

 0.00
1133568
1
1


 0.00
species
1
3
1287055

species

 0.00
84377
0
2
1
1

1045858
1
1
strain

 0.00

6
1
8
1643688
0

 0.00
order

170
0
8
1
5
family

 0.00


 0.00
genus
4
1
8
171
5

2
1
1
2633828
0

 0.00
no rank


 0.00
species
1
1
2838237

2
1
173

 0.00
species


 0.00
order
24
1
15
0
136

1
2
4
0
2845253

 0.00
family

genus

 0.00
157
0
3
1
2


 0.00
species
1
1
2787628


 0.00
species
1
1
744515

family

 0.00
1643685
0
12
10
1

4
1
6
138
1

 0.00
genus

1
1
2
2649934
0

 0.00
no rank


 0.00
species
1
1
293092

140
1
1
species

 0.00

1
1
2
229155
0

 0.00
species

1
1
1104446

 0.00
strain


 0.00
genus
5
6
1
1
64895

species

 0.00
29518
1
1

2
1
2
64897
0

 0.00
species

2
1
521010

 0.00
strain


 0.00
species
1
2
29519

2
1
3
0
2791015

 0.00
family

399320
0
2
1
2
genus

 0.00

species

 0.00
1129264
1
2

0
2951104
4
1
1
family

 0.00

genus

 0.00
2951107
0
1
1
3

1
1
2
150829
0

 0.00
species

strain

 0.00
545695
1
1

1
57723
5
1
8
phylum

 0.00

2
1
1
305072
0

 0.00
no rank


 0.00
species
1
1
1978231

no rank

 0.00
0
458031
1
1
3

clade

 0.00
0
1660250
2
1
1


 0.00
species
1
1
1660251


 0.00
class
2
1
2
0
1562566

genus

 0.00
458032
1
2


 0.00
phylum
20
1
28
0
32066

class

 0.00
0
203490
28
1
19

order

 0.00
203491
0
1
28
18

0
1129771
8
1
8
family

 0.00

2
32067
7
8
1
genus

 0.00

1
1
109328

 0.00
species

157691
1
1
species

 0.00

no rank

 0.00
2633022
0
1
1
2

1
1
712357

 0.00
species

2
1
157687

 0.00
species


 0.00
species
1
1
157688

family

 0.00
203492
0
20
1
9

genus

 0.00
2
848
20
1
8

2663009
2
1
species

 0.00


 0.00
species
1
2
1755100

1
861
2
4
1
species

 0.00

469617
3
1
strain

 0.00

no rank

 0.00
0
2648384
2
1
1

1
1
671211

 0.00
species


 0.00
species
9
1
851

0
2323
1
14
34
no rank

 0.00

1
14
33
1783234
0

 0.00
clade

0
1783273
10
1
4
clade

 0.00

363464
0
1
1
3
phylum

 0.00

no rank

 0.00
0
1046947
1
1
2

1
1
1862133

 0.00
species

1794811
0
3
1
1
clade

 0.00


 0.00
phylum
1
1
2
0
1752727


 0.00
species
1
1
2026716

3
1
2
1794810
0

 0.00
clade


 0.00
phylum
2
1
2
0
1752722

species

 0.00
2026804
2
1

1
2
3
95901
0

 0.00
clade

1104668
0
1
2
2
no rank

 0.00

1
2
1619077

 0.00
species

phylum

 0.00
95818
1
11
6
1

1
2
3
1895827
0

 0.00
no rank

species

 0.00
713059
1
1


 0.00
species
1
1
713051


 0.00
genus
1
1
2
0
2905967

2841264
1
1
species

 0.00

2093818
0
5
1
2
class

 0.00


 0.00
order
2
1
4
2093819
0

3
2
1
2093822
0

 0.00
family


 0.00
genus
2
1
2
0
2093823


 0.00
species
2
1
2572088

0
221235
5
1
1
phylum

 0.00

1
1
4
0
2900568

 0.00
order

family

 0.00
0
2900569
3
1
1

0
2900570
1
1
2
genus

 0.00

species

 0.00
2735562
1
1


 0.00
phylum
1
1
3
0
1619053

no rank

 0.00
1801912
0
1
1
2


 0.00
species
1
1
2030811


 0.00
phylum
10
1
2
200918
0

class

 0.00
0
188708
9
1
2

order

 0.00
2419
0
1
1
4


 0.00
family
1
1
3
1643950
0


 0.00
genus
2
1
1
0
2420


 0.00
no rank
1
1
2676525

order

 0.00
1643947
0
1
1
4

1
1
3
0
1643949

 0.00
family

1
1
2
2778400
0

 0.00
genus

2108365
1
1
species

 0.00

phylum

 0.09
315
1224
1
3452
994

7
1
10
0
1553900

 0.00
class


 0.00
order
4
1
2
2024973
0


 0.00
family
2
1
3
0
2024974

2
1
2
0
2698753

 0.00
genus


 0.00
species
2
1
2493639

213481
0
1
5
5
order

 0.00

family

 0.00
0
213483
1
5
4


 0.00
genus
3
1
5
958
0

2835041
1
5
species

 0.00

0
1
2633795

 0.00
no rank


 0.01
class
527
1
264
28211
61

120
208
1
356
47

 0.01
order

4
1
1
0
255475

 0.00
family

1
1
3
182269
0

 0.00
genus

0
2638230
1
1
2
no rank

 0.00

species

 0.00
2906072
1
1


 0.00
family
1
15
6
5
2831106

genus

 0.00
46913
5
10
1
5

400770
1
1
species

 0.00

2
1
2801335

 0.00
species

1
2
2
196773
1

 0.00
no rank


 0.00
species
1
1
2978393

family

 0.00
1
69277
1
7
10

2
1
1
2911176
0

 0.00
genus

1670800
1
1
species

 0.00

245876
0
3
1
1
genus

 0.00


 0.00
no rank
2
1
1
0
2641084

1
1
2599600

 0.00
species

68287
0
4
4
1
genus

 0.00


 0.00
species
2
1
2725666

1
1
381

 0.00
species

325217
1
1
no rank

 0.00

41
1
30
82115
1

 0.00
family


 0.00
genus
1
2
4
323620
0

352475
1
1
species

 0.00

2643062
0
1
1
2
no rank

 0.00

879274
1
1
species

 0.00

0
227292
10
1
6
no rank

 0.00


 0.00
genus
2
1
1
106591
0

species

 0.00
106592
1
1

7
1
5
28105
0

 0.00
genus

species group

 0.00
0
663276
2
1
1

1
1
380

 0.00
species

0
2613772
1
1
2
no rank

 0.00

species

 0.00
794846
1
1

194963
2
2
1
3
species

 0.00

1408224
1
1
strain

 0.00

227290
12
15
32
1
no rank

 0.00

379
5
12
1
18
genus

 0.00

0
384
1
2
3
species

 0.00


 0.00
no rank
1
1
386

no rank

 0.00
387
1
1

species

 0.00
1076926
1
1

2267833
1
2
species

 0.00


 0.00
species
1
1
1368430

0
2613769
7
1
5
no rank

 0.00

1435607
1
1
species

 0.00


 0.00
species
1
1
1571470

1
3
2917727

 0.00
species

species

 0.00
2795216
2
1

1
357
1
2
2
genus

 0.00

2735528
1
1
species

 0.00


 0.00
family
7
1
3
0
45404

no rank

 0.00
45405
0
1
2
3

1978229
1
1
species

 0.00

2572036
1
1
species

 0.00

120652
0
3
1
1
genus

 0.00

0
199596
1
1
2
species

 0.00

strain

 0.00
395965
1
1

no rank

 0.00
0
119042
6
3
1


 0.00
genus
3
1
2
212791
0

2
2
1
0
2632691

 0.00
no rank


 0.00
species
1
2
1894999


 0.00
genus
1
1
2
2874214
0

1
1
708113

 0.00
species

family

 0.00
118882
0
6
1
8


 0.00
no rank
7
1
6
2826938
1


 0.00
genus
5
1
6
234
1

1
1
271865

 0.00
species

species

 0.00
529
1
1


 0.00
species
1
1
370111

2
1
1
2632610
0

 0.00
no rank

species

 0.00
2840456
1
1

family

 0.00
0
655351
1
1
4


 0.00
genus
1
1
3
0
655352

2631913
0
1
1
2
no rank

 0.00

species

 0.00
1798205
1
1

2
1
3
0
2843305

 0.00
family

genus

 0.00
0
1632780
2
2
1

1868589
1
2
species

 0.00

family

 0.00
0
2831100
5
7
1


 0.00
genus
1
7
4
85413
1

4
2653178
1
6
3
no rank

 0.00


 0.00
species
1
1
2020412

1
1
1842539

 0.00
species

3
1
6
45401
0

 0.00
family

genus

 0.00
1
81
2
2
1


 0.00
species
1
1
84

29407
0
1
1
3
genus

 0.00

1
1
2
2619116
0

 0.00
no rank


 0.00
species
1
1
674703

1
1
2
335928
0

 0.00
family

1
1
279

 0.00
genus

0
31993
4
1
1
family

 0.00

0
133
1
1
3
genus

 0.00

no rank

 0.00
0
2625913
2
1
1

187303
1
1
species

 0.00

1
33
10
5
119045

 0.00
family

15
407
6
1
24
genus

 0.00

2615210
2
1
8
4
no rank

 0.00


 0.00
species
1
1
2067957

species

 0.00
2953579
4
1

1
1
2603276

 0.00
species

1
1
570505

 0.00
species


 0.00
genus
2
1
2
1
2282523

223967
1
1
species

 0.00


 0.00
genus
2
1
186650


 0.00
family
2
1
2
772
0

1
2
773

 0.00
genus

12
35
1
41294
9

 0.00
family


 0.00
genus
1
1
1395974


 0.00
genus
1
25
10
15
374

2840469
1
1
species

 0.00

species

 0.00
1355477
2
1


 0.00
no rank
3
1
3
1
2631580

1
1
858422

 0.00
species

2170627
1
1
species

 0.00

species

 0.00
1274631
1
1


 0.00
species
1
1
375

species

 0.00
1404864
1
1

1
1
1437360

 0.00
species

order

 0.00
0
1921002
7
5
1

2100208
0
5
1
6
family

 0.00

4
1
2
0
2100211

 0.00
genus

species

 0.00
244581
1
4


 0.00
genus
3
1
1
0
1509243

1509244
0
2
1
1
species

 0.00

1
1
1414854

 0.00
strain

18
1
33
0
204458

 0.00
order


 0.00
family
33
1
17
2
76892

genus

 0.00
9
75
5
1
15

69395
1
1
species

 0.00


 0.00
species
2
1
2010972

species

 0.00
2708539
1
1

species

 0.00
88688
1
2

1
1
3
0
76890

 0.00
genus

0
78587
2
1
1
species

 0.00

1
1
573065

 0.00
strain

9
41275
1
15
8
genus

 0.00


 0.00
no rank
1
3
3
1
2622653


 0.00
species
1
1
2861285

1
1
1827469

 0.00
species


 0.00
species
1
1
2
0
74313


 0.00
strain
1
1
633149

2774189
1
1
species

 0.00

species

 0.00
293
1
1

0
766
35
1
28
order

 0.00


 0.00
family
23
1
13
0
775

23
1
12
33988
0

 0.00
tribe

0
69474
1
7
2
genus

 0.00

species

 0.00
784
1
7

1
16
9
0
780

 0.00
genus


 0.00
species group
2
1
1
0
1129742

species

 0.00
788
1
1


 0.00
no rank
2
1
2
0
114295

species

 0.00
2698648
1
2

species group

 0.00
114277
0
12
1
3

786
0
12
1
2
species

 0.00

293614
1
12
strain

 0.00

1
1
114292

 0.00
species group

family

 0.00
0
942
1
12
14

0
768
1
2
4
genus

 0.00

0
106179
1
2
3
species group

 0.00


 0.00
species
2
1
2
948
1

1
1
1392506

 0.00
strain

0
952
6
1
8
tribe

 0.00

5
1
8
953
2

 0.00
genus

4
1
6
1
2640676

 0.00
no rank


 0.00
species
2
1
169402


 0.00
species
1
1
2584115


 0.00
species
1
2
282263

genus

 0.00
943
0
3
1
2

0
106178
2
2
1
species group

 0.00

species

 0.00
779
2
1

order

 0.00
0
204441
8
1
14


 0.00
family
3
1
1
597359
0

0
1647175
1
1
2
genus

 0.00


 0.00
species
1
1
2775080

0
941843
2
1
1
no rank

 0.00


 0.00
species
1
1
2026786

433
0
5
5
1
family

 0.00

genus

 0.00
1
125216
1
2
2

2768162
1
1
species

 0.00

genus

 0.00
434
0
3
1
2

3
1
438

 0.00
species

3
1
1
0
2829815

 0.00
family


 0.00
genus
2
1
1
204447
0

species

 0.00
1789672
1
1

1
1
5
0
2800060

 0.00
order


 0.00
family
1
1
4
69657
0


 0.00
genus
1
1
3
85
0

1
1
2
0
2630699

 0.00
no rank

1
1
87

 0.00
species

order

 0.00
204455
8
38
43
1

1
2854170
1
10
14
family

 0.00


 0.00
genus
2
1
1
302485
0

species

 0.00
1844006
1
1

genus

 0.00
0
2854186
2
1
1

1402135
1
1
species

 0.00


 0.00
genus
1
1
2
0
2433

species

 0.00
1891787
1
1

1
2
74030

 0.00
genus

0
191028
2
1
1
genus

 0.00

species

 0.00
506591
1
1

3
1
4
0
60136

 0.00
genus

3
3
1
0
196795

 0.00
no rank


 0.00
species
1
1
2867023

2070369
1
2
species

 0.00


 0.00
family
21
1
24
3
31989


 0.00
genus
2
1
1
2831925
0

species

 0.00
2605946
1
1

2
1
1
0
2613960

 0.00
genus

1
1
2613965

 0.00
species


 0.00
genus
1
15
7
265
5

147645
3
1
species

 0.00

2688777
0
3
1
3
no rank

 0.00

species

 0.00
2500532
1
2

species

 0.00
2760307
1
1

1545044
1
1
species

 0.00

1
3
59779

 0.00
species

genus

 0.00
0
1648497
3
1
1

2
1
1
0
2634418

 0.00
no rank


 0.00
species
1
1
1886600

2
1
1
0
1209946

 0.00
genus

564137
1
1
species

 0.00

1855413
0
2
1
1
genus

 0.00

1
1
1267768

 0.00
species

1
1
2
366614
0

 0.00
genus

195105
1
1
species

 0.00


 0.00
no rank
1
1
2
0
285892

2899152
1
1
species

 0.00

204457
13
1
133
33
order

 0.00


 0.00
family
25
1
117
38
41297

genus

 0.00
1
165697
1
4
3


 0.00
no rank
2
3
1
1
2614943

1
2
292913

 0.00
species

genus

 0.00
165695
1
3
1
3

species

 0.00
76947
1
1

13690
1
1
species

 0.00


 0.00
genus
15
1
71
13687
30

13689
1
1
species

 0.00

15
196159
5
1
33
no rank

 0.00


 0.00
species
1
11
2565555

3
1
1517554

 0.00
species

species

 0.00
28214
1
1

3
1
1523415

 0.00
species

1609977
1
1
species

 0.00


 0.00
species
2
1
1
0
397260

strain

 0.00
1123269
1
1

1549858
1
1
species

 0.00


 0.00
species
1
1
2759526

152682
1
1
species

 0.00

1
1
2938948

 0.00
species


 0.00
species
1
1
1560345

3
1
1
1434046
0

 0.00
genus

2
1
1
0
2614947

 0.00
no rank

2584094
1
1
species

 0.00

3
1
2
335929
1

 0.00
family

1
1
2
1855416
0

 0.00
genus

species

 0.00
225971
1
1

family

 0.00
2820280
0
1
1
4

0
1508451
3
1
1
genus

 0.00

1
1
2
2629224
0

 0.00
no rank

2862331
1
1
species

 0.00

class

 0.04
86
1236
1772
1
411


 0.00
order
17
10
1
135613
0

1
1
3
0
1738654

 0.00
family

0
1738655
1
1
2
genus

 0.00

1
1
1548547

 0.00
species

4
1
1
0
72276

 0.00
family

3
1
1
133193
0

 0.00
genus


 0.00
species
2
1
1
0
351052


 0.00
strain
1
1
187272


 0.00
family
7
1
5
1046
1

2
67575
4
6
1
genus

 0.00

no rank

 0.00
115860
0
2
2
1

2
1
1869214

 0.00
species

species

 0.00
2498451
2
1

1
1
4
1676141
0

 0.00
family

0
1676142
3
1
1
genus

 0.00


 0.00
no rank
2
1
1
0
2613841

2771012
1
1
species

 0.00

4
1
5
1
1706369

 0.00
order

4
1
3
0
1706373

 0.00
family

genus

 0.00
0
48073
3
1
3

species

 0.00
86173
1
2

1
1
48074

 0.00
species

2
135614
86
1
29
order

 0.00

4
1
4
1
1775411

 0.00
family


 0.00
genus
2
1
2
1
242605

1
1
2589080

 0.00
species

1
1
75309

 0.00
genus

1
80
24
32033
7

 0.00
family

genus

 0.00
338
2
9
1
5


 0.00
species
1
5
2
3
339

no rank

 0.00
487886
2
1

343
1
2
2
1
species

 0.00

487909
1
1
no rank

 0.00

83618
0
1
1
3
genus

 0.00

species

 0.00
415229
0
2
1
1


 0.00
strain
1
1
1045855

0
83614
3
7
1
genus

 0.00


 0.00
species
5
1
1176533

2
1
2006110

 0.00
species

9
1
48
19
40323

 0.00
genus

3
1
2
1
196198

 0.00
no rank

1
2
1904944

 0.00
species


 0.00
species
5
1
216778

5
21
1
995085
2

 0.00
species group

no rank

 0.00
0
2961925
2
1
1

2072405
1
1
species

 0.00


 0.00
species
1
18
2
17
40324

1
1
868597

 0.00
strain

genus

 0.00
2
68
3
1
8

species

 0.00
69
1
1


 0.00
species
1
5
435897

no rank

 0.00
0
118884
1
1
3


 0.00
clade
2
1
1
0
1076628

1076629
1
1
species

 0.00


 0.00
order
1
38
29
0
135622

5
5
1
0
267889

 0.00
family

5
1
4
28228
0

 0.00
genus

196834
0
3
1
5
no rank

 0.00

species

 0.00
2583805
2
1

2689569
1
3
species

 0.00


 0.00
family
1
11
6
267888
0


 0.00
genus
5
11
1
3
53246


 0.00
no rank
2
1
1
0
194690

species

 0.00
2894202
1
1

288
0
1
7
2
species

 0.00

strain

 0.00
342610
1
7

267893
0
1
1
4
family

 0.00

1
1
3
0
135575

 0.00
genus


 0.00
no rank
2
1
1
2614829
0

species

 0.00
1874361
1
1

family

 0.00
267890
0
15
1
6

1
15
5
22
7

 0.00
genus

1028752
1
1
species

 0.00

1
4
332186

 0.00
species


 0.00
no rank
2
3
1
196818
2

1
1
2908650

 0.00
species

72275
0
7
6
1
family

 0.00

4
5
1
0
2903219

 0.00
no rank

226
0
1
5
3
genus

 0.00

no rank

 0.00
2614992
1
3

2
1
28108

 0.00
species

genus

 0.00
0
2650549
2
1
1

2259620
1
1
species

 0.00


 0.01
order
92
1
531
2
72274

family

 0.01
11
135621
528
1
88

genus

 0.00
2901189
0
2
1
1

797277
1
1
species

 0.00

3
1
4
0
351

 0.00
subfamily


 0.00
genus
3
1
3
352
0

2
353
2
3
1
species

 0.00

1
1
1328314

 0.00
strain

255
286
1
509
75
genus

 0.01

species

 0.00
122355
3
1

485898
1
1
species

 0.00


 0.00
species group
83
1
13
136843
9


 0.00
species
2
1
47878


 0.00
species
4
1
76760

species

 0.00
47883
1
1


 0.00
species
1
1
651740

1114970
1
1
species

 0.00

42
294
2
1
54
species

 0.00


 0.00
strain
1
12
216595


 0.00
species
2
1
2
200451
1

1
1
1282356

 0.00
strain

1
1
200450

 0.00
species

7
1
76758

 0.00
species

76761
1
1
species

 0.00

species

 0.00
53407
1
1

species

 0.00
2830842
1
1

2842349
1
1
species

 0.00


 0.00
species
1
1
95300


 0.00
no rank
26
85
1
196821
38

species

 0.00
1758730
1
1

species

 0.00
2590776
1
1


 0.00
species
1
2
2861799

2898483
2
1
species

 0.00

2597770
1
1
species

 0.00


 0.00
species
2
1
2971912

species

 0.00
253237
1
3


 0.00
species
1
2
2604941

species

 0.00
2083054
1
3

1
1
2749807

 0.00
species

species

 0.00
306
1
2

2745519
9
1
species

 0.00

species

 0.00
1283291
3
1

species

 0.00
2605424
1
1


 0.00
species
1
1
2706126

2730851
1
1
species

 0.00

1
1
2749808

 0.00
species


 0.00
species
1
1
2320270


 0.00
species
1
1
2498848

2662033
1
1
species

 0.00

1
1
2901380

 0.00
species

1
1
1344094

 0.00
species

1
1
2926671

 0.00
species


 0.00
species
2
1
2654238

species

 0.00
2054919
1
3


 0.00
species
1
1
2842346

species

 0.00
359110
1
1

2666183
1
1
species

 0.00

2867264
2
1
species

 0.00


 0.00
species
1
2
2906062

3
10
1
136842
1

 0.00
species group

1
1
587753

 0.00
species


 0.00
species
1
8
296

species

 0.00
930166
1
1


 0.00
species group
18
1
5
136845
0


 0.00
species
1
1
47880

species

 0.00
12
303
2
13
1


 0.00
strain
1
1
1331671

4
1
47885

 0.00
species


 0.00
species
6
1
2745518

species

 0.00
216142
1
1

species

 0.00
198620
2
1

species

 0.00
2745511
1
1


 0.00
species
1
1
2961896

species group

 0.00
136849
4
3
1
16

species

 0.00
1190415
1
2

species

 0.00
33069
1
10

14
1
6
136841
0

 0.00
species group

0
1232139
2
4
1
species subgroup

 0.00


 0.00
species
1
4
301

287
2
1
species

 0.00

species

 0.00
300
1
1


 0.00
species
1
7
43263

1
1
46677

 0.00
species


 0.00
genus
4
1
6
0
2901164

species group

 0.00
2
136846
1
4
5

74829
0
1
1
2
species

 0.00

1123016
1
1
strain

 0.00

0
578833
1
1
2
species subgroup

 0.00

316
1
1
species

 0.00


 0.00
family
3
1
1
0
2887365

2742
0
2
1
1
genus

 0.00

1033846
1
1
species

 0.00


 0.00
order
1
178
86
91347
4

1
140
50
45
543

 0.00
family

0
561
1
8
2
genus

 0.00

species

 0.00
562
1
8


 0.00
no rank
9
6
1
191675
0


 0.00
clade
6
1
8
84563
0

1
4
2
1449912
0

 0.00
genus

species

 0.00
1345115
1
4


 0.00
genus
2
1
1
0
1906661

species

 0.00
1070130
1
1


 0.00
clade
3
1
1
84564
0

1699619
0
1
1
2
genus

 0.00

1
1
1594731

 0.00
species

1
1
2
0
579

 0.00
genus

61648
1
1
species

 0.00

genus

 0.00
158876
0
2
1
2


 0.00
species
1
2
158877

1
46
6
1
2890311

 0.00
no rank

genus

 0.00
570
35
45
1
5


 0.00
species
1
4
1463165

species

 0.00
571
1
1

1134687
1
2
species

 0.00

species

 0.00
573
1
3

0
590
4
1
4
genus

 0.00

3
1
4
28901
0

 0.00
species

4
1
2
3
59201

 0.00
subspecies

no rank

 0.00
57743
1
1

genus

 0.00
0
413496
1
1
2

species

 0.00
28141
1
1

genus

 0.00
547
4
4
1
14

354276
4
3
1
10
species group

 0.00

158836
2
1
species

 0.00


 0.00
species
4
1
550

genus

 0.00
83654
1
1
4
3

2627398
1
1
no rank

 0.00


 0.00
species
1
2
83655

1
1
3
0
1330547

 0.00
genus

0
2632876
2
1
1
no rank

 0.00

1
1
2067958

 0.00
species


 0.00
genus
1
2
3
544
0

0
1344959
2
1
2
species group

 0.00

species

 0.00
546
2
1

genus

 0.00
0
1177214
3
1
1

2
1
1
2735709
0

 0.00
no rank

species

 0.00
2864218
1
1

2
3
1
0
2815296

 0.00
genus

2579935
1
3
species

 0.00

genus

 0.00
82976
0
2
1
1

1
1
82977

 0.00
species

36866
0
1
1
2
no rank

 0.00


 0.00
species
1
1
2675795

9
1
12
0
1903411

 0.00
family

genus

 0.00
613
8
2
9
1


 0.00
species
1
1
2741499

1
2
3
0
629

 0.00
genus


 0.00
species group
1
1
1649845

1
1
631

 0.00
species


 0.00
genus
3
1
1
0
1964366

2636213
0
2
1
1
no rank

 0.00

species

 0.00
2126321
1
1


 0.00
family
7
1
4
1903410
0


 0.00
genus
1
7
3
122277
0


 0.00
species
1
6
2488639


 0.00
species
1
1
2774015

family

 0.00
0
1903412
1
1
3

0
635
2
1
1
genus

 0.00

636
1
1
species

 0.00

8
1
6
1903414
0

 0.00
family

1
1
3
0
583

 0.00
genus

no rank

 0.00
257482
0
1
1
2

species

 0.00
1921565
1
1

1
1
2
637
0

 0.00
genus

638
1
1
species

 0.00

genus

 0.00
1
586
4
1
2


 0.00
species
1
3
587

family

 0.00
0
1903409
11
1
8

1
1
2
0
551

 0.00
genus


 0.00
species
1
1
1922217


 0.00
genus
3
1
3
32199
0

1
9
3
1
2
species

 0.00

2727851
1
2
forma specialis

 0.00

1
53335
1
4
5
genus

 0.00

553
1
1
species

 0.00

no rank

 0.00
0
2630326
3
1
2

1
1
2575375

 0.00
species

2490851
1
1
species

 0.00

order

 0.00
118969
0
1
16
11

1
12
6
118968
0

 0.00
family

0
254245
1
1
2
genus

 0.00

species

 0.00
254246
1
1

genus

 0.00
0
776
3
1
11

species

 0.00
777
1
1

species

 0.00
2054173
10
1

0
444
4
4
1
family

 0.00


 0.00
genus
3
4
1
445
0

species

 0.00
45065
1
2

species

 0.00
446
1
2

order

 0.00
0
72273
1
1
5


 0.00
family
4
1
1
0
34064


 0.00
genus
3
1
1
0
262

no rank

 0.00
2610885
0
1
1
2

species

 0.00
1395624
1
1

order

 0.00
0
135615
4
2
1

family

 0.00
0
868
3
1
2


 0.00
genus
2
1
2
0
2717


 0.00
species
1
2
2718

16
1
85
2
135619

 0.00
order

28256
23
7
1
79
family

 0.00


 0.00
genus
6
56
1
39
2745

1
1
2733488

 0.00
species


 0.00
no rank
3
1
15
2609666
13

1883416
1
1
species

 0.00

1
1
2982692

 0.00
species

2733484
1
1
species

 0.00

135620
0
4
1
8
family

 0.00


 0.00
no rank
0
1
221817

3018276
genus
1
1

1
1
2895352

 0.00
species

genus

 0.00
0
28253
3
1
2

1
1
2773454

 0.00
species

383750
1
1
species

 0.00


 0.00
genus
2
1
1
0
267849


 0.00
species
1
1
2614693

135625
0
24
69
1
order

 0.00

712
21
23
1
69
family

 0.00

2
724
7
31
1
genus

 0.00

species

 0.00
729
22
1
24
2


 0.00
strain
2
1
862965

1
1
2
2609962
0

 0.00
no rank

712310
1
1
species

 0.00

1
1
727

 0.00
species

1
3
730

 0.00
species

75984
1
1
genus

 0.00


 0.00
genus
1
1
2
0
2094023


 0.00
species
1
1
738

11
1
5
416916
1

 0.00
genus

3
8
1
2
2639383

 0.00
no rank


 0.00
species
1
4
2820817

2
1
712150

 0.00
species


 0.00
species
1
2
732

genus

 0.00
0
1249016
2
1
1

1
1
1032623

 0.00
species


 0.00
genus
2
1
1
713
0


 0.00
species
1
1
715

292486
0
3
1
2
genus

 0.00


 0.00
species
1
1
762


 0.00
no rank
1
1
2685287

order

 0.02
0
2887326
1
613
53

1
613
52
5
468

 0.02
family

no rank

 0.00
0
54393
1
1
2

1889775
1
1
species

 0.00

0
475
1
173
6
genus

 0.00

species

 0.00
476
1
1

species

 0.00
34062
1
167

2685852
0
2
1
1
no rank

 0.00

species

 0.00
2904122
1
1

480
1
4
species

 0.00


 0.01
genus
29
1
337
469
124

species

 0.00
2004650
1
1

1
1
108981

 0.00
species

202954
1
1
species

 0.00

species

 0.00
756892
1
1

1324350
1
2
species

 0.00

species

 0.00
487316
1
1

108980
1
6
species

 0.00

28090
7
1
species

 0.00

species

 0.00
1871111
1
1

29430
5
1
species

 0.00

1
30
3
6
909768

 0.00
species group

48296
1
3
species

 0.00

species

 0.00
470
21
1

2053287
1
1
species

 0.00


 0.00
species
1
1
106648

no rank

 0.00
1
196816
1
20
9


 0.00
species
1
1
2853158

11
1
1646498

 0.00
species

species

 0.00
2004644
1
2

species

 0.00
2798861
1
1

1
1
2925837

 0.00
species

1808001
1
1
species

 0.00

1407071
1
1
species

 0.00

species

 0.00
1758189
1
1

40215
1
39
species

 0.00

2
95
1
94
40214

 0.00
species


 0.00
strain
1
1
1242245

134534
1
1
species

 0.00

97
1
14
36
497

 0.00
genus


 0.00
species
1
1
256325

0
334543
2
1
1
species

 0.00

259536
1
1
strain

 0.00

no rank

 0.00
196806
27
53
1
8

571800
1
2
species

 0.00

species

 0.00
1028416
6
1

species

 0.00
1699624
2
1

1
1
2733866

 0.00
species

species

 0.00
2203895
1
1


 0.00
species
1
1
56811


 0.00
species
1
13
2708350


 0.00
species
1
4
861445

species

 0.00
330922
1
2

0
135623
27
1
30
order

 0.00


 0.00
family
26
1
30
0
641

0
246861
2
1
1
genus

 0.00

1
1
673

 0.00
species

0
657
1
1
3
genus

 0.00

2
1
1
38293
0

 0.00
species

1
1
85581

 0.00
subspecies

511678
0
3
1
2
genus

 0.00

1
3
80852

 0.00
species

18
1
25
2
662

 0.00
genus


 0.00
species
1
1
666

1481923
1
1
species

 0.00

1
1
2
2614977
0

 0.00
no rank

species

 0.00
2819101
1
1

2
1
2572923

 0.00
species


 0.00
species
1
1
1481914

species group

 0.00
717610
0
3
1
4

species

 0.00
663
2
1


 0.00
species
1
2
670

species

 0.00
672
1
1


 0.00
species
1
2
246167


 0.00
species
1
1
2841925

1
1
553239

 0.00
species

species group

 0.00
0
1891919
2
1
3

1
3
300876

 0.00
species

species

 0.00
29494
1
1

4
1
676

 0.00
species

order

 0.00
0
135624
1
22
9

8
1
22
84642
0

 0.00
family

genus

 0.00
129577
0
3
1
3

no rank

 0.00
2636315
0
2
1
3

511062
3
1
species

 0.00

12
642
19
1
4
genus

 0.00

651
1
3
species

 0.00

645
1
1
species

 0.00

3
1
648

 0.00
species

2008785
0
1
4
5
class

 0.00


 0.00
order
4
1
4
0
119069

family

 0.00
206349
0
4
1
3

genus

 0.00
0
70774
2
1
4

1
4
297

 0.00
species

subphylum

 0.00
0
68525
90
1
87

class

 0.00
29547
0
42
1
66

1
66
41
0
213849

 0.00
order


 0.00
family
7
1
10
72293
0

1
10
6
209
0

 0.00
genus


 0.00
species
2
1
76936


 0.00
species
4
1
210

species

 0.00
104628
1
1


 0.00
no rank
2
1
3
0
2593540

1
3
2849641

 0.00
species

4
1
1
2932623
0

 0.00
family

1
1
3
0
57665

 0.00
genus


 0.00
species
2
1
1
194424
0


 0.00
strain
1
1
1193502

1
2
2
0
2771471

 0.00
family

202746
1
2
genus

 0.00


 0.00
family
1
22
11
72294
0


 0.00
genus
22
1
10
194
4

0
201
1
1
2
species

 0.00


 0.00
strain
1
1
306263

2042961
1
3
species

 0.00


 0.00
species
3
1
195

species

 0.00
198
0
1
4
2

subspecies

 0.00
91353
1
4


 0.00
species
1
1
203

197
1
1
species

 0.00

species

 0.00
199
1
5

family

 0.00
6
2808963
1
31
16

genus

 0.00
0
2321187
1
1
2

1850254
1
1
species

 0.00


 0.00
genus
1
9
6
5
28196

1
1
2
2593671
0

 0.00
no rank

1
1
2961520

 0.00
species

1278212
0
1
2
2
species

 0.00


 0.00
strain
1
2
663365

1054033
1
1
species

 0.00

genus

 0.00
2321115
0
2
4
1

4
1
1462615

 0.00
species


 0.00
genus
8
1
3
2321111
1

1
5
28198

 0.00
species

2
1
28197

 0.00
species

genus

 0.00
2321114
0
2
1
3


 0.00
species
3
1
197482

21
1
47
0
28221

 0.00
class

0
213118
15
1
8
order

 0.00

0
1403365
1
1
2
no rank

 0.00

species

 0.00
2044940
1
1


 0.00
family
1
1
213121

6
1
11
213119
0

 0.00
family

genus

 0.00
0
45654
2
1
1

45656
1
1
species

 0.00

2
2
1
2299
0

 0.00
genus


 0.00
species
2
1
571177

2
1
3
0
2904687

 0.00
genus

species

 0.00
0
2296
2
2
1

2
1
177437

 0.00
strain

1
1
3
0
28222

 0.00
genus

0
28223
1
1
2
species

 0.00


 0.00
strain
1
1
651182

1
3
8
213115
0

 0.00
order

7
3
1
0
194924

 0.00
family


 0.00
genus
2
1
3
0
872

1
2
2
2593640
0

 0.00
no rank

1
2
2666132

 0.00
species


 0.00
genus
1
1
3
2794998
0


 0.00
species
2
1
1
880
0

strain

 0.00
526222
1
1

6
1
11
3
29

 0.00
order

1
1
5
0
80811

 0.00
suborder


 0.00
family
1
1
4
0
31

genus

 0.00
0
32
1
1
3

2
1
1
0
33

 0.00
species


 0.00
strain
1
1
1334629

suborder

 0.00
0
80812
5
2
1

1
49
2
1
4
family

 0.00

39643
0
3
1
1
genus

 0.00

2
1
1
56
0

 0.00
species


 0.00
strain
1
1
448385

12
4
1
0
69541

 0.00
order

11
1
4
213422
0

 0.00
family

2883512
0
3
1
1
genus

 0.00


 0.00
species
1
1
2
0
225194

1
1
404380

 0.00
strain


 0.00
genus
4
2
1
0
28231


 0.00
species
1
1
1340425

2
1
1
0
2627627

 0.00
no rank

1
1
1277350

 0.00
species

genus

 0.00
2910589
0
1
1
3


 0.00
species
2
1
1
351604
0

351605
1
1
strain

 0.00

49
28216
213
740
1
class

 0.02

140
1
614
100
80840

 0.02
order

8
1
15
506
1

 0.00
family

1
1
2
517
0

 0.00
genus


 0.00
species
1
1
1416803

1
1
507

 0.00
genus


 0.00
genus
2
7
1
1
222


 0.00
species
1
6
1353891

5
1
2
90243
1

 0.00
genus

species

 0.00
90245
4
1

2
2
1
224471
1

 0.00
no rank

212743
1
1
genus

 0.00


 0.00
family
89
1
25
119060
1

2
1
37
0
157932

 0.00
genus


 0.00
species
37
1
29443

2
1
2
240411
0

 0.00
genus

species

 0.00
356302
2
1


 0.00
genus
1
13
2
48736
1

species

 0.00
190721
12
1

0
47670
2
1
10
genus

 0.00

47671
10
1
species

 0.00


 0.00
genus
2
7
1
93217
0


 0.00
species
7
1
93219

genus

 0.00
44013
6
14
1
6

576610
1
1
species

 0.00


 0.00
no rank
4
1
7
1
2640945

1855619
3
1
species

 0.00

2576932
1
2
species

 0.00

1
1
1758390

 0.00
species

1
1
2
0
1827195

 0.00
genus

1
1
2646786

 0.00
no rank


 0.00
genus
1
3
5
1822464
0

species

 0.00
1761016
1
1


 0.00
species
1
1
134537

species

 0.00
36873
0
2
1
1

266265
1
1
strain

 0.00

genus

 0.00
106589
1
1

6
2
1
2975441
0

 0.00
family

3
1
1
65047
0

 0.00
genus


 0.00
no rank
1
1
2
0
2648776

1658665
1
1
species

 0.00

2
1
1
34102
0

 0.00
genus

639200
1
1
species

 0.00


 0.00
family
3
1
1
995019
0

genus

 0.00
40544
0
2
1
1

species

 0.00
40545
1
1

166
1
35
75682
35

 0.00
family

401469
4
5
1
15
genus

 0.00


 0.00
species
1
4
401471

2495591
4
1
species

 0.00


 0.00
no rank
1
3
2
2630295
1

species

 0.00
2058625
1
2


 0.00
genus
1
65
12
29580
36

2610881
5
17
1
6
no rank

 0.00

2
1
1537274

 0.00
species

1938606
4
1
species

 0.00

species

 0.00
2497863
1
1

2
1
375286

 0.00
species

species

 0.00
1644131
3
1

2
1
6
55508
3

 0.00
species

strain

 0.00
1349767
1
3


 0.00
species
1
4
2590869

species

 0.00
29581
1
1

1
1
368607

 0.00
species

no rank

 0.00
11
119059
3
1
14

1
1
2955019

 0.00
species


 0.00
species
2
1
2955020

3
1
4
202907
2

 0.00
genus

1
1
279058

 0.00
species

1
1
158899

 0.00
species

33
1
11
5
2895353

 0.00
no rank

75654
0
2
1
1
genus

 0.00

1
1
2728021

 0.00
species


 0.00
genus
1
4
1522432


 0.00
genus
1
23
7
13
149698


 0.00
species
3
1
2045208

no rank

 0.00
2
2609279
5
7
1

species

 0.00
1337838
1
1

2852099
2
1
species

 0.00

1
1
2738142

 0.00
species

species

 0.00
2769491
1
1

60
1
239
84
80864

 0.01
family

genus

 0.00
47420
13
4
16
1


 0.00
no rank
1
3
3
2610897
1

1
1
2565558

 0.00
species


 0.00
species
1
1
2184519

83494
0
5
1
3
no rank

 0.00

2952996
1
1
species

 0.00

2893879
1
4
species

 0.00

283
3
9
1
16
genus

 0.00

363952
1
1
species

 0.00

species

 0.00
285
1
1

2
1
5
32013
0

 0.00
species


 0.00
strain
1
5
1219032

379895
2
1
species

 0.00

1
3
225991

 0.00
species

1
1
2
0
2638500

 0.00
no rank

2918299
1
1
species

 0.00

genus

 0.00
34072
33
1
62
8


 0.00
no rank
1
4
2
2
663243

1
2
2774875

 0.00
species


 0.00
species
1
17
3
13
34073

strain

 0.00
595537
2
1

1246301
2
1
strain

 0.00


 0.00
species
1
8
2
436515
7


 0.00
strain
1
1
1321609

4
13
1
80865
8

 0.00
genus

0
2613839
1
1
2
no rank

 0.00

species

 0.00
742013
1
1

species

 0.00
80866
4
1

12916
7
8
1
24
genus

 0.00

15
1
6
2684926
1

 0.00
no rank

1
1
1858609

 0.00
species

1842533
1
8
species

 0.00

2
1
358220

 0.00
species

species

 0.00
2478662
1
1


 0.00
species
2
1
2518343

species

 0.00
553814
1
2


 0.00
genus
6
1
6
52972
2

2
1
2
1
2638319

 0.00
no rank

1
1
2895563

 0.00
species

37448
1
1
species

 0.00

216465
0
1
1
2
species

 0.00

strain

 0.00
365044
1
1


 0.00
genus
4
1
5
0
219181

species

 0.00
2816950
1
1

2109914
1
2
species

 0.00


 0.00
no rank
2
1
1
0
2645081

1658672
1
1
species

 0.00


 0.00
genus
1
1
3
0
665874


 0.00
no rank
2
1
1
0
2626134

1678129
1
1
species

 0.00

genus

 0.00
28065
0
3
1
5

no rank

 0.00
2627954
0
3
2
1


 0.00
species
1
1
2822760

2752316
1
1
species

 0.00

species

 0.00
1842727
1
1

genus

 0.00
0
352450
2
2
1

2109915
1
2
species

 0.00


 0.00
genus
2
1
3
238749
0


 0.00
species
1
3
1288495


 0.00
order
18
17
1
0
206389

family

 0.00
2008795
1
3
2
1

1
1
2
0
88875

 0.00
genus


 0.00
species
1
1
76259

1
5
5
2008794
0

 0.00
family

1
1
33057

 0.00
genus

genus

 0.00
12960
0
3
1
4


 0.00
no rank
1
4
2
2629479
0

2027405
4
1
species

 0.00

family

 0.00
75787
0
6
2
1

0
90628
1
1
2
no rank

 0.00

1
1
151985

 0.00
species


 0.00
genus
3
1
1
551759
0


 0.00
species
1
1
2
0
551760


 0.00
strain
1
1
76114


 0.00
family
3
1
8
0
2808923

2808942
0
8
1
2
genus

 0.00

1751046
8
1
species

 0.00

order

 0.00
0
206351
29
47
1

8
2
1
1499392
0

 0.00
family

no rank

 0.00
90153
0
4
1
1

genus

 0.00
535
0
3
1
1

1
1
2
0
2641838

 0.00
no rank

species

 0.00
2059672
1
1

187
0
1
1
3
genus

 0.00

0
2620219
2
1
1
no rank

 0.00

1938604
1
1
species

 0.00

20
45
1
481
2

 0.00
family

1
1
2
2593687
0

 0.00
genus

species

 0.00
2067065
1
1

2
1
1
212742
0

 0.00
genus

1056807
1
1
species

 0.00


 0.00
genus
1
1
538


 0.00
genus
2
1
3
0
32257


 0.00
species
1
1
502

species

 0.00
505
1
1

482
20
11
38
1
genus

 0.00

1
2
490

 0.00
species

2
1
2
0
496

 0.00
species

strain

 0.00
997348
1
2

5
1
28449

 0.00
species

0
2623750
1
1
3
no rank

 0.00

species

 0.00
641148
0
1
1
2

1
1
641149

 0.00
strain

4
495
2
5
1
species

 0.00

subspecies

 0.00
88719
1
1

488
3
1
species

 0.00


 0.00
no rank
3
1
3
33809
0


 0.00
species
1
2
1891241

species

 0.00
1690485
1
1

22
10
1
1
32003

 0.00
order


 0.00
family
3
1
2
32011
1

0
1679002
1
1
2
genus

 0.00

1581680
1
1
species

 0.00

family

 0.00
2008793
0
2
1
6

genus

 0.00
0
311181
2
1
1

1
1
311182

 0.00
species

genus

 0.00
1054211
0
1
1
3


 0.00
species
2
1
1
0
748811

1223802
1
1
strain

 0.00

family

 0.00
2803844
0
1
1
3

0
2803845
1
1
2
genus

 0.00

species

 0.00
2732067
1
1

family

 0.00
90627
0
2
1
5

1
2
4
0
453161

 0.00
genus

no rank

 0.00
2647919
0
2
1
1

species

 0.00
2559597
1
1


 0.00
species
1
1
453162

1
1
2008790

 0.00
family


 0.00
family
1
1
3
0
2772226

1
1
2
0
2772198

 0.00
genus

1
1
2715678

 0.00
species

48479
8
17
41
1
no rank

 0.00

1393463
2
1
species

 0.00

species

 0.00
1393492
1
1

1256581
1
1
species

 0.00


 0.00
species
2
1
1393505

species

 0.00
581112
1
1

2
1
1393423

 0.00
species

1204709
1
2
species

 0.00


 0.00
species
1
1
1489284


 0.00
species
1
1
1489290

1
1
1132276

 0.00
species


 0.00
species
1
1
1393499

1393529
1
1
species

 0.00


 0.00
species
1
1
1637487

1
1
1393584

 0.00
species


 0.00
species
1
13
77133


 0.00
species
2
1
581114


 0.00
no rank
2
1
2
49928
0


 0.00
species
1
2
2829681

1159
6078
1
1783272
80

 0.15
clade

67819
0
1
1
3
phylum

 0.00

1
1
2
1042316
0

 0.00
no rank

species

 0.00
2033014
1
1

1
4
12
0
1297

 0.00
phylum

1
4
11
0
188787

 0.00
class


 0.00
order
1
3
7
68933
0

1
3
6
188786
0

 0.00
family

1
3
5
270
1

 0.00
genus

1
1
2
0
37636

 0.00
species

743525
1
1
strain

 0.00


 0.00
species
2
1
1
274
0

798128
1
1
strain

 0.00

1
1
3
0
118964

 0.00
order

2
1
1
0
183710

 0.00
family


 0.00
genus
1
1
1298

544448
0
45
28
1
phylum

 0.00

0
31969
1
20
38
class

 0.00


 0.00
order
9
1
18
0
2085

1665628
0
1
1
2
no rank

 0.00

1609546
1
1
species

 0.00


 0.00
family
15
8
1
2092
0

7
1
4
2093
0

 0.00
genus

species

 0.00
754516
1
1

2
1
3
2683645
0

 0.00
no rank


 0.00
species
1
1
640330


 0.00
species
1
1
2801918

2
1
1
0
65123

 0.00
species


 0.00
strain
1
1
1197325


 0.00
genus
3
1
2
0
2767358

1
1
28903

 0.00
species

1
1
29556

 0.00
species

4
2
1
0
2923352

 0.00
genus

29562
1
1
species

 0.00

species

 0.00
0
2118
1
1
2


 0.00
strain
1
1
267748

order

 0.00
0
186328
11
1
7


 0.00
family
5
1
3
2131
0

2132
0
3
1
4
genus

 0.00


 0.00
species
1
1
2133

species

 0.00
216931
1
1


 0.00
species
1
1
1963034

0
33925
1
4
5
family

 0.00

genus

 0.00
46239
0
1
4
4

species

 0.00
138853
0
1
2
2

1
2
81460

 0.00
strain


 0.00
species
2
1
216427


 0.00
order
8
4
1
0
186329

0
2146
7
4
1
family

 0.00

33926
0
2
1
3
genus

 0.00

species group

 0.00
1
85625
2
1
2

1
1
135727

 0.00
species

0
2147
2
1
3
genus

 0.00

1
1
2148

 0.00
species


 0.00
species
1
1
35623

658142
0
2
1
1
no rank

 0.00

1
1
658143

 0.00
species

2
1
4
0
713063

 0.00
no rank

species

 0.00
2231116
4
1

order
2790996
2
3

3
2
2895623
family

2995234
genus
3
2

2104
2
1
species

 0.00

1
1
92758

 0.00
species


 0.03
phylum
1
1122
467
1239
22


 0.00
class
16
1
17
526524
0

order

 0.00
1
526525
17
1
15

2810280
family
1
2

1
2
3025755
genus


 0.00
species
2
1
1
29348
0

428126
1
1
strain

 0.00

15
1
12
128827
1

 0.00
family

2
1
2
0
2749267

 0.00
genus


 0.00
species
2
1
2583452


 0.00
genus
2
1
1
1729679
0


 0.00
species
1
1
1702221

0
544447
1
1
2
no rank

 0.00

2109692
1
1
species

 0.00

1647
2
5
1
10
genus

 0.00

1648
3
1
species

 0.00

2624170
0
1
5
3
no rank

 0.00

2714928
1
1
species

 0.00

species

 0.00
2714929
1
4

6
186801
1
329
169
class

 0.01


 0.00
order
4
8
1
0
53433

387655
0
3
1
8
no rank

 0.00

2
1
8
0
1769008

 0.00
genus

1323375
8
1
species

 0.00

8
1
4
68295
0

 0.00
order

0
227387
2
1
2
family

 0.00

227388
2
1
genus

 0.00

family

 0.00
0
543371
1
2
5

1
1
44000

 0.00
genus


 0.00
genus
3
1
1
0
28895


 0.00
no rank
1
1
2
0
2622527

species

 0.00
1550240
1
1


 0.00
no rank
3
1
2
0
218638


 0.00
species
1
3
244328

order

 0.01
45
186802
154
1
308

family

 0.00
2937909
0
1
1
4

51196
0
1
1
3
genus

 0.00

2
1
1
51197
0

 0.00
species


 0.00
strain
1
1
645991

12
4
1
0
538999

 0.00
no rank

genus

 0.00
2717089
0
1
1
2

species

 0.00
2086584
1
1

543314
0
1
2
5
family

 0.00

1
1
2
0
143393

 0.00
species

1
1
888727

 0.00
strain

1
1
2
86331
0

 0.00
genus

114527
1
1
species

 0.00

family

 0.00
539000
0
4
1
1

genus

 0.00
73918
0
3
1
1


 0.00
no rank
2
1
1
2619059
0

species

 0.00
2293838
1
1

68298
0
1
1
3
family

 0.00


 0.00
genus
2
1
1
2293313
0

1
1
2293317

 0.00
species

no rank

 0.00
30
39779
74
1
3

species

 0.00
2806438
1
2

1898207
1
42
species

 0.00

1
63
31
0
31979

 0.00
family


 0.00
genus
2
1
1
390805
0

1424294
1
1
species

 0.00

1485
7
1
57
23
genus

 0.00

species

 0.00
1519
1
6


 0.00
species
1
3
1492

1216932
1
3
species

 0.00


 0.00
species
1
1
1561

1
2
1513

 0.00
species

1520
1
5
species

 0.00

1
1
1504

 0.00
species

species

 0.00
704125
1
1


 0.00
species
1
1
2
0
217159

536227
1
1
strain

 0.00

species

 0.00
36745
1
1

1
1
1488

 0.00
species


 0.00
no rank
1
4
5
0
2614128


 0.00
species
1
1
1970093


 0.00
species
1
1
97138

755731
1
1
species

 0.00

species

 0.00
1155388
1
1

1
2
182773

 0.00
species

2068654
1
1
species

 0.00

species

 0.00
1491
1
6

94869
1
2
species

 0.00

10
1
1502

 0.00
species

1
4
3
0
189971

 0.00
no rank

1
1
2830659

 0.00
species

species

 0.00
1898204
1
3


 0.00
genus
2
1
1
0
1649459

species

 0.00
154046
1
1

43
1
23
216572
11

 0.00
family


 0.00
no rank
1
6
2
473772
0

species

 0.00
2485925
1
6

2
3
1
0
35829

 0.00
genus


 0.00
species
3
1
1515


 0.00
genus
2
1
1
0
946234

292800
1
1
species

 0.00

genus

 0.00
0
1508657
2
1
2


 0.00
species
2
1
2488810

9
1
13
1
1263

 0.00
genus

0
1264
1
3
2
species

 0.00

1
3
697329

 0.00
strain


 0.00
species
2
1
1160721

species

 0.00
1265
1
1

2564099
2
1
species

 0.00

no rank

 0.00
0
2608920
1
4
3

1
3
2831966

 0.00
species

species

 0.00
657323
1
1

2892396
0
1
1
2
genus

 0.00

species

 0.00
2714353
1
1

genus

 0.00
4
216851
3
6
1

species

 0.00
1
853
2
2
1

657322
1
1
strain

 0.00

6
1
3
186806
0

 0.00
family

genus

 0.00
1730
0
2
1
3

no rank

 0.00
2624479
0
1
2
2

2841037
2
1
species

 0.00

1
1
2
33951
0

 0.00
genus

52694
1
1
species

 0.00

family

 0.00
2603322
0
4
1
5

2
3
1
0
1348611

 0.00
genus

1
3
1185412

 0.00
species

1
1
2
2603323
0

 0.00
genus

2173034
1
1
species

 0.00


 0.00
family
11
1
9
0
186804

genus

 0.00
1870884
0
4
1
3

no rank

 0.00
2635829
1
1

species

 0.00
1496
1
3

2
3
1
0
1849828

 0.00
genus

species

 0.00
1505
3
1


 0.00
genus
1
1
2
2743582
0

89152
1
1
species

 0.00

3
1
1
0
1501226

 0.00
genus

0
2626894
1
1
2
no rank

 0.00

2724150
1
1
species

 0.00

family

 0.00
186803
14
61
1
55

2
1
3
2840493
0

 0.00
no rank

2
2
1
39491
0

 0.00
species

2
1
657317

 0.00
strain


 0.00
genus
3
1
4
2316020
0

species

 0.00
33038
1
1

46228
0
2
2
1
species

 0.00

471875
1
2
strain

 0.00

genus

 0.00
1506577
0
1
1
2

1
1
36835

 0.00
species


 0.00
genus
2
2
1
2719313
0

208479
1
2
species

 0.00

1
1
2
0
1843210

 0.00
genus

1727145
1
1
species

 0.00


 0.00
genus
18
1
6
830
2

185008
3
1
species

 0.00

2
11
1
10
831

 0.00
species


 0.00
strain
1
1
657324


 0.00
species
2
1
2
43305
0

2
1
515622

 0.00
strain

1769710
0
1
1
2
genus

 0.00

1653434
1
1
species

 0.00

genus

 0.00
207244
0
2
1
1

1
1
649756

 0.00
species

1
1
2
186928
0

 0.00
no rank


 0.00
species
1
1
712982

genus

 0.00
2944152
0
2
1
1

2763670
1
1
species

 0.00

genus

 0.00
0
572511
1
6
6


 0.00
species
2
2
1
1
40520


 0.00
strain
1
1
657314

418240
1
1
2
2
species

 0.00


 0.00
strain
1
1
1121115

1796616
1
2
species

 0.00

0
2944193
1
1
2
genus

 0.00

2763672
1
1
species

 0.00

3
1
1
0
2005359

 0.00
genus

no rank

 0.00
0
2635385
2
1
1

1
1
2823316

 0.00
species


 0.00
genus
1
1
2
0
189330

species

 0.00
88431
1
1


 0.00
genus
2
1
2
46205
0

185007
1
2
species

 0.00


 0.00
genus
2
1
5
2719231
0


 0.00
species
1
1
2
84030
0


 0.00
strain
1
1
717608

species

 0.00
0
29370
1
1
2


 0.00
strain
1
1
1297793

2
1
1
0
1164882

 0.00
genus

1
1
617123

 0.00
species


 0.00
genus
5
2
1
0
33042

0
2684943
1
1
2
no rank

 0.00


 0.00
species
1
1
751585

1
1
2
116085
0

 0.00
species

717962
1
1
strain

 0.00

4
91061
239
1
718
class

 0.02


 0.01
order
107
450
1
3
186826

1
186827
7
15
1
family

 0.00

genus

 0.00
0
46123
12
1
2

46125
12
1
species

 0.00

no rank

 0.00
881649
0
1
1
2

species

 0.00
2969821
1
1

2
1
1
0
89092

 0.00
genus

1
1
89093

 0.00
species

33958
5
1
47
38
family

 0.00

2767887
0
4
5
1
genus

 0.00


 0.00
species
1
4
1624

0
1623
2
1
1
species

 0.00


 0.00
strain
1
1
1069534


 0.00
genus
3
1
2
0
2742598

species

 0.00
227942
1
1

species

 0.00
1598
1
1

1
2
2
1
46255

 0.00
genus

46256
1
1
species

 0.00


 0.00
genus
1
3
4
0
2767879

2
2
1
0
1602

 0.00
species

strain

 0.00
1423720
1
2

species

 0.00
392416
1
1

1
2
2
0
2759736

 0.00
genus

species

 0.00
1597
1
2

10
18
1
2
1578

 0.00
genus

species

 0.00
1584
1
1

species

 0.00
2012495
1
1

4
1
33959

 0.00
species

1
1
2269374

 0.00
species

147802
3
1
species

 0.00

303541
2
1
species

 0.00

1
1
47770

 0.00
species

1
1
151781

 0.00
species


 0.00
species
2
1
1604

2
1
1
0
2767892

 0.00
genus

1
1
240427

 0.00
species


 0.00
genus
1
4
3
1253
0

2
4
1
1670797
0

 0.00
species group

1254
4
1
species

 0.00


 0.00
genus
1
3
3
1243
0

species

 0.00
33964
1
1


 0.00
species
2
1
1245

2767888
1
1
genus

 0.00

1
1
3
0
2767881

 0.00
genus

no rank

 0.00
2767908
0
2
1
1

2940297
1
1
species

 0.00

0
1300
39
1
351
family

 0.01

genus

 0.00
3
1357
5
1
3

1358
1
1
species

 0.00


 0.00
species
1
1
1366

1301
81
346
1
35
genus

 0.01


 0.00
species
2
21
1
1305
19

strain

 0.00
388919
1
2

1
2
1501662

 0.00
species

1156431
1
2
species

 0.00

species group

 0.00
119603
0
2
1
1


 0.00
species
1
1
1336


 0.00
species
2
17
1
16
1303

1458253
1
1
subspecies

 0.00

species

 0.00
113107
1
2

1343
1
3
species

 0.00


 0.00
species
1
7
1302

species

 0.00
0
1318
3
2
1


 0.00
strain
1
1
1114965

1
1
760570

 0.00
strain

1335
1
2
species

 0.00


 0.00
species
2
1
1433513

1304
9
1
species

 0.00

species group

 0.00
0
671232
1
1
2

species

 0.00
1328
1
1

1308
128
1
species

 0.00

1307
1
1
species

 0.00

7
2608887
1
32
8
no rank

 0.00

1
11
2598453

 0.00
species

4
1
712633

 0.00
species

1
1
712624

 0.00
species


 0.00
species
2
1
2972784

2759692
1
3
species

 0.00

species

 0.00
2710759
1
1


 0.00
species
3
1
712623

68892
1
1
species

 0.00

29
1
2
28037
27

 0.00
species


 0.00
strain
1
2
365659

1
1
45634

 0.00
species

1313
2
1
species

 0.00

family

 0.00
186828
0
13
23
1

0
1470540
2
2
1
genus

 0.00

1
2
2496265

 0.00
species

genus

 0.00
0
117563
3
13
1


 0.00
species
1
1
137732

species

 0.00
46124
12
1

2
3
1
0
29393

 0.00
genus

1
3
29394

 0.00
species

0
2747
5
5
1
genus

 0.00


 0.00
no rank
3
4
1
0
257487

1564681
1
1
species

 0.00

3
1
208596

 0.00
species

2748
1
1
species

 0.00

family

 0.00
81852
0
11
1
9

genus

 0.00
2737
0
3
1
1


 0.00
no rank
1
1
2
2648499
0

2920380
1
1
species

 0.00

3
1350
10
1
5
genus

 0.00

1
1
71452

 0.00
species

2
1
417368

 0.00
species

species

 0.00
1352
1
1

species

 0.00
44008
1
3

no rank

 0.00
0
1526609
2
1
1


 0.00
species
1
1
1903720


 0.01
order
1
263
129
4
1385


 0.00
family
3
1
1
0
186823

0
41371
2
1
1
no rank

 0.00


 0.00
species
1
1
2901836

186817
3
65
75
1
family

 0.00

genus

 0.00
0
200903
1
1
2

1
1
2213194

 0.00
species


 0.00
genus
2
1
1
2944627
0

1
1
2592382

 0.00
species

genus

 0.00
1055323
0
1
1
2


 0.00
species
1
1
33936


 0.00
genus
2
4
1
2675229
0

4
1
450367

 0.00
species

genus

 0.00
0
2800373
1
1
2

species

 0.00
86664
1
1


 0.00
genus
2
1
2
0
1276290

1
2
35841

 0.00
species

genus

 0.00
1906945
0
3
1
2

no rank

 0.00
2637039
0
2
1
2

2
1
2916802

 0.00
species


 0.00
genus
1
1
2
0
2675232


 0.00
species
1
1
220684

12
1
7
400634
0

 0.00
genus

1
1
28031

 0.00
species

no rank

 0.00
1
2636778
4
1
6


 0.00
species
1
1
2072025

species

 0.00
2936682
1
1

species

 0.00
2070463
1
3

species

 0.00
33935
1
5

2675231
0
3
1
1
genus

 0.00


 0.00
no rank
1
1
2
2675270
0


 0.00
species
1
1
2880221

genus

 0.00
459532
1
1

2675233
0
5
1
4
genus

 0.00


 0.00
no rank
2
2
1
0
2675274


 0.00
species
2
1
2745198

2567941
1
1
species

 0.00

species

 0.00
152268
1
1

genus

 0.00
0
2817139
1
1
2

1398
1
1
species

 0.00

0
2675234
3
1
1
genus

 0.00

0
2675276
2
1
1
no rank

 0.00

species

 0.00
2924032
1
1

13
27
1
1386
6

 0.00
genus

species group

 0.00
4
86661
5
1
15

species

 0.00
2026191
1
2

1
1
1428

 0.00
species


 0.00
species
1
7
1392


 0.00
species
1
1
1396

species group

 0.00
653685
1
1
3
3


 0.00
species
1
1
96241

species

 0.00
1452
1
1

no rank

 0.00
0
185979
3
1
4

2666127
1
1
species

 0.00

1
1
2011012

 0.00
species

species

 0.00
2884833
1
1

genus

 0.00
1329200
0
2
1
1

255247
1
1
species

 0.00

129337
1
1
3
3
genus

 0.00

species

 0.00
1921421
1
1

species group

 0.00
1505648
1
1


 0.00
no rank
1
1
2
197483
0

species

 0.00
1889774
1
1


 0.00
genus
4
5
1
3
84406

2
1
3
0
2620237

 0.00
no rank

1
1
2700081

 0.00
species

1
1
403957

 0.00
species

2
1
2
2837504
0

 0.00
genus

species

 0.00
1476025
1
2

1
6
3
0
186824

 0.00
family

2023
0
2
6
1
genus

 0.00


 0.00
species
6
1
2026

no rank

 0.00
539002
0
8
7
1

0
539742
1
2
2
no rank

 0.00


 0.00
genus
2
1
33986

no rank

 0.00
0
539738
5
1
5

genus

 0.00
1378
0
5
1
4

species

 0.00
84135
1
2

29391
1
1
species

 0.00

1379
2
1
species

 0.00

186818
0
13
1
11
family

 0.00

genus

 0.00
651660
0
2
1
4

species

 0.00
2320858
1
4


 0.00
genus
4
1
2
0
1372

species

 0.00
161360
0
1
1
2

1
1
1185653

 0.00
strain

1
1
2233542

 0.00
species

4
7
1
4
1569

 0.00
genus

298596
1
1
species

 0.00

0
2647733
2
1
2
no rank

 0.00

2
1
2762563

 0.00
species


 0.00
family
14
11
1
0
186822

55080
0
2
1
1
genus

 0.00

1
1
1465

 0.00
species

genus

 0.00
44249
2
1
9
8

185978
0
1
4
5
no rank

 0.00

species

 0.00
2660554
1
1

1
1
1536775

 0.00
species

2758563
1
1
species

 0.00


 0.00
species
1
1
1536769


 0.00
species
1
2
1406

species

 0.00
1401
1
1


 0.00
genus
1
1
3
329857
0


 0.00
no rank
2
1
1
0
2636738

1
1
1619153

 0.00
species


 0.00
family
2
1
1
0
186820

genus

 0.00
1637
1
1


 0.00
family
22
145
1
1
90964


 0.00
genus
2
1
2
0
69965

1903056
1
2
species

 0.00

genus

 0.00
2803850
0
1
2
2

1296
1
2
species

 0.00

140
1
17
13
1279

 0.00
genus


 0.00
species
1
1
170573

species

 0.00
28035
1
2

1292
15
1
species

 0.00

1290
11
2
1
12
species

 0.00

1
1
145391

 0.00
subspecies

species

 0.00
214473
1
3

46
1282
2
1
47
species

 0.00

1449752
1
1
strain

 0.00

45972
14
1
species

 0.00

20
1
29388

 0.00
species

species

 0.00
2750012
1
1


 0.00
species
1
2
29382


 0.00
species
1
1
33028

1
1
1288

 0.00
species


 0.00
species
1
5
61015

species

 0.00
1283
3
1

0
909932
30
31
1
class

 0.00


 0.00
order
1
6
15
0
909929


 0.00
family
1
2
6
1843490
0

0
365348
1
1
3
genus

 0.00

0
2629460
1
1
2
no rank

 0.00

484770
1
1
species

 0.00


 0.00
genus
1
1
2
0
2093783

1
1
1930071

 0.00
species


 0.00
family
1
4
8
0
1843491

970
0
1
3
5
genus

 0.00


 0.00
species
1
2
3
0
971

2
1
2
0
114197

 0.00
subspecies

strain

 0.00
927704
1
2

2637378
1
1
no rank

 0.00

158846
0
1
1
2
genus

 0.00


 0.00
species
1
1
437897

1
2
4
0
1843488

 0.00
order

909930
0
1
2
3
family

 0.00


 0.00
genus
2
1
2
33024
0

33025
2
1
species

 0.00


 0.00
order
10
23
1
1843489
0

31977
0
9
23
1
family

 0.00

genus

 0.00
29465
3
6
22
1

species

 0.00
39778
1
1

species

 0.00
29466
1
16

0
2630086
1
1
2
no rank

 0.00

1
1
2682455

 0.00
species

39777
1
1
species

 0.00

0
39948
2
1
1
genus

 0.00

1
1
487173

 0.00
species

class

 0.00
1737404
0
1
5
12

order

 0.00
0
1737405
4
1
8

family

 0.00
0
1570339
7
1
4

1
1
165779

 0.00
genus

2
1
2
0
150022

 0.00
genus

1
2
1260

 0.00
species

genus

 0.00
0
162289
3
1
1

2637196
0
1
1
2
no rank

 0.00

species

 0.00
2921087
1
1

1
1
3
1737407
0

 0.00
no rank


 0.00
genus
1
1
2
1582879
0

46507
1
1
species

 0.00

1
4617
483
20
201174

 0.11
phylum

84992
0
13
4
1
class

 0.00

order

 0.00
0
84993
1
4
12

4
1
1
84994
0

 0.00
family

0
53634
3
1
1
genus

 0.00

53635
0
1
1
2
species

 0.00


 0.00
strain
1
1
525909

3
1
7
633392
0

 0.00
family

genus

 0.00
1648491
0
1
2
3

2633173
0
1
2
2
no rank

 0.00

1
2
2849779

 0.00
species

467975
0
1
1
3
genus

 0.00


 0.00
no rank
1
1
2
0
2624035


 0.00
species
1
1
2722752

9
6
1
1497346
0

 0.00
class


 0.00
order
6
1
8
2
588673

family

 0.00
0
2976758
3
1
1

0
2976759
2
1
1
genus

 0.00


 0.00
species
1
1
2884022


 0.00
family
3
1
4
320583
0

0
191494
3
3
1
genus

 0.00

2
1
3
0
191495

 0.00
species

3
1
469383

 0.00
strain

class

 0.00
84998
4
12
1
11

order

 0.00
84999
0
5
1
3

1643824
2
4
1
3
family

 0.00

0
133925
1
1
3
genus

 0.00

0
2638792
2
1
1
no rank

 0.00


 0.00
species
1
1
712411

order

 0.00
1643822
0
1
4
6

2
1643826
5
4
1
family

 0.00

1
1
2
0
79603

 0.00
genus

species

 0.00
79604
1
1

0
84111
1
1
2
genus

 0.00


 0.00
species
1
1
84112

1760
590
443
4572
1
class

 0.11

29
1
1904
85009
7

 0.05
order

13
1857
1
2
31957

 0.05
family


 0.05
genus
7
1851
1
1912216
40

species

 0.00
33011
39
1

1
3
2559073

 0.00
species

species

 0.00
33010
1
5


 0.04
species
3
1
1764
1747
1757

1
1734925
1
7
2
subspecies

 0.00


 0.00
strain
1
6
1114967

genus

 0.00
0
72763
2
1
3

1
1
2161816

 0.00
species

1
1
1285901

 0.00
species


 0.00
genus
2
1
2
1
2801844

1
1
1547448

 0.00
species

family

 0.00
85015
3
1
40
15

116532
0
2
1
1
no rank

 0.00

species

 0.00
1871072
1
1

genus

 0.00
1839
16
9
29
1

1774216
1
1
species

 0.00

2712223
1
1
species

 0.00

3
2615069
4
1
6
no rank

 0.00

2763008
1
1
species

 0.00

species

 0.00
2592334
1
1

1
1
2714939

 0.00
species

1
1
2736757

 0.00
species

2894081
4
1
species

 0.00


 0.00
genus
1
2
2040

genus

 0.00
2044
0
2
1
5

5
1
2045

 0.00
species

19
34
1
4
85012

 0.00
order

5
1
9
0
83676

 0.00
family

genus

 0.00
2
2013
4
1
9


 0.00
species
1
1
2014


 0.00
no rank
2
1
6
2649073
1

1
5
2831968

 0.00
species

1
10
8
2004
1

 0.00
family


 0.00
genus
7
9
1
2
83681

93944
1
3
species

 0.00

404386
0
2
1
1
species

 0.00


 0.00
strain
1
1
1122611

species

 0.00
2219224
2
1

no rank

 0.00
0
2593643
1
1
2


 0.00
species
1
1
1909395

family

 0.00
4
2012
5
1
11

2019
1
1
genus

 0.00

1
6
3
1988
4

 0.00
genus

no rank

 0.00
2626254
1
2
1
2

species

 0.00
2742128
1
1


 0.00
order
5
1
1
1217098
0


 0.00
family
1
1
4
0
1217100

0
281472
1
1
3
genus

 0.00

2624397
0
2
1
1
no rank

 0.00

species

 0.00
1798224
1
1

order

 0.00
0
1643682
1
1
4

1
1
3
85030
0

 0.00
family

1
1
2
0
88138

 0.00
genus

1
1
477641

 0.00
species

0
85011
584
1
60
order

 0.01


 0.01
family
59
584
1
18
2062

1
1
2995704
genus

2126346
1
1
species

 0.00

genus

 0.01
1883
443
53
564
1

1
1
2496836

 0.00
species

1
1
2865673

 0.00
species

1
1
2763006

 0.00
species

species

 0.00
2014920
1
2

285558
1
1
species

 0.00


 0.00
species
1
1
1914


 0.00
species
2
1
1413221

species

 0.00
68214
2
1

2
1
1888

 0.00
species

1
1
28894

 0.00
species

1
1
173860

 0.00
species


 0.00
species
2
1
1
1930
0

680198
1
1
strain

 0.00

2
1
1
0
348043

 0.00
species

1
1
1214101

 0.00
strain

species

 0.00
2878388
2
1


 0.00
species
1
1
2653200

66871
1
2
species

 0.00

193462
1
1
species

 0.00

1
1
42881

 0.00
species

species

 0.00
1977088
1
1

1
1
1950

 0.00
species

22
1
81
36
2593676

 0.00
no rank

2175864
1
2
species

 0.00

2662065
2
1
species

 0.00


 0.00
species
1
1
1841249

species

 0.00
2083284
1
1

species

 0.00
2801029
2
1

species

 0.00
2730915
1
1


 0.00
species
1
1
2072505


 0.00
species
1
1
2720714


 0.00
species
1
9
2742136

1
1
1136432

 0.00
species


 0.00
species
1
1
2981134

2964669
1
1
species

 0.00

1
1
2879846

 0.00
species

1
1
2898276

 0.00
species

species

 0.00
444103
1
1

2750025
5
1
species

 0.00

1
2
2923272

 0.00
species

species

 0.00
2742137
2
1

2880933
1
4
species

 0.00


 0.00
species
2
1
2939414

1848900
1
4
species

 0.00

1
1
68270

 0.00
species


 0.00
species
1
1
319947

68249
2
1
species

 0.00


 0.00
species
1
1
1642299

1
1
67304

 0.00
species

1
1
67258

 0.00
species


 0.00
species
1
2
2293571


 0.00
species
5
1
1915

228398
0
1
genus

 0.00

genus

 0.00
2805681
0
1
1
3

no rank

 0.00
0
2846306
2
1
1


 0.00
species
1
1
2896848

0
85014
8
1
10
order

 0.00

0
85034
10
1
7
family

 0.00


 0.00
genus
3
1
1
283810
0


 0.00
species
2
1
1
0
283811

446470
1
1
strain

 0.00

9
1
3
58113
0

 0.00
genus


 0.00
no rank
9
1
2
2637084
0


 0.00
species
9
1
2867006


 0.01
order
1
560
102
85007
20

0
85026
16
1
8
family

 0.00

2053
5
7
16
1
genus

 0.00

2657482
1
1
no rank

 0.00

2055
1
1
species

 0.00

2054
1
1
species

 0.00

249058
1
6
species

 0.00

1
1
1004901

 0.00
species

species

 0.00
2665643
1
1

4
3
1
85029
0

 0.00
family


 0.00
genus
3
1
3
0
37914

no rank

 0.00
2617939
1
1

2
1
322509

 0.00
species


 0.00
family
24
69
1
17
1762


 0.00
genus
6
1
4
2
670516

1
3
404941

 0.00
species

1774
0
2
1
1
species

 0.00

subspecies

 0.00
2480908
1
1

1
16
10
6
1763

 0.00
genus

1
1
1768

 0.00
species

2642494
1
1
no rank

 0.00

2
1
1778

 0.00
species

species

 0.00
1801
1
1

species

 0.00
1389713
2
1


 0.00
species group
1
1
2
120793
0

species

 0.00
2775496
1
1


 0.00
species
1
1
152142


 0.00
species
1
1
577492

1866885
12
9
1
30
genus

 0.00

1
1
216929

 0.00
species

species

 0.00
758802
1
1

species

 0.00
258505
1
5

species

 0.00
36814
0
1
1
2

strain

 0.00
710685
1
1

species

 0.00
1795
1
1

8
1
319706

 0.00
species

98668
1
1
species

 0.00


 0.00
family
19
107
1
85025
1

genus

 0.00
1
1817
1
4
5

0
2637762
2
1
1
no rank

 0.00

1
1
1047172

 0.00
species

species

 0.00
135487
1
1


 0.00
species
1
1
1824


 0.00
genus
13
102
1
1827
78

15
1
5
2840174
3

 0.00
species group


 0.00
species
2
7
1
6
334542


 0.00
strain
1
1
1303681

4
1833
5
1
2
species

 0.00

strain

 0.00
234621
1
1


 0.00
species
1
1
37919

species

 0.00
1829
1
1


 0.00
no rank
4
5
1
192944
1

2054902
1
1
species

 0.00

1653478
2
1
species

 0.00


 0.00
species
1
1
1723645

2
1
1828

 0.00
species

family

 0.00
2805586
0
5
1
3

2
1
5
0
1847725

 0.00
genus

species

 0.00
1528099
5
1

1
338
40
1653
2

 0.01
family

43
1716
39
1
336
genus

 0.01

1
1
36808

 0.00
species

species

 0.00
39791
1
1

1
65
38304

 0.00
species

1705
1
1
species

 0.00

43770
1
1
species

 0.00

1
1
38302

 0.00
species

2594913
1
11
species

 0.00

43768
1
4
species

 0.00


 0.00
species
1
1
187491

species

 0.00
2768834
1
1


 0.00
species
2
1
1
0
1404244

1
1
1404245

 0.00
strain

species

 0.00
53374
1
5

1
34
1979527

 0.00
species

species

 0.00
43765
1
1

species

 0.00
156976
1
9


 0.00
species
6
1
2
0
1121358

6
1
558173

 0.00
strain

1
1
156978

 0.00
species

species

 0.00
37637
2
1


 0.00
species
1
1
191610

1
4
38290

 0.00
species

1
12
169292

 0.00
species

species

 0.00
575200
0
1
3
2

3
1
1224163

 0.00
strain


 0.00
species
1
11
401472


 0.00
species
1
1
43990

38284
1
1
species

 0.00

1
2
2763010

 0.00
species

2
5
1
38289
3

 0.00
species

strain

 0.00
306537
1
2


 0.00
species
1
98
161879

0
38288
2
1
1
species

 0.00

585529
1
1
strain

 0.00


 0.00
species
1
1
134034

1
1
161899

 0.00
species

1
5
43769

 0.00
species

species

 0.00
146827
1
1


 0.00
family
3
1
2
0
85028

genus

 0.00
2060
1
1
2
2

1
1
47312

 0.00
species


 0.00
order
4
1
3
2039638
0


 0.00
family
1
3
3
2162846
0


 0.00
genus
1
3
2
0
622681

species

 0.00
1884904
3
1

order

 0.00
85004
0
1
75
12


 0.00
family
11
1
75
20
31953


 0.00
genus
1
55
10
9
1678

species

 0.00
1681
1
1


 0.00
species
1
40
4
216816
31

2
1
1
0
1679

 0.00
subspecies

1
1
722911

 0.00
strain

8
1
1682

 0.00
subspecies


 0.00
species
2
1
1685

1
1
35760

 0.00
species

77635
1
1
species

 0.00

33905
1
1
species

 0.00


 0.01
order
124
1
422
27
85006

1
1
2
85021
0

 0.00
family

53457
1
1
genus

 0.00

family

 0.00
0
85018
3
1
5

genus

 0.00
0
2885697
2
1
1


 0.00
species
1
1
2716227

1184606
0
1
2
2
genus

 0.00

species

 0.00
100225
2
1

family

 0.01
1268
3
37
238
1

7
1
68
2
32207

 0.00
genus

species

 0.00
37923
1
6

5
2047
1
7
2
species

 0.00

762948
1
2
strain

 0.00

34
1
43675

 0.00
species


 0.00
species
16
1
169480

172042
1
3
species

 0.00

genus

 0.00
0
1742989
3
2
1

1
1
37930

 0.00
species

species

 0.00
162496
1
1

0
1742990
1
1
3
genus

 0.00


 0.00
no rank
2
1
1
0
2639426


 0.00
species
1
1
2853258


 0.00
genus
1
1
1742993

0
1160973
3
1
3
genus

 0.00

no rank

 0.00
2634694
0
2
3
1

species

 0.00
2170745
1
3

genus

 0.00
57493
2
82
1
7

species

 0.00
1049583
2
1


 0.00
species
1
16
71999

species

 0.00
1272
1
1

species

 0.00
72000
59
1

no rank

 0.00
2649579
1
1

1
1
388357

 0.00
species

1
1663
7
16
1
genus

 0.00


 0.00
species
1
1
2817475

3
235627
4
11
1
no rank

 0.00

species

 0.00
2565366
1
1

1
6
1477518

 0.00
species

species

 0.00
2020130
1
1

3
1
2895818

 0.00
species

genus

 0.00
1269
29
1
62
5

species

 0.00
566027
1
1


 0.00
species
26
1
1270

0
2620948
2
1
6
no rank

 0.00

6
1
2856555

 0.00
species

85019
0
1
3
3
family

 0.00


 0.00
genus
1
3
2
0
1696


 0.00
species
1
3
33889

145357
0
1
17
3
family

 0.00

genus

 0.00
0
57495
17
1
2


 0.00
species
1
17
1274

family

 0.00
85016
0
2
1
7


 0.00
genus
1
1
3
0
1707

2620175
0
1
1
2
no rank

 0.00

2654191
1
1
species

 0.00

0
1926259
1
1
3
genus

 0.00


 0.00
no rank
2
1
1
2649176
0

1
1
2654547

 0.00
species


 0.00
family
9
1
6
85020
0

6
43668
1
9
5
genus

 0.00

species

 0.00
2942909
1
1

1
1
47848

 0.00
species

1
1
2
43669
0

 0.00
species

1
1
446465

 0.00
strain

family

 0.00
85023
57
50
1
118


 0.00
genus
1
3
4
1
55968

1
1
2
0
2621730

 0.00
no rank

2875729
1
1
species

 0.00


 0.00
species
1
1
1935379

genus

 0.00
2034
1
6
1
9


 0.00
no rank
2
1
4
257496
0

species

 0.00
2795488
1
4


 0.00
species
1
1
33881


 0.00
species
2
3
1
2035
0

1
3
138532

 0.00
no rank

1
1
3
0
337004

 0.00
genus

0
2630066
1
1
2
no rank

 0.00

1
1
2963406

 0.00
species

1
3
4
190323
1

 0.00
genus


 0.00
no rank
2
1
1
0
2624265

2480625
1
1
species

 0.00

1
1
150123

 0.00
species

genus

 0.00
0
2680004
1
1
2

2592654
1
1
species

 0.00

235888
0
3
1
1
genus

 0.00

0
2632331
2
1
1
no rank

 0.00

1
1
2583374

 0.00
species

genus

 0.00
0
255204
2
1
1

species

 0.00
2820673
1
1

genus

 0.00
33886
2
1


 0.00
genus
11
25
1
33882
14


 0.00
species
1
1
300019

1
1
2614638

 0.00
species


 0.00
species
3
1
1072463


 0.00
species
1
1
156977

162426
1
1
species

 0.00

1
2
3
2609290
0

 0.00
no rank

2483401
1
1
species

 0.00


 0.00
species
1
1
2851648

1
1
582680

 0.00
species

2782167
1
1
species

 0.00

0
1573
1
3
2
genus

 0.00

3
1
1401995

 0.00
species

1
2
2
1
33877

 0.00
genus

659012
1
1
species

 0.00

3
2
1
46352
0

 0.00
genus

no rank

 0.00
2615065
1
1
2
2

1
1
2810513

 0.00
species


 0.00
genus
7
1
3
110934
0

2
1
7
2641148
0

 0.00
no rank


 0.00
species
7
1
2773266

3
1
1
0
881616

 0.00
genus

no rank

 0.00
2618217
0
1
1
2

1
1
2735133

 0.00
species

family

 0.00
0
145358
1
2
3

genus

 0.00
0
154116
2
1
2


 0.00
species
1
2
2589797

family

 0.00
2805590
0
2
1
7

genus

 0.00
265976
0
1
1
3

2
1
1
0
247333

 0.00
species

1123251
1
1
strain

 0.00

125287
0
1
1
3
genus

 0.00

2
1
1
0
2615080

 0.00
no rank

1
1
2934161

 0.00
species

14
1
44
85008
0

 0.00
order

18
28056
13
44
1
family

 0.00


 0.00
genus
1
1
2
0
35753

53360
1
1
species

 0.00

907364
1
1
genus

 0.00

2
1865
2
3
1
genus

 0.00

2626549
1
1
no rank

 0.00

1873
15
1
21
7
genus

 0.00


 0.00
no rank
1
1
2
2617518
0

species

 0.00
2583243
1
1


 0.00
species
1
1
356851

species

 0.00
47865
1
1

1
1
291594

 0.00
species

species

 0.00
709883
1
2

85013
0
1
4
8
order

 0.00


 0.00
family
5
3
1
74712
0

1854
0
3
1
4
genus

 0.00


 0.00
species
1
1
106370

2
1
2
1859
0

 0.00
species

strain

 0.00
326424
2
1


 0.00
no rank
1
1
2
1920255
0


 0.00
species
1
1
1882833

14
57
1
0
2037

 0.00
order


 0.00
family
13
1
57
0
2049

genus

 0.00
16
1654
10
1
40


 0.00
species
1
3
1655

1
2
544580

 0.00
species

no rank

 0.00
2609248
6
6
1
18


 0.00
species
1
2
712122


 0.00
species
1
2
712116

0
706438
1
4
2
species

 0.00

strain

 0.00
706439
4
1

1
4
2789425

 0.00
species

1
1
55565

 0.00
species

17
1
2
2529408
1

 0.00
genus

species

 0.00
1660
1
16

order

 0.00
1643683
0
5
1
1

family

 0.00
85032
0
1
1
4

genus

 0.00
0
28048
3
1
1


 0.00
species
2
1
1
0
28049

351607
1
1
strain

 0.00

0
85010
282
1
34
order

 0.01

family

 0.01
2070
78
282
1
33

genus

 0.00
1813
12
5
1
27

1
1
700274

 0.00
species

1
6
2618356

 0.00
no rank

species

 0.00
31958
1
1

species

 0.00
33910
1
7

0
39845
3
9
1
genus

 0.00

0
2642662
9
1
2
no rank

 0.00

species

 0.00
2268449
1
9

1
26
3
7
1847

 0.00
genus


 0.00
no rank
2
19
1
0
2619320

2865833
19
1
species

 0.00

674734
0
3
2
1
genus

 0.00

2
2
1
2636053
0

 0.00
no rank

species

 0.00
1653480
1
2

genus

 0.00
1835
2
3
1
4

species

 0.00
455344
1
1

1
1
60894

 0.00
species

1
13
4
3
165301

 0.00
genus


 0.00
species
5
1
1586287

0
2643253
2
5
1
no rank

 0.00


 0.00
species
1
5
2951806


 0.00
genus
4
22
1
2029
6

860235
1
8
species

 0.00

2
1
8
0
2637669

 0.00
no rank

species

 0.00
703222
1
8


 0.00
genus
1
1
40566

1
100
6
32
2071

 0.00
genus

6
1
2
0
103731

 0.00
species


 0.00
strain
6
1
1179773

0
2593673
2
60
1
no rank

 0.00

1
60
2781735

 0.00
species

species

 0.00
103733
2
1

4
1
5
84995
0

 0.00
class

order

 0.00
84996
0
1
4
4

84997
0
4
1
3
family

 0.00

2
4
1
1
42255

 0.00
genus

2653851
3
1
species

 0.00

200795
0
16
3
1
phylum

 0.00

32061
0
10
1
2
class

 0.00

32064
0
4
1
1
order

 0.00


 0.00
suborder
1
1
3
1508595
0

1508635
0
1
1
2
family

 0.00

1
1
120961

 0.00
genus

189772
0
1
1
5
order

 0.00

family

 0.00
189773
0
4
1
1

1
1
3
64
0

 0.00
genus


 0.00
species
1
1
2
0
65

316274
1
1
strain

 0.00


 0.00
class
1
1
5
292625
0

order

 0.00
292629
0
4
1
1

1
1
3
292628
0

 0.00
family


 0.00
no rank
2
1
1
1324991
0


 0.00
species
1
1
2024896


 0.01
clade
132
1
223
1798711
0


 0.01
phylum
1
218
128
1117
11

class
3028117
127
207

1890424
0
25
1
31
order

 0.00


 0.00
family
4
14
1
1890431
0

217161
0
1
14
3
genus

 0.00


 0.00
species
2
1
14
1173032
0

strain

 0.00
1173020
1
14


 0.00
family
1
1
4
1213
0

2892030
0
1
1
3
genus

 0.00


 0.00
species
2
1
1
2892031
0

2829509
1
1
strain

 0.00

4
1
1
0
1890429

 0.00
family

genus

 0.00
155977
0
1
1
3

species

 0.00
0
155978
2
1
1

strain

 0.00
329726
1
1

0
1890430
1
1
4
family

 0.00

283819
0
1
1
3
genus

 0.00

283820
0
1
1
2
species

 0.00


 0.00
strain
1
1
2824559

family

 0.00
0
2881426
9
1
4

3
1
2
167375
0

 0.00
genus

no rank

 0.00
0
2627006
2
2
1

2831057
1
2
species

 0.00


 0.00
genus
1
2
5
1218
0


 0.00
species
2
1
4
1219
0

1
1
93060

 0.00
strain

subspecies

 0.00
142479
0
2
1
1

1
1
59919

 0.00
strain


 0.00
family
4
1
5
0
1890426

4
1
4
1129
0

 0.00
genus

no rank

 0.00
2626047
0
3
4
1

2
1
1353136

 0.00
species


 0.00
species
1
2
1827144


 0.00
order
48
1
154
80
1161

no rank

 0.00
1219117
0
1
3
2


 0.00
species
3
1
1940762


 0.00
family
12
1
6
0
2661849


 0.00
genus
1
12
5
1186
0

2
1
3
1
2619626

 0.00
no rank

1170562
1
2
species

 0.00

2
9
1
938406
0

 0.00
species

1973478
9
1
strain

 0.00


 0.00
family
16
1
19
1162
0

genus

 0.00
0
264688
3
1
1

species

 0.00
1164
0
2
1
1


 0.00
strain
1
1
551115

5
1177
18
1
12
genus

 0.00

0
224012
1
1
2
species

 0.00

224013
1
1
strain

 0.00

species

 0.00
0
1178
2
2
1

2
1
449208

 0.00
strain


 0.00
no rank
8
1
5
2593658
2

1973475
1
1
species

 0.00

species

 0.00
1869241
1
1


 0.00
species
1
3
2914041


 0.00
species
1
1
2576903

species

 0.00
0
1306274
2
1
2

strain

 0.00
2038116
2
1


 0.00
family
4
1
1
0
1185


 0.00
genus
3
1
1
373984
0


 0.00
no rank
1
1
2
2676603
0

species

 0.00
373994
1
1

8
1
5
0
1182

 0.00
family

383614
1
3
genus

 0.00

1
5
3
1203
0

 0.00
genus

no rank

 0.00
0
2618749
1
5
2

5
1
2005464

 0.00
species

3
1
9
1892259
0

 0.00
family


 0.00
genus
3
1
1
0
752201

0
984207
2
1
1
species

 0.00

1
1
984208

 0.00
strain

genus

 0.00
0
159191
2
1
5

0
137816
2
1
1
species

 0.00

1
1
2761619

 0.00
strain

1
1
2
0
70799

 0.00
species

1
1
1914872

 0.00
strain

5
28
1
1892263
0

 0.00
family

genus

 0.00
1190
0
4
28
1

8
494603
1
28
3
no rank

 0.00

species

 0.00
2005456
1
1

species

 0.00
1191
19
1

32
17
1
1301283
0

 0.00
subclass


 0.00
order
5
1
13
1150
2

family

 0.00
0
1892255
1
1
4

1
1
3
0
241421

 0.00
genus


 0.00
species
1
1
2
0
241425

strain

 0.00
1173022
1
1


 0.00
family
4
1
1
0
1892254

1155738
0
3
1
1
genus

 0.00

species

 0.00
1155739
0
2
1
1

1454205
1
1
strain

 0.00

0
1892252
1
1
4
family

 0.00


 0.00
genus
1
1
3
0
44471

species

 0.00
0
119532
1
1
2


 0.00
strain
1
1
669365

18
1
12
1118
0

 0.00
order


 0.00
family
10
4
1
1890450
0


 0.00
no rank
4
1
1
0
92682


 0.00
genus
3
1
1
0
76023

no rank

 0.00
0
2632878
2
1
1

65093
1
1
species

 0.00


 0.00
genus
3
1
5
0
28070

2
1
2
0
2546356

 0.00
species

2
1
65393

 0.00
strain

2546359
0
1
1
2
species

 0.00

strain

 0.00
497965
1
1

family

 0.00
0
2815910
5
1
4

0
102234
3
1
5
genus

 0.00

species

 0.00
102235
0
2
1
5


 0.00
strain
5
1
292563

family

 0.00
0
1890449
3
3
1

genus

 0.00
1125
0
2
3
1

1126
1
3
species

 0.00

5
1
1
0
307595

 0.00
order

family

 0.00
1890422
0
1
1
4

33071
0
1
1
3
genus

 0.00


 0.00
species
1
1
2
0
33072


 0.00
strain
1
1
251221

10
1
11
2881377
0

 0.00
order

family

 0.00
0
1890438
7
1
9

2
1
3
47251
0

 0.00
genus

2
1
2
0
2650499

 0.00
no rank


 0.00
species
1
2
1080068


 0.00
genus
3
7
1
0
2917830


 0.00
species
2
7
1
2917832
0


 0.00
strain
1
7
2016068

family

 0.00
0
2881378
3
1
1

genus

 0.00
0
146785
1
1
2


 0.00
no rank
1
1
2622553


 0.00
phylum
5
1
3
1798710
0

2
5
1
1897007
0

 0.00
no rank

species

 0.00
2052166
1
5

clade

 0.02
1
1783270
364
785
1

8
19
1
0
65842

 0.00
phylum

204430
0
7
19
1
class

 0.00

6
1
19
218872
0

 0.00
order


 0.00
family
5
1
19
0
204431

genus

 0.00
0
832
1
19
4


 0.00
species
19
1
3
1
833


 0.00
subspecies
2
18
1
834
0

59374
18
1
strain

 0.00

1
1
4
0
456828

 0.00
phylum


 0.00
genus
3
1
1
456826
0

1
1
2
0
456827

 0.00
species

1
1
459349

 0.00
strain

1
68336
342
760
1
clade

 0.02


 0.00
phylum
9
3
1
0
1134404

0
1852932
1
1
2
no rank

 0.00

species

 0.00
2026749
1
1


 0.00
class
6
2
1
0
795747

0
795748
2
1
5
order

 0.00

family

 0.00
795749
0
2
1
4

genus

 0.00
0
795750
2
1
3


 0.00
no rank
2
1
2
2639715
0

species

 0.00
2651167
1
2

phylum

 0.00
1090
0
1
3
10

no rank

 0.00
0
44765
1
2
2


 0.00
species
2
1
2268192


 0.00
class
7
1
1
191410
0

order

 0.00
191411
0
1
1
6

family

 0.00
191412
0
1
1
5


 0.00
no rank
4
1
1
274493
0

0
1091
1
1
3
genus

 0.00

1
1
2
0
1096

 0.00
species

290317
1
1
strain

 0.00

phylum

 0.02
976
53
322
753
1

class

 0.00
0
117747
35
68
1

200666
0
68
1
34
order

 0.00

5
1
2
203473
0

 0.00
no rank

species

 0.00
2044944
5
1


 0.00
family
31
1
63
4
84566

84567
5
10
1
39
genus

 0.00


 0.00
species
1
2
2766984

species

 0.00
336820
2
1


 0.00
species
2
1
430522


 0.00
species
1
15
188932

no rank

 0.00
3
2628915
3
11
1


 0.00
species
1
1
2714940


 0.00
species
7
1
2856523


 0.00
species
1
1
2895286

species

 0.00
2789740
1
1

0
376469
4
1
4
genus

 0.00

no rank

 0.00
0
2632301
3
1
4


 0.00
species
1
1
2764720


 0.00
species
1
3
2592345

genus

 0.00
423349
3
1
6
5

2305508
1
1
species

 0.00

0
2617802
2
1
1
no rank

 0.00

1
1
2929803

 0.00
species


 0.00
species
1
1
652787

1
1
3
0
929509

 0.00
genus

species

 0.00
995
0
2
1
1

strain

 0.00
929556
1
1

9
1
8
0
28453

 0.00
genus


 0.00
species
4
1
371142

species

 0.00
258
1
1


 0.00
no rank
4
1
3
0
2609468

2713573
1
1
species

 0.00

1
1
1538644

 0.00
species

species

 0.00
2662364
1
1

species

 0.00
28454
1
1


 0.00
class
6
1
1
0
1937959

order

 0.00
0
1936988
5
1
1

0
1937961
4
1
1
family

 0.00

2349
0
1
1
3
genus

 0.00

0
2350
1
1
2
species

 0.00

1
1
760192

 0.00
strain

200643
3
217
1
71
class

 0.01

order

 0.01
171549
31
66
1
213

family

 0.00
2
815
1
35
13

genus

 0.00
12
816
9
23
1

1
0
2646097

 0.00
no rank


 0.00
species
1
1
674529

species

 0.00
246787
1
1

1
1
2715212

 0.00
species

1
2
28116

 0.00
species

1
2
371601

 0.00
species

species

 0.00
47678
2
1

818
2
1
species

 0.00


 0.00
genus
1
10
3
909656
1


 0.00
species
1
2
357276

species

 0.00
821
1
7

1
2005473
3
1
2
family

 0.00

2
1
1
2815786
0

 0.00
genus

2606626
1
1
species

 0.00

family

 0.00
2005525
0
2
1
5

0
375288
1
1
2
genus

 0.00

46503
1
1
species

 0.00

genus

 0.00
0
195950
1
1
2

1
1
712710

 0.00
species


 0.00
family
7
1
6
0
2005520

genus

 0.00
307628
0
4
5
1

species

 0.00
1642646
4
1

no rank

 0.00
0
2646834
1
1
2


 0.00
species
1
1
1978337

2811463
0
2
1
1
no rank

 0.00

1
1
2811465

 0.00
species

1
129
24
2
171552

 0.00
family

0
577309
1
1
3
genus

 0.00

1
1
2
0
454155

 0.00
species

1
1
762982

 0.00
strain


 0.00
genus
20
126
1
21
838

2
1
28131

 0.00
species

species

 0.00
2801997
1
2

0
52227
1
1
2
species

 0.00

strain

 0.00
908937
1
1


 0.00
no rank
48
1
7
2638335
16


 0.00
species
5
1
2913620


 0.00
species
4
1
2913616

2937774
1
2
species

 0.00

2691580
1
14
species

 0.00

species

 0.00
2913617
2
1

2913621
5
1
species

 0.00

28132
1
7
species

 0.00

165179
3
1
6
2
species

 0.00

strain

 0.00
537011
1
3


 0.00
species
2
1
25
839
19

strain

 0.00
264731
1
6

1
1
470565

 0.00
species

77095
9
2
1
13
species

 0.00

1
4
752555

 0.00
strain

171551
0
1
2
3
family

 0.00

genus

 0.00
836
1
2
1
2


 0.00
species
1
1
28124


 0.00
no rank
1
1
2
0
185291

1
1
2030927

 0.00
species

0
1853231
3
1
1
family

 0.00

genus

 0.00
574697
0
1
1
2

1
1
544645

 0.00
species


 0.00
family
1
4
5
0
171550

1
239759
1
4
4
genus

 0.00


 0.00
no rank
2
2
1
0
538948

2
1
538949

 0.00
species

1
1
626932

 0.00
species

1970189
0
1
1
4
order

 0.00

family

 0.00
1471398
0
3
1
1

0
1952944
2
1
1
no rank

 0.00

species

 0.00
2053594
1
1

0
117743
143
360
1
class

 0.01


 0.01
order
1
360
142
200644
13


 0.00
family
1
193
41
2762318
5

107
1
3
23
501783

 0.00
genus

237258
1
20
species

 0.00

2004710
64
1
species

 0.00


 0.00
genus
3
1
6
308865
3


 0.00
species
1
1
1117645

species

 0.00
238
2
1

genus

 0.00
0
59734
1
7
3

species

 0.00
343874
1
6

1
1
247

 0.00
species


 0.00
genus
2
1
1
0
1013

1014
1
1
species

 0.00


 0.00
genus
2
1
1
0
1433995

1
1
1118202

 0.00
species

2766734
0
2
1
1
genus

 0.00


 0.00
species
1
1
2898187

no rank

 0.00
4
2782232
23
62
1


 0.00
genus
1
8
4
2782231
0

1
4
266748

 0.00
species


 0.00
species
1
2
421525

1
2
266749

 0.00
species

1
15
3
2782229
0

 0.00
genus

13
1
2487072

 0.00
species


 0.00
species
2
1
1416779


 0.00
genus
15
35
1
10
59732

1
4
2754694

 0.00
species

1
1
1813611

 0.00
species

1
2
2929799

 0.00
species

7
8
1
1
2593645

 0.00
no rank

1
1
1721091

 0.00
species

2594269
1
1
species

 0.00


 0.00
species
1
1
2015076

1
1
878220

 0.00
species

2487064
1
2
species

 0.00


 0.00
species
1
1
2478663

1
1
1493872

 0.00
species

1
1
1609531

 0.00
species

species

 0.00
536441
7
1

1324352
1
1
species

 0.00

2
1
3
1778601
0

 0.00
genus


 0.00
no rank
1
3
2630820

no rank

 0.00
0
403978
2
2
1

species

 0.00
2021391
2
1

1
1
2
0
213321

 0.00
no rank


 0.00
species
1
1
213322

family

 0.00
0
39782
1
6
3

1
6
2
34098
0

 0.00
genus


 0.00
species
1
6
1653831

15
49546
1
145
93
family

 0.00

2
1
2
2893197
0

 0.00
genus

1
2
2893202

 0.00
species

3
1
1
0
252306

 0.00
genus

species

 0.00
0
252307
1
1
2

strain

 0.00
313596
1
1


 0.00
genus
4
1
2
178469
0

4
1
616991

 0.00
species


 0.00
genus
3
1
1
216431
0


 0.00
species
1
1
2
313588
0


 0.00
strain
1
1
216432


 0.00
genus
2
1
1
2831916
0


 0.00
species
1
1
2094025

1
2
143222

 0.00
genus

1518147
0
1
1
2
genus

 0.00

1790137
1
1
species

 0.00

2
1
3
0
225842

 0.00
genus

2
2
1
2644710
0

 0.00
no rank

species

 0.00
1798225
1
2


 0.00
genus
1
1
3
0
2058174

2
1
1
2631190
0

 0.00
no rank

species

 0.00
2908210
1
1

3
1
5
0
527198

 0.00
genus


 0.00
no rank
2
1
5
2643887
0


 0.00
species
5
1
2027857


 0.00
genus
1
7
6
0
1016

1
2
1019

 0.00
species

1017
2
1
species

 0.00

2
1
1
2640652
0

 0.00
no rank


 0.00
species
1
1
2545799

1848904
1
2
species

 0.00


 0.00
genus
3
1
1
261827
0

no rank

 0.00
0
2615009
2
1
1

species

 0.00
2686366
1
1

2
1
1
61432
0

 0.00
no rank

1871037
1
1
species

 0.00


 0.00
genus
4
5
1
252356
0


 0.00
no rank
2
1
1
2615042
0

species

 0.00
2496865
1
1

4
1
1836467

 0.00
species

1
1
2
0
76831

 0.00
genus

480520
1
1
species

 0.00

104267
0
2
1
1
genus

 0.00

species

 0.00
1850252
1
1

3
2
1
291183
0

 0.00
genus

0
2647285
1
2
2
no rank

 0.00

2057808
2
1
species

 0.00

genus

 0.00
0
49277
1
1
3

no rank

 0.00
0
2615011
2
1
1

species

 0.00
2570561
1
1

genus

 0.00
0
1573059
3
1
3

0
2623658
2
3
1
no rank

 0.00

1
3
2916754

 0.00
species

3
1
3
104264
0

 0.00
genus

species

 0.00
979
1
1

no rank

 0.00
2634405
1
2

genus

 0.00
0
52959
4
1
2


 0.00
species
1
1
2818493

1
1
2
196858
0

 0.00
no rank

species

 0.00
1855336
1
1

2
1
2
0
343334

 0.00
genus


 0.00
species
2
1
343403

3
1
1
0
363408

 0.00
genus


 0.00
no rank
2
1
1
0
2615035


 0.00
species
1
1
2719911


 0.00
genus
1
3
3
286104
0

species

 0.00
1382466
1
2


 0.00
species
1
1
2686077

76
1
23
22
237

 0.00
genus

species

 0.00
1114867
1
1

1
1
1751095

 0.00
species

1
1
2547394

 0.00
species

species

 0.00
2816357
2
1

1
1
1751056

 0.00
species


 0.00
no rank
1
42
11
196869
18

species

 0.00
2294119
1
1

3
1
2893885

 0.00
species


 0.00
species
1
4
935222

species

 0.00
2937442
1
2


 0.00
species
1
7
1179672

1
1
2478552

 0.00
species

1
3
2724135

 0.00
species


 0.00
species
1
1
2893886


 0.00
species
1
1
2614441

1
1
2739062

 0.00
species


 0.00
species
1
1
96345


 0.00
species
1
1
2602769


 0.00
species
1
1
459526


 0.00
species
1
1
1341165

species

 0.00
986
1
1

1306519
1
1
species

 0.00

1
1
2
358023
0

 0.00
genus

species

 0.00
1622118
1
1

8
3
1
0
1853228

 0.00
class

1853229
0
7
3
1
order

 0.00


 0.00
family
6
1
3
563835
1


 0.00
genus
2
1
1
0
1004303

species

 0.00
2875540
1
1


 0.00
genus
3
1
1
79328
0

2
1
1
0
79329

 0.00
species

strain

 0.00
485918
1
1


 0.00
class
51
1
58
0
768503


 0.00
order
1
51
57
768507
1

family

 0.00
2762301
0
2
1
4

1
2
3
869806
0

 0.00
genus


 0.00
species
1
2
2
1006
0

643867
2
1
strain

 0.00

family

 0.00
200667
0
1
2
5

2
1
1
340671
0

 0.00
no rank

species

 0.00
1257021
1
1

genus

 0.00
59739
0
1
1
2


 0.00
species
1
1
2494373


 0.00
family
1
6
8
0
2896860

3
1
1
861914
0

 0.00
genus

2
1
1
0
651143

 0.00
species


 0.00
strain
1
1
1166018

0
2173039
2
3
1
genus

 0.00


 0.00
species
1
3
1784714

0
120831
2
1
2
genus

 0.00

species

 0.00
2747268
2
1

family

 0.00
0
2762286
4
1
2

3
2
1
0
396811

 0.00
genus


 0.00
no rank
2
1
2
0
2685541

1
2
2904246

 0.00
species

family

 0.00
0
1853232
11
20
1

3
1
3
0
323449

 0.00
genus

2
1
388950

 0.00
species

species

 0.00
2694930
1
1


 0.00
genus
1
16
5
2
89966

2502781
1
9
species

 0.00

3
1
5
2615202
2

 0.00
no rank

2675878
1
1
species

 0.00

1
2
1484118

 0.00
species

genus

 0.00
0
299566
1
1
2

2745197
1
1
species

 0.00


 0.00
family
2
1
4
1501348
0

genus

 0.00
281119
0
3
1
2


 0.00
species
1
2
2
0
281120

452471
1
2
strain

 0.00

10
1
11
89373
0

 0.00
family

3
107
7
9
1
genus

 0.00


 0.00
species
1
1
1379870


 0.00
species
1
1
2666025


 0.00
no rank
2
1
3
0
2621999


 0.00
species
1
1
2710596


 0.00
species
1
1
2520506

2
1
564064

 0.00
species


 0.00
genus
1
1
3
978
0

1
1
2
985
0

 0.00
species

1
1
269798

 0.00
strain

3
1
4
0
1937968

 0.00
family

1937972
0
3
3
1
genus

 0.00

species

 0.00
999
0
2
1
3

1
3
880071

 0.00
strain

563798
0
3
1
5
family

 0.00

3
1
4
246875
0

 0.00
genus

species

 0.00
1727163
1
1


 0.00
no rank
2
1
2
2641541
0


 0.00
species
1
2
2772483

3
1
1
1379697
0

 0.00
phylum


 0.00
no rank
1
1
2
2751484
0

1
1
2053527

 0.00
species

3
1
6
142182
0

 0.00
phylum

0
219685
3
1
5
class

 0.00

4
1
3
219686
0

 0.00
order

3
1
3
219687
1

 0.00
family

genus

 0.00
0
1706036
2
2
1

861299
1
2
species

 0.00


 0.00
clade
1
40
53
1783257
0

phylum

 0.00
0
204428
1
1
7

class

 0.00
0
204429
6
1
1

order

 0.00
0
1963360
1
1
5

family

 0.00
0
92713
4
1
1

genus

 0.00
282132
0
3
1
1


 0.00
species
1
1
2
362787
0

264201
1
1
strain

 0.00

17
7
1
74201
0

 0.00
phylum


 0.00
class
1
1
6
0
1955630

0
717963
1
1
5
order

 0.00

family

 0.00
0
717964
4
1
1

genus

 0.00
0
511745
3
1
1


 0.00
no rank
1
1
2
0
2628272

2795386
1
1
species

 0.00

1
5
5
0
414999

 0.00
class


 0.00
order
4
1
5
0
415000

1
5
3
4
134623

 0.00
family


 0.00
genus
2
1
1
1961799
0

1
1
1838286

 0.00
species

1
1
5
0
203494

 0.00
class


 0.00
order
1
1
4
48461
0

3
1
1
1647988
0

 0.00
family

1951308
0
1
1
2
no rank

 0.00

1
1
2562705

 0.00
species


 0.00
phylum
28
32
1
3
203682

class

 0.00
0
2517206
1
1
5

order

 0.00
1127829
0
1
1
4

family

 0.00
1127830
0
3
1
1

genus

 0.00
0
380738
2
1
1


 0.00
species
1
1
174633

203683
1
22
1
28
class

 0.00

21
1
11
0
2691355

 0.00
order


 0.00
family
1
21
10
1914233
5

0
2051044
2
1
1
genus

 0.00


 0.00
species
1
1
692036


 0.00
genus
2
4
1
0
2807415


 0.00
species
4
1
2528023


 0.00
no rank
8
1
2
2052163
0


 0.00
species
1
8
2052164


 0.00
genus
2
1
1
2731450
0

1
1
2598579

 0.00
species

1
2
113

 0.00
genus

order

 0.00
1
112
5
1
6

1
4
5
0
126

 0.00
family


 0.00
genus
2
1
3
1649453
2

1
1
2527978

 0.00
species

2795777
0
1
1
2
genus

 0.00

species

 0.00
2528026
1
1

order

 0.00
0
2691356
4
1
1

3
1
1
1763524
0

 0.00
family


 0.00
genus
2
1
1
0
1511635

1
1
406548

 0.00
species

phylum

 0.00
0
200783
6
1
1


 0.00
class
1
1
5
187857
0

order

 0.00
0
32069
4
1
1


 0.00
family
1
1
3
0
64898

75905
0
1
1
2
genus

 0.00

species

 0.00
75906
1
1
