# Supplementary material for: Microbiome and mitogenomics of the chigger mite Pentidionis agamae: potential role as an Orientia vector and associations with divergent clades of Wolbachia and Borrelia
Source: BMC Genomics. 2024 Apr 17;25:380. doi: 10.1186/s12864-024-10301-6 (PMC11025265; doi:10.1186/s12864-024-10301-6)
Supplement: Supplementary file 5 — Additional file 5: Individual gene trees for single copy orthologs from Wolbachia [file 12864_2024_10301_MOESM5_ESM.pdf]

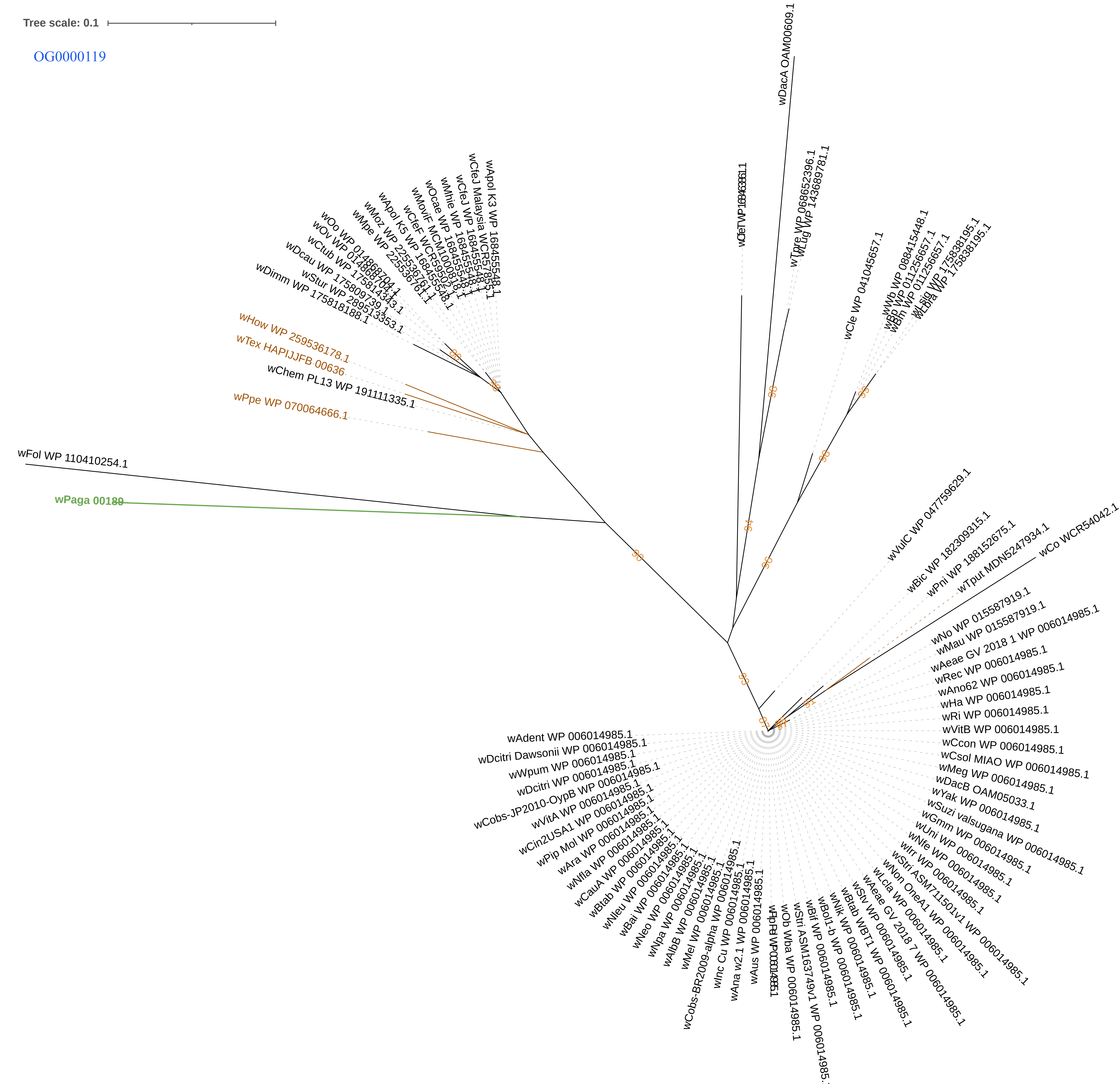

Tree scale: 0.1
OG0000119
wDacA OAM00609.1
wTpre WP 068652396.1
wLug WP 143689781.1
wCle WP 041045657.1
wWb WP 088415448.1
wBp WP 011256657.1
wBm WP 011256657.1
wLsig WP 175838195.1
wLbra WP 175838195.1
wVulC WP 047759629.1
wBic WP 182309315.1
wPni WP 188152675.1
wTput MDN5247934.1
wCo WCR54042.1
wNo WP 015587919.1
wMau WP 015587919.1
wAeae GV 2018 1 WP 006014985.1
wRec WP 006014985.1
wAno62 WP 006014985.1
wHa WP 006014985.1
wRi WP 006014985.1
wVitB WP 006014985.1
wCcon WP 006014985.1
wCsol MIAO WP 006014985.1
wMeg WP 006014985.1
wDacB OAM05033.1
wYak WP 006014985.1
wSuzi valsugana WP 006014985.1
wGmm WP 006014985.1
wUni WP 006014985.1
wNfe WP 006014985.1
wIrr WP 006014985.1
wStri ASM711501v1 WP 006014985.1
wNon OneA1 WP 006014985.1
wLcla WP 006014985.1
wAeae GV 2018 7 WP 006014985.1
wStv WP 006014985.1
wBtab WBT1 WP 006014985.1
wNik WP 006014985.1
wBol1-b WP 006014985.1
wBif WP 006014985.1
wOb Wba WP 006014985.1
wAus WP 006014985.1
wAna w2.1 WP 006014985.1
wInc Cu WP 006014985.1
wCobs-BR2009-alpha WP 006014985.1
wMel WP 006014985.1
wAlbB WP 006014985.1
wNpa WP 006014985.1
wNeo WP 006014985.1
wBai WP 006014985.1
wNleu WP 006014985.1
wBtab WP 006014985.1
wCauA WP 006014985.1
wNfla WP 006014985.1
wAra WP 006014985.1
wPip Mol WP 006014985.1
wCin2USA1 WP 006014985.1
wVitA WP 006014985.1
wCobs-JP2010-OypB WP 006014985.1
wDcitri WP 006014985.1
wWpum WP 006014985.1
wDcitri Dawsonii WP 006014985.1
wAdent WP 006014985.1
wFol WP 110410254.1
wPaga 00189
wPpe WP 070064666.1
wChem PL13 WP 191111335.1
wTex HAPIJJFB 00636
wHow WP 259536178.1
wDimm WP 175818188.1
wStur WP 289513353.1
wDcau WP 175809739.1
wCtub WP 175814343.1
wOv WP 014868704.1
wOo WP 014868704.1
wMpe WP 225536761.1
wMoz WP 225536761.1
wApol K5 WP 168455548.1
wOcae WP 168455548.1
wMhie WP 168455548.1
wCfeJ WP 168455548.1
wApol K3 WP 168455548.1

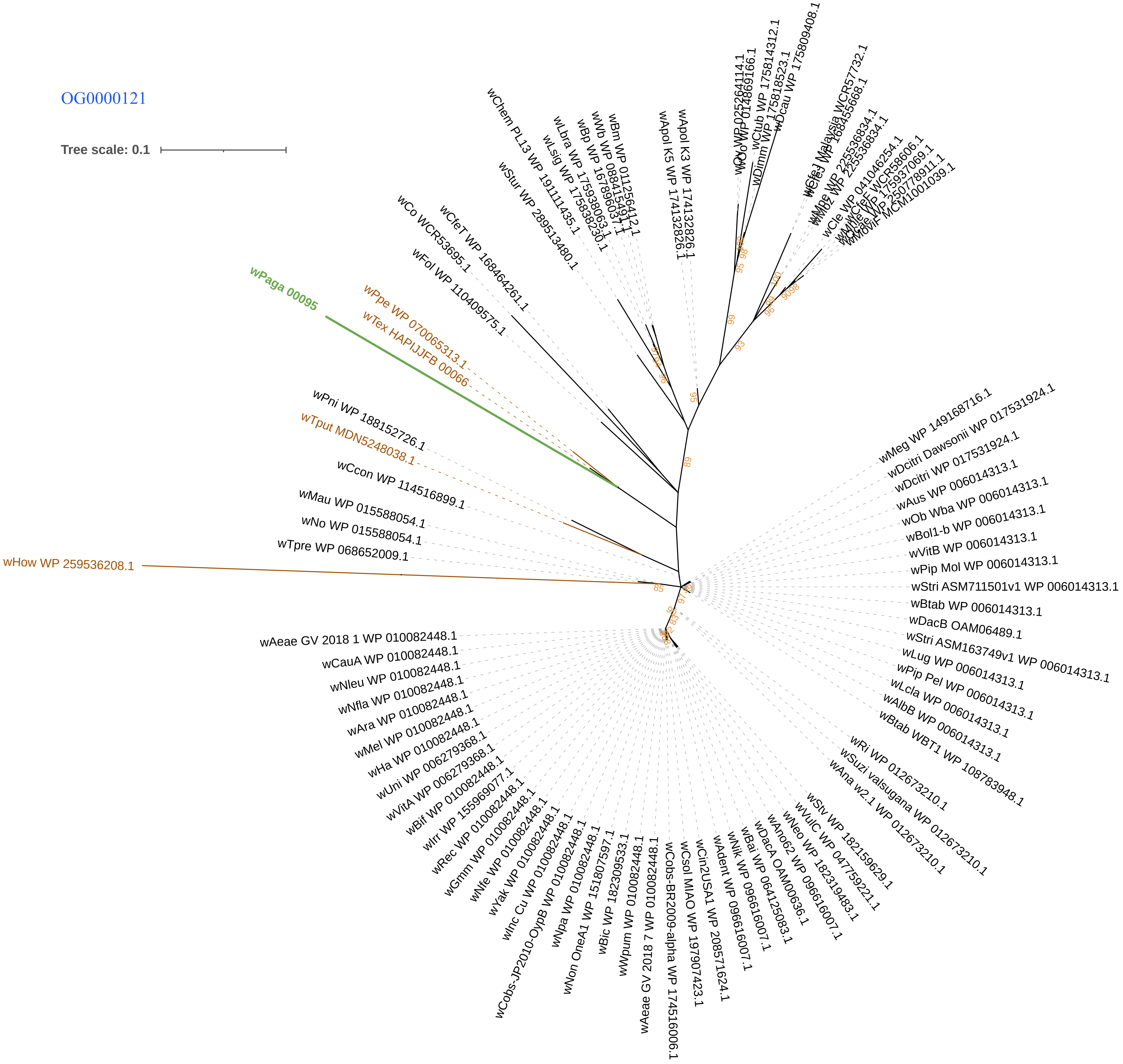
OG0000121
Tree scale: 0.1
wChem PL13 WP 191111435.1
wStur WP 289513480.1
wLsig WP 175838230.1
wLbra WP 175938063.1
wBp WP 167896037.1
wWb WP 088415491.1
wBm WP 011256412.1
wApol K5 WP 174132826.1
wApol K3 WP 174132826.1
wOv WP 025264114.1
wOo WP 014869166.1
wCtub WP 175814312.1
wDimm WP 175818523.1
wDcau WP 175809408.1
wCfeJ Malaysia WCR57732.1
wCfeJ WP 168455668.1
wMpe WP 225536834.1
wMoz WP 225536834.1
wCle WP 041046254.1
wCfeF WCR58606.1
wCfeT WP 168464261.1
wCo WCR53695.1
wFol WP 110409575.1
wPaga 00095
wPpe WP 070065313.1
wTex HAPIJJFB 00066
wPni WP 188152726.1
wTput MDN5248038.1
wCcon WP 114516899.1
wMau WP 015588054.1
wNo WP 015588054.1
wTpre WP 068652009.1
wHow WP 259536208.1
wMeg WP 149168716.1
wDcitri Dawsonii WP 017531924.1
wDcitri WP 017531924.1
wAus WP 006014313.1
wOb Wba WP 006014313.1
wBol1-b WP 006014313.1
wVitB WP 006014313.1
wPip Mol WP 006014313.1
wStri ASM711501v1 WP 006014313.1
wBtab WP 006014313.1
wDacB OAM06489.1
wStri ASM163749v1 WP 006014313.1
wLug WP 006014313.1
wPip Pel WP 006014313.1
wLcla WP 006014313.1
wAlbB WP 006014313.1
wBtab WBT1 WP 108783948.1
wRi WP 012673210.1
wSuzi valsugana WP 012673210.1
wAna w2.1 WP 012673210.1
wStv WP 182159629.1
wVulC WP 047759221.1
wNeo WP 182319483.1
wAno62 WP 096616007.1
wDacA OAM00636.1
wBai WP 064125083.1
wNik WP 096616007.1
wAdent WP 096616007.1
wCin2USA1 WP 208571624.1
wCsol MIAO WP 197907423.1
wCobs-BR2009-alpha WP 174516006.1
wAeae GV 2018 7 WP 010082448.1
wWpum WP 010082448.1
wBic WP 182309533.1
wNon OneA1 WP 151807597.1
wNpa WP 010082448.1
wCobs-JP2010-OypB WP 010082448.1
wInc Cu WP 010082448.1
wYak WP 010082448.1
wNfe WP 010082448.1
wGmm WP 010082448.1
wRec WP 010082448.1
wIrr WP 155969077.1
wBif WP 010082448.1
wVitA WP 006279368.1
wUni WP 006279368.1
wHa WP 010082448.1
wMel WP 010082448.1
wAra WP 010082448.1
wNfla WP 010082448.1
wNleu WP 010082448.1
wCauA WP 010082448.1
wAeae GV 2018 1 WP 010082448.1

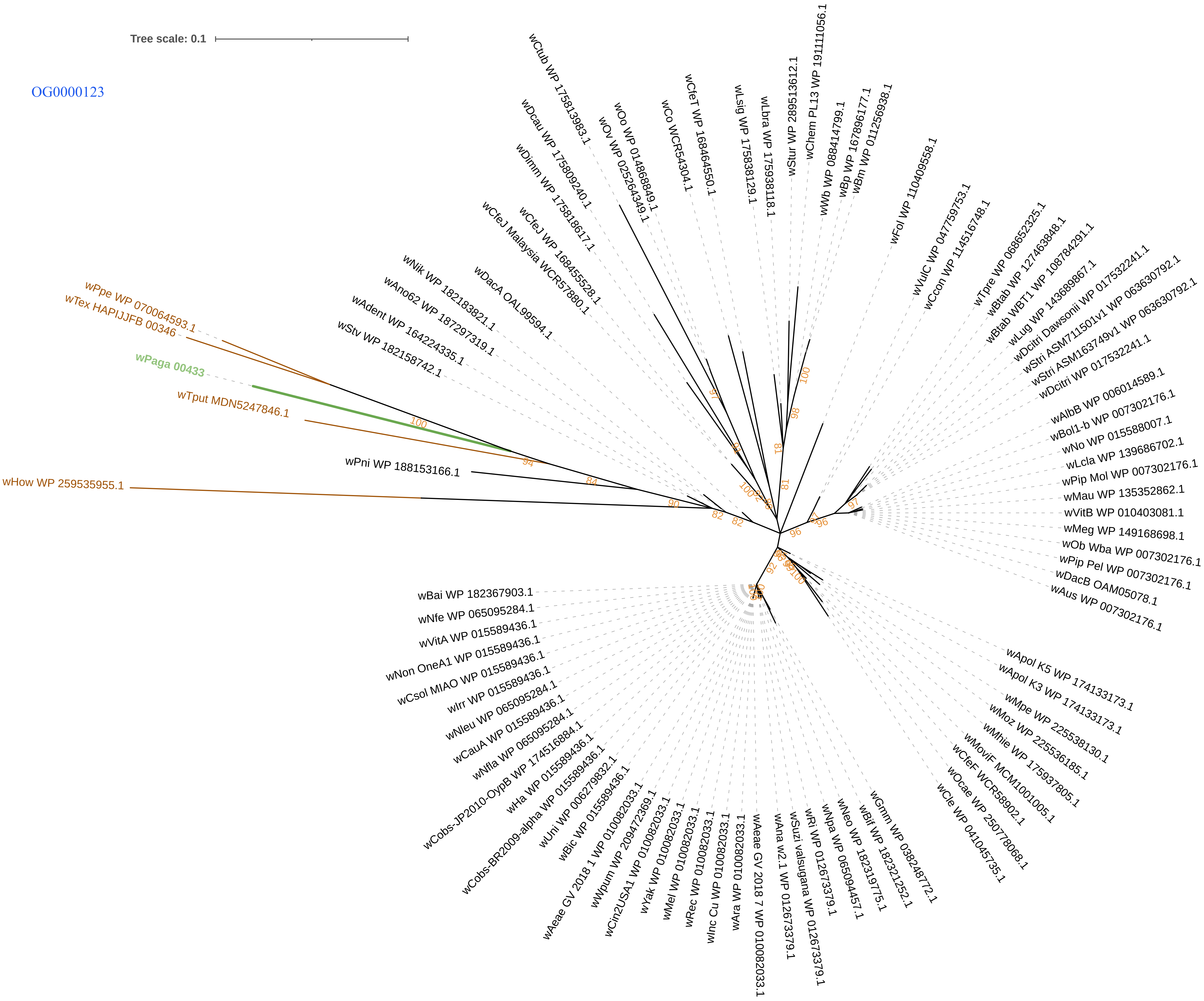
Tree scale: 0.1
OG0000123
wPpe WP 070064593.1
wTex HAPIJJFB 00346
wPaga 00433
wTput MDN5247846.1
wHow WP 259535955.1
wPni WP 188153166.1
wStv WP 182158742.1
wAdent WP 164224335.1
wAno62 WP 187297319.1
wNik WP 182183821.1
wDacA OAL99594.1
wCfeJ Malaysia WCR57880.1
wCfeJ WP 168455528.1
wDimm WP 175818617.1
wDcau WP 175809240.1
wCtub WP 175813983.1
wOv WP 025264349.1
wOo WP 014868849.1
wCo WCR54304.1
wCfeT WP 168464550.1
wLsig WP 175838129.1
wLbra WP 175938118.1
wStur WP 289513612.1
wChem PL13 WP 191111056.1
wWb WP 088414799.1
wBp WP 167896177.1
wBm WP 011256938.1
wFol WP 110409558.1
wVulC WP 047759753.1
wCcon WP 114516748.1
wTpre WP 068652325.1
wBtab WP 127463848.1
wBtab WBT1 WP 108784291.1
wLug WP 143689867.1
wDcitri Dawsonii WP 017532241.1
wStri ASM71501v1 WP 063630792.1
wStri ASM163749v1 WP 063630792.1
wDcitri WP 017532241.1
wAlbB WP 006014589.1
wBol1-b WP 007302176.1
wNo WP 015588007.1
wLcla WP 139686702.1
wPip Mol WP 007302176.1
wMau WP 135352862.1
wVitB WP 010403081.1
wMeg WP 149168698.1
wOb Wba WP 007302176.1
wPip Pel WP 007302176.1
wDacB OAM05078.1
wAus WP 007302176.1
wApol K5 WP 174133173.1
wApol K3 WP 174133173.1
wMpe WP 225538130.1
wMoz WP 225536185.1
wMhie WP 175937805.1
wMoviF MCM1001005.1
wCfeF WCR58902.1
wOcae WP 250778068.1
wCle WP 041045735.1
wGmm WP 038248772.1
wBif WP 182321252.1
wNeo WP 182319775.1
wNpa WP 065094457.1
wRi WP 012673379.1
wSuzi valsugana WP 012673379.1
wAna w2.1 WP 012673379.1
wAeae GV 2018 7 WP 010082033.1
wAra WP 010082033.1
wInc Cu WP 010082033.1
wRec WP 010082033.1
wMel WP 010082033.1
wYak WP 010082033.1
wCin2USA1 WP 010082033.1
wWpum WP 209472369.1
wAeae GV 2018 1 WP 010082033.1
wBic WP 015589436.1
wUni WP 006279832.1
wCobs-BR2009-alpha WP 015589436.1
wHa WP 015589436.1
wCobs-JP2010-OypB WP 174516884.1
wNfla WP 065095284.1
wCauA WP 015589436.1
wNleu WP 065095284.1
wIrr WP 015589436.1
wCsol MIAO WP 015589436.1
wNon OneA1 WP 015589436.1
wVitA WP 015589436.1
wNfe WP 065095284.1
wBai WP 182367903.1
100
94
84
90
82
82
97
97
100
81
98
100
81
100
96
96
97
92

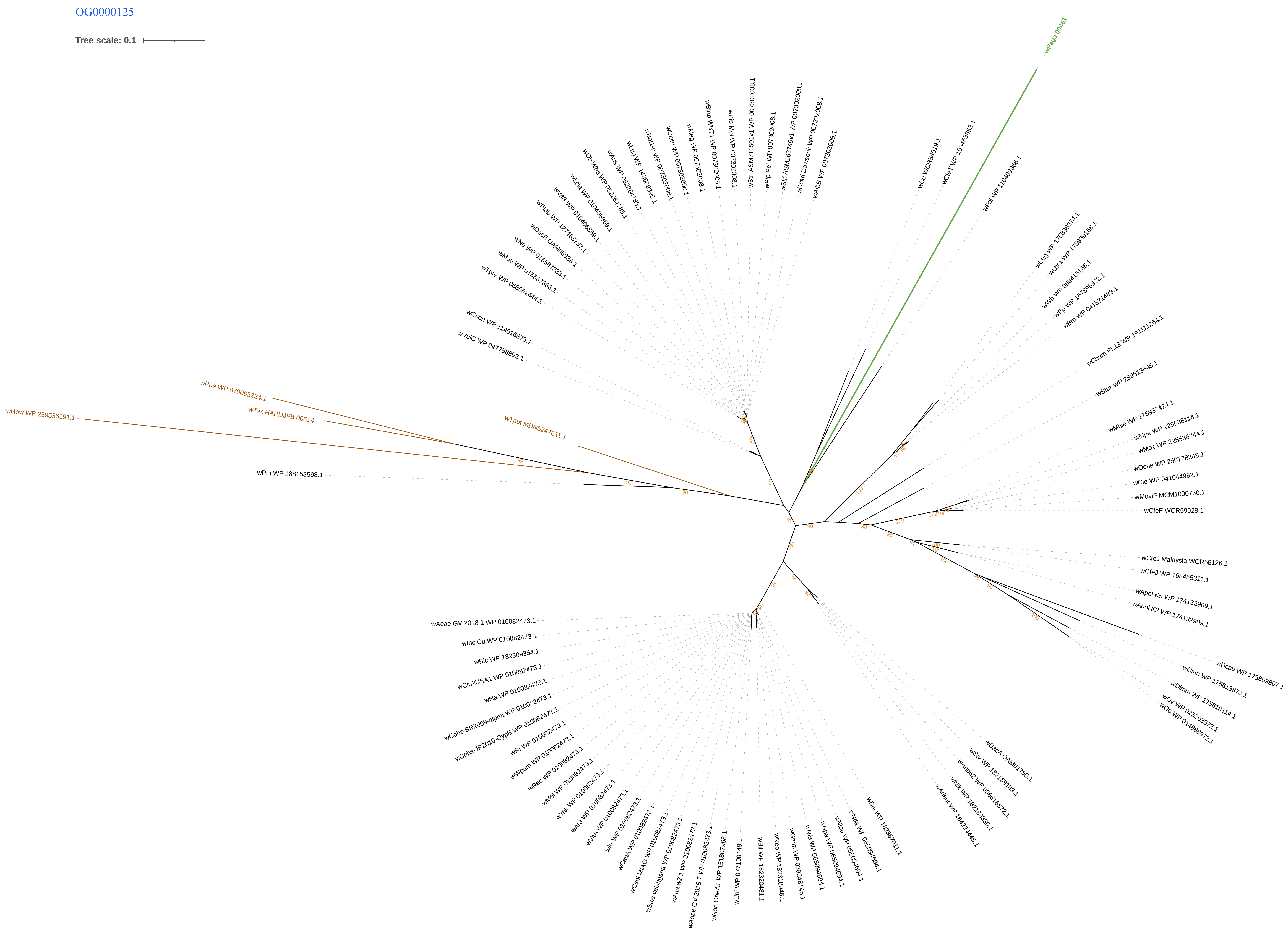

OG0000125
Tree scale: 0.1
wPaga 00461
wCo WCR54019.1
wCfeT WP 168463852.1
wFol WP 110409366.1
wLsig WP 175838374.1
wLbra WP 175939168.1
wWb WP 088415166.1
wBp WP 167896322.1
wBm WP 041571483.1
wChem PL13 WP 191111264.1
wStur WP 289513645.1
wMhie WP 175937424.1
wMpe WP 225538114.1
wMoz WP 225536744.1
wOcae WP 250778248.1
wCle WP 041044982.1
wMoviF MCM1000730.1
wCfeF WCR59028.1
wCfeJ Malaysia WCR58126.1
wCfeJ WP 168455311.1
wApol K5 WP 174132909.1
wApol K3 WP 174132909.1
wDcau WP 175809807.1
wCtub WP 175813873.1
wDimm WP 175818114.1
wOv WP 025263972.1
wOo WP 014868972.1
wDacA OAM01755.1
wStv WP 182159189.1
wAno62 WP 096616572.1
wNik WP 182183330.1
wAdent WP 164224445.1
wBai WP 182367011.1
wNfla WP 065094694.1
wNleu WP 065094694.1
wNpa WP 065094694.1
wNle WP 065094694.1
wGmm WP 038248146.1
wNeo WP 182318946.1
wBif WP 182320481.1
wUni WP 077190449.1
wNon OneA1 WP 151807968.1
wAeae GV 2018 7 WP 010082473.1
wAna w2.1 WP 010082473.1
wSuzi valsugana WP 010082473.1
wCsol MIAO WP 010082473.1
wCauA WP 010082473.1
wIrr WP 010082473.1
wVitA WP 010082473.1
wAra WP 010082473.1
wYak WP 010082473.1
wMel WP 010082473.1
wRec WP 010082473.1
wWpum WP 010082473.1
wRi WP 010082473.1
wCobs-JP2010-OypB WP 010082473.1
wCobs-BR2009-alpha WP 010082473.1
wHa WP 010082473.1
wCin2USA1 WP 010082473.1
wBic WP 182309354.1
wInc Cu WP 010082473.1
wAeae GV 2018 1 WP 010082473.1
wPni WP 188153598.1
wHow WP 259536191.1
wTex HAPIJJFB 00514
wPpe WP 070065224.1
wTput MDN5247611.1
wVulC WP 047758892.1
wCcon WP 114516875.1
wTpre WP 068652444.1
wMau WP 015587883.1
wNo WP 015587883.1
wDacB OAM05938.1
wBtab WP 127463737.1
wVitB WP 010406869.1
wLcla WP 010406869.1
wOb Wba WP 052264785.1
wAus WP 052264785.1
wLug WP 143689395.1
wBol1-b WP 007302008.1
wDcitri WP 007302008.1
wMeg WP 007302008.1
wBtab WBT1 WP 007302008.1
wPip Mol WP 007302008.1
wStri ASM711501v1 WP 007302008.1
wPip Pel WP 007302008.1
wStri ASM163749v1 WP 007302008.1
wDcitri Dawsonii WP 007302008.1
wAlbB WP 007302008.1

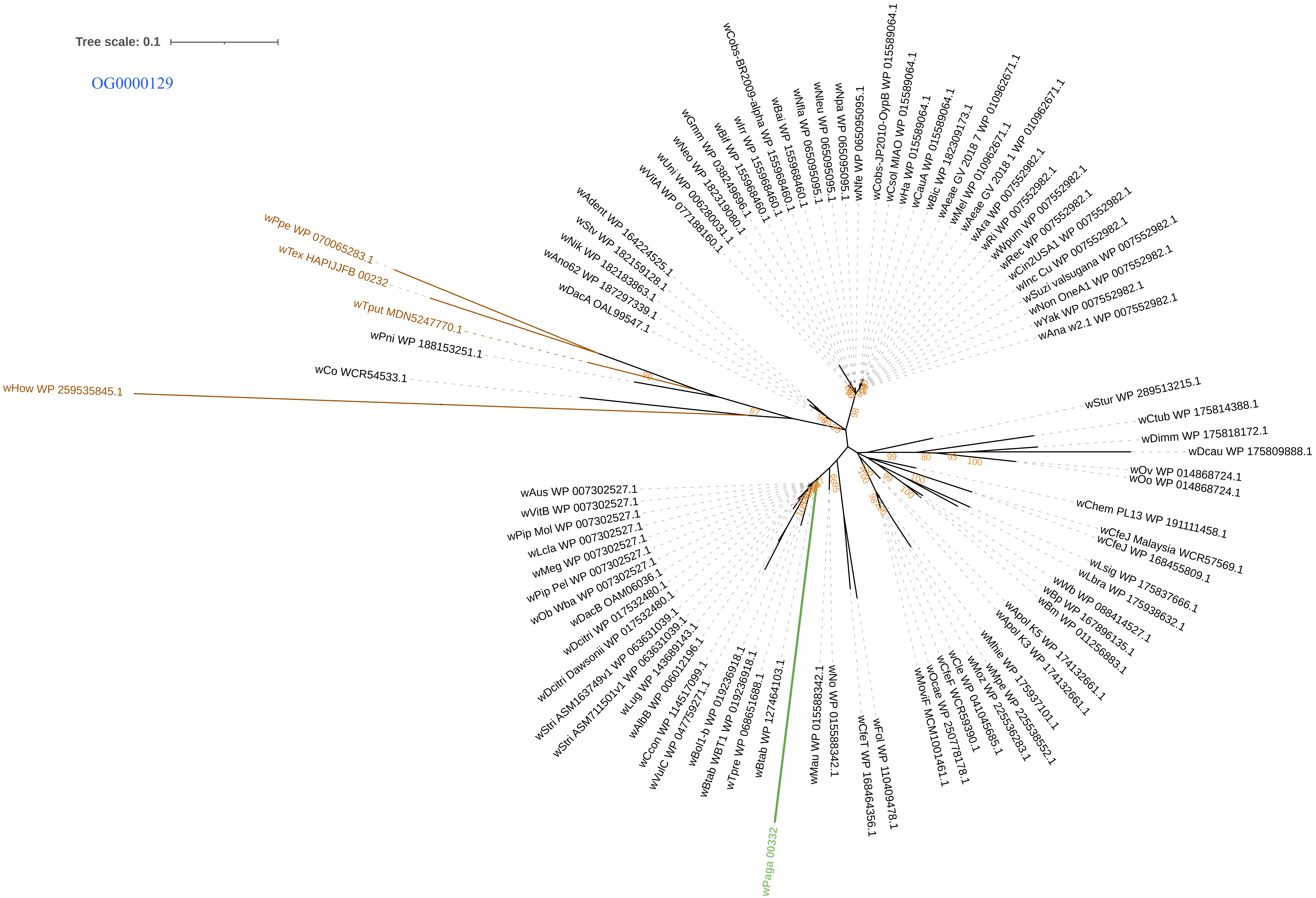
Tree scale: 0.1
OG0000129
wPpe WP 070065283.1
wTex HAPIJJFB 00232
wTput MDN5247770.1
wPni WP 188153251.1
wCo WCR54533.1
wHow WP 259535845.1
wAdent WP 164224525.1
wStv WP 182159128.1
wNik WP 182183863.1
wAno62 WP 187297339.1
wDacA OAL99547.1
wVitA WP 071188160.1
wUni WP 006280031.1
wNeo WP 182319080.1
wGmm WP 038249696.1
wBif WP 155968460.1
wIrr WP 155968460.1
wCobs-BR2009-alpha WP 155968460.1
wBai WP 155968460.1
wNfla WP 065095095.1
wNleu WP 065095095.1
wNpa WP 065095095.1
wNfe WP 065095095.1
wCobs-JP2010-OypB WP 015589064.1
wCsol MIAO WP 015589064.1
wHa WP 015589064.1
wCauA WP 015589064.1
wBic WP 182309173.1
wAeae GV 2018 7 WP 010962671.1
wMel WP 010962671.1
wAeae GV 2018 1 WP 010962671.1
wAra WP 007552982.1
wRi WP 007552982.1
wWpum WP 007552982.1
wRec WP 007552982.1
wCin2USA1 WP 007552982.1
wInc Cu WP 007552982.1
wSuzi valsugana WP 007552982.1
wNon OneA1 WP 007552982.1
wYak WP 007552982.1
wAna w2.1 WP 007552982.1
wStur WP 289513215.1
wCtub WP 175814388.1
wDimm WP 175818172.1
wDcau WP 175809888.1
wOv WP 014868724.1
wOo WP 014868724.1
wChem PL13 WP 191111458.1
wCfeJ Malaysia WCR57569.1
wCfeJ WP 168455809.1
wLsig WP 175837666.1
wLbra WP 175938632.1
wWb WP 088414527.1
wBp WP 167896135.1
wBm WP 011256883.1
wApol K5 WP 174132661.1
wApol K3 WP 174132661.1
wMhie WP 175937101.1
wMpe WP 225538552.1
wMoz WP 225536283.1
wCle WP 041045685.1
wCfeF WCR59390.1
wOcae WP 250778178.1
wMoviF MCM1001461.1
wFol WP 110409478.1
wCfeT WP 168464356.1
wNo WP 015588342.1
wMau WP 015588342.1
wPaga 00332
wBtab WP 127464103.1
wTpre WP 068651688.1
wBtab WBT1 WP 019236918.1
wBol1-b WP 019236918.1
wVulC WP 047759271.1
wCcon WP 114517099.1
wAlbB WP 006012196.1
wLug WP 143689143.1
wStri ASM711501v1 WP 063631039.1
wStri ASM163749v1 WP 063631039.1
wDcitri Dawsonii WP 017532480.1
wDcitri WP 017532480.1
wDacB OAM06036.1
wOb Wba WP 007302527.1
wPip Pel WP 007302527.1
wMeg WP 007302527.1
wLcla WP 007302527.1
wPip Mol WP 007302527.1
wVitB WP 007302527.1
wAus WP 007302527.1
99
87
98
99
80
93
100
100
99
100
100
95

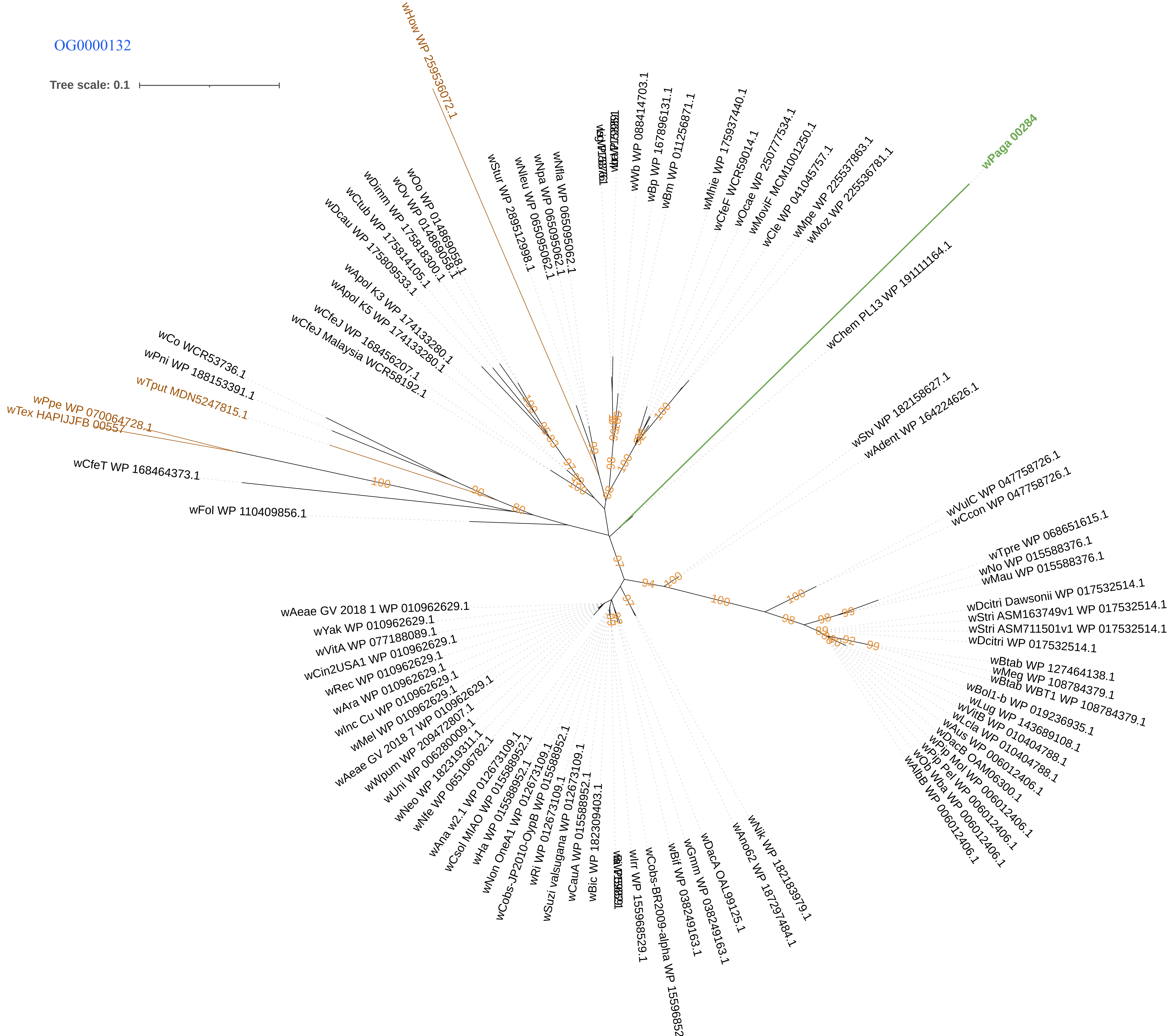

OG0000132
Tree scale: 0.1
wHow WP 259536072.1
wPaga 00284
wChem PL13 WP 191111164.1
wStur WP 289512998.1
wNleu WP 065095062.1
wNpa WP 065095062.1
wNfla WP 065095062.1
wWb WP 088414703.1
wBp WP 167896131.1
wBm WP 011256871.1
wMhie WP 175937440.1
wCfeF WCR59014.1
wOcae WP 250777534.1
wMoviF MCM1001250.1
wCle WP 041045757.1
wMpe WP 225537863.1
wMoz WP 225536781.1
wOo WP 014869058.1
wOv WP 014869058.1
wDimm WP 175818300.1
wCtub WP 175814105.1
wDcau WP 175809533.1
wApol K3 WP 174133280.1
wApol K5 WP 174133280.1
wCfeJ WP 168456207.1
wCfeJ Malaysia WCR58192.1
wCo WCR53736.1
wPni WP 188153391.1
wTput MDN5247815.1
wPpe WP 070064728.1
wTex HAPIJJFB 00557
wCfeT WP 168464373.1
wFol WP 110409856.1
wStv WP 182158627.1
wAdent WP 164224626.1
wVulC WP 047758726.1
wCcon WP 047758726.1
wTpre WP 068651615.1
wNo WP 015588376.1
wMau WP 015588376.1
wDcitri Dawsonii WP 017532514.1
wStri ASM163749v1 WP 017532514.1
wStri ASM711501v1 WP 017532514.1
wDcitri WP 017532514.1
wBtab WP 127464138.1
wMeg WP 108784379.1
wBtab WBT1 WP 108784379.1
wBol1-b WP 019236935.1
wLug WP 143689108.1
wVitB WP 010404788.1
wLcla WP 010404788.1
wAus WP 006012406.1
wDacB OAM06300.1
wPip Mol WP 006012406.1
wPip Pel WP 006012406.1
wOb Wba WP 006012406.1
wAlbB WP 006012406.1
wAeae GV 2018 1 WP 010962629.1
wYak WP 010962629.1
wVitA WP 077188089.1
wCin2USA1 WP 010962629.1
wRec WP 010962629.1
wAra WP 010962629.1
wInc Cu WP 010962629.1
wMel WP 010962629.1
wAeae GV 2018 7 WP 010962629.1
wWpum WP 209472807.1
wUni WP 006280009.1
wNeo WP 182319311.1
wNfe WP 065106782.1
wAna w2.1 WP 012673109.1
wCsol MIAO WP 015588952.1
wHa WP 015588952.1
wNon OneA1 WP 012673109.1
wCobs-JP2010-OypB WP 015588952.1
wRi WP 012673109.1
wSuzi valsugana WP 012673109.1
wCauA WP 015588952.1
wBic WP 182309403.1
wIrr WP 155968529.1
wCobs-BR2009-alpha WP 15596852
wBif WP 038249163.1
wGmm WP 038249163.1
wDacA OAL99125.1
wAno62 WP 187297484.1
wNik WP 182183979.1
100
100
100
100
100
100
100
99
99
98
98
97
97
97
94
92
89
80

OG0000133

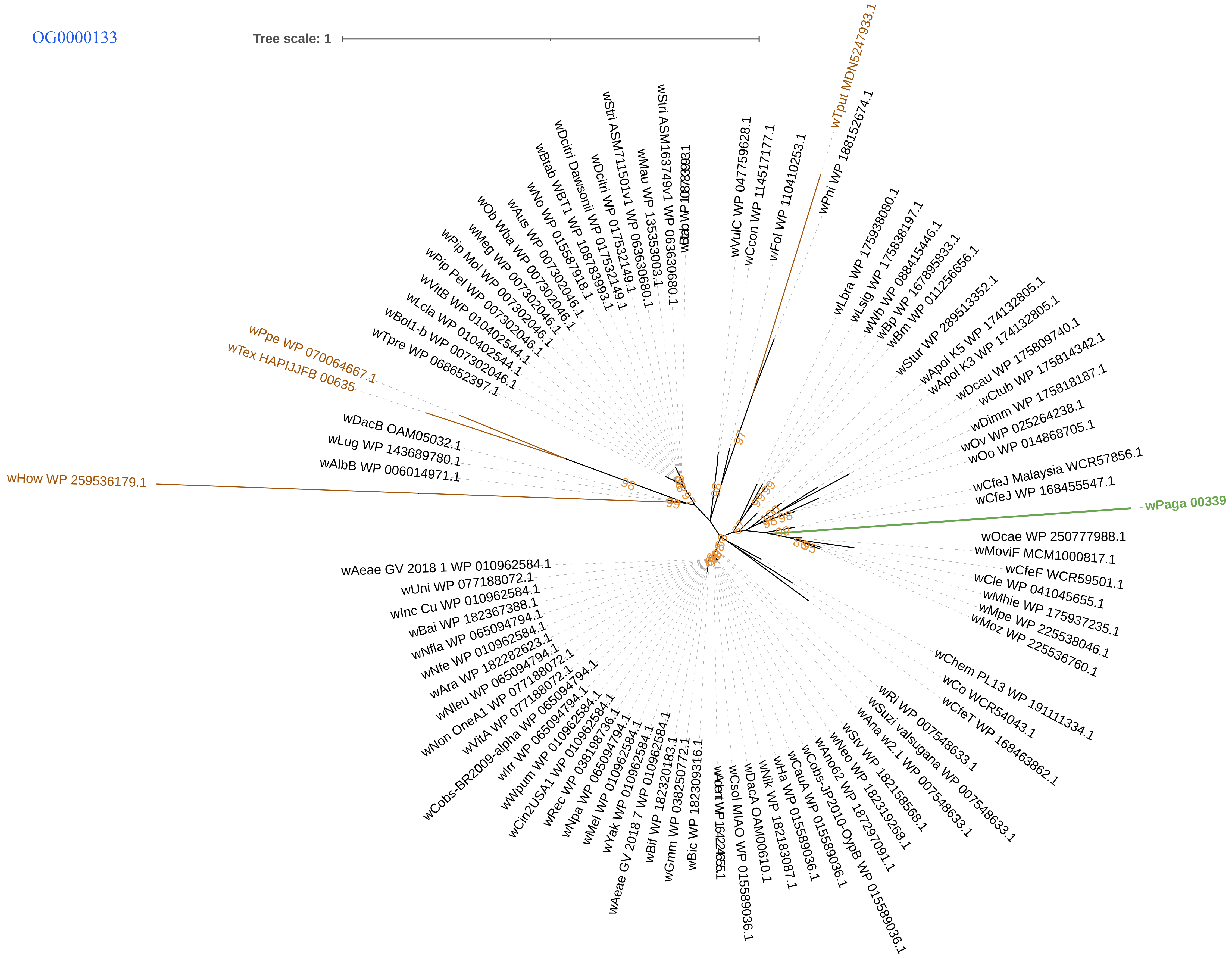

Tree scale: 1
wTput MDN5247933.1
wPni WP 188152674.1
wFol WP 110410253.1
wCcon WP 114517177.1
wVulC WP 047759628.1
wBtab WP 108783993.1
wStri ASM163749v1 WP 063630680.1
wMau WP 135353003.1
wStri ASM711501v1 WP 063630680.1
wDcitri WP 017532149.1
wDcitri Dawsonii WP 017532149.1
wBtab WBT1 WP 108783993.1
wNo WP 015587918.1
wAus WP 007302046.1
wOb Wba WP 007302046.1
wMeg WP 007302046.1
wPip Mol WP 007302046.1
wPip Pel WP 007302046.1
wVitB WP 010402544.1
wLcla WP 010402544.1
wBol1-b WP 007302046.1
wTpre WP 068652397.1
wPpe WP 070064667.1
wTex HAPIJJFB 00635
wDacB OAM05032.1
wLug WP 143689780.1
wAlbB WP 006014971.1
wHow WP 259536179.1
wAeae GV 2018 1 WP 010962584.1
wUni WP 077188072.1
wInc Cu WP 010962584.1
wBai WP 182367388.1
wNfla WP 065094794.1
wNfe WP 010962584.1
wAra WP 182282623.1
wNleu WP 065094794.1
wNon OneA1 WP 077188072.1
wVitA WP 077188072.1
wCobs-BR2009-alpha WP 065094794.1
wIrr WP 065094794.1
wWpum WP 010962584.1
wCin2USA1 WP 010962584.1
wRec WP 038198736.1
wNpa WP 065094794.1
wMel WP 010962584.1
wYak WP 010962584.1
wAeae GV 2018 7 WP 010962584.1
wBif WP 182320183.1
wGmm WP 038250772.1
wBic WP 182309316.1
wCsol MIAO WP 015589036.1
wDacA OAM00610.1
wNik WP 182183087.1
wHa WP 015589036.1
wCauA WP 015589036.1
wCobs-JP2010-OypB WP 015589036.1
wAno62 WP 187297091.1
wNeo WP 182319268.1
wStv WP 182158568.1
wAna w2.1 WP 007548633.1
wSuzi valsugana WP 007548633.1
wRi WP 007548633.1
wCfeT WP 168463862.1
wCo WCR54043.1
wChem PL13 WP 191111334.1
wMoz WP 225536760.1
wMpe WP 225538046.1
wMhie WP 175937235.1
wCle WP 041045655.1
wCfeF WCR59501.1
wMoviF MCM1000817.1
wOcae WP 250777988.1
wPaga 00339
wCfeJ WP 168455547.1
wCfeJ Malaysia WCR57856.1
wOo WP 014868705.1
wOv WP 025264238.1
wDimm WP 175818187.1
wCtub WP 175814342.1
wDcau WP 175809740.1
wApol K3 WP 174132805.1
wApol K5 WP 174132805.1
wStur WP 289513352.1
wBm WP 011256656.1
wBp WP 167895833.1
wWb WP 088415446.1
wLsig WP 175838197.1
wLbra WP 175938080.1

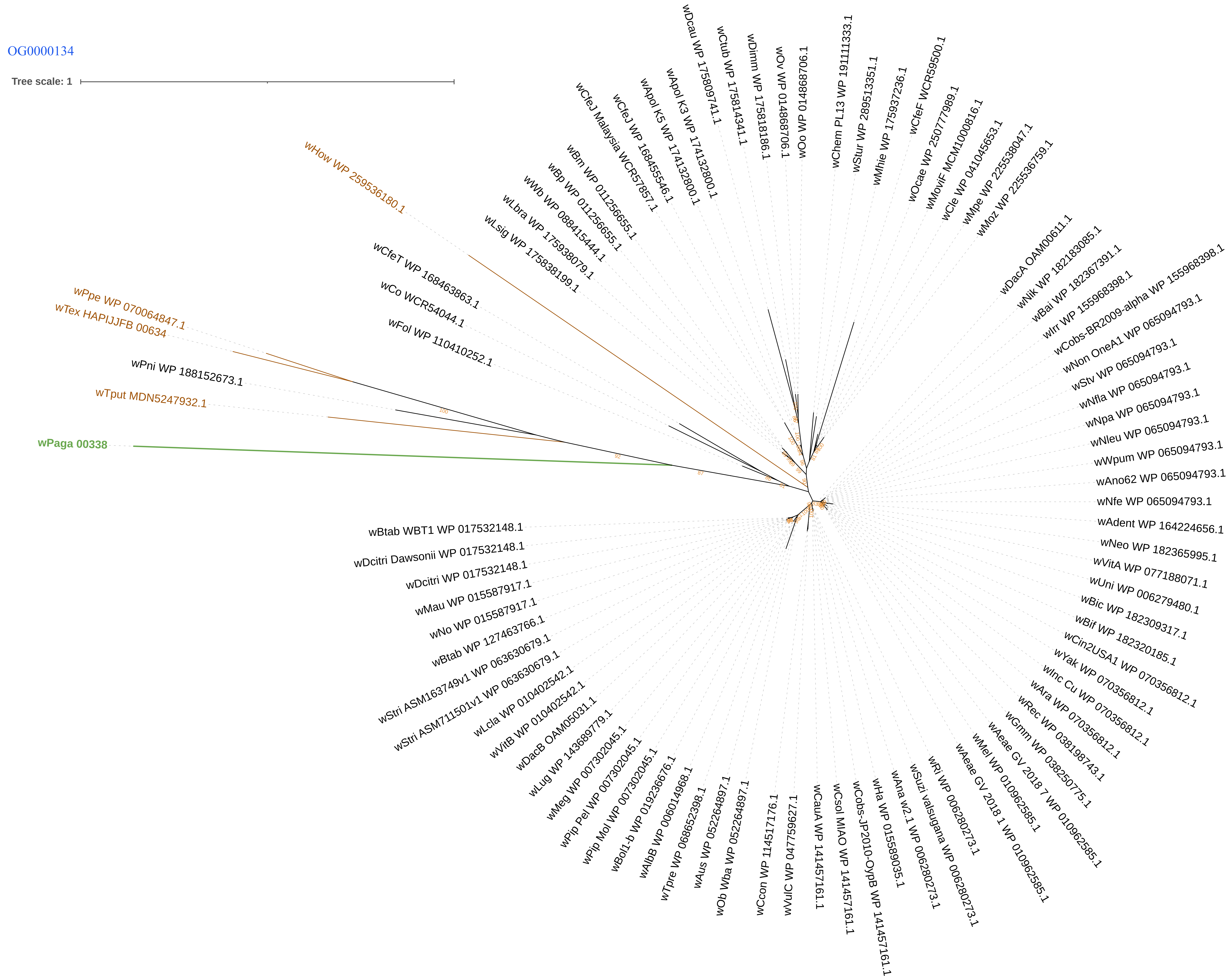
OG0000134
Tree scale: 1
wHow WP 259536180.1
wPpe WP 070064847.1
wTex HAPIJJFB 00634
wPni WP 188152673.1
wTput MDN5247932.1
wPaga 00338
wCfeT WP 168463863.1
wCo WCR54044.1
wFol WP 110410252.1
wLsig WP 175838199.1
wLbra WP 175938079.1
wWb WP 088415444.1
wBp WP 011256655.1
wBm WP 011256655.1
wCfeJ Malaysia WCR57857.1
wCfeJ WP 168455546.1
wApol K5 WP 174132800.1
wApol K3 WP 174132800.1
wDcau WP 175809741.1
wCtub WP 175814341.1
wDimm WP 175818186.1
wOv WP 014868706.1
wOo WP 014868706.1
wChem PL13 WP 191111333.1
wStur WP 289513351.1
wMhie WP 175937236.1
wCfeF WCR59500.1
wOcae WP 250777989.1
wMoviF MCM1000816.1
wCle WP 041045653.1
wMpe WP 225538047.1
wMoz WP 225536759.1
wDacA OAM00611.1
wNik WP 182183085.1
wBai WP 182367391.1
wIrr WP 155968398.1
wCobs-BR2009-alpha WP 155968398.1
wNon OneA1 WP 065094793.1
wStv WP 065094793.1
wNfla WP 065094793.1
wNpa WP 065094793.1
wNleu WP 065094793.1
wWpum WP 065094793.1
wAno62 WP 065094793.1
wNfe WP 065094793.1
wAdent WP 164224656.1
wNeo WP 182365995.1
wVitA WP 077188071.1
wUni WP 006279480.1
wBic WP 182309317.1
wBif WP 182320185.1
wCin2USA1 WP 070356812.1
wYak WP 070356812.1
wInc Cu WP 070356812.1
wAra WP 070356812.1
wRec WP 038198743.1
wGmm WP 038250775.1
wAeae GV 2018 7 WP 010962585.1
wMel WP 010962585.1
wAeae GV 2018 1 WP 010962585.1
wRi WP 006280273.1
wSuzi valsugana WP 006280273.1
wAna w2.1 WP 006280273.1
wHa WP 015589035.1
wCobs-JP2010-OypB WP 141457161.1
wCsol MIAO WP 141457161.1
wCauA WP 141457161.1
wVulC WP 047759627.1
wCcon WP 114517176.1
wOb Wba WP 052264897.1
wAus WP 052264897.1
wTpre WP 068652398.1
wAlbB WP 006014968.1
wBol1-b WP 019236676.1
wPip Mol WP 007302045.1
wPip Pel WP 007302045.1
wMeg WP 007302045.1
wLug WP 143689779.1
wDacB OAM05031.1
wVitB WP 010402542.1
wLcla WP 010402542.1
wStri ASM711501v1 WP 063630679.1
wStri ASM163749v1 WP 063630679.1
wBtab WP 127463766.1
wNo WP 015587917.1
wMau WP 015587917.1
wDcitri WP 017532148.1
wDcitri Dawsonii WP 017532148.1
wBtab WBT1 WP 017532148.1

OG0000136

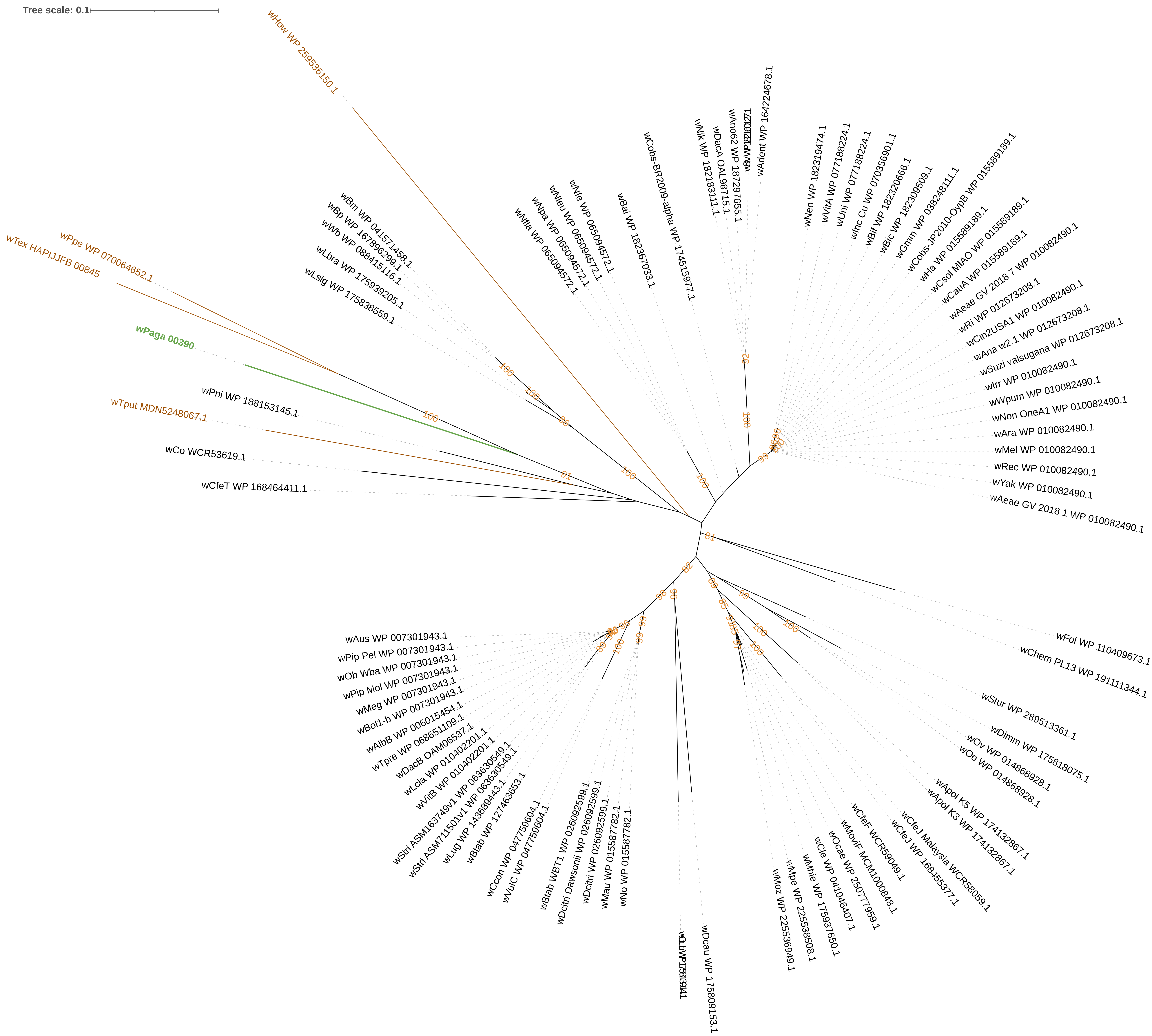

Tree scale: 0.1
wHow WP 259536150.1
wPpe WP 070064652.1
wTex HAPIJJFB 00845
wPaga 00390
wTput MDN5248067.1
wPni WP 188153145.1
wCo WCR53619.1
wCfeT WP 168464411.1
wBm WP 041571458.1
wBp WP 167896299.1
wWb WP 088415116.1
wLbra WP 175939205.1
wLsig WP 175838559.1
wNfe WP 065094572.1
wNleu WP 065094572.1
wNpa WP 065094572.1
wNfla WP 065094572.1
wBai WP 182367033.1
wCobs-BR2009-alpha WP 174515977.1
wNik WP 182183111.1
wDacA OAL98715.1
wAno62 WP 187297655.1
wAdent WP 164224678.1
wNeo WP 182319474.1
wVitA WP 077188224.1
wUni WP 077188224.1
wInc Cu WP 070356901.1
wBif WP 182320666.1
wBic WP 182309509.1
wGmm WP 038248111.1
wCobs-JP2010-OypB WP 015589189.1
wHa WP 015589189.1
wCsol MIAO WP 015589189.1
wCauA WP 015589189.1
wAeae GV 2018 7 WP 010082490.1
wRi WP 012673208.1
wCin2USA1 WP 010082490.1
wAna w2.1 WP 012673208.1
wSuzi valsugana WP 012673208.1
wIrr WP 010082490.1
wWpum WP 010082490.1
wNon OneA1 WP 010082490.1
wAra WP 010082490.1
wMel WP 010082490.1
wRec WP 010082490.1
wYak WP 010082490.1
wAeae GV 2018 1 WP 010082490.1
wFol WP 110409673.1
wChem PL13 WP 191111344.1
wStur WP 289513361.1
wDimm WP 175818075.1
wOv WP 014868928.1
wOo WP 014868928.1
wApol K5 WP 174132867.1
wApol K3 WP 174132867.1
wCfeJ Malaysia WCR58059.1
wCfeJ WP 168455377.1
wCfeF WCR59049.1
wMovif MCM1000848.1
wOcae WP 250777959.1
wCle WP 041046407.1
wMhie WP 175937650.1
wMpe WP 225538508.1
wMoz WP 225536949.1
wDcau WP 175809153.1
wNo WP 015587782.1
wMau WP 015587782.1
wDcitri WP 026092599.1
wDcitri Dawsonii WP 026092599.1
wBtab WBT1 WP 026092599.1
wVulC WP 047759604.1
wCcon WP 047759604.1
wBtab WP 127463653.1
wLug WP 143689443.1
wStri ASM711501v1 WP 063630549.1
wStri ASM163749v1 WP 063630549.1
wVitB WP 010402201.1
wLcla WP 010402201.1
wDacB OAM06537.1
wTpre WP 068651109.1
wAlbB WP 006015454.1
wBol1-b WP 007301943.1
wMeg WP 007301943.1
wPip Mol WP 007301943.1
wOb Wba WP 007301943.1
wPip Pel WP 007301943.1
wAus WP 007301943.1

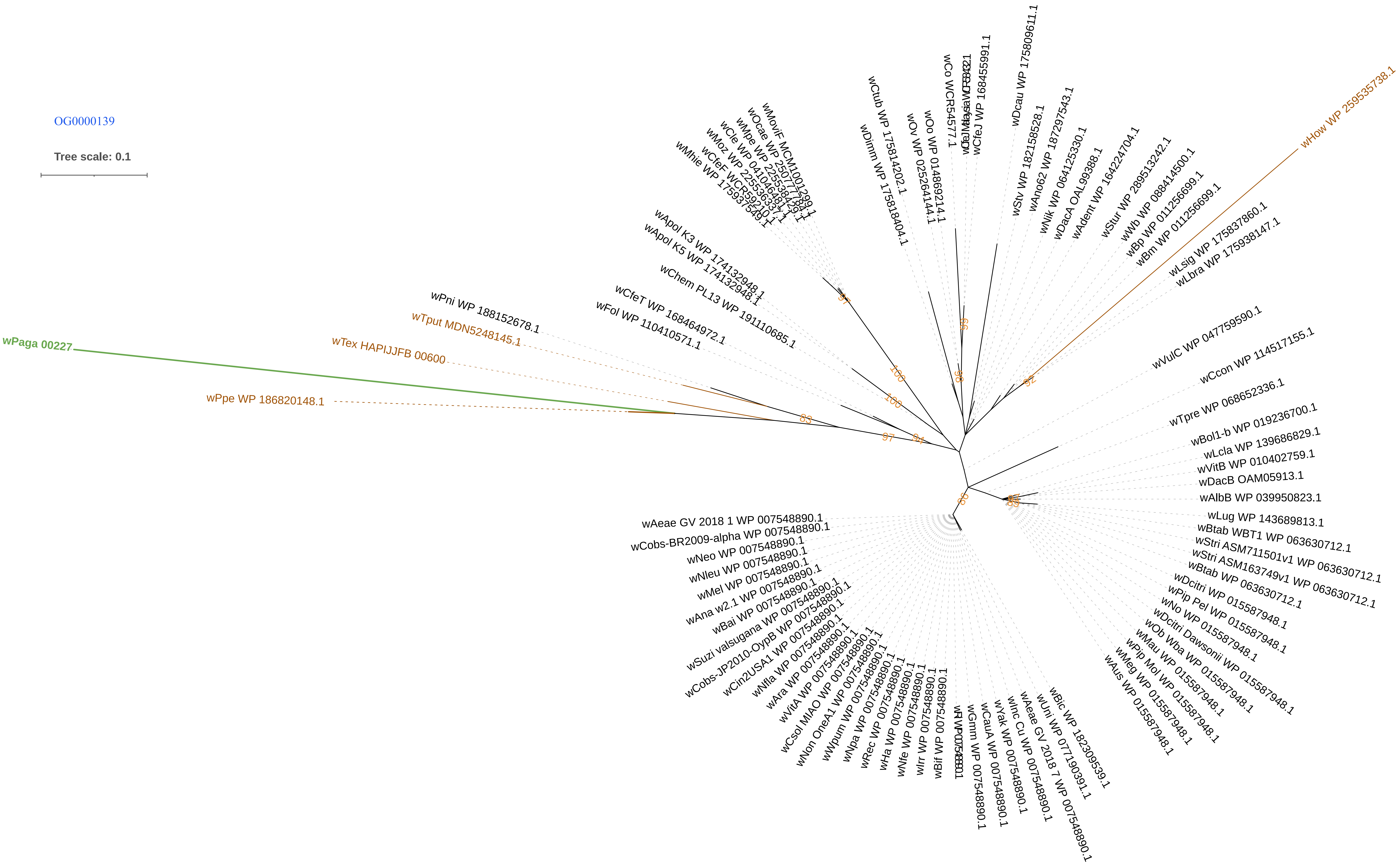

OG0000139
Tree scale: 0.1
wPaga 00227
wPpe WP 186820148.1
wTex HAPIJJFB 00600
wTput MDN5248145.1
wPni WP 188152678.1
wFol WP 110410571.1
wCfeT WP 168464972.1
wChem PL13 WP 191110685.1
wApol K5 WP 174132948.1
wApol K3 WP 174132948.1
wMhie WP 175937549.1
wCfeF WCR59210.1
wMoz WP 225536337.1
wCle WP 041046487.1
wMpe WP 225538429.1
wOcae WP 250777784.1
wMoviF MCM1001299.1
wDimm WP 175818404.1
wCtub WP 175814202.1
wOv WP 025264144.1
wOo WP 014869214.1
wCo WCR54577.1
wCfeJ WP 168455991.1
wDcau WP 175809611.1
wStv WP 182158528.1
wAno62 WP 187297543.1
wNik WP 064125330.1
wDacA OAL99388.1
wAdent WP 164224704.1
wStur WP 289513242.1
wWb WP 088414500.1
wBp WP 011256699.1
wBm WP 011256699.1
wLsig WP 175837860.1
wLbra WP 175938147.1
wHow WP 259535738.1
wVulC WP 047759590.1
wCcon WP 114517155.1
wTpre WP 068652336.1
wBol1-b WP 019236700.1
wLcla WP 139686829.1
wVitB WP 010402759.1
wDacB OAM05913.1
wAlbB WP 039950823.1
wLug WP 143689813.1
wBtab WBT1 WP 063630712.1
wStri ASM711501v1 WP 063630712.1
wStri ASM163749v1 WP 063630712.1
wBtab WP 063630712.1
wDcitri WP 015587948.1
wPip Pel WP 015587948.1
wNo WP 015587948.1
wDcitri Dawsonii WP 015587948.1
wOb Wba WP 015587948.1
wMau WP 015587948.1
wPip Mol WP 015587948.1
wMeg WP 015587948.1
wAus WP 015587948.1
wBic WP 182309539.1
wUni WP 077190391.1
wAeae GV 2018 7 WP 007548890.1
wInc Cu WP 007548890.1
wYak WP 007548890.1
wCauA WP 007548890.1
wGmm WP 007548890.1
wBif WP 007548890.1
wIrr WP 007548890.1
wNfe WP 007548890.1
wHa WP 007548890.1
wRec WP 007548890.1
wNpa WP 007548890.1
wWpum WP 007548890.1
wNon OneA1 WP 007548890.1
wCsol MIAO WP 007548890.1
wVitA WP 007548890.1
wAra WP 007548890.1
wNfla WP 007548890.1
wCin2USA1 WP 007548890.1
wCobs-JP2010-OypB WP 007548890.1
wSuzi valsugana WP 007548890.1
wBai WP 007548890.1
wAna w2.1 WP 007548890.1
wMel WP 007548890.1
wNleu WP 007548890.1
wNeo WP 007548890.1
wCobs-BR2009-alpha WP 007548890.1
wAeae GV 2018 1 WP 007548890.1

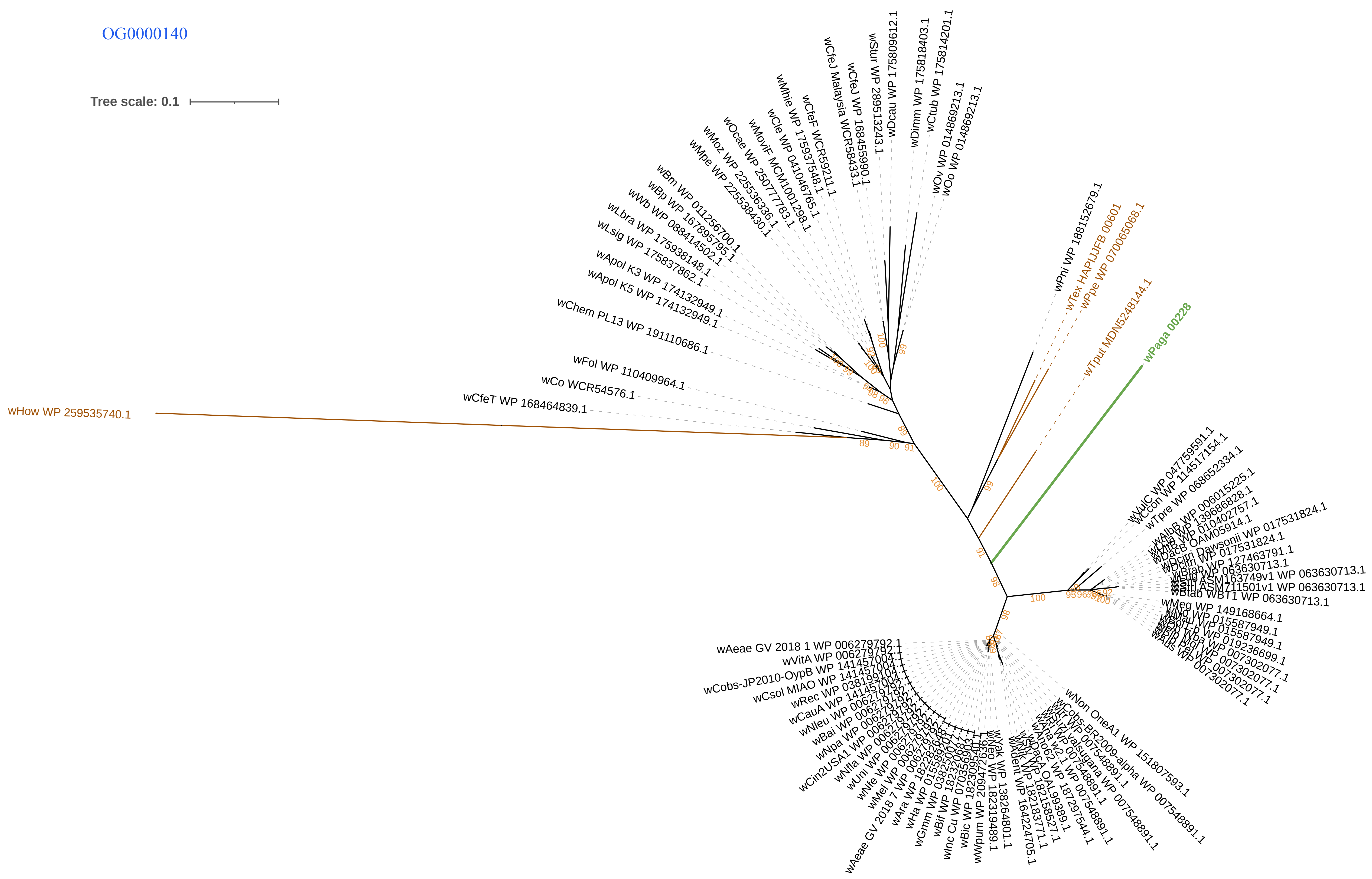
OG0000140
Tree scale: 0.1
wHow WP 259535740.1
wCfeT WP 168464839.1
wCo WCR54576.1
wFol WP 110409964.1
wChem PL13 WP 191110686.1
wApol K5 WP 174132949.1
wApol K3 WP 174132949.1
wLsig WP 175837862.1
wLbra WP 175938148.1
wWb WP 088414502.1
wBp WP 167895795.1
wBm WP 011256700.1
wMpe WP 225538430.1
wMoz WP 225536336.1
wOcae WP 250777783.1
wMoviF MCM1001298.1
wCle WP 041046765.1
wMhie WP 175937548.1
wCfeF WCR59211.1
wCfeJ Malaysia WCR58433.1
wCfeJ WP 168455990.1
wStur WP 289513243.1
wDcau WP 175809612.1
wDimm WP 175818403.1
wCtub WP 175814201.1
wOv WP 014869213.1
wOo WP 014869213.1
wPni WP 188152679.1
wTex HAPIJJFB 00601
wPpe WP 070065068.1
wTput MDN5248144.1
wPaga 00228
wUlC WP 047759591.1
wCcon WP 114517154.1
wTpre WP 068652334.1
wMeg WP 149168664.1
wAeae GV 2018 1 WP 006279792.1
wVitA WP 006279792.1
wCobs-JP2010-OypB WP 141457004.1
wCsol MIAO WP 141457004.1
wNon OneA1 WP 151807593.1
wCobs-BR2009-alpha WP 007548891.1
wYak WP 138264801.1
wNeo WP 182319489.1
wAdent WP 164224705.1
wAeae GV 2018 7 WP 182282648.1

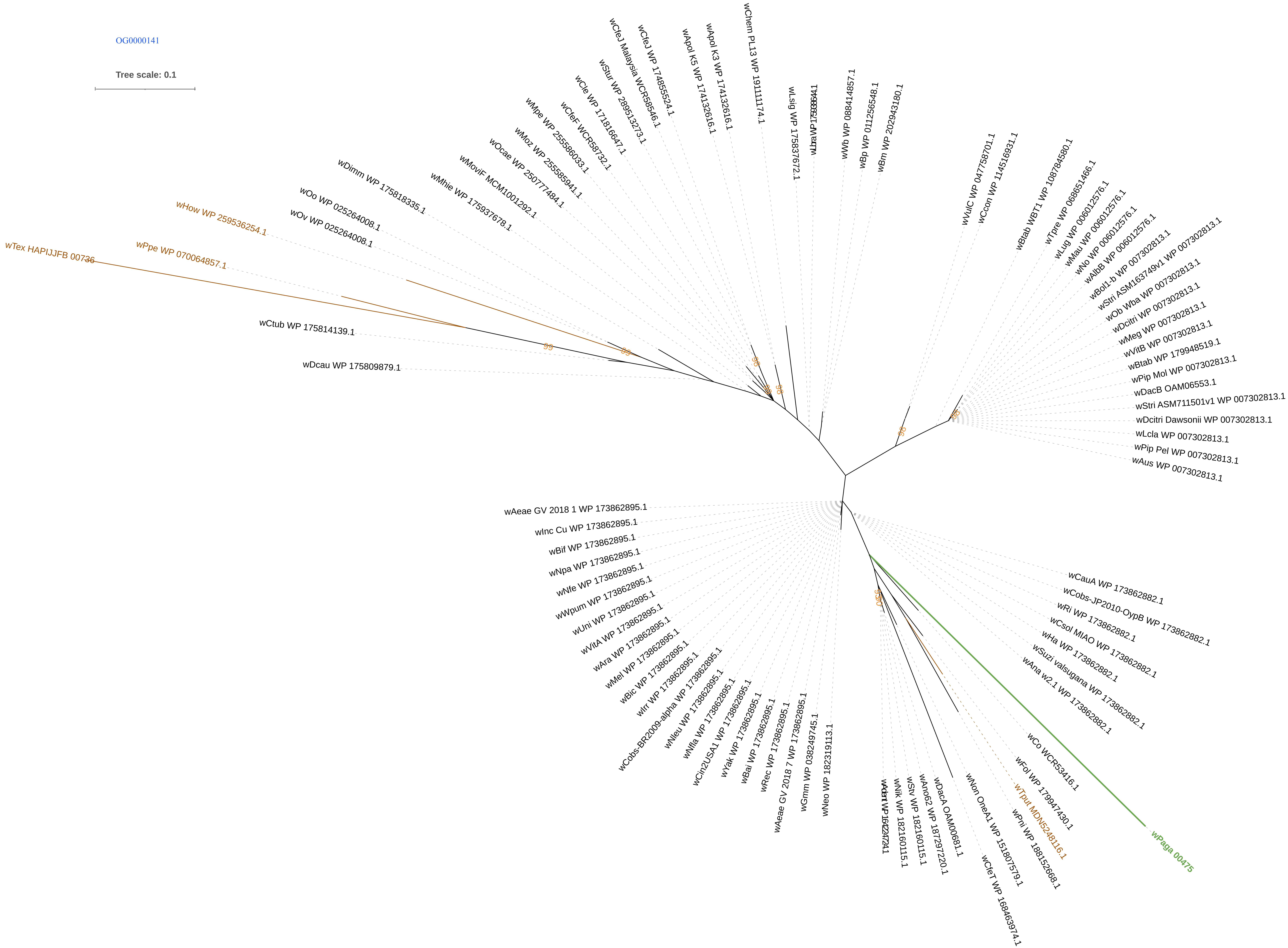

OG0000141
Tree scale: 0.1
wChem PL13 WP 191111174.1
wApol K3 WP 174132616.1
wApol K5 WP 174132616.1
wCfeJ WP 174855524.1
wCfeJ Malaysia WCR58546.1
wStur WP 289513273.1
wCle WP 171816647.1
wCfeF WCR58732.1
wMpe WP 255586033.1
wMoz WP 255585941.1
wOcae WP 250777484.1
wMoviF MCM1001292.1
wMhie WP 175937678.1
wDimm WP 175818335.1
wOo WP 025264008.1
wOv WP 025264008.1
wHow WP 259536254.1
wPpe WP 070064857.1
wTex HAPIJJFB 00736
wCtub WP 175814139.1
wDcau WP 175809879.1
wLsig WP 175837672.1
wWb WP 088414857.1
wBp WP 011256548.1
wBm WP 202943180.1
wVulC WP 047758701.1
wCcon WP 114516931.1
wBtab WBT1 WP 108784580.1
wTpre WP 068651466.1
wLug WP 006012576.1
wMau WP 006012576.1
wNo WP 006012576.1
wAlbB WP 006012576.1
wBol1-b WP 007302813.1
wStri ASM163749v1 WP 007302813.1
wOb Wba WP 007302813.1
wDcitri WP 007302813.1
wMeg WP 007302813.1
wVitB WP 007302813.1
wBtab WP 179948519.1
wPip Mol WP 007302813.1
wDacB OAM06553.1
wStri ASM711501v1 WP 007302813.1
wDcitri Dawsonii WP 007302813.1
wLcla WP 007302813.1
wPip Pel WP 007302813.1
wAus WP 007302813.1
wAeae GV 2018 1 WP 173862895.1
wInc Cu WP 173862895.1
wBif WP 173862895.1
wNpa WP 173862895.1
wNfe WP 173862895.1
wWpum WP 173862895.1
wUni WP 173862895.1
wVitA WP 173862895.1
wAra WP 173862895.1
wMel WP 173862895.1
wBic WP 173862895.1
wIrr WP 173862895.1
wCobs-BR2009-alpha WP 173862895.1
wNleu WP 173862895.1
wNfla WP 173862895.1
wCin2USA1 WP 173862895.1
wYak WP 173862895.1
wBai WP 173862895.1
wRec WP 173862895.1
wAeae GV 2018 7 WP 173862895.1
wGmm WP 038249745.1
wNeo WP 182319113.1
wNik WP 182160115.1
wStv WP 182160115.1
wAno62 WP 187297220.1
wDacA OAM00681.1
wNon OneA1 WP 151807579.1
wCfeT WP 168463974.1
wPni WP 188152668.1
wTput MDN5248116.1
wFol WP 179947430.1
wCo WCR53416.1
wPaga 00475
wCauA WP 173862882.1
wCobs-JP2010-OypB WP 173862882.1
wRi WP 173862882.1
wCsol MIAO WP 173862882.1
wHa WP 173862882.1
wSuzi valsugana WP 173862882.1
wAna w2.1 WP 173862882.1

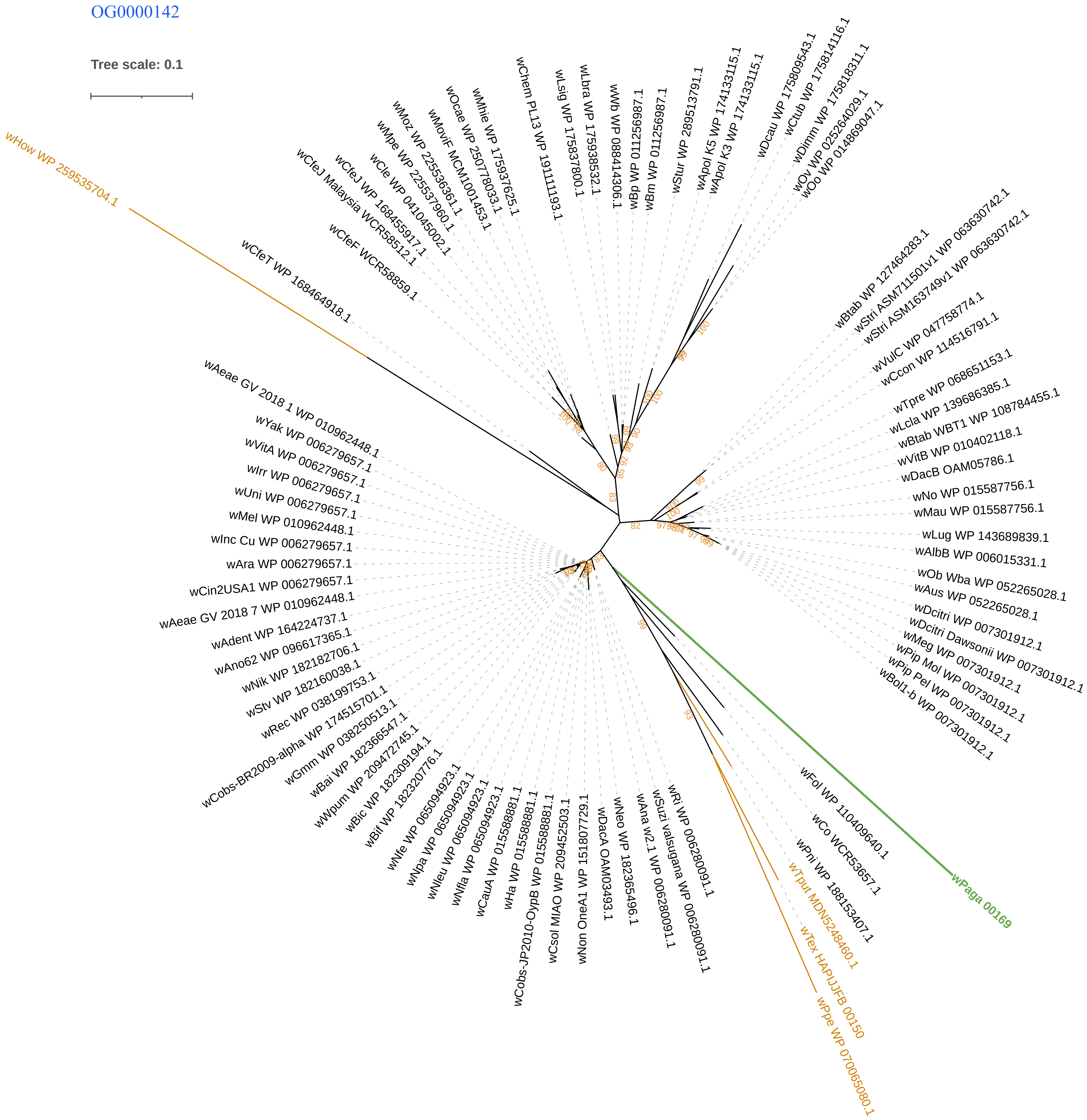
OG0000142
Tree scale: 0.1
wHow WP 259535704.1
wCfeT WP 168464918.1
wCfeF WCR58859.1
wCfeJ Malaysia WCR58512.1
wCfeJ WP 168455917.1
wCle WP 041045002.1
wMpe WP 225537960.1
wMoz WP 225536361.1
wMovIF MCM1001453.1
wOcae WP 250778033.1
wMhie WP 175937625.1
wChem PL13 WP 191111193.1
wLsig WP 175837800.1
wLbra WP 175938532.1
wWb WP 088414306.1
wBp WP 011256987.1
wBm WP 011256987.1
wStur WP 289513791.1
wApol K5 WP 174133115.1
wApol K3 WP 174133115.1
wDcau WP 175809543.1
wCtub WP 175814116.1
wDimm WP 175818311.1
wOv WP 025264029.1
wOo WP 014869047.1
wBtab WP 127464283.1
wStri ASM711501v1 WP 063630742.1
wStri ASM163749v1 WP 063630742.1
wVulC WP 047758774.1
wCcon WP 114516791.1
wTpre WP 068651153.1
wLcla WP 139686385.1
wBtab WBT1 WP 108784455.1
wVitB WP 010402118.1
wDacB OAM05786.1
wNo WP 015587756.1
wMau WP 015587756.1
wLug WP 143689839.1
wAlbB WP 006015331.1
wOb Wba WP 052265028.1
wAus WP 052265028.1
wDcitri WP 007301912.1
wDcitri Dawsonii WP 007301912.1
wMeg WP 007301912.1
wPip Mol WP 007301912.1
wPip Pel WP 007301912.1
wBol1-b WP 007301912.1
wFol WP 110409640.1
wCo WCR53657.1
wPni WP 188153407.1
wPaga 00169
wTput MDN5248460.1
wTex HAPIJJFB 00150
wPpe WP 070065080.1
wRi WP 006280091.1
wSuzi valsugana WP 006280091.1
wAna w2.1 WP 006280091.1
wNeo WP 182365496.1
wDacA OAM03493.1
wNon OneA1 WP 151807729.1
wCsol MIAO WP 209452503.1
wCobs-JP2010-OypB WP 015588881.1
wHa WP 015588881.1
wCauA WP 015588881.1
wNfla WP 065094923.1
wNleu WP 065094923.1
wNpa WP 065094923.1
wNfe WP 065094923.1
wBif WP 182320776.1
wBic WP 182309194.1
wWpum WP 209472745.1
wBai WP 182366547.1
wGmm WP 038250513.1
wCobs-BR2009-alpha WP 174515701.1
wRec WP 038199753.1
wStv WP 182160038.1
wNik WP 182182706.1
wAno62 WP 096617365.1
wAdent WP 164224737.1
wAeae GV 2018 7 WP 010962448.1
wCin2USA1 WP 006279657.1
wAra WP 006279657.1
wInc Cu WP 006279657.1
wMel WP 010962448.1
wUni WP 006279657.1
wIrr WP 006279657.1
wVitA WP 006279657.1
wYak WP 006279657.1
wAeae GV 2018 1 WP 010962448.1

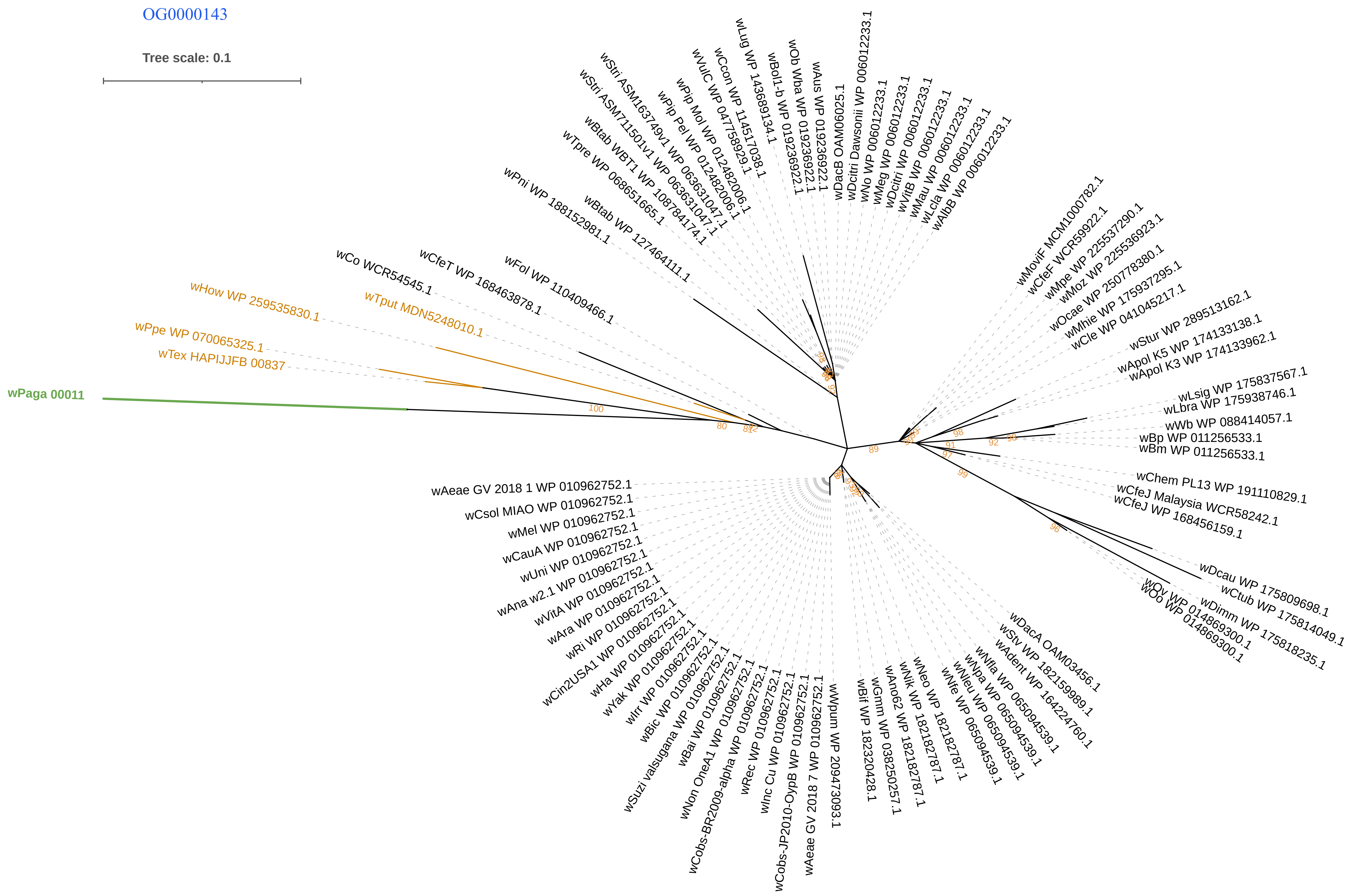
OG0000143
Tree scale: 0.1
wPaga 00011
wTex HAPIJJFB 00837
wPpe WP 070065325.1
wHow WP 259535830.1
wTput MDN5248010.1
wCo WCR54545.1
wCfeT WP 168463878.1
wFol WP 110409466.1
wPni WP 188152981.1
wBtab WP 127464111.1
wTpre WP 068651665.1
wBtab WBT1 WP 108784174.1
wStri ASM711501v1 WP 063631047.1
wStri ASM1637491v1 WP 063631047.1
wPip Pel WP 012482006.1
wPip Mol WP 012482006.1
wVulC WP 047758929.1
wCcon WP 114517038.1
wLug WP 143689134.1
wBol1-b WP 019236922.1
wOb Wba WP 019236922.1
wAus WP 019236922.1
wDacB OAM06025.1
wDcitri Dawsonii WP 006012233.1
wNo WP 006012233.1
wMeg WP 006012233.1
wDcitri WP 006012233.1
wVitB WP 006012233.1
wMau WP 006012233.1
wLcla WP 006012233.1
wAlbB WP 006012233.1
wMoviF MCM1000782.1
wCfeF WCR59922.1
wMpe WP 225537290.1
wMoz WP 225536923.1
wOcae WP 250778380.1
wMhie WP 175937295.1
wCle WP 041045217.1
wStur WP 289513162.1
wApol K5 WP 174133138.1
wApol K3 WP 174133962.1
wLsig WP 175837567.1
wLbra WP 175938746.1
wWb WP 088414057.1
wBp WP 011256533.1
wBm WP 011256533.1
wChem PL13 WP 191110829.1
wCfeJ Malaysia WCR58242.1
wCfeJ WP 168456159.1
wDcau WP 175809698.1
wCtub WP 175814049.1
wDimm WP 175818235.1
wOv WP 014869300.1
wOo WP 014869300.1
wDacA OAM03456.1
wStv WP 182159989.1
wAdent WP 164224760.1
wNfla WP 065094539.1
wNpa WP 065094539.1
wNleu WP 065094539.1
wNfe WP 065094539.1
wNeo WP 182182787.1
wNik WP 182182787.1
wAno62 WP 038250257.1
wGmm WP 038250257.1
wBif WP 182320428.1
wWpum WP 209473093.1
wAeae GV 2018 7 WP 010962752.1
wCobs-JP2010-OypB WP 010962752.1
wInc Cu WP 010962752.1
wRec WP 010962752.1
wCobs-BR2009-alpha WP 010962752.1
wNon OneA1 WP 010962752.1
wBai WP 010962752.1
wSuzi valsugana WP 010962752.1
wBic WP 010962752.1
wIrr WP 010962752.1
wYak WP 010962752.1
wHa WP 010962752.1
wCin2USA1 WP 010962752.1
wRi WP 010962752.1
wAra WP 010962752.1
wVitA WP 010962752.1
wAna w2.1 WP 010962752.1
wUni WP 010962752.1
wCauA WP 010962752.1
wMel WP 010962752.1
wCsol MIAO WP 010962752.1
wAeae GV 2018 1 WP 010962752.1

OG0000144

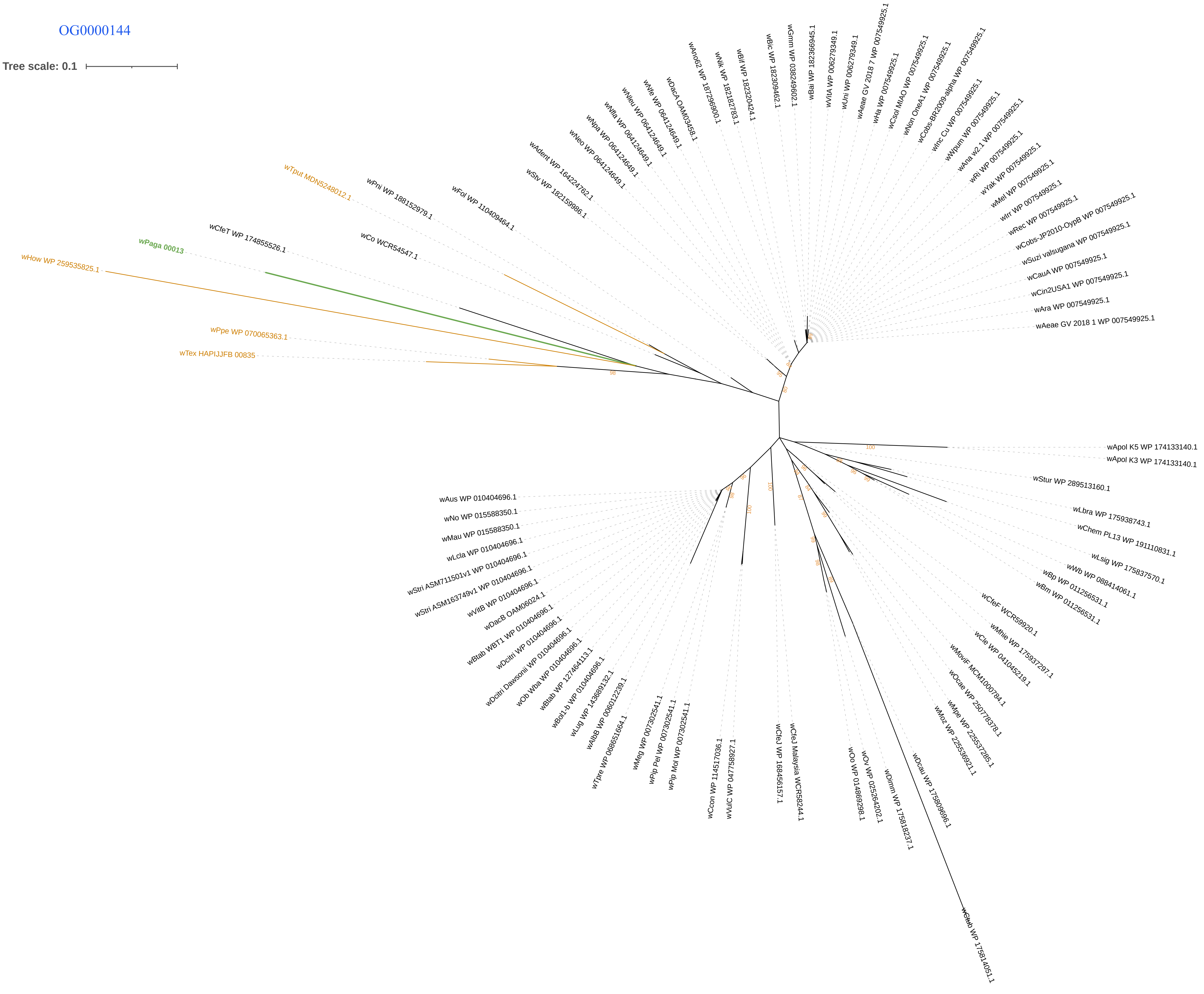

Tree scale: 0.1
wTput MDN5248012.1
wPni WP 188152979.1
wFol WP 110409464.1
wStv WP 182159986.1
wAdent WP 164224762.1
wNeo WP 064124649.1
wNpa WP 064124649.1
wNfla WP 064124649.1
wNleu WP 064124649.1
wNfe WP 064124649.1
wDacA OAM03458.1
wAno62 WP 187296900.1
wNik WP 182182783.1
wBif WP 182320424.1
wBic WP 182309462.1
wGmm WP 038249602.1
wBai WP 182366945.1
wWitA WP 006279349.1
wUni WP 006279349.1
wAeae GV 2018 7 WP 007549925.1
wHa WP 007549925.1
wCsol MIAO WP 007549925.1
wNon OneA1 WP 007549925.1
wCobs-BR2009-alpha WP 007549925.1
wInc Cu WP 007549925.1
wWpum WP 007549925.1
wAna w2.1 WP 007549925.1
wRi WP 007549925.1
wYak WP 007549925.1
wMel WP 007549925.1
wIrr WP 007549925.1
wRec WP 007549925.1
wCobs-JP2010-OypB WP 007549925.1
wSuzi valsugana WP 007549925.1
wCauA WP 007549925.1
wCin2USA1 WP 007549925.1
wAra WP 007549925.1
wAeae GV 2018 1 WP 007549925.1
wCfeT WP 174855526.1
wCo WCR54547.1
wPaga 00013
wHow WP 259535825.1
wPpe WP 070065363.1
wTex HAPIJJFB 00835
wApol K5 WP 174133140.1
wApol K3 WP 174133140.1
wStur WP 289513160.1
wLbra WP 175938743.1
wChem PL13 WP 191110831.1
wLsig WP 175837570.1
wWb WP 088414061.1
wBp WP 011256531.1
wBm WP 011256531.1
wCfeF WCR59920.1
wMhie WP 175937297.1
wCle WP 041045219.1
wMoviF MCM1000784.1
wOcae WP 250778378.1
wMpe WP 225537285.1
wMoz WP 225536921.1
wDcau WP 175809696.1
wCtub WP 175814051.1
wDimm WP 175818237.1
wOv WP 025264202.1
wOo WP 014869298.1
wCfeJ Malaysia WCR58244.1
wCfeJ WP 168456157.1
wVulC WP 047758927.1
wCcon WP 114517036.1
wPip Mol WP 007302541.1
wPip Pel WP 007302541.1
wMeg WP 007302541.1
wTpre WP 068651664.1
wAlbB WP 006012239.1
wLug WP 143689132.1
wBol1-b WP 010404696.1
wBtab WP 127464113.1
wOb Wba WP 010404696.1
wDcitri Dawsonii WP 010404696.1
wDcitri WP 010404696.1
wBtab WBT1 WP 010404696.1
wDacB OAM06024.1
wVitB WP 010404696.1
wStri ASM163749v1 WP 010404696.1
wStri ASM711501v1 WP 010404696.1
wLcla WP 010404696.1
wMau WP 015588350.1
wNo WP 015588350.1
wAus WP 010404696.1

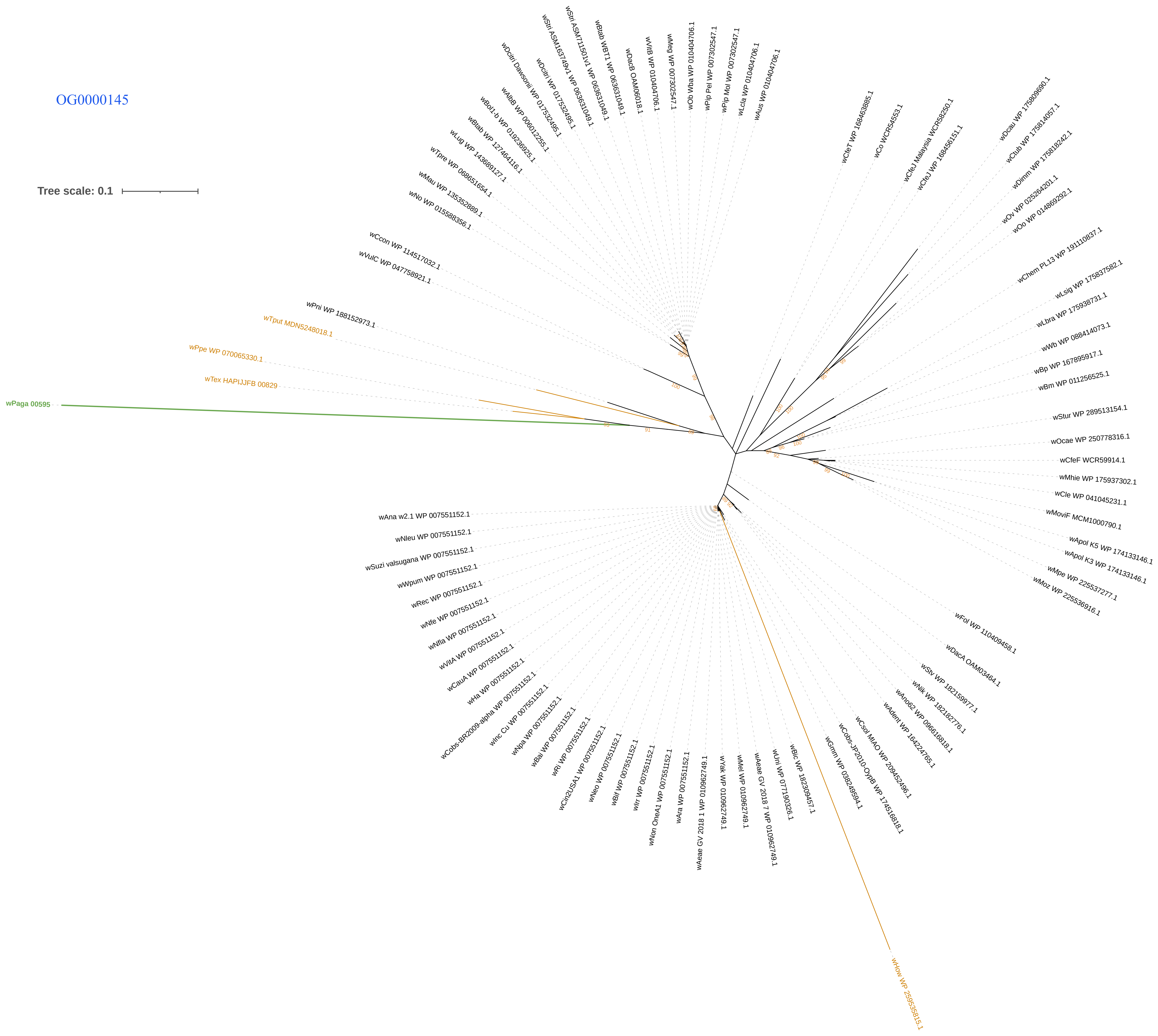
OG0000145
Tree scale: 0.1
wNo WP 015588356.1
wMau WP 135352889.1
wTpre WP 068651654.1
wLug WP 143689127.1
wBtab WP 127464116.1
wBol1-b WP 019236925.1
wAlbB WP 006012255.1
wDcitri Dawsonii WP 017532495.1
wDcitri WP 017532495.1
wStri ASM163749v1 WP 063631049.1
wStri ASM71150lv1 WP 063631049.1
wBtab WBT1 WP 063631049.1
wDacB OAM06018.1
wVitB WP 010404706.1
wMeg WP 007302547.1
wOb Wba WP 010404706.1
wPip Pel WP 007302547.1
wPip Mol WP 007302547.1
wLcla WP 010404706.1
wAus WP 010404706.1
wCcon WP 114517032.1
wVulC WP 047758921.1
wPni WP 188152973.1
wTput MDN5248018.1
wPpe WP 070065330.1
wTex HAPIJJFB 00829
wPaga 00595
wCfeT WP 168463885.1
wCo WCR54553.1
wCfeJ Malaysia WCR58250.1
wCfeJ WP 168456151.1
wDcau WP 175809690.1
wCtub WP 175814057.1
wDimm WP 175818242.1
wOv WP 025264201.1
wOo WP 014869292.1
wChem PL13 WP 191110837.1
wLsig WP 175837582.1
wLbra WP 175938731.1
wWb WP 088414073.1
wBp WP 167895917.1
wBm WP 011256525.1
wStur WP 289513154.1
wOcae WP 250778316.1
wCfeF WCR59914.1
wMhie WP 175937302.1
wCle WP 041045231.1
wMoviF MCM1000790.1
wApol K5 WP 174133146.1
wApol K3 WP 174133146.1
wMpe WP 225537277.1
wMoz WP 225536916.1
wFol WP 110409458.1
wDacA OAM03464.1
wStv WP 182159977.1
wNik WP 182182776.1
wAno62 WP 096616818.1
wAdent WP 164224765.1
wCsol MIAO WP 209452496.1
wCobs-JP2010-OypB WP 174516818.1
wGmm WP 038249594.1
wBic WP 182309457.1
wUni WP 077190326.1
wAeae GV 2018 7 WP 010962749.1
wMel WP 010962749.1
wYak WP 010962749.1
wAeae GV 2018 1 WP 010962749.1
wAra WP 007551152.1
wNon OneA1 WP 007551152.1
wIrr WP 007551152.1
wBif WP 007551152.1
wNeo WP 007551152.1
wCin2USA1 WP 007551152.1
wRi WP 007551152.1
wBai WP 007551152.1
wNpa WP 007551152.1
wInc Cu WP 007551152.1
wCobs-BR2009-alpha WP 007551152.1
wHa WP 007551152.1
wCauA WP 007551152.1
wVitA WP 007551152.1
wNfla WP 007551152.1
wNfe WP 007551152.1
wRec WP 007551152.1
wWpum WP 007551152.1
wSuzi valsugana WP 007551152.1
wNleu WP 007551152.1
wAna w2.1 WP 007551152.1
wHow WP 259535815.1

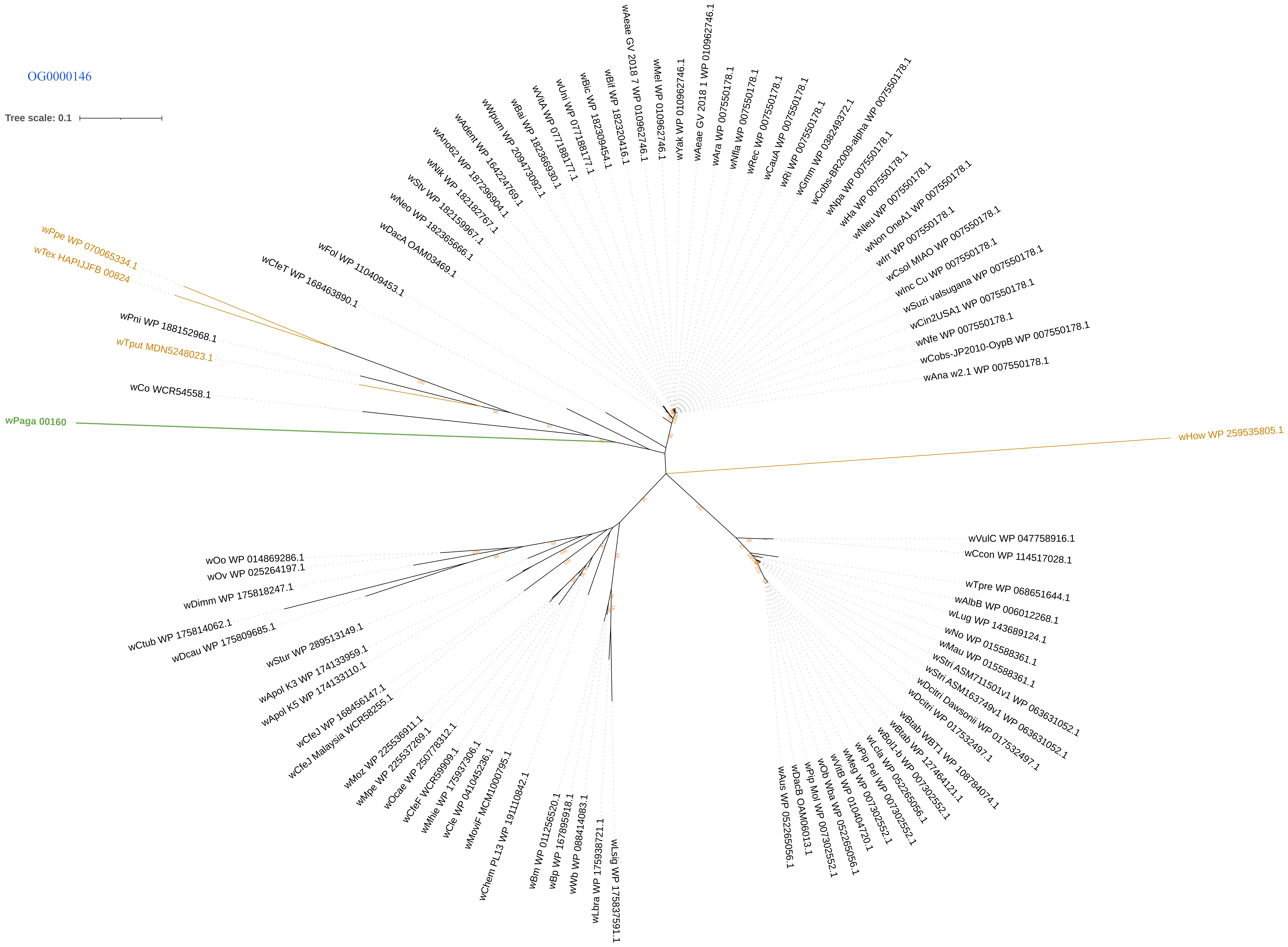
OG0000146
Tree scale: 0.1
wAeae GV 2018 7 WP 010962746.1
wMel WP 010962746.1
wYak WP 010962746.1
wAeae GV 2018 1 WP 010962746.1
wBif WP 182320416.1
wBic WP 182309454.1
wUni WP 077188177.1
wVitA WP 077188177.1
wBai WP 182366930.1
wWpum WP 209473092.1
wAdent WP 164224769.1
wAno62 WP 187296904.1
wNik WP 182182767.1
wStv WP 182159967.1
wNeo WP 182365666.1
wDacA OAM03469.1
wFol WP 110409453.1
wCfeT WP 168463890.1
wPpe WP 070065334.1
wTex HAPIJJFB 00824
wPni WP 188152968.1
wTput MDN5248023.1
wCo WCR54558.1
wPaga 00160
wAra WP 007550178.1
wNfla WP 007550178.1
wRec WP 007550178.1
wCauA WP 007550178.1
wRi WP 007550178.1
wGmm WP 038249372.1
wCobs-BR2009-alpha WP 007550178.1
wNpa WP 007550178.1
wHa WP 007550178.1
wNleu WP 007550178.1
wNon OneA1 WP 007550178.1
wIrr WP 007550178.1
wCsol MIAO WP 007550178.1
wInc Cu WP 007550178.1
wSuzi valsugana WP 007550178.1
wCin2USA1 WP 007550178.1
wNfe WP 007550178.1
wCobs-JP2010-OypB WP 007550178.1
wAna w2.1 WP 007550178.1
wHow WP 259535805.1
wVulC WP 047758916.1
wCcon WP 114517028.1
wTpre WP 068651644.1
wAlbB WP 006012268.1
wLug WP 143689124.1
wNo WP 015588361.1
wMau WP 015588361.1
wStri ASM711501v1 WP 063631052.1
wStri ASM163749v1 WP 063631052.1
wDcitri Dawsonii WP 017532497.1
wDcitri WP 017532497.1
wBtab WBT1 WP 108784074.1
wBtab WP 127464121.1
wBol1-b WP 007302552.1
wLcla WP 052265056.1
wPip Pel WP 007302552.1
wMeg WP 007302552.1
wVitB WP 010404720.1
wOb Wba WP 052265056.1
wPip Mol WP 007302552.1
wDacB OAM06013.1
wAus WP 052265056.1
wOo WP 014869286.1
wOv WP 025264197.1
wDimm WP 175818247.1
wCtub WP 175814062.1
wDcau WP 175809685.1
wStur WP 289513149.1
wApol K3 WP 174133959.1
wApol K5 WP 174133110.1
wCfeJ WP 168456147.1
wCfeJ Malaysia WCR58255.1
wMoz WP 225536911.1
wMpe WP 225537269.1
wOcae WP 250778312.1
wCfeF WCR59909.1
wMhie WP 175937306.1
wCle WP 041045236.1
wMoviF MCM1000795.1
wChem PL13 WP 191110842.1
wBm WP 011256520.1
wBp WP 167895918.1
wWb WP 088414083.1
wLbra WP 175938721.1
wLsig WP 175837591.1

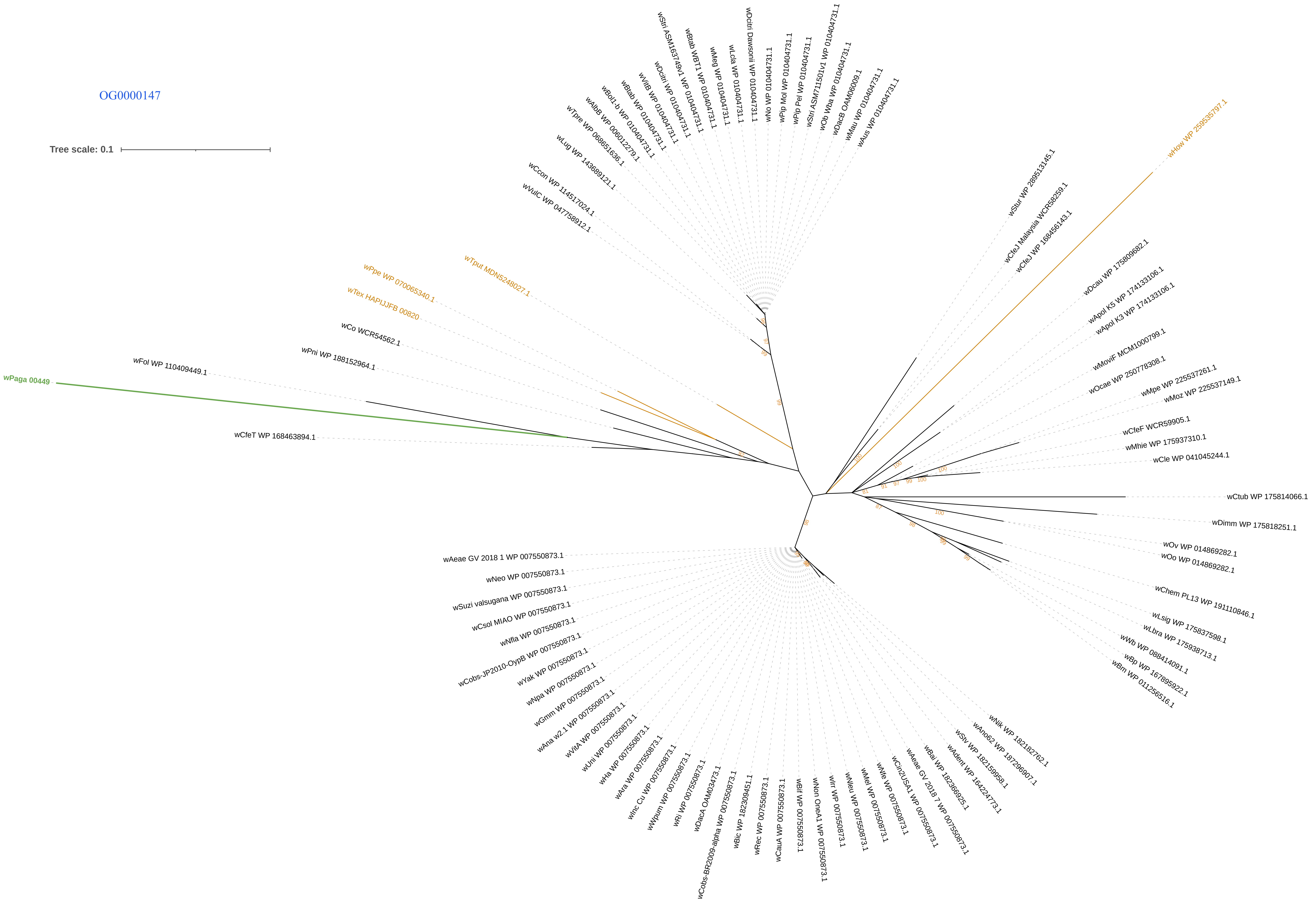

OG0000147
Tree scale: 0.1
wPaga 00449
wFol WP 110409449.1
wCfeT WP 168463894.1
wPni WP 188152964.1
wCo WCR54562.1
wTex HAPIJJFB 00820
wPpe WP 070065340.1
wTput MDN5248027.1
wVulC WP 047758912.1
wCcon WP 114517024.1
wLug WP 143689121.1
wTpre WP 068651636.1
wAlbB WP 006012279.1
wBol1-b WP 010404731.1
wBtab WP 010404731.1
wVitB WP 010404731.1
wDcitri WP 010404731.1
wStri ASM163749v1 WP 010404731.1
wBtab WBT1 WP 010404731.1
wMeg WP 010404731.1
wLcla WP 010404731.1
wDcitri Dawsonii WP 010404731.1
wNo WP 010404731.1
wPip Mol WP 010404731.1
wPip Pel WP 010404731.1
wStri ASM711501v1 WP 010404731.1
wOb Wba WP 010404731.1
wDacB OAM06009.1
wMau WP 010404731.1
wAus WP 010404731.1
wHow WP 259535797.1
wStur WP 289513145.1
wCfeJ Malaysia WCR58259.1
wCfeJ WP 168456143.1
wDcau WP 175809682.1
wApol K5 WP 174133106.1
wApol K3 WP 174133106.1
wMoviF MCM1000799.1
wOcae WP 250778308.1
wMpe WP 225537261.1
wMoz WP 225537149.1
wCfeF WCR59905.1
wMhie WP 175937310.1
wCle WP 041045244.1
wCtub WP 175814066.1
wDimm WP 175818251.1
wOv WP 014869282.1
wOo WP 014869282.1
wChem PL13 WP 191110846.1
wLsig WP 175837598.1
wLbra WP 175938713.1
wWb WP 088414091.1
wBp WP 167895922.1
wBm WP 011256516.1
wNik WP 182182762.1
wAno62 WP 187296907.1
wStv WP 182159958.1
wAdent WP 164224773.1
wBai WP 182366925.1
wAeae GV 2018 7 WP 007550873.1
wCin2USA1 WP 007550873.1
wNfe WP 007550873.1
wMel WP 007550873.1
wNleu WP 007550873.1
wIrr WP 007550873.1
wNon OneA1 WP 007550873.1
wBif WP 007550873.1
wCauA WP 007550873.1
wRec WP 007550873.1
wBic WP 182309451.1
wCobs-BR2009-alpha WP 007550873.1
wDacA OAM03473.1
wRi WP 007550873.1
wWpum WP 007550873.1
wInc Cu WP 007550873.1
wAra WP 007550873.1
wHa WP 007550873.1
wUni WP 007550873.1
wVitA WP 007550873.1
wAna w2.1 WP 007550873.1
wGmm WP 007550873.1
wNpa WP 007550873.1
wYak WP 007550873.1
wCobs-JP2010-OypB WP 007550873.1
wNfla WP 007550873.1
wCsol MIAO WP 007550873.1
wSuzi valsugana WP 007550873.1
wNeo WP 007550873.1
wAeae GV 2018 1 WP 007550873.1

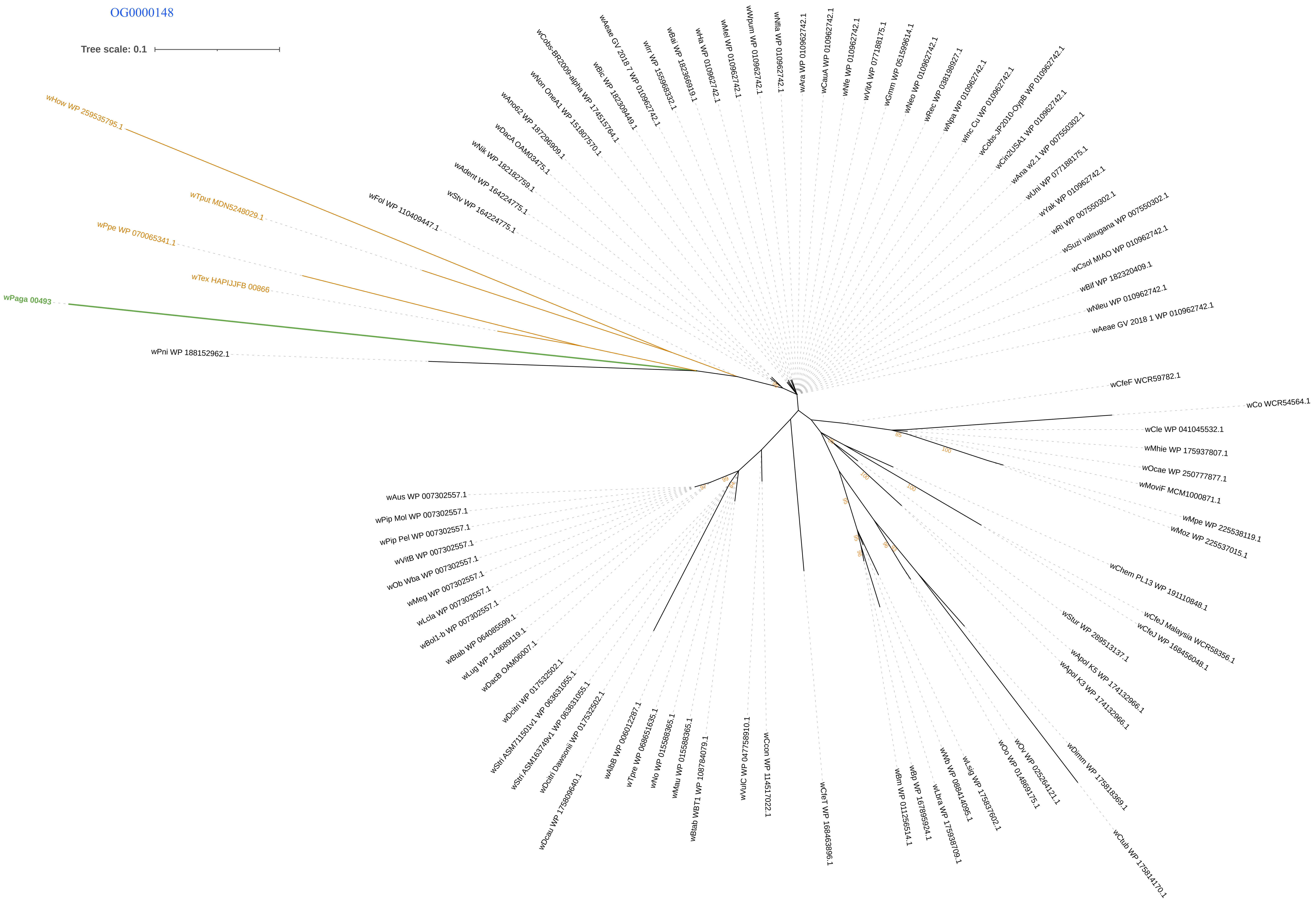

OG0000148
Tree scale: 0.1
wHow WP 259535795.1
wTput MDN5248029.1
wPpe WP 070065341.1
wTex HAPIJJFB 00866
wPaga 00493
wPni WP 188152962.1
wFol WP 110409447.1
wStv WP 164224775.1
wAdent WP 164224775.1
wNik WP 182182759.1
wDacA OAM03475.1
wAno62 WP 187296909.1
wNon OneA1 WP 151807570.1
wCobs-BR2009-alpha WP 174515764.1
wBic WP 182309449.1
wAeae GV 2018 7 WP 010962742.1
wIrr WP 155968332.1
wBai WP 182366919.1
wHa WP 010962742.1
wMel WP 010962742.1
wWpum WP 010962742.1
wNfla WP 010962742.1
wAra WP 010962742.1
wCauA WP 010962742.1
wNfe WP 010962742.1
wVitA WP 077188175.1
wGmm WP 051599614.1
wNeo WP 010962742.1
wRec WP 038198927.1
wNpa WP 010962742.1
wInc Cu WP 010962742.1
wCobs-JP2010-OypB WP 010962742.1
wCin2USA1 WP 010962742.1
wAna w2.1 WP 007550302.1
wUni WP 077188175.1
wYak WP 010962742.1
wRi WP 007550302.1
wSuzi valsugana WP 007550302.1
wCsol MIAO WP 010962742.1
wBif WP 182320409.1
wNleu WP 010962742.1
wAeae GV 2018 1 WP 010962742.1
wCfeF WCR59782.1
wCo WCR54564.1
wCle WP 041045532.1
wMhie WP 175937807.1
wOcae WP 250777877.1
wMoviF MCM1000871.1
wMpe WP 225538119.1
wMoz WP 225537015.1
wChem PL13 WP 191110848.1
wCfeJ Malaysia WCR58356.1
wCfeJ WP 168456048.1
wStur WP 289513137.1
wApol K5 WP 174132966.1
wApol K3 WP 174132966.1
wDimm WP 175818369.1
wCtub WP 175814170.1
wOv WP 025264121.1
wOo WP 014869175.1
wLsig WP 175837602.1
wWb WP 088414095.1
wLbra WP 175938709.1
wBp WP 167895924.1
wBm WP 011256514.1
wCfeT WP 168463896.1
wCcon WP 114517022.1
wVulC WP 047758910.1
wBtab WBT1 WP 108784079.1
wMau WP 015588365.1
wNo WP 015588365.1
wTpre WP 068651635.1
wAlbB WP 006012287.1
wDcau WP 175809640.1
wDcitri Dawsonii WP 017532502.1
wStri ASM163749v1 WP 063631055.1
wStri ASM711501v1 WP 063631055.1
wDcitri WP 017532502.1
wDacB OAM06007.1
wLug WP 143689119.1
wBtab WP 064085599.1
wBol1-b WP 007302557.1
wLcla WP 007302557.1
wMeg WP 007302557.1
wOb Wba WP 007302557.1
wVitB WP 007302557.1
wPip Pel WP 007302557.1
wPip Mol WP 007302557.1
wAus WP 007302557.1

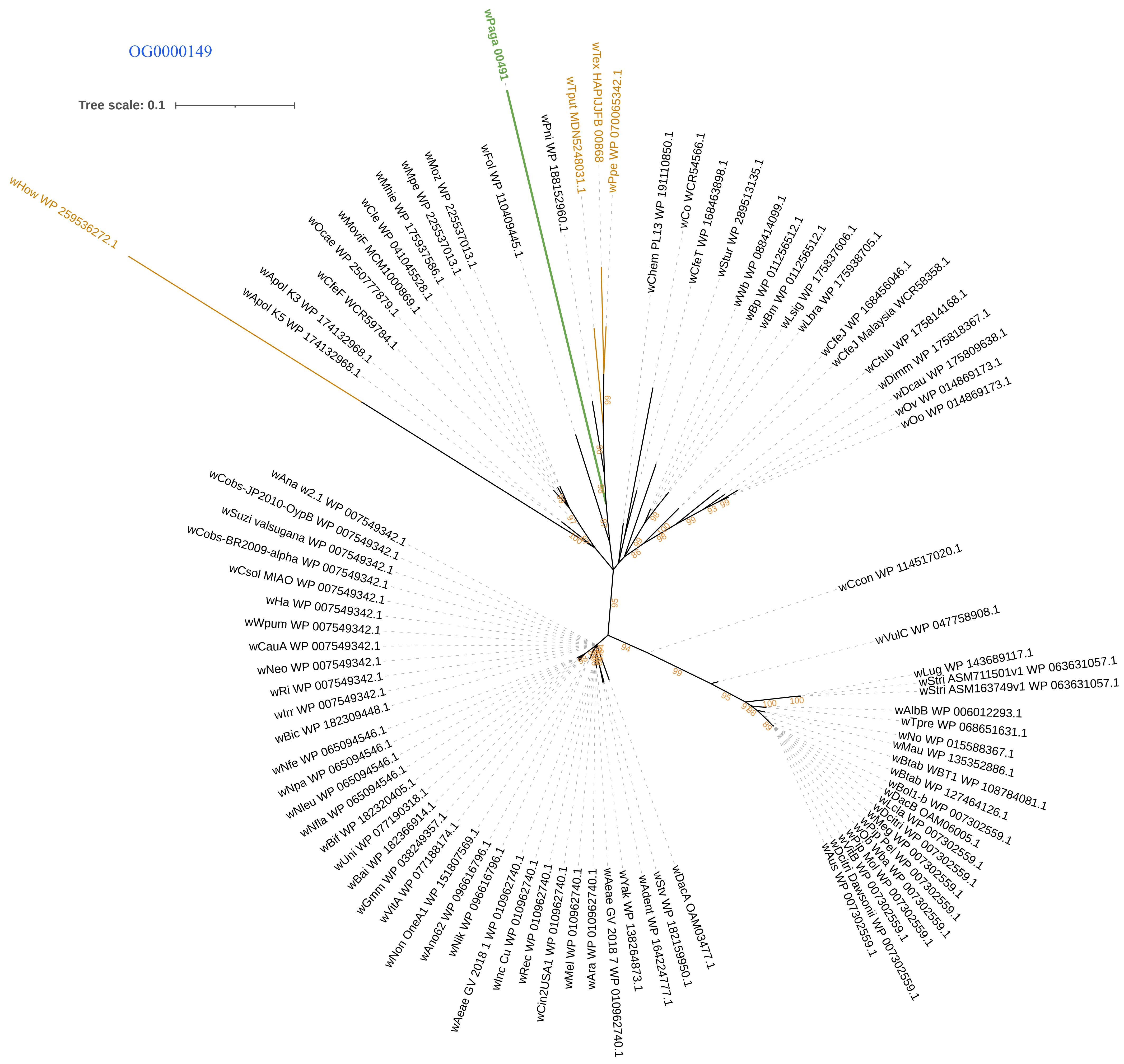

OG0000149
Tree scale: 0.1
wPaga 00491
wTex HAPIJJFB 00868
wTput MDN5248031.1
wPpe WP 070065342.1
wPni WP 188152960.1
wFol WP 110409445.1
wMoz WP 225537013.1
wMpe WP 225537013.1
wMhie WP 175937586.1
wCle WP 041045528.1
wMoviF MCM1000869.1
wOcae WP 250777879.1
wCfeF WCR59784.1
wApol K3 WP 174132968.1
wApol K5 WP 174132968.1
wHow WP 259536272.1
wChem PL13 WP 191110850.1
wCo WCR54566.1
wCfeT WP 168463898.1
wStur WP 289513135.1
wWb WP 088414099.1
wBp WP 011256512.1
wBm WP 011256512.1
wLsig WP 175837606.1
wLbra WP 175938705.1
wCfeJ WP 168456046.1
wCfeJ Malaysia WCR58358.1
wCtub WP 175814168.1
wDimm WP 175818367.1
wDcau WP 175809638.1
wOv WP 014869173.1
wOo WP 014869173.1
wAna w2.1 WP 007549342.1
wCobs-JP2010-OypB WP 007549342.1
wSuzi valsugana WP 007549342.1
wCobs-BR2009-alpha WP 007549342.1
wCsol MIAO WP 007549342.1
wHa WP 007549342.1
wWpum WP 007549342.1
wCauA WP 007549342.1
wNeo WP 007549342.1
wRi WP 007549342.1
wIrr WP 007549342.1
wBic WP 182309448.1
wNfe WP 065094546.1
wNpa WP 065094546.1
wNleu WP 065094546.1
wNfla WP 065094546.1
wBif WP 182320405.1
wUni WP 077190318.1
wBai WP 182366914.1
wGmm WP 038249357.1
wVitA WP 077188174.1
wNon OneA1 WP 151807569.1
wAno62 WP 096616796.1
wNik WP 096616796.1
wAeae GV 2018 1 WP 010962740.1
wInc Cu WP 010962740.1
wRec WP 010962740.1
wCin2USA1 WP 010962740.1
wMel WP 010962740.1
wAra WP 010962740.1
wAeae GV 2018 7 WP 010962740.1
wYak WP 138264873.1
wAdent WP 164224777.1
wStv WP 182159950.1
wDacA OAM03477.1
wCcon WP 114517020.1
wVulC WP 047758908.1
wLug WP 143689117.1
wStri ASM711501v1 WP 063631057.1
wStri ASM163749v1 WP 063631057.1
wAlbB WP 006012293.1
wTpre WP 068651631.1
wNo WP 015588367.1
wMau WP 135352886.1
wBtab WBT1 WP 108784081.1
wBtab WP 127464126.1
wBol1-b WP 007302559.1
wDacB OAM06005.1
wLcla WP 007302559.1
wDcitri WP 007302559.1
wMeg WP 007302559.1
wPip Pel WP 007302559.1
wOb WP 007302559.1
wPip Mol WP 007302559.1
wVitB WP 007302559.1
wDcitri Dawsoni WP 007302559.1
wAus WP 007302559.1

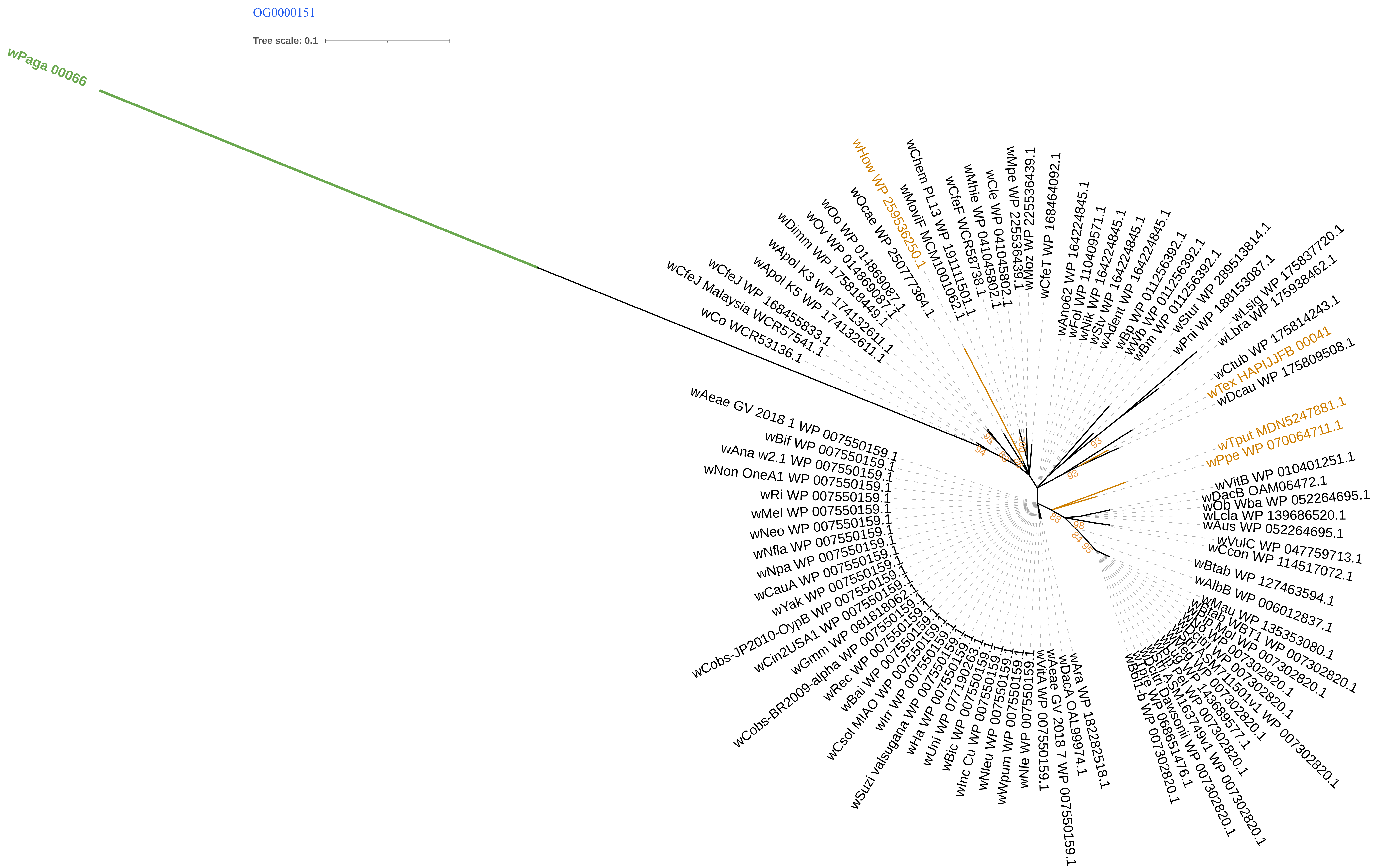
OG0000151
Tree scale: 0.1
wPaga 00066
wHow WP 259536250.1
wChem PL13 WP 191111501.1
wMoviF MCM1001062.1
wOcae WP 250777364.1
wCfeF WCR58738.1
wMhie WP 041045802.1
wCle WP 041045802.1
wMpe WP 225536439.1
wMoz WP 225536439.1
wCfeT WP 168464092.1
wOo WP 014869087.1
wOv WP 014869087.1
wDimm WP 175818449.1
wApol K3 WP 174132611.1
wApol K5 WP 174132611.1
wCfeJ WP 168455833.1
wCfeJ Malaysia WCR57541.1
wCo WCR53136.1
wAno62 WP 164224845.1
wFol WP 110409571.1
wNik WP 164224845.1
wStv WP 164224845.1
wAdent WP 164224845.1
wBp WP 011256392.1
wWb WP 011256392.1
wBm WP 011256392.1
wStur WP 289513814.1
wPni WP 188153087.1
wLsig WP 175837720.1
wLbra WP 175938462.1
wCtub WP 175814243.1
wTex HAPIJJFB 00041
wDcau WP 175809508.1
wTput MDN5247881.1
wPpe WP 070064711.1
wVitB WP 010401251.1
wDacB OAM06472.1
wOb Wba WP 052264695.1
wLcla WP 139686520.1
wAus WP 052264695.1
wVulC WP 047759713.1
wCcon WP 114517072.1
wBtab WP 127463594.1
wAlbB WP 006012837.1
wMau WP 135353080.1
wAeae GV 2018 1 WP 007550159.1
wBif WP 007550159.1
wAna w2.1 WP 007550159.1
wNon OneA1 WP 007550159.1
wRi WP 007550159.1
wMel WP 007550159.1
wNeo WP 007550159.1
wNfla WP 007550159.1
wNpa WP 007550159.1
wCauA WP 007550159.1
wYak WP 007550159.1
wCobs-JP2010-OypB WP 007550159.1
wCin2USA1 WP 007550159.1
wGmm WP 081818062.1
wCobs-BR2009-alpha WP 007550159.1
wRec WP 007550159.1
wBai WP 007550159.1
wCsol MIAO WP 007550159.1
wIrr WP 007550159.1
wSuzi valsugana WP 007550159.1
wHa WP 007550159.1
wUni WP 077190263.1
wBic WP 007550159.1
wInc Cu WP 007550159.1
wNleu WP 007550159.1
wMpum WP 007550159.1
wNfe WP 007550159.1
wVitA WP 007550159.1
wAeae GV 2018 7 WP 007550159.1
wDacA OAL99974.1
wAra WP 182282518.1
wBol1-b WP 007302820.1
93
94
88
93
93
88
98
84
95

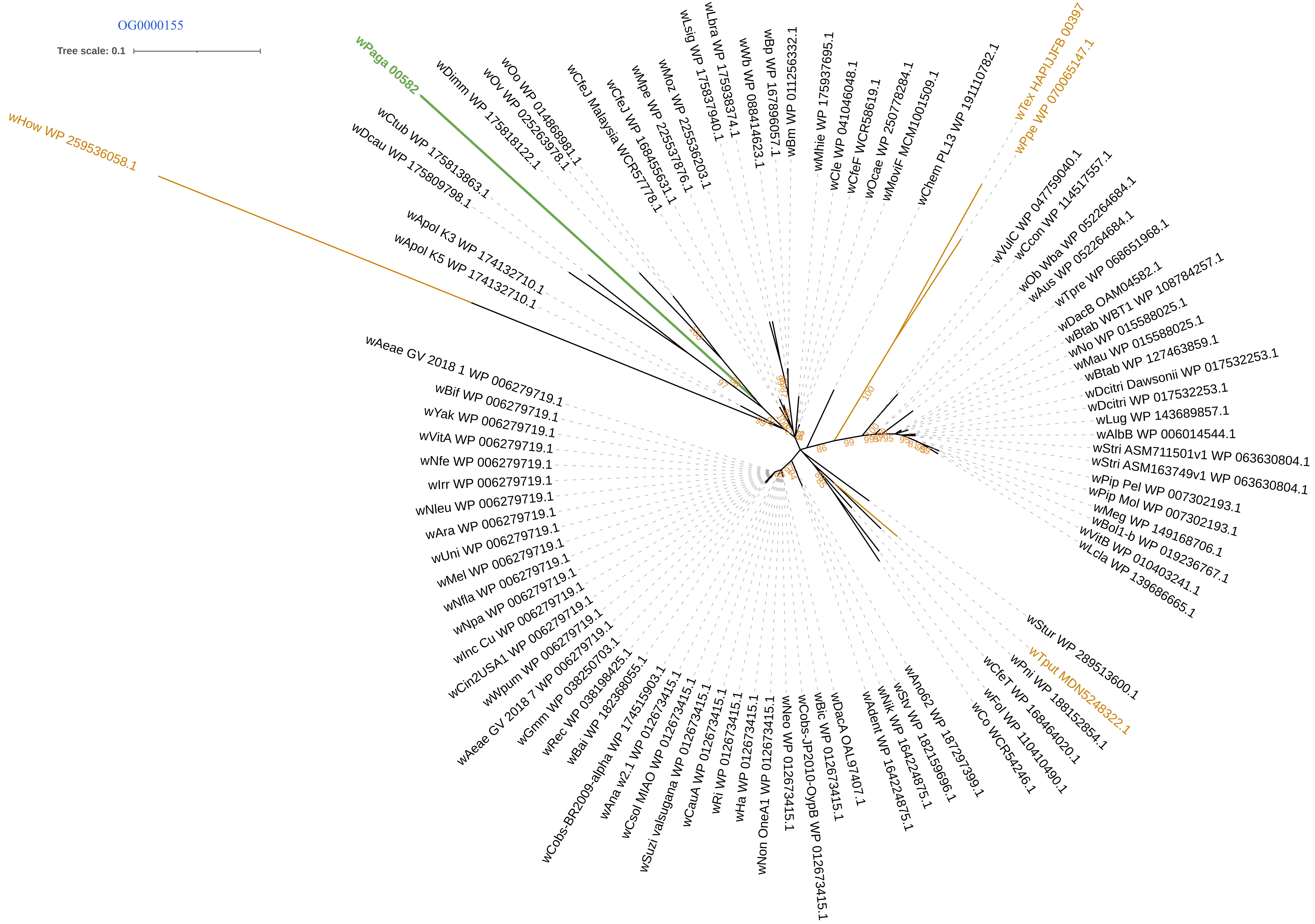
OG0000155
Tree scale: 0.1
wHow WP 259536058.1
wPaga 00582
wCtub WP 175813863.1
wDcau WP 175809798.1
wDimm WP 175818122.1
wOo WP 014868981.1
wOv WP 025263978.1
wCfeJ Malaysia WCR57778.1
wCfeJ WP 168455631.1
wMpe WP 225537876.1
wMoz WP 225536203.1
wLsig WP 175837940.1
wLbra WP 175938374.1
wWb WP 088414623.1
wBp WP 167896057.1
wBm WP 011256332.1
wMhie WP 175937695.1
wCle WP 041046048.1
wCfeF WCR58619.1
wOcae WP 250778284.1
wMoviF MCM1001509.1
wChem PL13 WP 191110782.1
wTex HAPIJJFB 00397
wPpe WP 070065147.1
wVulC WP 047759040.1
wCcon WP 114517557.1
wOb Wba WP 052264684.1
wAus WP 052264684.1
wTpre WP 068651968.1
wDacB OAM04582.1
wBtab WBT1 WP 108784257.1
wNo WP 015588025.1
wMau WP 015588025.1
wBtab WP 127463859.1
wDcitri Dawsonii WP 017532253.1
wDcitri WP 017532253.1
wLug WP 143689857.1
wAlbB WP 006014544.1
wStri ASM711501v1 WP 063630804.1
wStri ASM163749v1 WP 063630804.1
wPip Pel WP 007302193.1
wPip Mol WP 007302193.1
wMeg WP 149168706.1
wBol1-b WP 019236767.1
wVitB WP 010403241.1
wLcla WP 139686665.1
wStur WP 289513600.1
wTput MDN5248322.1
wPni WP 188152854.1
wCfeT WP 168464020.1
wFol WP 110410490.1
wCo WCR54246.1
wAno62 WP 187297399.1
wStv WP 182159696.1
wNik WP 164224875.1
wAdent WP 164224875.1
wDacA OAL97407.1
wBic WP 012673415.1
wCobs-JP2010-OypB WP 012673415.1
wNeo WP 012673415.1
wNon OneA1 WP 012673415.1
wHa WP 012673415.1
wRi WP 012673415.1
wCauA WP 012673415.1
wSuzi valsugana WP 012673415.1
wCsol MIAO WP 012673415.1
wAna w2.1 WP 012673415.1
wCobs-BR2009-alpha WP 174515903.1
wBai WP 182368055.1
wRec WP 038198425.1
wGmm WP 038250703.1
wAeae GV 2018 7 WP 006279719.1
wNpum WP 006279719.1
wCin2USA1 WP 006279719.1
wInc Cu WP 006279719.1
wNpa WP 006279719.1
wNfla WP 006279719.1
wMel WP 006279719.1
wUni WP 006279719.1
wAra WP 006279719.1
wNleu WP 006279719.1
wIrr WP 006279719.1
wNfe WP 006279719.1
wVitA WP 006279719.1
wYak WP 006279719.1
wBif WP 006279719.1
wAeae GV 2018 1 WP 006279719.1
wApol K5 WP 174132710.1
wApol K3 WP 174132710.1

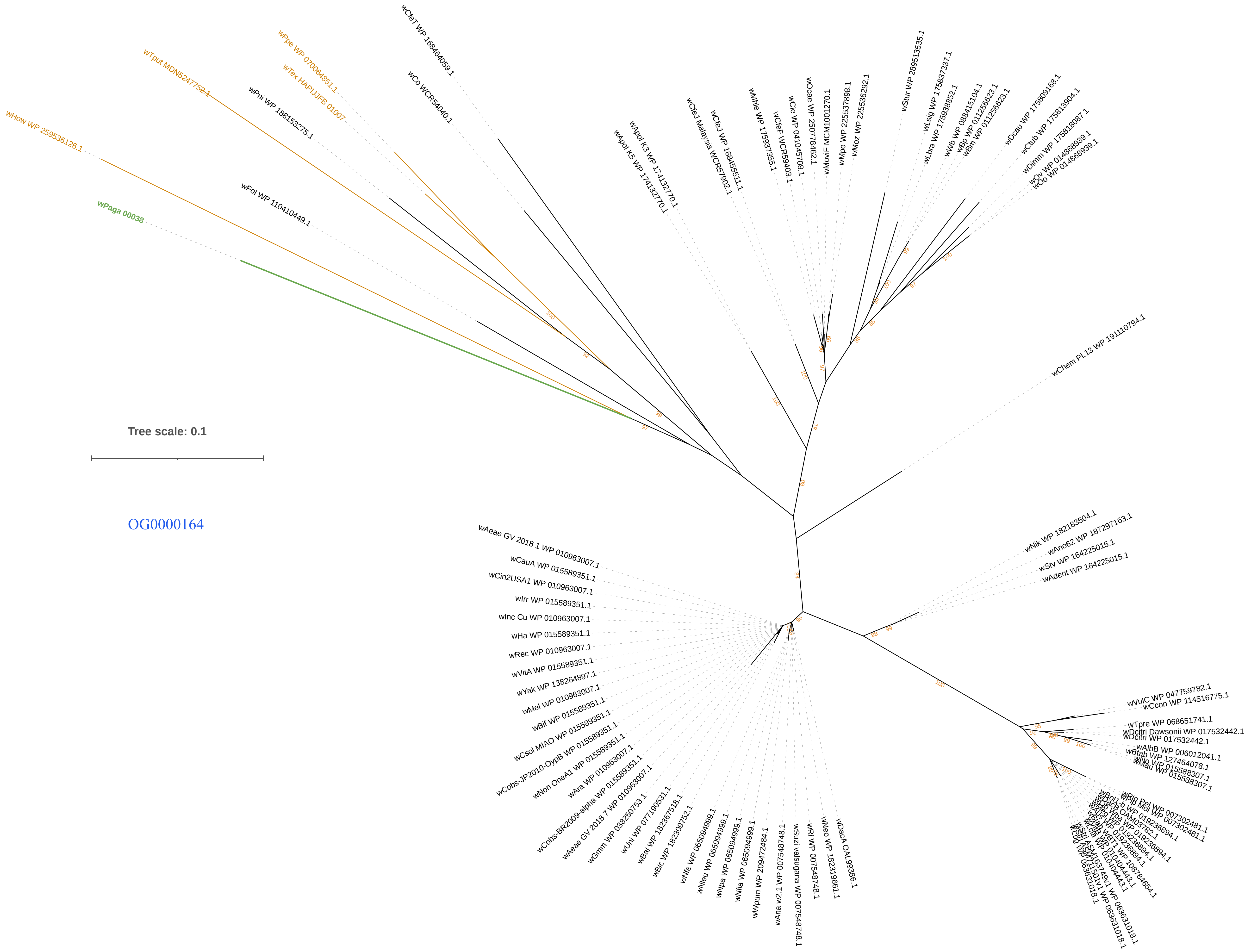
wCfeT WP 168464059.1
wPpe WP 070064851.1
wTput MDN5247752.1
wTex HAPIJJFB 01007
wCo WCR54040.1
wPni WP 188153275.1
wHow WP 259536126.1
wFol WP 110410449.1
wPaga 00038
wApol K3 WP 174132770.1
wApol K5 WP 174132770.1
wCfeJ Malaysia WCR57902.1
wCfeJ WP 168455511.1
wMhie WP 175937355.1
wCfeF WCR59403.1
wCle WP 041045708.1
wOcae WP 250778462.1
wMoviF MCM1001270.1
wMpe WP 225537898.1
wMoz WP 225536292.1
wStur WP 289513535.1
wLsig WP 175837337.1
wLbra WP 175938852.1
wWb WP 088415104.1
wBp WP 011256623.1
wBm WP 011256623.1
wDcau WP 175809168.1
wCtub WP 175813904.1
wDimm WP 175818087.1
wOv WP 014868939.1
wOo WP 014868939.1
wChem PL13 WP 191110794.1
Tree scale: 0.1
OG0000164
wNik WP 182183504.1
wAno62 WP 187297163.1
wStv WP 164225015.1
wAdent WP 164225015.1
wAeae GV 2018 1 WP 010963007.1
wCauA WP 015589351.1
wCin2USA1 WP 010963007.1
wIrr WP 015589351.1
wInc Cu WP 010963007.1
wHa WP 015589351.1
wRec WP 010963007.1
wVitA WP 015589351.1
wYak WP 138264897.1
wMel WP 010963007.1
wBif WP 015589351.1
wCsol MIAO WP 015589351.1
wCobs-JP2010-OypB WP 015589351.1
wNon OneA1 WP 015589351.1
wAra WP 010963007.1
wCobs-BR2009-alpha WP 015589351.1
wAeae GV 2018 7 WP 010963007.1
wGmm WP 038250753.1
wUni WP 077190531.1
wBai WP 182367518.1
wBic WP 182309752.1
wNfe WP 065094999.1
wNleu WP 065094999.1
wNpa WP 065094999.1
wNfla WP 065094999.1
wWpum WP 209472484.1
wAna w2.1 WP 007548748.1
wSuzi valsugana WP 007548748.1
wRi WP 007548748.1
wNeo WP 182319661.1
wDacA OAL99386.1
wVulC WP 047759782.1
wCcon WP 114516775.1
wTpre WP 068651741.1
wDcitri Dawsonii WP 017532442.1
wDcitri WP 017532442.1
wAlbB WP 006012041.1
wBrab WP 127464078.1
wNo WP 015588307.1
wMau WP 015588307.1
wPip Pel WP 007302481.1
wPip Mol WP 007302481.1

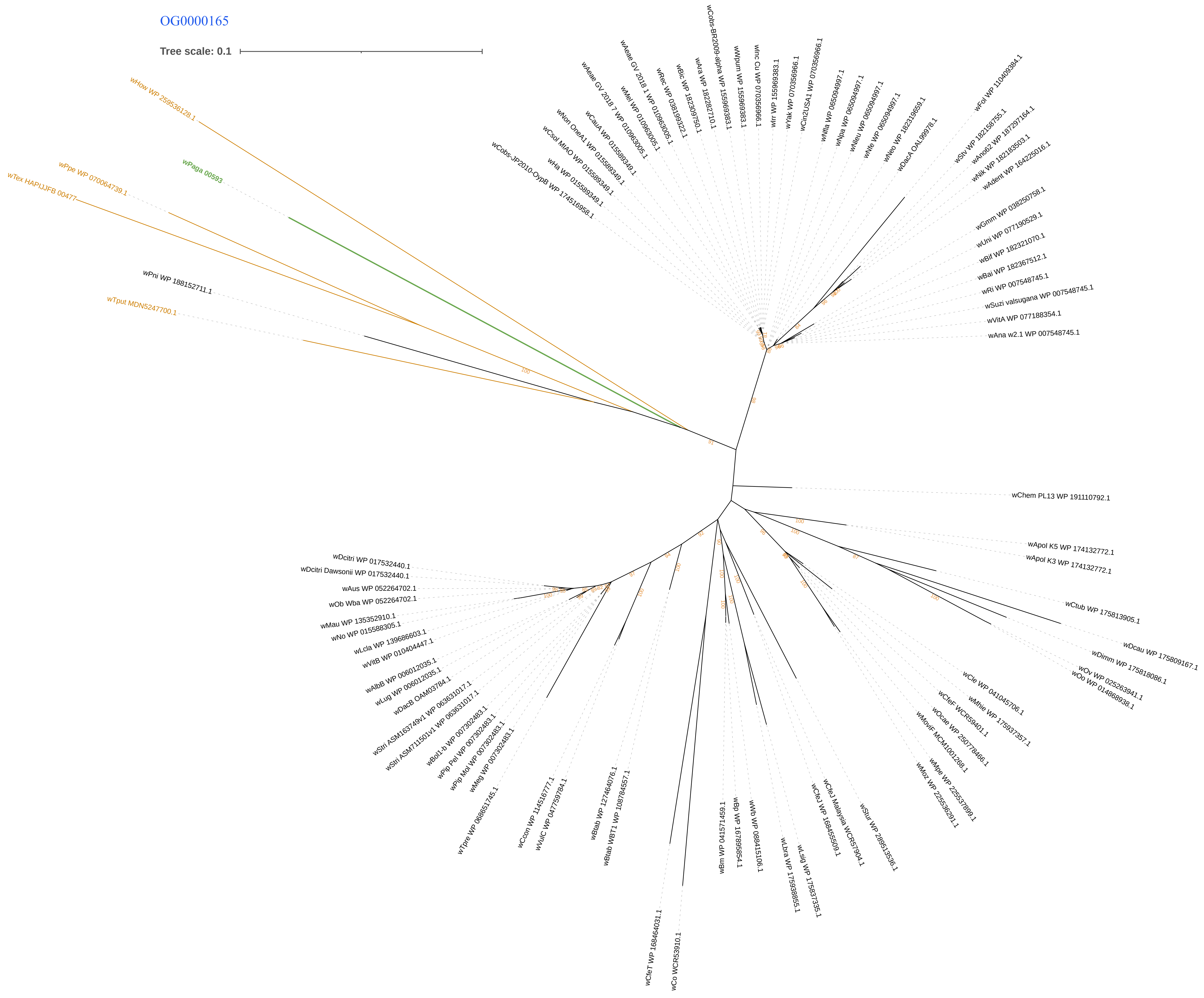
OG0000165
Tree scale: 0.1
wHow WP 259536128.1
wPpe WP 070064739.1
wPaga 00593
wTex HAPIJJFB 00477
wPni WP 188152711.1
wTput MDN5247700.1
wCobs-JP2010-OypB WP 174516958.1
wHa WP 015589349.1
wCsol MIAO WP 015589349.1
wNon OneA1 WP 015589349.1
wCauA WP 015589349.1
wAeae GV 2018 7 WP 010963005.1
wMel WP 010963005.1
wAeae GV 2018 1 WP 010963005.1
wRec WP 038199322.1
wBic WP 182309750.1
wAra WP 182282710.1
wCobs-BR2009-alpha WP 155969383.1
wWpum WP 155969383.1
wInc Cu WP 070356966.1
wIrr WP 155969383.1
wYak WP 070356966.1
wCin2USA1 WP 070356966.1
wNfla WP 065094997.1
wNpa WP 065094997.1
wNleu WP 065094997.1
wNfe WP 065094997.1
wNeo WP 182219659.1
wDacA OAL99978.1
wFol WP 110409384.1
wStv WP 182158755.1
wAno62 WP 187297164.1
wNlk WP 182183503.1
wAdent WP 164225016.1
wGmm WP 038250758.1
wUni WP 077190529.1
wBif WP 182321070.1
wBai WP 182367512.1
wRi WP 007548745.1
wSuzi valsugana WP 007548745.1
wVitA WP 077188354.1
wAna w2.1 WP 007548745.1
wChem PL13 WP 191110792.1
wApol K5 WP 174132772.1
wApol K3 WP 174132772.1
wCtub WP 175813905.1
wDcau WP 175809167.1
wDimm WP 175818086.1
wOv WP 025263941.1
wOo WP 014868938.1
wCle WP 041045706.1
wMhie WP 175937357.1
wCfeF WCR59401.1
wOcae WP 250778466.1
wMovif MCM1001268.1
wMpe WP 225537899.1
wMoz WP 225536291.1
wCfeJ Malaysia WCR57904.1
wCfeJ WP 168455509.1
wStur WP 289513536.1
wLsig WP 175837335.1
wLbra WP 175938855.1
wWb WP 088415106.1
wBp WP 167895854.1
wBm WP 041571459.1
wCfeT WP 168464031.1
wCo WCR53910.1
wBtab WBT1 WP 108784557.1
wBtab WP 127464076.1
wVulC WP 047759784.1
wCcon WP 114516777.1
wTpre WP 068651745.1
wMeg WP 007302483.1
wPip Mol WP 007302483.1
wPip Pel WP 007302483.1
wBol1-b WP 007302483.1
wStri ASM711501v1 WP 063631017.1
wStri ASM163749v1 WP 063631017.1
wDacB OAM03784.1
wLug WP 006012035.1
wAlbB WP 006012035.1
wVitB WP 010404447.1
wLcla WP 139686603.1
wNo WP 015588305.1
wMau WP 135352910.1
wOb Wba WP 052264702.1
wAus WP 052264702.1
wDcitri Dawsonii WP 017532440.1
wDcitri WP 017532440.1

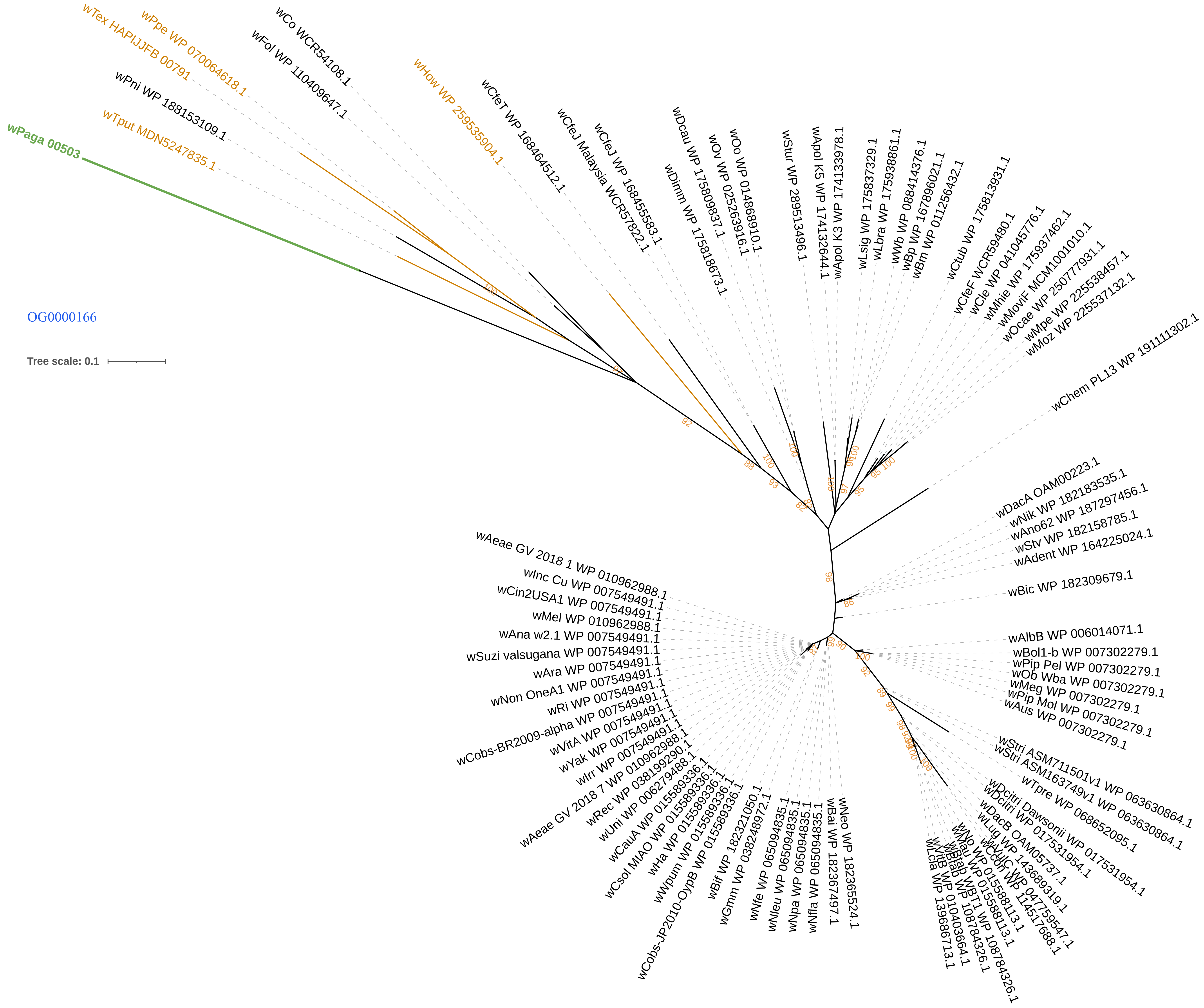

OG0000166
Tree scale: 0.1
wPaga 00503
wTput MDN5247835.1
wPni WP 188153109.1
wTex HAPIJJFB 00791
wPpe WP 070064618.1
wFol WP 110409647.1
wCo WCR54108.1
wHow WP 259535904.1
wCfeT WP 168464512.1
wCfeJ Malaysia WCR57822.1
wCfeJ WP 168455583.1
wDimm WP 175818673.1
wDcau WP 175809837.1
wOv WP 025263916.1
wOo WP 014868910.1
wStur WP 289513496.1
wApol K5 WP 174132644.1
wApol K3 WP 174133978.1
wLsig WP 175837329.1
wLbra WP 175938861.1
wWb WP 088414376.1
wBp WP 167896021.1
wBm WP 011256432.1
wCtub WP 175813931.1
wCfeF WCR59480.1
wCle WP 041045776.1
wMhie WP 175937462.1
wMoviF MCM1001010.1
wOcae WP 250777931.1
wMpe WP 225538457.1
wMoz WP 225537132.1
wChem PL13 WP 191111302.1
wDacA OAM00223.1
wNik WP 182183535.1
wAno62 WP 187297456.1
wStv WP 182158785.1
wAdent WP 164225024.1
wBic WP 182309679.1
wAlbB WP 006014071.1
wBol1-b WP 007302279.1
wPip Pel WP 007302279.1
wOb Wba WP 007302279.1
wMeg WP 007302279.1
wPip Mol WP 007302279.1
wAus WP 007302279.1
wStri ASM711501v1 WP 063630864.1
wStri ASM163749v1 WP 063630864.1
wTpre WP 068652095.1
wDcitri Dawsonii WP 017531954.1
wDcitri WP 017531954.1
wDacB OAM05737.1
wLug WP 143689319.1
wVulC WP 047759547.1
wCcon WP 114517688.1
wNo WP 015588113.1
wMau WP 015588113.1
wBtab WBT1 WP 108784326.1
wBtab WP 108784326.1
wVitB WP 010403664.1
wLcla WP 139686713.1
wNeo WP 182365524.1
wBai WP 182367497.1
wNfla WP 065094835.1
wNpa WP 065094835.1
wNleu WP 065094835.1
wNfe WP 065094835.1
wGmm WP 038248972.1
wBif WP 182321050.1
wCobs-JP2010-OypB WP 015589336.1
wWpum WP 015589336.1
wHa WP 015589336.1
wCsol MIAO WP 015589336.1
wCauA WP 015589336.1
wUni WP 006279488.1
wRec WP 038199290.1
wAeae GV 2018 7 WP 010962988.1
wIrr WP 007549491.1
wYak WP 007549491.1
wVitA WP 007549491.1
wCobs-BR2009-alpha WP 007549491.1
wRi WP 007549491.1
wNon OneA1 WP 007549491.1
wAra WP 007549491.1
wSuzi valsugana WP 007549491.1
wAna w2.1 WP 007549491.1
wMel WP 010962988.1
wCin2USA1 WP 007549491.1
wInc Cu WP 007549491.1
wAeae GV 2018 1 WP 010962988.1

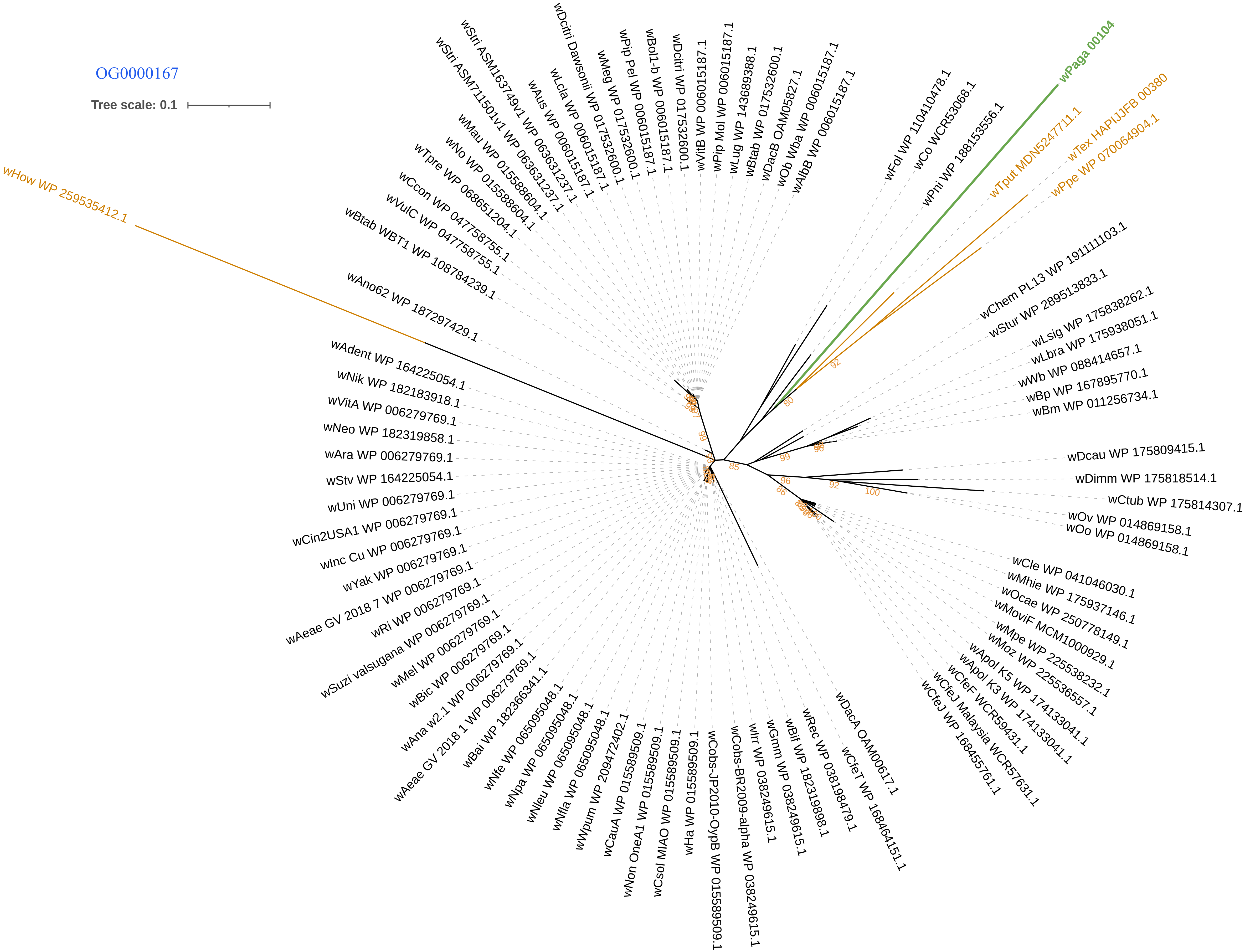
OG0000167
Tree scale: 0.1
wHow WP 259535412.1
wAno62 WP 187297429.1
wBtab WBT1 WP 108784239.1
wVulC WP 047758755.1
wCcon WP 047758755.1
wTpre WP 068651204.1
wNo WP 015588604.1
wMau WP 015588604.1
wStri ASM711501v1 WP 063631237.1
wStri ASM163749v1 WP 063631237.1
wAus WP 006015187.1
wLcla WP 006015187.1
wDcitri Dawsonii WP 017532600.1
wMeg WP 017532600.1
wPip Pel WP 006015187.1
wBol1-b WP 006015187.1
wDcitri WP 017532600.1
wVitB WP 006015187.1
wPip Mol WP 006015187.1
wLug WP 143689388.1
wBtab WP 017532600.1
wDacB OAM05827.1
wOb Wba WP 006015187.1
wAlbB WP 006015187.1
wFol WP 110410478.1
wCo WCR53068.1
wPni WP 188153556.1
wPaga 00104
wTput MDN5247711.1
wTex HAPIJJFB 00380
wPpe WP 070064904.1
wChem PL13 WP 191111103.1
wStur WP 289513833.1
wLsig WP 175838262.1
wLbra WP 175938051.1
wWb WP 088414657.1
wBp WP 167895770.1
wBm WP 011256734.1
wDcau WP 175809415.1
wDimm WP 175818514.1
wCtub WP 175814307.1
wOv WP 014869158.1
wOo WP 014869158.1
wCle WP 041046030.1
wMhie WP 175937146.1
wOcae WP 250778149.1
wMoviF MCM1000929.1
wMpe WP 225538232.1
wMoz WP 225536557.1
wApol K5 WP 174133041.1
wApol K3 WP 174133041.1
wCfeF WCR59431.1
wCfeJ Malaysia WCR57631.1
wCfeJ WP 168455761.1
wDacA OAM00617.1
wCfeT WP 168464151.1
wRec WP 038198479.1
wBif WP 182319898.1
wGmm WP 038249615.1
wIrr WP 038249615.1
wCobs-BR2009-alpha WP 038249615.1
wCobs-JP2010-OypB WP 015589509.1
wHa WP 015589509.1
wCsol MIAO WP 015589509.1
wNon OneA1 WP 015589509.1
wCauA WP 209472402.1
wWpum WP 065095048.1
wNfla WP 065095048.1
wNleu WP 065095048.1
wNpa WP 065095048.1
wNfe WP 065095048.1
wBai WP 182366341.1
wAeae GV 2018 1 WP 006279769.1
wAna w2.1 WP 006279769.1
wBic WP 006279769.1
wMel WP 006279769.1
wSuzi valsugana WP 006279769.1
wRi WP 006279769.1
wAeae GV 2018 7 WP 006279769.1
wYak WP 006279769.1
wInc Cu WP 006279769.1
wCin2USA1 WP 006279769.1
wUni WP 006279769.1
wStv WP 164225054.1
wAra WP 006279769.1
wNeo WP 182319858.1
wVitA WP 006279769.1
wNik WP 182183918.1
wAdent WP 164225054.1

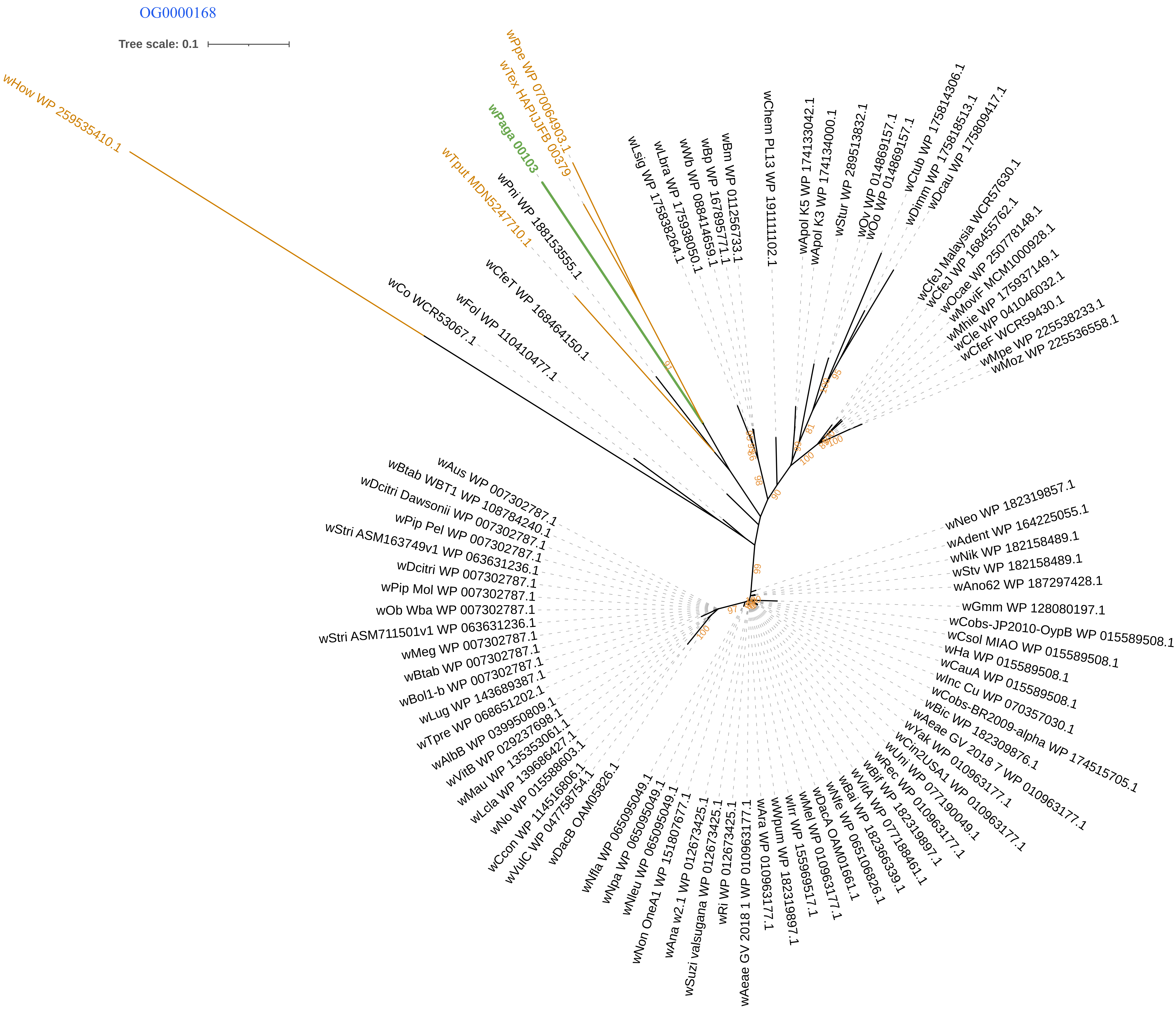
OG0000168
Tree scale: 0.1
wHow WP 259535410.1
wPpe WP 070064903.1
wTex HAPIJJFB 00379
wPaga 00103
wTput MDN5247710.1
wPni WP 188153555.1
wCfeT WP 168464150.1
wCo WCR53067.1
wFol WP 110410477.1
wLsig WP 175838264.1
wLbra WP 175938050.1
wWb WP 088414659.1
wBp WP 167895771.1
wBm WP 011256733.1
wChem PL13 WP 191111102.1
wApol K5 WP 174133042.1
wApol K3 WP 174134000.1
wStur WP 289513832.1
wOv WP 014869157.1
wOo WP 014869157.1
wCtub WP 175814306.1
wDimm WP 175818513.1
wDcau WP 175809417.1
wCfeJ Malaysia WCR57630.1
wCfeJ WP 168455762.1
wOcae WP 250778148.1
wMoviF MCM1000928.1
wMhie WP 175937149.1
wCle WP 041046032.1
wCfeF WCR59430.1
wMpe WP 225538233.1
wMoz WP 225536558.1
wNeo WP 182319857.1
wAdent WP 164225055.1
wNik WP 182158489.1
wStv WP 182158489.1
wAno62 WP 187297428.1
wGmm WP 128080197.1
wCobs-JP2010-OypB WP 015589508.1
wCsol MIAO WP 015589508.1
wHa WP 015589508.1
wCauA WP 015589508.1
wInc Cu WP 070357030.1
wCobs-BR2009-alpha WP 174515705.1
wBic WP 182309876.1
wAeae GV 2018 7 WP 010963177.1
wYak WP 010963177.1
wCin2USA1 WP 010963177.1
wUni WP 077190049.1
wRec WP 010963177.1
wBif WP 182319897.1
wVitA WP 077188461.1
wBai WP 182366339.1
wNfe WP 065106826.1
wDacA OAM01661.1
wMel WP 010963177.1
wIrr WP 155969517.1
wWpum WP 182319897.1
wAra WP 010963177.1
wAeae GV 2018 1 WP 010963177.1
wRi WP 012673425.1
wSuzi valsugana WP 012673425.1
wAna w2.1 WP 012673425.1
wNon OneA1 WP 151807677.1
wNleu WP 065095049.1
wNpa WP 065095049.1
wNfla WP 065095049.1
wDacB OAM05826.1
wVulC WP 047758754.1
wCcon WP 114516806.1
wNo WP 015588603.1
wLcla WP 139686427.1
wMau WP 135353061.1
wVitB WP 029237698.1
wAlbB WP 039950809.1
wTpre WP 068651202.1
wLug WP 143689387.1
wBol1-b WP 007302787.1
wBtab WP 007302787.1
wMeg WP 007302787.1
wStri ASM711501v1 WP 063631236.1
wOb Wba WP 007302787.1
wPip Mol WP 007302787.1
wDcitri WP 007302787.1
wStri ASM163749v1 WP 063631236.1
wPip Pel WP 007302787.1
wDcitri Dawsonii WP 007302787.1
wBtab WBT1 WP 108784240.1
wAus WP 007302787.1

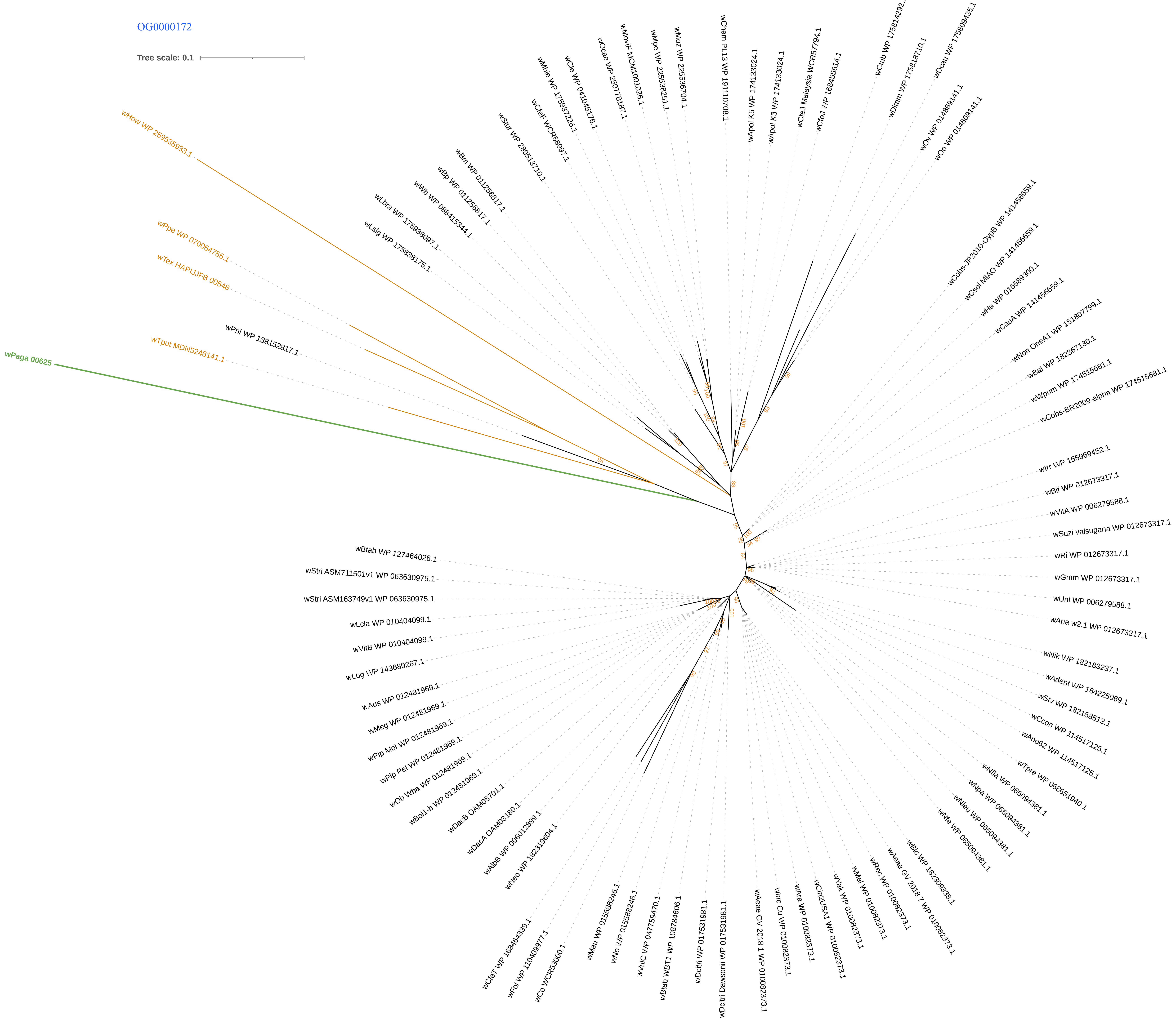
OG0000172
Tree scale: 0.1
wHow WP 259535933.1
wPpe WP 070064756.1
wTex HAPIJJFB 00548
wPni WP 188152817.1
wTput MDN5248141.1
wPaga 00625
wLsig WP 175838175.1
wLbra WP 175938097.1
wWb WP 088415344.1
wBp WP 011256817.1
wBm WP 011256817.1
wStur WP 289513710.1
wCfeF WCR58997.1
wMhie WP 175937226.1
wCle WP 041045176.1
wOcae WP 250778187.1
wMoviF MCM1001026.1
wMpe WP 225538251.1
wMoz WP 225536704.1
wChem PL13 WP 191110708.1
wApol K5 WP 174133024.1
wApol K3 WP 174133024.1
wCfeJ Malaysia WCR57794.1
wCfeJ WP 168455614.1
wCtub WP 175814292.1
wDimm WP 175818710.1
wDcau WP 175809435.1
wOv WP 014869141.1
wOo WP 014869141.1
wCobs-JP2010-OypB WP 141456659.1
wCsol MIAO WP 141456659.1
wHa WP 015589300.1
wCauA WP 141456659.1
wNon OneA1 WP 151807799.1
wBai WP 182367130.1
wWpum WP 174515681.1
wCobs-BR2009-alpha WP 174515681.1
wIrr WP 155969452.1
wBif WP 012673317.1
wVitA WP 006279588.1
wSuzi valsugana WP 012673317.1
wRi WP 012673317.1
wGmm WP 012673317.1
wUni WP 006279588.1
wAna w2.1 WP 012673317.1
wNik WP 182183237.1
wAdent WP 164225069.1
wStv WP 182158512.1
wCcon WP 114517125.1
wAno62 WP 114517125.1
wTpre WP 068651940.1
wNfla WP 065094381.1
wNpa WP 065094381.1
wNleu WP 065094381.1
wNfe WP 065094381.1
wBic WP 182309338.1
wAeae GV 2018 7 WP 010082373.1
wRec WP 010082373.1
wMel WP 010082373.1
wYak WP 010082373.1
wCin2USA1 WP 010082373.1
wAra WP 010082373.1
wInc Cu WP 010082373.1
wAeae GV 2018 1 WP 010082373.1
wDcitri Dawsonii WP 017531981.1
wDcitri WP 017531981.1
wBtab WBT1 WP 108784606.1
wVulC WP 047759470.1
wNo WP 015588246.1
wMau WP 015588246.1
wCo WCR53000.1
wFol WP 110409977.1
wCfeT WP 168464339.1
wNeo WP 182319604.1
wAlbB WP 006012899.1
wDacA OAM03180.1
wDacB OAM05701.1
wBol1-b WP 012481969.1
wOb Wba WP 012481969.1
wPip Pel WP 012481969.1
wPip Mol WP 012481969.1
wMeg WP 012481969.1
wAus WP 012481969.1
wLug WP 143689267.1
wVitB WP 010404099.1
wLcla WP 010404099.1
wStri ASM163749v1 WP 063630975.1
wStri ASM711501v1 WP 063630975.1
wBtab WP 127464026.1

OG0000177

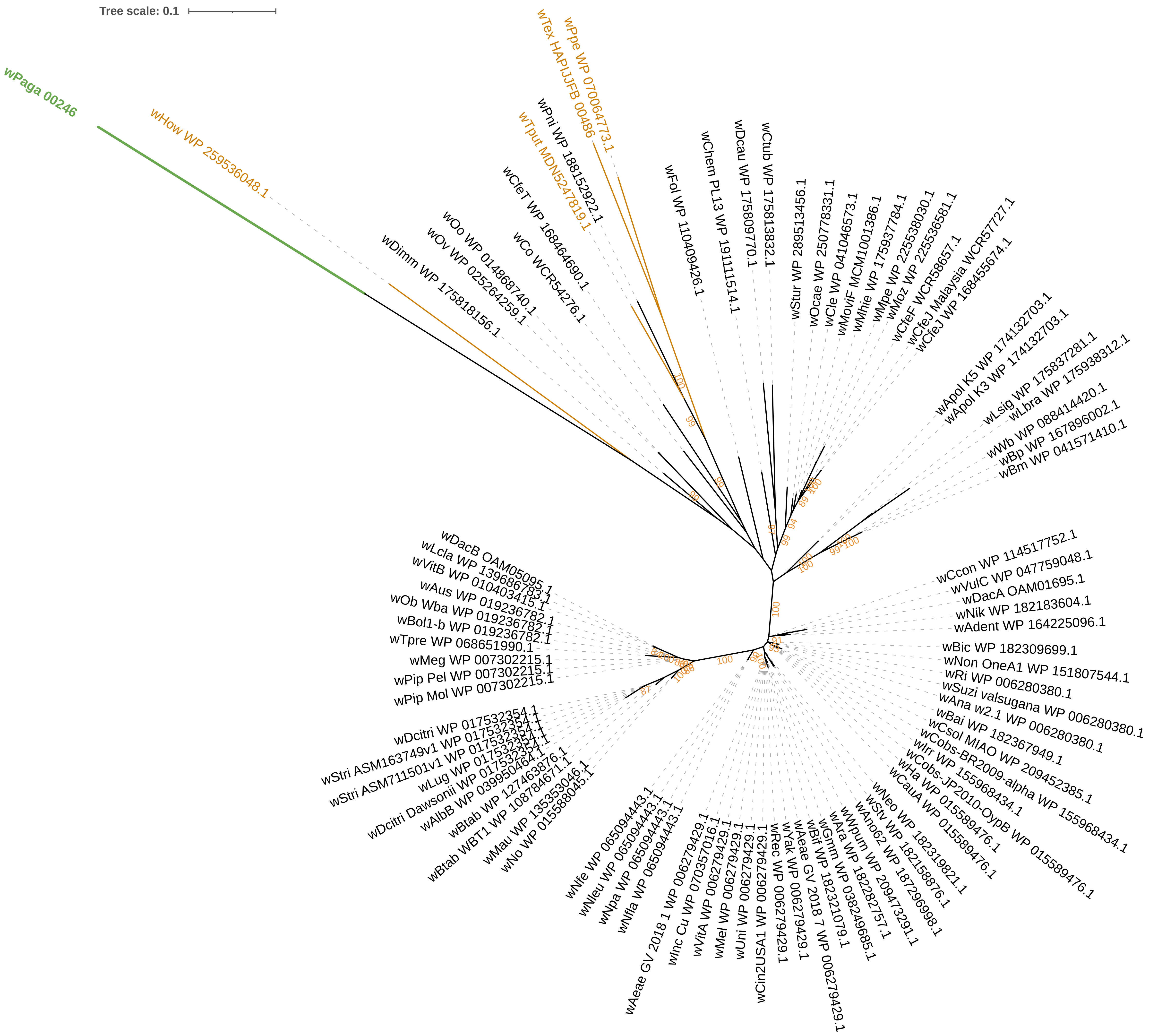
Tree scale: 0.1
wPaga 00246
wHow WP 259536048.1
wTex HAPIJJFB 00486
wPpe WP 070064773.1
wTput MDN5247819.1
wPni WP 188152922.1
wCfeT WP 168464690.1
wCo WCR54276.1
wOo WP 014868740.1
wOv WP 025264259.1
wDimm WP 175818156.1
wFol WP 110409426.1
wChem PL13 WP 191111514.1
wDcau WP 175809770.1
wCtub WP 175813832.1
wStur WP 289513456.1
wOcae WP 250778331.1
wCle WP 041046573.1
wMoviF MCM1001386.1
wMhie WP 175937784.1
wMpe WP 225538030.1
wMoz WP 225536581.1
wCfeF WCR58657.1
wCfeJ Malaysia WCR57727.1
wCfeJ WP 168455674.1
wApol K5 WP 174132703.1
wApol K3 WP 174132703.1
wLsig WP 175837281.1
wLbra WP 175938312.1
wWb WP 088414420.1
wBp WP 167896002.1
wBm WP 041571410.1
wCcon WP 114517752.1
wVulC WP 047759048.1
wDacA OAM01695.1
wNik WP 182183604.1
wAdent WP 164225096.1
wBic WP 182309699.1
wNon OneA1 WP 151807544.1
wRi WP 006280380.1
wSuzi valsugana WP 006280380.1
wAna w2.1 WP 006280380.1
wBai WP 182367949.1
wCsol MIAO WP 209452385.1
wCobs-BR2009-alpha WP 155968434.1
wIrr WP 155968434.1
wCobs-JP2010-OypB WP 015589476.1
wHa WP 015589476.1
wCauA WP 015589476.1
wNeo WP 182319821.1
wStv WP 182158876.1
wAno62 WP 187296998.1
wWpum WP 209473291.1
wAra WP 182282757.1
wGmm WP 038249685.1
wBif WP 182321079.1
wAeae GV 2018 7 WP 006279429.1
wYak WP 006279429.1
wRec WP 006279429.1
wCin2USA1 WP 006279429.1
wUni WP 006279429.1
wMel WP 006279429.1
wVitA WP 006279429.1
wInc Cu WP 070357016.1
wAeae GV 2018 1 WP 006279429.1
wNfla WP 065094443.1
wNpa WP 065094443.1
wNleu WP 065094443.1
wNfe WP 065094443.1
wNo WP 015580045.1
wMau WP 135353046.1
wBtab WBT1 WP 108784671.1
wBtab WP 127463876.1
wAlbB WP 039950464.1
wDcitri Dawsonii WP 017532354.1
wLug WP 017532354.1
wStri ASM711501v1 WP 017532354.1
wStri ASM163749v1 WP 017532354.1
wDcitri WP 017532354.1
wPip Mol WP 007302215.1
wPip Pel WP 007302215.1
wMeg WP 007302215.1
wTpre WP 068651990.1
wBol1-b WP 019236782.1
wOb Wba WP 019236782.1
wAus WP 019236782.1
wVitB WP 010403415.1
wLcla WP 139686783.1
wDacB OAM05095.1

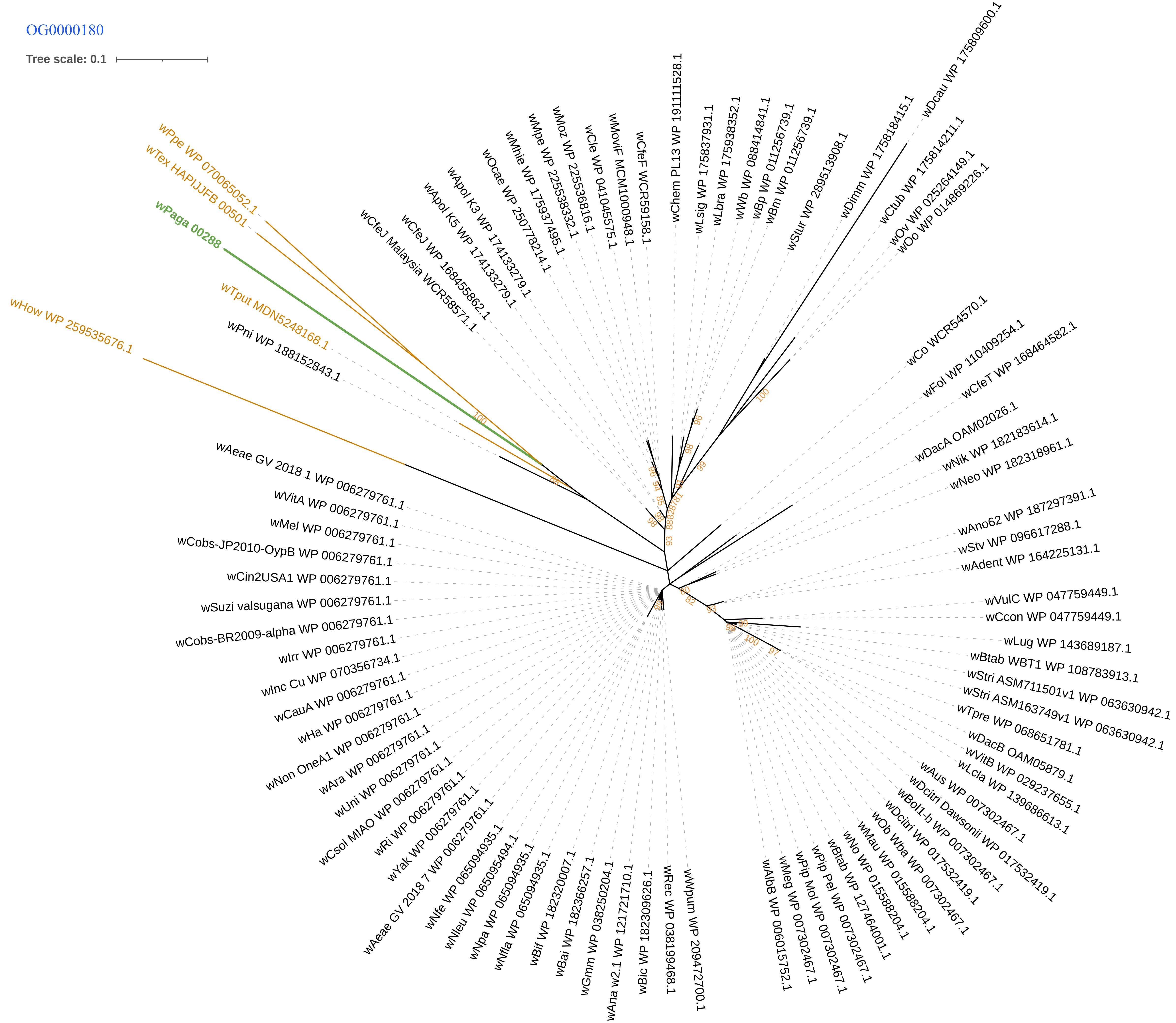
OG0000180
Tree scale: 0.1
wPpe WP 070065052.1
wTex HAPIJJFB 00501
wPaga 00288
wTput MDN5248168.1
wHow WP 259535676.1
wPni WP 188152843.1
wCfeJ Malaysia WCR58571.1
wCfeJ WP 168455862.1
wApol K5 WP 174133279.1
wApol K3 WP 174133279.1
wOcae WP 250778214.1
wMhie WP 175937495.1
wMpe WP 225538332.1
wMoz WP 225536816.1
wCle WP 041045575.1
wMoviF MCM1000948.1
wCfeF WCR59158.1
wChem PL13 WP 191111528.1
wLsig WP 175837931.1
wLbra WP 175938352.1
wWb WP 088414841.1
wBp WP 011256739.1
wBm WP 011256739.1
wStur WP 289513908.1
wDimm WP 175818415.1
wDcau WP 175809600.1
wCtub WP 175814211.1
wOv WP 025264149.1
wOo WP 014869226.1
wCo WCR54570.1
wFol WP 110409254.1
wCfeT WP 168464582.1
wDacA OAM02026.1
wNik WP 182183614.1
wNeo WP 182318961.1
wAno62 WP 187297391.1
wStv WP 096617288.1
wAdent WP 164225131.1
wVulC WP 047759449.1
wCcon WP 047759449.1
wLug WP 143689187.1
wBtab WBT1 WP 108783913.1
wStri ASM711501v1 WP 063630942.1
wStri ASM163749v1 WP 063630942.1
wTpre WP 068651781.1
wDacB OAM05879.1
wVitB WP 029237655.1
wLcla WP 139686613.1
wAus WP 007302467.1
wDcitri Dawsonii WP 017532419.1
wBol1-b WP 007302467.1
wDcitri WP 017532419.1
wOb Wba WP 007302467.1
wMau WP 015588204.1
wNo WP 015588204.1
wBtab WP 127464001.1
wPip Pel WP 007302467.1
wPip Mol WP 007302467.1
wMeg WP 007302467.1
wAlbB WP 006015752.1
wWpum WP 209472700.1
wRec WP 038199468.1
wBic WP 182309626.1
wAna w2.1 WP 121721710.1
wGmm WP 038250204.1
wBai WP 182366257.1
wBif WP 182320007.1
wNfla WP 065094935.1
wNpa WP 065094935.1
wNleu WP 065095494.1
wNfe WP 065094935.1
wAeae GV 2018 7 WP 006279761.1
wYak WP 006279761.1
wRi WP 006279761.1
wCsol MIAO WP 006279761.1
wUni WP 006279761.1
wAra WP 006279761.1
wNon OneA1 WP 006279761.1
wHa WP 006279761.1
wCauA WP 006279761.1
wInc Cu WP 070356734.1
wIrr WP 006279761.1
wCobs-BR2009-alpha WP 006279761.1
wSuzi valsugana WP 006279761.1
wCin2USA1 WP 006279761.1
wCobs-JP2010-OypB WP 006279761.1
wMel WP 006279761.1
wVitA WP 006279761.1
wAeae GV 2018 1 WP 006279761.1

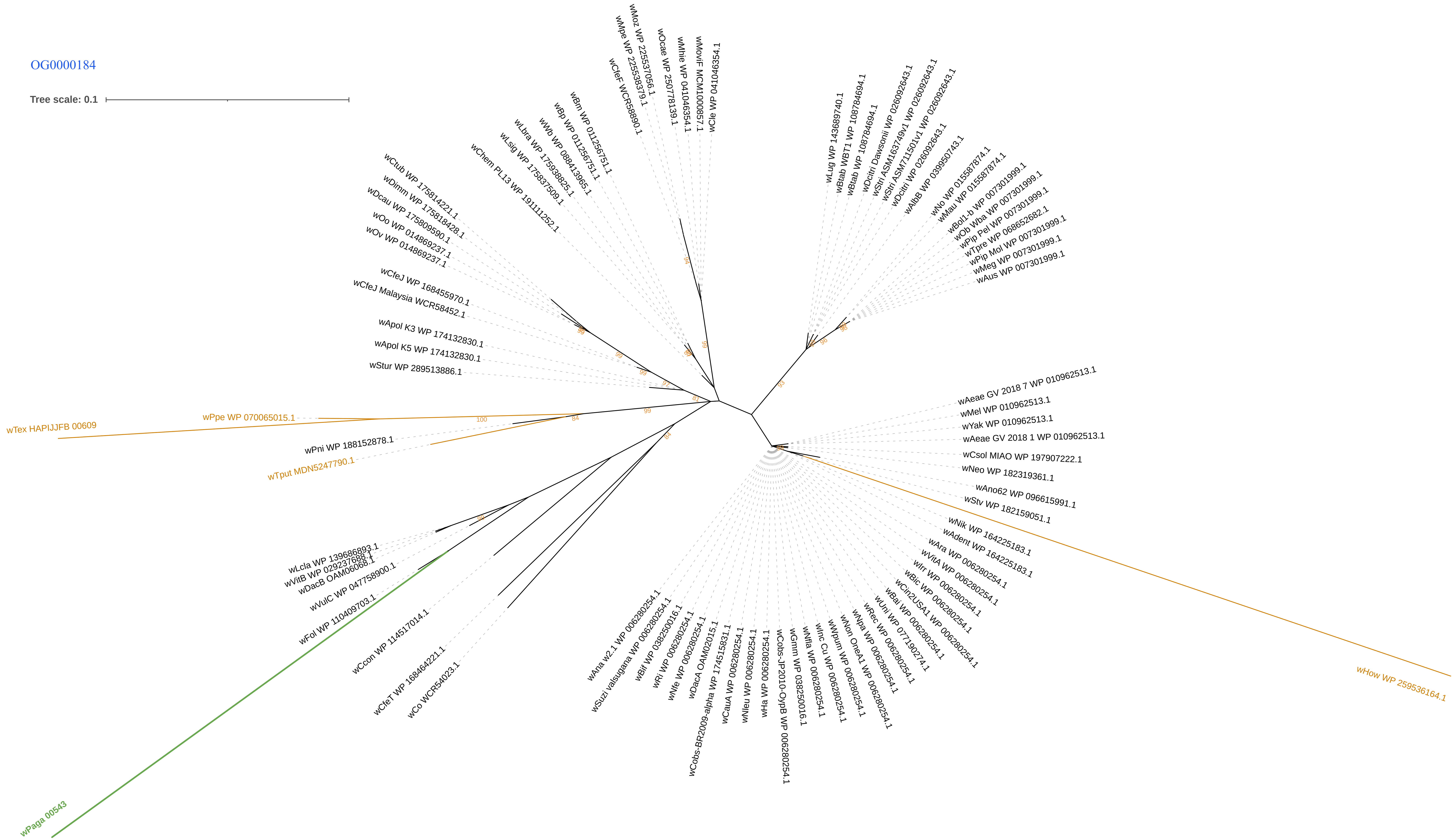
OG0000184
Tree scale: 0.1
wMoz WP 225537056.1
wMpe WP 225538379.1
wCfeF WCR58890.1
wOcae WP 250778139.1
wMhie WP 041046354.1
wMoviF MCM1000857.1
wCle WP 041046354.1
wBm WP 011256751.1
wBp WP 011256751.1
wWb WP 088413965.1
wLbra WP 175938825.1
wLsig WP 175837509.1
wChem PL13 WP 191111252.1
wCtub WP 175814221.1
wDimm WP 175818428.1
wDcau WP 175809590.1
wOo WP 014869237.1
wOv WP 014869237.1
wCfeJ WP 168455970.1
wCfeJ Malaysia WCR58452.1
wApol K3 WP 174132830.1
wApol K5 WP 174132830.1
wStur WP 289513886.1
wPpe WP 070065015.1
wTex HAPIJJFB 00609
wPni WP 188152878.1
wTput MDN5247790.1
wLcla WP 139686893.1
wVitB WP 029237688.1
wDacB OAM06068.1
wVulC WP 047758900.1
wFol WP 110409703.1
wCcon WP 114517014.1
wCfeT WP 168464221.1
wCo WCR54023.1
wPaga 00543
wLug WP 143689740.1
wBtab WBT1 WP 108784694.1
wBtab WP 108784694.1
wDcitri Dawsonii WP 026092643.1
wStri ASM163749v1 WP 026092643.1
wStri ASM711501v1 WP 026092643.1
wDcitri WP 026092643.1
wAlbB WP 039950743.1
wNo WP 015587874.1
wMau WP 015587874.1
wBol1-b WP 007301999.1
wOb Wba WP 007301999.1
wPip Pel WP 007301999.1
wTpre WP 068652682.1
wPip Mol WP 007301999.1
wMeg WP 007301999.1
wAus WP 007301999.1
wAeae GV 2018 7 WP 010962513.1
wMel WP 010962513.1
wYak WP 010962513.1
wAeae GV 2018 1 WP 010962513.1
wCsol MIAO WP 197907222.1
wNeo WP 182319361.1
wAno62 WP 096615991.1
wStv WP 182159051.1
wNik WP 164225183.1
wAdent WP 164225183.1
wAra WP 006280254.1
wVitA WP 006280254.1
wIrr WP 006280254.1
wBic WP 006280254.1
wCin2USA1 WP 006280254.1
wBai WP 006280254.1
wUni WP 077190274.1
wRec WP 006280254.1
wNpa WP 006280254.1
wNon OneA1 WP 006280254.1
wWpum WP 006280254.1
wInc Cu WP 006280254.1
wNfla WP 006280254.1
wGmm WP 038250016.1
wCobs-JP2010-OypB WP 006280254.1
wHa WP 006280254.1
wNleu WP 006280254.1
wCauA WP 006280254.1
wCobs-BR2009-alpha WP 174515831.1
wDacA OAM02015.1
wNfe WP 006280254.1
wRi WP 006280254.1
wBif WP 038250016.1
wSuzi valsugana WP 006280254.1
wAna w2.1 WP 006280254.1
wHow WP 259536164.1

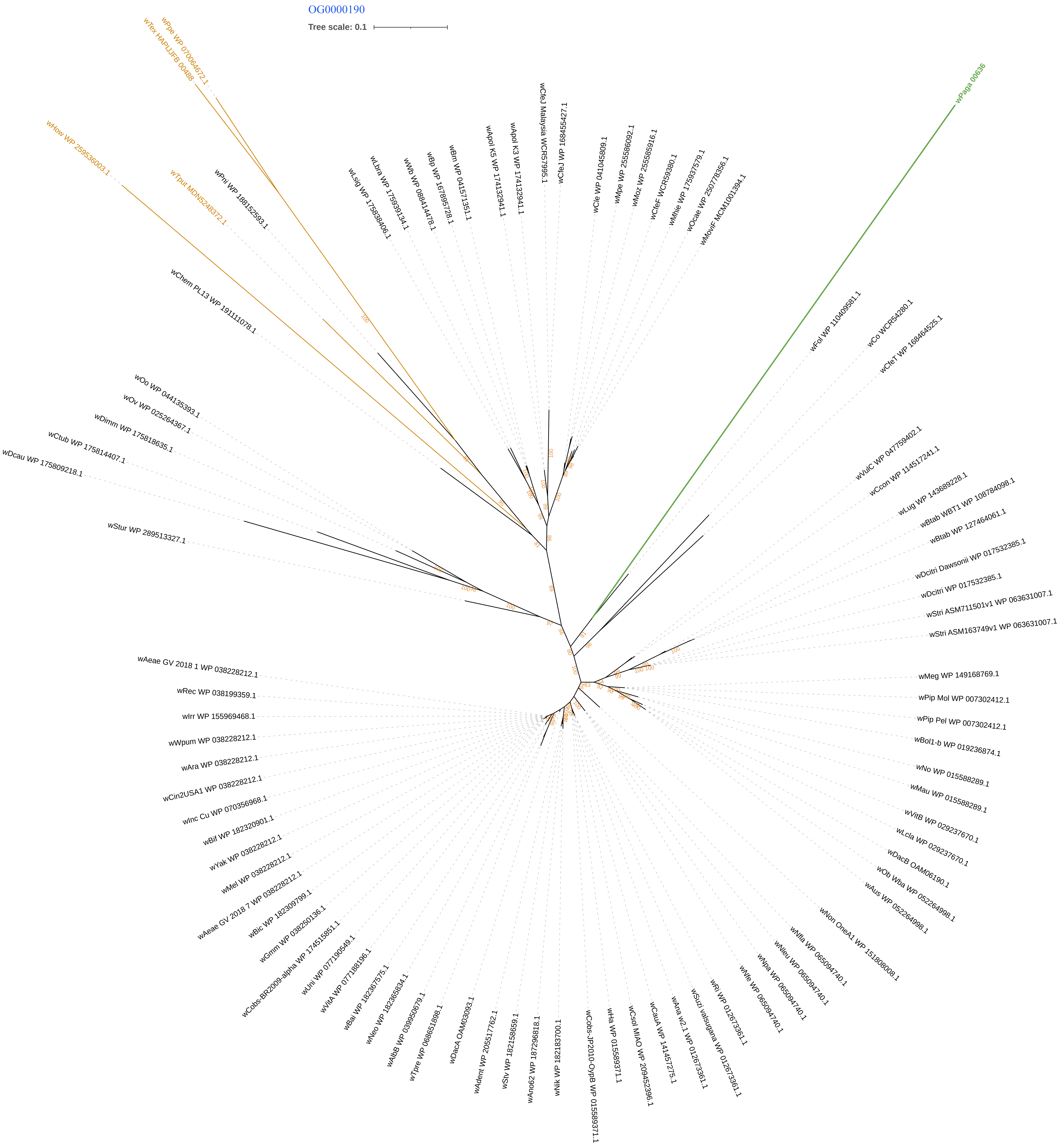
OG0000190
Tree scale: 0.1
wPpe WP 070064672.1
wTex HAPIJJFB 00488
wHow WP 259536003.1
wTput MDN5248372.1
wPni WP 188152593.1
wLsig WP 175838406.1
wLbra WP 175939134.1
wWb WP 088414478.1
wBp WP 167895728.1
wBm WP 041571351.1
wApol K5 WP 174132941.1
wApol K3 WP 174132941.1
wCfeJ Malaysia WCR57995.1
wCfeJ WP 168455427.1
wCle WP 041045809.1
wMpe WP 255586092.1
wMoz WP 255585916.1
wCfeF WCR59380.1
wMhie WP 175937579.1
wOcae WP 250778356.1
wMoviF MCM1001394.1
wPaga 00636
wFol WP 110409581.1
wCo WCR54280.1
wCfeT WP 168464525.1
wChem PL13 WP 191111078.1
wOo WP 044135393.1
wOv WP 025264367.1
wDimm WP 175818635.1
wCtub WP 175814407.1
wDcau WP 175809218.1
wStur WP 289513327.1
wVulC WP 047759402.1
wCcon WP 114517241.1
wLug WP 143689228.1
wBtab WBT1 WP 108784098.1
wBtab WP 127464061.1
wDcitri Dawsonii WP 017532385.1
wDcitri WP 017532385.1
wStri ASM711501v1 WP 063631007.1
wStri ASM163749v1 WP 063631007.1
wMeg WP 149168769.1
wPip Mol WP 007302412.1
wPip Pel WP 007302412.1
wBol1-b WP 019236874.1
wNo WP 015588289.1
wMau WP 015588289.1
wVitB WP 029237670.1
wLcla WP 029237670.1
wDacB OAM06190.1
wOb Wba WP 052264998.1
wAus WP 052264998.1
wNon OneA1 WP 151808008.1
wNfla WP 065094740.1
wNleu WP 065094740.1
wNpa WP 065094740.1
wNfe WP 065094740.1
wRi WP 012673361.1
wSuzi valsugana WP 012673361.1
wAna w2.1 WP 012673361.1
wCauA WP 141457275.1
wCsol MIAO WP 209452396.1
wHa WP 015589371.1
wCobs-JP2010-OypB WP 015589371.1
wNlk WP 182183700.1
wAno62 WP 187296818.1
wStv WP 182158659.1
wAdent WP 205517762.1
wDacA OAM03093.1
wTpre WP 068651898.1
wAlbB WP 039950679.1
wNeo WP 182365834.1
wBai WP 182367575.1
wVitA WP 077188196.1
wUni WP 077190549.1
wCobs-BR2009-alpha WP 174515851.1
wGmm WP 038250136.1
wBic WP 182309799.1
wAeae GV 2018 7 WP 038228212.1
wMel WP 038228212.1
wYak WP 038228212.1
wBif WP 182320901.1
wInc Cu WP 070356968.1
wCin2USA1 WP 038228212.1
wAra WP 038228212.1
wWpum WP 038228212.1
wIrr WP 155969468.1
wRec WP 038199359.1
wAeae GV 2018 1 WP 038228212.1
